# Cofunctional Subpathways Were Regulated by Transcription Factor with Common Motif, Common Family, or Common Tissue

**DOI:** 10.1155/2015/780357

**Published:** 2015-11-24

**Authors:** Fei Su, Desi Shang, Yanjun Xu, Li Feng, Haixiu Yang, Baoquan Liu, Shengyang Su, Lina Chen, Xia Li

**Affiliations:** ^1^College of Bioinformatics Science and Technology, Harbin Medical University, Harbin 150081, China; ^2^Department of Anatomy, Harbin Medical University, Harbin 150081, China; ^3^Department of Computer Science, University of Texas at Dallas, 800 W Campbell Road, Richardson, TX 75080, USA

## Abstract

Dissecting the characteristics of the transcription factor (TF) regulatory subpathway is helpful for understanding the TF underlying regulatory function in complex biological systems. To gain insight into the influence of TFs on their regulatory subpathways, we constructed a global TF-subpathways network (TSN) to analyze systematically the regulatory effect of common-motif, common-family, or common-tissue TFs on subpathways. We performed cluster analysis to show that the common-motif, common-family, or common-tissue TFs that regulated the same pathway classes tended to cluster together and contribute to the same biological function that led to disease initiation and progression. We analyzed the Jaccard coefficient to show that the functional consistency of subpathways regulated by the TF pairs with common motif, common family, or common tissue was significantly greater than the random TF pairs at the subpathway level, pathway level, and pathway class level. For example, HNF4A (hepatocyte nuclear factor 4, alpha) and NR1I3 (nuclear receptor subfamily 1, group I, member 3) were a pair of TFs with common motif, common family, and common tissue. They were involved in drug metabolism pathways and were liver-specific factors required for physiological transcription. In short, we inferred that the cofunctional subpathways were regulated by common-motif, common-family, or common-tissue TFs.

## 1. Introduction

The mechanism of transcriptional regulation of coding genes is one of the basic contents in systems biology. Transcriptional factors (TFs) are proteins that regulate several target genes by binding DNA motifs at the transcriptional level [1–5]. Some investigators have reported that TFs take part in many important biological functions and human diseases, such as cell differentiation, proliferation, immune response, apoptosis, cardiac diseases, and tumor development [6–9]. Dissecting the characteristics of TFs is helpful for understanding their regulatory function in complex biological systems. An increasing number of studies have demonstrated that TFs with similar motifs often (which were defined as common-motif TFs in the paper) recognize target genes with similar expression patterns [10], and in a similar way, TFs with different motifs may directly show different functions [11]. Several researchers have revealed that TFs in the common family (were defined as common-family TFs in the paper) have strong homology and the TFs are functionally related [12]; TFs in the transcription factor family are functional redundancies [13]; and TF family members share a relatively high degree of similarity in genomic structure and gene arrangement [12–14]. Several studies have demonstrated that TFs in the common tissue or cell line (were defined as common-tissue TFs in the paper) play critical roles in disease progression [15].

Currently, the regulatory functional interpretation of TFs mainly relies on activating or inhibiting their target genes [16, 17]. Several studies have revealed the functions of TFs based on the enrichment analysis of their target genes from a number of pathways [18–22]. Subpathways (local regions within an entire biological pathway) exert certain functions on practical biological processes, disease development, and drug treatment [23–27]. Subpathway recognition is crucial because it is capable of focusing on local variation, increasing our power to identify the underlying causal genes, and may give us more detailed explanations of the biological process and pathogenesis of human disease.

In this study, we dissected the regulatory influence of common-motif, common-family, or common-tissue TFs on cofunctional subpathways. Cofunctional subpathways are defined as subpathways that belong to the same pathway or pathway class. To gain insight into the relationships between TFs and their regulatory subpathways, we constructed a bipartite network TF-subpathways network (TSN) to explore the regulatory influence of TFs on subpathways. Through one-way cluster analysis, the common-motif, common-family, or common-tissue TFs that regulated the same pathway classes tended to cluster together, and through Jaccard coefficient analysis, the subpathways regulated by the TFs with common motif, common family, or common tissue were functionally consistent at the subpathway, pathway, and pathway class levels. These findings help us to understand the mechanisms of the regulatory effects of TFs on subpathways and provide a more detailed picture of biological processes and complex human diseases.

## 2. Materials and Methods

### 2.1. Preparation of the Data

#### 2.1.1. TF Information

Transfac Professional (licensed on December 15, 2012) [28] provided details of the relations between TFs and their target genes, verified by biologists. We acquired 4598 transcription relations between 492 human TFs and 1557 target genes. We extracted 1061 human TF binding motifs. We extracted 80 unique TFs belonging to 29 families. We obtained 343 unique TFs expressed on 122 unique human tissues or cell lines.

#### 2.1.2. Subpathway Data

We used R package of iSubpathwayMiner [24] to find the subpathways in public Kyoto Encyclopedia of Genes and Genomes (KEGG) (http://www.kegg.jp/) pathways database [29]. According to the pathway classification in KEGG, all subpathways were grouped into cellular processes (CP), environmental information processing (EIP), human diseases (HD), metabolism (Met), and organismal systems (OS).

### 2.2. Identifying the Subpathways Regulated by TFs

Cumulative hypergeometric enrichment analysis was used for identification of subpathways, in which TF target genes were significantly enriched. The *p* value can be calculated to evaluate the enrichment significance for that subpathway as follows:

(1) $$\begin{array}{c} p=1-\overset{r-1}{\underset{x=0}{\sum }}\frac{\left(\begin{array}{c} t\\ x \end{array}\right)\left(\begin{array}{c} m-t\\ n-x \end{array}\right)}{\left(\begin{array}{c} m\\ n \end{array}\right)}. \end{array}$$

We supposed that the whole human genome had *m* genes, and among those, *t* genes were included in the subpathway. The number of target genes of one TF is *n*, of which *r* are involved in the same subpathways.

### 2.3. Evaluating the Consistent Function of Subpathways

We proposed a hypothesis that one TF pair regulated the consistent functional subpathways if they had common motif, common family, or common tissue. We applied the Jaccard coefficient to evaluate the consistent function of subpathways for TF pairs with a common motif, common family, or common tissue. The Jaccard coefficient is

(2) $$\begin{array}{c} J\left(\;X,Y\;\right)=\frac{\left|X\cap Y\right|}{\left|X\cup Y\right|}. \end{array}$$

Assume TF1 and TF2 are a pair of common-motif, common-family, or common-tissue TFs, where *X* represents the subpathways regulated by TF1 and *Y* represents the subpathways regulated by TF2. *J* is the number of intersections of *X* and *Y* divided by the number of unions of *X* and *Y*. The range of Jaccard coefficient, *J*, is from 0 to 1. If TF1 and TF2 annotated the same subpathway set, then *J* = 1, and if TF1 and TF2 annotated entirely different subpathways, then *J* = 0. The higher Jaccard value means stronger similarity, while lower values mean weaker similarity, so we can use the Jaccard coefficient to evaluate the consistent functionality of subpathways regulated by a pair of TFs with common motif, common family, or common tissue. At the pathways or pathway classes level, Jaccard coefficient is also used to evaluate the consistent functionality of subpathways, where *X* represents the pathways or pathway classes that include the subpathways regulated by TF1 and *Y* represents the pathways or pathway classes that include the subpathways regulated by TF2.

## 3. Results

### 3.1. TF-Subpathway Network (TSN)

**Figure 1:**
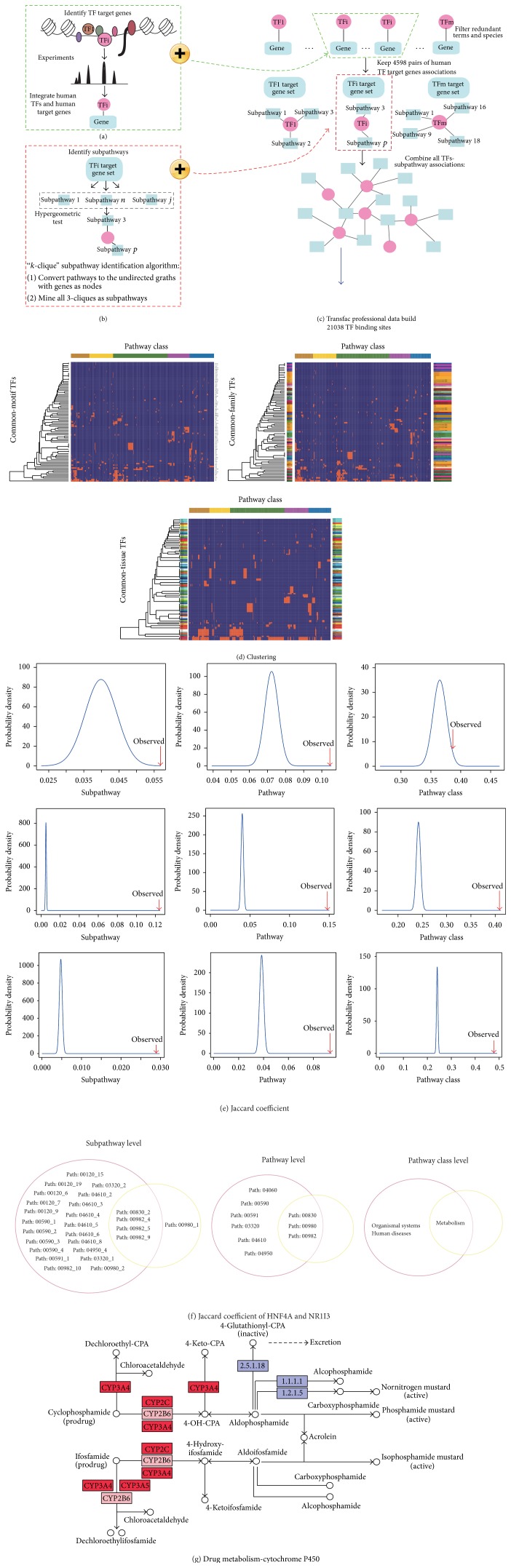
Schematic of the construction of the global TSN based on TF target genes and pathway structure data from KEGG. (a) We extracted 21038 TFs that were verified through biology experiment from Transfac Professional database and then filtered redundant terms and species and obtained 4598 pairs of human TF target gene associations. (b) We used the *k*-clique subpathway method to identify significantly enriched subpathways to generate TF-subpathway associations. (c) We combined these TF-subpathway associations to form the TSN. (d) We used one-way clustering between TFs and subpathways to analyze the regulatory effects of common-motif, common-family, and common-tissue TFs on cofunctional subpathways. (e) Jaccard coefficient was applied to evaluate the functional consistency of the common-motif, common-family, and common-tissue TFs at the subpathway, pathway, and pathway class levels. (f) HNF4A and NR1I3 were a pair of TFs with common motif, common family, and common tissue. Jaccard coefficient at subpathways level, pathways level, and pathway classes level. (g) HNF4A regulated subpathway 00982-10, and NR1I3 regulated subpathway 00982-4, and they have shared genes (CYP3A4, CYP3A5, and CYP2C).

**Figure 2:**
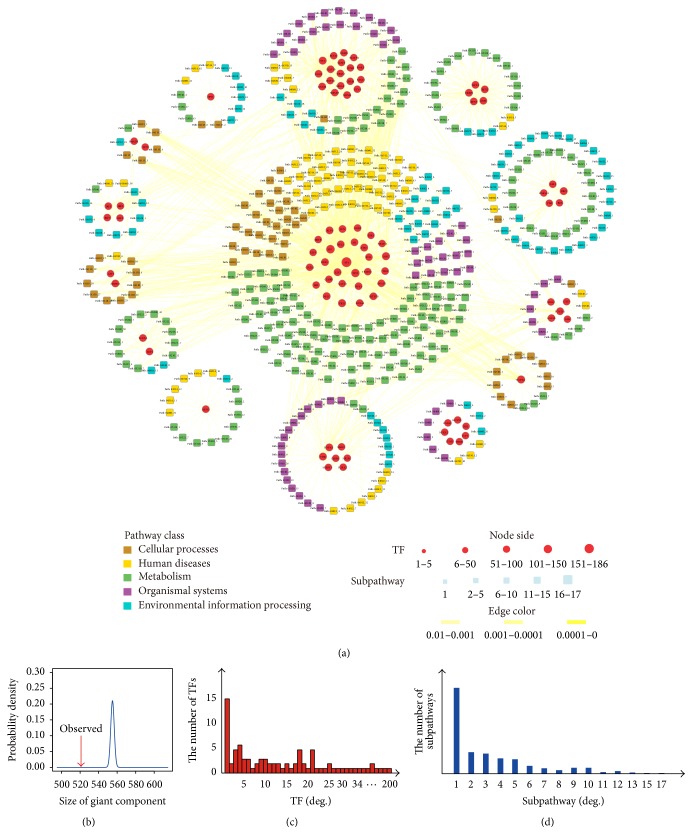
The TSN is a bipartite network. (a) The circles and rectangles in the network represent TFs and subpathways, respectively. A pair of TFs and a subpathway were connected by an edge if the set of target genes of TF was significantly enriched in the corresponding subpathway. Node size was proportional to the degree of the node. Edges were colored according to the enrichment significance (*p* < 0.01) of associations between TF target genes and subpathways. (b) Component size distributions of TFs and subpathways in the TSN and random network. The actual size of the real network giant component was smaller than that of the 1000 randomly shuffled TFs (521 versus 554.9630, experiential *p* = 0). (c) Bar plot of degree of TF distribution in the TSN. (d) Bar plot of degree of distribution of subpathways in TSN.

We constructed a global TSN based on TFs and subpathway data (Figure 1) and systematically analyzed the characteristics of TFs that affected subpathways. We obtained all terms of TF binding sites (*n* = 21 038) information from the authoritative Transfac database. After filtering, we obtained 4598 specific human transcriptional regulatory relations between 492 TFs and 1557 target genes, verified by biologists. For each TF, we used the “*k*-cliques” subpathway identification method provided by the iSubpathwayMiner software package to identify significantly enriched subpathways based on the TF-regulated target gene set, when *k* = 3 and *p* < 0.01. *k* = 3 meant that the distance among the genes in one subpathway was not greater than 3 to ensure that genes in the subpathways have highly similar functions. We obtained 1490 significant TF-subpathway associations between 89 TFs and 468 subpathways (Table S1 in Supplementary Material available online at http://dx.doi.org/10.1155/2015/780357). We constructed a bipartite network consisting of TFs and subpathways in which a TF and a subpathway were connected if the TF target genes were significantly enriched in the corresponding subpathways (Figure 2).

The TSN was composed of 557 nodes (468 subpathways and 89 TFs) and 1490 edges (Figure 2(a)). We calculated the size of the giant component of TSN (Figure 2(b)). The giant component was the largest connected component of a network and measured local functional clustering when compared to random networks. The TSN network connected most TFs and subpathways into a highly interlinked giant component, with strong local clustering of TFs and subpathways. We constructed 1000 random networks by randomly shuffling the relations between the 1490 pairs of TFs and subpathways, while keeping the number of each TF and subpathway unchanged. We compared the giant component of the real TSN and 1000 random networks, which showed that the actual size of the giant component of the TSN (521) was significantly smaller (experiential *p* = 0) than the average giant component of randomized networks (554.963).

We paid close attention to the TF degree distribution. As shown in Figure 2(c), a small number of TFs had higher connectivity, which meant that these TFs regulated many subpathways. For example, SP1 had the highest degree (186) (Table S2). This might be because it could activate or inhibit the expression of a number of essential oncogenes and tumor suppressors, and SP1 took part in the main biological processes such as cell growth, apoptosis, differentiation, immune response, response to DNA damage, and chromatin remodeling [30, 31]. Tumor suppressor gene TP53 had higher degree (72) than other TFs, and it is always present in many types of human cancer. For example, TP53 gene mutations are more frequent in cervical adenocarcinoma [32]. In addition, we also calculated the degree distribution of the subpathways (Figure 2(d)). We evaluated which kind of subpathway was preferentially regulated by TFs. We grouped all the subpathways into the categories of Met, OS, CP, HD, and EIP, according to the pathway classification of KEGG. The average degrees of the five subpathway groups were 4.0000, 2.6026, 5.2982, 3.0372, and 1.9250 (*p* = 2.77 × 10^−11^ by one-way ANOVA test), respectively. Therefore, the cellular processes subpathways had the greatest opportunity of being regulated by TFs, such as the cell cycle subpathway (path: 04110_18) and TP53 signaling pathway (path: 04115_1). This finding was consistent with a previous study that showed that TF preferred to regulate cellular process genes [33].

### 3.2. Common-Motif TFs Tend to Regulate Cofunctional Subpathways

The common conserved sequence of the TF binding sites is called a motif. Some studies have indicated that motif sequence is associated with TF function. TFs with similar motifs often recognize target genes with similar expression patterns [10], and TFs with different motifs may directly influence different TF function [11], and TFs influence pathways initiated by specific DNA-binding motifs [34]. To explore whether the common-motif TFs tended to regulate cofunctional subpathways, we extracted 1061 human DNA motifs from the Transfac database. We obtained 15 150 pairs of TFs with similar DNA motifs (using STAMP software (http://www.benoslab.pitt.edu/stamp) [35] with *e* value < 0.01), of which 610 pairs (83 unique TFs in the TSN) had similar DNA motifs. These TFs annotated 436 subpathways, forming 1402 relations (Table S3).

**Figure 3:**
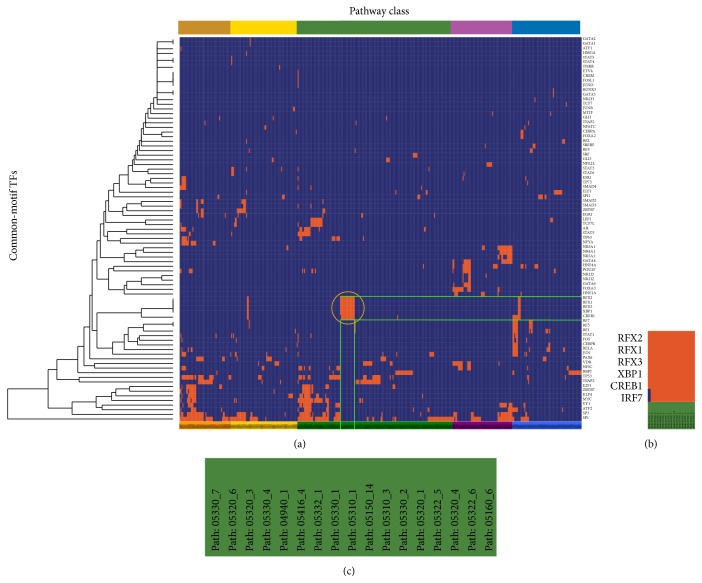
One-way clustering to TFs with common motif of the TSN. (a) One-way clustering between 83 TFs and 436 subpathways. The corresponding cell was colored orange if there was an edge between the TF and subpathway in the TSN. Subpathway labels were colored according to the pathway class colors used in Figure 2. (b) Zoom-in plot of part of the orange circled region in (a), showing the TFs with similar motif and pathway class. (c) Zoom-in plot of part of the subpathway labels in the lower-right region of (a).

To examine whether common-motif TFs regulated the cofunctional subpathways, we applied one-way clustering to the 83 unique TFs and 436 unique subpathways (Figure 3(a)). The common-motif TFs that regulated the same pathway classes tended to group together. For example, IRF7 (the interferon regulatory factor 7) driven regulatory cascade in which genetic variation on chromosome 15q25 leads to type 1 diabetes (pathway: 04940) [36] and XBP1 (X-box binding protein 1) induce pancreatic beta cell dysfunction and apoptosis of type 1 diabetes [37] (Figure 3(b)). That is, common-motif TFs tend to regulate the same pathway classes and lead to the same disease.

**Figure 4:**
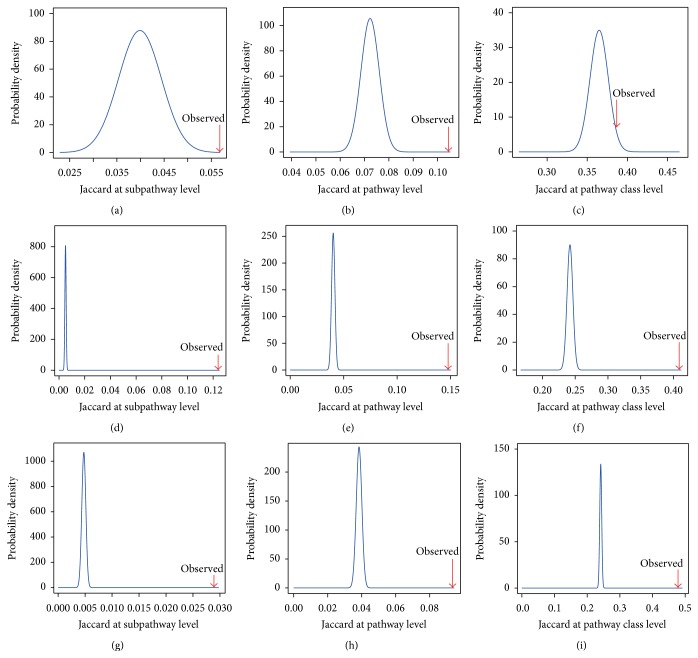
Jaccard coefficient. (a) The average Jaccard coefficient of common-motif TF pairs in TSN (0.0568) was significantly greater than that of 1000 times randomly shuffled TFs (0.0399) at the subpathway level (experiential *p* = 0.007). (b) The average Jaccard coefficient of real common-motif TF pairs was significantly higher than that of random TF pairs (0.1045 versus 0.0723, experiential *p* = 0) at the pathway level. (c) The average Jaccard coefficient of real common-motif TF pairs was significantly higher than that of random TF pairs (0.3867 versus 0.3649, experiential, *p* = 0.005) at the pathway class level. The result implied that TFs with similar DNA motifs regulated the cofunctional subpathway. (d) The average Jaccard coefficient of real common-family TF pairs in TSN (0.1239) was significantly higher than that of random TF pairs (0.005) at the subpathway level (experiential *p* = 0). (e) The average Jaccard coefficient of real common-family TF pairs in TSN (0.1478) was significantly higher than that of random TF pairs (0.0402) at the pathway level (experiential *p* = 0). (f) The average Jaccard coefficient of real common-family TF pairs in TSN (0.4088) was significantly higher than that of random TF pairs (0.2420) at the pathway class level (experiential *p* = 0). The result implied that common-family TFs regulated the cofunctional subpathway. (g) The average Jaccard coefficient of real common-family TF pairs in TSN (0.0289) was significantly higher than that of random TF pairs (0.0048) at the subpathway level (experiential *p* = 0). (h) The average Jaccard coefficient of real common-family TF pairs in TSN (0.0935) was significantly higher than that of random TF pairs (0.0386) at the pathway level (experiential *p* = 0). (i) The average Jaccard coefficient of real common-family TF pairs in TSN (0.4779) was significantly higher than that of random TF pairs (0.2413) at the pathway class level (experiential *p* = 0).

**Figure 5:**
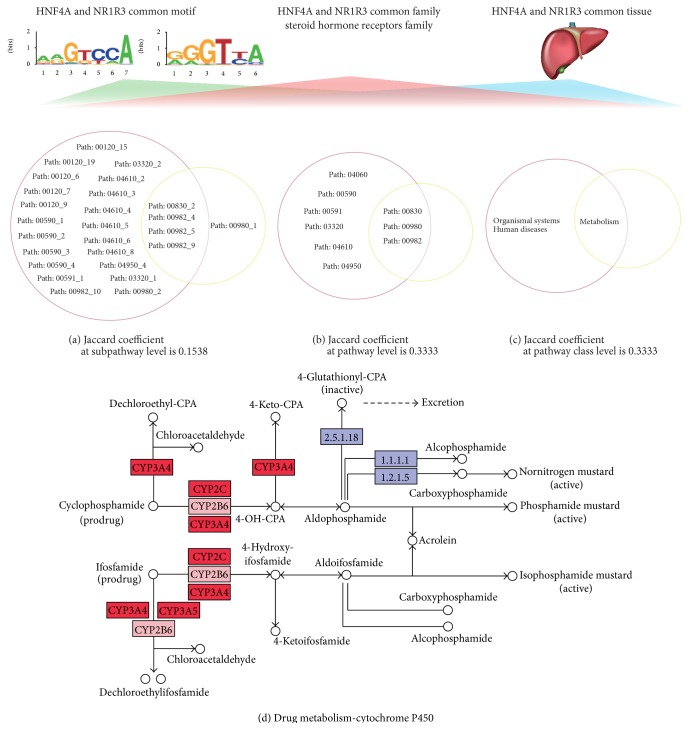
Common-motif, common-family, and common-tissue TF pair. HNF4A and NR1I3 are a pair of TFs with a common motif, common family (steroid hormone receptors), and common tissue (liver). (a) Jaccard coefficient at the subpathway level of HNF4A and NR1I3 was 0.1538. (b) Jaccard coefficient at the pathway level was 0.3333. (c) Jaccard coefficient at the pathway class level was 0.3333. (d) HNF4A regulated subpathway 00982-10, and NR1I3 regulated subpathway 00982-4. Red genes represent the shared genes between subpathways 00982-10 and 00982-4. Pink genes represent the genes only in subpathway 00982-4. Purple nodes represent the genes in the entire pathway 00982.

To further investigate the effect of the common motif TFs on subpathway, we randomly shuffled the 610 pairs of common-motif TFs 1000 times, while keeping the number of each TF unchanged. The average Jaccard coefficient of the real common-motif TF pairs was significantly higher than the average value for the 1000 times randomly shuffled TF pairs at the subpathway level. The mean Jaccard coefficient of the real common-motif TF pairs was 0.0568 compared with 0.0399 for the randomly shuffled TF pairs (experiential *p* = 0.007, Figure 4(a)). The average Jaccard coefficient of the real common-motif TF pairs was significantly higher than for the randomly shuffled TF pairs (0.1045 versus 0.0723, experiential *p* = 0, Figure 4(b)) at the pathway level. The average Jaccard coefficient of the real common-motif TF pairs was significantly higher than that of the randomly shuffled TF pairs (0.3867 versus 0.3649, experiential *p* = 0.005, Figure 4(c)) at the pathway class level. These results revealed a high degree of consistent functionality of subpathways regulated by the common-motif TFs. Thus, the TSN can help us explain the consistent function of the subpathways regulated by the common-motif TFs. For example, HNF4A (hepatocyte nuclear factor 4, alpha) and NR1I3 (nuclear receptor subfamily 1, group I, member 3) have similar DNA-binding motifs (using STAMP software [35] with *e* value < 0.01) (Figure 5), and the Jaccard coefficient at the subpathway level was 0.1538 (Figure 5(a)); the Jaccard coefficient at the pathway level was 0.3333 (Figure 5(b)); the Jaccard coefficient at the pathway class level was 0.3333 (Figure 5(c)). They, respectively, regulated subpathways 00982_4 and s 00982-10, and these subpathways belonged to the same drug metabolism-cytochrome P450 pathway (pathway: 00982, Figure 5(d)) [38]. HNF4A and NR1I3 regulate the drug-metabolizing enzyme cytochrome P450 3A4 (CYP3A4) expression. HNF4A is critically involved in the NR1I3-mediated transcriptional activation of CYP3A4. CYP3A4 is thought to be involved in the metabolism of nearly 50% of all the drugs currently prescribed, and alteration in the activity or expression of this enzyme seems to be a key predictor of drug responsiveness and toxicity [38]. Recent studies have identified HNF4A sites in the CYP2C8 and CYP2C9 promoter. Silencing HNF4A reduces the constitutive expression of CYP2C8 and CYP2C9. CYP2C enzymes are expressed constitutively and comprise about 20% of the total cytochrome P450 in human liver [39]. Constitutive androstane receptor NR1I3 activates CYP2B6, CYP2C9, and CYP3A4 expression to increase metabolic capability [40].

### 3.3. Common Family TFs Tend to Regulate Cofunctional Subpathways

TFs in a common family had similar structure. Some researchers have revealed functional redundancies of the TF family, and common-family TFs show functional similarity [12–14]. Whether common-family TFs tend to regulate the cofunctional subpathways was still unknown. We extracted 80 unique TFs belonging to 28 families from the Transfac database, and these TFs regulated 419 unique subpathways from five pathway classes in KEGG. Eighty unique TFs and 419 unique subpathways formed 1353 relations (Table S4).

**Figure 6:**
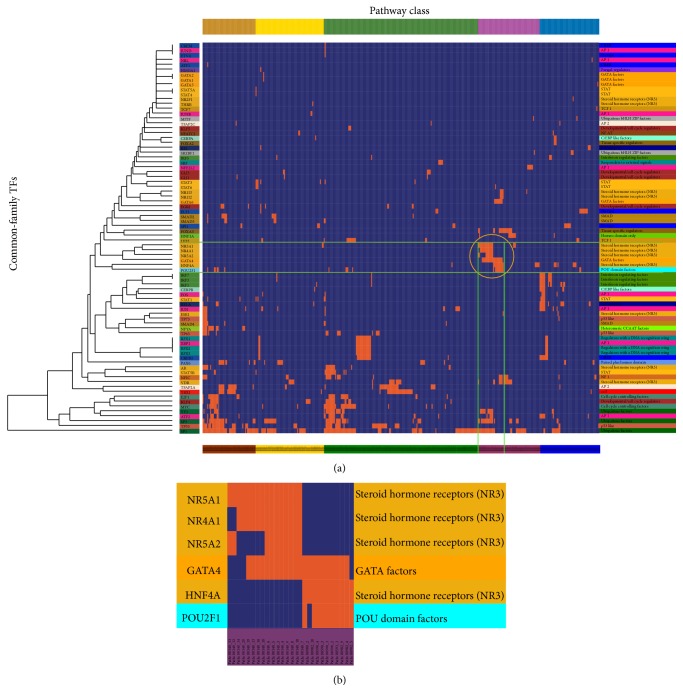
One-way clustering to TFs with common family of the TSN. (a) One-way clustering between 80 TFs and 419 subpathways. The corresponding cell was colored orange if there was an edge between the TF and subpathway in the TSN. Subpathway labels were colored according to the pathway class colors used in Figure 2. (b) Zoom-in plot of part of the orange circled region in (a), showing the common family TFs and subpathways.

To test the functional consistency of subpathways regulated by the common-family TFs, we performed one-way clustering to the 80 TFs and 419 subpathways (Figure 6(a)). The TFs that regulated the same pathway classes tended to gather in the same families at the local level. For example, the steroid hormone receptors family of TFs (NR5A1, NR4A1, and NR5A2) regulated the steroid hormone biosynthesis pathway (pathway: 00140, Figure 6(b)). That is, common-family TFs tended to regulate the cofunctional subpathways. This local clustering phenomenon may be interpreted by the small average number of TFs (2.86) in each family.

To further analyze the effects of the common-family TFs on subpathways, we applied the Jaccard coefficient to evaluate the consistent functionality of subpathways regulated by pairs of TFs with common family. We randomly shuffled the family information of TFs 1000 times and kept the number of each TF (80 unique TFs) and family (28 unique families) unchanged. The average Jaccard coefficient of real common-family TF pairs was greater than that of the randomly shuffled TF pairs (0.1239 versus 0.0050, experiential *p* = 0, Figure 4(d)) at the subpathway level. The average Jaccard coefficient of real common-family TF pairs was significantly greater than that of the randomly shuffled TF pairs (0.1477 versus 0.0402, experiential *p* = 0, Figure 4(e)) at the pathway level. The average Jaccard coefficient of real common-family TF pairs was significantly greater than that of randomly shuffled TF pairs (0.4088 versus 0.2420, experiential *p* = 0, Figure 4(f)) at the pathway class level. These results indicated that common-family TFs regulate cofunctional subpathways. For example, HNF4A and NR1I3 appeared in the common steroid hormone receptors TF family, and they, respectively, regulated subpathways 0098-24 and 00982-10, and these subpathways belonged to the same drug metabolism-cytochrome P450 pathway (Figure 5).

### 3.4. Common-Tissue TFs Tend to Regulate Cofunctional Subpathways

TFs are always expressed in specific tissues or cell lines. Common-tissue TFs have functional consistency [15]. We wanted to explore the consistent function of subpathways regulated by the common-tissue TFs. We obtained 1464 relations between 122 unique tissues or cell lines and 343 unique TFs, of which 47 unique TFs were from 45 unique tissues and enriched in 385 unique subpathways in the TSN. So, there are 3494 relations between 47 TFs and 385 subpathways (Table S5). We retained tissues or cell types that included at least three unique TFs and obtained 44 unique TFs belonging to 21 tissue types, and these TF target genes were located in 378 unique subpathways, forming 2339 relations between TFs and subpathways.

**Figure 7:**
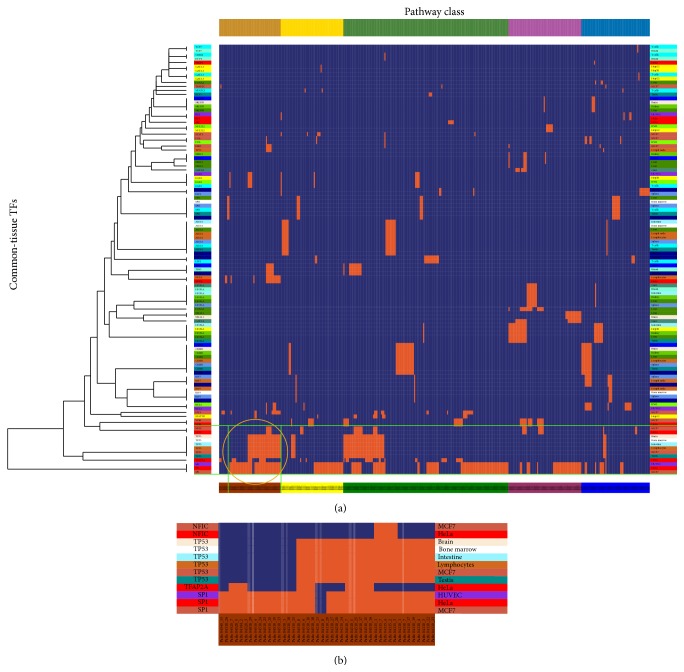
One-way clustering to TFs with common tissue of the TSN. (a) One-way clustering between 44 unique TFs and 378 unique subpathways. The corresponding cell was colored orange if there was an edge between the TF and subpathway in the TSN. Subpathway labels were colored according to the pathway class colors used in Figure 2. (b) Zoom-in plot of part of the orange circled region in (a), showing the common tissue TFs and subpathways.

To examine whether common-tissue TFs regulated the cofunctional subpathways, we applied one-way clustering to the 44 unique TFs and 378 unique subpathways (Figure 7(a)). The TFs that regulated the same pathway classes tended to gather in the same tissues at the local level. For example, SP1 and TP53 were expressed in MCF7 cells (Figure 7(b)), increasing binding of SP1 complex to the TP53 promoter region, which enhanced expression of tumor suppressor factor TP53 and led to breast cancer cell apoptosis (pathway: 04210) [41]. This means that cofunctional subpathways regulated by common-tissue TFs tended to form many small clusters. This local clustering phenomenon might be explained by the following reasons: (i) the average amount of TF (5.35) related to tissues or cell lines was small; (ii) many TFs belonged to different tissue or cell types; and (iii) some special tissue TFs had a fundamental biological function and took part in many basic functions [42].

To test the effects of common-tissue TFs on subpathways in a general sense, we calculated the Jaccard coefficient for pairs of common-tissue TFs. We randomly shuffled the tissue information of TFs (47 unique TFs and 45 unique tissues) 1000 times and kept the number of each TF unchanged. The average Jaccard coefficient of real common-tissue TF pairs was significantly greater than that of randomly shuffled TF pairs at the subpathway level (0.0289 versus 0.0048, experiential *p* = 0, Figure 4(g)). The average Jaccard coefficient of real common-tissue TF pairs was significantly greater than that of randomly shuffled TF pairs at the pathway level (0.0935 versus 0.0386, experiential *p* = 0, Figure 4(h)). The average Jaccard coefficient of real common-tissue TF pairs was significantly greater than that of randomly shuffled TF pairs at the pathway class level (0.4779 versus 0.2413, experiential *p* = 0, Figure 4(i)). We conclude that common-tissue TFs tend to regulate the consistent functional subpathway at different functional levels. For example, HNF4A and NR1I3 were expressed in the liver (Figure 5), where they, respectively, regulated subpathways 00982-4 and subpathways 00982-10, which belonged to the same drug metabolism-cytochrome P450 pathway. HNF4A and NR1I3 regulated CYP3A4, and CYP3A4 promoter activity was most pronounced in liver cells, indicating that CYP3A4 is a liver-specific factor that is required for physiological transcriptional response [38]. Constitutive androstane receptor NR1I3 is predominantly expressed in the liver and it activates CYP2B6, CYP2C9, and CYP3A4 expression to increase metabolic capability [40].

## 4. Discussion and Conclusion

To gain insight into the influence of TF characteristics on their regulatory subpathways, we constructed a TSN using the TF target gene associations and *k*-clique subpathway identification method. We used the *k*-clique (*k* = 3) method to divide large pathways into multiple subpathways. This ensured that the identified TF-regulated subpathways were located in the TF-related local regions of the pathways and also increased the tendency for genes in a subpathway to share similar biological functions and be involved in similar biological processes. To date, most pathway identification methods have only identified entire pathways without considering the scale of these pathways. However, each pathway has obviously different scales. These differences in scale hinder the evaluation of TF-related pathways from global networks and the identification of TF-related subpathways that are usually located in the local regions of the pathways. By using the subpathway identification method, we ensured that each subpathway was represented on a small scale.

Through our analyses of the TSN, we found that the cofunctional subpathways were regulated by the common-motif, common-family, or common-tissue TFs. We performed one-way clustering on the TSN based on common-motif, common-family, and common-tissue TFs. The results showed that all of the TFs with three types of characters that regulated the same pathway classes tended to cluster together. Furthermore, we applied the Jaccard coefficient to establish whether common-motif, common-family, and common-tissue TFs regulate cofunctional subpathways. Our results provided strong support for cofunctional subpathways that were regulated by TFs with three types of characters. For example, HNF4A and NR1I3 were a pair of TFs with a common motif, common family (steroid hormone receptors), and common tissue (liver). HNF4A and NR1I3 were expressed in the liver and, respectively, regulated subpathways 00982-4 and 00982-10, which belonged to the same drug metabolism-cytochrome P450 pathway. HNF4A and NR1I3 regulated expression of the drug-metabolizing enzyme cytochrome P450 3A4 (CYP3A4) expression [28]. HNF4A is critically involved in the NR1I3-mediated transcriptional activation of CYP3A4, which is thought to be involved in the metabolism of nearly 50% of all the drugs currently prescribed. Alteration in the activity or expression of CYP3A4 seems to be a key predictor of drug responsiveness and toxicity. CYP3A4 promoter activity was most pronounced in liver cells, indicating that CYP3A4 is a liver-specific factor that is required for physiological transcriptional response [38]. Recent studies have identified HNF4A sites in the CYP2C8 and CYP2C9 promoter. Silencing HNF4A reduces constitutive expression of CYP2C8 and CYP2C9, as shown by quantitative polymerase chain reaction analysis [39]. Constitutive androstane receptor NR1I3 activated CYP2B6, CYP2C9, and CYP3A4 expression to increase metabolic capability [40]. We further discussed the clinical or cell development study of HNF4A and NR1I3. We increased the following four aspects of analysis. First, we downloaded the large-scale liver cancer RNAseq data from TCGA public database (http://cancergenome.nih.gov/); we used the MARS method of R package “DEGseq” to analyze the 423 samples (373 liver cancer samples and 50 normal samples) and found HNF5A (*q* value < 1.0*e* − 6) and NR1I3 (*q* value < 1.0*e* − 6) were significantly differentially expressed in the liver cancer dataset; thus, they may be used for the study of liver cancer. Second, we searched PubMed database and confirmed that HNF4 and NR1I3 were associated with liver cancer. Previous studies have demonstrated that downregulation of HNF4A was associated with hepatocellular carcinoma (HCC) progression in rodents and humans. In addition, the study of Huang et al. suggested that NR1I3 was a primary regulator of drug metabolism and detoxification and NR1I3 activation or transient strictly limited liver growth [43–45]. Third, we extracted the direct neighbours of this pair of TFs in the protein-protein interaction network and performed the pathway enrichment analysis by DAVID (https://david.ncifcrf.gov/). Results suggested that these genes were significantly enriched in some well-known clinical or cell development related pathways of KEGG such as “pathways in cancer” (hsa05200, *p* = 1.33*e* − 04) and “cell cycle pathway” (hsa04110, *p* = 2.88*e* − 04). Finally, we tested the Pearson correlation coefficient of this pair of TFs, and the result showed that they were significant positive correlation (*r* = 0.4257, *p* < 2.2*e* − 16), which indicated that these two TFs may collectively regulate their downstream target genes; thus, they played important role in the initiation and progression of liver cancer. In summary, the above analysis result indicated that this pair of TFs (HNF4A and NR1I3) may be potential clinical biomarkers of liver cancer.

To confirm the validity of our results, we also constructed the TSN with *k* = 4 (*p* < 0.01) (Table S6–S9). First, we compared the two networks and found that the two networks were robust. There were 1470 edges between 99 TFs and 343 subpathways in the TSN with *k* = 4, while there were 1490 edges between 89 TFs and 468 subpathways in the TSN with *k* = 3. The overlap of TFs (86 TFs) in the two networks was statistically significant (hypergeometric test *p* < 1.0*e* − 6). In the TSN with *k* = 4, there were 343 subpathways corresponding to 95 pathways, and the overlap of pathways (87 pathways) in the two networks was statistically significant (hypergeometric test *p* < 1.0*e* − 6). Thus, these two networks were robust. We then repeated some of the analyses and found that the results of the two networks were similar. We compared one-way clustering results of the two networks and found that the results of two networks were similar. The clustering results of TSN with *k* = 4 (Figures S1, S2, and S3) showed that the subpathways regulated by TFs with common motif, common family, and common tissue tended to cluster in the same pathway class. In particular, we verified the consistent function of subpathways regulated by the TFs with common motif, common family, and common tissue by calculating Jaccard coefficient and found the results of two networks were similar. In the TSN with *k* = 4, the average Jaccard coefficient of the real common-motif, common-family, and common-tissue TF pairs was significantly higher than the random TF pairs by shuffling TSN 1000 times at the subpathway level, pathway level, and pathway class level (Table S10). As a control, TSN with *k* = 4 presented the similarity of results when compared with that of *k* = 3. These results indicated that our conclusions were robust in different threshold of subpathway networks. In summary, our study is significant for understanding the TFs underlying the regulatory function in complex biological systems.

## Supplementary Material

Figure S1. One-way clustering to TFs with common motif of the TSN with k=4. A. One-way clustering between 90 TFs and 320 subpathways. The corresponding cell was colored orange if there was an edge between the TF and subpathway in the TSN with k=4. Subpathway labels were colored according to the pathway class colors used in Figure 2.
Figure S2. One-way clustering to TFs with common family of the TSN with k=4. A. One-way clustering between 78 TFs and 239 subpathways. The corresponding cell was colored orange if there was an edge between the TF and subpathway in the TSN with k=4. Subpathway labels were colored according to the pathway class colors used in Figure 2.
Figure S3. One-way clustering to TFs with common tissue of the TSN with k=4. A. One-way clustering between 60 unique TFs and 269 unique subpathways. The corresponding cell was colored orange if there was an edge between the TF and subpathway in the TSN with k=4. Subpathway labels were colored according to the pathway class colors used in Figure 2.
Table S1. The detailed information of the significant TF-subpathway associations between TFs and subpathways in the TSN.
Table S2. The detailed information of the TFs degree distribution in the TSN.
Table S3. The detailed information of the co-motif TFs regulated sub-pathways.
Table S4. The detailed information of the co-family TFs regulated sub-pathways.
Table S5. The detailed information of the co-tissue TFs regulated sub-pathways.
Table S6. The detailed information of the significant TF-subpathway associations between TFs and subpathways in the TSN with k=4.
Table S7. The detailed information of the co-motif TFs regulated sub-pathways of TSN with k=4.
Table S8. The detailed information of the co-family TFs regulated sub-pathways of TSN with k=4.
Table S9. The detailed information of the co-tissue TFs regulated sub-pathways of TSN with k=4.
Table S10. Real vs. random Jaccard coefficient of the TSN with k=4 at the every pathway level.
