# Supplementary material for: Cofunctional Subpathways Were Regulated by Transcription Factor with Common Motif, Common Family, or Common Tissue
Source: Biomed Res Int. 2015 Nov 24;2015:780357. doi: 10.1155/2015/780357 (PMC4672121; doi:10.1155/2015/780357)
Supplement: Supplementary file 1 — Figure S1. One-way clustering to TFs with common motif of the TSN with k=4. A. One-way clustering between 90 TFs and 320 subpathways. The corresponding cell was colored orange if there was an edge between the TF and subpathway in the TSN with k=4. Subpathway labels were colored according to the pathway class colors used in Figure 2. Figure S2. One-way clustering to TFs with common family of the TSN with k=4. A. One-way clustering between 78 TFs and 239 subpathways. The corresponding cell was colored orange if there was an edge between the TF and subpathway in the TSN with k=4. Subpathway labels were colored according to the pathway class colors used in Figure 2. Figure S3. One-way clustering to TFs with common tissue of the TSN with k=4. A. One-way clustering between 60 unique TFs and 269 unique subpathways. The corresponding cell was colored orange if there was an edge between the TF and subpathway in the TSN with k=4. Subpathway labels were colored according to the pathway class colors used in Figure 2. Table S1. The detailed information of the significant TF-subpathway associations between TFs and subpathways in the TSN. Table S2. The detailed information of the TFs degree distribution in the TSN. Table S3. The detailed information of the co-motif TFs regulated sub-pathways. Table S4. The detailed information of the co-family TFs regulated sub-pathways. Table S5. The detailed information of the co-tissue TFs regulated sub-pathways. Table S6. The detailed information of the significant TF-subpathway associations between TFs and subpathways in the TSN with k=4. Table S7. The detailed information of the co-motif TFs regulated sub-pathways of TSN with k=4. Table S8. The detailed information of the co-family TFs regulated sub-pathways of TSN with k=4. Table S9. The detailed information of the co-tissue TFs regulated sub-pathways of TSN with k=4. Table S10. Real vs. random Jaccard coefficient of the TSN with k=4 at the every pathway level. [file 780357.f1.pdf]

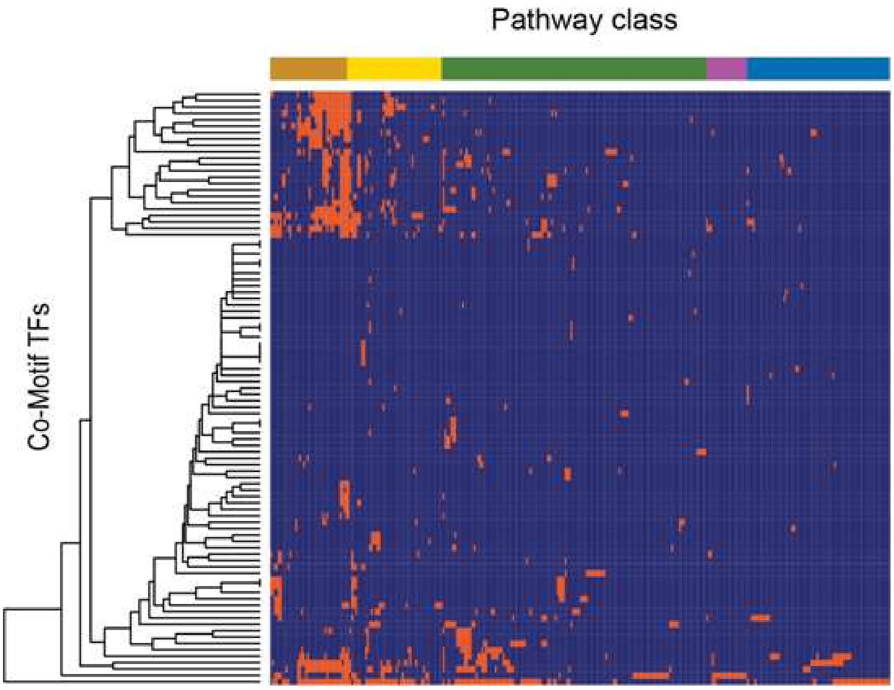

Figure S1. One-way clustering to TFs with common motif of the TSN with k=4. A. One-way clustering between 90 TFs and 320 subpathways. The corresponding cell was colored orange if there was an edge between the TF and subpathway in the TSN with k=4. Subpathway labels were colored according to the pathway class colors used in Figure 2.

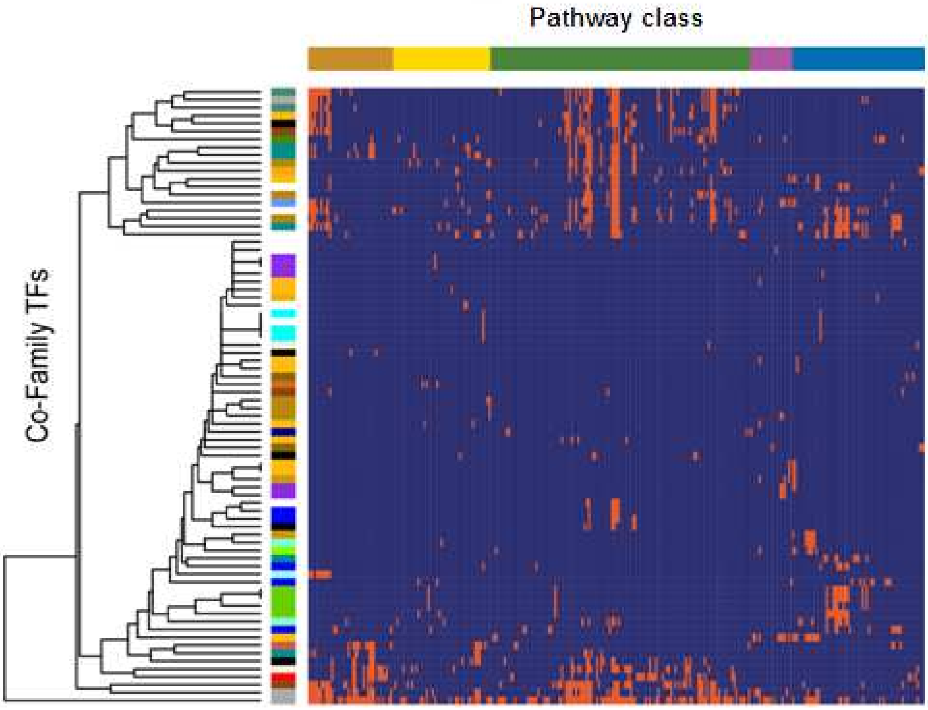

Figure S2. One-way clustering to TFs with common family of the TSN with k=4. A. One-way clustering between 78 TFs and 239 subpathways. The corresponding cell was colored orange if there was an edge between the TF and subpathway in the TSN with k=4. Subpathway labels were colored according to the pathway class colors used in Figure 2.

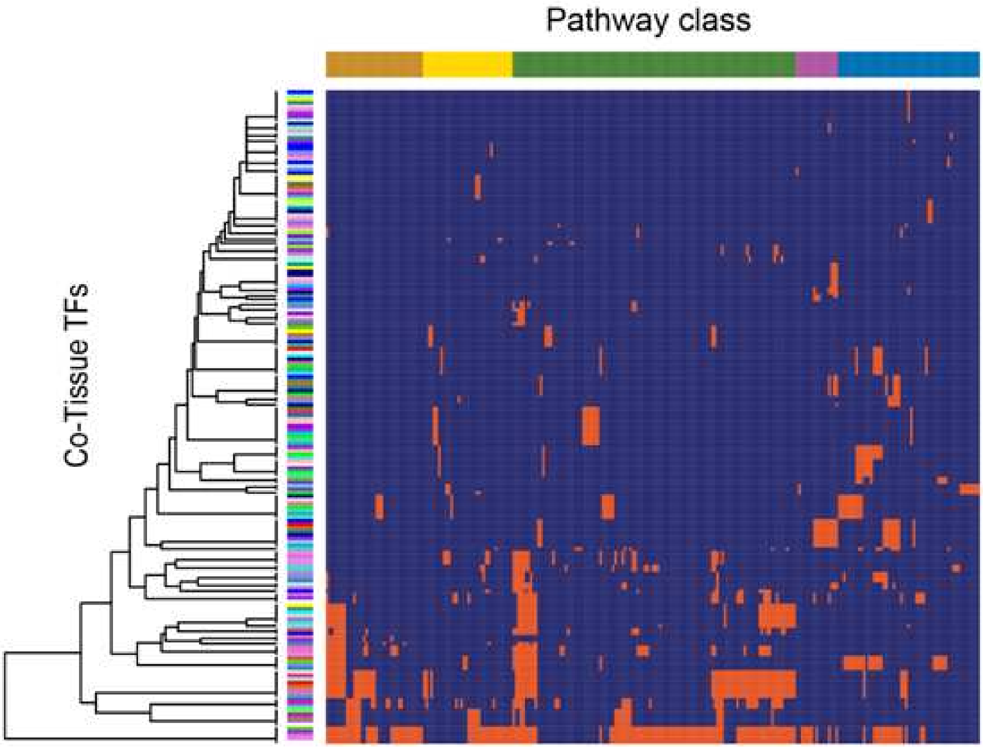

Figure S3. One-way clustering to TFs with common tissue of the TSN with k=4. A. One-way clustering between 60 unique TFs and 269 unique subpathways. The corresponding cell was colored orange if there was an edge between the TF and subpathway in the TSN with k=4. Subpathway labels were colored according to the pathway class colors used in Figure 2.

Table S1. The detailed information of the significant TF-subpathway associations between TFs and subpathways in the TSN.

TF_Name	Subpathway	P_Value
TP53	path:04115_1	0
TP53	path:04115_2	0
TP53	path:04115_3	0
TP53	path:04115_4	0
TP53	path:04115_7	0
SP1	path:04510_1	7.85E-13
SP1	path:04510_19	1.96E-12
TP53	path:04110_1	2.55E-11
SP1	path:04110_18	4.28E-11
SP1	path:04110_19	1.05E-10
SP1	path:04510_10	1.05E-10
SP1	path:04510_6	1.07E-10
SP1	path:04110_17	1.86E-10
SP1	path:04115_1	3.47E-10
SP1	path:04510_8	6.16E-10
TCF7L2	path:04310_7	1.19E-09
SP1	path:04110_20	2.92E-09
SP1	path:04110_27	2.92E-09
TP53	path:04110_7	4.42E-09
RELA	path:04620_12	4.80E-09
SP1	path:04110_23	5.22E-09
SP1	path:05200_51	5.54E-09
SP1	path:04110_3	6.46E-09
SP1	path:04110_4	6.46E-09
NR5A1	path:00140_13	6.63E-09
JUND	path:05200_29	6.84E-09
RELA	path:04620_18	8.85E-09
E2F1	path:04110_17	9.20E-09
E2F1	path:05200_52	9.20E-09
RELA	path:04620_16	1.03E-08
TCF7L2	path:04310_6	1.48E-08

SP1	path:05200_52	1.55E-08
MITF	path:04916_3	1.75E-08
SP1	path:05200_47	2.07E-08
SP1	path:05214_14	2.07E-08
SP1	path:05220_9	2.07E-08
FOS	path:05200_29	2.16E-08
RELA	path:04062_2	3.87E-08
HNF4A	path:00982_4	5.38E-08
HNF4A	path:00982_5	5.38E-08
HNF4A	path:00982_9	5.38E-08
RELA	path:04620_17	6.51E-08
RELA	path:04620_22	6.51E-08
SP1	path:04110_26	7.70E-08
RELA	path:04620_9	7.78E-08
SP1	path:04510_18	8.07E-08
E2F1	path:05212_13	1.10E-07
E2F1	path:05219_4	1.10E-07
E2F1	path:05223_3	1.10E-07
E2F1	path:04110_23	1.13E-07
E2F1	path:04110_26	1.13E-07
E2F1	path:04110_3	1.13E-07
E2F1	path:04110_4	1.13E-07
E2F1	path:05214_15	1.13E-07
E2F1	path:05218_7	1.13E-07
E2F1	path:05220_11	1.13E-07
SP1	path:05214_13	1.18E-07
SP1	path:05220_12	1.18E-07
SP3	path:04510_10	1.24E-07
SP3	path:04510_6	1.24E-07
SP3	path:04510_8	1.24E-07
RELA	path:04620_13	1.51E-07
RFX3	path:04514_1	1.53E-07
RFX3	path:04514_3	1.53E-07
RFX3	path:04612_3	1.53E-07

RFX3	path:04672_4	1.53E-07
RFX3	path:04672_5	1.53E-07
RFX3	path:04940_1	1.53E-07
RFX3	path:05150_14	1.53E-07
RFX3	path:05310_1	1.53E-07
RFX3	path:05310_3	1.53E-07
RFX3	path:05320_1	1.53E-07
RFX3	path:05320_3	1.53E-07
RFX3	path:05320_4	1.53E-07
RFX3	path:05320_6	1.53E-07
RFX3	path:05322_5	1.53E-07
RFX3	path:05322_6	1.53E-07
RFX3	path:05330_1	1.53E-07
RFX3	path:05330_2	1.53E-07
RFX3	path:05330_4	1.53E-07
RFX3	path:05332_1	1.53E-07
RFX3	path:05416_4	1.53E-07
E2F1	path:04110_19	1.74E-07
E2F1	path:05200_53	1.85E-07
RELA	path:04062_1	2.04E-07
RELA	path:04620_14	2.07E-07
SP3	path:04110_19	2.26E-07
SP3	path:04512_5	2.26E-07
HNF4A	path:00982_10	2.52E-07
SP1	path:04512_5	2.72E-07
SP3	path:04110_17	3.14E-07
TP53	path:04110_17	3.28E-07
SP3	path:04510_1	3.35E-07
IRF3	path:04620_11	3.65E-07
IRF3	path:04620_2	3.65E-07
IRF7	path:04620_11	3.65E-07
IRF7	path:04620_2	3.65E-07
E2F1	path:05222_1	3.73E-07
RFX2	path:04514_1	3.82E-07

RFX2	path:04514_3	3.82E-07
RFX2	path:04612_3	3.82E-07
RFX2	path:04672_4	3.82E-07
RFX2	path:04672_5	3.82E-07
RFX2	path:04940_1	3.82E-07
RFX2	path:05150_14	3.82E-07
RFX2	path:05310_1	3.82E-07
RFX2	path:05310_3	3.82E-07
RFX2	path:05320_1	3.82E-07
RFX2	path:05320_3	3.82E-07
RFX2	path:05320_4	3.82E-07
RFX2	path:05320_6	3.82E-07
RFX2	path:05322_5	3.82E-07
RFX2	path:05322_6	3.82E-07
RFX2	path:05330_1	3.82E-07
RFX2	path:05330_2	3.82E-07
RFX2	path:05330_4	3.82E-07
RFX2	path:05332_1	3.82E-07
RFX2	path:05416_4	3.82E-07
RFX3	path:05330_7	4.00E-07
SP1	path:04110_7	4.97E-07
SP1	path:05222_1	5.36E-07
AKNA	path:04060_31	6.20E-07
AKNA	path:04514_10	6.20E-07
AKNA	path:04514_11	6.20E-07
AKNA	path:04514_12	6.20E-07
AKNA	path:04514_78	6.20E-07
AKNA	path:04514_9	6.20E-07
AKNA	path:04672_2	6.20E-07
AKNA	path:05144_9	6.20E-07
AKNA	path:05145_20	6.20E-07
AKNA	path:05310_2	6.20E-07
AKNA	path:05320_2	6.20E-07
AKNA	path:05320_5	6.20E-07

AKNA	path:05322_7	6.20E-07
AKNA	path:05330_12	6.20E-07
AKNA	path:05330_3	6.20E-07
AKNA	path:05416_3	6.20E-07
SP1	path:04512_6	6.24E-07
SP3	path:04510_19	6.37E-07
CEBPB	path:04620_12	6.69E-07
SP1	path:04810_29	7.92E-07
SP1	path:05146_2	7.92E-07
SP3	path:05200_51	8.65E-07
SP1	path:05146_9	9.10E-07
SP1	path:04610_3	9.21E-07
SP1	path:04610_6	9.21E-07
SP1	path:05200_29	9.21E-07
SP1	path:05214_15	9.21E-07
SP1	path:05218_7	9.21E-07
SP1	path:05220_11	9.21E-07
CEBPB	path:04620_18	9.27E-07
CEBPB	path:04620_16	9.58E-07
RFX2	path:05330_7	1.00E-06
NR5A1	path:00140_8	1.09E-06
NR5A1	path:00140_9	1.09E-06
SP3	path:05146_2	1.13E-06
NR5A1	path:00140_16	1.15E-06
NR5A1	path:00140_18	1.15E-06
SP1	path:05146_1	1.23E-06
NR5A1	path:00140_10	1.53E-06
SP1	path:04512_12	1.66E-06
NR5A1	path:00140_19	1.72E-06
NR5A1	path:00140_7	1.72E-06
SP3	path:05146_1	1.74E-06
SP1	path:04630_3	1.97E-06
NFE2L2	path:00480_2	2.02E-06
RELA	path:04620_1	2.11E-06

RFX1	path:04514_1	2.14E-06
RFX1	path:04514_3	2.14E-06
RFX1	path:04612_3	2.14E-06
RFX1	path:04672_4	2.14E-06
RFX1	path:04672_5	2.14E-06
RFX1	path:04940_1	2.14E-06
RFX1	path:05150_14	2.14E-06
RFX1	path:05310_1	2.14E-06
RFX1	path:05310_3	2.14E-06
RFX1	path:05320_1	2.14E-06
RFX1	path:05320_3	2.14E-06
RFX1	path:05320_4	2.14E-06
RFX1	path:05320_6	2.14E-06
RFX1	path:05322_5	2.14E-06
RFX1	path:05322_6	2.14E-06
RFX1	path:05330_1	2.14E-06
RFX1	path:05330_2	2.14E-06
RFX1	path:05330_4	2.14E-06
RFX1	path:05332_1	2.14E-06
RFX1	path:05416_4	2.14E-06
TFAP2A	path:05200_8	2.16E-06
NR5A1	path:00140_5	2.18E-06
NR5A1	path:00140_6	2.70E-06
E2F1	path:04110_20	2.73E-06
E2F1	path:04110_27	2.73E-06
SPI1	path:04145_6	3.11E-06
SP1	path:05200_53	4.11E-06
TCF7L2	path:05200_31	4.20E-06
TCF7L2	path:05210_12	4.20E-06
TCF7L2	path:05210_7	4.20E-06
TCF7L2	path:05216_4	4.20E-06
HNF4A	path:04610_2	4.35E-06
HNF4A	path:04610_3	4.35E-06
CEBPB	path:04620_17	4.38E-06

CEBPB	path:04620_22	4.38E-06
SP3	path:04110_20	4.71E-06
SP3	path:04110_27	4.71E-06
CEBPB	path:04620_9	4.77E-06
SP1	path:04512_11	5.55E-06
SP1	path:04610_4	5.55E-06
RFX1	path:05330_7	5.59E-06
SP1	path:04630_4	5.95E-06
SP1	path:04115_4	6.18E-06
NR4A1	path:00140_13	6.42E-06
JUN	path:04620_18	6.60E-06
TFAP2A	path:05200_15	6.62E-06
HNF4A	path:04610_5	6.69E-06
MYC	path:05200_29	7.20E-06
SP1	path:04144_2	7.61E-06
PAX6	path:04510_1	7.62E-06
PAX6	path:04510_19	7.62E-06
ZBTB7A	path:04110_25	7.64E-06
SP1	path:04512_13	7.86E-06
ELF1	path:04060_8	8.41E-06
ZBTB7A	path:04110_27	9.07E-06
SP1	path:04512_9	9.36E-06
TCF7L2	path:05213_1	9.42E-06
ELF1	path:04664_2	1.05E-05
E2F1	path:05214_13	1.07E-05
E2F1	path:05214_21	1.07E-05
E2F1	path:05220_12	1.07E-05
SP1	path:04540_13	1.22E-05
CEBPB	path:04060_56	1.23E-05
SP3	path:05200_52	1.23E-05
IRF3	path:04620_12	1.24E-05
IRF7	path:04620_12	1.24E-05
LEF1	path:05200_31	1.30E-05
LEF1	path:05210_12	1.30E-05

LEF1	path:05210_7	1.30E-05
LEF1	path:05216_4	1.30E-05
SP1	path:04310_6	1.33E-05
SP1	path:04912_8	1.33E-05
SP1	path:05200_48	1.33E-05
SP1	path:05214_12	1.33E-05
SP1	path:05218_6	1.33E-05
SP1	path:05220_8	1.33E-05
PAX6	path:05414_2	1.40E-05
TFAP2A	path:04510_18	1.42E-05
SP1	path:04110_22	1.43E-05
SP1	path:04510_20	1.43E-05
SP1	path:00140_10	1.44E-05
SP1	path:00140_3	1.44E-05
SP1	path:00140_4	1.44E-05
SP1	path:05212_13	1.44E-05
SP1	path:05219_4	1.44E-05
SP1	path:05223_3	1.44E-05
ATF2	path:04620_18	1.46E-05
TP53	path:04110_19	1.48E-05
E2F1	path:05200_51	1.56E-05
E2F1	path:05215_7	1.56E-05
SP1	path:04512_3	1.66E-05
NR5A1	path:00140_11	1.69E-05
E2F1	path:04110_7	1.74E-05
SP1	path:04310_7	1.77E-05
SP1	path:05414_2	1.77E-05
SP3	path:05146_9	1.78E-05
TFAP2A	path:05200_11	1.91E-05
TFAP2A	path:05200_12	1.91E-05
TFAP2A	path:05200_4	1.91E-05
TP53	path:05200_48	1.95E-05
TP53	path:05200_8	1.95E-05
TP53	path:05214_12	1.95E-05

TP53	path:05218_6	1.95E-05
TP53	path:05220_8	1.95E-05
SP1	path:04512_10	2.01E-05
SP1	path:05200_3	2.01E-05
SP1	path:05222_4	2.01E-05
JUN	path:04620_17	2.03E-05
JUN	path:04620_22	2.03E-05
JUN	path:04620_9	2.03E-05
JUN	path:05200_29	2.03E-05
TCF7L2	path:05221_1	2.10E-05
TFAP2A	path:05200_10	2.20E-05
TFAP2A	path:05200_5	2.22E-05
TP63	path:04110_1	2.23E-05
TCF7L2	path:05200_42	2.26E-05
HNF4A	path:00830_2	2.36E-05
FOXA3	path:00982_11	2.48E-05
FOXA3	path:00982_3	2.48E-05
FOXA3	path:00982_7	2.48E-05
FOXA3	path:00982_8	2.48E-05
LEF1	path:05213_1	2.72E-05
PAX6	path:04510_20	2.77E-05
SP1	path:00140_13	2.77E-05
SP1	path:00140_19	2.77E-05
SP1	path:00140_7	2.77E-05
IRF1	path:04620_12	2.78E-05
ATF2	path:04620_17	3.02E-05
ATF2	path:04620_22	3.02E-05
ATF2	path:04620_9	3.02E-05
SP3	path:04110_23	3.02E-05
SP3	path:05214_13	3.02E-05
SP3	path:05220_12	3.02E-05
SP3	path:04110_3	3.13E-05
SP3	path:04110_4	3.13E-05
SREBF1	path:04910_5	3.27E-05

SP3	path:04512_12	3.50E-05
SP3	path:04512_3	3.50E-05
POU2F1	path:00830_2	3.54E-05
TFAP2A	path:04510_17	3.81E-05
HNF1A	path:00140_12	3.95E-05
SP3	path:00140_10	4.16E-05
SP3	path:00140_3	4.16E-05
SP3	path:00140_4	4.16E-05
SP3	path:05219_4	4.16E-05
PAX6	path:04810_29	4.23E-05
SP1	path:04115_3	4.23E-05
KLF4	path:04110_17	4.70E-05
KLF4	path:04110_19	4.70E-05
SP1	path:04650_20	4.84E-05
PAX6	path:04512_23	4.96E-05
PAX6	path:04670_9	4.96E-05
PAX6	path:05131_5	4.96E-05
PAX6	path:05131_6	4.96E-05
SP1	path:04115_7	4.96E-05
SP1	path:00140_5	5.07E-05
SP1	path:05200_50	5.07E-05
FOXA3	path:00982_4	5.10E-05
FOXA3	path:00982_5	5.10E-05
FOXA3	path:00982_9	5.10E-05
SMAD2	path:04110_25	5.26E-05
SMAD2	path:04510_1	5.26E-05
SMAD2	path:04510_19	5.26E-05
SMAD2	path:04512_13	5.26E-05
PAX6	path:04510_4	5.33E-05
STAT3	path:04630_4	5.36E-05
IRF3	path:04060_38	5.44E-05
IRF7	path:04060_38	5.44E-05
SP3	path:04512_13	5.52E-05
FOXA3	path:00982_2	5.58E-05

SP3	path:00140_11	5.63E-05
SP3	path:00140_13	5.63E-05
SP3	path:00140_19	5.63E-05
SP3	path:00140_7	5.63E-05
SP3	path:04512_6	5.63E-05
SP3	path:05214_15	5.63E-05
SP3	path:05218_7	5.63E-05
SP3	path:05220_11	5.63E-05
IRF3	path:04650_6	5.75E-05
IRF3	path:05160_6	5.75E-05
IRF7	path:04650_6	5.75E-05
IRF7	path:05160_6	5.75E-05
LEF1	path:05221_1	5.77E-05
NR5A1	path:00140_22	5.77E-05
NR5A1	path:00140_23	5.77E-05
NR5A1	path:00140_24	5.77E-05
LEF1	path:05200_42	6.00E-05
JUN	path:04620_12	6.18E-05
SP3	path:04510_18	6.32E-05
PAX6	path:04512_24	6.38E-05
PAX6	path:04514_57	6.38E-05
PAX6	path:05100_3	6.38E-05
PAX6	path:05140_1	6.38E-05
SMAD2	path:04110_18	6.60E-05
TP73	path:04115_2	6.67E-05
TP73	path:04115_3	6.67E-05
TP73	path:04115_4	6.67E-05
TP73	path:04115_7	6.67E-05
SP1	path:04115_2	6.74E-05
SP1	path:04510_4	6.74E-05
TP73	path:04115_1	6.75E-05
SP1	path:05218_8	7.27E-05
FOXA3	path:00982_10	7.28E-05
SP3	path:04512_9	7.41E-05

TP53	path:04110_22	7.56E-05
TP53	path:05200_47	7.56E-05
TP53	path:05214_14	7.56E-05
TP53	path:05220_9	7.56E-05
TP63	path:04110_7	8.12E-05
TP63	path:04115_1	8.12E-05
TP63	path:04115_2	8.12E-05
TP63	path:04115_3	8.12E-05
TP63	path:04115_4	8.12E-05
TP63	path:04115_7	8.12E-05
TP63	path:05200_48	8.12E-05
TP63	path:05214_12	8.12E-05
TP63	path:05218_6	8.12E-05
TP63	path:05220_8	8.12E-05
TFAP2A	path:04510_7	8.35E-05
TFAP2A	path:05200_29	8.35E-05
SP3	path:00140_5	8.41E-05
MYC	path:05200_50	8.52E-05
MYC	path:05222_2	8.52E-05
MYC	path:05222_7	8.52E-05
SP1	path:00140_6	8.66E-05
MYC	path:05222_1	8.86E-05
ENO1	path:00140_10	8.93E-05
ENO1	path:00140_13	8.93E-05
ENO1	path:00140_14	8.93E-05
ENO1	path:00140_16	8.93E-05
ENO1	path:00140_18	8.93E-05
ENO1	path:00140_19	8.93E-05
ENO1	path:00140_20	8.93E-05
ENO1	path:00140_3	8.93E-05
ENO1	path:00140_4	8.93E-05
ENO1	path:00140_7	8.93E-05
ENO1	path:00140_8	8.93E-05
ENO1	path:00140_9	8.93E-05

NR5A1	path:04060_21	8.93E-05
PAX6	path:04514_56	9.92E-05
HNF4A	path:00120_9	0.000100826
HNF4A	path:03320_2	0.000100826
HNF4A	path:04610_4	0.000100826
SP1	path:04512_14	0.000101569
ENO1	path:00140_5	0.000103024
STAT3	path:05200_29	0.000108961
SPI1	path:05140_4	0.000110411
NR5A1	path:00140_12	0.000116509
ENO1	path:00140_6	0.000116921
TP53	path:05200_20	0.00011707
TP53	path:05214_13	0.00011707
TP53	path:05220_12	0.00011707
LEF1	path:04310_7	0.000118706
NR1I2	path:00982_4	0.00011908
NR1I2	path:00982_5	0.00011908
NR1I2	path:00982_9	0.00011908
SMAD2	path:04512_12	0.000121975
NFYA	path:04115_2	0.000123767
SP1	path:00140_8	0.000123921
SP1	path:00140_9	0.000123921
SP1	path:04010_5	0.000123921
SP3	path:00140_12	0.000126279
SP3	path:00140_6	0.000126279
SP3	path:04110_18	0.000128011
PAX6	path:04514_49	0.000131273
PAX6	path:04514_50	0.000131273
NR2F1	path:03320_2	0.000132961
SP1	path:04610_7	0.000133832
SP1	path:05218_5	0.000133832
SP1	path:05222_2	0.000133832
RELA	path:04060_62	0.000137451
SP1	path:04510_17	0.000141426

SP3	path:05200_48	0.00015602
SP3	path:05214_12	0.00015602
SP3	path:05218_6	0.00015602
SP3	path:05220_8	0.00015602
JUN	path:04620_16	0.000158904
TP63	path:05200_47	0.000159403
TP63	path:05214_14	0.000159403
TP63	path:05220_9	0.000159403
SMAD2	path:04512_6	0.00016587
SP1	path:04512_27	0.00016592
TP53	path:05200_51	0.000172314
PAX6	path:05100_10	0.000173551
XBP1	path:04514_1	0.000178543
XBP1	path:04514_3	0.000178543
XBP1	path:04612_3	0.000178543
XBP1	path:04672_4	0.000178543
XBP1	path:04672_5	0.000178543
XBP1	path:04940_1	0.000178543
XBP1	path:05150_14	0.000178543
XBP1	path:05310_1	0.000178543
XBP1	path:05310_3	0.000178543
XBP1	path:05320_1	0.000178543
XBP1	path:05320_3	0.000178543
XBP1	path:05320_4	0.000178543
XBP1	path:05320_6	0.000178543
XBP1	path:05322_5	0.000178543
XBP1	path:05322_6	0.000178543
XBP1	path:05330_1	0.000178543
XBP1	path:05330_2	0.000178543
XBP1	path:05330_4	0.000178543
XBP1	path:05332_1	0.000178543
XBP1	path:05416_4	0.000178543
HNF4A	path:04610_6	0.000179282
CEBPA	path:04610_4	0.00018207

CEBPA	path:04610_6	0.00018207
NR1I3	path:00830_2	0.000186607
FOXA3	path:00140_11	0.000188543
CEBPB	path:04062_2	0.000193217
E2F1	path:04110_1	0.000197534
E2F1	path:05218_8	0.000197534
RUNX3	path:04612_4	0.000198099
REL	path:04650_22	0.00019873
REL	path:04650_23	0.00019873
TP53	path:04110_23	0.000198814
SP1	path:04610_8	0.000203885
SP1	path:04620_18	0.000203885
TP53	path:04110_3	0.000210553
TP53	path:05214_10	0.000210553
TP53	path:04722_19	0.000218217
PAX6	path:04510_10	0.000221093
PAX6	path:04510_6	0.000221093
PAX6	path:04510_8	0.000221093
SP1	path:04630_1	0.000231547
SP1	path:00140_16	0.000245716
SP1	path:00140_18	0.000245716
SP1	path:04512_18	0.000245716
SP1	path:05140_6	0.000245716
SP1	path:05214_21	0.000245716
FOXA3	path:00982_6	0.000248984
SPI1	path:04670_2	0.000252274
STAT4	path:04630_4	0.000257265
TP53	path:04210_23	0.000275763
TP53	path:04210_24	0.000275763
TP53	path:05200_11	0.000275763
TP53	path:05200_12	0.000275763
TP53	path:05200_4	0.000275763
TP53	path:05215_11	0.000275763
HNF4A	path:04950_4	0.000287791

SP1	path:05200_4	0.000289957
MYC	path:04110_20	0.000292768
MYC	path:04110_27	0.000292768
TXK	path:04612_7	0.000297672
NFYA	path:04110_23	0.000299258
NFYA	path:04110_3	0.000299258
SP3	path:04110_26	0.000307175
E2F1	path:04110_22	0.000308684
JUN	path:04650_20	0.000308777
SP1	path:05219_3	0.000310459
FOXA3	path:00591_1	0.000312584
TFAP2A	path:05214_10	0.000317116
TFAP2A	path:05215_10	0.000317116
XBP1	path:05330_7	0.000327268
FOXA3	path:00140_1	0.000327469
FOXA3	path:00140_25	0.000327469
FOXA3	path:00140_26	0.000327469
FOXA3	path:00980_2	0.000327469
TP53	path:05200_10	0.000338058
NFYA	path:04110_17	0.000340548
NFYA	path:04110_7	0.000340548
ESR1	path:04115_1	0.000345486
ESR1	path:05200_29	0.000345486
KLF5	path:05200_31	0.000357162
KLF5	path:05210_12	0.000357162
KLF5	path:05210_7	0.000357162
TP53	path:05200_5	0.000367024
STAT5A	path:04630_4	0.000370487
STAT5B	path:04630_4	0.000370487
ENO1	path:00140_11	0.000376878
MYC	path:04110_18	0.00038916
NR5A1	path:00140_14	0.000393628
ZBTB7A	path:05200_51	0.000396677
ZBTB7A	path:05214_13	0.000396677

ZBTB7A	path:05214_15	0.000396677
ZBTB7A	path:05218_7	0.000396677
ZBTB7A	path:05219_4	0.000396677
ZBTB7A	path:05220_11	0.000396677
ZBTB7A	path:05220_12	0.000396677
ATF2	path:04620_12	0.000407893
YY1	path:05214_13	0.000410896
YY1	path:05220_12	0.000410896
SP1	path:05218_4	0.000411461
SP1	path:04520_7	0.000412181
GLI3	path:04340_2	0.000416702
GLI3	path:05200_34	0.000416702
TP53	path:04210_20	0.000421718
SP3	path:04512_27	0.000423815
ZBTB7B	path:04512_11	0.000424221
ZBTB7B	path:04512_13	0.000424221
ZBTB7B	path:04512_14	0.000424221
ZBTB7B	path:05146_9	0.000424221
TFAP2A	path:05215_11	0.000436788
YY1	path:05200_51	0.00043761
SP1	path:00140_20	0.000441307
SP1	path:04010_26	0.000441307
SP1	path:05142_20	0.000441307
SP1	path:05210_10	0.000441307
SP1	path:05216_4	0.000441307
NR5A2	path:00140_11	0.000442761
SP1	path:04510_7	0.000448433
TFAP2A	path:05200_21	0.000448647
MITF	path:00350_1	0.00045151
YY1	path:04110_19	0.000476218
ZBTB7B	path:04512_10	0.000476318
ZBTB7B	path:04512_12	0.000476318
ZBTB7B	path:04512_27	0.000476318
ZBTB7B	path:04512_3	0.000476318

ZBTB7B	path:04512_5	0.000476318
ZBTB7B	path:05146_1	0.000476318
ZBTB7B	path:05146_2	0.000476318
YY1	path:04110_17	0.000485911
SP3	path:04512_10	0.000488202
FOXA3	path:00140_12	0.000502586
HNF4A	path:00591_1	0.000515682
MYC	path:04110_23	0.000518594
MYC	path:04110_26	0.000518594
MYC	path:04110_3	0.000518594
MYC	path:04110_4	0.000518594
ZBTB7B	path:04512_6	0.000520973
ELF1	path:04664_10	0.000533253
ELF1	path:04664_3	0.000533253
ELF1	path:04664_5	0.000533253
ELF1	path:04664_9	0.000533253
NR1I3	path:00982_4	0.000535628
NR1I3	path:00982_5	0.000535628
NR1I3	path:00982_9	0.000535628
ZBTB7B	path:04512_9	0.000536621
SP3	path:04512_19	0.000541628
SP1	path:04512_19	0.000549822
RELA	path:04115_1	0.0005539
TFAP2A	path:04144_2	0.00057548
TFAP2A	path:04520_5	0.00057548
TP53	path:04510_17	0.000580605
FOXA2	path:04610_3	0.000581017
FOXA2	path:04610_4	0.000581017
FOXA2	path:04610_6	0.000581017
TP73	path:04722_19	0.000595015
GATA4	path:00590_1	0.00061049
GATA4	path:00590_2	0.00061049
GATA4	path:00590_3	0.00061049
GATA4	path:00590_4	0.00061049

GATA4	path:00591_1	0.00061049
GATA4	path:00980_2	0.00061049
PAX6	path:04670_20	0.000614792
SP3	path:00120_2	0.000618445
SP3	path:00120_3	0.000618445
SP3	path:00140_8	0.000618445
SP3	path:00140_9	0.000618445
SP3	path:05200_47	0.000618445
SP3	path:05214_14	0.000618445
SP3	path:05220_9	0.000618445
SMAD4	path:04110_25	0.000620016
IRF1	path:04620_1	0.000622656
POU2F1	path:04110_18	0.000623378
YBX1	path:04510_16	0.00062354
YBX1	path:05218_2	0.00062354
NR5A1	path:00140_20	0.00062425
NR5A1	path:00140_3	0.00062425
NR5A1	path:00140_4	0.00062425
THRB	path:04080_2	0.000625072
CREB1	path:04620_18	0.000627719
ZBTB7A	path:05200_52	0.000644363
ZBTB7A	path:05222_1	0.000644363
ZBTB7A	path:05222_2	0.000644363
POU2F1	path:00982_4	0.000646745
POU2F1	path:00982_5	0.000646745
POU2F1	path:00982_9	0.000646745
POU2F1	path:05200_51	0.000646745
TXK	path:04650_22	0.000654737
TXK	path:04650_23	0.000654737
STAT6	path:04630_2	0.000657478
STAT6	path:04630_3	0.000657478
STAT6	path:04630_4	0.000657478
AR	path:04115_1	0.000666989
AR	path:04115_3	0.000666989

POU2F1	path:04110_17	0.000667343
POU2F1	path:04110_19	0.000667343
POU2F1	path:05200_50	0.000667343
LEF1	path:04310_6	0.000671935
PAX6	path:04670_14	0.00067845
ELF1	path:04650_15	0.000687264
ELF1	path:04664_1	0.000687264
ELF1	path:05310_4	0.000687264
ELF1	path:05310_5	0.000687264
KLF4	path:04110_20	0.000691257
KLF4	path:04110_27	0.000691257
EGR1	path:04510_10	0.00069989
EGR1	path:04510_18	0.00069989
MYC	path:04110_17	0.000714486
MYC	path:04110_7	0.000714486
SP1	path:04510_23	0.000718519
SP1	path:04722_19	0.000718519
SP1	path:05142_21	0.000718519
SP1	path:05200_31	0.000718519
SP1	path:05200_42	0.000718519
SP1	path:05210_12	0.000718519
SP1	path:05210_7	0.000718519
HNF4A	path:00980_2	0.000733563
SMAD4	path:05200_29	0.000734371
SMAD4	path:04110_18	0.000743893
ATF2	path:04620_16	0.000747082
SP3	path:04512_11	0.000758402
SP3	path:05218_8	0.000758402
STAT3	path:05142_4	0.000767514
NFYA	path:04115_3	0.000776011
NFYA	path:04115_4	0.000776011
NFYA	path:04115_7	0.000776011
NR4A1	path:00140_10	0.00077713
NR4A1	path:00140_14	0.00077713

NR4A1	path:00140_16	0.00077713
NR4A1	path:00140_18	0.00077713
NR4A1	path:00140_20	0.00077713
NR4A1	path:00140_3	0.00077713
NR4A1	path:00140_4	0.00077713
NR4A1	path:00140_8	0.00077713
NR4A1	path:00140_9	0.00077713
ZBTB7A	path:04350_6	0.000800541
YBX1	path:04510_15	0.000802879
YBX1	path:04510_25	0.000802879
YBX1	path:04510_5	0.000802879
SP3	path:04144_2	0.000814667
SP3	path:04810_29	0.000814667
ENO1	path:00140_22	0.000819573
ENO1	path:00140_23	0.000819573
ENO1	path:00140_24	0.000819573
CREM	path:05200_29	0.000820491
ETV4	path:05200_29	0.000820491
NR4A1	path:00140_19	0.000832486
NR4A1	path:00140_7	0.000832486
CEBPB	path:04062_1	0.000853445
POU2F1	path:00590_1	0.000870331
POU2F1	path:00590_2	0.000870331
POU2F1	path:00590_3	0.000870331
POU2F1	path:00590_4	0.000870331
POU2F1	path:05200_52	0.000870331
POU2F1	path:05222_1	0.000870331
POU2F1	path:05222_2	0.000870331
JUN	path:04310_7	0.000877028
SPI1	path:05200_43	0.000902646
NRL	path:04744_2	0.00090302
SP3	path:04512_14	0.000906884
SMAD3	path:04110_25	0.000921676
SMAD3	path:04512_13	0.000921676

SMAD3	path:05200_29	0.000921676
SP3	path:00120_1	0.000926143
SP3	path:00140_16	0.000926143
SP3	path:00140_18	0.000926143
SP3	path:04512_18	0.000926143
NR4A1	path:00140_5	0.000960385
SPI1	path:04670_15	0.000961365
TP53	path:05200_15	0.000968321
FOXA3	path:00830_2	0.000975791
ZBTB7A	path:04110_1	0.001010691
ZBTB7A	path:05218_8	0.001010691
SP1	path:04060_32	0.001014956
SP1	path:05100_3	0.001014956
SP1	path:05145_21	0.001014956
SP1	path:05200_56	0.001014956
KLF4	path:04110_23	0.001048885
KLF4	path:04110_26	0.001048885
KLF4	path:04110_3	0.001048885
KLF4	path:04110_4	0.001048885
SMAD3	path:04110_18	0.001049896
SMAD3	path:04510_1	0.00105392
NR4A1	path:00140_6	0.001089761
SP1	path:04210_24	0.001107767
SP1	path:05142_10	0.001107767
SP1	path:05142_4	0.001107767
TP53	path:04510_7	0.001110697
NFYA	path:04115_1	0.001111281
TFAP2A	path:05215_8	0.001124594
HNF4A	path:03320_1	0.001131883
TXK	path:04350_11	0.001138141
RBPJ	path:04620_10	0.001146301
RBPJ	path:04620_15	0.001146301
RBPJ	path:04620_25	0.001146301
RBPJ	path:04622_3	0.001146301

RBPJ	path:04623_5	0.001146301
RBPJ	path:05120_3	0.001146301
RBPJ	path:05140_13	0.001146301
RBPJ	path:05140_9	0.001146301
RBPJ	path:05142_21	0.001146301
RBPJ	path:05145_5	0.001146301
RBPJ	path:05160_17	0.001146301
RBPJ	path:05215_12	0.001146301
RBPJ	path:05215_2	0.001146301
RBPJ	path:05220_6	0.001146301
SP1	path:04510_15	0.001164758
SP1	path:05215_10	0.001164758
YBX1	path:04510_17	0.001170159
SMAD3	path:04510_19	0.001170463
ZBTB7A	path:04110_20	0.001223683
ZBTB7A	path:04110_22	0.001223683
RBPJ	path:04062_19	0.001225306
RBPJ	path:04210_3	0.001225306
RBPJ	path:04722_24	0.001225306
RBPJ	path:05120_6	0.001225306
RBPJ	path:05131_9	0.001225306
RBPJ	path:05160_3	0.001225306
ELF1	path:04650_14	0.001225713
GATA4	path:00140_8	0.001226506
GATA4	path:00140_9	0.001226506
GATA4	path:00140_10	0.001238279
GATA4	path:00140_13	0.001238279
GATA4	path:00140_16	0.001238279
GATA4	path:00140_18	0.001238279
GATA4	path:00140_19	0.001238279
GATA4	path:00140_3	0.001238279
GATA4	path:00140_4	0.001238279
GATA4	path:00140_7	0.001238279
GATA4	path:00982_4	0.001238279

GATA4	path:00982_5	0.001238279
GATA4	path:00982_9	0.001238279
TFAP2A	path:04520_3	0.001262516
TFAP2A	path:04520_7	0.001262516
TFAP2A	path:04510_10	0.001269093
TFAP2A	path:04510_8	0.001271633
SP3	path:04110_22	0.001275861
TFAP2A	path:04510_6	0.001276164
CREB1	path:04620_17	0.001277819
CREB1	path:04620_22	0.001277819
CREB1	path:04620_9	0.001277819
EGR1	path:04512_3	0.00129686
STAT1	path:05210_10	0.001312831
SMAD3	path:04512_12	0.001326293
ENO1	path:00140_12	0.001347395
SP3	path:00140_20	0.001366718
SP3	path:04510_17	0.001366718
SP3	path:05212_13	0.001366718
SP3	path:05223_3	0.001366718
TP53	path:05218_8	0.001385048
JUN	path:04115_1	0.001389686
SP1	path:04630_2	0.001390056
SP1	path:00980_2	0.001393247
RBPJ	path:04621_4	0.001400007
SREBF1	path:04910_6	0.00143191
SP3	path:05414_2	0.00143924
NFYA	path:04110_1	0.00144529
GATA4	path:00140_5	0.001470007
YBX1	path:04510_7	0.001493429
YBX1	path:05218_4	0.001493429
KLF4	path:04110_7	0.001498093
POU2F1	path:04110_25	0.001500996
STAT1	path:04630_4	0.001501202
RBPJ	path:04620_18	0.00151031

RBPJ	path:04660_1	0.00151031
ZBTB7A	path:04110_18	0.001518502
SPI1	path:04060_41	0.00153244
SPI1	path:04670_14	0.00153244
SP1	path:00140_11	0.001534931
RBPJ	path:04622_5	0.001558335
RBPJ	path:04662_1	0.001558335
RBPJ	path:05160_8	0.001558335
SP1	path:04510_13	0.001603207
SP1	path:04520_5	0.001603207
SP1	path:05222_7	0.001603207
VDR	path:00982_11	0.001635842
VDR	path:00982_3	0.001635842
VDR	path:00982_7	0.001635842
VDR	path:00982_8	0.001635842
MYC	path:05200_47	0.00165995
MYC	path:05214_14	0.00165995
MYC	path:05220_9	0.00165995
SP1	path:00240_2	0.001690362
TP53	path:05219_3	0.001691972
HNF4A	path:00120_6	0.001698537
HNF4A	path:04610_8	0.001698537
GATA4	path:00140_6	0.001710596
HNF4A	path:00590_2	0.001722691
HNF4A	path:00590_3	0.001722691
TP53	path:04510_18	0.001774645
TP53	path:05214_11	0.001774645
TP53	path:05218_5	0.001774645
RELA	path:04110_19	0.001781655
RELA	path:04650_23	0.001781655
HNF4A	path:00590_1	0.001808381
TCF7L2	path:05210_10	0.001830462
SMAD3	path:04512_6	0.001837367
GATA6	path:00590_1	0.00185453

GATA6	path:00590_2	0.00185453
GATA6	path:00590_3	0.00185453
GATA6	path:00590_4	0.00185453
GATA6	path:00591_1	0.00185453
RELA	path:04110_17	0.001869278
RELA	path:04110_7	0.001869278
SP1	path:00140_1	0.001893526
SP1	path:00140_25	0.001893526
SP1	path:04620_9	0.001893526
HNF4A	path:00590_4	0.001898001
RBPJ	path:05131_12	0.001907232
RBPJ	path:05222_10	0.001907232
IRF1	path:04620_13	0.00193948
IRF1	path:04620_14	0.00193948
TP53	path:04110_20	0.001943417
TP53	path:04110_27	0.001943417
ZBTB7A	path:04110_23	0.001957069
ZBTB7A	path:04110_26	0.001957069
ZBTB7A	path:04110_3	0.001957069
ZBTB7A	path:04110_4	0.001957069
GATA4	path:00982_10	0.001958811
RELA	path:04650_22	0.00197363
MYC	path:05214_13	0.001974254
MYC	path:05214_21	0.001974254
MYC	path:05220_12	0.001974254
IRF1	path:04620_18	0.002013452
MYC	path:05200_51	0.002023761
MYC	path:05210_10	0.002023761
MYC	path:05212_13	0.002023761
MYC	path:05216_4	0.002023761
MYC	path:05219_4	0.002023761
MYC	path:05223_3	0.002023761
MYC	path:05200_31	0.002048915
MYC	path:05210_12	0.002048915

MYC	path:05210_7	0.002048915
MYC	path:05214_15	0.002048915
MYC	path:05218_3	0.002048915
MYC	path:05218_7	0.002048915
MYC	path:05220_11	0.002048915
YBX1	path:04510_18	0.002082955
RBPJ	path:04920_11	0.002086483
STAT1	path:04620_16	0.002113614
STAT1	path:04620_18	0.002113614
IRF1	path:04620_16	0.002131661
NFYA	path:04110_22	0.002132484
TCF7L2	path:05215_4	0.002138846
SP1	path:04060_22	0.002149604
SP1	path:04512_20	0.002149604
SP1	path:05146_5	0.002149604
SP1	path:05220_5	0.002149604
PAX6	path:04670_2	0.002170272
SP1	path:00982_10	0.002175912
SP1	path:04210_20	0.002175912
SP1	path:04510_24	0.002175912
RBPJ	path:05131_10	0.002195318
RBPJ	path:05222_9	0.002195318
NR5A2	path:00140_12	0.002212517
SP3	path:04115_4	0.002218693
NR5A1	path:04350_13	0.002225649
ZBTB7A	path:04110_19	0.002271569
ATF2	path:05200_29	0.002319558
RBPJ	path:04920_1	0.002376334
TFAP2A	path:04520_2	0.002407854
TFAP2A	path:05214_11	0.002407854
TFAP2A	path:05219_2	0.002407854
TCF7L2	path:05200_50	0.002450012
ZBTB7A	path:04110_17	0.002452708
TP53	path:04012_12	0.002466645

TP53	path:04510_16	0.002466645
MYC	path:05200_21	0.002484447
SP1	path:04510_25	0.0024986
SP1	path:04510_5	0.0024986
STAT5B	path:05200_47	0.002527186
STAT5B	path:05200_51	0.002527186
STAT5B	path:05214_13	0.002527186
STAT5B	path:05214_14	0.002527186
STAT5B	path:05214_15	0.002527186
STAT5B	path:05218_7	0.002527186
STAT5B	path:05219_4	0.002527186
STAT5B	path:05220_11	0.002527186
STAT5B	path:05220_12	0.002527186
STAT5B	path:05220_9	0.002527186
E2F1	path:05200_54	0.002554435
RBPJ	path:04722_13	0.002559734
RBPJ	path:04620_9	0.00259278
RBPJ	path:05142_8	0.00259278
RBPJ	path:05200_18	0.00259278
SP3	path:04060_43	0.002641876
SP3	path:05144_3	0.002641876
LEF1	path:04916_3	0.002645905
LEF1	path:05210_10	0.002645905
JUN	path:04210_24	0.002648312
AR	path:04110_18	0.002649237
TFAP2A	path:05212_11	0.002699451
JUNB	path:04350_10	0.002723194
JUNB	path:04350_12	0.002723194
SMAD2	path:04510_10	0.002748036
SMAD2	path:04510_6	0.002748036
SMAD2	path:04510_8	0.002748036
TP53	path:05218_2	0.002754132
TP53	path:04912_8	0.002756828
TCF7L2	path:05222_7	0.00276327

FOSL1	path:05200_29	0.002765029
SP1	path:04510_16	0.002771235
TP63	path:05200_19	0.002783081
TP63	path:05200_20	0.002783081
TP63	path:05215_3	0.002837304
ZBTB7B	path:04510_1	0.002839716
ZBTB7B	path:04510_10	0.002839716
ZBTB7B	path:04510_19	0.002839716
ZBTB7B	path:04510_6	0.002839716
ZBTB7B	path:04510_8	0.002839716
KLF4	path:05200_47	0.002858346
KLF4	path:05214_14	0.002858346
KLF4	path:05220_9	0.002858346
SP3	path:04510_7	0.002877506
NR5A2	path:00140_8	0.002898659
NR5A2	path:00140_9	0.002898659
GLI1	path:04340_2	0.002914772
GLI1	path:05200_34	0.002914772
MYC	path:05200_53	0.002948165
ESR1	path:04115_3	0.002948202
ESR1	path:04115_4	0.002948202
ESR1	path:04115_7	0.002948202
ESR1	path:04610_8	0.002948202
JUN	path:04610_7	0.002952732
JUN	path:04940_4	0.002952732
JUN	path:05120_6	0.002952732
JUN	path:05320_9	0.002952732
JUN	path:05330_5	0.002952732
JUN	path:05332_2	0.002952732
JUN	path:05332_5	0.002952732
GATA2	path:04060_47	0.002976989
ESR1	path:04115_2	0.002979325
TP53	path:05200_19	0.002997803
SRF	path:05410_2	0.003033557

SRF	path:05414_3	0.003033557
SRF	path:05416_1	0.003033557
SMAD4	path:04115_2	0.003046128
SMAD4	path:04115_3	0.003046128
SMAD4	path:04115_4	0.003046128
SMAD4	path:04115_7	0.003046128
ATF2	path:04110_18	0.003077219
TCF7L2	path:05222_2	0.003078131
NR5A2	path:00140_18	0.003089823
NR5A2	path:00140_3	0.003089823
NR5A2	path:00140_4	0.003089823
TP63	path:05200_17	0.003115907
LEF1	path:05215_4	0.003117148
NFE2L2	path:00480_1	0.003129559
NFE2L2	path:00480_3	0.003129559
NFE2L2	path:00480_4	0.003129559
NFE2L2	path:00480_5	0.003129559
NFE2L2	path:00480_6	0.003129559
YY1	path:05218_8	0.003139302
EGR1	path:04510_6	0.003153788
EGR1	path:04510_8	0.003153788
EGR1	path:04540_13	0.003153788
EGR1	path:05146_9	0.003153788
EGR1	path:05218_4	0.003153788
POU2F1	path:04110_20	0.0031585
POU2F1	path:04110_27	0.0031585
FOS	path:04610_7	0.003173805
FOS	path:04620_18	0.003173805
GLI3	path:05200_57	0.003234702
GLI3	path:05217_2	0.003234702
TP53	path:05215_3	0.00331496
TFAP2A	path:04510_16	0.003332624
TFAP2A	path:05212_12	0.003332624
VDR	path:00982_4	0.003361044

VDR	path:00982_5	0.003361044
VDR	path:00982_9	0.003361044
KLF4	path:04115_1	0.003364391
KLF4	path:05214_13	0.003364391
KLF4	path:05220_12	0.003364391
SMAD4	path:04115_1	0.003366083
SP1	path:00240_17	0.003391309
TCF7L2	path:05222_1	0.003394233
MYC	path:05213_1	0.003437632
POU2F1	path:04664_5	0.003467546
NR5A2	path:00140_7	0.003474881
SPI1	path:04514_62	0.003570772
SPI1	path:04670_10	0.003570772
AR	path:04110_23	0.003572289
KLF4	path:05200_31	0.003572583
KLF4	path:05200_51	0.003572583
KLF4	path:05210_12	0.003572583
KLF4	path:05210_7	0.003572583
KLF4	path:05214_15	0.003572583
KLF4	path:05218_7	0.003572583
KLF4	path:05219_4	0.003572583
KLF4	path:05220_11	0.003572583
TP53	path:05214_1	0.003580594
TP53	path:05214_4	0.003580594
LEF1	path:05200_50	0.003595279
SP3	path:05200_53	0.003634657
VDR	path:00982_2	0.003675018
SMAD2	path:05146_9	0.003683338
YY1	path:04110_20	0.003725909
YY1	path:04110_27	0.003725909
YY1	path:05219_3	0.003725909
SRF	path:05020_9	0.00379009
NFYA	path:04110_12	0.003794515
SPI1	path:04650_13	0.003805584

HNF4A	path:00120_19	0.003812707
HNF4A	path:00120_7	0.003812707
STAT1	path:04620_17	0.003819783
STAT1	path:04620_22	0.003819783
STAT1	path:04620_9	0.003819783
STAT1	path:05200_29	0.003819783
TFAP2A	path:04080_7	0.003857334
TFAP2A	path:04912_8	0.003857334
NR5A2	path:00140_5	0.00385968
SP1	path:00120_2	0.003885876
SP1	path:00120_3	0.003885876
SP1	path:00140_14	0.003885876
SP1	path:05142_17	0.003885876
SP1	path:05142_18	0.003885876
RBPJ	path:05215_3	0.00389941
TP53	path:05200_17	0.00392722
SP1	path:04010_10	0.003931316
SP1	path:05142_11	0.003931316
SP1	path:05200_19	0.003935354
SP1	path:05200_20	0.003935354
MYC	path:05200_52	0.003950679
GLI3	path:05217_1	0.003961103
NFATC1	path:04060_32	0.003964221
NFATC1	path:04060_33	0.003964221
NFATC1	path:04650_20	0.003964221
GATA3	path:04612_4	0.003994597
STAT6	path:00590_4	0.003994719
TFAP2C	path:04912_8	0.00404499
TFAP2C	path:05219_2	0.00404499
TFAP2A	path:05214_1	0.004058678
TFAP2A	path:05214_17	0.004058678
TFAP2A	path:05214_2	0.004058678
TFAP2A	path:05214_3	0.004058678
TFAP2A	path:05214_4	0.004058678

TFAP2A	path:05214_5	0.004058678
TFAP2A	path:05214_6	0.004058678
TFAP2A	path:05214_8	0.004058678
SP3	path:04115_1	0.00406173
LEF1	path:05222_7	0.004078733
ATF2	path:04110_23	0.004084659
ATF2	path:04110_26	0.004084659
ATF2	path:04110_3	0.004084659
ATF2	path:04110_4	0.004084659
TP63	path:05215_11	0.004120782
CREB1	path:04620_12	0.004131401
CREB1	path:04620_16	0.004131401
CREB1	path:04672_4	0.004131401
CREB1	path:04672_5	0.004131401
CREB1	path:04940_1	0.004131401
CREB1	path:05310_1	0.004131401
CREB1	path:05310_3	0.004131401
CREB1	path:05320_1	0.004131401
CREB1	path:05320_3	0.004131401
CREB1	path:05320_4	0.004131401
CREB1	path:05320_6	0.004131401
CREB1	path:05322_5	0.004131401
CREB1	path:05322_6	0.004131401
CREB1	path:05330_1	0.004131401
CREB1	path:05330_2	0.004131401
CREB1	path:05330_4	0.004131401
CREB1	path:05332_1	0.004131401
CREB1	path:05416_4	0.004131401
REL	path:04060_8	0.004162941
PAX6	path:04512_9	0.004167242
CEBPA	path:04060_54	0.0042174
NR5A2	path:00140_6	0.004244219
CREB1	path:04514_1	0.004288489
CREB1	path:04514_3	0.004288489

CREB1	path:04612_3	0.004288489
CREB1	path:05150_14	0.004288489
TP53	path:04110_8	0.004291125
NR1I2	path:00830_2	0.004391058
NFIC	path:00591_1	0.004396514
NFIC	path:00982_7	0.004396514
NFIC	path:00982_8	0.004396514
NFIC	path:04080_7	0.004396514
NFIC	path:05200_48	0.004396514
NFIC	path:05214_12	0.004396514
NFIC	path:05218_5	0.004396514
NFIC	path:05218_6	0.004396514
NFIC	path:05220_8	0.004396514
TP53	path:04110_4	0.004511216
TP53	path:04510_15	0.004511216
TP53	path:05215_10	0.004511216
SRF	path:05020_8	0.004545882
NFYA	path:04110_26	0.004551637
NFYA	path:04110_8	0.004551637
LEF1	path:05222_2	0.004566365
SP3	path:00982_10	0.004572948
SP3	path:04510_20	0.004572948
SP3	path:04610_7	0.004572948
GLI1	path:04510_11	0.004577555
ATF2	path:04110_19	0.004580141
ATF2	path:04612_7	0.004580141
ATF2	path:04650_20	0.004580141
ATF2	path:05020_9	0.004580141
E2F1	path:05014_4	0.004589831
IRF1	path:04620_17	0.004615096
IRF1	path:04620_22	0.004615096
IRF1	path:04620_9	0.004615096
IRF1	path:05160_6	0.004615096
ATF2	path:04110_17	0.004619377

HMGA1	path:04060_8	0.004640977
SMAD4	path:05210_10	0.004709635
NFYA	path:04110_4	0.004713566
VDR	path:00982_10	0.004788204
JUN	path:04310_6	0.004858763
PAX6	path:04510_17	0.004905129
E2F1	path:04115_1	0.00499983
IRF5	path:04060_38	0.005011517
IRF5	path:04620_11	0.005011517
IRF5	path:04620_2	0.005011517
IRF5	path:04650_6	0.005011517
IRF5	path:05160_6	0.005011517
STAT5B	path:05200_52	0.005012521
LEF1	path:05222_1	0.005057313
IRF1	path:04620_11	0.005102853
IRF1	path:04620_2	0.005102853
SP1	path:00330_9	0.005126386
SP1	path:04520_3	0.005126386
IRF3	path:04620_14	0.005131788
IRF7	path:04620_14	0.005131788
STAT1	path:04110_18	0.00514314
YY1	path:05200_48	0.005151692
YY1	path:05214_12	0.005151692
YY1	path:05218_6	0.005151692
YY1	path:05220_8	0.005151692
REL	path:04612_7	0.005200652
KLF4	path:05200_53	0.005224296
IRF3	path:04620_18	0.005305274
IRF7	path:04620_18	0.005305274
GATA6	path:00982_4	0.005342537
GATA6	path:00982_5	0.005342537
GATA6	path:00982_9	0.005342537
SPI1	path:04145_2	0.005348786
SPI1	path:05150_17	0.005348786

GLI3	path:04340_1	0.005356158
NFYA	path:04110_11	0.005383343
ESR1	path:00830_2	0.005413618
TFAP2A	path:04510_15	0.005458314
IRF3	path:04620_16	0.005500251
IRF7	path:04620_16	0.005500251
SP1	path:00240_16	0.005536058
SP1	path:00330_1	0.005536058
SP1	path:05214_2	0.005536058
SP1	path:05214_5	0.005536058
LEF1	path:04916_11	0.00555092
NFYA	path:04110_19	0.005565492
YBX1	path:04510_10	0.005634473
YBX1	path:04510_6	0.005634473
YBX1	path:04510_8	0.005634473
YBX1	path:04520_3	0.005634473
YBX1	path:04520_5	0.005634473
YBX1	path:04520_7	0.005634473
YBX1	path:05100_15	0.005634473
YBX1	path:05100_2	0.005634473
YBX1	path:05200_9	0.005634473
YBX1	path:05211_1	0.005634473
YBX1	path:05211_2	0.005634473
YBX1	path:05211_7	0.005634473
YBX1	path:05211_8	0.005634473
YBX1	path:05213_6	0.005634473
YBX1	path:05223_5	0.005634473
YBX1	path:05223_7	0.005634473
SMAD2	path:04512_11	0.005671238
SP3	path:00140_26	0.005691454
SP3	path:04610_8	0.005691454
SP3	path:05222_1	0.005691454
FOS	path:04115_1	0.005719136
TFAP2A	path:04020_2	0.005723968

ATF2	path:00140_8	0.005778895
ATF2	path:00140_9	0.005778895
AR	path:04115_2	0.005794552
AR	path:04115_4	0.005794552
AR	path:04115_7	0.005794552
AR	path:05200_16	0.005794552
AR	path:05200_47	0.005794552
AR	path:05214_13	0.005794552
AR	path:05214_14	0.005794552
AR	path:05220_12	0.005794552
AR	path:05220_9	0.005794552
SMAD2	path:04512_14	0.005849438
MYC	path:05221_1	0.005908255
YY1	path:04110_23	0.005922984
NFIC	path:00982_11	0.005988829
NFIC	path:00982_3	0.005988829
AR	path:05200_51	0.006067474
AR	path:05216_4	0.006067474
AR	path:05219_4	0.006067474
SP1	path:00120_1	0.006087664
SP1	path:00140_12	0.006087664
SP1	path:05014_3	0.006087664
SP1	path:05145_19	0.006087664
SP1	path:05145_8	0.006087664
STAT1	path:04145_5	0.006088364
STAT1	path:04630_3	0.006088364
YBX1	path:05212_1	0.00609256
SP1	path:00350_2	0.006192277
SP1	path:00350_3	0.006192277
SP1	path:04110_25	0.006192277
SP1	path:05221_1	0.006192277
SP3	path:00140_1	0.006217062
SP3	path:00140_25	0.006217062
AR	path:05214_15	0.006236059

AR	path:05215_1	0.006236059
AR	path:05218_7	0.006236059
AR	path:05220_11	0.006236059
FOS	path:04620_17	0.006237239
FOS	path:04620_22	0.006237239
FOS	path:04620_9	0.006237239
NR1I3	path:00980_1	0.006275482
FOS	path:04310_7	0.006308233
ELF1	path:04650_1	0.006320724
ELF1	path:05142_21	0.006320724
MYC	path:05200_42	0.006520738
ATF2	path:00140_18	0.006675857
ATF2	path:05214_13	0.006675857
ATF2	path:05214_21	0.006675857
ATF2	path:05220_12	0.006675857
TCF7	path:04660_20	0.00669167
PAX6	path:04510_7	0.006713786
SP3	path:05219_3	0.00677863
IRF1	path:04060_51	0.00677924
SMAD4	path:05210_9	0.006805486
E2F1	path:04110_16	0.006872543
POU2F1	path:00591_1	0.006943045
TP53	path:04510_25	0.006951959
TP53	path:04510_5	0.006951959
YBX1	path:04012_5	0.007005212
YBX1	path:04520_2	0.007005212
YBX1	path:05212_11	0.007005212
YBX1	path:05214_11	0.007005212
YBX1	path:05223_6	0.007005212
EGR1	path:05212_11	0.007008495
NFIC	path:05200_47	0.007050709
NFIC	path:05214_14	0.007050709
NFIC	path:05220_9	0.007050709
KLF4	path:05200_52	0.007103944

SMAD3	path:05210_10	0.007105406
KLF4	path:04512_4	0.007140402
KLF4	path:04512_7	0.007140402
KLF4	path:04512_8	0.007140402
KLF4	path:05145_14	0.007140402
MYC	path:05218_8	0.007148499
IRF1	path:04670_2	0.00717245
HNF4A	path:00120_15	0.00730316
ATF2	path:05200_51	0.007303382
ATF2	path:05210_10	0.007303382
ATF2	path:05212_13	0.007303382
ATF2	path:05219_4	0.007303382
ATF2	path:05223_3	0.007303382
SPI1	path:04650_12	0.007340609
STAT3	path:04060_3	0.007358355
YY1	path:04650_20	0.007430866
SP1	path:04060_47	0.007433507
SP1	path:04940_4	0.007433507
SP1	path:05016_8	0.007433507
SP1	path:05320_9	0.007433507
SP1	path:05330_5	0.007433507
SP1	path:05332_2	0.007433507
SP1	path:05332_5	0.007433507
TP53	path:04010_21	0.007443002
TP53	path:05160_12	0.007459365
TP53	path:05213_2	0.007459365
JUN	path:04512_23	0.007569048
JUN	path:05131_6	0.007569048
YY1	path:00140_14	0.00758906
YY1	path:00140_8	0.00758906
YY1	path:00140_9	0.00758906
YY1	path:04110_7	0.00758906
YY1	path:05200_47	0.00758906
YY1	path:05214_14	0.00758906

YY1	path:05220_9	0.00758906
SPI1	path:04514_55	0.007630608
JUN	path:04115_3	0.007635426
JUN	path:04115_4	0.007635426
SP3	path:04110_7	0.007662288
KLF4	path:04512_1	0.007702669
KLF4	path:04512_2	0.007702669
TP53	path:04010_15	0.007702858
TFAP2A	path:04510_25	0.007739985
TFAP2A	path:04510_5	0.007739985
TFAP2A	path:05214_16	0.007739985
TFAP2A	path:05214_7	0.007739985
LEF1	path:04916_8	0.007825657
NFIC	path:00982_4	0.00787147
NFIC	path:00982_5	0.00787147
NFIC	path:00982_9	0.00787147
NFIC	path:04010_30	0.00787147
NFIC	path:04010_31	0.00787147
NFIC	path:04115_2	0.00787147
NFIC	path:04115_3	0.00787147
NFIC	path:04115_4	0.00787147
NFIC	path:04115_7	0.00787147
NFIC	path:04660_8	0.00787147
NFIC	path:04662_6	0.00787147
NFIC	path:04722_19	0.00787147
NFIC	path:05160_15	0.00787147
NFIC	path:05200_51	0.00787147
NFIC	path:05214_13	0.00787147
NFIC	path:05220_12	0.00787147
POU2F1	path:04664_2	0.007941331
STAT3	path:04630_2	0.00794491
STAT3	path:04630_3	0.00794491
JUN	path:04115_7	0.007953336
NFIC	path:04115_1	0.008000492

E2F1	path:04110_15	0.008121608
YBX1	path:05212_12	0.008169375
ATF2	path:00140_7	0.008207011
ATF2	path:05214_15	0.008207011
ATF2	path:05218_7	0.008207011
ATF2	path:05220_11	0.008207011
EGR1	path:05212_12	0.008365691
STAT3	path:04060_8	0.008398489
SPI1	path:04650_10	0.008430928
HNF1A	path:00120_5	0.008512637
HNF1A	path:00140_1	0.008512637
HNF1A	path:00140_22	0.008512637
HNF1A	path:00140_23	0.008512637
HNF1A	path:00140_24	0.008512637
HNF1A	path:00140_25	0.008512637
HNF1A	path:00140_26	0.008512637
HNF1A	path:00982_6	0.008512637
HNF1A	path:04610_6	0.008512637
TCF7L2	path:05200_29	0.008546233
SP1	path:04512_22	0.008608739
SP1	path:04650_7	0.008608739
SP1	path:05014_4	0.008608739
SP1	path:05140_10	0.008608739
SP1	path:05200_38	0.008608739
SP1	path:05212_14	0.008608739
SP1	path:05215_7	0.008608739
YBX1	path:04910_2	0.008638548
POU2F1	path:00980_2	0.008647196
MYC	path:05218_5	0.008651174
GATA6	path:00982_10	0.008695178
PAX6	path:04510_18	0.008727391
VDR	path:00140_11	0.008771608
VDR	path:00591_1	0.008771608
VDR	path:00982_6	0.008771608

VDR	path:04012_1	0.008771608
VDR	path:04012_11	0.008771608
VDR	path:04012_12	0.008771608
VDR	path:04012_4	0.008771608
VDR	path:04012_7	0.008771608
VDR	path:05200_9	0.008771608
VDR	path:05214_1	0.008771608
VDR	path:05214_17	0.008771608
VDR	path:05214_2	0.008771608
VDR	path:05214_3	0.008771608
VDR	path:05214_4	0.008771608
VDR	path:05214_5	0.008771608
VDR	path:05214_6	0.008771608
VDR	path:05214_8	0.008771608
VDR	path:05218_2	0.008771608
VDR	path:05223_6	0.008771608
YBX1	path:05214_1	0.008831833
YBX1	path:05214_17	0.008831833
YBX1	path:05214_2	0.008831833
YBX1	path:05214_3	0.008831833
YBX1	path:05214_4	0.008831833
YBX1	path:05214_5	0.008831833
YBX1	path:05214_6	0.008831833
YBX1	path:05214_8	0.008831833
TCF7L2	path:04630_1	0.008845159
JUN	path:04115_2	0.008970954
SP1	path:00240_5	0.009002988
IRF1	path:04062_2	0.009036068
TFAP2A	path:04520_9	0.009046641
STAT5B	path:05218_8	0.009060668
TP53	path:04010_16	0.009062653
TP53	path:04621_7	0.009062653
TP53	path:05014_4	0.009062653
TP53	path:05160_15	0.009062653

TP53	path:05219_4	0.009062653
VDR	path:04012_14	0.0090937
ATF2	path:00140_5	0.009108588
ATF2	path:04110_16	0.009108588
ATF2	path:05142_10	0.009108588
ATF2	path:05142_4	0.009108588
MYC	path:04110_22	0.009112071
MYC	path:05212_11	0.009112071
TP53	path:04510_10	0.009131774
E2F1	path:04510_11	0.009147152
E2F1	path:05222_2	0.009147152
SP1	path:04110_1	0.00916136
CEBPA	path:04610_3	0.00918123
NFIC	path:00982_2	0.00921451
SP1	path:05200_24	0.00927462
TP53	path:04510_8	0.00935253
IRF3	path:04620_17	0.009380523
IRF3	path:04620_22	0.009380523
IRF7	path:04620_17	0.009380523
IRF7	path:04620_22	0.009380523
GATA1	path:04060_47	0.009387437
VDR	path:05214_10	0.009415594
YY1	path:00140_10	0.009417621
YY1	path:00140_16	0.009417621
YY1	path:00140_18	0.009417621
YY1	path:00140_20	0.009417621
YY1	path:00140_3	0.009417621
YY1	path:00140_4	0.009417621
YY1	path:04650_7	0.009417621
YY1	path:05210_10	0.009417621
YY1	path:05212_13	0.009417621
YY1	path:05219_4	0.009417621
YY1	path:05223_3	0.009417621
TFAP2C	path:04520_9	0.009425324

STAT5B	path:04110_20	0.009500448
STAT5B	path:04110_27	0.009500448
STAT5B	path:05200_29	0.009500448
STAT5B	path:05218_5	0.009500448
IRF3	path:04620_9	0.009501335
IRF7	path:04620_9	0.009501335
MYC	path:04310_7	0.009572529
MYC	path:04630_1	0.009572529
TP53	path:04510_6	0.009576653
CREB1	path:05200_18	0.009649083
YBX1	path:04810_32	0.009732845
YBX1	path:05200_6	0.009732845
VDR	path:00980_2	0.00973729
VDR	path:00140_1	0.009786942
VDR	path:00140_25	0.009786942
VDR	path:00140_26	0.009786942
VDR	path:05215_11	0.009786942
EGR1	path:04060_8	0.009869535
SREBF1	path:00100_9	0.009882974
SREBF1	path:03320_2	0.009882974
ATF1	path:04110_18	0.009914183
YBX1	path:05214_10	0.009960619
YBX1	path:05215_10	0.009960619
TCF7L2	path:04110_18	0.009975012
YY1	path:00140_13	0.009975361
YY1	path:00140_19	0.009975361
YY1	path:00140_7	0.009975361
YY1	path:04115_4	0.009975361
YY1	path:05214_15	0.009975361
YY1	path:05218_7	0.009975361
YY1	path:05220_11	0.009975361

Table S2. The detailed information of the TFs degree distribution in the TSN.

TF_Name	Degree
AKNA	16
AR	20
ATF1	1
ATF2	36
CEBPA	4
CEBPB	9
CREB1	27
CREM	1
E2F1	33
EGR1	11
ELF1	13
ENO1	19
ESR1	8
ETV4	1
FOS	8
FOSL1	1
FOXA2	3
FOXA3	18
GATA1	1
GATA2	1
GATA3	1
GATA4	22
GATA6	9
GLI1	3
GLI3	6
HMGA1	1
HNF1A	10
HNF4A	25
IRF1	15
IRF3	12
IRF5	5
IRF7	12

JUN	25
JUNB	2
JUND	1
KLF4	31
KLF5	3
LEF1	18
MITF	2
MYC	45
NFATC1	3
NFE2L2	6
NFIC	32
NFYA	17
NR1I2	4
NR1I3	5
NR2F1	1
NR4A1	14
NR5A1	21
NR5A2	10
NRL	1
PAX6	28
POU2F1	23
RBPJ	37
REL	4
RELA	18
RFX1	21
RFX2	21
RFX3	21
RUNX3	1
SMAD2	13
SMAD3	9
SMAD4	10
SP1	186
SP3	87
SPI1	15

SREBF1	4
SRF	5
STAT1	11
STAT3	7
STAT4	1
STAT5A	1
STAT5B	17
STAT6	4
TCF7	1
TCF7L2	18
TFAP2A	46
TFAP2C	3
THRB	1
TP53	72
TP63	19
TP73	6
TXK	4
VDR	35
XBP1	21
YBX1	45
YY1	40
ZBTB7A	24
ZBTB7B	18

Table S3. The detailed information of the co-motif TFs regulated sub-pathways.

TF	Subpathway	P_value
AR	path:05220_9	0.005795
AR	path:05200_16	0.005795
AR	path:05219_4	0.006067
AR	path:05214_13	0.005795
AR	path:04115_1	0.000667
AR	path:05218_7	0.006236
AR	path:04115_2	0.005795
AR	path:04115_7	0.005795
AR	path:05215_1	0.006236
AR	path:04115_4	0.005795
AR	path:05214_14	0.005795
AR	path:05216_4	0.006067
AR	path:05200_47	0.005795
AR	path:04115_3	0.000667
AR	path:05200_51	0.006067
AR	path:04110_18	0.002649
AR	path:05214_15	0.006236
AR	path:04110_23	0.003572
AR	path:05220_12	0.005795
AR	path:05220_11	0.006236
ATF1	path:04110_18	0.009914
ATF2	path:00140_5	0.009109
ATF2	path:05219_4	0.007303
ATF2	path:04620_22	3.02E-05
ATF2	path:04110_26	0.004085
ATF2	path:04110_4	0.004085
ATF2	path:04110_3	0.004085
ATF2	path:05200_51	0.007303
ATF2	path:05142_10	0.009109
ATF2	path:04650_20	0.00458
ATF2	path:04620_17	3.02E-05
ATF2	path:00140_7	0.008207

ATF2	path:05220_12	0.006676
ATF2	path:04110_23	0.004085
ATF2	path:05020_9	0.00458
ATF2	path:05214_21	0.006676
ATF2	path:04110_18	0.003077
ATF2	path:00140_9	0.005779
ATF2	path:04620_16	0.000747
ATF2	path:05218_7	0.008207
ATF2	path:05214_15	0.008207
ATF2	path:05214_13	0.006676
ATF2	path:05200_29	0.00232
ATF2	path:04110_19	0.00458
ATF2	path:04620_9	3.02E-05
ATF2	path:05220_11	0.008207
ATF2	path:04110_16	0.009109
ATF2	path:04620_12	0.000408
ATF2	path:05212_13	0.007303
ATF2	path:04612_7	0.00458
ATF2	path:05142_4	0.009109
ATF2	path:00140_8	0.005779
ATF2	path:04110_17	0.004619
ATF2	path:05210_10	0.007303
ATF2	path:05223_3	0.007303
ATF2	path:04620_18	1.46E-05
ATF2	path:00140_18	0.006676
CEBPA	path:04610_6	0.000182
CEBPA	path:04610_4	0.000182
CEBPA	path:04610_3	0.009181
CEBPA	path:04060_54	0.004217
CEBPB	path:04062_2	0.000193
CEBPB	path:04620_12	6.69E-07
CEBPB	path:04620_17	4.38E-06
CEBPB	path:04062_1	0.000853
CEBPB	path:04620_18	9.27E-07

CEBPB	path:04620_16	9.58E-07
CEBPB	path:04060_56	1.23E-05
CEBPB	path:04620_22	4.38E-06
CEBPB	path:04620_9	4.77E-06
CREB1	path:04620_17	0.001278
CREB1	path:04514_3	0.004288
CREB1	path:05320_4	0.004131
CREB1	path:05322_6	0.004131
CREB1	path:05322_5	0.004131
CREB1	path:05320_1	0.004131
CREB1	path:05330_2	0.004131
CREB1	path:05310_3	0.004131
CREB1	path:05150_14	0.004288
CREB1	path:04620_22	0.001278
CREB1	path:04672_4	0.004131
CREB1	path:04620_16	0.004131
CREB1	path:05310_1	0.004131
CREB1	path:04620_12	0.004131
CREB1	path:05330_1	0.004131
CREB1	path:04620_9	0.001278
CREB1	path:04612_3	0.004288
CREB1	path:04514_1	0.004288
CREB1	path:04620_18	0.000628
CREB1	path:05332_1	0.004131
CREB1	path:05416_4	0.004131
CREB1	path:04940_1	0.004131
CREB1	path:05330_4	0.004131
CREB1	path:05320_3	0.004131
CREB1	path:05320_6	0.004131
CREB1	path:05200_18	0.009649
CREB1	path:04672_5	0.004131
CREM	path:05200_29	0.00082
E2F1	path:04110_22	0.000309
E2F1	path:04110_4	1.13E-07

E2F1	path:04110_26	1.13E-07
E2F1	path:05214_21	1.07E-05
E2F1	path:04110_20	2.73E-06
E2F1	path:04115_1	0.005
E2F1	path:05218_7	1.13E-07
E2F1	path:05200_52	9.20E-09
E2F1	path:05200_54	0.002554
E2F1	path:04110_3	1.13E-07
E2F1	path:05200_51	1.56E-05
E2F1	path:04110_15	0.008122
E2F1	path:05214_13	1.07E-05
E2F1	path:05222_1	3.73E-07
E2F1	path:05222_2	0.009147
E2F1	path:05215_7	1.56E-05
E2F1	path:04110_19	1.74E-07
E2F1	path:05014_4	0.00459
E2F1	path:05223_3	1.10E-07
E2F1	path:05220_12	1.07E-05
E2F1	path:05212_13	1.10E-07
E2F1	path:04110_7	1.74E-05
E2F1	path:05220_11	1.13E-07
E2F1	path:04510_11	0.009147
E2F1	path:04110_23	1.13E-07
E2F1	path:04110_17	9.20E-09
E2F1	path:04110_27	2.73E-06
E2F1	path:05214_15	1.13E-07
E2F1	path:05200_53	1.85E-07
E2F1	path:05218_8	0.000198
E2F1	path:04110_1	0.000198
E2F1	path:04110_16	0.006873
E2F1	path:05219_4	1.10E-07
EGR1	path:05218_4	0.003154
EGR1	path:04510_10	0.0007
EGR1	path:04060_8	0.00987

EGR1	path:04510_8	0.003154
EGR1	path:04510_18	0.0007
EGR1	path:04540_13	0.003154
EGR1	path:05212_12	0.008366
EGR1	path:04512_3	0.001297
EGR1	path:04510_6	0.003154
EGR1	path:05146_9	0.003154
EGR1	path:05212_11	0.007008
ELF1	path:04650_15	0.000687
ELF1	path:05310_5	0.000687
ELF1	path:04650_1	0.006321
ELF1	path:04650_14	0.001226
ELF1	path:04664_10	0.000533
ELF1	path:04664_3	0.000533
ELF1	path:05310_4	0.000687
ELF1	path:04664_2	1.05E-05
ELF1	path:04060_8	8.41E-06
ELF1	path:04664_9	0.000533
ELF1	path:04664_1	0.000687
ELF1	path:04664_5	0.000533
ELF1	path:05142_21	0.006321
ESR1	path:04115_4	0.002948
ESR1	path:04115_1	0.000345
ESR1	path:05200_29	0.000345
ESR1	path:04115_3	0.002948
ESR1	path:04610_8	0.002948
ESR1	path:04115_7	0.002948
ESR1	path:00830_2	0.005414
ESR1	path:04115_2	0.002979
ETV4	path:05200_29	0.00082
FOS	path:04620_22	0.006237
FOS	path:04610_7	0.003174
FOS	path:04620_17	0.006237
FOS	path:04115_1	0.005719

FOS	path:05200_29	2.16E-08
FOS	path:04310_7	0.006308
FOS	path:04620_9	0.006237
FOS	path:04620_18	0.003174
FOSL1	path:05200_29	0.002765
FOXA2	path:04610_6	0.000581
FOXA2	path:04610_4	0.000581
FOXA2	path:04610_3	0.000581
FOXA3	path:00982_2	5.58E-05
FOXA3	path:00982_10	7.28E-05
FOXA3	path:00982_8	2.48E-05
FOXA3	path:00980_2	0.000327
FOXA3	path:00982_4	5.10E-05
FOXA3	path:00982_9	5.10E-05
FOXA3	path:00982_3	2.48E-05
FOXA3	path:00591_1	0.000313
FOXA3	path:00140_1	0.000327
FOXA3	path:00140_12	0.000503
FOXA3	path:00982_5	5.10E-05
FOXA3	path:00140_11	0.000189
FOXA3	path:00982_6	0.000249
FOXA3	path:00140_26	0.000327
FOXA3	path:00982_11	2.48E-05
FOXA3	path:00140_25	0.000327
FOXA3	path:00830_2	0.000976
FOXA3	path:00982_7	2.48E-05
GATA1	path:04060_47	0.009387
GATA2	path:04060_47	0.002977
GATA3	path:04612_4	0.003995
GATA4	path:00140_18	0.001238
GATA4	path:00140_7	0.001238
GATA4	path:00590_1	0.00061
GATA4	path:00982_5	0.001238
GATA4	path:00140_9	0.001227

GATA4	path:00140_8	0.001227
GATA4	path:00590_3	0.00061
GATA4	path:00140_16	0.001238
GATA4	path:00982_10	0.001959
GATA4	path:00591_1	0.00061
GATA4	path:00140_5	0.00147
GATA4	path:00982_4	0.001238
GATA4	path:00140_19	0.001238
GATA4	path:00140_13	0.001238
GATA4	path:00140_6	0.001711
GATA4	path:00140_3	0.001238
GATA4	path:00590_4	0.00061
GATA4	path:00980_2	0.00061
GATA4	path:00982_9	0.001238
GATA4	path:00140_4	0.001238
GATA4	path:00140_10	0.001238
GATA4	path:00590_2	0.00061
GATA6	path:00591_1	0.001855
GATA6	path:00982_9	0.005343
GATA6	path:00590_2	0.001855
GATA6	path:00590_4	0.001855
GATA6	path:00982_10	0.008695
GATA6	path:00982_5	0.005343
GATA6	path:00590_3	0.001855
GATA6	path:00982_4	0.005343
GATA6	path:00590_1	0.001855
GLI1	path:04340_2	0.002915
GLI1	path:04510_11	0.004578
GLI1	path:05200_34	0.002915
GLI3	path:05200_34	0.000417
GLI3	path:05200_57	0.003235
GLI3	path:04340_2	0.000417
GLI3	path:04340_1	0.005356
GLI3	path:05217_2	0.003235

GLI3	path:05217_1	0.003961
HMGA1	path:04060_8	0.004641
HNF1A	path:00140_26	0.008513
HNF1A	path:00120_5	0.008513
HNF1A	path:00140_24	0.008513
HNF1A	path:04610_6	0.008513
HNF1A	path:00140_23	0.008513
HNF1A	path:00140_25	0.008513
HNF1A	path:00140_1	0.008513
HNF1A	path:00982_6	0.008513
HNF1A	path:00140_12	3.95E-05
HNF1A	path:00140_22	0.008513
HNF4A	path:00982_5	5.38E-08
HNF4A	path:00120_19	0.003813
HNF4A	path:00120_15	0.007303
HNF4A	path:04610_6	0.000179
HNF4A	path:00120_7	0.003813
HNF4A	path:00830_2	2.36E-05
HNF4A	path:00980_2	0.000734
HNF4A	path:04610_3	4.35E-06
HNF4A	path:04950_4	0.000288
HNF4A	path:00120_9	0.000101
HNF4A	path:00982_9	5.38E-08
HNF4A	path:00590_4	0.001898
HNF4A	path:04610_4	0.000101
HNF4A	path:04610_2	4.35E-06
HNF4A	path:00590_1	0.001808
HNF4A	path:00591_1	0.000516
HNF4A	path:00982_4	5.38E-08
HNF4A	path:03320_2	0.000101
HNF4A	path:03320_1	0.001132
HNF4A	path:04610_5	6.69E-06
HNF4A	path:00590_3	0.001723
HNF4A	path:04610_8	0.001699

HNF4A	path:00120_6	0.001699
HNF4A	path:00982_10	2.52E-07
HNF4A	path:00590_2	0.001723
IRF1	path:04620_22	0.004615
IRF1	path:04620_12	2.78E-05
IRF1	path:04620_16	0.002132
IRF1	path:04620_2	0.005103
IRF1	path:04620_11	0.005103
IRF1	path:04670_2	0.007172
IRF1	path:04620_1	0.000623
IRF1	path:04060_51	0.006779
IRF1	path:04620_13	0.001939
IRF1	path:04620_18	0.002013
IRF1	path:04620_9	0.004615
IRF1	path:04062_2	0.009036
IRF1	path:04620_17	0.004615
IRF1	path:05160_6	0.004615
IRF1	path:04620_14	0.001939
IRF3	path:04060_38	5.44E-05
IRF3	path:04650_6	5.75E-05
IRF3	path:04620_12	1.24E-05
IRF3	path:04620_18	0.005305
IRF3	path:04620_22	0.009381
IRF3	path:04620_16	0.0055
IRF3	path:04620_9	0.009501
IRF3	path:04620_2	3.65E-07
IRF3	path:04620_17	0.009381
IRF3	path:04620_11	3.65E-07
IRF3	path:04620_14	0.005132
IRF3	path:05160_6	5.75E-05
IRF5	path:04620_11	0.005012
IRF5	path:04060_38	0.005012
IRF5	path:04650_6	0.005012
IRF5	path:05160_6	0.005012

IRF5	path:04620_2	0.005012
IRF7	path:04620_12	1.24E-05
IRF7	path:04620_22	0.009381
IRF7	path:04620_18	0.005305
IRF7	path:04650_6	5.75E-05
IRF7	path:04060_38	5.44E-05
IRF7	path:04620_9	0.009501
IRF7	path:04620_17	0.009381
IRF7	path:04620_11	3.65E-07
IRF7	path:04620_16	0.0055
IRF7	path:04620_2	3.65E-07
IRF7	path:05160_6	5.75E-05
IRF7	path:04620_14	0.005132
JUN	path:04610_7	0.002953
JUN	path:04620_22	2.03E-05
JUN	path:04115_3	0.007635
JUN	path:04620_9	2.03E-05
JUN	path:04620_12	6.18E-05
JUN	path:05200_29	2.03E-05
JUN	path:04650_20	0.000309
JUN	path:04210_24	0.002648
JUN	path:04620_17	2.03E-05
JUN	path:05120_6	0.002953
JUN	path:04940_4	0.002953
JUN	path:05320_9	0.002953
JUN	path:04310_6	0.004859
JUN	path:04115_4	0.007635
JUN	path:04620_16	0.000159
JUN	path:05330_5	0.002953
JUN	path:05332_5	0.002953
JUN	path:04512_23	0.007569
JUN	path:04115_1	0.00139
JUN	path:04115_2	0.008971
JUN	path:04620_18	6.60E-06

JUN	path:04310_7	0.000877
JUN	path:04115_7	0.007953
JUN	path:05332_2	0.002953
JUN	path:05131_6	0.007569
JUNB	path:04350_12	0.002723
JUNB	path:04350_10	0.002723
JUND	path:05200_29	6.84E-09
KLF4	path:05200_51	0.003573
KLF4	path:05220_11	0.003573
KLF4	path:04110_3	0.001049
KLF4	path:05210_12	0.003573
KLF4	path:04512_4	0.00714
KLF4	path:04110_20	0.000691
KLF4	path:04110_26	0.001049
KLF4	path:04110_19	4.70E-05
KLF4	path:04115_1	0.003364
KLF4	path:05210_7	0.003573
KLF4	path:05214_14	0.002858
KLF4	path:05220_12	0.003364
KLF4	path:04110_4	0.001049
KLF4	path:05220_9	0.002858
KLF4	path:05218_7	0.003573
KLF4	path:05219_4	0.003573
KLF4	path:04110_27	0.000691
KLF4	path:04512_8	0.00714
KLF4	path:05214_13	0.003364
KLF4	path:04110_23	0.001049
KLF4	path:04512_1	0.007703
KLF4	path:05200_31	0.003573
KLF4	path:05200_47	0.002858
KLF4	path:05200_52	0.007104
KLF4	path:05214_15	0.003573
KLF4	path:04512_2	0.007703
KLF4	path:05145_14	0.00714

KLF4	path:04110_17	4.70E-05
KLF4	path:05200_53	0.005224
KLF4	path:04512_7	0.00714
KLF4	path:04110_7	0.001498
LEF1	path:04310_6	0.000672
LEF1	path:05222_1	0.005057
LEF1	path:04916_8	0.007826
LEF1	path:05210_7	1.30E-05
LEF1	path:05210_12	1.30E-05
LEF1	path:05200_31	1.30E-05
LEF1	path:05200_42	6.00E-05
LEF1	path:05222_2	0.004566
LEF1	path:05200_50	0.003595
LEF1	path:04310_7	0.000119
LEF1	path:05216_4	1.30E-05
LEF1	path:04916_3	0.002646
LEF1	path:05213_1	2.72E-05
LEF1	path:05221_1	5.77E-05
LEF1	path:05210_10	0.002646
LEF1	path:05222_7	0.004079
LEF1	path:05215_4	0.003117
LEF1	path:04916_11	0.005551
MITF	path:00350_1	0.000452
MITF	path:04916_3	1.75E-08
MYC	path:05214_21	0.001974
MYC	path:05200_29	7.20E-06
MYC	path:05210_7	0.002049
MYC	path:04110_26	0.000519
MYC	path:04110_3	0.000519
MYC	path:05218_5	0.008651
MYC	path:05218_3	0.002049
MYC	path:05218_7	0.002049
MYC	path:05210_10	0.002024
MYC	path:05200_31	0.002049

MYC	path:05210_12	0.002049
MYC	path:05200_51	0.002024
MYC	path:05220_9	0.00166
MYC	path:04110_7	0.000714
MYC	path:04110_17	0.000714
MYC	path:05220_11	0.002049
MYC	path:04110_22	0.009112
MYC	path:05214_14	0.00166
MYC	path:04110_4	0.000519
MYC	path:04310_7	0.009573
MYC	path:05200_53	0.002948
MYC	path:05223_3	0.002024
MYC	path:05200_52	0.003951
MYC	path:05212_11	0.009112
MYC	path:05218_8	0.007148
MYC	path:05200_42	0.006521
MYC	path:05200_50	8.52E-05
MYC	path:04110_23	0.000519
MYC	path:05212_13	0.002024
MYC	path:04110_27	0.000293
MYC	path:04110_18	0.000389
MYC	path:05222_1	8.86E-05
MYC	path:05221_1	0.005908
MYC	path:05222_2	8.52E-05
MYC	path:05222_7	8.52E-05
MYC	path:05214_13	0.001974
MYC	path:05219_4	0.002024
MYC	path:05220_12	0.001974
MYC	path:04630_1	0.009573
MYC	path:05200_21	0.002484
MYC	path:05216_4	0.002024
MYC	path:05214_15	0.002049
MYC	path:05213_1	0.003438
MYC	path:04110_20	0.000293

MYC	path:05200_47	0.00166
NFATC1	path:04650_20	0.003964
NFATC1	path:04060_32	0.003964
NFATC1	path:04060_33	0.003964
NFE2L2	path:00480_2	2.02E-06
NFE2L2	path:00480_5	0.00313
NFE2L2	path:00480_4	0.00313
NFE2L2	path:00480_1	0.00313
NFE2L2	path:00480_3	0.00313
NFE2L2	path:00480_6	0.00313
NFIC	path:05200_48	0.004397
NFIC	path:05220_8	0.004397
NFIC	path:05218_5	0.004397
NFIC	path:05200_51	0.007871
NFIC	path:05214_14	0.007051
NFIC	path:04115_1	0.008
NFIC	path:00982_9	0.007871
NFIC	path:04722_19	0.007871
NFIC	path:04115_4	0.007871
NFIC	path:05160_15	0.007871
NFIC	path:00982_7	0.004397
NFIC	path:04115_7	0.007871
NFIC	path:05220_9	0.007051
NFIC	path:05214_12	0.004397
NFIC	path:04010_30	0.007871
NFIC	path:04660_8	0.007871
NFIC	path:05200_47	0.007051
NFIC	path:00982_11	0.005989
NFIC	path:00982_2	0.009215
NFIC	path:05214_13	0.007871
NFIC	path:00591_1	0.004397
NFIC	path:04115_3	0.007871
NFIC	path:00982_8	0.004397
NFIC	path:05220_12	0.007871

NFIC	path:04080_7	0.004397
NFIC	path:04115_2	0.007871
NFIC	path:04662_6	0.007871
NFIC	path:04010_31	0.007871
NFIC	path:00982_5	0.007871
NFIC	path:00982_3	0.005989
NFIC	path:00982_4	0.007871
NFIC	path:05218_6	0.004397
NFYA	path:04110_4	0.004714
NFYA	path:04110_22	0.002132
NFYA	path:04115_7	0.000776
NFYA	path:04110_26	0.004552
NFYA	path:04115_1	0.001111
NFYA	path:04110_19	0.005565
NFYA	path:04110_12	0.003795
NFYA	path:04115_3	0.000776
NFYA	path:04110_23	0.000299
NFYA	path:04110_7	0.000341
NFYA	path:04110_1	0.001445
NFYA	path:04110_8	0.004552
NFYA	path:04110_17	0.000341
NFYA	path:04115_4	0.000776
NFYA	path:04115_2	0.000124
NFYA	path:04110_3	0.000299
NFYA	path:04110_11	0.005383
NR1I2	path:00982_5	0.000119
NR1I2	path:00830_2	0.004391
NR1I2	path:00982_4	0.000119
NR1I2	path:00982_9	0.000119
NR1I3	path:00982_5	0.000536
NR1I3	path:00982_9	0.000536
NR1I3	path:00982_4	0.000536
NR1I3	path:00830_2	0.000187
NR1I3	path:00980_1	0.006275

NR2F1	path:03320_2	0.000133
NR4A1	path:00140_8	0.000777
NR4A1	path:00140_5	0.00096
NR4A1	path:00140_10	0.000777
NR4A1	path:00140_7	0.000832
NR4A1	path:00140_4	0.000777
NR4A1	path:00140_9	0.000777
NR4A1	path:00140_6	0.00109
NR4A1	path:00140_16	0.000777
NR4A1	path:00140_14	0.000777
NR4A1	path:00140_20	0.000777
NR4A1	path:00140_18	0.000777
NR4A1	path:00140_13	6.42E-06
NR4A1	path:00140_19	0.000832
NR4A1	path:00140_3	0.000777
NR5A1	path:04350_13	0.002226
NR5A1	path:00140_23	5.77E-05
NR5A1	path:00140_22	5.77E-05
NR5A1	path:00140_16	1.15E-06
NR5A1	path:00140_24	5.77E-05
NR5A1	path:04060_21	8.93E-05
NR5A1	path:00140_4	0.000624
NR5A1	path:00140_18	1.15E-06
NR5A1	path:00140_7	1.72E-06
NR5A1	path:00140_5	2.18E-06
NR5A1	path:00140_6	2.70E-06
NR5A1	path:00140_10	1.53E-06
NR5A1	path:00140_20	0.000624
NR5A1	path:00140_9	1.09E-06
NR5A1	path:00140_8	1.09E-06
NR5A1	path:00140_19	1.72E-06
NR5A1	path:00140_13	6.63E-09
NR5A1	path:00140_14	0.000394
NR5A1	path:00140_3	0.000624

NR5A1	path:00140_12	0.000117
NR5A1	path:00140_11	1.69E-05
NR5A2	path:00140_4	0.00309
NR5A2	path:00140_18	0.00309
NR5A2	path:00140_9	0.002899
NR5A2	path:00140_11	0.000443
NR5A2	path:00140_5	0.00386
NR5A2	path:00140_12	0.002213
NR5A2	path:00140_6	0.004244
NR5A2	path:00140_7	0.003475
NR5A2	path:00140_8	0.002899
NR5A2	path:00140_3	0.00309
PAX6	path:04512_23	4.96E-05
PAX6	path:04510_6	0.000221
PAX6	path:04510_10	0.000221
PAX6	path:04514_57	6.38E-05
PAX6	path:04670_9	4.96E-05
PAX6	path:04510_18	0.008727
PAX6	path:05100_3	6.38E-05
PAX6	path:04510_20	2.77E-05
PAX6	path:04670_20	0.000615
PAX6	path:04810_29	4.23E-05
PAX6	path:04510_8	0.000221
PAX6	path:04512_24	6.38E-05
PAX6	path:04670_2	0.00217
PAX6	path:04514_56	9.92E-05
PAX6	path:04670_14	0.000678
PAX6	path:05100_10	0.000174
PAX6	path:04514_49	0.000131
PAX6	path:04510_17	0.004905
PAX6	path:04510_7	0.006714
PAX6	path:05414_2	1.40E-05
PAX6	path:05131_6	4.96E-05
PAX6	path:04510_4	5.33E-05

PAX6	path:04510_1	7.62E-06
PAX6	path:05131_5	4.96E-05
PAX6	path:04514_50	0.000131
PAX6	path:04512_9	0.004167
PAX6	path:05140_1	6.38E-05
PAX6	path:04510_19	7.62E-06
POU2F1	path:05200_52	0.00087
POU2F1	path:00590_2	0.00087
POU2F1	path:05200_51	0.000647
POU2F1	path:00591_1	0.006943
POU2F1	path:00590_1	0.00087
POU2F1	path:05200_50	0.000667
POU2F1	path:04110_19	0.000667
POU2F1	path:00982_5	0.000647
POU2F1	path:00830_2	3.54E-05
POU2F1	path:00980_2	0.008647
POU2F1	path:00982_4	0.000647
POU2F1	path:04110_17	0.000667
POU2F1	path:04664_5	0.003468
POU2F1	path:04664_2	0.007941
POU2F1	path:04110_25	0.001501
POU2F1	path:05222_1	0.00087
POU2F1	path:04110_18	0.000623
POU2F1	path:05222_2	0.00087
POU2F1	path:04110_20	0.003159
POU2F1	path:00982_9	0.000647
POU2F1	path:04110_27	0.003159
POU2F1	path:00590_3	0.00087
POU2F1	path:00590_4	0.00087
RBPJ	path:05160_17	0.001146
RBPJ	path:05215_12	0.001146
RBPJ	path:04920_1	0.002376
RBPJ	path:05140_9	0.001146
RBPJ	path:04620_25	0.001146

RBPJ	path:05222_10	0.001907
RBPJ	path:04660_1	0.00151
RBPJ	path:04620_18	0.00151
RBPJ	path:05145_5	0.001146
RBPJ	path:05215_2	0.001146
RBPJ	path:05140_13	0.001146
RBPJ	path:05142_21	0.001146
RBPJ	path:05220_6	0.001146
RBPJ	path:04920_11	0.002086
RBPJ	path:05200_18	0.002593
RBPJ	path:05142_8	0.002593
RBPJ	path:04062_19	0.001225
RBPJ	path:04622_5	0.001558
RBPJ	path:04621_4	0.0014
RBPJ	path:05160_3	0.001225
RBPJ	path:04623_5	0.001146
RBPJ	path:05131_12	0.001907
RBPJ	path:05160_8	0.001558
RBPJ	path:05215_3	0.003899
RBPJ	path:04662_1	0.001558
RBPJ	path:04722_13	0.00256
RBPJ	path:04722_24	0.001225
RBPJ	path:04210_3	0.001225
RBPJ	path:05131_9	0.001225
RBPJ	path:04620_10	0.001146
RBPJ	path:04622_3	0.001146
RBPJ	path:05120_3	0.001146
RBPJ	path:04620_9	0.002593
RBPJ	path:04620_15	0.001146
RBPJ	path:05120_6	0.001225
RBPJ	path:05222_9	0.002195
RBPJ	path:05131_10	0.002195
REL	path:04612_7	0.005201
REL	path:04060_8	0.004163

REL	path:04650_23	0.000199
REL	path:04650_22	0.000199
RELA	path:04620_22	6.51E-08
RELA	path:04062_2	3.87E-08
RELA	path:04620_18	8.85E-09
RELA	path:04620_17	6.51E-08
RELA	path:04620_9	7.78E-08
RELA	path:04620_13	1.51E-07
RELA	path:04110_7	0.001869
RELA	path:04115_1	0.000554
RELA	path:04110_17	0.001869
RELA	path:04650_23	0.001782
RELA	path:04060_62	0.000137
RELA	path:04620_1	2.11E-06
RELA	path:04620_12	4.80E-09
RELA	path:04110_19	0.001782
RELA	path:04620_14	2.07E-07
RELA	path:04650_22	0.001974
RELA	path:04620_16	1.03E-08
RELA	path:04062_1	2.04E-07
RFX1	path:05320_3	2.14E-06
RFX1	path:05330_7	5.59E-06
RFX1	path:05310_1	2.14E-06
RFX1	path:05322_6	2.14E-06
RFX1	path:05320_4	2.14E-06
RFX1	path:05330_4	2.14E-06
RFX1	path:04612_3	2.14E-06
RFX1	path:05150_14	2.14E-06
RFX1	path:05310_3	2.14E-06
RFX1	path:05330_2	2.14E-06
RFX1	path:04940_1	2.14E-06
RFX1	path:05320_6	2.14E-06
RFX1	path:04514_3	2.14E-06
RFX1	path:04672_4	2.14E-06

RFX1	path:05322_5	2.14E-06
RFX1	path:04514_1	2.14E-06
RFX1	path:04672_5	2.14E-06
RFX1	path:05330_1	2.14E-06
RFX1	path:05320_1	2.14E-06
RFX1	path:05332_1	2.14E-06
RFX1	path:05416_4	2.14E-06
RFX2	path:05330_4	3.82E-07
RFX2	path:05310_1	3.82E-07
RFX2	path:05320_1	3.82E-07
RFX2	path:04612_3	3.82E-07
RFX2	path:05322_6	3.82E-07
RFX2	path:05150_14	3.82E-07
RFX2	path:05320_4	3.82E-07
RFX2	path:05322_5	3.82E-07
RFX2	path:05330_7	1.00E-06
RFX2	path:05330_1	3.82E-07
RFX2	path:04672_4	3.82E-07
RFX2	path:05332_1	3.82E-07
RFX2	path:05330_2	3.82E-07
RFX2	path:05320_3	3.82E-07
RFX2	path:04672_5	3.82E-07
RFX2	path:04514_3	3.82E-07
RFX2	path:05310_3	3.82E-07
RFX2	path:05416_4	3.82E-07
RFX2	path:04940_1	3.82E-07
RFX2	path:04514_1	3.82E-07
RFX2	path:05320_6	3.82E-07
RFX3	path:05320_1	1.53E-07
RFX3	path:05330_7	4.00E-07
RFX3	path:04672_5	1.53E-07
RFX3	path:05330_2	1.53E-07
RFX3	path:04612_3	1.53E-07
RFX3	path:04672_4	1.53E-07

RFX3	path:04514_1	1.53E-07
RFX3	path:05330_4	1.53E-07
RFX3	path:05310_1	1.53E-07
RFX3	path:05322_5	1.53E-07
RFX3	path:05150_14	1.53E-07
RFX3	path:05310_3	1.53E-07
RFX3	path:05320_4	1.53E-07
RFX3	path:05322_6	1.53E-07
RFX3	path:05320_3	1.53E-07
RFX3	path:05330_1	1.53E-07
RFX3	path:04940_1	1.53E-07
RFX3	path:05416_4	1.53E-07
RFX3	path:05320_6	1.53E-07
RFX3	path:04514_3	1.53E-07
RFX3	path:05332_1	1.53E-07
RUNX3	path:04612_4	0.000198
SMAD2	path:04510_6	0.002748
SMAD2	path:04510_8	0.002748
SMAD2	path:05146_9	0.003683
SMAD2	path:04110_18	6.60E-05
SMAD2	path:04512_11	0.005671
SMAD2	path:04510_10	0.002748
SMAD2	path:04512_13	5.26E-05
SMAD2	path:04512_6	0.000166
SMAD2	path:04110_25	5.26E-05
SMAD2	path:04510_1	5.26E-05
SMAD2	path:04512_12	0.000122
SMAD2	path:04510_19	5.26E-05
SMAD2	path:04512_14	0.005849
SMAD3	path:04512_13	0.000922
SMAD3	path:04110_18	0.00105
SMAD3	path:04110_25	0.000922
SMAD3	path:05200_29	0.000922
SMAD3	path:04512_12	0.001326

SMAD3	path:05210_10	0.007105
SMAD3	path:04512_6	0.001837
SMAD3	path:04510_1	0.001054
SMAD3	path:04510_19	0.00117
SMAD4	path:04115_2	0.003046
SMAD4	path:04115_7	0.003046
SMAD4	path:05210_9	0.006805
SMAD4	path:04115_4	0.003046
SMAD4	path:05200_29	0.000734
SMAD4	path:04110_25	0.00062
SMAD4	path:04115_3	0.003046
SMAD4	path:04110_18	0.000744
SMAD4	path:05210_10	0.00471
SMAD4	path:04115_1	0.003366
SP1	path:04512_20	0.00215
SP1	path:04510_17	0.000141
SP1	path:04110_3	6.46E-09
SP1	path:05200_51	5.54E-09
SP1	path:05014_4	0.008609
SP1	path:00140_19	2.77E-05
SP1	path:00140_6	8.66E-05
SP1	path:05218_6	1.33E-05
SP1	path:04510_10	1.05E-10
SP1	path:04510_8	6.16E-10
SP1	path:04510_25	0.002499
SP1	path:05200_29	9.21E-07
SP1	path:05218_7	9.21E-07
SP1	path:05212_13	1.44E-05
SP1	path:05016_8	0.007434
SP1	path:05216_4	0.000441
SP1	path:04510_6	1.07E-10
SP1	path:04620_18	0.000204
SP1	path:05414_2	1.77E-05
SP1	path:04110_22	1.43E-05

SP1	path:05146_2	7.92E-07
SP1	path:04512_11	5.55E-06
SP1	path:04610_3	9.21E-07
SP1	path:05142_11	0.003931
SP1	path:05140_6	0.000246
SP1	path:05200_4	0.00029
SP1	path:00120_2	0.003886
SP1	path:04115_2	6.74E-05
SP1	path:00140_11	0.001535
SP1	path:05222_4	2.01E-05
SP1	path:00140_4	1.44E-05
SP1	path:05142_10	0.001108
SP1	path:04520_5	0.001603
SP1	path:04610_7	0.000134
SP1	path:04512_18	0.000246
SP1	path:05146_5	0.00215
SP1	path:04010_10	0.003931
SP1	path:05145_8	0.006088
SP1	path:04115_3	4.23E-05
SP1	path:04110_23	5.22E-09
SP1	path:04630_3	1.97E-06
SP1	path:04512_10	2.01E-05
SP1	path:04110_19	1.05E-10
SP1	path:05200_56	0.001015
SP1	path:05200_48	1.33E-05
SP1	path:05210_12	0.000719
SP1	path:00140_9	0.000124
SP1	path:05214_21	0.000246
SP1	path:04110_26	7.70E-08
SP1	path:05014_3	0.006088
SP1	path:04115_4	6.18E-06
SP1	path:04610_4	5.55E-06
SP1	path:05146_1	1.23E-06
SP1	path:04510_7	0.000448

SP1	path:04510_13	0.001603
SP1	path:00140_5	5.07E-05
SP1	path:04810_29	7.92E-07
SP1	path:05218_4	0.000411
SP1	path:04512_6	6.24E-07
SP1	path:04110_17	1.86E-10
SP1	path:05214_14	2.07E-08
SP1	path:04510_16	0.002771
SP1	path:04620_9	0.001894
SP1	path:05200_31	0.000719
SP1	path:00140_3	1.44E-05
SP1	path:04010_26	0.000441
SP1	path:05214_2	0.005536
SP1	path:05200_19	0.003935
SP1	path:00140_25	0.001894
SP1	path:04512_22	0.008609
SP1	path:05222_7	0.001603
SP1	path:05200_20	0.003935
SP1	path:00140_1	0.001894
SP1	path:00980_2	0.001393
SP1	path:04110_4	6.46E-09
SP1	path:04520_3	0.005126
SP1	path:00350_3	0.006192
SP1	path:04630_4	5.95E-06
SP1	path:00140_8	0.000124
SP1	path:04630_2	0.00139
SP1	path:05320_9	0.007434
SP1	path:04512_5	2.72E-07
SP1	path:04510_1	7.85E-13
SP1	path:04110_7	4.97E-07
SP1	path:05140_10	0.008609
SP1	path:05219_4	1.44E-05
SP1	path:04510_24	0.002176
SP1	path:05218_5	0.000134

SP1	path:04512_14	0.000102
SP1	path:05200_52	1.55E-08
SP1	path:05330_5	0.007434
SP1	path:05212_14	0.008609
SP1	path:04110_27	2.92E-09
SP1	path:00140_14	0.003886
SP1	path:00330_9	0.005126
SP1	path:00330_1	0.005536
SP1	path:04540_13	1.22E-05
SP1	path:04510_15	0.001165
SP1	path:00120_1	0.006088
SP1	path:00350_2	0.006192
SP1	path:04115_7	4.96E-05
SP1	path:04512_27	0.000166
SP1	path:04650_7	0.008609
SP1	path:00140_13	2.77E-05
SP1	path:05146_9	9.10E-07
SP1	path:04512_3	1.66E-05
SP1	path:05200_38	0.008609
SP1	path:05220_12	1.18E-07
SP1	path:05145_21	0.001015
SP1	path:05210_7	0.000719
SP1	path:05215_7	0.008609
SP1	path:04210_20	0.002176
SP1	path:05100_3	0.001015
SP1	path:04510_18	8.07E-08
SP1	path:04510_23	0.000719
SP1	path:04310_7	1.77E-05
SP1	path:00140_16	0.000246
SP1	path:04512_13	7.86E-06
SP1	path:04520_7	0.000412
SP1	path:05200_24	0.009275
SP1	path:00140_10	1.44E-05
SP1	path:00140_18	0.000246

SP1	path:05200_53	4.11E-06
SP1	path:05214_5	0.005536
SP1	path:04060_32	0.001015
SP1	path:05332_2	0.007434
SP1	path:05220_9	2.07E-08
SP1	path:04610_6	9.21E-07
SP1	path:05332_5	0.007434
SP1	path:04722_19	0.000719
SP1	path:04110_18	4.28E-11
SP1	path:04510_5	0.002499
SP1	path:00240_16	0.005536
SP1	path:05215_10	0.001165
SP1	path:00240_5	0.009003
SP1	path:05214_15	9.21E-07
SP1	path:00140_12	0.006088
SP1	path:05210_10	0.000441
SP1	path:04115_1	3.47E-10
SP1	path:05214_12	1.33E-05
SP1	path:05200_42	0.000719
SP1	path:05142_4	0.001108
SP1	path:05220_5	0.00215
SP1	path:00140_7	2.77E-05
SP1	path:04310_6	1.33E-05
SP1	path:04110_25	0.006192
SP1	path:04512_19	0.00055
SP1	path:04110_1	0.009161
SP1	path:05218_8	7.27E-05
SP1	path:00982_10	0.002176
SP1	path:04010_5	0.000124
SP1	path:05219_3	0.00031
SP1	path:04510_19	1.96E-12
SP1	path:04510_20	1.43E-05
SP1	path:05142_21	0.000719
SP1	path:00140_20	0.000441

SP1	path:05200_50	5.07E-05
SP1	path:05214_13	1.18E-07
SP1	path:05223_3	1.44E-05
SP1	path:04510_4	6.74E-05
SP1	path:05220_11	9.21E-07
SP1	path:05222_1	5.36E-07
SP1	path:04940_4	0.007434
SP1	path:00240_2	0.00169
SP1	path:05220_8	1.33E-05
SP1	path:00240_17	0.003391
SP1	path:05221_1	0.006192
SP1	path:05200_3	2.01E-05
SP1	path:04610_8	0.000204
SP1	path:05222_2	0.000134
SP1	path:05142_20	0.000441
SP1	path:04144_2	7.61E-06
SP1	path:04912_8	1.33E-05
SP1	path:05200_47	2.07E-08
SP1	path:04210_24	0.001108
SP1	path:04512_12	1.66E-06
SP1	path:04060_22	0.00215
SP1	path:04650_20	4.84E-05
SP1	path:05142_17	0.003886
SP1	path:04512_9	9.36E-06
SP1	path:04060_47	0.007434
SP1	path:04110_20	2.92E-09
SP1	path:05142_18	0.003886
SP1	path:00120_3	0.003886
SP1	path:05145_19	0.006088
SP1	path:04630_1	0.000232
SP3	path:04610_7	0.004573
SP3	path:05200_52	1.23E-05
SP3	path:04510_10	1.24E-07
SP3	path:05200_51	8.65E-07

SP3	path:05212_13	0.001367
SP3	path:04110_3	3.13E-05
SP3	path:04512_18	0.000926
SP3	path:05218_7	5.63E-05
SP3	path:00140_11	5.63E-05
SP3	path:04510_6	1.24E-07
SP3	path:00140_26	0.005691
SP3	path:05146_2	1.13E-06
SP3	path:04512_27	0.000424
SP3	path:04110_17	3.14E-07
SP3	path:04512_11	0.000758
SP3	path:00140_9	0.000618
SP3	path:00140_6	0.000126
SP3	path:04110_22	0.001276
SP3	path:04510_18	6.32E-05
SP3	path:05144_3	0.002642
SP3	path:00140_13	5.63E-05
SP3	path:04110_26	0.000307
SP3	path:04510_1	3.35E-07
SP3	path:04110_23	3.02E-05
SP3	path:00140_19	5.63E-05
SP3	path:05200_53	0.003635
SP3	path:00140_4	4.16E-05
SP3	path:05200_48	0.000156
SP3	path:04110_18	0.000128
SP3	path:05214_14	0.000618
SP3	path:05214_15	5.63E-05
SP3	path:05220_12	3.02E-05
SP3	path:00140_10	4.16E-05
SP3	path:00140_16	0.000926
SP3	path:05220_9	0.000618
SP3	path:00120_1	0.000926
SP3	path:00120_2	0.000618
SP3	path:00140_7	5.63E-05

SP3	path:05414_2	0.001439
SP3	path:04810_29	0.000815
SP3	path:05146_9	1.78E-05
SP3	path:04512_14	0.000907
SP3	path:04115_1	0.004062
SP3	path:05223_3	0.001367
SP3	path:04512_5	2.26E-07
SP3	path:00140_5	8.41E-05
SP3	path:04512_6	5.63E-05
SP3	path:05218_8	0.000758
SP3	path:00140_1	0.006217
SP3	path:04110_4	3.13E-05
SP3	path:04510_20	0.004573
SP3	path:04110_7	0.007662
SP3	path:05146_1	1.74E-06
SP3	path:05220_11	5.63E-05
SP3	path:05219_4	4.16E-05
SP3	path:05214_13	3.02E-05
SP3	path:04110_19	2.26E-07
SP3	path:04510_7	0.002878
SP3	path:00982_10	0.004573
SP3	path:00140_12	0.000126
SP3	path:04512_9	7.41E-05
SP3	path:04512_10	0.000488
SP3	path:04060_43	0.002642
SP3	path:00140_8	0.000618
SP3	path:00140_25	0.006217
SP3	path:00140_18	0.000926
SP3	path:04510_19	6.37E-07
SP3	path:05219_3	0.006779
SP3	path:04512_3	3.50E-05
SP3	path:00140_20	0.001367
SP3	path:04512_13	5.52E-05
SP3	path:05222_1	0.005691

SP3	path:05218_6	0.000156
SP3	path:04510_17	0.001367
SP3	path:04144_2	0.000815
SP3	path:04110_27	4.71E-06
SP3	path:04512_19	0.000542
SP3	path:04512_12	3.50E-05
SP3	path:04610_8	0.005691
SP3	path:05214_12	0.000156
SP3	path:05220_8	0.000156
SP3	path:00120_3	0.000618
SP3	path:00140_3	4.16E-05
SP3	path:04115_4	0.002219
SP3	path:05200_47	0.000618
SP3	path:04110_20	4.71E-06
SP3	path:04510_8	1.24E-07
SPI1	path:05200_43	0.000903
SPI1	path:04145_6	3.11E-06
SPI1	path:04650_13	0.003806
SPI1	path:05140_4	0.00011
SPI1	path:04514_55	0.007631
SPI1	path:05150_17	0.005349
SPI1	path:04650_12	0.007341
SPI1	path:04145_2	0.005349
SPI1	path:04060_41	0.001532
SPI1	path:04650_10	0.008431
SPI1	path:04514_62	0.003571
SPI1	path:04670_2	0.000252
SPI1	path:04670_14	0.001532
SPI1	path:04670_10	0.003571
SPI1	path:04670_15	0.000961
SREBF1	path:04910_6	0.001432
SREBF1	path:00100_9	0.009883
SREBF1	path:04910_5	3.27E-05
SREBF1	path:03320_2	0.009883

SRF	path:05020_8	0.004546
SRF	path:05416_1	0.003034
SRF	path:05020_9	0.00379
SRF	path:05414_3	0.003034
SRF	path:05410_2	0.003034
STAT1	path:04620_9	0.00382
STAT1	path:04620_18	0.002114
STAT1	path:04620_22	0.00382
STAT1	path:05200_29	0.00382
STAT1	path:04620_16	0.002114
STAT1	path:04630_4	0.001501
STAT1	path:05210_10	0.001313
STAT1	path:04145_5	0.006088
STAT1	path:04630_3	0.006088
STAT1	path:04110_18	0.005143
STAT1	path:04620_17	0.00382
STAT3	path:04060_8	0.008398
STAT3	path:04630_4	5.36E-05
STAT3	path:04630_3	0.007945
STAT3	path:04060_3	0.007358
STAT3	path:04630_2	0.007945
STAT3	path:05142_4	0.000768
STAT3	path:05200_29	0.000109
STAT4	path:04630_4	0.000257
STAT5A	path:04630_4	0.00037
STAT5B	path:05200_52	0.005013
STAT5B	path:05218_8	0.009061
STAT5B	path:05214_15	0.002527
STAT5B	path:05220_11	0.002527
STAT5B	path:05220_9	0.002527
STAT5B	path:05218_7	0.002527
STAT5B	path:05214_14	0.002527
STAT5B	path:05219_4	0.002527
STAT5B	path:05200_47	0.002527

STAT5B	path:04110_27	0.0095
STAT5B	path:04630_4	0.00037
STAT5B	path:05218_5	0.0095
STAT5B	path:05214_13	0.002527
STAT5B	path:05200_51	0.002527
STAT5B	path:05200_29	0.0095
STAT5B	path:05220_12	0.002527
STAT5B	path:04110_20	0.0095
STAT6	path:04630_2	0.000657
STAT6	path:04630_4	0.000657
STAT6	path:00590_4	0.003995
STAT6	path:04630_3	0.000657
TCF7	path:04660_20	0.006692
TCF7L2	path:05200_31	4.20E-06
TCF7L2	path:05200_42	2.26E-05
TCF7L2	path:05222_2	0.003078
TCF7L2	path:04110_18	0.009975
TCF7L2	path:05222_1	0.003394
TCF7L2	path:04310_7	1.19E-09
TCF7L2	path:05216_4	4.20E-06
TCF7L2	path:05210_7	4.20E-06
TCF7L2	path:05200_50	0.00245
TCF7L2	path:05221_1	2.10E-05
TCF7L2	path:05215_4	0.002139
TCF7L2	path:05213_1	9.42E-06
TCF7L2	path:05210_12	4.20E-06
TCF7L2	path:05222_7	0.002763
TCF7L2	path:04630_1	0.008845
TCF7L2	path:05200_29	0.008546
TCF7L2	path:05210_10	0.00183
TCF7L2	path:04310_6	1.48E-08
TFAP2A	path:05214_16	0.00774
TFAP2A	path:05214_7	0.00774
TFAP2A	path:05214_11	0.002408

TFAP2A	path:05214_4	0.004059
TFAP2A	path:05214_10	0.000317
TFAP2A	path:04510_15	0.005458
TFAP2A	path:04520_5	0.000575
TFAP2A	path:05215_8	0.001125
TFAP2A	path:05200_10	2.20E-05
TFAP2A	path:04510_18	1.42E-05
TFAP2A	path:04510_10	0.001269
TFAP2A	path:04080_7	0.003857
TFAP2A	path:04510_16	0.003333
TFAP2A	path:04520_2	0.002408
TFAP2A	path:04510_5	0.00774
TFAP2A	path:05214_6	0.004059
TFAP2A	path:05215_11	0.000437
TFAP2A	path:05214_3	0.004059
TFAP2A	path:04510_25	0.00774
TFAP2A	path:05219_2	0.002408
TFAP2A	path:04510_6	0.001276
TFAP2A	path:05214_8	0.004059
TFAP2A	path:05200_11	1.91E-05
TFAP2A	path:05214_1	0.004059
TFAP2A	path:04144_2	0.000575
TFAP2A	path:05214_2	0.004059
TFAP2A	path:05200_5	2.22E-05
TFAP2A	path:05200_12	1.91E-05
TFAP2A	path:04520_3	0.001263
TFAP2A	path:05200_15	6.62E-06
TFAP2A	path:05200_21	0.000449
TFAP2A	path:04912_8	0.003857
TFAP2A	path:04510_8	0.001272
TFAP2A	path:05200_29	8.35E-05
TFAP2A	path:05200_4	1.91E-05
TFAP2A	path:05200_8	2.16E-06
TFAP2A	path:05214_17	0.004059

TFAP2A	path:05214_5	0.004059
TFAP2A	path:05212_12	0.003333
TFAP2A	path:04520_9	0.009047
TFAP2A	path:04510_17	3.81E-05
TFAP2A	path:05215_10	0.000317
TFAP2A	path:04520_7	0.001263
TFAP2A	path:04510_7	8.35E-05
TFAP2A	path:05212_11	0.002699
TFAP2A	path:04020_2	0.005724
TFAP2C	path:05219_2	0.004045
TFAP2C	path:04912_8	0.004045
TFAP2C	path:04520_9	0.009425
THRB	path:04080_2	0.000625
TP53	path:04010_15	0.007703
TP53	path:04510_6	0.009577
TP53	path:05200_20	0.000117
TP53	path:04510_25	0.006952
TP53	path:05200_15	0.000968
TP53	path:04115_7	0
TP53	path:05160_15	0.009063
TP53	path:04210_23	0.000276
TP53	path:04110_23	0.000199
TP53	path:05200_48	1.95E-05
TP53	path:05214_10	0.000211
TP53	path:04010_21	0.007443
TP53	path:04110_22	7.56E-05
TP53	path:04110_8	0.004291
TP53	path:05218_8	0.001385
TP53	path:05200_5	0.000367
TP53	path:05220_12	0.000117
TP53	path:05215_11	0.000276
TP53	path:05200_11	0.000276
TP53	path:04210_20	0.000422
TP53	path:05200_8	1.95E-05

TP53	path:05220_9	7.56E-05
TP53	path:05215_3	0.003315
TP53	path:05219_4	0.009063
TP53	path:04510_16	0.002467
TP53	path:05218_6	1.95E-05
TP53	path:05214_12	1.95E-05
TP53	path:04510_5	0.006952
TP53	path:05160_12	0.007459
TP53	path:05200_12	0.000276
TP53	path:04110_17	3.28E-07
TP53	path:04722_19	0.000218
TP53	path:04115_1	0
TP53	path:04110_4	0.004511
TP53	path:05014_4	0.009063
TP53	path:05214_4	0.003581
TP53	path:05214_13	0.000117
TP53	path:05215_10	0.004511
TP53	path:05200_51	0.000172
TP53	path:05214_1	0.003581
TP53	path:04510_17	0.000581
TP53	path:04510_7	0.001111
TP53	path:04012_12	0.002467
TP53	path:04110_7	4.42E-09
TP53	path:05218_5	0.001775
TP53	path:04115_3	0
TP53	path:05200_4	0.000276
TP53	path:04510_10	0.009132
TP53	path:04115_2	0
TP53	path:04110_27	0.001943
TP53	path:04912_8	0.002757
TP53	path:05218_2	0.002754
TP53	path:04510_15	0.004511
TP53	path:04010_16	0.009063
TP53	path:05214_11	0.001775

TP53	path:04110_19	1.48E-05
TP53	path:04115_4	0
TP53	path:05219_3	0.001692
TP53	path:04110_20	0.001943
TP53	path:05200_47	7.56E-05
TP53	path:05200_10	0.000338
TP53	path:04110_1	2.55E-11
TP53	path:04510_8	0.009353
TP53	path:05213_2	0.007459
TP53	path:04621_7	0.009063
TP53	path:05200_17	0.003927
TP53	path:05214_14	7.56E-05
TP53	path:05220_8	1.95E-05
TP53	path:04510_18	0.001775
TP53	path:05200_19	0.002998
TP53	path:04110_3	0.000211
TP53	path:04210_24	0.000276
TP63	path:04115_1	8.12E-05
TP63	path:05220_9	0.000159
TP63	path:04110_7	8.12E-05
TP63	path:05215_11	0.004121
TP63	path:04115_7	8.12E-05
TP63	path:04110_1	2.23E-05
TP63	path:05200_48	8.12E-05
TP63	path:05214_12	8.12E-05
TP63	path:05218_6	8.12E-05
TP63	path:05200_20	0.002783
TP63	path:05200_19	0.002783
TP63	path:04115_4	8.12E-05
TP63	path:04115_3	8.12E-05
TP63	path:05215_3	0.002837
TP63	path:05220_8	8.12E-05
TP63	path:05200_17	0.003116
TP63	path:04115_2	8.12E-05

TP63	path:05200_47	0.000159
TP63	path:05214_14	0.000159
TP73	path:04115_1	6.75E-05
TP73	path:04722_19	0.000595
TP73	path:04115_4	6.67E-05
TP73	path:04115_2	6.67E-05
TP73	path:04115_3	6.67E-05
TP73	path:04115_7	6.67E-05
VDR	path:00591_1	0.008772
VDR	path:00140_11	0.008772
VDR	path:05214_10	0.009416
VDR	path:00982_2	0.003675
VDR	path:00140_1	0.009787
VDR	path:00980_2	0.009737
VDR	path:05214_1	0.008772
VDR	path:00982_7	0.001636
VDR	path:05214_3	0.008772
VDR	path:05200_9	0.008772
VDR	path:00140_26	0.009787
VDR	path:00982_10	0.004788
VDR	path:00982_9	0.003361
VDR	path:00140_25	0.009787
VDR	path:05218_2	0.008772
VDR	path:05214_4	0.008772
VDR	path:00982_8	0.001636
VDR	path:04012_14	0.009094
VDR	path:04012_11	0.008772
VDR	path:00982_5	0.003361
VDR	path:05223_6	0.008772
VDR	path:05215_11	0.009787
VDR	path:04012_4	0.008772
VDR	path:00982_4	0.003361
VDR	path:05214_8	0.008772
VDR	path:05214_5	0.008772

VDR	path:05214_17	0.008772
VDR	path:04012_7	0.008772
VDR	path:04012_12	0.008772
VDR	path:00982_3	0.001636
VDR	path:00982_6	0.008772
VDR	path:00982_11	0.001636
VDR	path:05214_2	0.008772
VDR	path:04012_1	0.008772
VDR	path:05214_6	0.008772
XBP1	path:05320_4	0.000179
XBP1	path:05330_7	0.000327
XBP1	path:04672_4	0.000179
XBP1	path:05310_1	0.000179
XBP1	path:05416_4	0.000179
XBP1	path:04940_1	0.000179
XBP1	path:05322_5	0.000179
XBP1	path:04672_5	0.000179
XBP1	path:05332_1	0.000179
XBP1	path:05330_2	0.000179
XBP1	path:05320_6	0.000179
XBP1	path:05150_14	0.000179
XBP1	path:05310_3	0.000179
XBP1	path:04514_3	0.000179
XBP1	path:05322_6	0.000179
XBP1	path:05320_1	0.000179
XBP1	path:04612_3	0.000179
XBP1	path:04514_1	0.000179
XBP1	path:05320_3	0.000179
XBP1	path:05330_1	0.000179
XBP1	path:05330_4	0.000179
YY1	path:00140_4	0.009418
YY1	path:05219_4	0.009418
YY1	path:05214_15	0.009975
YY1	path:00140_16	0.009418

YY1	path:00140_13	0.009975
YY1	path:05214_13	0.000411
YY1	path:00140_18	0.009418
YY1	path:00140_8	0.007589
YY1	path:05223_3	0.009418
YY1	path:00140_19	0.009975
YY1	path:05214_14	0.007589
YY1	path:00140_10	0.009418
YY1	path:05212_13	0.009418
YY1	path:04110_23	0.005923
YY1	path:05200_48	0.005152
YY1	path:00140_7	0.009975
YY1	path:00140_9	0.007589
YY1	path:00140_3	0.009418
YY1	path:05200_47	0.007589
YY1	path:05220_9	0.007589
YY1	path:04115_4	0.009975
YY1	path:04110_20	0.003726
YY1	path:00140_20	0.009418
YY1	path:00140_14	0.007589
YY1	path:04110_19	0.000476
YY1	path:04110_7	0.007589
YY1	path:05218_7	0.009975
YY1	path:04110_17	0.000486
YY1	path:04650_7	0.009418
YY1	path:05200_51	0.000438
YY1	path:05218_8	0.003139
YY1	path:05219_3	0.003726
YY1	path:05210_10	0.009418
YY1	path:05220_11	0.009975
YY1	path:05214_12	0.005152
YY1	path:05218_6	0.005152
YY1	path:04650_20	0.007431
YY1	path:05220_8	0.005152

YY1	path:05220_12	0.000411
YY1	path:04110_27	0.003726
ZBTB7A	path:04110_22	0.001224
ZBTB7A	path:04110_18	0.001519
ZBTB7A	path:05200_52	0.000644
ZBTB7A	path:04350_6	0.000801
ZBTB7A	path:04110_25	7.64E-06
ZBTB7A	path:05218_7	0.000397
ZBTB7A	path:04110_4	0.001957
ZBTB7A	path:04110_1	0.001011
ZBTB7A	path:04110_23	0.001957
ZBTB7A	path:04110_20	0.001224
ZBTB7A	path:04110_26	0.001957
ZBTB7A	path:04110_3	0.001957
ZBTB7A	path:05214_13	0.000397
ZBTB7A	path:05220_11	0.000397
ZBTB7A	path:05222_2	0.000644
ZBTB7A	path:04110_27	9.07E-06
ZBTB7A	path:04110_19	0.002272
ZBTB7A	path:04110_17	0.002453
ZBTB7A	path:05218_8	0.001011
ZBTB7A	path:05214_15	0.000397
ZBTB7A	path:05219_4	0.000397
ZBTB7A	path:05200_51	0.000397
ZBTB7A	path:05222_1	0.000644
ZBTB7A	path:05220_12	0.000397
ZBTB7B	path:04510_8	0.00284
ZBTB7B	path:04512_5	0.000476
ZBTB7B	path:05146_9	0.000424
ZBTB7B	path:04512_9	0.000537
ZBTB7B	path:04512_10	0.000476
ZBTB7B	path:04512_14	0.000424
ZBTB7B	path:04512_3	0.000476
ZBTB7B	path:04510_10	0.00284

ZBTB7B	path:04512_11	0.000424
ZBTB7B	path:04512_12	0.000476
ZBTB7B	path:04510_19	0.00284
ZBTB7B	path:04510_1	0.00284
ZBTB7B	path:04510_6	0.00284
ZBTB7B	path:05146_1	0.000476
ZBTB7B	path:04512_13	0.000424
ZBTB7B	path:04512_6	0.000521
ZBTB7B	path:05146_2	0.000476
ZBTB7B	path:04512_27	0.000476

Table S4. The detailed information of the co-family TFs regulated sub-pathways.

Family	TF	Subpathway	P_Value
AP-1(-like) components	ATF2	path:00140_18	0.006675857
AP-1(-like) components	ATF2	path:00140_5	0.009108588
AP-1(-like) components	ATF2	path:00140_7	0.008207011
AP-1(-like) components	ATF2	path:00140_8	0.005778895
AP-1(-like) components	ATF2	path:00140_9	0.005778895
AP-1(-like) components	ATF2	path:04110_16	0.009108588
AP-1(-like) components	ATF2	path:04110_17	0.004619377
AP-1(-like) components	ATF2	path:04110_18	0.003077219
AP-1(-like) components	ATF2	path:04110_19	0.004580141
AP-1(-like) components	ATF2	path:04110_23	0.004084659
AP-1(-like) components	ATF2	path:04110_26	0.004084659
AP-1(-like) components	ATF2	path:04110_3	0.004084659
AP-1(-like) components	ATF2	path:04110_4	0.004084659
AP-1(-like) components	ATF2	path:04612_7	0.004580141
AP-1(-like) components	ATF2	path:04620_12	0.000407893
AP-1(-like) components	ATF2	path:04620_16	0.000747082
AP-1(-like) components	ATF2	path:04620_17	3.02E-05
AP-1(-like) components	ATF2	path:04620_18	1.46E-05
AP-1(-like) components	ATF2	path:04620_22	3.02E-05
AP-1(-like) components	ATF2	path:04620_9	3.02E-05
AP-1(-like) components	ATF2	path:04650_20	0.004580141
AP-1(-like) components	ATF2	path:05020_9	0.004580141
AP-1(-like) components	ATF2	path:05142_10	0.009108588
AP-1(-like) components	ATF2	path:05142_4	0.009108588
AP-1(-like) components	ATF2	path:05200_29	0.002319558
AP-1(-like) components	ATF2	path:05200_51	0.007303382
AP-1(-like) components	ATF2	path:05210_10	0.007303382
AP-1(-like) components	ATF2	path:05212_13	0.007303382
AP-1(-like) components	ATF2	path:05214_13	0.006675857
AP-1(-like) components	ATF2	path:05214_15	0.008207011
AP-1(-like) components	ATF2	path:05214_21	0.006675857
AP-1(-like) components	ATF2	path:05218_7	0.008207011
AP-1(-like) components	ATF2	path:05219_4	0.007303382

AP-1(-like) components	ATF2	path:05220_11	0.008207011
AP-1(-like) components	ATF2	path:05220_12	0.006675857
AP-1(-like) components	ATF2	path:05223_3	0.007303382
AP-1(-like) components	FOS	path:04115_1	0.005719136
AP-1(-like) components	FOS	path:04310_7	0.006308233
AP-1(-like) components	FOS	path:04610_7	0.003173805
AP-1(-like) components	FOS	path:04620_17	0.006237239
AP-1(-like) components	FOS	path:04620_18	0.003173805
AP-1(-like) components	FOS	path:04620_22	0.006237239
AP-1(-like) components	FOS	path:04620_9	0.006237239
AP-1(-like) components	FOS	path:05200_29	2.16E-08
AP-1(-like) components	JUN	path:04115_1	0.001389686
AP-1(-like) components	JUN	path:04115_2	0.008970954
AP-1(-like) components	JUN	path:04115_3	0.007635426
AP-1(-like) components	JUN	path:04115_4	0.007635426
AP-1(-like) components	JUN	path:04115_7	0.007953336
AP-1(-like) components	JUN	path:04210_24	0.002648312
AP-1(-like) components	JUN	path:04310_6	0.004858763
AP-1(-like) components	JUN	path:04310_7	0.000877028
AP-1(-like) components	JUN	path:04512_23	0.007569048
AP-1(-like) components	JUN	path:04610_7	0.002952732
AP-1(-like) components	JUN	path:04620_12	6.18E-05
AP-1(-like) components	JUN	path:04620_16	0.000158904
AP-1(-like) components	JUN	path:04620_17	2.03E-05
AP-1(-like) components	JUN	path:04620_18	6.60E-06
AP-1(-like) components	JUN	path:04620_22	2.03E-05
AP-1(-like) components	JUN	path:04620_9	2.03E-05
AP-1(-like) components	JUN	path:04650_20	0.000308777
AP-1(-like) components	JUN	path:04940_4	0.002952732
AP-1(-like) components	JUN	path:05120_6	0.002952732
AP-1(-like) components	JUN	path:05131_6	0.007569048
AP-1(-like) components	JUN	path:05200_29	2.03E-05
AP-1(-like) components	JUN	path:05320_9	0.002952732
AP-1(-like) components	JUN	path:05330_5	0.002952732

AP-1(-like) components	JUN	path:05332_2	0.002952732
AP-1(-like) components	JUN	path:05332_5	0.002952732
AP-1(-like) components	JUNB	path:04350_10	0.002723194
AP-1(-like) components	JUNB	path:04350_12	0.002723194
AP-1(-like) components	JUND	path:05200_29	6.84E-09
AP-1(-like) components	NFE2L2	path:00480_1	0.003129559
AP-1(-like) components	NFE2L2	path:00480_2	2.02E-06
AP-1(-like) components	NFE2L2	path:00480_3	0.003129559
AP-1(-like) components	NFE2L2	path:00480_4	0.003129559
AP-1(-like) components	NFE2L2	path:00480_5	0.003129559
AP-1(-like) components	NFE2L2	path:00480_6	0.003129559
AP-1(-like) components	NRL	path:04744_2	0.00090302
AP-1(-like) components	XBP1	path:04514_1	0.000178543
AP-1(-like) components	XBP1	path:04514_3	0.000178543
AP-1(-like) components	XBP1	path:04612_3	0.000178543
AP-1(-like) components	XBP1	path:04672_4	0.000178543
AP-1(-like) components	XBP1	path:04672_5	0.000178543
AP-1(-like) components	XBP1	path:04940_1	0.000178543
AP-1(-like) components	XBP1	path:05150_14	0.000178543
AP-1(-like) components	XBP1	path:05310_1	0.000178543
AP-1(-like) components	XBP1	path:05310_3	0.000178543
AP-1(-like) components	XBP1	path:05320_1	0.000178543
AP-1(-like) components	XBP1	path:05320_3	0.000178543
AP-1(-like) components	XBP1	path:05320_4	0.000178543
AP-1(-like) components	XBP1	path:05320_6	0.000178543
AP-1(-like) components	XBP1	path:05322_5	0.000178543
AP-1(-like) components	XBP1	path:05322_6	0.000178543
AP-1(-like) components	XBP1	path:05330_1	0.000178543
AP-1(-like) components	XBP1	path:05330_2	0.000178543
AP-1(-like) components	XBP1	path:05330_4	0.000178543
AP-1(-like) components	XBP1	path:05330_7	0.000327268
AP-1(-like) components	XBP1	path:05332_1	0.000178543
AP-1(-like) components	XBP1	path:05416_4	0.000178543
AP-2	TFAP2A	path:04020_2	0.005723968

AP-2	TFAP2A	path:04080_7	0.003857334
AP-2	TFAP2A	path:04144_2	0.00057548
AP-2	TFAP2A	path:04510_10	0.001269093
AP-2	TFAP2A	path:04510_15	0.005458314
AP-2	TFAP2A	path:04510_16	0.003332624
AP-2	TFAP2A	path:04510_17	3.81E-05
AP-2	TFAP2A	path:04510_18	1.42E-05
AP-2	TFAP2A	path:04510_25	0.007739985
AP-2	TFAP2A	path:04510_5	0.007739985
AP-2	TFAP2A	path:04510_6	0.001276164
AP-2	TFAP2A	path:04510_7	8.35E-05
AP-2	TFAP2A	path:04510_8	0.001271633
AP-2	TFAP2A	path:04520_2	0.002407854
AP-2	TFAP2A	path:04520_3	0.001262516
AP-2	TFAP2A	path:04520_5	0.00057548
AP-2	TFAP2A	path:04520_7	0.001262516
AP-2	TFAP2A	path:04520_9	0.009046641
AP-2	TFAP2A	path:04912_8	0.003857334
AP-2	TFAP2A	path:05200_10	2.20E-05
AP-2	TFAP2A	path:05200_11	1.91E-05
AP-2	TFAP2A	path:05200_12	1.91E-05
AP-2	TFAP2A	path:05200_15	6.62E-06
AP-2	TFAP2A	path:05200_21	0.000448647
AP-2	TFAP2A	path:05200_29	8.35E-05
AP-2	TFAP2A	path:05200_4	1.91E-05
AP-2	TFAP2A	path:05200_5	2.22E-05
AP-2	TFAP2A	path:05200_8	2.16E-06
AP-2	TFAP2A	path:05212_11	0.002699451
AP-2	TFAP2A	path:05212_12	0.003332624
AP-2	TFAP2A	path:05214_1	0.004058678
AP-2	TFAP2A	path:05214_10	0.000317116
AP-2	TFAP2A	path:05214_11	0.002407854
AP-2	TFAP2A	path:05214_16	0.007739985
AP-2	TFAP2A	path:05214_17	0.004058678

AP-2	TFAP2A	path:05214_2	0.004058678
AP-2	TFAP2A	path:05214_3	0.004058678
AP-2	TFAP2A	path:05214_4	0.004058678
AP-2	TFAP2A	path:05214_5	0.004058678
AP-2	TFAP2A	path:05214_6	0.004058678
AP-2	TFAP2A	path:05214_7	0.007739985
AP-2	TFAP2A	path:05214_8	0.004058678
AP-2	TFAP2A	path:05215_10	0.000317116
AP-2	TFAP2A	path:05215_11	0.000436788
AP-2	TFAP2A	path:05215_8	0.001124594
AP-2	TFAP2A	path:05219_2	0.002407854
AP-2	TFAP2C	path:04520_9	0.009425324
AP-2	TFAP2C	path:04912_8	0.00404499
AP-2	TFAP2C	path:05219_2	0.00404499
C/EBP-like factors	CEBPA	path:04060_54	0.0042174
C/EBP-like factors	CEBPA	path:04610_3	0.00918123
C/EBP-like factors	CEBPA	path:04610_4	0.00018207
C/EBP-like factors	CEBPA	path:04610_6	0.00018207
C/EBP-like factors	CEBPB	path:04060_56	1.23E-05
C/EBP-like factors	CEBPB	path:04062_1	0.000853445
C/EBP-like factors	CEBPB	path:04062_2	0.000193217
C/EBP-like factors	CEBPB	path:04620_12	6.69E-07
C/EBP-like factors	CEBPB	path:04620_16	9.58E-07
C/EBP-like factors	CEBPB	path:04620_17	4.38E-06
C/EBP-like factors	CEBPB	path:04620_18	9.27E-07
C/EBP-like factors	CEBPB	path:04620_22	4.38E-06
C/EBP-like factors	CEBPB	path:04620_9	4.77E-06
Cell-cycle controlling factors	E2F1	path:04110_1	0.000197534
Cell-cycle controlling factors	E2F1	path:04110_15	0.008121608
Cell-cycle controlling factors	E2F1	path:04110_16	0.006872543
Cell-cycle controlling factors	E2F1	path:04110_17	9.20E-09
Cell-cycle controlling factors	E2F1	path:04110_19	1.74E-07
Cell-cycle controlling factors	E2F1	path:04110_20	2.73E-06
Cell-cycle controlling factors	E2F1	path:04110_22	0.000308684

Cell-cycle controlling factors	E2F1	path:04110_23	1.13E-07
Cell-cycle controlling factors	E2F1	path:04110_26	1.13E-07
Cell-cycle controlling factors	E2F1	path:04110_27	2.73E-06
Cell-cycle controlling factors	E2F1	path:04110_3	1.13E-07
Cell-cycle controlling factors	E2F1	path:04110_4	1.13E-07
Cell-cycle controlling factors	E2F1	path:04110_7	1.74E-05
Cell-cycle controlling factors	E2F1	path:04115_1	0.00499983
Cell-cycle controlling factors	E2F1	path:04510_11	0.009147152
Cell-cycle controlling factors	E2F1	path:05014_4	0.004589831
Cell-cycle controlling factors	E2F1	path:05200_51	1.56E-05
Cell-cycle controlling factors	E2F1	path:05200_52	9.20E-09
Cell-cycle controlling factors	E2F1	path:05200_53	1.85E-07
Cell-cycle controlling factors	E2F1	path:05200_54	0.002554435
Cell-cycle controlling factors	E2F1	path:05212_13	1.10E-07
Cell-cycle controlling factors	E2F1	path:05214_13	1.07E-05
Cell-cycle controlling factors	E2F1	path:05214_15	1.13E-07
Cell-cycle controlling factors	E2F1	path:05214_21	1.07E-05
Cell-cycle controlling factors	E2F1	path:05215_7	1.56E-05
Cell-cycle controlling factors	E2F1	path:05218_7	1.13E-07
Cell-cycle controlling factors	E2F1	path:05218_8	0.000197534
Cell-cycle controlling factors	E2F1	path:05219_4	1.10E-07
Cell-cycle controlling factors	E2F1	path:05220_11	1.13E-07
Cell-cycle controlling factors	E2F1	path:05220_12	1.07E-05
Cell-cycle controlling factors	E2F1	path:05222_1	3.73E-07
Cell-cycle controlling factors	E2F1	path:05222_2	0.009147152
Cell-cycle controlling factors	E2F1	path:05223_3	1.10E-07
Cell-cycle controlling factors	MYC	path:04110_17	0.000714486
Cell-cycle controlling factors	MYC	path:04110_18	0.00038916
Cell-cycle controlling factors	MYC	path:04110_20	0.000292768
Cell-cycle controlling factors	MYC	path:04110_22	0.009112071
Cell-cycle controlling factors	MYC	path:04110_23	0.000518594
Cell-cycle controlling factors	MYC	path:04110_26	0.000518594
Cell-cycle controlling factors	MYC	path:04110_27	0.000292768
Cell-cycle controlling factors	MYC	path:04110_3	0.000518594

Cell-cycle controlling factors	MYC	path:04110_4	0.000518594
Cell-cycle controlling factors	MYC	path:04110_7	0.000714486
Cell-cycle controlling factors	MYC	path:04310_7	0.009572529
Cell-cycle controlling factors	MYC	path:04630_1	0.009572529
Cell-cycle controlling factors	MYC	path:05200_21	0.002484447
Cell-cycle controlling factors	MYC	path:05200_29	7.20E-06
Cell-cycle controlling factors	MYC	path:05200_31	0.002048915
Cell-cycle controlling factors	MYC	path:05200_42	0.006520738
Cell-cycle controlling factors	MYC	path:05200_47	0.00165995
Cell-cycle controlling factors	MYC	path:05200_50	8.52E-05
Cell-cycle controlling factors	MYC	path:05200_51	0.002023761
Cell-cycle controlling factors	MYC	path:05200_52	0.003950679
Cell-cycle controlling factors	MYC	path:05200_53	0.002948165
Cell-cycle controlling factors	MYC	path:05210_10	0.002023761
Cell-cycle controlling factors	MYC	path:05210_12	0.002048915
Cell-cycle controlling factors	MYC	path:05210_7	0.002048915
Cell-cycle controlling factors	MYC	path:05212_11	0.009112071
Cell-cycle controlling factors	MYC	path:05212_13	0.002023761
Cell-cycle controlling factors	MYC	path:05213_1	0.003437632
Cell-cycle controlling factors	MYC	path:05214_13	0.001974254
Cell-cycle controlling factors	MYC	path:05214_14	0.00165995
Cell-cycle controlling factors	MYC	path:05214_15	0.002048915
Cell-cycle controlling factors	MYC	path:05214_21	0.001974254
Cell-cycle controlling factors	MYC	path:05216_4	0.002023761
Cell-cycle controlling factors	MYC	path:05218_3	0.002048915
Cell-cycle controlling factors	MYC	path:05218_5	0.008651174
Cell-cycle controlling factors	MYC	path:05218_7	0.002048915
Cell-cycle controlling factors	MYC	path:05218_8	0.007148499
Cell-cycle controlling factors	MYC	path:05219_4	0.002023761
Cell-cycle controlling factors	MYC	path:05220_11	0.002048915
Cell-cycle controlling factors	MYC	path:05220_12	0.001974254
Cell-cycle controlling factors	MYC	path:05220_9	0.00165995
Cell-cycle controlling factors	MYC	path:05221_1	0.005908255
Cell-cycle controlling factors	MYC	path:05222_1	8.86E-05

Cell-cycle controlling factors	MYC	path:05222_2	8.52E-05
Cell-cycle controlling factors	MYC	path:05222_7	8.52E-05
Cell-cycle controlling factors	MYC	path:05223_3	0.002023761
CREB	ATF1	path:04110_18	0.009914183
CREB	CREB1	path:04514_1	0.004288489
CREB	CREB1	path:04514_3	0.004288489
CREB	CREB1	path:04612_3	0.004288489
CREB	CREB1	path:04620_12	0.004131401
CREB	CREB1	path:04620_16	0.004131401
CREB	CREB1	path:04620_17	0.001277819
CREB	CREB1	path:04620_18	0.000627719
CREB	CREB1	path:04620_22	0.001277819
CREB	CREB1	path:04620_9	0.001277819
CREB	CREB1	path:04672_4	0.004131401
CREB	CREB1	path:04672_5	0.004131401
CREB	CREB1	path:04940_1	0.004131401
CREB	CREB1	path:05150_14	0.004288489
CREB	CREB1	path:05200_18	0.009649083
CREB	CREB1	path:05310_1	0.004131401
CREB	CREB1	path:05310_3	0.004131401
CREB	CREB1	path:05320_1	0.004131401
CREB	CREB1	path:05320_3	0.004131401
CREB	CREB1	path:05320_4	0.004131401
CREB	CREB1	path:05320_6	0.004131401
CREB	CREB1	path:05322_5	0.004131401
CREB	CREB1	path:05322_6	0.004131401
CREB	CREB1	path:05330_1	0.004131401
CREB	CREB1	path:05330_2	0.004131401
CREB	CREB1	path:05330_4	0.004131401
CREB	CREB1	path:05332_1	0.004131401
CREB	CREB1	path:05416_4	0.004131401
CREB	CREM	path:05200_29	0.000820491
csd	YBX1	path:04012_5	0.007005212
csd	YBX1	path:04510_10	0.005634473

csd	YBX1	path:04510_15	0.000802879
csd	YBX1	path:04510_16	0.00062354
csd	YBX1	path:04510_17	0.001170159
csd	YBX1	path:04510_18	0.002082955
csd	YBX1	path:04510_25	0.000802879
csd	YBX1	path:04510_5	0.000802879
csd	YBX1	path:04510_6	0.005634473
csd	YBX1	path:04510_7	0.001493429
csd	YBX1	path:04510_8	0.005634473
csd	YBX1	path:04520_2	0.007005212
csd	YBX1	path:04520_3	0.005634473
csd	YBX1	path:04520_5	0.005634473
csd	YBX1	path:04520_7	0.005634473
csd	YBX1	path:04810_32	0.009732845
csd	YBX1	path:04910_2	0.008638548
csd	YBX1	path:05100_15	0.005634473
csd	YBX1	path:05100_2	0.005634473
csd	YBX1	path:05200_6	0.009732845
csd	YBX1	path:05200_9	0.005634473
csd	YBX1	path:05211_1	0.005634473
csd	YBX1	path:05211_2	0.005634473
csd	YBX1	path:05211_7	0.005634473
csd	YBX1	path:05211_8	0.005634473
csd	YBX1	path:05212_1	0.00609256
csd	YBX1	path:05212_11	0.007005212
csd	YBX1	path:05212_12	0.008169375
csd	YBX1	path:05213_6	0.005634473
csd	YBX1	path:05214_1	0.008831833
csd	YBX1	path:05214_10	0.009960619
csd	YBX1	path:05214_11	0.007005212
csd	YBX1	path:05214_17	0.008831833
csd	YBX1	path:05214_2	0.008831833
csd	YBX1	path:05214_3	0.008831833
csd	YBX1	path:05214_4	0.008831833

csd	YBX1	path:05214_5	0.008831833
csd	YBX1	path:05214_6	0.008831833
csd	YBX1	path:05214_8	0.008831833
csd	YBX1	path:05215_10	0.009960619
csd	YBX1	path:05218_2	0.00062354
csd	YBX1	path:05218_4	0.001493429
csd	YBX1	path:05223_5	0.005634473
csd	YBX1	path:05223_6	0.007005212
csd	YBX1	path:05223_7	0.005634473
Developmental / cell cycle regulators	EGR1	path:04060_8	0.009869535
Developmental / cell cycle regulators	EGR1	path:04510_10	0.00069989
Developmental / cell cycle regulators	EGR1	path:04510_18	0.00069989
Developmental / cell cycle regulators	EGR1	path:04510_6	0.003153788
Developmental / cell cycle regulators	EGR1	path:04510_8	0.003153788
Developmental / cell cycle regulators	EGR1	path:04512_3	0.00129686
Developmental / cell cycle regulators	EGR1	path:04540_13	0.003153788
Developmental / cell cycle regulators	EGR1	path:05146_9	0.003153788
Developmental / cell cycle regulators	EGR1	path:05212_11	0.007008495
Developmental / cell cycle regulators	EGR1	path:05212_12	0.008365691
Developmental / cell cycle regulators	EGR1	path:05218_4	0.003153788
Developmental / cell cycle regulators	GLI1	path:04340_2	0.002914772
Developmental / cell cycle regulators	GLI1	path:04510_11	0.004577555
Developmental / cell cycle regulators	GLI1	path:05200_34	0.002914772
Developmental / cell cycle regulators	GLI3	path:04340_1	0.005356158
Developmental / cell cycle regulators	GLI3	path:04340_2	0.000416702
Developmental / cell cycle regulators	GLI3	path:05200_34	0.000416702
Developmental / cell cycle regulators	GLI3	path:05200_57	0.003234702
Developmental / cell cycle regulators	GLI3	path:05217_1	0.003961103
Developmental / cell cycle regulators	GLI3	path:05217_2	0.003234702
Developmental / cell cycle regulators	KLF4	path:04110_17	4.70E-05
Developmental / cell cycle regulators	KLF4	path:04110_19	4.70E-05
Developmental / cell cycle regulators	KLF4	path:04110_20	0.000691257
Developmental / cell cycle regulators	KLF4	path:04110_23	0.001048885
Developmental / cell cycle regulators	KLF4	path:04110_26	0.001048885

Developmental / cell cycle regulators	KLF4	path:04110_27	0.000691257
Developmental / cell cycle regulators	KLF4	path:04110_3	0.001048885
Developmental / cell cycle regulators	KLF4	path:04110_4	0.001048885
Developmental / cell cycle regulators	KLF4	path:04110_7	0.001498093
Developmental / cell cycle regulators	KLF4	path:04115_1	0.003364391
Developmental / cell cycle regulators	KLF4	path:04512_1	0.007702669
Developmental / cell cycle regulators	KLF4	path:04512_2	0.007702669
Developmental / cell cycle regulators	KLF4	path:04512_4	0.007140402
Developmental / cell cycle regulators	KLF4	path:04512_7	0.007140402
Developmental / cell cycle regulators	KLF4	path:04512_8	0.007140402
Developmental / cell cycle regulators	KLF4	path:05145_14	0.007140402
Developmental / cell cycle regulators	KLF4	path:05200_31	0.003572583
Developmental / cell cycle regulators	KLF4	path:05200_47	0.002858346
Developmental / cell cycle regulators	KLF4	path:05200_51	0.003572583
Developmental / cell cycle regulators	KLF4	path:05200_52	0.007103944
Developmental / cell cycle regulators	KLF4	path:05200_53	0.005224296
Developmental / cell cycle regulators	KLF4	path:05210_12	0.003572583
Developmental / cell cycle regulators	KLF4	path:05210_7	0.003572583
Developmental / cell cycle regulators	KLF4	path:05214_13	0.003364391
Developmental / cell cycle regulators	KLF4	path:05214_14	0.002858346
Developmental / cell cycle regulators	KLF4	path:05214_15	0.003572583
Developmental / cell cycle regulators	KLF4	path:05218_7	0.003572583
Developmental / cell cycle regulators	KLF4	path:05219_4	0.003572583
Developmental / cell cycle regulators	KLF4	path:05220_11	0.003572583
Developmental / cell cycle regulators	KLF4	path:05220_12	0.003364391
Developmental / cell cycle regulators	KLF4	path:05220_9	0.002858346
Developmental / cell cycle regulators	KLF5	path:05200_31	0.000357162
Developmental / cell cycle regulators	KLF5	path:05210_12	0.000357162
Developmental / cell cycle regulators	KLF5	path:05210_7	0.000357162
Ets-type	ELF1	path:04060_8	8.41E-06
Ets-type	ELF1	path:04650_1	0.006320724
Ets-type	ELF1	path:04650_14	0.001225713
Ets-type	ELF1	path:04650_15	0.000687264
Ets-type	ELF1	path:04664_1	0.000687264

Ets-type	ELF1	path:04664_10	0.000533253
Ets-type	ELF1	path:04664_2	1.05E-05
Ets-type	ELF1	path:04664_3	0.000533253
Ets-type	ELF1	path:04664_5	0.000533253
Ets-type	ELF1	path:04664_9	0.000533253
Ets-type	ELF1	path:05142_21	0.006320724
Ets-type	ELF1	path:05310_4	0.000687264
Ets-type	ELF1	path:05310_5	0.000687264
Ets-type	ETV4	path:05200_29	0.000820491
Ets-type	SPI1	path:04060_41	0.00153244
Ets-type	SPI1	path:04145_2	0.005348786
Ets-type	SPI1	path:04145_6	3.11E-06
Ets-type	SPI1	path:04514_55	0.007630608
Ets-type	SPI1	path:04514_62	0.003570772
Ets-type	SPI1	path:04650_10	0.008430928
Ets-type	SPI1	path:04650_12	0.007340609
Ets-type	SPI1	path:04650_13	0.003805584
Ets-type	SPI1	path:04670_10	0.003570772
Ets-type	SPI1	path:04670_14	0.00153244
Ets-type	SPI1	path:04670_15	0.000961365
Ets-type	SPI1	path:04670_2	0.000252274
Ets-type	SPI1	path:05140_4	0.000110411
Ets-type	SPI1	path:05150_17	0.005348786
Ets-type	SPI1	path:05200_43	0.000902646
Fungal regulators	HMGA1	path:04060_8	0.004640977
GATA-Factors	GATA1	path:04060_47	0.009387437
GATA-Factors	GATA2	path:04060_47	0.002976989
GATA-Factors	GATA3	path:04612_4	0.003994597
GATA-Factors	GATA4	path:00140_10	0.001238279
GATA-Factors	GATA4	path:00140_13	0.001238279
GATA-Factors	GATA4	path:00140_16	0.001238279
GATA-Factors	GATA4	path:00140_18	0.001238279
GATA-Factors	GATA4	path:00140_19	0.001238279
GATA-Factors	GATA4	path:00140_3	0.001238279

GATA-Factors	GATA4	path:00140_4	0.001238279
GATA-Factors	GATA4	path:00140_5	0.001470007
GATA-Factors	GATA4	path:00140_6	0.001710596
GATA-Factors	GATA4	path:00140_7	0.001238279
GATA-Factors	GATA4	path:00140_8	0.001226506
GATA-Factors	GATA4	path:00140_9	0.001226506
GATA-Factors	GATA4	path:00590_1	0.00061049
GATA-Factors	GATA4	path:00590_2	0.00061049
GATA-Factors	GATA4	path:00590_3	0.00061049
GATA-Factors	GATA4	path:00590_4	0.00061049
GATA-Factors	GATA4	path:00591_1	0.00061049
GATA-Factors	GATA4	path:00980_2	0.00061049
GATA-Factors	GATA4	path:00982_10	0.001958811
GATA-Factors	GATA4	path:00982_4	0.001238279
GATA-Factors	GATA4	path:00982_5	0.001238279
GATA-Factors	GATA4	path:00982_9	0.001238279
GATA-Factors	GATA6	path:00590_1	0.00185453
GATA-Factors	GATA6	path:00590_2	0.00185453
GATA-Factors	GATA6	path:00590_3	0.00185453
GATA-Factors	GATA6	path:00590_4	0.00185453
GATA-Factors	GATA6	path:00591_1	0.00185453
GATA-Factors	GATA6	path:00982_10	0.008695178
GATA-Factors	GATA6	path:00982_4	0.005342537
GATA-Factors	GATA6	path:00982_5	0.005342537
GATA-Factors	GATA6	path:00982_9	0.005342537
Heteromeric CCAAT factors	NFYA	path:04110_1	0.00144529
Heteromeric CCAAT factors	NFYA	path:04110_11	0.005383343
Heteromeric CCAAT factors	NFYA	path:04110_12	0.003794515
Heteromeric CCAAT factors	NFYA	path:04110_17	0.000340548
Heteromeric CCAAT factors	NFYA	path:04110_19	0.005565492
Heteromeric CCAAT factors	NFYA	path:04110_22	0.002132484
Heteromeric CCAAT factors	NFYA	path:04110_23	0.000299258
Heteromeric CCAAT factors	NFYA	path:04110_26	0.004551637
Heteromeric CCAAT factors	NFYA	path:04110_3	0.000299258

Heteromeric CCAAT factors	NFYA	path:04110_4	0.004713566
Heteromeric CCAAT factors	NFYA	path:04110_7	0.000340548
Heteromeric CCAAT factors	NFYA	path:04110_8	0.004551637
Heteromeric CCAAT factors	NFYA	path:04115_1	0.001111281
Heteromeric CCAAT factors	NFYA	path:04115_2	0.000123767
Heteromeric CCAAT factors	NFYA	path:04115_3	0.000776011
Heteromeric CCAAT factors	NFYA	path:04115_4	0.000776011
Heteromeric CCAAT factors	NFYA	path:04115_7	0.000776011
Homeo domain only	HNF1A	path:00120_5	0.008512637
Homeo domain only	HNF1A	path:00140_1	0.008512637
Homeo domain only	HNF1A	path:00140_12	3.95E-05
Homeo domain only	HNF1A	path:00140_22	0.008512637
Homeo domain only	HNF1A	path:00140_23	0.008512637
Homeo domain only	HNF1A	path:00140_24	0.008512637
Homeo domain only	HNF1A	path:00140_25	0.008512637
Homeo domain only	HNF1A	path:00140_26	0.008512637
Homeo domain only	HNF1A	path:00982_6	0.008512637
Homeo domain only	HNF1A	path:04610_6	0.008512637
Interferon-regulating factors	IRF1	path:04060_51	0.00677924
Interferon-regulating factors	IRF1	path:04062_2	0.009036068
Interferon-regulating factors	IRF1	path:04620_1	0.000622656
Interferon-regulating factors	IRF1	path:04620_11	0.005102853
Interferon-regulating factors	IRF1	path:04620_12	2.78E-05
Interferon-regulating factors	IRF1	path:04620_13	0.00193948
Interferon-regulating factors	IRF1	path:04620_14	0.00193948
Interferon-regulating factors	IRF1	path:04620_16	0.002131661
Interferon-regulating factors	IRF1	path:04620_17	0.004615096
Interferon-regulating factors	IRF1	path:04620_18	0.002013452
Interferon-regulating factors	IRF1	path:04620_2	0.005102853
Interferon-regulating factors	IRF1	path:04620_22	0.004615096
Interferon-regulating factors	IRF1	path:04620_9	0.004615096
Interferon-regulating factors	IRF1	path:04670_2	0.00717245
Interferon-regulating factors	IRF1	path:05160_6	0.004615096
Interferon-regulating factors	IRF3	path:04060_38	5.44E-05

Interferon-regulating factors	IRF3	path:04620_11	3.65E-07
Interferon-regulating factors	IRF3	path:04620_12	1.24E-05
Interferon-regulating factors	IRF3	path:04620_14	0.005131788
Interferon-regulating factors	IRF3	path:04620_16	0.005500251
Interferon-regulating factors	IRF3	path:04620_17	0.009380523
Interferon-regulating factors	IRF3	path:04620_18	0.005305274
Interferon-regulating factors	IRF3	path:04620_2	3.65E-07
Interferon-regulating factors	IRF3	path:04620_22	0.009380523
Interferon-regulating factors	IRF3	path:04620_9	0.009501335
Interferon-regulating factors	IRF3	path:04650_6	5.75E-05
Interferon-regulating factors	IRF3	path:05160_6	5.75E-05
Interferon-regulating factors	IRF5	path:04060_38	0.005011517
Interferon-regulating factors	IRF5	path:04620_11	0.005011517
Interferon-regulating factors	IRF5	path:04620_2	0.005011517
Interferon-regulating factors	IRF5	path:04650_6	0.005011517
Interferon-regulating factors	IRF5	path:05160_6	0.005011517
Interferon-regulating factors	IRF7	path:04060_38	5.44E-05
Interferon-regulating factors	IRF7	path:04620_11	3.65E-07
Interferon-regulating factors	IRF7	path:04620_12	1.24E-05
Interferon-regulating factors	IRF7	path:04620_14	0.005131788
Interferon-regulating factors	IRF7	path:04620_16	0.005500251
Interferon-regulating factors	IRF7	path:04620_17	0.009380523
Interferon-regulating factors	IRF7	path:04620_18	0.005305274
Interferon-regulating factors	IRF7	path:04620_2	3.65E-07
Interferon-regulating factors	IRF7	path:04620_22	0.009380523
Interferon-regulating factors	IRF7	path:04620_9	0.009501335
Interferon-regulating factors	IRF7	path:04650_6	5.75E-05
Interferon-regulating factors	IRF7	path:05160_6	5.75E-05
NF-1	NFIC	path:00591_1	0.004396514
NF-1	NFIC	path:00982_11	0.005988829
NF-1	NFIC	path:00982_2	0.00921451
NF-1	NFIC	path:00982_3	0.005988829
NF-1	NFIC	path:00982_4	0.00787147
NF-1	NFIC	path:00982_5	0.00787147

NF-1	NFIC	path:00982_7	0.004396514
NF-1	NFIC	path:00982_8	0.004396514
NF-1	NFIC	path:00982_9	0.00787147
NF-1	NFIC	path:04010_30	0.00787147
NF-1	NFIC	path:04010_31	0.00787147
NF-1	NFIC	path:04080_7	0.004396514
NF-1	NFIC	path:04115_1	0.008000492
NF-1	NFIC	path:04115_2	0.00787147
NF-1	NFIC	path:04115_3	0.00787147
NF-1	NFIC	path:04115_4	0.00787147
NF-1	NFIC	path:04115_7	0.00787147
NF-1	NFIC	path:04660_8	0.00787147
NF-1	NFIC	path:04662_6	0.00787147
NF-1	NFIC	path:04722_19	0.00787147
NF-1	NFIC	path:05160_15	0.00787147
NF-1	NFIC	path:05200_47	0.007050709
NF-1	NFIC	path:05200_48	0.004396514
NF-1	NFIC	path:05200_51	0.00787147
NF-1	NFIC	path:05214_12	0.004396514
NF-1	NFIC	path:05214_13	0.00787147
NF-1	NFIC	path:05214_14	0.007050709
NF-1	NFIC	path:05218_5	0.004396514
NF-1	NFIC	path:05218_6	0.004396514
NF-1	NFIC	path:05220_12	0.00787147
NF-1	NFIC	path:05220_8	0.004396514
NF-1	NFIC	path:05220_9	0.007050709
NF-AT	NFATC1	path:04060_32	0.003964221
NF-AT	NFATC1	path:04060_33	0.003964221
NF-AT	NFATC1	path:04650_20	0.003964221
p53-like	TP53	path:04010_15	0.007702858
p53-like	TP53	path:04010_16	0.009062653
p53-like	TP53	path:04010_21	0.007443002
p53-like	TP53	path:04012_12	0.002466645
p53-like	TP53	path:04110_1	2.55E-11

p53-like	TP53	path:04110_17	3.28E-07
p53-like	TP53	path:04110_19	1.48E-05
p53-like	TP53	path:04110_20	0.001943417
p53-like	TP53	path:04110_22	7.56E-05
p53-like	TP53	path:04110_23	0.000198814
p53-like	TP53	path:04110_27	0.001943417
p53-like	TP53	path:04110_3	0.000210553
p53-like	TP53	path:04110_4	0.004511216
p53-like	TP53	path:04110_7	4.42E-09
p53-like	TP53	path:04110_8	0.004291125
p53-like	TP53	path:04115_1	0
p53-like	TP53	path:04115_2	0
p53-like	TP53	path:04115_3	0
p53-like	TP53	path:04115_4	0
p53-like	TP53	path:04115_7	0
p53-like	TP53	path:04210_20	0.000421718
p53-like	TP53	path:04210_23	0.000275763
p53-like	TP53	path:04210_24	0.000275763
p53-like	TP53	path:04510_10	0.009131774
p53-like	TP53	path:04510_15	0.004511216
p53-like	TP53	path:04510_16	0.002466645
p53-like	TP53	path:04510_17	0.000580605
p53-like	TP53	path:04510_18	0.001774645
p53-like	TP53	path:04510_25	0.006951959
p53-like	TP53	path:04510_5	0.006951959
p53-like	TP53	path:04510_6	0.009576653
p53-like	TP53	path:04510_7	0.001110697
p53-like	TP53	path:04510_8	0.00935253
p53-like	TP53	path:04621_7	0.009062653
p53-like	TP53	path:04722_19	0.000218217
p53-like	TP53	path:04912_8	0.002756828
p53-like	TP53	path:05014_4	0.009062653
p53-like	TP53	path:05160_12	0.007459365
p53-like	TP53	path:05160_15	0.009062653

p53-like	TP53	path:05200_10	0.000338058
p53-like	TP53	path:05200_11	0.000275763
p53-like	TP53	path:05200_12	0.000275763
p53-like	TP53	path:05200_15	0.000968321
p53-like	TP53	path:05200_17	0.00392722
p53-like	TP53	path:05200_19	0.002997803
p53-like	TP53	path:05200_20	0.00011707
p53-like	TP53	path:05200_4	0.000275763
p53-like	TP53	path:05200_47	7.56E-05
p53-like	TP53	path:05200_48	1.95E-05
p53-like	TP53	path:05200_5	0.000367024
p53-like	TP53	path:05200_51	0.000172314
p53-like	TP53	path:05200_8	1.95E-05
p53-like	TP53	path:05213_2	0.007459365
p53-like	TP53	path:05214_1	0.003580594
p53-like	TP53	path:05214_10	0.000210553
p53-like	TP53	path:05214_11	0.001774645
p53-like	TP53	path:05214_12	1.95E-05
p53-like	TP53	path:05214_13	0.00011707
p53-like	TP53	path:05214_14	7.56E-05
p53-like	TP53	path:05214_4	0.003580594
p53-like	TP53	path:05215_10	0.004511216
p53-like	TP53	path:05215_11	0.000275763
p53-like	TP53	path:05215_3	0.00331496
p53-like	TP53	path:05218_2	0.002754132
p53-like	TP53	path:05218_5	0.001774645
p53-like	TP53	path:05218_6	1.95E-05
p53-like	TP53	path:05218_8	0.001385048
p53-like	TP53	path:05219_3	0.001691972
p53-like	TP53	path:05219_4	0.009062653
p53-like	TP53	path:05220_12	0.00011707
p53-like	TP53	path:05220_8	1.95E-05
p53-like	TP53	path:05220_9	7.56E-05
p53-like	TP63	path:04110_1	2.23E-05

p53-like	TP63	path:04110_7	8.12E-05
p53-like	TP63	path:04115_1	8.12E-05
p53-like	TP63	path:04115_2	8.12E-05
p53-like	TP63	path:04115_3	8.12E-05
p53-like	TP63	path:04115_4	8.12E-05
p53-like	TP63	path:04115_7	8.12E-05
p53-like	TP63	path:05200_17	0.003115907
p53-like	TP63	path:05200_19	0.002783081
p53-like	TP63	path:05200_20	0.002783081
p53-like	TP63	path:05200_47	0.000159403
p53-like	TP63	path:05200_48	8.12E-05
p53-like	TP63	path:05214_12	8.12E-05
p53-like	TP63	path:05214_14	0.000159403
p53-like	TP63	path:05215_11	0.004120782
p53-like	TP63	path:05215_3	0.002837304
p53-like	TP63	path:05218_6	8.12E-05
p53-like	TP63	path:05220_8	8.12E-05
p53-like	TP63	path:05220_9	0.000159403
p53-like	TP73	path:04115_1	6.75E-05
p53-like	TP73	path:04115_2	6.67E-05
p53-like	TP73	path:04115_3	6.67E-05
p53-like	TP73	path:04115_4	6.67E-05
p53-like	TP73	path:04115_7	6.67E-05
p53-like	TP73	path:04722_19	0.000595015
Paired plus homeo domain	PAX6	path:04510_1	7.62E-06
Paired plus homeo domain	PAX6	path:04510_10	0.000221093
Paired plus homeo domain	PAX6	path:04510_17	0.004905129
Paired plus homeo domain	PAX6	path:04510_18	0.008727391
Paired plus homeo domain	PAX6	path:04510_19	7.62E-06
Paired plus homeo domain	PAX6	path:04510_20	2.77E-05
Paired plus homeo domain	PAX6	path:04510_4	5.33E-05
Paired plus homeo domain	PAX6	path:04510_6	0.000221093
Paired plus homeo domain	PAX6	path:04510_7	0.006713786
Paired plus homeo domain	PAX6	path:04510_8	0.000221093

Paired plus homeo domain	PAX6	path:04512_23	4.96E-05
Paired plus homeo domain	PAX6	path:04512_24	6.38E-05
Paired plus homeo domain	PAX6	path:04512_9	0.004167242
Paired plus homeo domain	PAX6	path:04514_49	0.000131273
Paired plus homeo domain	PAX6	path:04514_50	0.000131273
Paired plus homeo domain	PAX6	path:04514_56	9.92E-05
Paired plus homeo domain	PAX6	path:04514_57	6.38E-05
Paired plus homeo domain	PAX6	path:04670_14	0.00067845
Paired plus homeo domain	PAX6	path:04670_2	0.002170272
Paired plus homeo domain	PAX6	path:04670_20	0.000614792
Paired plus homeo domain	PAX6	path:04670_9	4.96E-05
Paired plus homeo domain	PAX6	path:04810_29	4.23E-05
Paired plus homeo domain	PAX6	path:05100_10	0.000173551
Paired plus homeo domain	PAX6	path:05100_3	6.38E-05
Paired plus homeo domain	PAX6	path:05131_5	4.96E-05
Paired plus homeo domain	PAX6	path:05131_6	4.96E-05
Paired plus homeo domain	PAX6	path:05140_1	6.38E-05
Paired plus homeo domain	PAX6	path:05414_2	1.40E-05
POU domain factors	POU2F1	path:00590_1	0.000870331
POU domain factors	POU2F1	path:00590_2	0.000870331
POU domain factors	POU2F1	path:00590_3	0.000870331
POU domain factors	POU2F1	path:00590_4	0.000870331
POU domain factors	POU2F1	path:00591_1	0.006943045
POU domain factors	POU2F1	path:00830_2	3.54E-05
POU domain factors	POU2F1	path:00980_2	0.008647196
POU domain factors	POU2F1	path:00982_4	0.000646745
POU domain factors	POU2F1	path:00982_5	0.000646745
POU domain factors	POU2F1	path:00982_9	0.000646745
POU domain factors	POU2F1	path:04110_17	0.000667343
POU domain factors	POU2F1	path:04110_18	0.000623378
POU domain factors	POU2F1	path:04110_19	0.000667343
POU domain factors	POU2F1	path:04110_20	0.0031585
POU domain factors	POU2F1	path:04110_25	0.001500996
POU domain factors	POU2F1	path:04110_27	0.0031585

POU domain factors	POU2F1	path:04664_2	0.007941331
POU domain factors	POU2F1	path:04664_5	0.003467546
POU domain factors	POU2F1	path:05200_50	0.000667343
POU domain factors	POU2F1	path:05200_51	0.000646745
POU domain factors	POU2F1	path:05200_52	0.000870331
POU domain factors	POU2F1	path:05222_1	0.000870331
POU domain factors	POU2F1	path:05222_2	0.000870331
Regulators with a DNA recognition wing	RFX1	path:04514_1	2.14E-06
Regulators with a DNA recognition wing	RFX1	path:04514_3	2.14E-06
Regulators with a DNA recognition wing	RFX1	path:04612_3	2.14E-06
Regulators with a DNA recognition wing	RFX1	path:04672_4	2.14E-06
Regulators with a DNA recognition wing	RFX1	path:04672_5	2.14E-06
Regulators with a DNA recognition wing	RFX1	path:04940_1	2.14E-06
Regulators with a DNA recognition wing	RFX1	path:05150_14	2.14E-06
Regulators with a DNA recognition wing	RFX1	path:05310_1	2.14E-06
Regulators with a DNA recognition wing	RFX1	path:05310_3	2.14E-06
Regulators with a DNA recognition wing	RFX1	path:05320_1	2.14E-06
Regulators with a DNA recognition wing	RFX1	path:05320_3	2.14E-06
Regulators with a DNA recognition wing	RFX1	path:05320_4	2.14E-06
Regulators with a DNA recognition wing	RFX1	path:05320_6	2.14E-06
Regulators with a DNA recognition wing	RFX1	path:05322_5	2.14E-06
Regulators with a DNA recognition wing	RFX1	path:05322_6	2.14E-06
Regulators with a DNA recognition wing	RFX1	path:05330_1	2.14E-06
Regulators with a DNA recognition wing	RFX1	path:05330_2	2.14E-06
Regulators with a DNA recognition wing	RFX1	path:05330_4	2.14E-06
Regulators with a DNA recognition wing	RFX1	path:05330_7	5.59E-06
Regulators with a DNA recognition wing	RFX1	path:05332_1	2.14E-06
Regulators with a DNA recognition wing	RFX1	path:05416_4	2.14E-06
Regulators with a DNA recognition wing	RFX2	path:04514_1	3.82E-07
Regulators with a DNA recognition wing	RFX2	path:04514_3	3.82E-07
Regulators with a DNA recognition wing	RFX2	path:04612_3	3.82E-07
Regulators with a DNA recognition wing	RFX2	path:04672_4	3.82E-07
Regulators with a DNA recognition wing	RFX2	path:04672_5	3.82E-07
Regulators with a DNA recognition wing	RFX2	path:04940_1	3.82E-07

Regulators with a DNA recognition wing	RFX2	path:05150_14	3.82E-07
Regulators with a DNA recognition wing	RFX2	path:05310_1	3.82E-07
Regulators with a DNA recognition wing	RFX2	path:05310_3	3.82E-07
Regulators with a DNA recognition wing	RFX2	path:05320_1	3.82E-07
Regulators with a DNA recognition wing	RFX2	path:05320_3	3.82E-07
Regulators with a DNA recognition wing	RFX2	path:05320_4	3.82E-07
Regulators with a DNA recognition wing	RFX2	path:05320_6	3.82E-07
Regulators with a DNA recognition wing	RFX2	path:05322_5	3.82E-07
Regulators with a DNA recognition wing	RFX2	path:05322_6	3.82E-07
Regulators with a DNA recognition wing	RFX2	path:05330_1	3.82E-07
Regulators with a DNA recognition wing	RFX2	path:05330_2	3.82E-07
Regulators with a DNA recognition wing	RFX2	path:05330_4	3.82E-07
Regulators with a DNA recognition wing	RFX2	path:05330_7	1.00E-06
Regulators with a DNA recognition wing	RFX2	path:05332_1	3.82E-07
Regulators with a DNA recognition wing	RFX2	path:05416_4	3.82E-07
Regulators with a DNA recognition wing	RFX3	path:04514_1	1.53E-07
Regulators with a DNA recognition wing	RFX3	path:04514_3	1.53E-07
Regulators with a DNA recognition wing	RFX3	path:04612_3	1.53E-07
Regulators with a DNA recognition wing	RFX3	path:04672_4	1.53E-07
Regulators with a DNA recognition wing	RFX3	path:04672_5	1.53E-07
Regulators with a DNA recognition wing	RFX3	path:04940_1	1.53E-07
Regulators with a DNA recognition wing	RFX3	path:05150_14	1.53E-07
Regulators with a DNA recognition wing	RFX3	path:05310_1	1.53E-07
Regulators with a DNA recognition wing	RFX3	path:05310_3	1.53E-07
Regulators with a DNA recognition wing	RFX3	path:05320_1	1.53E-07
Regulators with a DNA recognition wing	RFX3	path:05320_3	1.53E-07
Regulators with a DNA recognition wing	RFX3	path:05320_4	1.53E-07
Regulators with a DNA recognition wing	RFX3	path:05320_6	1.53E-07
Regulators with a DNA recognition wing	RFX3	path:05322_5	1.53E-07
Regulators with a DNA recognition wing	RFX3	path:05322_6	1.53E-07
Regulators with a DNA recognition wing	RFX3	path:05330_1	1.53E-07
Regulators with a DNA recognition wing	RFX3	path:05330_2	1.53E-07
Regulators with a DNA recognition wing	RFX3	path:05330_4	1.53E-07
Regulators with a DNA recognition wing	RFX3	path:05330_7	4.00E-07

Regulators with a DNA recognition wing	RFX3	path:05332_1	1.53E-07
Regulators with a DNA recognition wing	RFX3	path:05416_4	1.53E-07
Rel/ankyrin	REL	path:04060_8	0.004162941
Rel/ankyrin	REL	path:04612_7	0.005200652
Rel/ankyrin	REL	path:04650_22	0.00019873
Rel/ankyrin	REL	path:04650_23	0.00019873
Rel/ankyrin	RELA	path:04060_62	0.000137451
Rel/ankyrin	RELA	path:04062_1	2.04E-07
Rel/ankyrin	RELA	path:04062_2	3.87E-08
Rel/ankyrin	RELA	path:04110_17	0.001869278
Rel/ankyrin	RELA	path:04110_19	0.001781655
Rel/ankyrin	RELA	path:04110_7	0.001869278
Rel/ankyrin	RELA	path:04115_1	0.0005539
Rel/ankyrin	RELA	path:04620_1	2.11E-06
Rel/ankyrin	RELA	path:04620_12	4.80E-09
Rel/ankyrin	RELA	path:04620_13	1.51E-07
Rel/ankyrin	RELA	path:04620_14	2.07E-07
Rel/ankyrin	RELA	path:04620_16	1.03E-08
Rel/ankyrin	RELA	path:04620_17	6.51E-08
Rel/ankyrin	RELA	path:04620_18	8.85E-09
Rel/ankyrin	RELA	path:04620_22	6.51E-08
Rel/ankyrin	RELA	path:04620_9	7.78E-08
Rel/ankyrin	RELA	path:04650_22	0.00197363
Rel/ankyrin	RELA	path:04650_23	0.001781655
Responders to external signals	SRF	path:05020_8	0.004545882
Responders to external signals	SRF	path:05020_9	0.00379009
Responders to external signals	SRF	path:05410_2	0.003033557
Responders to external signals	SRF	path:05414_3	0.003033557
Responders to external signals	SRF	path:05416_1	0.003033557
Runt	RUNX3	path:04612_4	0.000198099
SMAD	SMAD2	path:04110_18	6.60E-05
SMAD	SMAD2	path:04110_25	5.26E-05
SMAD	SMAD2	path:04510_1	5.26E-05
SMAD	SMAD2	path:04510_10	0.002748036

SMAD	SMAD2	path:04510_19	5.26E-05
SMAD	SMAD2	path:04510_6	0.002748036
SMAD	SMAD2	path:04510_8	0.002748036
SMAD	SMAD2	path:04512_11	0.005671238
SMAD	SMAD2	path:04512_12	0.000121975
SMAD	SMAD2	path:04512_13	5.26E-05
SMAD	SMAD2	path:04512_14	0.005849438
SMAD	SMAD2	path:04512_6	0.00016587
SMAD	SMAD2	path:05146_9	0.003683338
SMAD	SMAD3	path:04110_18	0.001049896
SMAD	SMAD3	path:04110_25	0.000921676
SMAD	SMAD3	path:04510_1	0.00105392
SMAD	SMAD3	path:04510_19	0.001170463
SMAD	SMAD3	path:04512_12	0.001326293
SMAD	SMAD3	path:04512_13	0.000921676
SMAD	SMAD3	path:04512_6	0.001837367
SMAD	SMAD3	path:05200_29	0.000921676
SMAD	SMAD3	path:05210_10	0.007105406
SMAD	SMAD4	path:04110_18	0.000743893
SMAD	SMAD4	path:04110_25	0.000620016
SMAD	SMAD4	path:04115_1	0.003366083
SMAD	SMAD4	path:04115_2	0.003046128
SMAD	SMAD4	path:04115_3	0.003046128
SMAD	SMAD4	path:04115_4	0.003046128
SMAD	SMAD4	path:04115_7	0.003046128
SMAD	SMAD4	path:05200_29	0.000734371
SMAD	SMAD4	path:05210_10	0.004709635
SMAD	SMAD4	path:05210_9	0.006805486
STAT	STAT1	path:04110_18	0.00514314
STAT	STAT1	path:04145_5	0.006088364
STAT	STAT1	path:04620_16	0.002113614
STAT	STAT1	path:04620_17	0.003819783
STAT	STAT1	path:04620_18	0.002113614
STAT	STAT1	path:04620_22	0.003819783

STAT	STAT1	path:04620_9	0.003819783
STAT	STAT1	path:04630_3	0.006088364
STAT	STAT1	path:04630_4	0.001501202
STAT	STAT1	path:05200_29	0.003819783
STAT	STAT1	path:05210_10	0.001312831
STAT	STAT3	path:04060_3	0.007358355
STAT	STAT3	path:04060_8	0.008398489
STAT	STAT3	path:04630_2	0.00794491
STAT	STAT3	path:04630_3	0.00794491
STAT	STAT3	path:04630_4	5.36E-05
STAT	STAT3	path:05142_4	0.000767514
STAT	STAT3	path:05200_29	0.000108961
STAT	STAT4	path:04630_4	0.000257265
STAT	STAT5A	path:04630_4	0.000370487
STAT	STAT5B	path:04110_20	0.009500448
STAT	STAT5B	path:04110_27	0.009500448
STAT	STAT5B	path:04630_4	0.000370487
STAT	STAT5B	path:05200_29	0.009500448
STAT	STAT5B	path:05200_47	0.002527186
STAT	STAT5B	path:05200_51	0.002527186
STAT	STAT5B	path:05200_52	0.005012521
STAT	STAT5B	path:05214_13	0.002527186
STAT	STAT5B	path:05214_14	0.002527186
STAT	STAT5B	path:05214_15	0.002527186
STAT	STAT5B	path:05218_5	0.009500448
STAT	STAT5B	path:05218_7	0.002527186
STAT	STAT5B	path:05218_8	0.009060668
STAT	STAT5B	path:05219_4	0.002527186
STAT	STAT5B	path:05220_11	0.002527186
STAT	STAT5B	path:05220_12	0.002527186
STAT	STAT5B	path:05220_9	0.002527186
STAT	STAT6	path:00590_4	0.003994719
STAT	STAT6	path:04630_2	0.000657478
STAT	STAT6	path:04630_3	0.000657478

STAT	STAT6	path:04630_4	0.000657478
Steroid hormone receptors (NR3)	AR	path:04110_18	0.002649237
Steroid hormone receptors (NR3)	AR	path:04110_23	0.003572289
Steroid hormone receptors (NR3)	AR	path:04115_1	0.000666989
Steroid hormone receptors (NR3)	AR	path:04115_2	0.005794552
Steroid hormone receptors (NR3)	AR	path:04115_3	0.000666989
Steroid hormone receptors (NR3)	AR	path:04115_4	0.005794552
Steroid hormone receptors (NR3)	AR	path:04115_7	0.005794552
Steroid hormone receptors (NR3)	AR	path:05200_16	0.005794552
Steroid hormone receptors (NR3)	AR	path:05200_47	0.005794552
Steroid hormone receptors (NR3)	AR	path:05200_51	0.006067474
Steroid hormone receptors (NR3)	AR	path:05214_13	0.005794552
Steroid hormone receptors (NR3)	AR	path:05214_14	0.005794552
Steroid hormone receptors (NR3)	AR	path:05214_15	0.006236059
Steroid hormone receptors (NR3)	AR	path:05215_1	0.006236059
Steroid hormone receptors (NR3)	AR	path:05216_4	0.006067474
Steroid hormone receptors (NR3)	AR	path:05218_7	0.006236059
Steroid hormone receptors (NR3)	AR	path:05219_4	0.006067474
Steroid hormone receptors (NR3)	AR	path:05220_11	0.006236059
Steroid hormone receptors (NR3)	AR	path:05220_12	0.005794552
Steroid hormone receptors (NR3)	AR	path:05220_9	0.005794552
Steroid hormone receptors (NR3)	ESR1	path:00830_2	0.005413618
Steroid hormone receptors (NR3)	ESR1	path:04115_1	0.000345486
Steroid hormone receptors (NR3)	ESR1	path:04115_2	0.002979325
Steroid hormone receptors (NR3)	ESR1	path:04115_3	0.002948202
Steroid hormone receptors (NR3)	ESR1	path:04115_4	0.002948202
Steroid hormone receptors (NR3)	ESR1	path:04115_7	0.002948202
Steroid hormone receptors (NR3)	ESR1	path:04610_8	0.002948202
Steroid hormone receptors (NR3)	ESR1	path:05200_29	0.000345486
Steroid hormone receptors (NR3)	HNF4A	path:00120_15	0.00730316
Steroid hormone receptors (NR3)	HNF4A	path:00120_19	0.003812707
Steroid hormone receptors (NR3)	HNF4A	path:00120_6	0.001698537
Steroid hormone receptors (NR3)	HNF4A	path:00120_7	0.003812707
Steroid hormone receptors (NR3)	HNF4A	path:00120_9	0.000100826

Steroid hormone receptors (NR3)	HNF4A	path:00590_1	0.001808381
Steroid hormone receptors (NR3)	HNF4A	path:00590_2	0.001722691
Steroid hormone receptors (NR3)	HNF4A	path:00590_3	0.001722691
Steroid hormone receptors (NR3)	HNF4A	path:00590_4	0.001898001
Steroid hormone receptors (NR3)	HNF4A	path:00591_1	0.000515682
Steroid hormone receptors (NR3)	HNF4A	path:00830_2	2.36E-05
Steroid hormone receptors (NR3)	HNF4A	path:00980_2	0.000733563
Steroid hormone receptors (NR3)	HNF4A	path:00982_10	2.52E-07
Steroid hormone receptors (NR3)	HNF4A	path:00982_4	5.38E-08
Steroid hormone receptors (NR3)	HNF4A	path:00982_5	5.38E-08
Steroid hormone receptors (NR3)	HNF4A	path:00982_9	5.38E-08
Steroid hormone receptors (NR3)	HNF4A	path:03320_1	0.001131883
Steroid hormone receptors (NR3)	HNF4A	path:03320_2	0.000100826
Steroid hormone receptors (NR3)	HNF4A	path:04610_2	4.35E-06
Steroid hormone receptors (NR3)	HNF4A	path:04610_3	4.35E-06
Steroid hormone receptors (NR3)	HNF4A	path:04610_4	0.000100826
Steroid hormone receptors (NR3)	HNF4A	path:04610_5	6.69E-06
Steroid hormone receptors (NR3)	HNF4A	path:04610_6	0.000179282
Steroid hormone receptors (NR3)	HNF4A	path:04610_8	0.001698537
Steroid hormone receptors (NR3)	HNF4A	path:04950_4	0.000287791
Steroid hormone receptors (NR3)	NR1I2	path:00830_2	0.004391058
Steroid hormone receptors (NR3)	NR1I2	path:00982_4	0.00011908
Steroid hormone receptors (NR3)	NR1I2	path:00982_5	0.00011908
Steroid hormone receptors (NR3)	NR1I2	path:00982_9	0.00011908
Steroid hormone receptors (NR3)	NR1I3	path:00830_2	0.000186607
Steroid hormone receptors (NR3)	NR1I3	path:00980_1	0.006275482
Steroid hormone receptors (NR3)	NR1I3	path:00982_4	0.000535628
Steroid hormone receptors (NR3)	NR1I3	path:00982_5	0.000535628
Steroid hormone receptors (NR3)	NR1I3	path:00982_9	0.000535628
Steroid hormone receptors (NR3)	NR2F1	path:03320_2	0.000132961
Steroid hormone receptors (NR3)	NR4A1	path:00140_10	0.00077713
Steroid hormone receptors (NR3)	NR4A1	path:00140_13	6.42E-06
Steroid hormone receptors (NR3)	NR4A1	path:00140_14	0.00077713
Steroid hormone receptors (NR3)	NR4A1	path:00140_16	0.00077713

Steroid hormone receptors (NR3)	NR4A1	path:00140_18	0.00077713
Steroid hormone receptors (NR3)	NR4A1	path:00140_19	0.000832486
Steroid hormone receptors (NR3)	NR4A1	path:00140_20	0.00077713
Steroid hormone receptors (NR3)	NR4A1	path:00140_3	0.00077713
Steroid hormone receptors (NR3)	NR4A1	path:00140_4	0.00077713
Steroid hormone receptors (NR3)	NR4A1	path:00140_5	0.000960385
Steroid hormone receptors (NR3)	NR4A1	path:00140_6	0.001089761
Steroid hormone receptors (NR3)	NR4A1	path:00140_7	0.000832486
Steroid hormone receptors (NR3)	NR4A1	path:00140_8	0.00077713
Steroid hormone receptors (NR3)	NR4A1	path:00140_9	0.00077713
Steroid hormone receptors (NR3)	NR5A1	path:00140_10	1.53E-06
Steroid hormone receptors (NR3)	NR5A1	path:00140_11	1.69E-05
Steroid hormone receptors (NR3)	NR5A1	path:00140_12	0.000116509
Steroid hormone receptors (NR3)	NR5A1	path:00140_13	6.63E-09
Steroid hormone receptors (NR3)	NR5A1	path:00140_14	0.000393628
Steroid hormone receptors (NR3)	NR5A1	path:00140_16	1.15E-06
Steroid hormone receptors (NR3)	NR5A1	path:00140_18	1.15E-06
Steroid hormone receptors (NR3)	NR5A1	path:00140_19	1.72E-06
Steroid hormone receptors (NR3)	NR5A1	path:00140_20	0.00062425
Steroid hormone receptors (NR3)	NR5A1	path:00140_22	5.77E-05
Steroid hormone receptors (NR3)	NR5A1	path:00140_23	5.77E-05
Steroid hormone receptors (NR3)	NR5A1	path:00140_24	5.77E-05
Steroid hormone receptors (NR3)	NR5A1	path:00140_3	0.00062425
Steroid hormone receptors (NR3)	NR5A1	path:00140_4	0.00062425
Steroid hormone receptors (NR3)	NR5A1	path:00140_5	2.18E-06
Steroid hormone receptors (NR3)	NR5A1	path:00140_6	2.70E-06
Steroid hormone receptors (NR3)	NR5A1	path:00140_7	1.72E-06
Steroid hormone receptors (NR3)	NR5A1	path:00140_8	1.09E-06
Steroid hormone receptors (NR3)	NR5A1	path:00140_9	1.09E-06
Steroid hormone receptors (NR3)	NR5A1	path:04060_21	8.93E-05
Steroid hormone receptors (NR3)	NR5A1	path:04350_13	0.002225649
Steroid hormone receptors (NR3)	NR5A2	path:00140_11	0.000442761
Steroid hormone receptors (NR3)	NR5A2	path:00140_12	0.002212517
Steroid hormone receptors (NR3)	NR5A2	path:00140_18	0.003089823

Steroid hormone receptors (NR3)	NR5A2	path:00140_3	0.003089823
Steroid hormone receptors (NR3)	NR5A2	path:00140_4	0.003089823
Steroid hormone receptors (NR3)	NR5A2	path:00140_5	0.00385968
Steroid hormone receptors (NR3)	NR5A2	path:00140_6	0.004244219
Steroid hormone receptors (NR3)	NR5A2	path:00140_7	0.003474881
Steroid hormone receptors (NR3)	NR5A2	path:00140_8	0.002898659
Steroid hormone receptors (NR3)	NR5A2	path:00140_9	0.002898659
Steroid hormone receptors (NR3)	THRB	path:04080_2	0.000625072
Steroid hormone receptors (NR3)	VDR	path:00140_1	0.009786942
Steroid hormone receptors (NR3)	VDR	path:00140_11	0.008771608
Steroid hormone receptors (NR3)	VDR	path:00140_25	0.009786942
Steroid hormone receptors (NR3)	VDR	path:00140_26	0.009786942
Steroid hormone receptors (NR3)	VDR	path:00591_1	0.008771608
Steroid hormone receptors (NR3)	VDR	path:00980_2	0.00973729
Steroid hormone receptors (NR3)	VDR	path:00982_10	0.004788204
Steroid hormone receptors (NR3)	VDR	path:00982_11	0.001635842
Steroid hormone receptors (NR3)	VDR	path:00982_2	0.003675018
Steroid hormone receptors (NR3)	VDR	path:00982_3	0.001635842
Steroid hormone receptors (NR3)	VDR	path:00982_4	0.003361044
Steroid hormone receptors (NR3)	VDR	path:00982_5	0.003361044
Steroid hormone receptors (NR3)	VDR	path:00982_6	0.008771608
Steroid hormone receptors (NR3)	VDR	path:00982_7	0.001635842
Steroid hormone receptors (NR3)	VDR	path:00982_8	0.001635842
Steroid hormone receptors (NR3)	VDR	path:00982_9	0.003361044
Steroid hormone receptors (NR3)	VDR	path:04012_1	0.008771608
Steroid hormone receptors (NR3)	VDR	path:04012_11	0.008771608
Steroid hormone receptors (NR3)	VDR	path:04012_12	0.008771608
Steroid hormone receptors (NR3)	VDR	path:04012_14	0.0090937
Steroid hormone receptors (NR3)	VDR	path:04012_4	0.008771608
Steroid hormone receptors (NR3)	VDR	path:04012_7	0.008771608
Steroid hormone receptors (NR3)	VDR	path:05200_9	0.008771608
Steroid hormone receptors (NR3)	VDR	path:05214_1	0.008771608
Steroid hormone receptors (NR3)	VDR	path:05214_10	0.009415594
Steroid hormone receptors (NR3)	VDR	path:05214_17	0.008771608

Steroid hormone receptors (NR3)	VDR	path:05214_2	0.008771608
Steroid hormone receptors (NR3)	VDR	path:05214_3	0.008771608
Steroid hormone receptors (NR3)	VDR	path:05214_4	0.008771608
Steroid hormone receptors (NR3)	VDR	path:05214_5	0.008771608
Steroid hormone receptors (NR3)	VDR	path:05214_6	0.008771608
Steroid hormone receptors (NR3)	VDR	path:05214_8	0.008771608
Steroid hormone receptors (NR3)	VDR	path:05215_11	0.009786942
Steroid hormone receptors (NR3)	VDR	path:05218_2	0.008771608
Steroid hormone receptors (NR3)	VDR	path:05223_6	0.008771608
TCF-1	LEF1	path:04310_6	0.000671935
TCF-1	LEF1	path:04310_7	0.000118706
TCF-1	LEF1	path:04916_11	0.00555092
TCF-1	LEF1	path:04916_3	0.002645905
TCF-1	LEF1	path:04916_8	0.007825657
TCF-1	LEF1	path:05200_31	1.30E-05
TCF-1	LEF1	path:05200_42	6.00E-05
TCF-1	LEF1	path:05200_50	0.003595279
TCF-1	LEF1	path:05210_10	0.002645905
TCF-1	LEF1	path:05210_12	1.30E-05
TCF-1	LEF1	path:05210_7	1.30E-05
TCF-1	LEF1	path:05213_1	2.72E-05
TCF-1	LEF1	path:05215_4	0.003117148
TCF-1	LEF1	path:05216_4	1.30E-05
TCF-1	LEF1	path:05221_1	5.77E-05
TCF-1	LEF1	path:05222_1	0.005057313
TCF-1	LEF1	path:05222_2	0.004566365
TCF-1	LEF1	path:05222_7	0.004078733
TCF-1	TCF7	path:04660_20	0.00669167
Tissue-specific regulators	FOXA2	path:04610_3	0.000581017
Tissue-specific regulators	FOXA2	path:04610_4	0.000581017
Tissue-specific regulators	FOXA2	path:04610_6	0.000581017
Tissue-specific regulators	FOXA3	path:00140_1	0.000327469
Tissue-specific regulators	FOXA3	path:00140_11	0.000188543
Tissue-specific regulators	FOXA3	path:00140_12	0.000502586

Tissue-specific regulators	FOXA3	path:00140_25	0.000327469
Tissue-specific regulators	FOXA3	path:00140_26	0.000327469
Tissue-specific regulators	FOXA3	path:00591_1	0.000312584
Tissue-specific regulators	FOXA3	path:00830_2	0.000975791
Tissue-specific regulators	FOXA3	path:00980_2	0.000327469
Tissue-specific regulators	FOXA3	path:00982_10	7.28E-05
Tissue-specific regulators	FOXA3	path:00982_11	2.48E-05
Tissue-specific regulators	FOXA3	path:00982_2	5.58E-05
Tissue-specific regulators	FOXA3	path:00982_3	2.48E-05
Tissue-specific regulators	FOXA3	path:00982_4	5.10E-05
Tissue-specific regulators	FOXA3	path:00982_5	5.10E-05
Tissue-specific regulators	FOXA3	path:00982_6	0.000248984
Tissue-specific regulators	FOXA3	path:00982_7	2.48E-05
Tissue-specific regulators	FOXA3	path:00982_8	2.48E-05
Tissue-specific regulators	FOXA3	path:00982_9	5.10E-05
Ubiquitous bHLH-ZIP factors	MITF	path:00350_1	0.00045151
Ubiquitous bHLH-ZIP factors	MITF	path:04916_3	1.75E-08
Ubiquitous bHLH-ZIP factors	SREBF1	path:00100_9	0.009882974
Ubiquitous bHLH-ZIP factors	SREBF1	path:03320_2	0.009882974
Ubiquitous bHLH-ZIP factors	SREBF1	path:04910_5	3.27E-05
Ubiquitous bHLH-ZIP factors	SREBF1	path:04910_6	0.00143191
Ubiquitous factors	SP1	path:00120_1	0.006087664
Ubiquitous factors	SP1	path:00120_2	0.003885876
Ubiquitous factors	SP1	path:00120_3	0.003885876
Ubiquitous factors	SP1	path:00140_1	0.001893526
Ubiquitous factors	SP1	path:00140_10	1.44E-05
Ubiquitous factors	SP1	path:00140_11	0.001534931
Ubiquitous factors	SP1	path:00140_12	0.006087664
Ubiquitous factors	SP1	path:00140_13	2.77E-05
Ubiquitous factors	SP1	path:00140_14	0.003885876
Ubiquitous factors	SP1	path:00140_16	0.000245716
Ubiquitous factors	SP1	path:00140_18	0.000245716
Ubiquitous factors	SP1	path:00140_19	2.77E-05
Ubiquitous factors	SP1	path:00140_20	0.000441307

Ubiquitous factors	SP1	path:00140_25	0.001893526
Ubiquitous factors	SP1	path:00140_3	1.44E-05
Ubiquitous factors	SP1	path:00140_4	1.44E-05
Ubiquitous factors	SP1	path:00140_5	5.07E-05
Ubiquitous factors	SP1	path:00140_6	8.66E-05
Ubiquitous factors	SP1	path:00140_7	2.77E-05
Ubiquitous factors	SP1	path:00140_8	0.000123921
Ubiquitous factors	SP1	path:00140_9	0.000123921
Ubiquitous factors	SP1	path:00240_16	0.005536058
Ubiquitous factors	SP1	path:00240_17	0.003391309
Ubiquitous factors	SP1	path:00240_2	0.001690362
Ubiquitous factors	SP1	path:00240_5	0.009002988
Ubiquitous factors	SP1	path:00330_1	0.005536058
Ubiquitous factors	SP1	path:00330_9	0.005126386
Ubiquitous factors	SP1	path:00350_2	0.006192277
Ubiquitous factors	SP1	path:00350_3	0.006192277
Ubiquitous factors	SP1	path:00980_2	0.001393247
Ubiquitous factors	SP1	path:00982_10	0.002175912
Ubiquitous factors	SP1	path:04010_10	0.003931316
Ubiquitous factors	SP1	path:04010_26	0.000441307
Ubiquitous factors	SP1	path:04010_5	0.000123921
Ubiquitous factors	SP1	path:04060_22	0.002149604
Ubiquitous factors	SP1	path:04060_32	0.001014956
Ubiquitous factors	SP1	path:04060_47	0.007433507
Ubiquitous factors	SP1	path:04110_1	0.00916136
Ubiquitous factors	SP1	path:04110_17	1.86E-10
Ubiquitous factors	SP1	path:04110_18	4.28E-11
Ubiquitous factors	SP1	path:04110_19	1.05E-10
Ubiquitous factors	SP1	path:04110_20	2.92E-09
Ubiquitous factors	SP1	path:04110_22	1.43E-05
Ubiquitous factors	SP1	path:04110_23	5.22E-09
Ubiquitous factors	SP1	path:04110_25	0.006192277
Ubiquitous factors	SP1	path:04110_26	7.70E-08
Ubiquitous factors	SP1	path:04110_27	2.92E-09

Ubiquitous factors	SP1	path:04110_3	6.46E-09
Ubiquitous factors	SP1	path:04110_4	6.46E-09
Ubiquitous factors	SP1	path:04110_7	4.97E-07
Ubiquitous factors	SP1	path:04115_1	3.47E-10
Ubiquitous factors	SP1	path:04115_2	6.74E-05
Ubiquitous factors	SP1	path:04115_3	4.23E-05
Ubiquitous factors	SP1	path:04115_4	6.18E-06
Ubiquitous factors	SP1	path:04115_7	4.96E-05
Ubiquitous factors	SP1	path:04144_2	7.61E-06
Ubiquitous factors	SP1	path:04210_20	0.002175912
Ubiquitous factors	SP1	path:04210_24	0.001107767
Ubiquitous factors	SP1	path:04310_6	1.33E-05
Ubiquitous factors	SP1	path:04310_7	1.77E-05
Ubiquitous factors	SP1	path:04510_1	7.85E-13
Ubiquitous factors	SP1	path:04510_10	1.05E-10
Ubiquitous factors	SP1	path:04510_13	0.001603207
Ubiquitous factors	SP1	path:04510_15	0.001164758
Ubiquitous factors	SP1	path:04510_16	0.002771235
Ubiquitous factors	SP1	path:04510_17	0.000141426
Ubiquitous factors	SP1	path:04510_18	8.07E-08
Ubiquitous factors	SP1	path:04510_19	1.96E-12
Ubiquitous factors	SP1	path:04510_20	1.43E-05
Ubiquitous factors	SP1	path:04510_23	0.000718519
Ubiquitous factors	SP1	path:04510_24	0.002175912
Ubiquitous factors	SP1	path:04510_25	0.0024986
Ubiquitous factors	SP1	path:04510_4	6.74E-05
Ubiquitous factors	SP1	path:04510_5	0.0024986
Ubiquitous factors	SP1	path:04510_6	1.07E-10
Ubiquitous factors	SP1	path:04510_7	0.000448433
Ubiquitous factors	SP1	path:04510_8	6.16E-10
Ubiquitous factors	SP1	path:04512_10	2.01E-05
Ubiquitous factors	SP1	path:04512_11	5.55E-06
Ubiquitous factors	SP1	path:04512_12	1.66E-06
Ubiquitous factors	SP1	path:04512_13	7.86E-06

Ubiquitous factors	SP1	path:04512_14	0.000101569
Ubiquitous factors	SP1	path:04512_18	0.000245716
Ubiquitous factors	SP1	path:04512_19	0.000549822
Ubiquitous factors	SP1	path:04512_20	0.002149604
Ubiquitous factors	SP1	path:04512_22	0.008608739
Ubiquitous factors	SP1	path:04512_27	0.00016592
Ubiquitous factors	SP1	path:04512_3	1.66E-05
Ubiquitous factors	SP1	path:04512_5	2.72E-07
Ubiquitous factors	SP1	path:04512_6	6.24E-07
Ubiquitous factors	SP1	path:04512_9	9.36E-06
Ubiquitous factors	SP1	path:04520_3	0.005126386
Ubiquitous factors	SP1	path:04520_5	0.001603207
Ubiquitous factors	SP1	path:04520_7	0.000412181
Ubiquitous factors	SP1	path:04540_13	1.22E-05
Ubiquitous factors	SP1	path:04610_3	9.21E-07
Ubiquitous factors	SP1	path:04610_4	5.55E-06
Ubiquitous factors	SP1	path:04610_6	9.21E-07
Ubiquitous factors	SP1	path:04610_7	0.000133832
Ubiquitous factors	SP1	path:04610_8	0.000203885
Ubiquitous factors	SP1	path:04620_18	0.000203885
Ubiquitous factors	SP1	path:04620_9	0.001893526
Ubiquitous factors	SP1	path:04630_1	0.000231547
Ubiquitous factors	SP1	path:04630_2	0.001390056
Ubiquitous factors	SP1	path:04630_3	1.97E-06
Ubiquitous factors	SP1	path:04630_4	5.95E-06
Ubiquitous factors	SP1	path:04650_20	4.84E-05
Ubiquitous factors	SP1	path:04650_7	0.008608739
Ubiquitous factors	SP1	path:04722_19	0.000718519
Ubiquitous factors	SP1	path:04810_29	7.92E-07
Ubiquitous factors	SP1	path:04912_8	1.33E-05
Ubiquitous factors	SP1	path:04940_4	0.007433507
Ubiquitous factors	SP1	path:05014_3	0.006087664
Ubiquitous factors	SP1	path:05014_4	0.008608739
Ubiquitous factors	SP1	path:05016_8	0.007433507

Ubiquitous factors	SP1	path:05100_3	0.001014956
Ubiquitous factors	SP1	path:05140_10	0.008608739
Ubiquitous factors	SP1	path:05140_6	0.000245716
Ubiquitous factors	SP1	path:05142_10	0.001107767
Ubiquitous factors	SP1	path:05142_11	0.003931316
Ubiquitous factors	SP1	path:05142_17	0.003885876
Ubiquitous factors	SP1	path:05142_18	0.003885876
Ubiquitous factors	SP1	path:05142_20	0.000441307
Ubiquitous factors	SP1	path:05142_21	0.000718519
Ubiquitous factors	SP1	path:05142_4	0.001107767
Ubiquitous factors	SP1	path:05145_19	0.006087664
Ubiquitous factors	SP1	path:05145_21	0.001014956
Ubiquitous factors	SP1	path:05145_8	0.006087664
Ubiquitous factors	SP1	path:05146_1	1.23E-06
Ubiquitous factors	SP1	path:05146_2	7.92E-07
Ubiquitous factors	SP1	path:05146_5	0.002149604
Ubiquitous factors	SP1	path:05146_9	9.10E-07
Ubiquitous factors	SP1	path:05200_19	0.003935354
Ubiquitous factors	SP1	path:05200_20	0.003935354
Ubiquitous factors	SP1	path:05200_24	0.00927462
Ubiquitous factors	SP1	path:05200_29	9.21E-07
Ubiquitous factors	SP1	path:05200_3	2.01E-05
Ubiquitous factors	SP1	path:05200_31	0.000718519
Ubiquitous factors	SP1	path:05200_38	0.008608739
Ubiquitous factors	SP1	path:05200_4	0.000289957
Ubiquitous factors	SP1	path:05200_42	0.000718519
Ubiquitous factors	SP1	path:05200_47	2.07E-08
Ubiquitous factors	SP1	path:05200_48	1.33E-05
Ubiquitous factors	SP1	path:05200_50	5.07E-05
Ubiquitous factors	SP1	path:05200_51	5.54E-09
Ubiquitous factors	SP1	path:05200_52	1.55E-08
Ubiquitous factors	SP1	path:05200_53	4.11E-06
Ubiquitous factors	SP1	path:05200_56	0.001014956
Ubiquitous factors	SP1	path:05210_10	0.000441307

Ubiquitous factors	SP1	path:05210_12	0.000718519
Ubiquitous factors	SP1	path:05210_7	0.000718519
Ubiquitous factors	SP1	path:05212_13	1.44E-05
Ubiquitous factors	SP1	path:05212_14	0.008608739
Ubiquitous factors	SP1	path:05214_12	1.33E-05
Ubiquitous factors	SP1	path:05214_13	1.18E-07
Ubiquitous factors	SP1	path:05214_14	2.07E-08
Ubiquitous factors	SP1	path:05214_15	9.21E-07
Ubiquitous factors	SP1	path:05214_2	0.005536058
Ubiquitous factors	SP1	path:05214_21	0.000245716
Ubiquitous factors	SP1	path:05214_5	0.005536058
Ubiquitous factors	SP1	path:05215_10	0.001164758
Ubiquitous factors	SP1	path:05215_7	0.008608739
Ubiquitous factors	SP1	path:05216_4	0.000441307
Ubiquitous factors	SP1	path:05218_4	0.000411461
Ubiquitous factors	SP1	path:05218_5	0.000133832
Ubiquitous factors	SP1	path:05218_6	1.33E-05
Ubiquitous factors	SP1	path:05218_7	9.21E-07
Ubiquitous factors	SP1	path:05218_8	7.27E-05
Ubiquitous factors	SP1	path:05219_3	0.000310459
Ubiquitous factors	SP1	path:05219_4	1.44E-05
Ubiquitous factors	SP1	path:05220_11	9.21E-07
Ubiquitous factors	SP1	path:05220_12	1.18E-07
Ubiquitous factors	SP1	path:05220_5	0.002149604
Ubiquitous factors	SP1	path:05220_8	1.33E-05
Ubiquitous factors	SP1	path:05220_9	2.07E-08
Ubiquitous factors	SP1	path:05221_1	0.006192277
Ubiquitous factors	SP1	path:05222_1	5.36E-07
Ubiquitous factors	SP1	path:05222_2	0.000133832
Ubiquitous factors	SP1	path:05222_4	2.01E-05
Ubiquitous factors	SP1	path:05222_7	0.001603207
Ubiquitous factors	SP1	path:05223_3	1.44E-05
Ubiquitous factors	SP1	path:05320_9	0.007433507
Ubiquitous factors	SP1	path:05330_5	0.007433507

Ubiquitous factors	SP1	path:05332_2	0.007433507
Ubiquitous factors	SP1	path:05332_5	0.007433507
Ubiquitous factors	SP1	path:05414_2	1.77E-05
Ubiquitous factors	SP3	path:00120_1	0.000926143
Ubiquitous factors	SP3	path:00120_2	0.000618445
Ubiquitous factors	SP3	path:00120_3	0.000618445
Ubiquitous factors	SP3	path:00140_1	0.006217062
Ubiquitous factors	SP3	path:00140_10	4.16E-05
Ubiquitous factors	SP3	path:00140_11	5.63E-05
Ubiquitous factors	SP3	path:00140_12	0.000126279
Ubiquitous factors	SP3	path:00140_13	5.63E-05
Ubiquitous factors	SP3	path:00140_16	0.000926143
Ubiquitous factors	SP3	path:00140_18	0.000926143
Ubiquitous factors	SP3	path:00140_19	5.63E-05
Ubiquitous factors	SP3	path:00140_20	0.001366718
Ubiquitous factors	SP3	path:00140_25	0.006217062
Ubiquitous factors	SP3	path:00140_26	0.005691454
Ubiquitous factors	SP3	path:00140_3	4.16E-05
Ubiquitous factors	SP3	path:00140_4	4.16E-05
Ubiquitous factors	SP3	path:00140_5	8.41E-05
Ubiquitous factors	SP3	path:00140_6	0.000126279
Ubiquitous factors	SP3	path:00140_7	5.63E-05
Ubiquitous factors	SP3	path:00140_8	0.000618445
Ubiquitous factors	SP3	path:00140_9	0.000618445
Ubiquitous factors	SP3	path:00982_10	0.004572948
Ubiquitous factors	SP3	path:04060_43	0.002641876
Ubiquitous factors	SP3	path:04110_17	3.14E-07
Ubiquitous factors	SP3	path:04110_18	0.000128011
Ubiquitous factors	SP3	path:04110_19	2.26E-07
Ubiquitous factors	SP3	path:04110_20	4.71E-06
Ubiquitous factors	SP3	path:04110_22	0.001275861
Ubiquitous factors	SP3	path:04110_23	3.02E-05
Ubiquitous factors	SP3	path:04110_26	0.000307175
Ubiquitous factors	SP3	path:04110_27	4.71E-06

Ubiquitous factors	SP3	path:04110_3	3.13E-05
Ubiquitous factors	SP3	path:04110_4	3.13E-05
Ubiquitous factors	SP3	path:04110_7	0.007662288
Ubiquitous factors	SP3	path:04115_1	0.00406173
Ubiquitous factors	SP3	path:04115_4	0.002218693
Ubiquitous factors	SP3	path:04144_2	0.000814667
Ubiquitous factors	SP3	path:04510_1	3.35E-07
Ubiquitous factors	SP3	path:04510_10	1.24E-07
Ubiquitous factors	SP3	path:04510_17	0.001366718
Ubiquitous factors	SP3	path:04510_18	6.32E-05
Ubiquitous factors	SP3	path:04510_19	6.37E-07
Ubiquitous factors	SP3	path:04510_20	0.004572948
Ubiquitous factors	SP3	path:04510_6	1.24E-07
Ubiquitous factors	SP3	path:04510_7	0.002877506
Ubiquitous factors	SP3	path:04510_8	1.24E-07
Ubiquitous factors	SP3	path:04512_10	0.000488202
Ubiquitous factors	SP3	path:04512_11	0.000758402
Ubiquitous factors	SP3	path:04512_12	3.50E-05
Ubiquitous factors	SP3	path:04512_13	5.52E-05
Ubiquitous factors	SP3	path:04512_14	0.000906884
Ubiquitous factors	SP3	path:04512_18	0.000926143
Ubiquitous factors	SP3	path:04512_19	0.000541628
Ubiquitous factors	SP3	path:04512_27	0.000423815
Ubiquitous factors	SP3	path:04512_3	3.50E-05
Ubiquitous factors	SP3	path:04512_5	2.26E-07
Ubiquitous factors	SP3	path:04512_6	5.63E-05
Ubiquitous factors	SP3	path:04512_9	7.41E-05
Ubiquitous factors	SP3	path:04610_7	0.004572948
Ubiquitous factors	SP3	path:04610_8	0.005691454
Ubiquitous factors	SP3	path:04810_29	0.000814667
Ubiquitous factors	SP3	path:05144_3	0.002641876
Ubiquitous factors	SP3	path:05146_1	1.74E-06
Ubiquitous factors	SP3	path:05146_2	1.13E-06
Ubiquitous factors	SP3	path:05146_9	1.78E-05

Ubiquitous factors	SP3	path:05200_47	0.000618445
Ubiquitous factors	SP3	path:05200_48	0.00015602
Ubiquitous factors	SP3	path:05200_51	8.65E-07
Ubiquitous factors	SP3	path:05200_52	1.23E-05
Ubiquitous factors	SP3	path:05200_53	0.003634657
Ubiquitous factors	SP3	path:05212_13	0.001366718
Ubiquitous factors	SP3	path:05214_12	0.00015602
Ubiquitous factors	SP3	path:05214_13	3.02E-05
Ubiquitous factors	SP3	path:05214_14	0.000618445
Ubiquitous factors	SP3	path:05214_15	5.63E-05
Ubiquitous factors	SP3	path:05218_6	0.00015602
Ubiquitous factors	SP3	path:05218_7	5.63E-05
Ubiquitous factors	SP3	path:05218_8	0.000758402
Ubiquitous factors	SP3	path:05219_3	0.00677863
Ubiquitous factors	SP3	path:05219_4	4.16E-05
Ubiquitous factors	SP3	path:05220_11	5.63E-05
Ubiquitous factors	SP3	path:05220_12	3.02E-05
Ubiquitous factors	SP3	path:05220_8	0.00015602
Ubiquitous factors	SP3	path:05220_9	0.000618445
Ubiquitous factors	SP3	path:05222_1	0.005691454
Ubiquitous factors	SP3	path:05223_3	0.001366718
Ubiquitous factors	SP3	path:05414_2	0.00143924
Ubiquitous factors	YY1	path:00140_10	0.009417621
Ubiquitous factors	YY1	path:00140_13	0.009975361
Ubiquitous factors	YY1	path:00140_14	0.00758906
Ubiquitous factors	YY1	path:00140_16	0.009417621
Ubiquitous factors	YY1	path:00140_18	0.009417621
Ubiquitous factors	YY1	path:00140_19	0.009975361
Ubiquitous factors	YY1	path:00140_20	0.009417621
Ubiquitous factors	YY1	path:00140_3	0.009417621
Ubiquitous factors	YY1	path:00140_4	0.009417621
Ubiquitous factors	YY1	path:00140_7	0.009975361
Ubiquitous factors	YY1	path:00140_8	0.00758906
Ubiquitous factors	YY1	path:00140_9	0.00758906

Ubiquitous factors	YY1	path:04110_17	0.000485911
Ubiquitous factors	YY1	path:04110_19	0.000476218
Ubiquitous factors	YY1	path:04110_20	0.003725909
Ubiquitous factors	YY1	path:04110_23	0.005922984
Ubiquitous factors	YY1	path:04110_27	0.003725909
Ubiquitous factors	YY1	path:04110_7	0.00758906
Ubiquitous factors	YY1	path:04115_4	0.009975361
Ubiquitous factors	YY1	path:04650_20	0.007430866
Ubiquitous factors	YY1	path:04650_7	0.009417621
Ubiquitous factors	YY1	path:05200_47	0.00758906
Ubiquitous factors	YY1	path:05200_48	0.005151692
Ubiquitous factors	YY1	path:05200_51	0.00043761
Ubiquitous factors	YY1	path:05210_10	0.009417621
Ubiquitous factors	YY1	path:05212_13	0.009417621
Ubiquitous factors	YY1	path:05214_12	0.005151692
Ubiquitous factors	YY1	path:05214_13	0.000410896
Ubiquitous factors	YY1	path:05214_14	0.00758906
Ubiquitous factors	YY1	path:05214_15	0.009975361
Ubiquitous factors	YY1	path:05218_6	0.005151692
Ubiquitous factors	YY1	path:05218_7	0.009975361
Ubiquitous factors	YY1	path:05218_8	0.003139302
Ubiquitous factors	YY1	path:05219_3	0.003725909
Ubiquitous factors	YY1	path:05219_4	0.009417621
Ubiquitous factors	YY1	path:05220_11	0.009975361
Ubiquitous factors	YY1	path:05220_12	0.000410896
Ubiquitous factors	YY1	path:05220_8	0.005151692
Ubiquitous factors	YY1	path:05220_9	0.00758906
Ubiquitous factors	YY1	path:05223_3	0.009417621

Table S5. The detailed information of the co-tissue TFs regulated sub-pathways.

Tissues	TF	P_Value	Subpathway
BL	TFAP2A	0.005723968	path:04020_2
BL	TFAP2A	0.003857334	path:04080_7
BL	TFAP2A	0.00057548	path:04144_2
BL	TFAP2A	0.001269093	path:04510_10
BL	TFAP2A	0.005458314	path:04510_15
BL	TFAP2A	0.003332624	path:04510_16
BL	TFAP2A	3.81E-05	path:04510_17
BL	TFAP2A	1.42E-05	path:04510_18
BL	TFAP2A	0.007739985	path:04510_25
BL	TFAP2A	0.007739985	path:04510_5
BL	TFAP2A	0.001276164	path:04510_6
BL	TFAP2A	8.35E-05	path:04510_7
BL	TFAP2A	0.001271633	path:04510_8
BL	TFAP2A	0.002407854	path:04520_2
BL	TFAP2A	0.001262516	path:04520_3
BL	TFAP2A	0.00057548	path:04520_5
BL	TFAP2A	0.001262516	path:04520_7
BL	TFAP2A	0.009046641	path:04520_9
BL	TFAP2A	0.003857334	path:04912_8
BL	TFAP2A	2.20E-05	path:05200_10
BL	TFAP2A	1.91E-05	path:05200_11
BL	TFAP2A	1.91E-05	path:05200_12
BL	TFAP2A	6.62E-06	path:05200_15
BL	TFAP2A	0.000448647	path:05200_21
BL	TFAP2A	8.35E-05	path:05200_29
BL	TFAP2A	1.91E-05	path:05200_4
BL	TFAP2A	2.22E-05	path:05200_5
BL	TFAP2A	2.16E-06	path:05200_8
BL	TFAP2A	0.002699451	path:05212_11
BL	TFAP2A	0.003332624	path:05212_12
BL	TFAP2A	0.004058678	path:05214_1
BL	TFAP2A	0.000317116	path:05214_10

BL	TFAP2A	0.002407854	path:05214_11
BL	TFAP2A	0.007739985	path:05214_16
BL	TFAP2A	0.004058678	path:05214_17
BL	TFAP2A	0.004058678	path:05214_2
BL	TFAP2A	0.004058678	path:05214_3
BL	TFAP2A	0.004058678	path:05214_4
BL	TFAP2A	0.004058678	path:05214_5
BL	TFAP2A	0.004058678	path:05214_6
BL	TFAP2A	0.007739985	path:05214_7
BL	TFAP2A	0.004058678	path:05214_8
BL	TFAP2A	0.000317116	path:05215_10
BL	TFAP2A	0.000436788	path:05215_11
BL	TFAP2A	0.001124594	path:05215_8
BL	TFAP2A	0.002407854	path:05219_2
BL	TP53	0.007702858	path:04010_15
BL	TP53	0.009062653	path:04010_16
BL	TP53	0.007443002	path:04010_21
BL	TP53	0.002466645	path:04012_12
BL	TP53	2.55E-11	path:04110_1
BL	TP53	3.28E-07	path:04110_17
BL	TP53	1.48E-05	path:04110_19
BL	TP53	0.001943417	path:04110_20
BL	TP53	7.56E-05	path:04110_22
BL	TP53	0.000198814	path:04110_23
BL	TP53	0.001943417	path:04110_27
BL	TP53	0.000210553	path:04110_3
BL	TP53	0.004511216	path:04110_4
BL	TP53	4.42E-09	path:04110_7
BL	TP53	0.004291125	path:04110_8
BL	TP53	0	path:04115_1
BL	TP53	0	path:04115_2
BL	TP53	0	path:04115_3
BL	TP53	0	path:04115_4
BL	TP53	0	path:04115_7

BL	TP53	0.000421718	path:04210_20
BL	TP53	0.000275763	path:04210_23
BL	TP53	0.000275763	path:04210_24
BL	TP53	0.009131774	path:04510_10
BL	TP53	0.004511216	path:04510_15
BL	TP53	0.002466645	path:04510_16
BL	TP53	0.000580605	path:04510_17
BL	TP53	0.001774645	path:04510_18
BL	TP53	0.006951959	path:04510_25
BL	TP53	0.006951959	path:04510_5
BL	TP53	0.009576653	path:04510_6
BL	TP53	0.001110697	path:04510_7
BL	TP53	0.00935253	path:04510_8
BL	TP53	0.009062653	path:04621_7
BL	TP53	0.000218217	path:04722_19
BL	TP53	0.002756828	path:04912_8
BL	TP53	0.009062653	path:05014_4
BL	TP53	0.007459365	path:05160_12
BL	TP53	0.009062653	path:05160_15
BL	TP53	0.000338058	path:05200_10
BL	TP53	0.000275763	path:05200_11
BL	TP53	0.000275763	path:05200_12
BL	TP53	0.000968321	path:05200_15
BL	TP53	0.00392722	path:05200_17
BL	TP53	0.002997803	path:05200_19
BL	TP53	0.00011707	path:05200_20
BL	TP53	0.000275763	path:05200_4
BL	TP53	7.56E-05	path:05200_47
BL	TP53	1.95E-05	path:05200_48
BL	TP53	0.000367024	path:05200_5
BL	TP53	0.000172314	path:05200_51
BL	TP53	1.95E-05	path:05200_8
BL	TP53	0.007459365	path:05213_2
BL	TP53	0.003580594	path:05214_1

BL	TP53	0.000210553	path:05214_10
BL	TP53	0.001774645	path:05214_11
BL	TP53	1.95E-05	path:05214_12
BL	TP53	0.00011707	path:05214_13
BL	TP53	7.56E-05	path:05214_14
BL	TP53	0.003580594	path:05214_4
BL	TP53	0.004511216	path:05215_10
BL	TP53	0.000275763	path:05215_11
BL	TP53	0.00331496	path:05215_3
BL	TP53	0.002754132	path:05218_2
BL	TP53	0.001774645	path:05218_5
BL	TP53	1.95E-05	path:05218_6
BL	TP53	0.001385048	path:05218_8
BL	TP53	0.001691972	path:05219_3
BL	TP53	0.009062653	path:05219_4
BL	TP53	0.00011707	path:05220_12
BL	TP53	1.95E-05	path:05220_8
BL	TP53	7.56E-05	path:05220_9
bone marrow	AKNA	6.20E-07	path:04060_31
bone marrow	AKNA	6.20E-07	path:04514_10
bone marrow	AKNA	6.20E-07	path:04514_11
bone marrow	AKNA	6.20E-07	path:04514_12
bone marrow	AKNA	6.20E-07	path:04514_78
bone marrow	AKNA	6.20E-07	path:04514_9
bone marrow	AKNA	6.20E-07	path:04672_2
bone marrow	AKNA	6.20E-07	path:05144_9
bone marrow	AKNA	6.20E-07	path:05145_20
bone marrow	AKNA	6.20E-07	path:05310_2
bone marrow	AKNA	6.20E-07	path:05320_2
bone marrow	AKNA	6.20E-07	path:05320_5
bone marrow	AKNA	6.20E-07	path:05322_7
bone marrow	AKNA	6.20E-07	path:05330_12
bone marrow	AKNA	6.20E-07	path:05330_3
bone marrow	AKNA	6.20E-07	path:05416_3

bone marrow	IRF5	0.005011517	path:04060_38
bone marrow	IRF5	0.005011517	path:04620_11
bone marrow	IRF5	0.005011517	path:04620_2
bone marrow	IRF5	0.005011517	path:04650_6
bone marrow	IRF5	0.005011517	path:05160_6
bone marrow	SPI1	0.00153244	path:04060_41
bone marrow	SPI1	0.005348786	path:04145_2
bone marrow	SPI1	3.11E-06	path:04145_6
bone marrow	SPI1	0.007630608	path:04514_55
bone marrow	SPI1	0.003570772	path:04514_62
bone marrow	SPI1	0.008430928	path:04650_10
bone marrow	SPI1	0.007340609	path:04650_12
bone marrow	SPI1	0.003805584	path:04650_13
bone marrow	SPI1	0.003570772	path:04670_10
bone marrow	SPI1	0.00153244	path:04670_14
bone marrow	SPI1	0.000961365	path:04670_15
bone marrow	SPI1	0.000252274	path:04670_2
bone marrow	SPI1	0.000110411	path:05140_4
bone marrow	SPI1	0.005348786	path:05150_17
bone marrow	SPI1	0.000902646	path:05200_43
bone marrow	TP53	0.007702858	path:04010_15
bone marrow	TP53	0.009062653	path:04010_16
bone marrow	TP53	0.007443002	path:04010_21
bone marrow	TP53	0.002466645	path:04012_12
bone marrow	TP53	2.55E-11	path:04110_1
bone marrow	TP53	3.28E-07	path:04110_17
bone marrow	TP53	1.48E-05	path:04110_19
bone marrow	TP53	0.001943417	path:04110_20
bone marrow	TP53	7.56E-05	path:04110_22
bone marrow	TP53	0.000198814	path:04110_23
bone marrow	TP53	0.001943417	path:04110_27
bone marrow	TP53	0.000210553	path:04110_3
bone marrow	TP53	0.004511216	path:04110_4
bone marrow	TP53	4.42E-09	path:04110_7

bone marrow	TP53	0.004291125	path:04110_8
bone marrow	TP53	0	path:04115_1
bone marrow	TP53	0	path:04115_2
bone marrow	TP53	0	path:04115_3
bone marrow	TP53	0	path:04115_4
bone marrow	TP53	0	path:04115_7
bone marrow	TP53	0.000421718	path:04210_20
bone marrow	TP53	0.000275763	path:04210_23
bone marrow	TP53	0.000275763	path:04210_24
bone marrow	TP53	0.009131774	path:04510_10
bone marrow	TP53	0.004511216	path:04510_15
bone marrow	TP53	0.002466645	path:04510_16
bone marrow	TP53	0.000580605	path:04510_17
bone marrow	TP53	0.001774645	path:04510_18
bone marrow	TP53	0.006951959	path:04510_25
bone marrow	TP53	0.006951959	path:04510_5
bone marrow	TP53	0.009576653	path:04510_6
bone marrow	TP53	0.001110697	path:04510_7
bone marrow	TP53	0.00935253	path:04510_8
bone marrow	TP53	0.009062653	path:04621_7
bone marrow	TP53	0.000218217	path:04722_19
bone marrow	TP53	0.002756828	path:04912_8
bone marrow	TP53	0.009062653	path:05014_4
bone marrow	TP53	0.007459365	path:05160_12
bone marrow	TP53	0.009062653	path:05160_15
bone marrow	TP53	0.000338058	path:05200_10
bone marrow	TP53	0.000275763	path:05200_11
bone marrow	TP53	0.000275763	path:05200_12
bone marrow	TP53	0.000968321	path:05200_15
bone marrow	TP53	0.00392722	path:05200_17
bone marrow	TP53	0.002997803	path:05200_19
bone marrow	TP53	0.00011707	path:05200_20
bone marrow	TP53	0.000275763	path:05200_4
bone marrow	TP53	7.56E-05	path:05200_47

bone marrow	TP53	1.95E-05	path:05200_48
bone marrow	TP53	0.000367024	path:05200_5
bone marrow	TP53	0.000172314	path:05200_51
bone marrow	TP53	1.95E-05	path:05200_8
bone marrow	TP53	0.007459365	path:05213_2
bone marrow	TP53	0.003580594	path:05214_1
bone marrow	TP53	0.000210553	path:05214_10
bone marrow	TP53	0.001774645	path:05214_11
bone marrow	TP53	1.95E-05	path:05214_12
bone marrow	TP53	0.00011707	path:05214_13
bone marrow	TP53	7.56E-05	path:05214_14
bone marrow	TP53	0.003580594	path:05214_4
bone marrow	TP53	0.004511216	path:05215_10
bone marrow	TP53	0.000275763	path:05215_11
bone marrow	TP53	0.00331496	path:05215_3
bone marrow	TP53	0.002754132	path:05218_2
bone marrow	TP53	0.001774645	path:05218_5
bone marrow	TP53	1.95E-05	path:05218_6
bone marrow	TP53	0.001385048	path:05218_8
bone marrow	TP53	0.001691972	path:05219_3
bone marrow	TP53	0.009062653	path:05219_4
bone marrow	TP53	0.00011707	path:05220_12
bone marrow	TP53	1.95E-05	path:05220_8
bone marrow	TP53	7.56E-05	path:05220_9
brain	CREB1	0.004288489	path:04514_1
brain	CREB1	0.004288489	path:04514_3
brain	CREB1	0.004288489	path:04612_3
brain	CREB1	0.004131401	path:04620_12
brain	CREB1	0.004131401	path:04620_16
brain	CREB1	0.001277819	path:04620_17
brain	CREB1	0.000627719	path:04620_18
brain	CREB1	0.001277819	path:04620_22
brain	CREB1	0.001277819	path:04620_9
brain	CREB1	0.004131401	path:04672_4

brain	CREB1	0.004131401	path:04672_5
brain	CREB1	0.004131401	path:04940_1
brain	CREB1	0.004288489	path:05150_14
brain	CREB1	0.009649083	path:05200_18
brain	CREB1	0.004131401	path:05310_1
brain	CREB1	0.004131401	path:05310_3
brain	CREB1	0.004131401	path:05320_1
brain	CREB1	0.004131401	path:05320_3
brain	CREB1	0.004131401	path:05320_4
brain	CREB1	0.004131401	path:05320_6
brain	CREB1	0.004131401	path:05322_5
brain	CREB1	0.004131401	path:05322_6
brain	CREB1	0.004131401	path:05330_1
brain	CREB1	0.004131401	path:05330_2
brain	CREB1	0.004131401	path:05330_4
brain	CREB1	0.004131401	path:05332_1
brain	CREB1	0.004131401	path:05416_4
brain	NR4A1	0.00077713	path:00140_10
brain	NR4A1	6.42E-06	path:00140_13
brain	NR4A1	0.00077713	path:00140_14
brain	NR4A1	0.00077713	path:00140_16
brain	NR4A1	0.00077713	path:00140_18
brain	NR4A1	0.000832486	path:00140_19
brain	NR4A1	0.00077713	path:00140_20
brain	NR4A1	0.00077713	path:00140_3
brain	NR4A1	0.00077713	path:00140_4
brain	NR4A1	0.000960385	path:00140_5
brain	NR4A1	0.001089761	path:00140_6
brain	NR4A1	0.000832486	path:00140_7
brain	NR4A1	0.00077713	path:00140_8
brain	NR4A1	0.00077713	path:00140_9
brain	SREBF1	0.009882974	path:00100_9
brain	SREBF1	0.009882974	path:03320_2
brain	SREBF1	3.27E-05	path:04910_5

brain	SREBF1	0.00143191	path:04910_6
brain	TP53	0.007702858	path:04010_15
brain	TP53	0.009062653	path:04010_16
brain	TP53	0.007443002	path:04010_21
brain	TP53	0.002466645	path:04012_12
brain	TP53	2.55E-11	path:04110_1
brain	TP53	3.28E-07	path:04110_17
brain	TP53	1.48E-05	path:04110_19
brain	TP53	0.001943417	path:04110_20
brain	TP53	7.56E-05	path:04110_22
brain	TP53	0.000198814	path:04110_23
brain	TP53	0.001943417	path:04110_27
brain	TP53	0.000210553	path:04110_3
brain	TP53	0.004511216	path:04110_4
brain	TP53	4.42E-09	path:04110_7
brain	TP53	0.004291125	path:04110_8
brain	TP53	0	path:04115_1
brain	TP53	0	path:04115_2
brain	TP53	0	path:04115_3
brain	TP53	0	path:04115_4
brain	TP53	0	path:04115_7
brain	TP53	0.000421718	path:04210_20
brain	TP53	0.000275763	path:04210_23
brain	TP53	0.000275763	path:04210_24
brain	TP53	0.009131774	path:04510_10
brain	TP53	0.004511216	path:04510_15
brain	TP53	0.002466645	path:04510_16
brain	TP53	0.000580605	path:04510_17
brain	TP53	0.001774645	path:04510_18
brain	TP53	0.006951959	path:04510_25
brain	TP53	0.006951959	path:04510_5
brain	TP53	0.009576653	path:04510_6
brain	TP53	0.001110697	path:04510_7
brain	TP53	0.00935253	path:04510_8

brain	TP53	0.009062653	path:04621_7
brain	TP53	0.000218217	path:04722_19
brain	TP53	0.002756828	path:04912_8
brain	TP53	0.009062653	path:05014_4
brain	TP53	0.007459365	path:05160_12
brain	TP53	0.009062653	path:05160_15
brain	TP53	0.000338058	path:05200_10
brain	TP53	0.000275763	path:05200_11
brain	TP53	0.000275763	path:05200_12
brain	TP53	0.000968321	path:05200_15
brain	TP53	0.00392722	path:05200_17
brain	TP53	0.002997803	path:05200_19
brain	TP53	0.00011707	path:05200_20
brain	TP53	0.000275763	path:05200_4
brain	TP53	7.56E-05	path:05200_47
brain	TP53	1.95E-05	path:05200_48
brain	TP53	0.000367024	path:05200_5
brain	TP53	0.000172314	path:05200_51
brain	TP53	1.95E-05	path:05200_8
brain	TP53	0.007459365	path:05213_2
brain	TP53	0.003580594	path:05214_1
brain	TP53	0.000210553	path:05214_10
brain	TP53	0.001774645	path:05214_11
brain	TP53	1.95E-05	path:05214_12
brain	TP53	0.00011707	path:05214_13
brain	TP53	7.56E-05	path:05214_14
brain	TP53	0.003580594	path:05214_4
brain	TP53	0.004511216	path:05215_10
brain	TP53	0.000275763	path:05215_11
brain	TP53	0.00331496	path:05215_3
brain	TP53	0.002754132	path:05218_2
brain	TP53	0.001774645	path:05218_5
brain	TP53	1.95E-05	path:05218_6
brain	TP53	0.001385048	path:05218_8

brain	TP53	0.001691972	path:05219_3
brain	TP53	0.009062653	path:05219_4
brain	TP53	0.00011707	path:05220_12
brain	TP53	1.95E-05	path:05220_8
brain	TP53	7.56E-05	path:05220_9
breast	ETV4	0.000820491	path:05200_29
breast	HNF1A	0.008512637	path:00120_5
breast	HNF1A	0.008512637	path:00140_1
breast	HNF1A	3.95E-05	path:00140_12
breast	HNF1A	0.008512637	path:00140_22
breast	HNF1A	0.008512637	path:00140_23
breast	HNF1A	0.008512637	path:00140_24
breast	HNF1A	0.008512637	path:00140_25
breast	HNF1A	0.008512637	path:00140_26
breast	HNF1A	0.008512637	path:00982_6
breast	HNF1A	0.008512637	path:04610_6
breast	TCF7	0.00669167	path:04660_20
breast	TP63	2.23E-05	path:04110_1
breast	TP63	8.12E-05	path:04110_7
breast	TP63	8.12E-05	path:04115_1
breast	TP63	8.12E-05	path:04115_2
breast	TP63	8.12E-05	path:04115_3
breast	TP63	8.12E-05	path:04115_4
breast	TP63	8.12E-05	path:04115_7
breast	TP63	0.003115907	path:05200_17
breast	TP63	0.002783081	path:05200_19
breast	TP63	0.002783081	path:05200_20
breast	TP63	0.000159403	path:05200_47
breast	TP63	8.12E-05	path:05200_48
breast	TP63	8.12E-05	path:05214_12
breast	TP63	0.000159403	path:05214_14
breast	TP63	0.004120782	path:05215_11
breast	TP63	0.002837304	path:05215_3
breast	TP63	8.12E-05	path:05218_6

breast	TP63	8.12E-05	path:05220_8
breast	TP63	0.000159403	path:05220_9
Caco	GATA4	0.001238279	path:00140_10
Caco	GATA4	0.001238279	path:00140_13
Caco	GATA4	0.001238279	path:00140_16
Caco	GATA4	0.001238279	path:00140_18
Caco	GATA4	0.001238279	path:00140_19
Caco	GATA4	0.001238279	path:00140_3
Caco	GATA4	0.001238279	path:00140_4
Caco	GATA4	0.001470007	path:00140_5
Caco	GATA4	0.001710596	path:00140_6
Caco	GATA4	0.001238279	path:00140_7
Caco	GATA4	0.001226506	path:00140_8
Caco	GATA4	0.001226506	path:00140_9
Caco	GATA4	0.00061049	path:00590_1
Caco	GATA4	0.00061049	path:00590_2
Caco	GATA4	0.00061049	path:00590_3
Caco	GATA4	0.00061049	path:00590_4
Caco	GATA4	0.00061049	path:00591_1
Caco	GATA4	0.00061049	path:00980_2
Caco	GATA4	0.001958811	path:00982_10
Caco	GATA4	0.001238279	path:00982_4
Caco	GATA4	0.001238279	path:00982_5
Caco	GATA4	0.001238279	path:00982_9
Caco	GATA6	0.00185453	path:00590_1
Caco	GATA6	0.00185453	path:00590_2
Caco	GATA6	0.00185453	path:00590_3
Caco	GATA6	0.00185453	path:00590_4
Caco	GATA6	0.00185453	path:00591_1
Caco	GATA6	0.008695178	path:00982_10
Caco	GATA6	0.005342537	path:00982_4
Caco	GATA6	0.005342537	path:00982_5
Caco	GATA6	0.005342537	path:00982_9
Caco	HNF1A	0.008512637	path:00120_5

Caco	HNF1A	0.008512637	path:00140_1
Caco	HNF1A	3.95E-05	path:00140_12
Caco	HNF1A	0.008512637	path:00140_22
Caco	HNF1A	0.008512637	path:00140_23
Caco	HNF1A	0.008512637	path:00140_24
Caco	HNF1A	0.008512637	path:00140_25
Caco	HNF1A	0.008512637	path:00140_26
Caco	HNF1A	0.008512637	path:00982_6
Caco	HNF1A	0.008512637	path:04610_6
colon	HNF4A	0.00730316	path:00120_15
colon	HNF4A	0.003812707	path:00120_19
colon	HNF4A	0.001698537	path:00120_6
colon	HNF4A	0.003812707	path:00120_7
colon	HNF4A	0.000100826	path:00120_9
colon	HNF4A	0.001808381	path:00590_1
colon	HNF4A	0.001722691	path:00590_2
colon	HNF4A	0.001722691	path:00590_3
colon	HNF4A	0.001898001	path:00590_4
colon	HNF4A	0.000515682	path:00591_1
colon	HNF4A	2.36E-05	path:00830_2
colon	HNF4A	0.000733563	path:00980_2
colon	HNF4A	2.52E-07	path:00982_10
colon	HNF4A	5.38E-08	path:00982_4
colon	HNF4A	5.38E-08	path:00982_5
colon	HNF4A	5.38E-08	path:00982_9
colon	HNF4A	0.001131883	path:03320_1
colon	HNF4A	0.000100826	path:03320_2
colon	HNF4A	4.35E-06	path:04610_2
colon	HNF4A	4.35E-06	path:04610_3
colon	HNF4A	0.000100826	path:04610_4
colon	HNF4A	6.69E-06	path:04610_5
colon	HNF4A	0.000179282	path:04610_6
colon	HNF4A	0.001698537	path:04610_8
colon	HNF4A	0.000287791	path:04950_4

colon	TP53	0.007702858	path:04010_15
colon	TP53	0.009062653	path:04010_16
colon	TP53	0.007443002	path:04010_21
colon	TP53	0.002466645	path:04012_12
colon	TP53	2.55E-11	path:04110_1
colon	TP53	3.28E-07	path:04110_17
colon	TP53	1.48E-05	path:04110_19
colon	TP53	0.001943417	path:04110_20
colon	TP53	7.56E-05	path:04110_22
colon	TP53	0.000198814	path:04110_23
colon	TP53	0.001943417	path:04110_27
colon	TP53	0.000210553	path:04110_3
colon	TP53	0.004511216	path:04110_4
colon	TP53	4.42E-09	path:04110_7
colon	TP53	0.004291125	path:04110_8
colon	TP53	0	path:04115_1
colon	TP53	0	path:04115_2
colon	TP53	0	path:04115_3
colon	TP53	0	path:04115_4
colon	TP53	0	path:04115_7
colon	TP53	0.000421718	path:04210_20
colon	TP53	0.000275763	path:04210_23
colon	TP53	0.000275763	path:04210_24
colon	TP53	0.009131774	path:04510_10
colon	TP53	0.004511216	path:04510_15
colon	TP53	0.002466645	path:04510_16
colon	TP53	0.000580605	path:04510_17
colon	TP53	0.001774645	path:04510_18
colon	TP53	0.006951959	path:04510_25
colon	TP53	0.006951959	path:04510_5
colon	TP53	0.009576653	path:04510_6
colon	TP53	0.001110697	path:04510_7
colon	TP53	0.00935253	path:04510_8
colon	TP53	0.009062653	path:04621_7

colon	TP53	0.000218217	path:04722_19
colon	TP53	0.002756828	path:04912_8
colon	TP53	0.009062653	path:05014_4
colon	TP53	0.007459365	path:05160_12
colon	TP53	0.009062653	path:05160_15
colon	TP53	0.000338058	path:05200_10
colon	TP53	0.000275763	path:05200_11
colon	TP53	0.000275763	path:05200_12
colon	TP53	0.000968321	path:05200_15
colon	TP53	0.00392722	path:05200_17
colon	TP53	0.002997803	path:05200_19
colon	TP53	0.00011707	path:05200_20
colon	TP53	0.000275763	path:05200_4
colon	TP53	7.56E-05	path:05200_47
colon	TP53	1.95E-05	path:05200_48
colon	TP53	0.000367024	path:05200_5
colon	TP53	0.000172314	path:05200_51
colon	TP53	1.95E-05	path:05200_8
colon	TP53	0.007459365	path:05213_2
colon	TP53	0.003580594	path:05214_1
colon	TP53	0.000210553	path:05214_10
colon	TP53	0.001774645	path:05214_11
colon	TP53	1.95E-05	path:05214_12
colon	TP53	0.00011707	path:05214_13
colon	TP53	7.56E-05	path:05214_14
colon	TP53	0.003580594	path:05214_4
colon	TP53	0.004511216	path:05215_10
colon	TP53	0.000275763	path:05215_11
colon	TP53	0.00331496	path:05215_3
colon	TP53	0.002754132	path:05218_2
colon	TP53	0.001774645	path:05218_5
colon	TP53	1.95E-05	path:05218_6
colon	TP53	0.001385048	path:05218_8
colon	TP53	0.001691972	path:05219_3

colon	TP53	0.009062653	path:05219_4
colon	TP53	0.00011707	path:05220_12
colon	TP53	1.95E-05	path:05220_8
colon	TP53	7.56E-05	path:05220_9
heart	CREB1	0.004288489	path:04514_1
heart	CREB1	0.004288489	path:04514_3
heart	CREB1	0.004288489	path:04612_3
heart	CREB1	0.004131401	path:04620_12
heart	CREB1	0.004131401	path:04620_16
heart	CREB1	0.001277819	path:04620_17
heart	CREB1	0.000627719	path:04620_18
heart	CREB1	0.001277819	path:04620_22
heart	CREB1	0.001277819	path:04620_9
heart	CREB1	0.004131401	path:04672_4
heart	CREB1	0.004131401	path:04672_5
heart	CREB1	0.004131401	path:04940_1
heart	CREB1	0.004288489	path:05150_14
heart	CREB1	0.009649083	path:05200_18
heart	CREB1	0.004131401	path:05310_1
heart	CREB1	0.004131401	path:05310_3
heart	CREB1	0.004131401	path:05320_1
heart	CREB1	0.004131401	path:05320_3
heart	CREB1	0.004131401	path:05320_4
heart	CREB1	0.004131401	path:05320_6
heart	CREB1	0.004131401	path:05322_5
heart	CREB1	0.004131401	path:05322_6
heart	CREB1	0.004131401	path:05330_1
heart	CREB1	0.004131401	path:05330_2
heart	CREB1	0.004131401	path:05330_4
heart	CREB1	0.004131401	path:05332_1
heart	CREB1	0.004131401	path:05416_4
heart	NR1I3	0.000186607	path:00830_2
heart	NR1I3	0.006275482	path:00980_1
heart	NR1I3	0.000535628	path:00982_4

heart	NR1I3	0.000535628	path:00982_5
heart	NR1I3	0.000535628	path:00982_9
heart	SREBF1	0.009882974	path:00100_9
heart	SREBF1	0.009882974	path:03320_2
heart	SREBF1	3.27E-05	path:04910_5
heart	SREBF1	0.00143191	path:04910_6
heart	TP63	2.23E-05	path:04110_1
heart	TP63	8.12E-05	path:04110_7
heart	TP63	8.12E-05	path:04115_1
heart	TP63	8.12E-05	path:04115_2
heart	TP63	8.12E-05	path:04115_3
heart	TP63	8.12E-05	path:04115_4
heart	TP63	8.12E-05	path:04115_7
heart	TP63	0.003115907	path:05200_17
heart	TP63	0.002783081	path:05200_19
heart	TP63	0.002783081	path:05200_20
heart	TP63	0.000159403	path:05200_47
heart	TP63	8.12E-05	path:05200_48
heart	TP63	8.12E-05	path:05214_12
heart	TP63	0.000159403	path:05214_14
heart	TP63	0.004120782	path:05215_11
heart	TP63	0.002837304	path:05215_3
heart	TP63	8.12E-05	path:05218_6
heart	TP63	8.12E-05	path:05220_8
heart	TP63	0.000159403	path:05220_9
HeLa	NFIC	0.004396514	path:00591_1
HeLa	NFIC	0.005988829	path:00982_11
HeLa	NFIC	0.00921451	path:00982_2
HeLa	NFIC	0.005988829	path:00982_3
HeLa	NFIC	0.00787147	path:00982_4
HeLa	NFIC	0.00787147	path:00982_5
HeLa	NFIC	0.004396514	path:00982_7
HeLa	NFIC	0.004396514	path:00982_8
HeLa	NFIC	0.00787147	path:00982_9

HeLa	NFIC	0.00787147	path:04010_30
HeLa	NFIC	0.00787147	path:04010_31
HeLa	NFIC	0.004396514	path:04080_7
HeLa	NFIC	0.008000492	path:04115_1
HeLa	NFIC	0.00787147	path:04115_2
HeLa	NFIC	0.00787147	path:04115_3
HeLa	NFIC	0.00787147	path:04115_4
HeLa	NFIC	0.00787147	path:04115_7
HeLa	NFIC	0.00787147	path:04660_8
HeLa	NFIC	0.00787147	path:04662_6
HeLa	NFIC	0.00787147	path:04722_19
HeLa	NFIC	0.00787147	path:05160_15
HeLa	NFIC	0.007050709	path:05200_47
HeLa	NFIC	0.004396514	path:05200_48
HeLa	NFIC	0.00787147	path:05200_51
HeLa	NFIC	0.004396514	path:05214_12
HeLa	NFIC	0.00787147	path:05214_13
HeLa	NFIC	0.007050709	path:05214_14
HeLa	NFIC	0.004396514	path:05218_5
HeLa	NFIC	0.004396514	path:05218_6
HeLa	NFIC	0.00787147	path:05220_12
HeLa	NFIC	0.004396514	path:05220_8
HeLa	NFIC	0.007050709	path:05220_9
HeLa	NFYA	0.00144529	path:04110_1
HeLa	NFYA	0.005383343	path:04110_11
HeLa	NFYA	0.003794515	path:04110_12
HeLa	NFYA	0.000340548	path:04110_17
HeLa	NFYA	0.005565492	path:04110_19
HeLa	NFYA	0.002132484	path:04110_22
HeLa	NFYA	0.000299258	path:04110_23
HeLa	NFYA	0.004551637	path:04110_26
HeLa	NFYA	0.000299258	path:04110_3
HeLa	NFYA	0.004713566	path:04110_4
HeLa	NFYA	0.000340548	path:04110_7

HeLa	NFYA	0.004551637	path:04110_8
HeLa	NFYA	0.001111281	path:04115_1
HeLa	NFYA	0.000123767	path:04115_2
HeLa	NFYA	0.000776011	path:04115_3
HeLa	NFYA	0.000776011	path:04115_4
HeLa	NFYA	0.000776011	path:04115_7
HeLa	NR2F1	0.000132961	path:03320_2
HeLa	REL	0.004162941	path:04060_8
HeLa	REL	0.005200652	path:04612_7
HeLa	REL	0.00019873	path:04650_22
HeLa	REL	0.00019873	path:04650_23
HeLa	SP1	0.006087664	path:00120_1
HeLa	SP1	0.003885876	path:00120_2
HeLa	SP1	0.003885876	path:00120_3
HeLa	SP1	0.001893526	path:00140_1
HeLa	SP1	1.44E-05	path:00140_10
HeLa	SP1	0.001534931	path:00140_11
HeLa	SP1	0.006087664	path:00140_12
HeLa	SP1	2.77E-05	path:00140_13
HeLa	SP1	0.003885876	path:00140_14
HeLa	SP1	0.000245716	path:00140_16
HeLa	SP1	0.000245716	path:00140_18
HeLa	SP1	2.77E-05	path:00140_19
HeLa	SP1	0.000441307	path:00140_20
HeLa	SP1	0.001893526	path:00140_25
HeLa	SP1	1.44E-05	path:00140_3
HeLa	SP1	1.44E-05	path:00140_4
HeLa	SP1	5.07E-05	path:00140_5
HeLa	SP1	8.66E-05	path:00140_6
HeLa	SP1	2.77E-05	path:00140_7
HeLa	SP1	0.000123921	path:00140_8
HeLa	SP1	0.000123921	path:00140_9
HeLa	SP1	0.005536058	path:00240_16
HeLa	SP1	0.003391309	path:00240_17

HeLa	SP1	0.001690362	path:00240_2
HeLa	SP1	0.009002988	path:00240_5
HeLa	SP1	0.005536058	path:00330_1
HeLa	SP1	0.005126386	path:00330_9
HeLa	SP1	0.006192277	path:00350_2
HeLa	SP1	0.006192277	path:00350_3
HeLa	SP1	0.001393247	path:00980_2
HeLa	SP1	0.002175912	path:00982_10
HeLa	SP1	0.003931316	path:04010_10
HeLa	SP1	0.000441307	path:04010_26
HeLa	SP1	0.000123921	path:04010_5
HeLa	SP1	0.002149604	path:04060_22
HeLa	SP1	0.001014956	path:04060_32
HeLa	SP1	0.007433507	path:04060_47
HeLa	SP1	0.00916136	path:04110_1
HeLa	SP1	1.86E-10	path:04110_17
HeLa	SP1	4.28E-11	path:04110_18
HeLa	SP1	1.05E-10	path:04110_19
HeLa	SP1	2.92E-09	path:04110_20
HeLa	SP1	1.43E-05	path:04110_22
HeLa	SP1	5.22E-09	path:04110_23
HeLa	SP1	0.006192277	path:04110_25
HeLa	SP1	7.70E-08	path:04110_26
HeLa	SP1	2.92E-09	path:04110_27
HeLa	SP1	6.46E-09	path:04110_3
HeLa	SP1	6.46E-09	path:04110_4
HeLa	SP1	4.97E-07	path:04110_7
HeLa	SP1	3.47E-10	path:04115_1
HeLa	SP1	6.74E-05	path:04115_2
HeLa	SP1	4.23E-05	path:04115_3
HeLa	SP1	6.18E-06	path:04115_4
HeLa	SP1	4.96E-05	path:04115_7
HeLa	SP1	7.61E-06	path:04144_2
HeLa	SP1	0.002175912	path:04210_20

HeLa	SP1	0.001107767	path:04210_24
HeLa	SP1	1.33E-05	path:04310_6
HeLa	SP1	1.77E-05	path:04310_7
HeLa	SP1	7.85E-13	path:04510_1
HeLa	SP1	1.05E-10	path:04510_10
HeLa	SP1	0.001603207	path:04510_13
HeLa	SP1	0.001164758	path:04510_15
HeLa	SP1	0.002771235	path:04510_16
HeLa	SP1	0.000141426	path:04510_17
HeLa	SP1	8.07E-08	path:04510_18
HeLa	SP1	1.96E-12	path:04510_19
HeLa	SP1	1.43E-05	path:04510_20
HeLa	SP1	0.000718519	path:04510_23
HeLa	SP1	0.002175912	path:04510_24
HeLa	SP1	0.0024986	path:04510_25
HeLa	SP1	6.74E-05	path:04510_4
HeLa	SP1	0.0024986	path:04510_5
HeLa	SP1	1.07E-10	path:04510_6
HeLa	SP1	0.000448433	path:04510_7
HeLa	SP1	6.16E-10	path:04510_8
HeLa	SP1	2.01E-05	path:04512_10
HeLa	SP1	5.55E-06	path:04512_11
HeLa	SP1	1.66E-06	path:04512_12
HeLa	SP1	7.86E-06	path:04512_13
HeLa	SP1	0.000101569	path:04512_14
HeLa	SP1	0.000245716	path:04512_18
HeLa	SP1	0.000549822	path:04512_19
HeLa	SP1	0.002149604	path:04512_20
HeLa	SP1	0.008608739	path:04512_22
HeLa	SP1	0.00016592	path:04512_27
HeLa	SP1	1.66E-05	path:04512_3
HeLa	SP1	2.72E-07	path:04512_5
HeLa	SP1	6.24E-07	path:04512_6
HeLa	SP1	9.36E-06	path:04512_9

HeLa	SP1	0.005126386	path:04520_3
HeLa	SP1	0.001603207	path:04520_5
HeLa	SP1	0.000412181	path:04520_7
HeLa	SP1	1.22E-05	path:04540_13
HeLa	SP1	9.21E-07	path:04610_3
HeLa	SP1	5.55E-06	path:04610_4
HeLa	SP1	9.21E-07	path:04610_6
HeLa	SP1	0.000133832	path:04610_7
HeLa	SP1	0.000203885	path:04610_8
HeLa	SP1	0.000203885	path:04620_18
HeLa	SP1	0.001893526	path:04620_9
HeLa	SP1	0.000231547	path:04630_1
HeLa	SP1	0.001390056	path:04630_2
HeLa	SP1	1.97E-06	path:04630_3
HeLa	SP1	5.95E-06	path:04630_4
HeLa	SP1	4.84E-05	path:04650_20
HeLa	SP1	0.008608739	path:04650_7
HeLa	SP1	0.000718519	path:04722_19
HeLa	SP1	7.92E-07	path:04810_29
HeLa	SP1	1.33E-05	path:04912_8
HeLa	SP1	0.007433507	path:04940_4
HeLa	SP1	0.006087664	path:05014_3
HeLa	SP1	0.008608739	path:05014_4
HeLa	SP1	0.007433507	path:05016_8
HeLa	SP1	0.001014956	path:05100_3
HeLa	SP1	0.008608739	path:05140_10
HeLa	SP1	0.000245716	path:05140_6
HeLa	SP1	0.001107767	path:05142_10
HeLa	SP1	0.003931316	path:05142_11
HeLa	SP1	0.003885876	path:05142_17
HeLa	SP1	0.003885876	path:05142_18
HeLa	SP1	0.000441307	path:05142_20
HeLa	SP1	0.000718519	path:05142_21
HeLa	SP1	0.001107767	path:05142_4

HeLa	SP1	0.006087664	path:05145_19
HeLa	SP1	0.001014956	path:05145_21
HeLa	SP1	0.006087664	path:05145_8
HeLa	SP1	1.23E-06	path:05146_1
HeLa	SP1	7.92E-07	path:05146_2
HeLa	SP1	0.002149604	path:05146_5
HeLa	SP1	9.10E-07	path:05146_9
HeLa	SP1	0.003935354	path:05200_19
HeLa	SP1	0.003935354	path:05200_20
HeLa	SP1	0.00927462	path:05200_24
HeLa	SP1	9.21E-07	path:05200_29
HeLa	SP1	2.01E-05	path:05200_3
HeLa	SP1	0.000718519	path:05200_31
HeLa	SP1	0.008608739	path:05200_38
HeLa	SP1	0.000289957	path:05200_4
HeLa	SP1	0.000718519	path:05200_42
HeLa	SP1	2.07E-08	path:05200_47
HeLa	SP1	1.33E-05	path:05200_48
HeLa	SP1	5.07E-05	path:05200_50
HeLa	SP1	5.54E-09	path:05200_51
HeLa	SP1	1.55E-08	path:05200_52
HeLa	SP1	4.11E-06	path:05200_53
HeLa	SP1	0.001014956	path:05200_56
HeLa	SP1	0.000441307	path:05210_10
HeLa	SP1	0.000718519	path:05210_12
HeLa	SP1	0.000718519	path:05210_7
HeLa	SP1	1.44E-05	path:05212_13
HeLa	SP1	0.008608739	path:05212_14
HeLa	SP1	1.33E-05	path:05214_12
HeLa	SP1	1.18E-07	path:05214_13
HeLa	SP1	2.07E-08	path:05214_14
HeLa	SP1	9.21E-07	path:05214_15
HeLa	SP1	0.005536058	path:05214_2
HeLa	SP1	0.000245716	path:05214_21

HeLa	SP1	0.005536058	path:05214_5
HeLa	SP1	0.001164758	path:05215_10
HeLa	SP1	0.008608739	path:05215_7
HeLa	SP1	0.000441307	path:05216_4
HeLa	SP1	0.000411461	path:05218_4
HeLa	SP1	0.000133832	path:05218_5
HeLa	SP1	1.33E-05	path:05218_6
HeLa	SP1	9.21E-07	path:05218_7
HeLa	SP1	7.27E-05	path:05218_8
HeLa	SP1	0.000310459	path:05219_3
HeLa	SP1	1.44E-05	path:05219_4
HeLa	SP1	9.21E-07	path:05220_11
HeLa	SP1	1.18E-07	path:05220_12
HeLa	SP1	0.002149604	path:05220_5
HeLa	SP1	1.33E-05	path:05220_8
HeLa	SP1	2.07E-08	path:05220_9
HeLa	SP1	0.006192277	path:05221_1
HeLa	SP1	5.36E-07	path:05222_1
HeLa	SP1	0.000133832	path:05222_2
HeLa	SP1	2.01E-05	path:05222_4
HeLa	SP1	0.001603207	path:05222_7
HeLa	SP1	1.44E-05	path:05223_3
HeLa	SP1	0.007433507	path:05320_9
HeLa	SP1	0.007433507	path:05330_5
HeLa	SP1	0.007433507	path:05332_2
HeLa	SP1	0.007433507	path:05332_5
HeLa	SP1	1.77E-05	path:05414_2
HeLa	SRF	0.004545882	path:05020_8
HeLa	SRF	0.00379009	path:05020_9
HeLa	SRF	0.003033557	path:05410_2
HeLa	SRF	0.003033557	path:05414_3
HeLa	SRF	0.003033557	path:05416_1
HeLa	TFAP2A	0.005723968	path:04020_2
HeLa	TFAP2A	0.003857334	path:04080_7

HeLa	TFAP2A	0.00057548	path:04144_2
HeLa	TFAP2A	0.001269093	path:04510_10
HeLa	TFAP2A	0.005458314	path:04510_15
HeLa	TFAP2A	0.003332624	path:04510_16
HeLa	TFAP2A	3.81E-05	path:04510_17
HeLa	TFAP2A	1.42E-05	path:04510_18
HeLa	TFAP2A	0.007739985	path:04510_25
HeLa	TFAP2A	0.007739985	path:04510_5
HeLa	TFAP2A	0.001276164	path:04510_6
HeLa	TFAP2A	8.35E-05	path:04510_7
HeLa	TFAP2A	0.001271633	path:04510_8
HeLa	TFAP2A	0.002407854	path:04520_2
HeLa	TFAP2A	0.001262516	path:04520_3
HeLa	TFAP2A	0.00057548	path:04520_5
HeLa	TFAP2A	0.001262516	path:04520_7
HeLa	TFAP2A	0.009046641	path:04520_9
HeLa	TFAP2A	0.003857334	path:04912_8
HeLa	TFAP2A	2.20E-05	path:05200_10
HeLa	TFAP2A	1.91E-05	path:05200_11
HeLa	TFAP2A	1.91E-05	path:05200_12
HeLa	TFAP2A	6.62E-06	path:05200_15
HeLa	TFAP2A	0.000448647	path:05200_21
HeLa	TFAP2A	8.35E-05	path:05200_29
HeLa	TFAP2A	1.91E-05	path:05200_4
HeLa	TFAP2A	2.22E-05	path:05200_5
HeLa	TFAP2A	2.16E-06	path:05200_8
HeLa	TFAP2A	0.002699451	path:05212_11
HeLa	TFAP2A	0.003332624	path:05212_12
HeLa	TFAP2A	0.004058678	path:05214_1
HeLa	TFAP2A	0.000317116	path:05214_10
HeLa	TFAP2A	0.002407854	path:05214_11
HeLa	TFAP2A	0.007739985	path:05214_16
HeLa	TFAP2A	0.004058678	path:05214_17
HeLa	TFAP2A	0.004058678	path:05214_2

HeLa	TFAP2A	0.004058678	path:05214_3
HeLa	TFAP2A	0.004058678	path:05214_4
HeLa	TFAP2A	0.004058678	path:05214_5
HeLa	TFAP2A	0.004058678	path:05214_6
HeLa	TFAP2A	0.007739985	path:05214_7
HeLa	TFAP2A	0.004058678	path:05214_8
HeLa	TFAP2A	0.000317116	path:05215_10
HeLa	TFAP2A	0.000436788	path:05215_11
HeLa	TFAP2A	0.001124594	path:05215_8
HeLa	TFAP2A	0.002407854	path:05219_2
HeLa	VDR	0.009786942	path:00140_1
HeLa	VDR	0.008771608	path:00140_11
HeLa	VDR	0.009786942	path:00140_25
HeLa	VDR	0.009786942	path:00140_26
HeLa	VDR	0.008771608	path:00591_1
HeLa	VDR	0.00973729	path:00980_2
HeLa	VDR	0.004788204	path:00982_10
HeLa	VDR	0.001635842	path:00982_11
HeLa	VDR	0.003675018	path:00982_2
HeLa	VDR	0.001635842	path:00982_3
HeLa	VDR	0.003361044	path:00982_4
HeLa	VDR	0.003361044	path:00982_5
HeLa	VDR	0.008771608	path:00982_6
HeLa	VDR	0.001635842	path:00982_7
HeLa	VDR	0.001635842	path:00982_8
HeLa	VDR	0.003361044	path:00982_9
HeLa	VDR	0.008771608	path:04012_1
HeLa	VDR	0.008771608	path:04012_11
HeLa	VDR	0.008771608	path:04012_12
HeLa	VDR	0.0090937	path:04012_14
HeLa	VDR	0.008771608	path:04012_4
HeLa	VDR	0.008771608	path:04012_7
HeLa	VDR	0.008771608	path:05200_9
HeLa	VDR	0.008771608	path:05214_1

HeLa	VDR	0.009415594	path:05214_10
HeLa	VDR	0.008771608	path:05214_17
HeLa	VDR	0.008771608	path:05214_2
HeLa	VDR	0.008771608	path:05214_3
HeLa	VDR	0.008771608	path:05214_4
HeLa	VDR	0.008771608	path:05214_5
HeLa	VDR	0.008771608	path:05214_6
HeLa	VDR	0.008771608	path:05214_8
HeLa	VDR	0.009786942	path:05215_11
HeLa	VDR	0.008771608	path:05218_2
HeLa	VDR	0.008771608	path:05223_6
hematopoietic	FOXA3	0.000327469	path:00140_1
hematopoietic	FOXA3	0.000188543	path:00140_11
hematopoietic	FOXA3	0.000502586	path:00140_12
hematopoietic	FOXA3	0.000327469	path:00140_25
hematopoietic	FOXA3	0.000327469	path:00140_26
hematopoietic	FOXA3	0.000312584	path:00591_1
hematopoietic	FOXA3	0.000975791	path:00830_2
hematopoietic	FOXA3	0.000327469	path:00980_2
hematopoietic	FOXA3	7.28E-05	path:00982_10
hematopoietic	FOXA3	2.48E-05	path:00982_11
hematopoietic	FOXA3	5.58E-05	path:00982_2
hematopoietic	FOXA3	2.48E-05	path:00982_3
hematopoietic	FOXA3	5.10E-05	path:00982_4
hematopoietic	FOXA3	5.10E-05	path:00982_5
hematopoietic	FOXA3	0.000248984	path:00982_6
hematopoietic	FOXA3	2.48E-05	path:00982_7
hematopoietic	FOXA3	2.48E-05	path:00982_8
hematopoietic	FOXA3	5.10E-05	path:00982_9
hematopoietic	STAT6	0.003994719	path:00590_4
hematopoietic	STAT6	0.000657478	path:04630_2
hematopoietic	STAT6	0.000657478	path:04630_3
hematopoietic	STAT6	0.000657478	path:04630_4
Hep3B	EGR1	0.009869535	path:04060_8

Hep3B	EGR1	0.00069989	path:04510_10
Hep3B	EGR1	0.00069989	path:04510_18
Hep3B	EGR1	0.003153788	path:04510_6
Hep3B	EGR1	0.003153788	path:04510_8
Hep3B	EGR1	0.00129686	path:04512_3
Hep3B	EGR1	0.003153788	path:04540_13
Hep3B	EGR1	0.003153788	path:05146_9
Hep3B	EGR1	0.007008495	path:05212_11
Hep3B	EGR1	0.008365691	path:05212_12
Hep3B	EGR1	0.003153788	path:05218_4
Hep3B	GATA2	0.002976989	path:04060_47
Hep3B	HNF4A	0.00730316	path:00120_15
Hep3B	HNF4A	0.003812707	path:00120_19
Hep3B	HNF4A	0.001698537	path:00120_6
Hep3B	HNF4A	0.003812707	path:00120_7
Hep3B	HNF4A	0.000100826	path:00120_9
Hep3B	HNF4A	0.001808381	path:00590_1
Hep3B	HNF4A	0.001722691	path:00590_2
Hep3B	HNF4A	0.001722691	path:00590_3
Hep3B	HNF4A	0.001898001	path:00590_4
Hep3B	HNF4A	0.000515682	path:00591_1
Hep3B	HNF4A	2.36E-05	path:00830_2
Hep3B	HNF4A	0.000733563	path:00980_2
Hep3B	HNF4A	2.52E-07	path:00982_10
Hep3B	HNF4A	5.38E-08	path:00982_4
Hep3B	HNF4A	5.38E-08	path:00982_5
Hep3B	HNF4A	5.38E-08	path:00982_9
Hep3B	HNF4A	0.001131883	path:03320_1
Hep3B	HNF4A	0.000100826	path:03320_2
Hep3B	HNF4A	4.35E-06	path:04610_2
Hep3B	HNF4A	4.35E-06	path:04610_3
Hep3B	HNF4A	0.000100826	path:04610_4
Hep3B	HNF4A	6.69E-06	path:04610_5
Hep3B	HNF4A	0.000179282	path:04610_6

Hep3B	HNF4A	0.001698537	path:04610_8
Hep3B	HNF4A	0.000287791	path:04950_4
HepG2	GATA2	0.002976989	path:04060_47
HepG2	GATA3	0.003994597	path:04612_4
HepG2	NFE2L2	0.003129559	path:00480_1
HepG2	NFE2L2	2.02E-06	path:00480_2
HepG2	NFE2L2	0.003129559	path:00480_3
HepG2	NFE2L2	0.003129559	path:00480_4
HepG2	NFE2L2	0.003129559	path:00480_5
HepG2	NFE2L2	0.003129559	path:00480_6
HepG2	STAT5B	0.009500448	path:04110_20
HepG2	STAT5B	0.009500448	path:04110_27
HepG2	STAT5B	0.000370487	path:04630_4
HepG2	STAT5B	0.009500448	path:05200_29
HepG2	STAT5B	0.002527186	path:05200_47
HepG2	STAT5B	0.002527186	path:05200_51
HepG2	STAT5B	0.005012521	path:05200_52
HepG2	STAT5B	0.002527186	path:05214_13
HepG2	STAT5B	0.002527186	path:05214_14
HepG2	STAT5B	0.002527186	path:05214_15
HepG2	STAT5B	0.009500448	path:05218_5
HepG2	STAT5B	0.002527186	path:05218_7
HepG2	STAT5B	0.009060668	path:05218_8
HepG2	STAT5B	0.002527186	path:05219_4
HepG2	STAT5B	0.002527186	path:05220_11
HepG2	STAT5B	0.002527186	path:05220_12
HepG2	STAT5B	0.002527186	path:05220_9
HL60	KLF4	4.70E-05	path:04110_17
HL60	KLF4	4.70E-05	path:04110_19
HL60	KLF4	0.000691257	path:04110_20
HL60	KLF4	0.001048885	path:04110_23
HL60	KLF4	0.001048885	path:04110_26
HL60	KLF4	0.000691257	path:04110_27
HL60	KLF4	0.001048885	path:04110_3

HL60	KLF4	0.001048885	path:04110_4
HL60	KLF4	0.001498093	path:04110_7
HL60	KLF4	0.003364391	path:04115_1
HL60	KLF4	0.007702669	path:04512_1
HL60	KLF4	0.007702669	path:04512_2
HL60	KLF4	0.007140402	path:04512_4
HL60	KLF4	0.007140402	path:04512_7
HL60	KLF4	0.007140402	path:04512_8
HL60	KLF4	0.007140402	path:05145_14
HL60	KLF4	0.003572583	path:05200_31
HL60	KLF4	0.002858346	path:05200_47
HL60	KLF4	0.003572583	path:05200_51
HL60	KLF4	0.007103944	path:05200_52
HL60	KLF4	0.005224296	path:05200_53
HL60	KLF4	0.003572583	path:05210_12
HL60	KLF4	0.003572583	path:05210_7
HL60	KLF4	0.003364391	path:05214_13
HL60	KLF4	0.002858346	path:05214_14
HL60	KLF4	0.003572583	path:05214_15
HL60	KLF4	0.003572583	path:05218_7
HL60	KLF4	0.003572583	path:05219_4
HL60	KLF4	0.003572583	path:05220_11
HL60	KLF4	0.003364391	path:05220_12
HL60	KLF4	0.002858346	path:05220_9
HL60	TP73	6.75E-05	path:04115_1
HL60	TP73	6.67E-05	path:04115_2
HL60	TP73	6.67E-05	path:04115_3
HL60	TP73	6.67E-05	path:04115_4
HL60	TP73	6.67E-05	path:04115_7
HL60	TP73	0.000595015	path:04722_19
HL60	VDR	0.009786942	path:00140_1
HL60	VDR	0.008771608	path:00140_11
HL60	VDR	0.009786942	path:00140_25
HL60	VDR	0.009786942	path:00140_26

HL60	VDR	0.008771608	path:00591_1
HL60	VDR	0.00973729	path:00980_2
HL60	VDR	0.004788204	path:00982_10
HL60	VDR	0.001635842	path:00982_11
HL60	VDR	0.003675018	path:00982_2
HL60	VDR	0.001635842	path:00982_3
HL60	VDR	0.003361044	path:00982_4
HL60	VDR	0.003361044	path:00982_5
HL60	VDR	0.008771608	path:00982_6
HL60	VDR	0.001635842	path:00982_7
HL60	VDR	0.001635842	path:00982_8
HL60	VDR	0.003361044	path:00982_9
HL60	VDR	0.008771608	path:04012_1
HL60	VDR	0.008771608	path:04012_11
HL60	VDR	0.008771608	path:04012_12
HL60	VDR	0.0090937	path:04012_14
HL60	VDR	0.008771608	path:04012_4
HL60	VDR	0.008771608	path:04012_7
HL60	VDR	0.008771608	path:05200_9
HL60	VDR	0.008771608	path:05214_1
HL60	VDR	0.009415594	path:05214_10
HL60	VDR	0.008771608	path:05214_17
HL60	VDR	0.008771608	path:05214_2
HL60	VDR	0.008771608	path:05214_3
HL60	VDR	0.008771608	path:05214_4
HL60	VDR	0.008771608	path:05214_5
HL60	VDR	0.008771608	path:05214_6
HL60	VDR	0.008771608	path:05214_8
HL60	VDR	0.009786942	path:05215_11
HL60	VDR	0.008771608	path:05218_2
HL60	VDR	0.008771608	path:05223_6
HUVEC	EGR1	0.009869535	path:04060_8
HUVEC	EGR1	0.00069989	path:04510_10
HUVEC	EGR1	0.00069989	path:04510_18

HUVEC	EGR1	0.003153788	path:04510_6
HUVEC	EGR1	0.003153788	path:04510_8
HUVEC	EGR1	0.00129686	path:04512_3
HUVEC	EGR1	0.003153788	path:04540_13
HUVEC	EGR1	0.003153788	path:05146_9
HUVEC	EGR1	0.007008495	path:05212_11
HUVEC	EGR1	0.008365691	path:05212_12
HUVEC	EGR1	0.003153788	path:05218_4
HUVEC	REL	0.004162941	path:04060_8
HUVEC	REL	0.005200652	path:04612_7
HUVEC	REL	0.00019873	path:04650_22
HUVEC	REL	0.00019873	path:04650_23
HUVEC	RELA	0.000137451	path:04060_62
HUVEC	RELA	2.04E-07	path:04062_1
HUVEC	RELA	3.87E-08	path:04062_2
HUVEC	RELA	0.001869278	path:04110_17
HUVEC	RELA	0.001781655	path:04110_19
HUVEC	RELA	0.001869278	path:04110_7
HUVEC	RELA	0.0005539	path:04115_1
HUVEC	RELA	2.11E-06	path:04620_1
HUVEC	RELA	4.80E-09	path:04620_12
HUVEC	RELA	1.51E-07	path:04620_13
HUVEC	RELA	2.07E-07	path:04620_14
HUVEC	RELA	1.03E-08	path:04620_16
HUVEC	RELA	6.51E-08	path:04620_17
HUVEC	RELA	8.85E-09	path:04620_18
HUVEC	RELA	6.51E-08	path:04620_22
HUVEC	RELA	7.78E-08	path:04620_9
HUVEC	RELA	0.00197363	path:04650_22
HUVEC	RELA	0.001781655	path:04650_23
HUVEC	SP1	0.006087664	path:00120_1
HUVEC	SP1	0.003885876	path:00120_2
HUVEC	SP1	0.003885876	path:00120_3
HUVEC	SP1	0.001893526	path:00140_1

HUVEC	SP1	1.44E-05	path:00140_10
HUVEC	SP1	0.001534931	path:00140_11
HUVEC	SP1	0.006087664	path:00140_12
HUVEC	SP1	2.77E-05	path:00140_13
HUVEC	SP1	0.003885876	path:00140_14
HUVEC	SP1	0.000245716	path:00140_16
HUVEC	SP1	0.000245716	path:00140_18
HUVEC	SP1	2.77E-05	path:00140_19
HUVEC	SP1	0.000441307	path:00140_20
HUVEC	SP1	0.001893526	path:00140_25
HUVEC	SP1	1.44E-05	path:00140_3
HUVEC	SP1	1.44E-05	path:00140_4
HUVEC	SP1	5.07E-05	path:00140_5
HUVEC	SP1	8.66E-05	path:00140_6
HUVEC	SP1	2.77E-05	path:00140_7
HUVEC	SP1	0.000123921	path:00140_8
HUVEC	SP1	0.000123921	path:00140_9
HUVEC	SP1	0.005536058	path:00240_16
HUVEC	SP1	0.003391309	path:00240_17
HUVEC	SP1	0.001690362	path:00240_2
HUVEC	SP1	0.009002988	path:00240_5
HUVEC	SP1	0.005536058	path:00330_1
HUVEC	SP1	0.005126386	path:00330_9
HUVEC	SP1	0.006192277	path:00350_2
HUVEC	SP1	0.006192277	path:00350_3
HUVEC	SP1	0.001393247	path:00980_2
HUVEC	SP1	0.002175912	path:00982_10
HUVEC	SP1	0.003931316	path:04010_10
HUVEC	SP1	0.000441307	path:04010_26
HUVEC	SP1	0.000123921	path:04010_5
HUVEC	SP1	0.002149604	path:04060_22
HUVEC	SP1	0.001014956	path:04060_32
HUVEC	SP1	0.007433507	path:04060_47
HUVEC	SP1	0.00916136	path:04110_1

HUVEC	SP1	1.86E-10	path:04110_17
HUVEC	SP1	4.28E-11	path:04110_18
HUVEC	SP1	1.05E-10	path:04110_19
HUVEC	SP1	2.92E-09	path:04110_20
HUVEC	SP1	1.43E-05	path:04110_22
HUVEC	SP1	5.22E-09	path:04110_23
HUVEC	SP1	0.006192277	path:04110_25
HUVEC	SP1	7.70E-08	path:04110_26
HUVEC	SP1	2.92E-09	path:04110_27
HUVEC	SP1	6.46E-09	path:04110_3
HUVEC	SP1	6.46E-09	path:04110_4
HUVEC	SP1	4.97E-07	path:04110_7
HUVEC	SP1	3.47E-10	path:04115_1
HUVEC	SP1	6.74E-05	path:04115_2
HUVEC	SP1	4.23E-05	path:04115_3
HUVEC	SP1	6.18E-06	path:04115_4
HUVEC	SP1	4.96E-05	path:04115_7
HUVEC	SP1	7.61E-06	path:04144_2
HUVEC	SP1	0.002175912	path:04210_20
HUVEC	SP1	0.001107767	path:04210_24
HUVEC	SP1	1.33E-05	path:04310_6
HUVEC	SP1	1.77E-05	path:04310_7
HUVEC	SP1	7.85E-13	path:04510_1
HUVEC	SP1	1.05E-10	path:04510_10
HUVEC	SP1	0.001603207	path:04510_13
HUVEC	SP1	0.001164758	path:04510_15
HUVEC	SP1	0.002771235	path:04510_16
HUVEC	SP1	0.000141426	path:04510_17
HUVEC	SP1	8.07E-08	path:04510_18
HUVEC	SP1	1.96E-12	path:04510_19
HUVEC	SP1	1.43E-05	path:04510_20
HUVEC	SP1	0.000718519	path:04510_23
HUVEC	SP1	0.002175912	path:04510_24
HUVEC	SP1	0.0024986	path:04510_25

HUVEC	SP1	6.74E-05	path:04510_4
HUVEC	SP1	0.0024986	path:04510_5
HUVEC	SP1	1.07E-10	path:04510_6
HUVEC	SP1	0.000448433	path:04510_7
HUVEC	SP1	6.16E-10	path:04510_8
HUVEC	SP1	2.01E-05	path:04512_10
HUVEC	SP1	5.55E-06	path:04512_11
HUVEC	SP1	1.66E-06	path:04512_12
HUVEC	SP1	7.86E-06	path:04512_13
HUVEC	SP1	0.000101569	path:04512_14
HUVEC	SP1	0.000245716	path:04512_18
HUVEC	SP1	0.000549822	path:04512_19
HUVEC	SP1	0.002149604	path:04512_20
HUVEC	SP1	0.008608739	path:04512_22
HUVEC	SP1	0.00016592	path:04512_27
HUVEC	SP1	1.66E-05	path:04512_3
HUVEC	SP1	2.72E-07	path:04512_5
HUVEC	SP1	6.24E-07	path:04512_6
HUVEC	SP1	9.36E-06	path:04512_9
HUVEC	SP1	0.005126386	path:04520_3
HUVEC	SP1	0.001603207	path:04520_5
HUVEC	SP1	0.000412181	path:04520_7
HUVEC	SP1	1.22E-05	path:04540_13
HUVEC	SP1	9.21E-07	path:04610_3
HUVEC	SP1	5.55E-06	path:04610_4
HUVEC	SP1	9.21E-07	path:04610_6
HUVEC	SP1	0.000133832	path:04610_7
HUVEC	SP1	0.000203885	path:04610_8
HUVEC	SP1	0.000203885	path:04620_18
HUVEC	SP1	0.001893526	path:04620_9
HUVEC	SP1	0.000231547	path:04630_1
HUVEC	SP1	0.001390056	path:04630_2
HUVEC	SP1	1.97E-06	path:04630_3
HUVEC	SP1	5.95E-06	path:04630_4

HUVEC	SP1	4.84E-05	path:04650_20
HUVEC	SP1	0.008608739	path:04650_7
HUVEC	SP1	0.000718519	path:04722_19
HUVEC	SP1	7.92E-07	path:04810_29
HUVEC	SP1	1.33E-05	path:04912_8
HUVEC	SP1	0.007433507	path:04940_4
HUVEC	SP1	0.006087664	path:05014_3
HUVEC	SP1	0.008608739	path:05014_4
HUVEC	SP1	0.007433507	path:05016_8
HUVEC	SP1	0.001014956	path:05100_3
HUVEC	SP1	0.008608739	path:05140_10
HUVEC	SP1	0.000245716	path:05140_6
HUVEC	SP1	0.001107767	path:05142_10
HUVEC	SP1	0.003931316	path:05142_11
HUVEC	SP1	0.003885876	path:05142_17
HUVEC	SP1	0.003885876	path:05142_18
HUVEC	SP1	0.000441307	path:05142_20
HUVEC	SP1	0.000718519	path:05142_21
HUVEC	SP1	0.001107767	path:05142_4
HUVEC	SP1	0.006087664	path:05145_19
HUVEC	SP1	0.001014956	path:05145_21
HUVEC	SP1	0.006087664	path:05145_8
HUVEC	SP1	1.23E-06	path:05146_1
HUVEC	SP1	7.92E-07	path:05146_2
HUVEC	SP1	0.002149604	path:05146_5
HUVEC	SP1	9.10E-07	path:05146_9
HUVEC	SP1	0.003935354	path:05200_19
HUVEC	SP1	0.003935354	path:05200_20
HUVEC	SP1	0.00927462	path:05200_24
HUVEC	SP1	9.21E-07	path:05200_29
HUVEC	SP1	2.01E-05	path:05200_3
HUVEC	SP1	0.000718519	path:05200_31
HUVEC	SP1	0.008608739	path:05200_38
HUVEC	SP1	0.000289957	path:05200_4

HUVEC	SP1	0.000718519	path:05200_42
HUVEC	SP1	2.07E-08	path:05200_47
HUVEC	SP1	1.33E-05	path:05200_48
HUVEC	SP1	5.07E-05	path:05200_50
HUVEC	SP1	5.54E-09	path:05200_51
HUVEC	SP1	1.55E-08	path:05200_52
HUVEC	SP1	4.11E-06	path:05200_53
HUVEC	SP1	0.001014956	path:05200_56
HUVEC	SP1	0.000441307	path:05210_10
HUVEC	SP1	0.000718519	path:05210_12
HUVEC	SP1	0.000718519	path:05210_7
HUVEC	SP1	1.44E-05	path:05212_13
HUVEC	SP1	0.008608739	path:05212_14
HUVEC	SP1	1.33E-05	path:05214_12
HUVEC	SP1	1.18E-07	path:05214_13
HUVEC	SP1	2.07E-08	path:05214_14
HUVEC	SP1	9.21E-07	path:05214_15
HUVEC	SP1	0.005536058	path:05214_2
HUVEC	SP1	0.000245716	path:05214_21
HUVEC	SP1	0.005536058	path:05214_5
HUVEC	SP1	0.001164758	path:05215_10
HUVEC	SP1	0.008608739	path:05215_7
HUVEC	SP1	0.000441307	path:05216_4
HUVEC	SP1	0.000411461	path:05218_4
HUVEC	SP1	0.000133832	path:05218_5
HUVEC	SP1	1.33E-05	path:05218_6
HUVEC	SP1	9.21E-07	path:05218_7
HUVEC	SP1	7.27E-05	path:05218_8
HUVEC	SP1	0.000310459	path:05219_3
HUVEC	SP1	1.44E-05	path:05219_4
HUVEC	SP1	9.21E-07	path:05220_11
HUVEC	SP1	1.18E-07	path:05220_12
HUVEC	SP1	0.002149604	path:05220_5
HUVEC	SP1	1.33E-05	path:05220_8

HUVEC	SP1	2.07E-08	path:05220_9
HUVEC	SP1	0.006192277	path:05221_1
HUVEC	SP1	5.36E-07	path:05222_1
HUVEC	SP1	0.000133832	path:05222_2
HUVEC	SP1	2.01E-05	path:05222_4
HUVEC	SP1	0.001603207	path:05222_7
HUVEC	SP1	1.44E-05	path:05223_3
HUVEC	SP1	0.007433507	path:05320_9
HUVEC	SP1	0.007433507	path:05330_5
HUVEC	SP1	0.007433507	path:05332_2
HUVEC	SP1	0.007433507	path:05332_5
HUVEC	SP1	1.77E-05	path:05414_2
intestine	AKNA	6.20E-07	path:04060_31
intestine	AKNA	6.20E-07	path:04514_10
intestine	AKNA	6.20E-07	path:04514_11
intestine	AKNA	6.20E-07	path:04514_12
intestine	AKNA	6.20E-07	path:04514_78
intestine	AKNA	6.20E-07	path:04514_9
intestine	AKNA	6.20E-07	path:04672_2
intestine	AKNA	6.20E-07	path:05144_9
intestine	AKNA	6.20E-07	path:05145_20
intestine	AKNA	6.20E-07	path:05310_2
intestine	AKNA	6.20E-07	path:05320_2
intestine	AKNA	6.20E-07	path:05320_5
intestine	AKNA	6.20E-07	path:05322_7
intestine	AKNA	6.20E-07	path:05330_12
intestine	AKNA	6.20E-07	path:05330_3
intestine	AKNA	6.20E-07	path:05416_3
intestine	HNF1A	0.008512637	path:00120_5
intestine	HNF1A	0.008512637	path:00140_1
intestine	HNF1A	3.95E-05	path:00140_12
intestine	HNF1A	0.008512637	path:00140_22
intestine	HNF1A	0.008512637	path:00140_23
intestine	HNF1A	0.008512637	path:00140_24

intestine	HNF1A	0.008512637	path:00140_25
intestine	HNF1A	0.008512637	path:00140_26
intestine	HNF1A	0.008512637	path:00982_6
intestine	HNF1A	0.008512637	path:04610_6
intestine	HNF4A	0.00730316	path:00120_15
intestine	HNF4A	0.003812707	path:00120_19
intestine	HNF4A	0.001698537	path:00120_6
intestine	HNF4A	0.003812707	path:00120_7
intestine	HNF4A	0.000100826	path:00120_9
intestine	HNF4A	0.001808381	path:00590_1
intestine	HNF4A	0.001722691	path:00590_2
intestine	HNF4A	0.001722691	path:00590_3
intestine	HNF4A	0.001898001	path:00590_4
intestine	HNF4A	0.000515682	path:00591_1
intestine	HNF4A	2.36E-05	path:00830_2
intestine	HNF4A	0.000733563	path:00980_2
intestine	HNF4A	2.52E-07	path:00982_10
intestine	HNF4A	5.38E-08	path:00982_4
intestine	HNF4A	5.38E-08	path:00982_5
intestine	HNF4A	5.38E-08	path:00982_9
intestine	HNF4A	0.001131883	path:03320_1
intestine	HNF4A	0.000100826	path:03320_2
intestine	HNF4A	4.35E-06	path:04610_2
intestine	HNF4A	4.35E-06	path:04610_3
intestine	HNF4A	0.000100826	path:04610_4
intestine	HNF4A	6.69E-06	path:04610_5
intestine	HNF4A	0.000179282	path:04610_6
intestine	HNF4A	0.001698537	path:04610_8
intestine	HNF4A	0.000287791	path:04950_4
intestine	TP53	0.007702858	path:04010_15
intestine	TP53	0.009062653	path:04010_16
intestine	TP53	0.007443002	path:04010_21
intestine	TP53	0.002466645	path:04012_12
intestine	TP53	2.55E-11	path:04110_1

intestine	TP53	3.28E-07	path:04110_17
intestine	TP53	1.48E-05	path:04110_19
intestine	TP53	0.001943417	path:04110_20
intestine	TP53	7.56E-05	path:04110_22
intestine	TP53	0.000198814	path:04110_23
intestine	TP53	0.001943417	path:04110_27
intestine	TP53	0.000210553	path:04110_3
intestine	TP53	0.004511216	path:04110_4
intestine	TP53	4.42E-09	path:04110_7
intestine	TP53	0.004291125	path:04110_8
intestine	TP53	0	path:04115_1
intestine	TP53	0	path:04115_2
intestine	TP53	0	path:04115_3
intestine	TP53	0	path:04115_4
intestine	TP53	0	path:04115_7
intestine	TP53	0.000421718	path:04210_20
intestine	TP53	0.000275763	path:04210_23
intestine	TP53	0.000275763	path:04210_24
intestine	TP53	0.009131774	path:04510_10
intestine	TP53	0.004511216	path:04510_15
intestine	TP53	0.002466645	path:04510_16
intestine	TP53	0.000580605	path:04510_17
intestine	TP53	0.001774645	path:04510_18
intestine	TP53	0.006951959	path:04510_25
intestine	TP53	0.006951959	path:04510_5
intestine	TP53	0.009576653	path:04510_6
intestine	TP53	0.001110697	path:04510_7
intestine	TP53	0.00935253	path:04510_8
intestine	TP53	0.009062653	path:04621_7
intestine	TP53	0.000218217	path:04722_19
intestine	TP53	0.002756828	path:04912_8
intestine	TP53	0.009062653	path:05014_4
intestine	TP53	0.007459365	path:05160_12
intestine	TP53	0.009062653	path:05160_15

intestine	TP53	0.000338058	path:05200_10
intestine	TP53	0.000275763	path:05200_11
intestine	TP53	0.000275763	path:05200_12
intestine	TP53	0.000968321	path:05200_15
intestine	TP53	0.00392722	path:05200_17
intestine	TP53	0.002997803	path:05200_19
intestine	TP53	0.00011707	path:05200_20
intestine	TP53	0.000275763	path:05200_4
intestine	TP53	7.56E-05	path:05200_47
intestine	TP53	1.95E-05	path:05200_48
intestine	TP53	0.000367024	path:05200_5
intestine	TP53	0.000172314	path:05200_51
intestine	TP53	1.95E-05	path:05200_8
intestine	TP53	0.007459365	path:05213_2
intestine	TP53	0.003580594	path:05214_1
intestine	TP53	0.000210553	path:05214_10
intestine	TP53	0.001774645	path:05214_11
intestine	TP53	1.95E-05	path:05214_12
intestine	TP53	0.00011707	path:05214_13
intestine	TP53	7.56E-05	path:05214_14
intestine	TP53	0.003580594	path:05214_4
intestine	TP53	0.004511216	path:05215_10
intestine	TP53	0.000275763	path:05215_11
intestine	TP53	0.00331496	path:05215_3
intestine	TP53	0.002754132	path:05218_2
intestine	TP53	0.001774645	path:05218_5
intestine	TP53	1.95E-05	path:05218_6
intestine	TP53	0.001385048	path:05218_8
intestine	TP53	0.001691972	path:05219_3
intestine	TP53	0.009062653	path:05219_4
intestine	TP53	0.00011707	path:05220_12
intestine	TP53	1.95E-05	path:05220_8
intestine	TP53	7.56E-05	path:05220_9
Jurkat	KLF4	4.70E-05	path:04110_17

Jurkat	KLF4	4.70E-05	path:04110_19
Jurkat	KLF4	0.000691257	path:04110_20
Jurkat	KLF4	0.001048885	path:04110_23
Jurkat	KLF4	0.001048885	path:04110_26
Jurkat	KLF4	0.000691257	path:04110_27
Jurkat	KLF4	0.001048885	path:04110_3
Jurkat	KLF4	0.001048885	path:04110_4
Jurkat	KLF4	0.001498093	path:04110_7
Jurkat	KLF4	0.003364391	path:04115_1
Jurkat	KLF4	0.007702669	path:04512_1
Jurkat	KLF4	0.007702669	path:04512_2
Jurkat	KLF4	0.007140402	path:04512_4
Jurkat	KLF4	0.007140402	path:04512_7
Jurkat	KLF4	0.007140402	path:04512_8
Jurkat	KLF4	0.007140402	path:05145_14
Jurkat	KLF4	0.003572583	path:05200_31
Jurkat	KLF4	0.002858346	path:05200_47
Jurkat	KLF4	0.003572583	path:05200_51
Jurkat	KLF4	0.007103944	path:05200_52
Jurkat	KLF4	0.005224296	path:05200_53
Jurkat	KLF4	0.003572583	path:05210_12
Jurkat	KLF4	0.003572583	path:05210_7
Jurkat	KLF4	0.003364391	path:05214_13
Jurkat	KLF4	0.002858346	path:05214_14
Jurkat	KLF4	0.003572583	path:05214_15
Jurkat	KLF4	0.003572583	path:05218_7
Jurkat	KLF4	0.003572583	path:05219_4
Jurkat	KLF4	0.003572583	path:05220_11
Jurkat	KLF4	0.003364391	path:05220_12
Jurkat	KLF4	0.002858346	path:05220_9
Jurkat	RELA	0.000137451	path:04060_62
Jurkat	RELA	2.04E-07	path:04062_1
Jurkat	RELA	3.87E-08	path:04062_2
Jurkat	RELA	0.001869278	path:04110_17

Jurkat	RELA	0.001781655	path:04110_19
Jurkat	RELA	0.001869278	path:04110_7
Jurkat	RELA	0.0005539	path:04115_1
Jurkat	RELA	2.11E-06	path:04620_1
Jurkat	RELA	4.80E-09	path:04620_12
Jurkat	RELA	1.51E-07	path:04620_13
Jurkat	RELA	2.07E-07	path:04620_14
Jurkat	RELA	1.03E-08	path:04620_16
Jurkat	RELA	6.51E-08	path:04620_17
Jurkat	RELA	8.85E-09	path:04620_18
Jurkat	RELA	6.51E-08	path:04620_22
Jurkat	RELA	7.78E-08	path:04620_9
Jurkat	RELA	0.00197363	path:04650_22
Jurkat	RELA	0.001781655	path:04650_23
K562	EGR1	0.009869535	path:04060_8
K562	EGR1	0.00069989	path:04510_10
K562	EGR1	0.00069989	path:04510_18
K562	EGR1	0.003153788	path:04510_6
K562	EGR1	0.003153788	path:04510_8
K562	EGR1	0.00129686	path:04512_3
K562	EGR1	0.003153788	path:04540_13
K562	EGR1	0.003153788	path:05146_9
K562	EGR1	0.007008495	path:05212_11
K562	EGR1	0.008365691	path:05212_12
K562	EGR1	0.003153788	path:05218_4
K562	FOS	0.005719136	path:04115_1
K562	FOS	0.006308233	path:04310_7
K562	FOS	0.003173805	path:04610_7
K562	FOS	0.006237239	path:04620_17
K562	FOS	0.003173805	path:04620_18
K562	FOS	0.006237239	path:04620_22
K562	FOS	0.006237239	path:04620_9
K562	FOS	2.16E-08	path:05200_29
K562	NFE2L2	0.003129559	path:00480_1

K562	NFE2L2	2.02E-06	path:00480_2
K562	NFE2L2	0.003129559	path:00480_3
K562	NFE2L2	0.003129559	path:00480_4
K562	NFE2L2	0.003129559	path:00480_5
K562	NFE2L2	0.003129559	path:00480_6
K562	RELA	0.000137451	path:04060_62
K562	RELA	2.04E-07	path:04062_1
K562	RELA	3.87E-08	path:04062_2
K562	RELA	0.001869278	path:04110_17
K562	RELA	0.001781655	path:04110_19
K562	RELA	0.001869278	path:04110_7
K562	RELA	0.0005539	path:04115_1
K562	RELA	2.11E-06	path:04620_1
K562	RELA	4.80E-09	path:04620_12
K562	RELA	1.51E-07	path:04620_13
K562	RELA	2.07E-07	path:04620_14
K562	RELA	1.03E-08	path:04620_16
K562	RELA	6.51E-08	path:04620_17
K562	RELA	8.85E-09	path:04620_18
K562	RELA	6.51E-08	path:04620_22
K562	RELA	7.78E-08	path:04620_9
K562	RELA	0.00197363	path:04650_22
K562	RELA	0.001781655	path:04650_23
Keratinocyte	CEBPA	0.0042174	path:04060_54
Keratinocyte	CEBPA	0.00918123	path:04610_3
Keratinocyte	CEBPA	0.00018207	path:04610_4
Keratinocyte	CEBPA	0.00018207	path:04610_6
Keratinocyte	TFAP2A	0.005723968	path:04020_2
Keratinocyte	TFAP2A	0.003857334	path:04080_7
Keratinocyte	TFAP2A	0.00057548	path:04144_2
Keratinocyte	TFAP2A	0.001269093	path:04510_10
Keratinocyte	TFAP2A	0.005458314	path:04510_15
Keratinocyte	TFAP2A	0.003332624	path:04510_16
Keratinocyte	TFAP2A	3.81E-05	path:04510_17

Keratinocyte	TFAP2A	1.42E-05	path:04510_18
Keratinocyte	TFAP2A	0.007739985	path:04510_25
Keratinocyte	TFAP2A	0.007739985	path:04510_5
Keratinocyte	TFAP2A	0.001276164	path:04510_6
Keratinocyte	TFAP2A	8.35E-05	path:04510_7
Keratinocyte	TFAP2A	0.001271633	path:04510_8
Keratinocyte	TFAP2A	0.002407854	path:04520_2
Keratinocyte	TFAP2A	0.001262516	path:04520_3
Keratinocyte	TFAP2A	0.00057548	path:04520_5
Keratinocyte	TFAP2A	0.001262516	path:04520_7
Keratinocyte	TFAP2A	0.009046641	path:04520_9
Keratinocyte	TFAP2A	0.003857334	path:04912_8
Keratinocyte	TFAP2A	2.20E-05	path:05200_10
Keratinocyte	TFAP2A	1.91E-05	path:05200_11
Keratinocyte	TFAP2A	1.91E-05	path:05200_12
Keratinocyte	TFAP2A	6.62E-06	path:05200_15
Keratinocyte	TFAP2A	0.000448647	path:05200_21
Keratinocyte	TFAP2A	8.35E-05	path:05200_29
Keratinocyte	TFAP2A	1.91E-05	path:05200_4
Keratinocyte	TFAP2A	2.22E-05	path:05200_5
Keratinocyte	TFAP2A	2.16E-06	path:05200_8
Keratinocyte	TFAP2A	0.002699451	path:05212_11
Keratinocyte	TFAP2A	0.003332624	path:05212_12
Keratinocyte	TFAP2A	0.004058678	path:05214_1
Keratinocyte	TFAP2A	0.000317116	path:05214_10
Keratinocyte	TFAP2A	0.002407854	path:05214_11
Keratinocyte	TFAP2A	0.007739985	path:05214_16
Keratinocyte	TFAP2A	0.004058678	path:05214_17
Keratinocyte	TFAP2A	0.004058678	path:05214_2
Keratinocyte	TFAP2A	0.004058678	path:05214_3
Keratinocyte	TFAP2A	0.004058678	path:05214_4
Keratinocyte	TFAP2A	0.004058678	path:05214_5
Keratinocyte	TFAP2A	0.004058678	path:05214_6
Keratinocyte	TFAP2A	0.007739985	path:05214_7

Keratinocyte	TFAP2A	0.004058678	path:05214_8
Keratinocyte	TFAP2A	0.000317116	path:05215_10
Keratinocyte	TFAP2A	0.000436788	path:05215_11
Keratinocyte	TFAP2A	0.001124594	path:05215_8
Keratinocyte	TFAP2A	0.002407854	path:05219_2
Keratinocyte	TP63	2.23E-05	path:04110_1
Keratinocyte	TP63	8.12E-05	path:04110_7
Keratinocyte	TP63	8.12E-05	path:04115_1
Keratinocyte	TP63	8.12E-05	path:04115_2
Keratinocyte	TP63	8.12E-05	path:04115_3
Keratinocyte	TP63	8.12E-05	path:04115_4
Keratinocyte	TP63	8.12E-05	path:04115_7
Keratinocyte	TP63	0.003115907	path:05200_17
Keratinocyte	TP63	0.002783081	path:05200_19
Keratinocyte	TP63	0.002783081	path:05200_20
Keratinocyte	TP63	0.000159403	path:05200_47
Keratinocyte	TP63	8.12E-05	path:05200_48
Keratinocyte	TP63	8.12E-05	path:05214_12
Keratinocyte	TP63	0.000159403	path:05214_14
Keratinocyte	TP63	0.004120782	path:05215_11
Keratinocyte	TP63	0.002837304	path:05215_3
Keratinocyte	TP63	8.12E-05	path:05218_6
Keratinocyte	TP63	8.12E-05	path:05220_8
Keratinocyte	TP63	0.000159403	path:05220_9
kidney	CREB1	0.004288489	path:04514_1
kidney	CREB1	0.004288489	path:04514_3
kidney	CREB1	0.004288489	path:04612_3
kidney	CREB1	0.004131401	path:04620_12
kidney	CREB1	0.004131401	path:04620_16
kidney	CREB1	0.001277819	path:04620_17
kidney	CREB1	0.000627719	path:04620_18
kidney	CREB1	0.001277819	path:04620_22
kidney	CREB1	0.001277819	path:04620_9
kidney	CREB1	0.004131401	path:04672_4

kidney	CREB1	0.004131401	path:04672_5
kidney	CREB1	0.004131401	path:04940_1
kidney	CREB1	0.004288489	path:05150_14
kidney	CREB1	0.009649083	path:05200_18
kidney	CREB1	0.004131401	path:05310_1
kidney	CREB1	0.004131401	path:05310_3
kidney	CREB1	0.004131401	path:05320_1
kidney	CREB1	0.004131401	path:05320_3
kidney	CREB1	0.004131401	path:05320_4
kidney	CREB1	0.004131401	path:05320_6
kidney	CREB1	0.004131401	path:05322_5
kidney	CREB1	0.004131401	path:05322_6
kidney	CREB1	0.004131401	path:05330_1
kidney	CREB1	0.004131401	path:05330_2
kidney	CREB1	0.004131401	path:05330_4
kidney	CREB1	0.004131401	path:05332_1
kidney	CREB1	0.004131401	path:05416_4
kidney	HNF1A	0.008512637	path:00120_5
kidney	HNF1A	0.008512637	path:00140_1
kidney	HNF1A	3.95E-05	path:00140_12
kidney	HNF1A	0.008512637	path:00140_22
kidney	HNF1A	0.008512637	path:00140_23
kidney	HNF1A	0.008512637	path:00140_24
kidney	HNF1A	0.008512637	path:00140_25
kidney	HNF1A	0.008512637	path:00140_26
kidney	HNF1A	0.008512637	path:00982_6
kidney	HNF1A	0.008512637	path:04610_6
kidney	HNF4A	0.00730316	path:00120_15
kidney	HNF4A	0.003812707	path:00120_19
kidney	HNF4A	0.001698537	path:00120_6
kidney	HNF4A	0.003812707	path:00120_7
kidney	HNF4A	0.000100826	path:00120_9
kidney	HNF4A	0.001808381	path:00590_1
kidney	HNF4A	0.001722691	path:00590_2

kidney	HNF4A	0.001722691	path:00590_3
kidney	HNF4A	0.001898001	path:00590_4
kidney	HNF4A	0.000515682	path:00591_1
kidney	HNF4A	2.36E-05	path:00830_2
kidney	HNF4A	0.000733563	path:00980_2
kidney	HNF4A	2.52E-07	path:00982_10
kidney	HNF4A	5.38E-08	path:00982_4
kidney	HNF4A	5.38E-08	path:00982_5
kidney	HNF4A	5.38E-08	path:00982_9
kidney	HNF4A	0.001131883	path:03320_1
kidney	HNF4A	0.000100826	path:03320_2
kidney	HNF4A	4.35E-06	path:04610_2
kidney	HNF4A	4.35E-06	path:04610_3
kidney	HNF4A	0.000100826	path:04610_4
kidney	HNF4A	6.69E-06	path:04610_5
kidney	HNF4A	0.000179282	path:04610_6
kidney	HNF4A	0.001698537	path:04610_8
kidney	HNF4A	0.000287791	path:04950_4
kidney	NR1I3	0.000186607	path:00830_2
kidney	NR1I3	0.006275482	path:00980_1
kidney	NR1I3	0.000535628	path:00982_4
kidney	NR1I3	0.000535628	path:00982_5
kidney	NR1I3	0.000535628	path:00982_9
kidney	SREBF1	0.009882974	path:00100_9
kidney	SREBF1	0.009882974	path:03320_2
kidney	SREBF1	3.27E-05	path:04910_5
kidney	SREBF1	0.00143191	path:04910_6
leukemia	SPI1	0.00153244	path:04060_41
leukemia	SPI1	0.005348786	path:04145_2
leukemia	SPI1	3.11E-06	path:04145_6
leukemia	SPI1	0.007630608	path:04514_55
leukemia	SPI1	0.003570772	path:04514_62
leukemia	SPI1	0.008430928	path:04650_10
leukemia	SPI1	0.007340609	path:04650_12

leukemia	SPI1	0.003805584	path:04650_13
leukemia	SPI1	0.003570772	path:04670_10
leukemia	SPI1	0.00153244	path:04670_14
leukemia	SPI1	0.000961365	path:04670_15
leukemia	SPI1	0.000252274	path:04670_2
leukemia	SPI1	0.000110411	path:05140_4
leukemia	SPI1	0.005348786	path:05150_17
leukemia	SPI1	0.000902646	path:05200_43
leukemia	TP73	6.75E-05	path:04115_1
leukemia	TP73	6.67E-05	path:04115_2
leukemia	TP73	6.67E-05	path:04115_3
leukemia	TP73	6.67E-05	path:04115_4
leukemia	TP73	6.67E-05	path:04115_7
leukemia	TP73	0.000595015	path:04722_19
liver	AKNA	6.20E-07	path:04060_31
liver	AKNA	6.20E-07	path:04514_10
liver	AKNA	6.20E-07	path:04514_11
liver	AKNA	6.20E-07	path:04514_12
liver	AKNA	6.20E-07	path:04514_78
liver	AKNA	6.20E-07	path:04514_9
liver	AKNA	6.20E-07	path:04672_2
liver	AKNA	6.20E-07	path:05144_9
liver	AKNA	6.20E-07	path:05145_20
liver	AKNA	6.20E-07	path:05310_2
liver	AKNA	6.20E-07	path:05320_2
liver	AKNA	6.20E-07	path:05320_5
liver	AKNA	6.20E-07	path:05322_7
liver	AKNA	6.20E-07	path:05330_12
liver	AKNA	6.20E-07	path:05330_3
liver	AKNA	6.20E-07	path:05416_3
liver	CREB1	0.004288489	path:04514_1
liver	CREB1	0.004288489	path:04514_3
liver	CREB1	0.004288489	path:04612_3
liver	CREB1	0.004131401	path:04620_12

liver	CREB1	0.004131401	path:04620_16
liver	CREB1	0.001277819	path:04620_17
liver	CREB1	0.000627719	path:04620_18
liver	CREB1	0.001277819	path:04620_22
liver	CREB1	0.001277819	path:04620_9
liver	CREB1	0.004131401	path:04672_4
liver	CREB1	0.004131401	path:04672_5
liver	CREB1	0.004131401	path:04940_1
liver	CREB1	0.004288489	path:05150_14
liver	CREB1	0.009649083	path:05200_18
liver	CREB1	0.004131401	path:05310_1
liver	CREB1	0.004131401	path:05310_3
liver	CREB1	0.004131401	path:05320_1
liver	CREB1	0.004131401	path:05320_3
liver	CREB1	0.004131401	path:05320_4
liver	CREB1	0.004131401	path:05320_6
liver	CREB1	0.004131401	path:05322_5
liver	CREB1	0.004131401	path:05322_6
liver	CREB1	0.004131401	path:05330_1
liver	CREB1	0.004131401	path:05330_2
liver	CREB1	0.004131401	path:05330_4
liver	CREB1	0.004131401	path:05332_1
liver	CREB1	0.004131401	path:05416_4
liver	FOXA2	0.000581017	path:04610_3
liver	FOXA2	0.000581017	path:04610_4
liver	FOXA2	0.000581017	path:04610_6
liver	FOXA3	0.000327469	path:00140_1
liver	FOXA3	0.000188543	path:00140_11
liver	FOXA3	0.000502586	path:00140_12
liver	FOXA3	0.000327469	path:00140_25
liver	FOXA3	0.000327469	path:00140_26
liver	FOXA3	0.000312584	path:00591_1
liver	FOXA3	0.000975791	path:00830_2
liver	FOXA3	0.000327469	path:00980_2

liver	FOXA3	7.28E-05	path:00982_10
liver	FOXA3	2.48E-05	path:00982_11
liver	FOXA3	5.58E-05	path:00982_2
liver	FOXA3	2.48E-05	path:00982_3
liver	FOXA3	5.10E-05	path:00982_4
liver	FOXA3	5.10E-05	path:00982_5
liver	FOXA3	0.000248984	path:00982_6
liver	FOXA3	2.48E-05	path:00982_7
liver	FOXA3	2.48E-05	path:00982_8
liver	FOXA3	5.10E-05	path:00982_9
liver	HNF1A	0.008512637	path:00120_5
liver	HNF1A	0.008512637	path:00140_1
liver	HNF1A	3.95E-05	path:00140_12
liver	HNF1A	0.008512637	path:00140_22
liver	HNF1A	0.008512637	path:00140_23
liver	HNF1A	0.008512637	path:00140_24
liver	HNF1A	0.008512637	path:00140_25
liver	HNF1A	0.008512637	path:00140_26
liver	HNF1A	0.008512637	path:00982_6
liver	HNF1A	0.008512637	path:04610_6
liver	HNF4A	0.00730316	path:00120_15
liver	HNF4A	0.003812707	path:00120_19
liver	HNF4A	0.001698537	path:00120_6
liver	HNF4A	0.003812707	path:00120_7
liver	HNF4A	0.000100826	path:00120_9
liver	HNF4A	0.001808381	path:00590_1
liver	HNF4A	0.001722691	path:00590_2
liver	HNF4A	0.001722691	path:00590_3
liver	HNF4A	0.001898001	path:00590_4
liver	HNF4A	0.000515682	path:00591_1
liver	HNF4A	2.36E-05	path:00830_2
liver	HNF4A	0.000733563	path:00980_2
liver	HNF4A	2.52E-07	path:00982_10
liver	HNF4A	5.38E-08	path:00982_4

liver	HNF4A	5.38E-08	path:00982_5
liver	HNF4A	5.38E-08	path:00982_9
liver	HNF4A	0.001131883	path:03320_1
liver	HNF4A	0.000100826	path:03320_2
liver	HNF4A	4.35E-06	path:04610_2
liver	HNF4A	4.35E-06	path:04610_3
liver	HNF4A	0.000100826	path:04610_4
liver	HNF4A	6.69E-06	path:04610_5
liver	HNF4A	0.000179282	path:04610_6
liver	HNF4A	0.001698537	path:04610_8
liver	HNF4A	0.000287791	path:04950_4
liver	NR1I2	0.004391058	path:00830_2
liver	NR1I2	0.00011908	path:00982_4
liver	NR1I2	0.00011908	path:00982_5
liver	NR1I2	0.00011908	path:00982_9
liver	NR1I3	0.000186607	path:00830_2
liver	NR1I3	0.006275482	path:00980_1
liver	NR1I3	0.000535628	path:00982_4
liver	NR1I3	0.000535628	path:00982_5
liver	NR1I3	0.000535628	path:00982_9
liver	NR4A1	0.00077713	path:00140_10
liver	NR4A1	6.42E-06	path:00140_13
liver	NR4A1	0.00077713	path:00140_14
liver	NR4A1	0.00077713	path:00140_16
liver	NR4A1	0.00077713	path:00140_18
liver	NR4A1	0.000832486	path:00140_19
liver	NR4A1	0.00077713	path:00140_20
liver	NR4A1	0.00077713	path:00140_3
liver	NR4A1	0.00077713	path:00140_4
liver	NR4A1	0.000960385	path:00140_5
liver	NR4A1	0.001089761	path:00140_6
liver	NR4A1	0.000832486	path:00140_7
liver	NR4A1	0.00077713	path:00140_8
liver	NR4A1	0.00077713	path:00140_9

liver	SPI1	0.00153244	path:04060_41
liver	SPI1	0.005348786	path:04145_2
liver	SPI1	3.11E-06	path:04145_6
liver	SPI1	0.007630608	path:04514_55
liver	SPI1	0.003570772	path:04514_62
liver	SPI1	0.008430928	path:04650_10
liver	SPI1	0.007340609	path:04650_12
liver	SPI1	0.003805584	path:04650_13
liver	SPI1	0.003570772	path:04670_10
liver	SPI1	0.00153244	path:04670_14
liver	SPI1	0.000961365	path:04670_15
liver	SPI1	0.000252274	path:04670_2
liver	SPI1	0.000110411	path:05140_4
liver	SPI1	0.005348786	path:05150_17
liver	SPI1	0.000902646	path:05200_43
liver	SREBF1	0.009882974	path:00100_9
liver	SREBF1	0.009882974	path:03320_2
liver	SREBF1	3.27E-05	path:04910_5
liver	SREBF1	0.00143191	path:04910_6
LNCaP	NFIC	0.004396514	path:00591_1
LNCaP	NFIC	0.005988829	path:00982_11
LNCaP	NFIC	0.00921451	path:00982_2
LNCaP	NFIC	0.005988829	path:00982_3
LNCaP	NFIC	0.00787147	path:00982_4
LNCaP	NFIC	0.00787147	path:00982_5
LNCaP	NFIC	0.004396514	path:00982_7
LNCaP	NFIC	0.004396514	path:00982_8
LNCaP	NFIC	0.00787147	path:00982_9
LNCaP	NFIC	0.00787147	path:04010_30
LNCaP	NFIC	0.00787147	path:04010_31
LNCaP	NFIC	0.004396514	path:04080_7
LNCaP	NFIC	0.008000492	path:04115_1
LNCaP	NFIC	0.00787147	path:04115_2
LNCaP	NFIC	0.00787147	path:04115_3

LNCaP	NFIC	0.00787147	path:04115_4
LNCaP	NFIC	0.00787147	path:04115_7
LNCaP	NFIC	0.00787147	path:04660_8
LNCaP	NFIC	0.00787147	path:04662_6
LNCaP	NFIC	0.00787147	path:04722_19
LNCaP	NFIC	0.00787147	path:05160_15
LNCaP	NFIC	0.007050709	path:05200_47
LNCaP	NFIC	0.004396514	path:05200_48
LNCaP	NFIC	0.00787147	path:05200_51
LNCaP	NFIC	0.004396514	path:05214_12
LNCaP	NFIC	0.00787147	path:05214_13
LNCaP	NFIC	0.007050709	path:05214_14
LNCaP	NFIC	0.004396514	path:05218_5
LNCaP	NFIC	0.004396514	path:05218_6
LNCaP	NFIC	0.00787147	path:05220_12
LNCaP	NFIC	0.004396514	path:05220_8
LNCaP	NFIC	0.007050709	path:05220_9
lung	NR1I3	0.000186607	path:00830_2
lung	NR1I3	0.006275482	path:00980_1
lung	NR1I3	0.000535628	path:00982_4
lung	NR1I3	0.000535628	path:00982_5
lung	NR1I3	0.000535628	path:00982_9
lung	SREBF1	0.009882974	path:00100_9
lung	SREBF1	0.009882974	path:03320_2
lung	SREBF1	3.27E-05	path:04910_5
lung	SREBF1	0.00143191	path:04910_6
lung	TP63	2.23E-05	path:04110_1
lung	TP63	8.12E-05	path:04110_7
lung	TP63	8.12E-05	path:04115_1
lung	TP63	8.12E-05	path:04115_2
lung	TP63	8.12E-05	path:04115_3
lung	TP63	8.12E-05	path:04115_4
lung	TP63	8.12E-05	path:04115_7
lung	TP63	0.003115907	path:05200_17

lung	TP63	0.002783081	path:05200_19
lung	TP63	0.002783081	path:05200_20
lung	TP63	0.000159403	path:05200_47
lung	TP63	8.12E-05	path:05200_48
lung	TP63	8.12E-05	path:05214_12
lung	TP63	0.000159403	path:05214_14
lung	TP63	0.004120782	path:05215_11
lung	TP63	0.002837304	path:05215_3
lung	TP63	8.12E-05	path:05218_6
lung	TP63	8.12E-05	path:05220_8
lung	TP63	0.000159403	path:05220_9
lymph node	AKNA	6.20E-07	path:04060_31
lymph node	AKNA	6.20E-07	path:04514_10
lymph node	AKNA	6.20E-07	path:04514_11
lymph node	AKNA	6.20E-07	path:04514_12
lymph node	AKNA	6.20E-07	path:04514_78
lymph node	AKNA	6.20E-07	path:04514_9
lymph node	AKNA	6.20E-07	path:04672_2
lymph node	AKNA	6.20E-07	path:05144_9
lymph node	AKNA	6.20E-07	path:05145_20
lymph node	AKNA	6.20E-07	path:05310_2
lymph node	AKNA	6.20E-07	path:05320_2
lymph node	AKNA	6.20E-07	path:05320_5
lymph node	AKNA	6.20E-07	path:05322_7
lymph node	AKNA	6.20E-07	path:05330_12
lymph node	AKNA	6.20E-07	path:05330_3
lymph node	AKNA	6.20E-07	path:05416_3
lymph node	IRF5	0.005011517	path:04060_38
lymph node	IRF5	0.005011517	path:04620_11
lymph node	IRF5	0.005011517	path:04620_2
lymph node	IRF5	0.005011517	path:04650_6
lymph node	IRF5	0.005011517	path:05160_6
lymph node	IRF7	5.44E-05	path:04060_38
lymph node	IRF7	3.65E-07	path:04620_11

lymph node	IRF7	1.24E-05	path:04620_12
lymph node	IRF7	0.005131788	path:04620_14
lymph node	IRF7	0.005500251	path:04620_16
lymph node	IRF7	0.009380523	path:04620_17
lymph node	IRF7	0.005305274	path:04620_18
lymph node	IRF7	3.65E-07	path:04620_2
lymph node	IRF7	0.009380523	path:04620_22
lymph node	IRF7	0.009501335	path:04620_9
lymph node	IRF7	5.75E-05	path:04650_6
lymph node	IRF7	5.75E-05	path:05160_6
lymph node	TP73	6.75E-05	path:04115_1
lymph node	TP73	6.67E-05	path:04115_2
lymph node	TP73	6.67E-05	path:04115_3
lymph node	TP73	6.67E-05	path:04115_4
lymph node	TP73	6.67E-05	path:04115_7
lymph node	TP73	0.000595015	path:04722_19
lymphocytes	AKNA	6.20E-07	path:04060_31
lymphocytes	AKNA	6.20E-07	path:04514_10
lymphocytes	AKNA	6.20E-07	path:04514_11
lymphocytes	AKNA	6.20E-07	path:04514_12
lymphocytes	AKNA	6.20E-07	path:04514_78
lymphocytes	AKNA	6.20E-07	path:04514_9
lymphocytes	AKNA	6.20E-07	path:04672_2
lymphocytes	AKNA	6.20E-07	path:05144_9
lymphocytes	AKNA	6.20E-07	path:05145_20
lymphocytes	AKNA	6.20E-07	path:05310_2
lymphocytes	AKNA	6.20E-07	path:05320_2
lymphocytes	AKNA	6.20E-07	path:05320_5
lymphocytes	AKNA	6.20E-07	path:05322_7
lymphocytes	AKNA	6.20E-07	path:05330_12
lymphocytes	AKNA	6.20E-07	path:05330_3
lymphocytes	AKNA	6.20E-07	path:05416_3
lymphocytes	CREB1	0.004288489	path:04514_1
lymphocytes	CREB1	0.004288489	path:04514_3

lymphocytes	CREB1	0.004288489	path:04612_3
lymphocytes	CREB1	0.004131401	path:04620_12
lymphocytes	CREB1	0.004131401	path:04620_16
lymphocytes	CREB1	0.001277819	path:04620_17
lymphocytes	CREB1	0.000627719	path:04620_18
lymphocytes	CREB1	0.001277819	path:04620_22
lymphocytes	CREB1	0.001277819	path:04620_9
lymphocytes	CREB1	0.004131401	path:04672_4
lymphocytes	CREB1	0.004131401	path:04672_5
lymphocytes	CREB1	0.004131401	path:04940_1
lymphocytes	CREB1	0.004288489	path:05150_14
lymphocytes	CREB1	0.009649083	path:05200_18
lymphocytes	CREB1	0.004131401	path:05310_1
lymphocytes	CREB1	0.004131401	path:05310_3
lymphocytes	CREB1	0.004131401	path:05320_1
lymphocytes	CREB1	0.004131401	path:05320_3
lymphocytes	CREB1	0.004131401	path:05320_4
lymphocytes	CREB1	0.004131401	path:05320_6
lymphocytes	CREB1	0.004131401	path:05322_5
lymphocytes	CREB1	0.004131401	path:05322_6
lymphocytes	CREB1	0.004131401	path:05330_1
lymphocytes	CREB1	0.004131401	path:05330_2
lymphocytes	CREB1	0.004131401	path:05330_4
lymphocytes	CREB1	0.004131401	path:05332_1
lymphocytes	CREB1	0.004131401	path:05416_4
lymphocytes	NFYA	0.00144529	path:04110_1
lymphocytes	NFYA	0.005383343	path:04110_11
lymphocytes	NFYA	0.003794515	path:04110_12
lymphocytes	NFYA	0.000340548	path:04110_17
lymphocytes	NFYA	0.005565492	path:04110_19
lymphocytes	NFYA	0.002132484	path:04110_22
lymphocytes	NFYA	0.000299258	path:04110_23
lymphocytes	NFYA	0.004551637	path:04110_26
lymphocytes	NFYA	0.000299258	path:04110_3

lymphocytes	NFYA	0.004713566	path:04110_4
lymphocytes	NFYA	0.000340548	path:04110_7
lymphocytes	NFYA	0.004551637	path:04110_8
lymphocytes	NFYA	0.001111281	path:04115_1
lymphocytes	NFYA	0.000123767	path:04115_2
lymphocytes	NFYA	0.000776011	path:04115_3
lymphocytes	NFYA	0.000776011	path:04115_4
lymphocytes	NFYA	0.000776011	path:04115_7
lymphocytes	TP53	0.007702858	path:04010_15
lymphocytes	TP53	0.009062653	path:04010_16
lymphocytes	TP53	0.007443002	path:04010_21
lymphocytes	TP53	0.002466645	path:04012_12
lymphocytes	TP53	2.55E-11	path:04110_1
lymphocytes	TP53	3.28E-07	path:04110_17
lymphocytes	TP53	1.48E-05	path:04110_19
lymphocytes	TP53	0.001943417	path:04110_20
lymphocytes	TP53	7.56E-05	path:04110_22
lymphocytes	TP53	0.000198814	path:04110_23
lymphocytes	TP53	0.001943417	path:04110_27
lymphocytes	TP53	0.000210553	path:04110_3
lymphocytes	TP53	0.004511216	path:04110_4
lymphocytes	TP53	4.42E-09	path:04110_7
lymphocytes	TP53	0.004291125	path:04110_8
lymphocytes	TP53	0	path:04115_1
lymphocytes	TP53	0	path:04115_2
lymphocytes	TP53	0	path:04115_3
lymphocytes	TP53	0	path:04115_4
lymphocytes	TP53	0	path:04115_7
lymphocytes	TP53	0.000421718	path:04210_20
lymphocytes	TP53	0.000275763	path:04210_23
lymphocytes	TP53	0.000275763	path:04210_24
lymphocytes	TP53	0.009131774	path:04510_10
lymphocytes	TP53	0.004511216	path:04510_15
lymphocytes	TP53	0.002466645	path:04510_16

lymphocytes	TP53	0.000580605	path:04510_17
lymphocytes	TP53	0.001774645	path:04510_18
lymphocytes	TP53	0.006951959	path:04510_25
lymphocytes	TP53	0.006951959	path:04510_5
lymphocytes	TP53	0.009576653	path:04510_6
lymphocytes	TP53	0.001110697	path:04510_7
lymphocytes	TP53	0.00935253	path:04510_8
lymphocytes	TP53	0.009062653	path:04621_7
lymphocytes	TP53	0.000218217	path:04722_19
lymphocytes	TP53	0.002756828	path:04912_8
lymphocytes	TP53	0.009062653	path:05014_4
lymphocytes	TP53	0.007459365	path:05160_12
lymphocytes	TP53	0.009062653	path:05160_15
lymphocytes	TP53	0.000338058	path:05200_10
lymphocytes	TP53	0.000275763	path:05200_11
lymphocytes	TP53	0.000275763	path:05200_12
lymphocytes	TP53	0.000968321	path:05200_15
lymphocytes	TP53	0.00392722	path:05200_17
lymphocytes	TP53	0.002997803	path:05200_19
lymphocytes	TP53	0.00011707	path:05200_20
lymphocytes	TP53	0.000275763	path:05200_4
lymphocytes	TP53	7.56E-05	path:05200_47
lymphocytes	TP53	1.95E-05	path:05200_48
lymphocytes	TP53	0.000367024	path:05200_5
lymphocytes	TP53	0.000172314	path:05200_51
lymphocytes	TP53	1.95E-05	path:05200_8
lymphocytes	TP53	0.007459365	path:05213_2
lymphocytes	TP53	0.003580594	path:05214_1
lymphocytes	TP53	0.000210553	path:05214_10
lymphocytes	TP53	0.001774645	path:05214_11
lymphocytes	TP53	1.95E-05	path:05214_12
lymphocytes	TP53	0.00011707	path:05214_13
lymphocytes	TP53	7.56E-05	path:05214_14
lymphocytes	TP53	0.003580594	path:05214_4

lymphocytes	TP53	0.004511216	path:05215_10
lymphocytes	TP53	0.000275763	path:05215_11
lymphocytes	TP53	0.00331496	path:05215_3
lymphocytes	TP53	0.002754132	path:05218_2
lymphocytes	TP53	0.001774645	path:05218_5
lymphocytes	TP53	1.95E-05	path:05218_6
lymphocytes	TP53	0.001385048	path:05218_8
lymphocytes	TP53	0.001691972	path:05219_3
lymphocytes	TP53	0.009062653	path:05219_4
lymphocytes	TP53	0.00011707	path:05220_12
lymphocytes	TP53	1.95E-05	path:05220_8
lymphocytes	TP53	7.56E-05	path:05220_9
macrophages	CEBPA	0.0042174	path:04060_54
macrophages	CEBPA	0.00918123	path:04610_3
macrophages	CEBPA	0.00018207	path:04610_4
macrophages	CEBPA	0.00018207	path:04610_6
macrophages	SPI1	0.00153244	path:04060_41
macrophages	SPI1	0.005348786	path:04145_2
macrophages	SPI1	3.11E-06	path:04145_6
macrophages	SPI1	0.007630608	path:04514_55
macrophages	SPI1	0.003570772	path:04514_62
macrophages	SPI1	0.008430928	path:04650_10
macrophages	SPI1	0.007340609	path:04650_12
macrophages	SPI1	0.003805584	path:04650_13
macrophages	SPI1	0.003570772	path:04670_10
macrophages	SPI1	0.00153244	path:04670_14
macrophages	SPI1	0.000961365	path:04670_15
macrophages	SPI1	0.000252274	path:04670_2
macrophages	SPI1	0.000110411	path:05140_4
macrophages	SPI1	0.005348786	path:05150_17
macrophages	SPI1	0.000902646	path:05200_43
MCF7	E2F1	0.000197534	path:04110_1
MCF7	E2F1	0.008121608	path:04110_15
MCF7	E2F1	0.006872543	path:04110_16

MCF7	E2F1	9.20E-09	path:04110_17
MCF7	E2F1	1.74E-07	path:04110_19
MCF7	E2F1	2.73E-06	path:04110_20
MCF7	E2F1	0.000308684	path:04110_22
MCF7	E2F1	1.13E-07	path:04110_23
MCF7	E2F1	1.13E-07	path:04110_26
MCF7	E2F1	2.73E-06	path:04110_27
MCF7	E2F1	1.13E-07	path:04110_3
MCF7	E2F1	1.13E-07	path:04110_4
MCF7	E2F1	1.74E-05	path:04110_7
MCF7	E2F1	0.00499983	path:04115_1
MCF7	E2F1	0.009147152	path:04510_11
MCF7	E2F1	0.004589831	path:05014_4
MCF7	E2F1	1.56E-05	path:05200_51
MCF7	E2F1	9.20E-09	path:05200_52
MCF7	E2F1	1.85E-07	path:05200_53
MCF7	E2F1	0.002554435	path:05200_54
MCF7	E2F1	1.10E-07	path:05212_13
MCF7	E2F1	1.07E-05	path:05214_13
MCF7	E2F1	1.13E-07	path:05214_15
MCF7	E2F1	1.07E-05	path:05214_21
MCF7	E2F1	1.56E-05	path:05215_7
MCF7	E2F1	1.13E-07	path:05218_7
MCF7	E2F1	0.000197534	path:05218_8
MCF7	E2F1	1.10E-07	path:05219_4
MCF7	E2F1	1.13E-07	path:05220_11
MCF7	E2F1	1.07E-05	path:05220_12
MCF7	E2F1	3.73E-07	path:05222_1
MCF7	E2F1	0.009147152	path:05222_2
MCF7	E2F1	1.10E-07	path:05223_3
MCF7	ESR1	0.005413618	path:00830_2
MCF7	ESR1	0.000345486	path:04115_1
MCF7	ESR1	0.002979325	path:04115_2
MCF7	ESR1	0.002948202	path:04115_3

MCF7	ESR1	0.002948202	path:04115_4
MCF7	ESR1	0.002948202	path:04115_7
MCF7	ESR1	0.002948202	path:04610_8
MCF7	ESR1	0.000345486	path:05200_29
MCF7	FOS	0.005719136	path:04115_1
MCF7	FOS	0.006308233	path:04310_7
MCF7	FOS	0.003173805	path:04610_7
MCF7	FOS	0.006237239	path:04620_17
MCF7	FOS	0.003173805	path:04620_18
MCF7	FOS	0.006237239	path:04620_22
MCF7	FOS	0.006237239	path:04620_9
MCF7	FOS	2.16E-08	path:05200_29
MCF7	NFIC	0.004396514	path:00591_1
MCF7	NFIC	0.005988829	path:00982_11
MCF7	NFIC	0.00921451	path:00982_2
MCF7	NFIC	0.005988829	path:00982_3
MCF7	NFIC	0.00787147	path:00982_4
MCF7	NFIC	0.00787147	path:00982_5
MCF7	NFIC	0.004396514	path:00982_7
MCF7	NFIC	0.004396514	path:00982_8
MCF7	NFIC	0.00787147	path:00982_9
MCF7	NFIC	0.00787147	path:04010_30
MCF7	NFIC	0.00787147	path:04010_31
MCF7	NFIC	0.004396514	path:04080_7
MCF7	NFIC	0.008000492	path:04115_1
MCF7	NFIC	0.00787147	path:04115_2
MCF7	NFIC	0.00787147	path:04115_3
MCF7	NFIC	0.00787147	path:04115_4
MCF7	NFIC	0.00787147	path:04115_7
MCF7	NFIC	0.00787147	path:04660_8
MCF7	NFIC	0.00787147	path:04662_6
MCF7	NFIC	0.00787147	path:04722_19
MCF7	NFIC	0.00787147	path:05160_15
MCF7	NFIC	0.007050709	path:05200_47

MCF7	NFIC	0.004396514	path:05200_48
MCF7	NFIC	0.00787147	path:05200_51
MCF7	NFIC	0.004396514	path:05214_12
MCF7	NFIC	0.00787147	path:05214_13
MCF7	NFIC	0.007050709	path:05214_14
MCF7	NFIC	0.004396514	path:05218_5
MCF7	NFIC	0.004396514	path:05218_6
MCF7	NFIC	0.00787147	path:05220_12
MCF7	NFIC	0.004396514	path:05220_8
MCF7	NFIC	0.007050709	path:05220_9
MCF7	SP1	0.006087664	path:00120_1
MCF7	SP1	0.003885876	path:00120_2
MCF7	SP1	0.003885876	path:00120_3
MCF7	SP1	0.001893526	path:00140_1
MCF7	SP1	1.44E-05	path:00140_10
MCF7	SP1	0.001534931	path:00140_11
MCF7	SP1	0.006087664	path:00140_12
MCF7	SP1	2.77E-05	path:00140_13
MCF7	SP1	0.003885876	path:00140_14
MCF7	SP1	0.000245716	path:00140_16
MCF7	SP1	0.000245716	path:00140_18
MCF7	SP1	2.77E-05	path:00140_19
MCF7	SP1	0.000441307	path:00140_20
MCF7	SP1	0.001893526	path:00140_25
MCF7	SP1	1.44E-05	path:00140_3
MCF7	SP1	1.44E-05	path:00140_4
MCF7	SP1	5.07E-05	path:00140_5
MCF7	SP1	8.66E-05	path:00140_6
MCF7	SP1	2.77E-05	path:00140_7
MCF7	SP1	0.000123921	path:00140_8
MCF7	SP1	0.000123921	path:00140_9
MCF7	SP1	0.005536058	path:00240_16
MCF7	SP1	0.003391309	path:00240_17
MCF7	SP1	0.001690362	path:00240_2

MCF7	SP1	0.009002988	path:00240_5
MCF7	SP1	0.005536058	path:00330_1
MCF7	SP1	0.005126386	path:00330_9
MCF7	SP1	0.006192277	path:00350_2
MCF7	SP1	0.006192277	path:00350_3
MCF7	SP1	0.001393247	path:00980_2
MCF7	SP1	0.002175912	path:00982_10
MCF7	SP1	0.003931316	path:04010_10
MCF7	SP1	0.000441307	path:04010_26
MCF7	SP1	0.000123921	path:04010_5
MCF7	SP1	0.002149604	path:04060_22
MCF7	SP1	0.001014956	path:04060_32
MCF7	SP1	0.007433507	path:04060_47
MCF7	SP1	0.00916136	path:04110_1
MCF7	SP1	1.86E-10	path:04110_17
MCF7	SP1	4.28E-11	path:04110_18
MCF7	SP1	1.05E-10	path:04110_19
MCF7	SP1	2.92E-09	path:04110_20
MCF7	SP1	1.43E-05	path:04110_22
MCF7	SP1	5.22E-09	path:04110_23
MCF7	SP1	0.006192277	path:04110_25
MCF7	SP1	7.70E-08	path:04110_26
MCF7	SP1	2.92E-09	path:04110_27
MCF7	SP1	6.46E-09	path:04110_3
MCF7	SP1	6.46E-09	path:04110_4
MCF7	SP1	4.97E-07	path:04110_7
MCF7	SP1	3.47E-10	path:04115_1
MCF7	SP1	6.74E-05	path:04115_2
MCF7	SP1	4.23E-05	path:04115_3
MCF7	SP1	6.18E-06	path:04115_4
MCF7	SP1	4.96E-05	path:04115_7
MCF7	SP1	7.61E-06	path:04144_2
MCF7	SP1	0.002175912	path:04210_20
MCF7	SP1	0.001107767	path:04210_24

MCF7	SP1	1.33E-05	path:04310_6
MCF7	SP1	1.77E-05	path:04310_7
MCF7	SP1	7.85E-13	path:04510_1
MCF7	SP1	1.05E-10	path:04510_10
MCF7	SP1	0.001603207	path:04510_13
MCF7	SP1	0.001164758	path:04510_15
MCF7	SP1	0.002771235	path:04510_16
MCF7	SP1	0.000141426	path:04510_17
MCF7	SP1	8.07E-08	path:04510_18
MCF7	SP1	1.96E-12	path:04510_19
MCF7	SP1	1.43E-05	path:04510_20
MCF7	SP1	0.000718519	path:04510_23
MCF7	SP1	0.002175912	path:04510_24
MCF7	SP1	0.0024986	path:04510_25
MCF7	SP1	6.74E-05	path:04510_4
MCF7	SP1	0.0024986	path:04510_5
MCF7	SP1	1.07E-10	path:04510_6
MCF7	SP1	0.000448433	path:04510_7
MCF7	SP1	6.16E-10	path:04510_8
MCF7	SP1	2.01E-05	path:04512_10
MCF7	SP1	5.55E-06	path:04512_11
MCF7	SP1	1.66E-06	path:04512_12
MCF7	SP1	7.86E-06	path:04512_13
MCF7	SP1	0.000101569	path:04512_14
MCF7	SP1	0.000245716	path:04512_18
MCF7	SP1	0.000549822	path:04512_19
MCF7	SP1	0.002149604	path:04512_20
MCF7	SP1	0.008608739	path:04512_22
MCF7	SP1	0.00016592	path:04512_27
MCF7	SP1	1.66E-05	path:04512_3
MCF7	SP1	2.72E-07	path:04512_5
MCF7	SP1	6.24E-07	path:04512_6
MCF7	SP1	9.36E-06	path:04512_9
MCF7	SP1	0.005126386	path:04520_3

MCF7	SP1	0.001603207	path:04520_5
MCF7	SP1	0.000412181	path:04520_7
MCF7	SP1	1.22E-05	path:04540_13
MCF7	SP1	9.21E-07	path:04610_3
MCF7	SP1	5.55E-06	path:04610_4
MCF7	SP1	9.21E-07	path:04610_6
MCF7	SP1	0.000133832	path:04610_7
MCF7	SP1	0.000203885	path:04610_8
MCF7	SP1	0.000203885	path:04620_18
MCF7	SP1	0.001893526	path:04620_9
MCF7	SP1	0.000231547	path:04630_1
MCF7	SP1	0.001390056	path:04630_2
MCF7	SP1	1.97E-06	path:04630_3
MCF7	SP1	5.95E-06	path:04630_4
MCF7	SP1	4.84E-05	path:04650_20
MCF7	SP1	0.008608739	path:04650_7
MCF7	SP1	0.000718519	path:04722_19
MCF7	SP1	7.92E-07	path:04810_29
MCF7	SP1	1.33E-05	path:04912_8
MCF7	SP1	0.007433507	path:04940_4
MCF7	SP1	0.006087664	path:05014_3
MCF7	SP1	0.008608739	path:05014_4
MCF7	SP1	0.007433507	path:05016_8
MCF7	SP1	0.001014956	path:05100_3
MCF7	SP1	0.008608739	path:05140_10
MCF7	SP1	0.000245716	path:05140_6
MCF7	SP1	0.001107767	path:05142_10
MCF7	SP1	0.003931316	path:05142_11
MCF7	SP1	0.003885876	path:05142_17
MCF7	SP1	0.003885876	path:05142_18
MCF7	SP1	0.000441307	path:05142_20
MCF7	SP1	0.000718519	path:05142_21
MCF7	SP1	0.001107767	path:05142_4
MCF7	SP1	0.006087664	path:05145_19

MCF7	SP1	0.001014956	path:05145_21
MCF7	SP1	0.006087664	path:05145_8
MCF7	SP1	1.23E-06	path:05146_1
MCF7	SP1	7.92E-07	path:05146_2
MCF7	SP1	0.002149604	path:05146_5
MCF7	SP1	9.10E-07	path:05146_9
MCF7	SP1	0.003935354	path:05200_19
MCF7	SP1	0.003935354	path:05200_20
MCF7	SP1	0.00927462	path:05200_24
MCF7	SP1	9.21E-07	path:05200_29
MCF7	SP1	2.01E-05	path:05200_3
MCF7	SP1	0.000718519	path:05200_31
MCF7	SP1	0.008608739	path:05200_38
MCF7	SP1	0.000289957	path:05200_4
MCF7	SP1	0.000718519	path:05200_42
MCF7	SP1	2.07E-08	path:05200_47
MCF7	SP1	1.33E-05	path:05200_48
MCF7	SP1	5.07E-05	path:05200_50
MCF7	SP1	5.54E-09	path:05200_51
MCF7	SP1	1.55E-08	path:05200_52
MCF7	SP1	4.11E-06	path:05200_53
MCF7	SP1	0.001014956	path:05200_56
MCF7	SP1	0.000441307	path:05210_10
MCF7	SP1	0.000718519	path:05210_12
MCF7	SP1	0.000718519	path:05210_7
MCF7	SP1	1.44E-05	path:05212_13
MCF7	SP1	0.008608739	path:05212_14
MCF7	SP1	1.33E-05	path:05214_12
MCF7	SP1	1.18E-07	path:05214_13
MCF7	SP1	2.07E-08	path:05214_14
MCF7	SP1	9.21E-07	path:05214_15
MCF7	SP1	0.005536058	path:05214_2
MCF7	SP1	0.000245716	path:05214_21
MCF7	SP1	0.005536058	path:05214_5

MCF7	SP1	0.001164758	path:05215_10
MCF7	SP1	0.008608739	path:05215_7
MCF7	SP1	0.000441307	path:05216_4
MCF7	SP1	0.000411461	path:05218_4
MCF7	SP1	0.000133832	path:05218_5
MCF7	SP1	1.33E-05	path:05218_6
MCF7	SP1	9.21E-07	path:05218_7
MCF7	SP1	7.27E-05	path:05218_8
MCF7	SP1	0.000310459	path:05219_3
MCF7	SP1	1.44E-05	path:05219_4
MCF7	SP1	9.21E-07	path:05220_11
MCF7	SP1	1.18E-07	path:05220_12
MCF7	SP1	0.002149604	path:05220_5
MCF7	SP1	1.33E-05	path:05220_8
MCF7	SP1	2.07E-08	path:05220_9
MCF7	SP1	0.006192277	path:05221_1
MCF7	SP1	5.36E-07	path:05222_1
MCF7	SP1	0.000133832	path:05222_2
MCF7	SP1	2.01E-05	path:05222_4
MCF7	SP1	0.001603207	path:05222_7
MCF7	SP1	1.44E-05	path:05223_3
MCF7	SP1	0.007433507	path:05320_9
MCF7	SP1	0.007433507	path:05330_5
MCF7	SP1	0.007433507	path:05332_2
MCF7	SP1	0.007433507	path:05332_5
MCF7	SP1	1.77E-05	path:05414_2
MCF7	STAT3	0.007358355	path:04060_3
MCF7	STAT3	0.008398489	path:04060_8
MCF7	STAT3	0.00794491	path:04630_2
MCF7	STAT3	0.00794491	path:04630_3
MCF7	STAT3	5.36E-05	path:04630_4
MCF7	STAT3	0.000767514	path:05142_4
MCF7	STAT3	0.000108961	path:05200_29
MCF7	TFAP2C	0.009425324	path:04520_9

MCF7	TFAP2C	0.00404499	path:04912_8
MCF7	TFAP2C	0.00404499	path:05219_2
MCF7	TP53	0.007702858	path:04010_15
MCF7	TP53	0.009062653	path:04010_16
MCF7	TP53	0.007443002	path:04010_21
MCF7	TP53	0.002466645	path:04012_12
MCF7	TP53	2.55E-11	path:04110_1
MCF7	TP53	3.28E-07	path:04110_17
MCF7	TP53	1.48E-05	path:04110_19
MCF7	TP53	0.001943417	path:04110_20
MCF7	TP53	7.56E-05	path:04110_22
MCF7	TP53	0.000198814	path:04110_23
MCF7	TP53	0.001943417	path:04110_27
MCF7	TP53	0.000210553	path:04110_3
MCF7	TP53	0.004511216	path:04110_4
MCF7	TP53	4.42E-09	path:04110_7
MCF7	TP53	0.004291125	path:04110_8
MCF7	TP53	0	path:04115_1
MCF7	TP53	0	path:04115_2
MCF7	TP53	0	path:04115_3
MCF7	TP53	0	path:04115_4
MCF7	TP53	0	path:04115_7
MCF7	TP53	0.000421718	path:04210_20
MCF7	TP53	0.000275763	path:04210_23
MCF7	TP53	0.000275763	path:04210_24
MCF7	TP53	0.009131774	path:04510_10
MCF7	TP53	0.004511216	path:04510_15
MCF7	TP53	0.002466645	path:04510_16
MCF7	TP53	0.000580605	path:04510_17
MCF7	TP53	0.001774645	path:04510_18
MCF7	TP53	0.006951959	path:04510_25
MCF7	TP53	0.006951959	path:04510_5
MCF7	TP53	0.009576653	path:04510_6
MCF7	TP53	0.001110697	path:04510_7

MCF7	TP53	0.00935253	path:04510_8
MCF7	TP53	0.009062653	path:04621_7
MCF7	TP53	0.000218217	path:04722_19
MCF7	TP53	0.002756828	path:04912_8
MCF7	TP53	0.009062653	path:05014_4
MCF7	TP53	0.007459365	path:05160_12
MCF7	TP53	0.009062653	path:05160_15
MCF7	TP53	0.000338058	path:05200_10
MCF7	TP53	0.000275763	path:05200_11
MCF7	TP53	0.000275763	path:05200_12
MCF7	TP53	0.000968321	path:05200_15
MCF7	TP53	0.00392722	path:05200_17
MCF7	TP53	0.002997803	path:05200_19
MCF7	TP53	0.00011707	path:05200_20
MCF7	TP53	0.000275763	path:05200_4
MCF7	TP53	7.56E-05	path:05200_47
MCF7	TP53	1.95E-05	path:05200_48
MCF7	TP53	0.000367024	path:05200_5
MCF7	TP53	0.000172314	path:05200_51
MCF7	TP53	1.95E-05	path:05200_8
MCF7	TP53	0.007459365	path:05213_2
MCF7	TP53	0.003580594	path:05214_1
MCF7	TP53	0.000210553	path:05214_10
MCF7	TP53	0.001774645	path:05214_11
MCF7	TP53	1.95E-05	path:05214_12
MCF7	TP53	0.00011707	path:05214_13
MCF7	TP53	7.56E-05	path:05214_14
MCF7	TP53	0.003580594	path:05214_4
MCF7	TP53	0.004511216	path:05215_10
MCF7	TP53	0.000275763	path:05215_11
MCF7	TP53	0.00331496	path:05215_3
MCF7	TP53	0.002754132	path:05218_2
MCF7	TP53	0.001774645	path:05218_5
MCF7	TP53	1.95E-05	path:05218_6

MCF7	TP53	0.001385048	path:05218_8
MCF7	TP53	0.001691972	path:05219_3
MCF7	TP53	0.009062653	path:05219_4
MCF7	TP53	0.00011707	path:05220_12
MCF7	TP53	1.95E-05	path:05220_8
MCF7	TP53	7.56E-05	path:05220_9
MCF7	VDR	0.009786942	path:00140_1
MCF7	VDR	0.008771608	path:00140_11
MCF7	VDR	0.009786942	path:00140_25
MCF7	VDR	0.009786942	path:00140_26
MCF7	VDR	0.008771608	path:00591_1
MCF7	VDR	0.00973729	path:00980_2
MCF7	VDR	0.004788204	path:00982_10
MCF7	VDR	0.001635842	path:00982_11
MCF7	VDR	0.003675018	path:00982_2
MCF7	VDR	0.001635842	path:00982_3
MCF7	VDR	0.003361044	path:00982_4
MCF7	VDR	0.003361044	path:00982_5
MCF7	VDR	0.008771608	path:00982_6
MCF7	VDR	0.001635842	path:00982_7
MCF7	VDR	0.001635842	path:00982_8
MCF7	VDR	0.003361044	path:00982_9
MCF7	VDR	0.008771608	path:04012_1
MCF7	VDR	0.008771608	path:04012_11
MCF7	VDR	0.008771608	path:04012_12
MCF7	VDR	0.0090937	path:04012_14
MCF7	VDR	0.008771608	path:04012_4
MCF7	VDR	0.008771608	path:04012_7
MCF7	VDR	0.008771608	path:05200_9
MCF7	VDR	0.008771608	path:05214_1
MCF7	VDR	0.009415594	path:05214_10
MCF7	VDR	0.008771608	path:05214_17
MCF7	VDR	0.008771608	path:05214_2
MCF7	VDR	0.008771608	path:05214_3

MCF7	VDR	0.008771608	path:05214_4
MCF7	VDR	0.008771608	path:05214_5
MCF7	VDR	0.008771608	path:05214_6
MCF7	VDR	0.008771608	path:05214_8
MCF7	VDR	0.009786942	path:05215_11
MCF7	VDR	0.008771608	path:05218_2
MCF7	VDR	0.008771608	path:05223_6
MDA-MB-231	E2F1	0.000197534	path:04110_1
MDA-MB-231	E2F1	0.008121608	path:04110_15
MDA-MB-231	E2F1	0.006872543	path:04110_16
MDA-MB-231	E2F1	9.20E-09	path:04110_17
MDA-MB-231	E2F1	1.74E-07	path:04110_19
MDA-MB-231	E2F1	2.73E-06	path:04110_20
MDA-MB-231	E2F1	0.000308684	path:04110_22
MDA-MB-231	E2F1	1.13E-07	path:04110_23
MDA-MB-231	E2F1	1.13E-07	path:04110_26
MDA-MB-231	E2F1	2.73E-06	path:04110_27
MDA-MB-231	E2F1	1.13E-07	path:04110_3
MDA-MB-231	E2F1	1.13E-07	path:04110_4
MDA-MB-231	E2F1	1.74E-05	path:04110_7
MDA-MB-231	E2F1	0.00499983	path:04115_1
MDA-MB-231	E2F1	0.009147152	path:04510_11
MDA-MB-231	E2F1	0.004589831	path:05014_4
MDA-MB-231	E2F1	1.56E-05	path:05200_51
MDA-MB-231	E2F1	9.20E-09	path:05200_52
MDA-MB-231	E2F1	1.85E-07	path:05200_53
MDA-MB-231	E2F1	0.002554435	path:05200_54
MDA-MB-231	E2F1	1.10E-07	path:05212_13
MDA-MB-231	E2F1	1.07E-05	path:05214_13
MDA-MB-231	E2F1	1.13E-07	path:05214_15
MDA-MB-231	E2F1	1.07E-05	path:05214_21
MDA-MB-231	E2F1	1.56E-05	path:05215_7
MDA-MB-231	E2F1	1.13E-07	path:05218_7
MDA-MB-231	E2F1	0.000197534	path:05218_8

MDA-MB-231	E2F1	1.10E-07	path:05219_4
MDA-MB-231	E2F1	1.13E-07	path:05220_11
MDA-MB-231	E2F1	1.07E-05	path:05220_12
MDA-MB-231	E2F1	3.73E-07	path:05222_1
MDA-MB-231	E2F1	0.009147152	path:05222_2
MDA-MB-231	E2F1	1.10E-07	path:05223_3
MDA-MB-231	STAT3	0.007358355	path:04060_3
MDA-MB-231	STAT3	0.008398489	path:04060_8
MDA-MB-231	STAT3	0.00794491	path:04630_2
MDA-MB-231	STAT3	0.00794491	path:04630_3
MDA-MB-231	STAT3	5.36E-05	path:04630_4
MDA-MB-231	STAT3	0.000767514	path:05142_4
MDA-MB-231	STAT3	0.000108961	path:05200_29
MDA-MB-468	E2F1	0.000197534	path:04110_1
MDA-MB-468	E2F1	0.008121608	path:04110_15
MDA-MB-468	E2F1	0.006872543	path:04110_16
MDA-MB-468	E2F1	9.20E-09	path:04110_17
MDA-MB-468	E2F1	1.74E-07	path:04110_19
MDA-MB-468	E2F1	2.73E-06	path:04110_20
MDA-MB-468	E2F1	0.000308684	path:04110_22
MDA-MB-468	E2F1	1.13E-07	path:04110_23
MDA-MB-468	E2F1	1.13E-07	path:04110_26
MDA-MB-468	E2F1	2.73E-06	path:04110_27
MDA-MB-468	E2F1	1.13E-07	path:04110_3
MDA-MB-468	E2F1	1.13E-07	path:04110_4
MDA-MB-468	E2F1	1.74E-05	path:04110_7
MDA-MB-468	E2F1	0.00499983	path:04115_1
MDA-MB-468	E2F1	0.009147152	path:04510_11
MDA-MB-468	E2F1	0.004589831	path:05014_4
MDA-MB-468	E2F1	1.56E-05	path:05200_51
MDA-MB-468	E2F1	9.20E-09	path:05200_52
MDA-MB-468	E2F1	1.85E-07	path:05200_53
MDA-MB-468	E2F1	0.002554435	path:05200_54
MDA-MB-468	E2F1	1.10E-07	path:05212_13

MDA-MB-468	E2F1	1.07E-05	path:05214_13
MDA-MB-468	E2F1	1.13E-07	path:05214_15
MDA-MB-468	E2F1	1.07E-05	path:05214_21
MDA-MB-468	E2F1	1.56E-05	path:05215_7
MDA-MB-468	E2F1	1.13E-07	path:05218_7
MDA-MB-468	E2F1	0.000197534	path:05218_8
MDA-MB-468	E2F1	1.10E-07	path:05219_4
MDA-MB-468	E2F1	1.13E-07	path:05220_11
MDA-MB-468	E2F1	1.07E-05	path:05220_12
MDA-MB-468	E2F1	3.73E-07	path:05222_1
MDA-MB-468	E2F1	0.009147152	path:05222_2
MDA-MB-468	E2F1	1.10E-07	path:05223_3
MDA-MB-468	STAT3	0.007358355	path:04060_3
MDA-MB-468	STAT3	0.008398489	path:04060_8
MDA-MB-468	STAT3	0.00794491	path:04630_2
MDA-MB-468	STAT3	0.00794491	path:04630_3
MDA-MB-468	STAT3	5.36E-05	path:04630_4
MDA-MB-468	STAT3	0.000767514	path:05142_4
MDA-MB-468	STAT3	0.000108961	path:05200_29
muscle	NR1I3	0.000186607	path:00830_2
muscle	NR1I3	0.006275482	path:00980_1
muscle	NR1I3	0.000535628	path:00982_4
muscle	NR1I3	0.000535628	path:00982_5
muscle	NR1I3	0.000535628	path:00982_9
muscle	NR4A1	0.00077713	path:00140_10
muscle	NR4A1	6.42E-06	path:00140_13
muscle	NR4A1	0.00077713	path:00140_14
muscle	NR4A1	0.00077713	path:00140_16
muscle	NR4A1	0.00077713	path:00140_18
muscle	NR4A1	0.000832486	path:00140_19
muscle	NR4A1	0.00077713	path:00140_20
muscle	NR4A1	0.00077713	path:00140_3
muscle	NR4A1	0.00077713	path:00140_4
muscle	NR4A1	0.000960385	path:00140_5

muscle	NR4A1	0.001089761	path:00140_6
muscle	NR4A1	0.000832486	path:00140_7
muscle	NR4A1	0.00077713	path:00140_8
muscle	NR4A1	0.00077713	path:00140_9
muscle	SREBF1	0.009882974	path:00100_9
muscle	SREBF1	0.009882974	path:03320_2
muscle	SREBF1	3.27E-05	path:04910_5
muscle	SREBF1	0.00143191	path:04910_6
neuroblastoma	GATA3	0.003994597	path:04612_4
neuroblastoma	TP73	6.75E-05	path:04115_1
neuroblastoma	TP73	6.67E-05	path:04115_2
neuroblastoma	TP73	6.67E-05	path:04115_3
neuroblastoma	TP73	6.67E-05	path:04115_4
neuroblastoma	TP73	6.67E-05	path:04115_7
neuroblastoma	TP73	0.000595015	path:04722_19
ovary	AKNA	6.20E-07	path:04060_31
ovary	AKNA	6.20E-07	path:04514_10
ovary	AKNA	6.20E-07	path:04514_11
ovary	AKNA	6.20E-07	path:04514_12
ovary	AKNA	6.20E-07	path:04514_78
ovary	AKNA	6.20E-07	path:04514_9
ovary	AKNA	6.20E-07	path:04672_2
ovary	AKNA	6.20E-07	path:05144_9
ovary	AKNA	6.20E-07	path:05145_20
ovary	AKNA	6.20E-07	path:05310_2
ovary	AKNA	6.20E-07	path:05320_2
ovary	AKNA	6.20E-07	path:05320_5
ovary	AKNA	6.20E-07	path:05322_7
ovary	AKNA	6.20E-07	path:05330_12
ovary	AKNA	6.20E-07	path:05330_3
ovary	AKNA	6.20E-07	path:05416_3
pancreas	ELF1	8.41E-06	path:04060_8
pancreas	ELF1	0.006320724	path:04650_1
pancreas	ELF1	0.001225713	path:04650_14

pancreas	ELF1	0.000687264	path:04650_15
pancreas	ELF1	0.000687264	path:04664_1
pancreas	ELF1	0.000533253	path:04664_10
pancreas	ELF1	1.05E-05	path:04664_2
pancreas	ELF1	0.000533253	path:04664_3
pancreas	ELF1	0.000533253	path:04664_5
pancreas	ELF1	0.000533253	path:04664_9
pancreas	ELF1	0.006320724	path:05142_21
pancreas	ELF1	0.000687264	path:05310_4
pancreas	ELF1	0.000687264	path:05310_5
pancreas	FOXA3	0.000327469	path:00140_1
pancreas	FOXA3	0.000188543	path:00140_11
pancreas	FOXA3	0.000502586	path:00140_12
pancreas	FOXA3	0.000327469	path:00140_25
pancreas	FOXA3	0.000327469	path:00140_26
pancreas	FOXA3	0.000312584	path:00591_1
pancreas	FOXA3	0.000975791	path:00830_2
pancreas	FOXA3	0.000327469	path:00980_2
pancreas	FOXA3	7.28E-05	path:00982_10
pancreas	FOXA3	2.48E-05	path:00982_11
pancreas	FOXA3	5.58E-05	path:00982_2
pancreas	FOXA3	2.48E-05	path:00982_3
pancreas	FOXA3	5.10E-05	path:00982_4
pancreas	FOXA3	5.10E-05	path:00982_5
pancreas	FOXA3	0.000248984	path:00982_6
pancreas	FOXA3	2.48E-05	path:00982_7
pancreas	FOXA3	2.48E-05	path:00982_8
pancreas	FOXA3	5.10E-05	path:00982_9
pancreas	HNF4A	0.00730316	path:00120_15
pancreas	HNF4A	0.003812707	path:00120_19
pancreas	HNF4A	0.001698537	path:00120_6
pancreas	HNF4A	0.003812707	path:00120_7
pancreas	HNF4A	0.000100826	path:00120_9
pancreas	HNF4A	0.001808381	path:00590_1

pancreas	HNF4A	0.001722691	path:00590_2
pancreas	HNF4A	0.001722691	path:00590_3
pancreas	HNF4A	0.001898001	path:00590_4
pancreas	HNF4A	0.000515682	path:00591_1
pancreas	HNF4A	2.36E-05	path:00830_2
pancreas	HNF4A	0.000733563	path:00980_2
pancreas	HNF4A	2.52E-07	path:00982_10
pancreas	HNF4A	5.38E-08	path:00982_4
pancreas	HNF4A	5.38E-08	path:00982_5
pancreas	HNF4A	5.38E-08	path:00982_9
pancreas	HNF4A	0.001131883	path:03320_1
pancreas	HNF4A	0.000100826	path:03320_2
pancreas	HNF4A	4.35E-06	path:04610_2
pancreas	HNF4A	4.35E-06	path:04610_3
pancreas	HNF4A	0.000100826	path:04610_4
pancreas	HNF4A	6.69E-06	path:04610_5
pancreas	HNF4A	0.000179282	path:04610_6
pancreas	HNF4A	0.001698537	path:04610_8
pancreas	HNF4A	0.000287791	path:04950_4
peripheral blood	AKNA	6.20E-07	path:04060_31
peripheral blood	AKNA	6.20E-07	path:04514_10
peripheral blood	AKNA	6.20E-07	path:04514_11
peripheral blood	AKNA	6.20E-07	path:04514_12
peripheral blood	AKNA	6.20E-07	path:04514_78
peripheral blood	AKNA	6.20E-07	path:04514_9
peripheral blood	AKNA	6.20E-07	path:04672_2
peripheral blood	AKNA	6.20E-07	path:05144_9
peripheral blood	AKNA	6.20E-07	path:05145_20
peripheral blood	AKNA	6.20E-07	path:05310_2
peripheral blood	AKNA	6.20E-07	path:05320_2
peripheral blood	AKNA	6.20E-07	path:05320_5
peripheral blood	AKNA	6.20E-07	path:05322_7
peripheral blood	AKNA	6.20E-07	path:05330_12
peripheral blood	AKNA	6.20E-07	path:05330_3

peripheral blood	AKNA	6.20E-07	path:05416_3
peripheral blood	IRF5	0.005011517	path:04060_38
peripheral blood	IRF5	0.005011517	path:04620_11
peripheral blood	IRF5	0.005011517	path:04620_2
peripheral blood	IRF5	0.005011517	path:04650_6
peripheral blood	IRF5	0.005011517	path:05160_6
peripheral blood	IRF7	5.44E-05	path:04060_38
peripheral blood	IRF7	3.65E-07	path:04620_11
peripheral blood	IRF7	1.24E-05	path:04620_12
peripheral blood	IRF7	0.005131788	path:04620_14
peripheral blood	IRF7	0.005500251	path:04620_16
peripheral blood	IRF7	0.009380523	path:04620_17
peripheral blood	IRF7	0.005305274	path:04620_18
peripheral blood	IRF7	3.65E-07	path:04620_2
peripheral blood	IRF7	0.009380523	path:04620_22
peripheral blood	IRF7	0.009501335	path:04620_9
peripheral blood	IRF7	5.75E-05	path:04650_6
peripheral blood	IRF7	5.75E-05	path:05160_6
placenta	KLF5	0.000357162	path:05200_31
placenta	KLF5	0.000357162	path:05210_12
placenta	KLF5	0.000357162	path:05210_7
placenta	SREBF1	0.009882974	path:00100_9
placenta	SREBF1	0.009882974	path:03320_2
placenta	SREBF1	3.27E-05	path:04910_5
placenta	SREBF1	0.00143191	path:04910_6
placenta	TP63	2.23E-05	path:04110_1
placenta	TP63	8.12E-05	path:04110_7
placenta	TP63	8.12E-05	path:04115_1
placenta	TP63	8.12E-05	path:04115_2
placenta	TP63	8.12E-05	path:04115_3
placenta	TP63	8.12E-05	path:04115_4
placenta	TP63	8.12E-05	path:04115_7
placenta	TP63	0.003115907	path:05200_17
placenta	TP63	0.002783081	path:05200_19

placenta	TP63	0.002783081	path:05200_20
placenta	TP63	0.000159403	path:05200_47
placenta	TP63	8.12E-05	path:05200_48
placenta	TP63	8.12E-05	path:05214_12
placenta	TP63	0.000159403	path:05214_14
placenta	TP63	0.004120782	path:05215_11
placenta	TP63	0.002837304	path:05215_3
placenta	TP63	8.12E-05	path:05218_6
placenta	TP63	8.12E-05	path:05220_8
placenta	TP63	0.000159403	path:05220_9
PMA	EGR1	0.009869535	path:04060_8
PMA	EGR1	0.00069989	path:04510_10
PMA	EGR1	0.00069989	path:04510_18
PMA	EGR1	0.003153788	path:04510_6
PMA	EGR1	0.003153788	path:04510_8
PMA	EGR1	0.00129686	path:04512_3
PMA	EGR1	0.003153788	path:04540_13
PMA	EGR1	0.003153788	path:05146_9
PMA	EGR1	0.007008495	path:05212_11
PMA	EGR1	0.008365691	path:05212_12
PMA	EGR1	0.003153788	path:05218_4
PMA	FOS	0.005719136	path:04115_1
PMA	FOS	0.006308233	path:04310_7
PMA	FOS	0.003173805	path:04610_7
PMA	FOS	0.006237239	path:04620_17
PMA	FOS	0.003173805	path:04620_18
PMA	FOS	0.006237239	path:04620_22
PMA	FOS	0.006237239	path:04620_9
PMA	FOS	2.16E-08	path:05200_29
PMA	RELA	0.000137451	path:04060_62
PMA	RELA	2.04E-07	path:04062_1
PMA	RELA	3.87E-08	path:04062_2
PMA	RELA	0.001869278	path:04110_17
PMA	RELA	0.001781655	path:04110_19

PMA	RELA	0.001869278	path:04110_7
PMA	RELA	0.0005539	path:04115_1
PMA	RELA	2.11E-06	path:04620_1
PMA	RELA	4.80E-09	path:04620_12
PMA	RELA	1.51E-07	path:04620_13
PMA	RELA	2.07E-07	path:04620_14
PMA	RELA	1.03E-08	path:04620_16
PMA	RELA	6.51E-08	path:04620_17
PMA	RELA	8.85E-09	path:04620_18
PMA	RELA	6.51E-08	path:04620_22
PMA	RELA	7.78E-08	path:04620_9
PMA	RELA	0.00197363	path:04650_22
PMA	RELA	0.001781655	path:04650_23
prostate	HNF1A	0.008512637	path:00120_5
prostate	HNF1A	0.008512637	path:00140_1
prostate	HNF1A	3.95E-05	path:00140_12
prostate	HNF1A	0.008512637	path:00140_22
prostate	HNF1A	0.008512637	path:00140_23
prostate	HNF1A	0.008512637	path:00140_24
prostate	HNF1A	0.008512637	path:00140_25
prostate	HNF1A	0.008512637	path:00140_26
prostate	HNF1A	0.008512637	path:00982_6
prostate	HNF1A	0.008512637	path:04610_6
prostate	TP63	2.23E-05	path:04110_1
prostate	TP63	8.12E-05	path:04110_7
prostate	TP63	8.12E-05	path:04115_1
prostate	TP63	8.12E-05	path:04115_2
prostate	TP63	8.12E-05	path:04115_3
prostate	TP63	8.12E-05	path:04115_4
prostate	TP63	8.12E-05	path:04115_7
prostate	TP63	0.003115907	path:05200_17
prostate	TP63	0.002783081	path:05200_19
prostate	TP63	0.002783081	path:05200_20
prostate	TP63	0.000159403	path:05200_47

prostate	TP63	8.12E-05	path:05200_48
prostate	TP63	8.12E-05	path:05214_12
prostate	TP63	0.000159403	path:05214_14
prostate	TP63	0.004120782	path:05215_11
prostate	TP63	0.002837304	path:05215_3
prostate	TP63	8.12E-05	path:05218_6
prostate	TP63	8.12E-05	path:05220_8
prostate	TP63	0.000159403	path:05220_9
small intestine	HNF4A	0.00730316	path:00120_15
small intestine	HNF4A	0.003812707	path:00120_19
small intestine	HNF4A	0.001698537	path:00120_6
small intestine	HNF4A	0.003812707	path:00120_7
small intestine	HNF4A	0.000100826	path:00120_9
small intestine	HNF4A	0.001808381	path:00590_1
small intestine	HNF4A	0.001722691	path:00590_2
small intestine	HNF4A	0.001722691	path:00590_3
small intestine	HNF4A	0.001898001	path:00590_4
small intestine	HNF4A	0.000515682	path:00591_1
small intestine	HNF4A	2.36E-05	path:00830_2
small intestine	HNF4A	0.000733563	path:00980_2
small intestine	HNF4A	2.52E-07	path:00982_10
small intestine	HNF4A	5.38E-08	path:00982_4
small intestine	HNF4A	5.38E-08	path:00982_5
small intestine	HNF4A	5.38E-08	path:00982_9
small intestine	HNF4A	0.001131883	path:03320_1
small intestine	HNF4A	0.000100826	path:03320_2
small intestine	HNF4A	4.35E-06	path:04610_2
small intestine	HNF4A	4.35E-06	path:04610_3
small intestine	HNF4A	0.000100826	path:04610_4
small intestine	HNF4A	6.69E-06	path:04610_5
small intestine	HNF4A	0.000179282	path:04610_6
small intestine	HNF4A	0.001698537	path:04610_8
small intestine	HNF4A	0.000287791	path:04950_4
small intestine	NR1I2	0.004391058	path:00830_2

small intestine	NR1I2	0.00011908	path:00982_4
small intestine	NR1I2	0.00011908	path:00982_5
small intestine	NR1I2	0.00011908	path:00982_9
spleen	AKNA	6.20E-07	path:04060_31
spleen	AKNA	6.20E-07	path:04514_10
spleen	AKNA	6.20E-07	path:04514_11
spleen	AKNA	6.20E-07	path:04514_12
spleen	AKNA	6.20E-07	path:04514_78
spleen	AKNA	6.20E-07	path:04514_9
spleen	AKNA	6.20E-07	path:04672_2
spleen	AKNA	6.20E-07	path:05144_9
spleen	AKNA	6.20E-07	path:05145_20
spleen	AKNA	6.20E-07	path:05310_2
spleen	AKNA	6.20E-07	path:05320_2
spleen	AKNA	6.20E-07	path:05320_5
spleen	AKNA	6.20E-07	path:05322_7
spleen	AKNA	6.20E-07	path:05330_12
spleen	AKNA	6.20E-07	path:05330_3
spleen	AKNA	6.20E-07	path:05416_3
spleen	CREB1	0.004288489	path:04514_1
spleen	CREB1	0.004288489	path:04514_3
spleen	CREB1	0.004288489	path:04612_3
spleen	CREB1	0.004131401	path:04620_12
spleen	CREB1	0.004131401	path:04620_16
spleen	CREB1	0.001277819	path:04620_17
spleen	CREB1	0.000627719	path:04620_18
spleen	CREB1	0.001277819	path:04620_22
spleen	CREB1	0.001277819	path:04620_9
spleen	CREB1	0.004131401	path:04672_4
spleen	CREB1	0.004131401	path:04672_5
spleen	CREB1	0.004131401	path:04940_1
spleen	CREB1	0.004288489	path:05150_14
spleen	CREB1	0.009649083	path:05200_18
spleen	CREB1	0.004131401	path:05310_1

spleen	CREB1	0.004131401	path:05310_3
spleen	CREB1	0.004131401	path:05320_1
spleen	CREB1	0.004131401	path:05320_3
spleen	CREB1	0.004131401	path:05320_4
spleen	CREB1	0.004131401	path:05320_6
spleen	CREB1	0.004131401	path:05322_5
spleen	CREB1	0.004131401	path:05322_6
spleen	CREB1	0.004131401	path:05330_1
spleen	CREB1	0.004131401	path:05330_2
spleen	CREB1	0.004131401	path:05330_4
spleen	CREB1	0.004131401	path:05332_1
spleen	CREB1	0.004131401	path:05416_4
spleen	ELF1	8.41E-06	path:04060_8
spleen	ELF1	0.006320724	path:04650_1
spleen	ELF1	0.001225713	path:04650_14
spleen	ELF1	0.000687264	path:04650_15
spleen	ELF1	0.000687264	path:04664_1
spleen	ELF1	0.000533253	path:04664_10
spleen	ELF1	1.05E-05	path:04664_2
spleen	ELF1	0.000533253	path:04664_3
spleen	ELF1	0.000533253	path:04664_5
spleen	ELF1	0.000533253	path:04664_9
spleen	ELF1	0.006320724	path:05142_21
spleen	ELF1	0.000687264	path:05310_4
spleen	ELF1	0.000687264	path:05310_5
spleen	HNF1A	0.008512637	path:00120_5
spleen	HNF1A	0.008512637	path:00140_1
spleen	HNF1A	3.95E-05	path:00140_12
spleen	HNF1A	0.008512637	path:00140_22
spleen	HNF1A	0.008512637	path:00140_23
spleen	HNF1A	0.008512637	path:00140_24
spleen	HNF1A	0.008512637	path:00140_25
spleen	HNF1A	0.008512637	path:00140_26
spleen	HNF1A	0.008512637	path:00982_6

spleen	HNF1A	0.008512637	path:04610_6
spleen	IRF5	0.005011517	path:04060_38
spleen	IRF5	0.005011517	path:04620_11
spleen	IRF5	0.005011517	path:04620_2
spleen	IRF5	0.005011517	path:04650_6
spleen	IRF5	0.005011517	path:05160_6
spleen	IRF7	5.44E-05	path:04060_38
spleen	IRF7	3.65E-07	path:04620_11
spleen	IRF7	1.24E-05	path:04620_12
spleen	IRF7	0.005131788	path:04620_14
spleen	IRF7	0.005500251	path:04620_16
spleen	IRF7	0.009380523	path:04620_17
spleen	IRF7	0.005305274	path:04620_18
spleen	IRF7	3.65E-07	path:04620_2
spleen	IRF7	0.009380523	path:04620_22
spleen	IRF7	0.009501335	path:04620_9
spleen	IRF7	5.75E-05	path:04650_6
spleen	IRF7	5.75E-05	path:05160_6
spleen	SPI1	0.00153244	path:04060_41
spleen	SPI1	0.005348786	path:04145_2
spleen	SPI1	3.11E-06	path:04145_6
spleen	SPI1	0.007630608	path:04514_55
spleen	SPI1	0.003570772	path:04514_62
spleen	SPI1	0.008430928	path:04650_10
spleen	SPI1	0.007340609	path:04650_12
spleen	SPI1	0.003805584	path:04650_13
spleen	SPI1	0.003570772	path:04670_10
spleen	SPI1	0.00153244	path:04670_14
spleen	SPI1	0.000961365	path:04670_15
spleen	SPI1	0.000252274	path:04670_2
spleen	SPI1	0.000110411	path:05140_4
spleen	SPI1	0.005348786	path:05150_17
spleen	SPI1	0.000902646	path:05200_43
stomach	HNF1A	0.008512637	path:00120_5

stomach	HNF1A	0.008512637	path:00140_1
stomach	HNF1A	3.95E-05	path:00140_12
stomach	HNF1A	0.008512637	path:00140_22
stomach	HNF1A	0.008512637	path:00140_23
stomach	HNF1A	0.008512637	path:00140_24
stomach	HNF1A	0.008512637	path:00140_25
stomach	HNF1A	0.008512637	path:00140_26
stomach	HNF1A	0.008512637	path:00982_6
stomach	HNF1A	0.008512637	path:04610_6
T cells	AKNA	6.20E-07	path:04060_31
T cells	AKNA	6.20E-07	path:04514_10
T cells	AKNA	6.20E-07	path:04514_11
T cells	AKNA	6.20E-07	path:04514_12
T cells	AKNA	6.20E-07	path:04514_78
T cells	AKNA	6.20E-07	path:04514_9
T cells	AKNA	6.20E-07	path:04672_2
T cells	AKNA	6.20E-07	path:05144_9
T cells	AKNA	6.20E-07	path:05145_20
T cells	AKNA	6.20E-07	path:05310_2
T cells	AKNA	6.20E-07	path:05320_2
T cells	AKNA	6.20E-07	path:05320_5
T cells	AKNA	6.20E-07	path:05322_7
T cells	AKNA	6.20E-07	path:05330_12
T cells	AKNA	6.20E-07	path:05330_3
T cells	AKNA	6.20E-07	path:05416_3
T cells	CREM	0.000820491	path:05200_29
T cells	EGR1	0.009869535	path:04060_8
T cells	EGR1	0.00069989	path:04510_10
T cells	EGR1	0.00069989	path:04510_18
T cells	EGR1	0.003153788	path:04510_6
T cells	EGR1	0.003153788	path:04510_8
T cells	EGR1	0.00129686	path:04512_3
T cells	EGR1	0.003153788	path:04540_13
T cells	EGR1	0.003153788	path:05146_9

T cells	EGR1	0.007008495	path:05212_11
T cells	EGR1	0.008365691	path:05212_12
T cells	EGR1	0.003153788	path:05218_4
T cells	GATA3	0.003994597	path:04612_4
T cells	LEF1	0.000671935	path:04310_6
T cells	LEF1	0.000118706	path:04310_7
T cells	LEF1	0.00555092	path:04916_11
T cells	LEF1	0.002645905	path:04916_3
T cells	LEF1	0.007825657	path:04916_8
T cells	LEF1	1.30E-05	path:05200_31
T cells	LEF1	6.00E-05	path:05200_42
T cells	LEF1	0.003595279	path:05200_50
T cells	LEF1	0.002645905	path:05210_10
T cells	LEF1	1.30E-05	path:05210_12
T cells	LEF1	1.30E-05	path:05210_7
T cells	LEF1	2.72E-05	path:05213_1
T cells	LEF1	0.003117148	path:05215_4
T cells	LEF1	1.30E-05	path:05216_4
T cells	LEF1	5.77E-05	path:05221_1
T cells	LEF1	0.005057313	path:05222_1
T cells	LEF1	0.004566365	path:05222_2
T cells	LEF1	0.004078733	path:05222_7
T cells	NFATC1	0.003964221	path:04060_32
T cells	NFATC1	0.003964221	path:04060_33
T cells	NFATC1	0.003964221	path:04650_20
T cells	SPI1	0.00153244	path:04060_41
T cells	SPI1	0.005348786	path:04145_2
T cells	SPI1	3.11E-06	path:04145_6
T cells	SPI1	0.007630608	path:04514_55
T cells	SPI1	0.003570772	path:04514_62
T cells	SPI1	0.008430928	path:04650_10
T cells	SPI1	0.007340609	path:04650_12
T cells	SPI1	0.003805584	path:04650_13
T cells	SPI1	0.003570772	path:04670_10

T cells	SPI1	0.00153244	path:04670_14
T cells	SPI1	0.000961365	path:04670_15
T cells	SPI1	0.000252274	path:04670_2
T cells	SPI1	0.000110411	path:05140_4
T cells	SPI1	0.005348786	path:05150_17
T cells	SPI1	0.000902646	path:05200_43
T cells	TCF7	0.00669167	path:04660_20
T47D	ESR1	0.005413618	path:00830_2
T47D	ESR1	0.000345486	path:04115_1
T47D	ESR1	0.002979325	path:04115_2
T47D	ESR1	0.002948202	path:04115_3
T47D	ESR1	0.002948202	path:04115_4
T47D	ESR1	0.002948202	path:04115_7
T47D	ESR1	0.002948202	path:04610_8
T47D	ESR1	0.000345486	path:05200_29
T47D	NFIC	0.004396514	path:00591_1
T47D	NFIC	0.005988829	path:00982_11
T47D	NFIC	0.00921451	path:00982_2
T47D	NFIC	0.005988829	path:00982_3
T47D	NFIC	0.00787147	path:00982_4
T47D	NFIC	0.00787147	path:00982_5
T47D	NFIC	0.004396514	path:00982_7
T47D	NFIC	0.004396514	path:00982_8
T47D	NFIC	0.00787147	path:00982_9
T47D	NFIC	0.00787147	path:04010_30
T47D	NFIC	0.00787147	path:04010_31
T47D	NFIC	0.004396514	path:04080_7
T47D	NFIC	0.008000492	path:04115_1
T47D	NFIC	0.00787147	path:04115_2
T47D	NFIC	0.00787147	path:04115_3
T47D	NFIC	0.00787147	path:04115_4
T47D	NFIC	0.00787147	path:04115_7
T47D	NFIC	0.00787147	path:04660_8
T47D	NFIC	0.00787147	path:04662_6

T47D	NFIC	0.00787147	path:04722_19
T47D	NFIC	0.00787147	path:05160_15
T47D	NFIC	0.007050709	path:05200_47
T47D	NFIC	0.004396514	path:05200_48
T47D	NFIC	0.00787147	path:05200_51
T47D	NFIC	0.004396514	path:05214_12
T47D	NFIC	0.00787147	path:05214_13
T47D	NFIC	0.007050709	path:05214_14
T47D	NFIC	0.004396514	path:05218_5
T47D	NFIC	0.004396514	path:05218_6
T47D	NFIC	0.00787147	path:05220_12
T47D	NFIC	0.004396514	path:05220_8
T47D	NFIC	0.007050709	path:05220_9
T47D	SP1	0.006087664	path:00120_1
T47D	SP1	0.003885876	path:00120_2
T47D	SP1	0.003885876	path:00120_3
T47D	SP1	0.001893526	path:00140_1
T47D	SP1	1.44E-05	path:00140_10
T47D	SP1	0.001534931	path:00140_11
T47D	SP1	0.006087664	path:00140_12
T47D	SP1	2.77E-05	path:00140_13
T47D	SP1	0.003885876	path:00140_14
T47D	SP1	0.000245716	path:00140_16
T47D	SP1	0.000245716	path:00140_18
T47D	SP1	2.77E-05	path:00140_19
T47D	SP1	0.000441307	path:00140_20
T47D	SP1	0.001893526	path:00140_25
T47D	SP1	1.44E-05	path:00140_3
T47D	SP1	1.44E-05	path:00140_4
T47D	SP1	5.07E-05	path:00140_5
T47D	SP1	8.66E-05	path:00140_6
T47D	SP1	2.77E-05	path:00140_7
T47D	SP1	0.000123921	path:00140_8
T47D	SP1	0.000123921	path:00140_9

T47D	SP1	0.005536058	path:00240_16
T47D	SP1	0.003391309	path:00240_17
T47D	SP1	0.001690362	path:00240_2
T47D	SP1	0.009002988	path:00240_5
T47D	SP1	0.005536058	path:00330_1
T47D	SP1	0.005126386	path:00330_9
T47D	SP1	0.006192277	path:00350_2
T47D	SP1	0.006192277	path:00350_3
T47D	SP1	0.001393247	path:00980_2
T47D	SP1	0.002175912	path:00982_10
T47D	SP1	0.003931316	path:04010_10
T47D	SP1	0.000441307	path:04010_26
T47D	SP1	0.000123921	path:04010_5
T47D	SP1	0.002149604	path:04060_22
T47D	SP1	0.001014956	path:04060_32
T47D	SP1	0.007433507	path:04060_47
T47D	SP1	0.00916136	path:04110_1
T47D	SP1	1.86E-10	path:04110_17
T47D	SP1	4.28E-11	path:04110_18
T47D	SP1	1.05E-10	path:04110_19
T47D	SP1	2.92E-09	path:04110_20
T47D	SP1	1.43E-05	path:04110_22
T47D	SP1	5.22E-09	path:04110_23
T47D	SP1	0.006192277	path:04110_25
T47D	SP1	7.70E-08	path:04110_26
T47D	SP1	2.92E-09	path:04110_27
T47D	SP1	6.46E-09	path:04110_3
T47D	SP1	6.46E-09	path:04110_4
T47D	SP1	4.97E-07	path:04110_7
T47D	SP1	3.47E-10	path:04115_1
T47D	SP1	6.74E-05	path:04115_2
T47D	SP1	4.23E-05	path:04115_3
T47D	SP1	6.18E-06	path:04115_4
T47D	SP1	4.96E-05	path:04115_7

T47D	SP1	7.61E-06	path:04144_2
T47D	SP1	0.002175912	path:04210_20
T47D	SP1	0.001107767	path:04210_24
T47D	SP1	1.33E-05	path:04310_6
T47D	SP1	1.77E-05	path:04310_7
T47D	SP1	7.85E-13	path:04510_1
T47D	SP1	1.05E-10	path:04510_10
T47D	SP1	0.001603207	path:04510_13
T47D	SP1	0.001164758	path:04510_15
T47D	SP1	0.002771235	path:04510_16
T47D	SP1	0.000141426	path:04510_17
T47D	SP1	8.07E-08	path:04510_18
T47D	SP1	1.96E-12	path:04510_19
T47D	SP1	1.43E-05	path:04510_20
T47D	SP1	0.000718519	path:04510_23
T47D	SP1	0.002175912	path:04510_24
T47D	SP1	0.0024986	path:04510_25
T47D	SP1	6.74E-05	path:04510_4
T47D	SP1	0.0024986	path:04510_5
T47D	SP1	1.07E-10	path:04510_6
T47D	SP1	0.000448433	path:04510_7
T47D	SP1	6.16E-10	path:04510_8
T47D	SP1	2.01E-05	path:04512_10
T47D	SP1	5.55E-06	path:04512_11
T47D	SP1	1.66E-06	path:04512_12
T47D	SP1	7.86E-06	path:04512_13
T47D	SP1	0.000101569	path:04512_14
T47D	SP1	0.000245716	path:04512_18
T47D	SP1	0.000549822	path:04512_19
T47D	SP1	0.002149604	path:04512_20
T47D	SP1	0.008608739	path:04512_22
T47D	SP1	0.00016592	path:04512_27
T47D	SP1	1.66E-05	path:04512_3
T47D	SP1	2.72E-07	path:04512_5

T47D	SP1	6.24E-07	path:04512_6
T47D	SP1	9.36E-06	path:04512_9
T47D	SP1	0.005126386	path:04520_3
T47D	SP1	0.001603207	path:04520_5
T47D	SP1	0.000412181	path:04520_7
T47D	SP1	1.22E-05	path:04540_13
T47D	SP1	9.21E-07	path:04610_3
T47D	SP1	5.55E-06	path:04610_4
T47D	SP1	9.21E-07	path:04610_6
T47D	SP1	0.000133832	path:04610_7
T47D	SP1	0.000203885	path:04610_8
T47D	SP1	0.000203885	path:04620_18
T47D	SP1	0.001893526	path:04620_9
T47D	SP1	0.000231547	path:04630_1
T47D	SP1	0.001390056	path:04630_2
T47D	SP1	1.97E-06	path:04630_3
T47D	SP1	5.95E-06	path:04630_4
T47D	SP1	4.84E-05	path:04650_20
T47D	SP1	0.008608739	path:04650_7
T47D	SP1	0.000718519	path:04722_19
T47D	SP1	7.92E-07	path:04810_29
T47D	SP1	1.33E-05	path:04912_8
T47D	SP1	0.007433507	path:04940_4
T47D	SP1	0.006087664	path:05014_3
T47D	SP1	0.008608739	path:05014_4
T47D	SP1	0.007433507	path:05016_8
T47D	SP1	0.001014956	path:05100_3
T47D	SP1	0.008608739	path:05140_10
T47D	SP1	0.000245716	path:05140_6
T47D	SP1	0.001107767	path:05142_10
T47D	SP1	0.003931316	path:05142_11
T47D	SP1	0.003885876	path:05142_17
T47D	SP1	0.003885876	path:05142_18
T47D	SP1	0.000441307	path:05142_20

T47D	SP1	0.000718519	path:05142_21
T47D	SP1	0.001107767	path:05142_4
T47D	SP1	0.006087664	path:05145_19
T47D	SP1	0.001014956	path:05145_21
T47D	SP1	0.006087664	path:05145_8
T47D	SP1	1.23E-06	path:05146_1
T47D	SP1	7.92E-07	path:05146_2
T47D	SP1	0.002149604	path:05146_5
T47D	SP1	9.10E-07	path:05146_9
T47D	SP1	0.003935354	path:05200_19
T47D	SP1	0.003935354	path:05200_20
T47D	SP1	0.00927462	path:05200_24
T47D	SP1	9.21E-07	path:05200_29
T47D	SP1	2.01E-05	path:05200_3
T47D	SP1	0.000718519	path:05200_31
T47D	SP1	0.008608739	path:05200_38
T47D	SP1	0.000289957	path:05200_4
T47D	SP1	0.000718519	path:05200_42
T47D	SP1	2.07E-08	path:05200_47
T47D	SP1	1.33E-05	path:05200_48
T47D	SP1	5.07E-05	path:05200_50
T47D	SP1	5.54E-09	path:05200_51
T47D	SP1	1.55E-08	path:05200_52
T47D	SP1	4.11E-06	path:05200_53
T47D	SP1	0.001014956	path:05200_56
T47D	SP1	0.000441307	path:05210_10
T47D	SP1	0.000718519	path:05210_12
T47D	SP1	0.000718519	path:05210_7
T47D	SP1	1.44E-05	path:05212_13
T47D	SP1	0.008608739	path:05212_14
T47D	SP1	1.33E-05	path:05214_12
T47D	SP1	1.18E-07	path:05214_13
T47D	SP1	2.07E-08	path:05214_14
T47D	SP1	9.21E-07	path:05214_15

T47D	SP1	0.005536058	path:05214_2
T47D	SP1	0.000245716	path:05214_21
T47D	SP1	0.005536058	path:05214_5
T47D	SP1	0.001164758	path:05215_10
T47D	SP1	0.008608739	path:05215_7
T47D	SP1	0.000441307	path:05216_4
T47D	SP1	0.000411461	path:05218_4
T47D	SP1	0.000133832	path:05218_5
T47D	SP1	1.33E-05	path:05218_6
T47D	SP1	9.21E-07	path:05218_7
T47D	SP1	7.27E-05	path:05218_8
T47D	SP1	0.000310459	path:05219_3
T47D	SP1	1.44E-05	path:05219_4
T47D	SP1	9.21E-07	path:05220_11
T47D	SP1	1.18E-07	path:05220_12
T47D	SP1	0.002149604	path:05220_5
T47D	SP1	1.33E-05	path:05220_8
T47D	SP1	2.07E-08	path:05220_9
T47D	SP1	0.006192277	path:05221_1
T47D	SP1	5.36E-07	path:05222_1
T47D	SP1	0.000133832	path:05222_2
T47D	SP1	2.01E-05	path:05222_4
T47D	SP1	0.001603207	path:05222_7
T47D	SP1	1.44E-05	path:05223_3
T47D	SP1	0.007433507	path:05320_9
T47D	SP1	0.007433507	path:05330_5
T47D	SP1	0.007433507	path:05332_2
T47D	SP1	0.007433507	path:05332_5
T47D	SP1	1.77E-05	path:05414_2
T98	NFIC	0.004396514	path:00591_1
T98	NFIC	0.005988829	path:00982_11
T98	NFIC	0.00921451	path:00982_2
T98	NFIC	0.005988829	path:00982_3
T98	NFIC	0.00787147	path:00982_4

T98	NFIC	0.00787147	path:00982_5
T98	NFIC	0.004396514	path:00982_7
T98	NFIC	0.004396514	path:00982_8
T98	NFIC	0.00787147	path:00982_9
T98	NFIC	0.00787147	path:04010_30
T98	NFIC	0.00787147	path:04010_31
T98	NFIC	0.004396514	path:04080_7
T98	NFIC	0.008000492	path:04115_1
T98	NFIC	0.00787147	path:04115_2
T98	NFIC	0.00787147	path:04115_3
T98	NFIC	0.00787147	path:04115_4
T98	NFIC	0.00787147	path:04115_7
T98	NFIC	0.00787147	path:04660_8
T98	NFIC	0.00787147	path:04662_6
T98	NFIC	0.00787147	path:04722_19
T98	NFIC	0.00787147	path:05160_15
T98	NFIC	0.007050709	path:05200_47
T98	NFIC	0.004396514	path:05200_48
T98	NFIC	0.00787147	path:05200_51
T98	NFIC	0.004396514	path:05214_12
T98	NFIC	0.00787147	path:05214_13
T98	NFIC	0.007050709	path:05214_14
T98	NFIC	0.004396514	path:05218_5
T98	NFIC	0.004396514	path:05218_6
T98	NFIC	0.00787147	path:05220_12
T98	NFIC	0.004396514	path:05220_8
T98	NFIC	0.007050709	path:05220_9
testis	AKNA	6.20E-07	path:04060_31
testis	AKNA	6.20E-07	path:04514_10
testis	AKNA	6.20E-07	path:04514_11
testis	AKNA	6.20E-07	path:04514_12
testis	AKNA	6.20E-07	path:04514_78
testis	AKNA	6.20E-07	path:04514_9
testis	AKNA	6.20E-07	path:04672_2

testis	AKNA	6.20E-07	path:05144_9
testis	AKNA	6.20E-07	path:05145_20
testis	AKNA	6.20E-07	path:05310_2
testis	AKNA	6.20E-07	path:05320_2
testis	AKNA	6.20E-07	path:05320_5
testis	AKNA	6.20E-07	path:05322_7
testis	AKNA	6.20E-07	path:05330_12
testis	AKNA	6.20E-07	path:05330_3
testis	AKNA	6.20E-07	path:05416_3
testis	CREB1	0.004288489	path:04514_1
testis	CREB1	0.004288489	path:04514_3
testis	CREB1	0.004288489	path:04612_3
testis	CREB1	0.004131401	path:04620_12
testis	CREB1	0.004131401	path:04620_16
testis	CREB1	0.001277819	path:04620_17
testis	CREB1	0.000627719	path:04620_18
testis	CREB1	0.001277819	path:04620_22
testis	CREB1	0.001277819	path:04620_9
testis	CREB1	0.004131401	path:04672_4
testis	CREB1	0.004131401	path:04672_5
testis	CREB1	0.004131401	path:04940_1
testis	CREB1	0.004288489	path:05150_14
testis	CREB1	0.009649083	path:05200_18
testis	CREB1	0.004131401	path:05310_1
testis	CREB1	0.004131401	path:05310_3
testis	CREB1	0.004131401	path:05320_1
testis	CREB1	0.004131401	path:05320_3
testis	CREB1	0.004131401	path:05320_4
testis	CREB1	0.004131401	path:05320_6
testis	CREB1	0.004131401	path:05322_5
testis	CREB1	0.004131401	path:05322_6
testis	CREB1	0.004131401	path:05330_1
testis	CREB1	0.004131401	path:05330_2
testis	CREB1	0.004131401	path:05330_4

testis	CREB1	0.004131401	path:05332_1
testis	CREB1	0.004131401	path:05416_4
testis	HNF4A	0.00730316	path:00120_15
testis	HNF4A	0.003812707	path:00120_19
testis	HNF4A	0.001698537	path:00120_6
testis	HNF4A	0.003812707	path:00120_7
testis	HNF4A	0.000100826	path:00120_9
testis	HNF4A	0.001808381	path:00590_1
testis	HNF4A	0.001722691	path:00590_2
testis	HNF4A	0.001722691	path:00590_3
testis	HNF4A	0.001898001	path:00590_4
testis	HNF4A	0.000515682	path:00591_1
testis	HNF4A	2.36E-05	path:00830_2
testis	HNF4A	0.000733563	path:00980_2
testis	HNF4A	2.52E-07	path:00982_10
testis	HNF4A	5.38E-08	path:00982_4
testis	HNF4A	5.38E-08	path:00982_5
testis	HNF4A	5.38E-08	path:00982_9
testis	HNF4A	0.001131883	path:03320_1
testis	HNF4A	0.000100826	path:03320_2
testis	HNF4A	4.35E-06	path:04610_2
testis	HNF4A	4.35E-06	path:04610_3
testis	HNF4A	0.000100826	path:04610_4
testis	HNF4A	6.69E-06	path:04610_5
testis	HNF4A	0.000179282	path:04610_6
testis	HNF4A	0.001698537	path:04610_8
testis	HNF4A	0.000287791	path:04950_4
testis	KLF5	0.000357162	path:05200_31
testis	KLF5	0.000357162	path:05210_12
testis	KLF5	0.000357162	path:05210_7
testis	SPI1	0.00153244	path:04060_41
testis	SPI1	0.005348786	path:04145_2
testis	SPI1	3.11E-06	path:04145_6
testis	SPI1	0.007630608	path:04514_55

testis	SPI1	0.003570772	path:04514_62
testis	SPI1	0.008430928	path:04650_10
testis	SPI1	0.007340609	path:04650_12
testis	SPI1	0.003805584	path:04650_13
testis	SPI1	0.003570772	path:04670_10
testis	SPI1	0.00153244	path:04670_14
testis	SPI1	0.000961365	path:04670_15
testis	SPI1	0.000252274	path:04670_2
testis	SPI1	0.000110411	path:05140_4
testis	SPI1	0.005348786	path:05150_17
testis	SPI1	0.000902646	path:05200_43
testis	TP53	0.007702858	path:04010_15
testis	TP53	0.009062653	path:04010_16
testis	TP53	0.007443002	path:04010_21
testis	TP53	0.002466645	path:04012_12
testis	TP53	2.55E-11	path:04110_1
testis	TP53	3.28E-07	path:04110_17
testis	TP53	1.48E-05	path:04110_19
testis	TP53	0.001943417	path:04110_20
testis	TP53	7.56E-05	path:04110_22
testis	TP53	0.000198814	path:04110_23
testis	TP53	0.001943417	path:04110_27
testis	TP53	0.000210553	path:04110_3
testis	TP53	0.004511216	path:04110_4
testis	TP53	4.42E-09	path:04110_7
testis	TP53	0.004291125	path:04110_8
testis	TP53	0	path:04115_1
testis	TP53	0	path:04115_2
testis	TP53	0	path:04115_3
testis	TP53	0	path:04115_4
testis	TP53	0	path:04115_7
testis	TP53	0.000421718	path:04210_20
testis	TP53	0.000275763	path:04210_23
testis	TP53	0.000275763	path:04210_24

testis	TP53	0.009131774	path:04510_10
testis	TP53	0.004511216	path:04510_15
testis	TP53	0.002466645	path:04510_16
testis	TP53	0.000580605	path:04510_17
testis	TP53	0.001774645	path:04510_18
testis	TP53	0.006951959	path:04510_25
testis	TP53	0.006951959	path:04510_5
testis	TP53	0.009576653	path:04510_6
testis	TP53	0.001110697	path:04510_7
testis	TP53	0.00935253	path:04510_8
testis	TP53	0.009062653	path:04621_7
testis	TP53	0.000218217	path:04722_19
testis	TP53	0.002756828	path:04912_8
testis	TP53	0.009062653	path:05014_4
testis	TP53	0.007459365	path:05160_12
testis	TP53	0.009062653	path:05160_15
testis	TP53	0.000338058	path:05200_10
testis	TP53	0.000275763	path:05200_11
testis	TP53	0.000275763	path:05200_12
testis	TP53	0.000968321	path:05200_15
testis	TP53	0.00392722	path:05200_17
testis	TP53	0.002997803	path:05200_19
testis	TP53	0.00011707	path:05200_20
testis	TP53	0.000275763	path:05200_4
testis	TP53	7.56E-05	path:05200_47
testis	TP53	1.95E-05	path:05200_48
testis	TP53	0.000367024	path:05200_5
testis	TP53	0.000172314	path:05200_51
testis	TP53	1.95E-05	path:05200_8
testis	TP53	0.007459365	path:05213_2
testis	TP53	0.003580594	path:05214_1
testis	TP53	0.000210553	path:05214_10
testis	TP53	0.001774645	path:05214_11
testis	TP53	1.95E-05	path:05214_12

testis	TP53	0.00011707	path:05214_13
testis	TP53	7.56E-05	path:05214_14
testis	TP53	0.003580594	path:05214_4
testis	TP53	0.004511216	path:05215_10
testis	TP53	0.000275763	path:05215_11
testis	TP53	0.00331496	path:05215_3
testis	TP53	0.002754132	path:05218_2
testis	TP53	0.001774645	path:05218_5
testis	TP53	1.95E-05	path:05218_6
testis	TP53	0.001385048	path:05218_8
testis	TP53	0.001691972	path:05219_3
testis	TP53	0.009062653	path:05219_4
testis	TP53	0.00011707	path:05220_12
testis	TP53	1.95E-05	path:05220_8
testis	TP53	7.56E-05	path:05220_9
thymus	AKNA	6.20E-07	path:04060_31
thymus	AKNA	6.20E-07	path:04514_10
thymus	AKNA	6.20E-07	path:04514_11
thymus	AKNA	6.20E-07	path:04514_12
thymus	AKNA	6.20E-07	path:04514_78
thymus	AKNA	6.20E-07	path:04514_9
thymus	AKNA	6.20E-07	path:04672_2
thymus	AKNA	6.20E-07	path:05144_9
thymus	AKNA	6.20E-07	path:05145_20
thymus	AKNA	6.20E-07	path:05310_2
thymus	AKNA	6.20E-07	path:05320_2
thymus	AKNA	6.20E-07	path:05320_5
thymus	AKNA	6.20E-07	path:05322_7
thymus	AKNA	6.20E-07	path:05330_12
thymus	AKNA	6.20E-07	path:05330_3
thymus	AKNA	6.20E-07	path:05416_3
thymus	CREB1	0.004288489	path:04514_1
thymus	CREB1	0.004288489	path:04514_3
thymus	CREB1	0.004288489	path:04612_3

thymus	CREB1	0.004131401	path:04620_12
thymus	CREB1	0.004131401	path:04620_16
thymus	CREB1	0.001277819	path:04620_17
thymus	CREB1	0.000627719	path:04620_18
thymus	CREB1	0.001277819	path:04620_22
thymus	CREB1	0.001277819	path:04620_9
thymus	CREB1	0.004131401	path:04672_4
thymus	CREB1	0.004131401	path:04672_5
thymus	CREB1	0.004131401	path:04940_1
thymus	CREB1	0.004288489	path:05150_14
thymus	CREB1	0.009649083	path:05200_18
thymus	CREB1	0.004131401	path:05310_1
thymus	CREB1	0.004131401	path:05310_3
thymus	CREB1	0.004131401	path:05320_1
thymus	CREB1	0.004131401	path:05320_3
thymus	CREB1	0.004131401	path:05320_4
thymus	CREB1	0.004131401	path:05320_6
thymus	CREB1	0.004131401	path:05322_5
thymus	CREB1	0.004131401	path:05322_6
thymus	CREB1	0.004131401	path:05330_1
thymus	CREB1	0.004131401	path:05330_2
thymus	CREB1	0.004131401	path:05330_4
thymus	CREB1	0.004131401	path:05332_1
thymus	CREB1	0.004131401	path:05416_4
thymus	ELF1	8.41E-06	path:04060_8
thymus	ELF1	0.006320724	path:04650_1
thymus	ELF1	0.001225713	path:04650_14
thymus	ELF1	0.000687264	path:04650_15
thymus	ELF1	0.000687264	path:04664_1
thymus	ELF1	0.000533253	path:04664_10
thymus	ELF1	1.05E-05	path:04664_2
thymus	ELF1	0.000533253	path:04664_3
thymus	ELF1	0.000533253	path:04664_5
thymus	ELF1	0.000533253	path:04664_9

thymus	ELF1	0.006320724	path:05142_21
thymus	ELF1	0.000687264	path:05310_4
thymus	ELF1	0.000687264	path:05310_5
thymus	IRF5	0.005011517	path:04060_38
thymus	IRF5	0.005011517	path:04620_11
thymus	IRF5	0.005011517	path:04620_2
thymus	IRF5	0.005011517	path:04650_6
thymus	IRF5	0.005011517	path:05160_6
thymus	IRF7	5.44E-05	path:04060_38
thymus	IRF7	3.65E-07	path:04620_11
thymus	IRF7	1.24E-05	path:04620_12
thymus	IRF7	0.005131788	path:04620_14
thymus	IRF7	0.005500251	path:04620_16
thymus	IRF7	0.009380523	path:04620_17
thymus	IRF7	0.005305274	path:04620_18
thymus	IRF7	3.65E-07	path:04620_2
thymus	IRF7	0.009380523	path:04620_22
thymus	IRF7	0.009501335	path:04620_9
thymus	IRF7	5.75E-05	path:04650_6
thymus	IRF7	5.75E-05	path:05160_6
thymus	LEF1	0.000671935	path:04310_6
thymus	LEF1	0.000118706	path:04310_7
thymus	LEF1	0.00555092	path:04916_11
thymus	LEF1	0.002645905	path:04916_3
thymus	LEF1	0.007825657	path:04916_8
thymus	LEF1	1.30E-05	path:05200_31
thymus	LEF1	6.00E-05	path:05200_42
thymus	LEF1	0.003595279	path:05200_50
thymus	LEF1	0.002645905	path:05210_10
thymus	LEF1	1.30E-05	path:05210_12
thymus	LEF1	1.30E-05	path:05210_7
thymus	LEF1	2.72E-05	path:05213_1
thymus	LEF1	0.003117148	path:05215_4
thymus	LEF1	1.30E-05	path:05216_4

thymus	LEF1	5.77E-05	path:05221_1
thymus	LEF1	0.005057313	path:05222_1
thymus	LEF1	0.004566365	path:05222_2
thymus	LEF1	0.004078733	path:05222_7
thymus	SPI1	0.00153244	path:04060_41
thymus	SPI1	0.005348786	path:04145_2
thymus	SPI1	3.11E-06	path:04145_6
thymus	SPI1	0.007630608	path:04514_55
thymus	SPI1	0.003570772	path:04514_62
thymus	SPI1	0.008430928	path:04650_10
thymus	SPI1	0.007340609	path:04650_12
thymus	SPI1	0.003805584	path:04650_13
thymus	SPI1	0.003570772	path:04670_10
thymus	SPI1	0.00153244	path:04670_14
thymus	SPI1	0.000961365	path:04670_15
thymus	SPI1	0.000252274	path:04670_2
thymus	SPI1	0.000110411	path:05140_4
thymus	SPI1	0.005348786	path:05150_17
thymus	SPI1	0.000902646	path:05200_43
thymus	TP63	2.23E-05	path:04110_1
thymus	TP63	8.12E-05	path:04110_7
thymus	TP63	8.12E-05	path:04115_1
thymus	TP63	8.12E-05	path:04115_2
thymus	TP63	8.12E-05	path:04115_3
thymus	TP63	8.12E-05	path:04115_4
thymus	TP63	8.12E-05	path:04115_7
thymus	TP63	0.003115907	path:05200_17
thymus	TP63	0.002783081	path:05200_19
thymus	TP63	0.002783081	path:05200_20
thymus	TP63	0.000159403	path:05200_47
thymus	TP63	8.12E-05	path:05200_48
thymus	TP63	8.12E-05	path:05214_12
thymus	TP63	0.000159403	path:05214_14
thymus	TP63	0.004120782	path:05215_11

thymus	TP63	0.002837304	path:05215_3
thymus	TP63	8.12E-05	path:05218_6
thymus	TP63	8.12E-05	path:05220_8
thymus	TP63	0.000159403	path:05220_9
thyroid	HNF1A	0.008512637	path:00120_5
thyroid	HNF1A	0.008512637	path:00140_1
thyroid	HNF1A	3.95E-05	path:00140_12
thyroid	HNF1A	0.008512637	path:00140_22
thyroid	HNF1A	0.008512637	path:00140_23
thyroid	HNF1A	0.008512637	path:00140_24
thyroid	HNF1A	0.008512637	path:00140_25
thyroid	HNF1A	0.008512637	path:00140_26
thyroid	HNF1A	0.008512637	path:00982_6
thyroid	HNF1A	0.008512637	path:04610_6
thyroid	NR4A1	0.00077713	path:00140_10
thyroid	NR4A1	6.42E-06	path:00140_13
thyroid	NR4A1	0.00077713	path:00140_14
thyroid	NR4A1	0.00077713	path:00140_16
thyroid	NR4A1	0.00077713	path:00140_18
thyroid	NR4A1	0.000832486	path:00140_19
thyroid	NR4A1	0.00077713	path:00140_20
thyroid	NR4A1	0.00077713	path:00140_3
thyroid	NR4A1	0.00077713	path:00140_4
thyroid	NR4A1	0.000960385	path:00140_5
thyroid	NR4A1	0.001089761	path:00140_6
thyroid	NR4A1	0.000832486	path:00140_7
thyroid	NR4A1	0.00077713	path:00140_8
thyroid	NR4A1	0.00077713	path:00140_9

Table S6. The detailed information of the significant TF-subpathway associations between TFs and subpathways in the TSN with k=4.

TF_Name	Subpathway	P_Value
SP1	path:04510_5	0
SP1	path:04510_6	0
SP1	path:04510_7	0
SP1	path:04510_13	0
SP1	path:05200_15	0
SP1	path:05200_25	0
SP1	path:05200_37	0
SP1	path:05200_38	0
SP1	path:05200_39	0
TP53	path:04115_2	0
SP1	path:05200_35	2.28E-14
SP1	path:04512_3	7.27E-14
SP1	path:04110_13	6.38E-13
SP1	path:04110_10	7.65E-13
SP1	path:04110_11	1.26E-12
SP1	path:04512_1	1.42E-12
SP1	path:05200_21	5.41E-12
IRF3	path:04620_3	6.11E-12
IRF3	path:04620_1	6.11E-12
IRF7	path:04620_3	6.11E-12
IRF7	path:04620_1	6.11E-12
SP1	path:04510_14	6.46E-12
SP1	path:04510_1	7.27E-12
SP1	path:04110_9	1.26E-11
IRF3	path:04620_4	1.29E-11
IRF7	path:04620_4	1.29E-11
SP1	path:04115_2	3.58E-11
SP1	path:04512_2	3.58E-11
SP1	path:05200_7	8.22E-11
SP1	path:05200_2	1.66E-10
TP53	path:04110_2	2.00E-10
SP1	path:04110_5	2.09E-10
SP1	path:05200_26	2.35E-10
SP1	path:05200_13	2.35E-10
SP1	path:05200_24	3.40E-10
IRF1	path:04620_4	3.47E-10
RELA	path:04620_4	3.88E-10
STAT3	path:05200_25	4.16E-10
E2F1	path:04110_11	4.74E-10
E2F1	path:04110_10	4.74E-10
E2F1	path:04110_9	4.74E-10

E2F1	path:04110_5	4.74E-10
SP1	path:05214_3	4.99E-10
SP1	path:05220_4	4.99E-10
RELA	path:04620_3	1.21E-09
STAT3	path:05200_37	1.34E-09
E2F1	path:04110_1	1.46E-09
RELA	path:04620_1	1.59E-09
TP53	path:04110_10	2.36E-09
TP53	path:04110_9	2.36E-09
TP53	path:04110_5	2.36E-09
SP3	path:05200_39	2.41E-09
SP3	path:04512_1	2.62E-09
SP3	path:04510_13	2.62E-09
SP3	path:04510_6	2.62E-09
SP3	path:04510_5	2.62E-09
SP3	path:04510_7	2.62E-09
SP1	path:04110_1	2.95E-09
SP1	path:05200_30	3.09E-09
SPI1	path:04670_4	3.21E-09
SPI1	path:04670_9	3.21E-09
SPI1	path:04670_8	3.21E-09
STAT3	path:05200_35	6.34E-09
SP1	path:05200_18	6.86E-09
TP53	path:04110_7	7.23E-09
TP53	path:04110_1	7.85E-09
HNF4A	path:04610_2	9.43E-09
FOS	path:05200_37	1.26E-08
PAX6	path:04510_6	1.43E-08
PAX6	path:04510_5	1.43E-08
PAX6	path:04510_7	1.43E-08
TCF7L2	path:04310_7	1.48E-08
SP3	path:04512_3	1.62E-08
SP3	path:04110_5	1.76E-08
NR5A1	path:00140_1	1.80E-08
JUND	path:05200_35	1.90E-08
SP1	path:04610_2	2.04E-08
HNF4A	path:03320_1	2.34E-08
TP53	path:04110_11	2.42E-08
FOS	path:05200_35	2.43E-08
FOS	path:05200_39	2.43E-08
TCF7L2	path:04310_5	3.41E-08
FOS	path:05200_25	3.44E-08
SP1	path:05219_3	3.46E-08
SP1	path:04510_4	3.46E-08

E2F1	path:05212_9	4.00E-08
NFYA	path:04110_2	4.19E-08
NFYA	path:04110_7	4.19E-08
ZBTB7A	path:04110_13	4.99E-08
E2F1	path:04110_2	5.15E-08
E2F1	path:05219_5	5.40E-08
ZBTB7A	path:04110_11	6.29E-08
SP3	path:05200_38	6.30E-08
TP53	path:05200_2	6.35E-08
E2F1	path:04110_7	6.37E-08
RELA	path:04062_1	6.41E-08
SP3	path:04110_11	6.86E-08
EGR1	path:04510_13	8.03E-08
SP1	path:00140_1	8.25E-08
SP1	path:04610_4	8.54E-08
SP3	path:04110_1	8.54E-08
SP1	path:04610_6	8.72E-08
E2F1	path:05200_37	9.41E-08
E2F1	path:05214_3	9.41E-08
E2F1	path:05220_4	9.41E-08
SP3	path:00140_1	9.88E-08
SP1	path:04510_12	1.03E-07
HNF4A	path:04610_1	1.04E-07
HNF4A	path:04610_3	1.04E-07
MYC	path:05200_24	1.07E-07
HNF4A	path:04610_5	1.07E-07
SP1	path:04610_1	1.15E-07
SP1	path:04610_3	1.15E-07
SP3	path:05200_37	1.26E-07
SPI1	path:04670_2	1.42E-07
STAT3	path:04630_2	1.47E-07
STAT3	path:05200_38	1.47E-07
RELA	path:04620_9	1.53E-07
RELA	path:04062_2	1.59E-07
RELA	path:04620_6	1.59E-07
RELA	path:04620_8	1.59E-07
SP3	path:04110_10	1.67E-07
SP3	path:04110_13	1.71E-07
SP3	path:04110_9	1.71E-07
RELA	path:04620_5	1.79E-07
E2F1	path:04110_13	2.09E-07
IRF1	path:04620_3	2.19E-07
SP3	path:05200_25	2.20E-07
SPI1	path:04670_6	2.26E-07

SPI1	path:04145_4	2.26E-07
IRF1	path:04620_1	2.40E-07
STAT3	path:05200_39	2.52E-07
SP3	path:04512_2	2.81E-07
IRF3	path:04620_7	2.93E-07
IRF3	path:04620_2	2.93E-07
IRF7	path:04620_7	2.93E-07
IRF7	path:04620_2	2.93E-07
TP53	path:05219_3	3.10E-07
SP1	path:05219_5	3.24E-07
TP53	path:05200_10	3.31E-07
TP53	path:05200_7	3.31E-07
JUND	path:05200_38	3.69E-07
PAX6	path:04510_4	4.20E-07
AKNA	path:04060_18	4.33E-07
AKNA	path:04514_8	4.33E-07
AKNA	path:04672_2	4.33E-07
AKNA	path:05144_4	4.33E-07
AKNA	path:05145_14	4.33E-07
AKNA	path:05310_1	4.33E-07
AKNA	path:05320_1	4.33E-07
AKNA	path:05322_3	4.33E-07
AKNA	path:05330_1	4.33E-07
AKNA	path:05416_3	4.33E-07
PAX6	path:04512_3	4.42E-07
E2F1	path:05223_3	4.42E-07
MITF	path:04916_8	4.58E-07
MITF	path:04916_3	4.58E-07
MITF	path:04916_6	4.58E-07
FOS	path:05200_38	4.60E-07
JUND	path:05200_39	5.05E-07
E2F1	path:05200_38	5.19E-07
SP1	path:05200_6	5.48E-07
JUND	path:05200_25	6.49E-07
E2F1	path:05222_1	6.50E-07
RFX3	path:04514_19	6.68E-07
RFX3	path:04612_3	6.68E-07
NFYA	path:04110_5	6.77E-07
NFE2L2	path:00480_1	7.00E-07
E2F1	path:05200_39	8.31E-07
TP53	path:05200_9	8.31E-07
SP1	path:05215_9	8.52E-07
SP3	path:04510_14	8.76E-07
SP3	path:04510_1	9.07E-07

TP53	path:05218_3	9.47E-07
HNF4A	path:00982_2	9.51E-07
TP53	path:05200_19	1.03E-06
TP53	path:05200_15	1.09E-06
TP53	path:05200_39	1.15E-06
STAT3	path:05200_24	1.17E-06
SP3	path:05214_3	1.25E-06
SP3	path:05220_4	1.25E-06
SP3	path:05200_15	1.36E-06
RBPJ	path:04620_3	1.51E-06
SP3	path:05200_35	1.52E-06
TP53	path:05214_3	1.52E-06
TP53	path:05220_4	1.52E-06
SP1	path:05218_3	1.57E-06
NFYA	path:04110_1	1.59E-06
TP53	path:05200_25	1.66E-06
RFX2	path:04514_19	1.67E-06
RFX2	path:04612_3	1.67E-06
SPI1	path:05140_3	1.97E-06
JUND	path:05200_37	2.04E-06
PAX6	path:04510_14	2.11E-06
PAX6	path:04510_1	2.11E-06
SP1	path:05218_5	2.17E-06
SP1	path:05222_1	2.17E-06
SPI1	path:05140_2	2.40E-06
TCF7L2	path:05216_3	2.45E-06
TFAP2A	path:05200_10	2.50E-06
E2F1	path:05218_5	2.80E-06
RELA	path:05200_37	2.84E-06
SP1	path:04610_5	2.91E-06
PAX6	path:05414_2	3.01E-06
CEBPA	path:04610_4	3.03E-06
SP1	path:04144_2	3.05E-06
YY1	path:04110_5	3.50E-06
SP1	path:04630_2	3.87E-06
HNF4A	path:04610_4	4.06E-06
IRF1	path:04620_7	4.18E-06
SP1	path:05200_9	4.33E-06
E2F1	path:05219_3	4.92E-06
SP1	path:05210_8	4.93E-06
SP1	path:04110_2	5.26E-06
TFAP2A	path:05200_19	5.35E-06
YY1	path:04110_1	5.46E-06
ESR1	path:05200_39	5.64E-06

ESR1	path:05200_25	5.64E-06
TP53	path:05200_37	6.13E-06
SP1	path:05200_33	6.48E-06
TFAP2A	path:05200_17	6.90E-06
SP1	path:04310_7	6.94E-06
SP1	path:05212_9	6.94E-06
TCF7L2	path:05213_1	7.20E-06
TCF7L2	path:05210_8	7.48E-06
SP1	path:05210_5	7.71E-06
SP1	path:05218_1	7.86E-06
TCF7L2	path:05210_5	7.93E-06
TFAP2A	path:05200_30	8.21E-06
TFAP2A	path:04510_12	8.21E-06
TFAP2A	path:05200_24	8.32E-06
TP53	path:05215_9	9.26E-06
RFX1	path:04514_19	9.33E-06
RFX1	path:04612_3	9.33E-06
SP3	path:05219_3	9.51E-06
SP1	path:04310_5	9.60E-06
SP1	path:05218_2	9.93E-06
SP1	path:04110_7	1.01E-05
RELA	path:05200_39	1.03E-05
NFYA	path:04110_11	1.08E-05
ZBTB7A	path:05200_26	1.08E-05
TP53	path:04110_6	1.13E-05
REL	path:04650_5	1.13E-05
CEBPB	path:04620_9	1.18E-05
CEBPB	path:04060_38	1.18E-05
CEBPB	path:04620_6	1.18E-05
CEBPB	path:04620_8	1.18E-05
CEBPB	path:04620_5	1.18E-05
AR	path:04115_2	1.25E-05
TCF7L2	path:05221_2	1.27E-05
TFAP2A	path:05200_7	1.38E-05
SP1	path:05200_14	1.45E-05
ESR1	path:05200_37	1.47E-05
TP53	path:05215_10	1.51E-05
LEF1	path:05216_3	1.52E-05
SP1	path:05414_2	1.55E-05
IRF3	path:04060_23	1.58E-05
IRF7	path:04060_23	1.58E-05
RELA	path:05200_25	1.61E-05
NFYA	path:04110_10	1.65E-05
NFYA	path:04110_9	1.65E-05

RELA	path:04620_7	1.66E-05
HNF4A	path:00830_1	1.68E-05
SMAD2	path:04512_3	1.70E-05
SMAD2	path:04512_1	1.70E-05
SP1	path:05223_3	1.76E-05
ZBTB7A	path:05200_38	1.83E-05
ZBTB7A	path:05200_18	1.83E-05
ZBTB7A	path:05200_15	1.83E-05
ZBTB7A	path:05200_35	1.83E-05
ZBTB7A	path:05200_39	1.83E-05
ZBTB7A	path:04110_10	1.83E-05
ZBTB7A	path:04110_9	1.83E-05
TCF7	path:04660_15	1.83E-05
ZBTB7A	path:05200_25	1.85E-05
TP53	path:05200_13	1.99E-05
NFYA	path:04115_2	2.03E-05
E2F1	path:05215_3	2.19E-05
NR5A2	path:00140_1	2.25E-05
MYC	path:04110_13	2.37E-05
MYC	path:05200_38	2.42E-05
MYC	path:04110_11	2.42E-05
MYC	path:05200_35	2.42E-05
MYC	path:05200_39	2.42E-05
MYC	path:05222_5	2.42E-05
MYC	path:04110_10	2.42E-05
MYC	path:04110_9	2.42E-05
SP1	path:05222_2	2.43E-05
LEF1	path:05213_1	2.46E-05
LEF1	path:05210_8	2.46E-05
LEF1	path:05210_5	2.46E-05
MYC	path:05200_25	2.50E-05
POU2F1	path:00830_1	2.52E-05
HNF4A	path:00980_1	2.54E-05
SP1	path:05142_3	2.58E-05
IRF5	path:04620_3	2.94E-05
IRF5	path:04620_1	2.94E-05
JUN	path:05200_37	3.15E-05
JUN	path:04620_9	3.15E-05
JUN	path:04620_6	3.15E-05
JUN	path:04620_8	3.15E-05
JUN	path:04620_5	3.15E-05
JUN	path:04650_3	3.15E-05
JUN	path:05200_35	3.15E-05
JUN	path:05200_39	3.15E-05

IRF3	path:04620_9	3.21E-05
IRF7	path:04620_9	3.21E-05
SP3	path:05219_5	3.24E-05
ZBTB7A	path:05200_37	3.34E-05
TFAP2A	path:05200_8	3.34E-05
IRF5	path:04620_4	3.43E-05
SP1	path:04610_7	3.53E-05
SP1	path:04510_16	3.54E-05
SP1	path:04350_1	3.54E-05
IRF3	path:04650_5	3.56E-05
IRF3	path:04650_3	3.56E-05
IRF7	path:04650_5	3.56E-05
IRF7	path:04650_3	3.56E-05
ATF2	path:05200_35	3.57E-05
ATF2	path:04620_9	3.57E-05
ATF2	path:04620_6	3.57E-05
ATF2	path:04620_8	3.57E-05
ATF2	path:04620_5	3.57E-05
SMAD2	path:04510_14	3.63E-05
SMAD2	path:04510_1	3.63E-05
SMAD2	path:04510_6	3.63E-05
SMAD2	path:04510_5	3.63E-05
SMAD2	path:04510_7	3.63E-05
TFAP2A	path:05200_38	3.67E-05
TFAP2A	path:05200_9	3.67E-05
LEF1	path:05221_2	3.67E-05
TFAP2A	path:05200_6	3.69E-05
SMAD4	path:05200_38	3.71E-05
SMAD4	path:05200_35	3.71E-05
SMAD4	path:05200_39	3.71E-05
ATF2	path:05200_37	3.80E-05
SMAD4	path:05200_25	3.97E-05
NFATC2	path:04060_4	4.21E-05
CEBPB	path:04620_3	4.26E-05
SREBF1	path:04910_4	4.39E-05
SP1	path:04210_9	4.41E-05
PAX6	path:04510_3	4.51E-05
YY1	path:04110_10	4.51E-05
YY1	path:04110_9	4.51E-05
SP1	path:04810_16	4.56E-05
TFAP2A	path:05200_35	4.58E-05
TFAP2A	path:05200_39	4.58E-05
RBPJ	path:04620_9	4.66E-05
RBPJ	path:04620_6	4.66E-05

**RBPJ**	**path:04620_5**	**4.66E-05**
**RBPJ**	**path:05215_4**	**4.66E-05**
**JUN**	**path:05200_25**	**4.74E-05**
**CEBPB**	**path:05200_37**	**4.78E-05**
**CEBPB**	**path:04620_1**	**4.78E-05**
**TP53**	**path:05218_5**	**4.87E-05**
**PAX6**	**path:04510_16**	**4.93E-05**
**PAX6**	**path:04810_16**	**4.93E-05**
**E2F1**	**path:05200_15**	**4.94E-05**
**MYC**	**path:05222_1**	**5.00E-05**
**E2F1**	**path:05200_35**	**5.08E-05**
**MYC**	**path:05200_37**	**5.15E-05**
**GATA4**	**path:00980_1**	**5.16E-05**
**SP1**	**path:05200_19**	**5.29E-05**
**FLI1**	**path:04512_1**	**5.32E-05**
**TCF7L2**	**path:05200_34**	**5.33E-05**
**TFAP2A**	**path:05200_2**	**5.56E-05**
**HNF1A**	**path:00140_1**	**5.73E-05**
**IRF3**	**path:05160_4**	**5.84E-05**
**IRF7**	**path:05160_4**	**5.84E-05**
**LEF1**	**path:04916_8**	**5.97E-05**
**YY1**	**path:05219_3**	**6.00E-05**
**YY1**	**path:04650_5**	**6.00E-05**
**TFAP2A**	**path:05200_25**	**6.16E-05**
**STAT3**	**path:05200_21**	**6.16E-05**
**SP1**	**path:04912_7**	**6.23E-05**
**TCF7L2**	**path:05200_21**	**6.50E-05**
**NR1I3**	**path:00830_1**	**6.80E-05**
**NR1I3**	**path:00980_1**	**6.80E-05**
**SP1**	**path:05215_7**	**6.88E-05**
**E2F1**	**path:05200_25**	**6.91E-05**
**SP1**	**path:05218_4**	**6.99E-05**
**ZBTB7A**	**path:05219_3**	**7.00E-05**
**TCF7L2**	**path:05200_26**	**7.09E-05**
**SP1**	**path:05200_10**	**7.15E-05**
**E2F1**	**path:04115_2**	**7.56E-05**
**LEF1**	**path:04916_3**	**7.73E-05**
**LEF1**	**path:04916_6**	**7.73E-05**
**PAX6**	**path:04512_1**	**7.80E-05**
**SMAD4**	**path:05200_37**	**7.85E-05**
**ATF2**	**path:04620_3**	**8.14E-05**
**ATF2**	**path:04110_13**	**8.73E-05**
**ATF2**	**path:04620_1**	**8.73E-05**
**SMAD2**	**path:04110_13**	**8.76E-05**

KLF4	path:04110_11	8.78E-05
KLF4	path:04110_10	8.78E-05
KLF4	path:04110_9	8.78E-05
KLF4	path:04110_5	8.78E-05
SMAD3	path:05200_38	8.80E-05
SMAD3	path:05200_35	8.80E-05
SMAD3	path:05200_39	8.80E-05
JUN	path:04620_3	8.92E-05
RELA	path:04670_2	8.94E-05
SP3	path:04510_12	8.95E-05
SP1	path:04350_4	8.96E-05
TP53	path:05219_5	9.08E-05
TP53	path:05200_21	9.08E-05
TP53	path:05218_1	9.08E-05
IRF1	path:04620_9	9.08E-05
HNF1A	path:00980_1	9.37E-05
HNF1A	path:04610_2	9.37E-05
SMAD3	path:05200_25	9.41E-05
RELA	path:04060_44	9.57E-05
TP53	path:05200_16	9.68E-05
TP53	path:05218_2	9.68E-05
LEF1	path:05200_34	9.78E-05
MITF	path:00350_1	9.86E-05
SP1	path:05200_16	0.000101
SP1	path:05142_11	0.000101
SP1	path:05222_5	0.000101
TP53	path:05200_26	0.000106
CEBPB	path:04620_4	0.000108
ZBTB7A	path:05219_5	0.000111
TP63	path:04110_10	0.000112
TP63	path:04110_9	0.000112
TP63	path:04110_5	0.000112
TP63	path:04115_2	0.000112
TP53	path:05200_24	0.000117
RBPJ	path:04620_1	0.000117
RELA	path:05200_38	0.00012
LEF1	path:05200_21	0.000121
JUN	path:04620_1	0.000121
FUS	path:00230_2	0.000121
RUNX3	path:04612_4	0.000121
SP3	path:05218_5	0.000125
TFAP2A	path:04510_13	0.000127
TXK	path:04612_5	0.00013
CEBPA	path:04610_1	0.000131

CEBPA	path:04610_3	0.000131
POU2F1	path:04110_11	0.000132
LEF1	path:05200_26	0.000133
STAT4	path:04630_2	0.000135
TP63	path:04110_1	0.000135
TFAP2A	path:04510_4	0.000135
PAX6	path:05100_6	0.00014
E2F1	path:05200_33	0.00014
POU2F1	path:04110_10	0.000141
POU2F1	path:04110_9	0.000141
FUS	path:00230_3	0.000143
ZBTB7A	path:05214_3	0.000149
ZBTB7A	path:05220_4	0.000149
KLF4	path:04110_1	0.000149
SP3	path:04510_4	0.000151
IRF2	path:04620_4	0.000151
ESR1	path:04115_2	0.000152
CEBPA	path:04610_2	0.000156
TFAP2A	path:05200_37	0.000157
SP1	path:05215_10	0.000161
SMAD3	path:05200_37	0.000164
SMAD3	path:04512_3	0.000164
SMAD3	path:04512_1	0.000164
ATF2	path:04620_4	0.000164
ATF2	path:05200_38	0.000164
PAX6	path:04514_5	0.000167
NFATC2	path:05142_11	0.000171
HIF1A	path:05200_37	0.000173
ETV4	path:05200_37	0.000176
STAT5A	path:04630_2	0.000194
STAT5B	path:04630_2	0.000194
RBPJ	path:05200_13	0.000198
ZBTB7A	path:05222_5	0.000199
RELA	path:05200_35	0.000203
YY1	path:05214_3	0.000204
YY1	path:05220_4	0.000204
YY1	path:04110_13	0.000204
E2F1	path:05223_4	0.000204
SP3	path:05200_13	0.00021
NFYA	path:04110_6	0.000211
GATA4	path:00591_1	0.000213
TCF7L2	path:05200_38	0.000217
RBPJ	path:05200_15	0.000219
TP53	path:04110_13	0.00022

AR	path:05200_35	0.000223
AR	path:04110_10	0.000223
AR	path:05200_25	0.000223
E2F1	path:05218_3	0.000224
TP73	path:04115_2	0.000225
TFAP2A	path:04144_2	0.000225
SP1	path:05200_20	0.000225
TCF7L2	path:05200_18	0.000226
TCF7L2	path:05200_15	0.000226
TCF7L2	path:05200_35	0.000226
TCF7L2	path:05200_39	0.000226
TP63	path:05219_3	0.000227
SMAD2	path:04110_10	0.00023
MYC	path:05200_21	0.000231
TP53	path:05215_6	0.000236
LEF1	path:04310_7	0.000237
PAX6	path:04514_17	0.000238
ATF2	path:05200_39	0.00024
SP1	path:05145_8	0.000243
SP1	path:05216_3	0.000243
SP1	path:04620_9	0.000245
PAX6	path:04510_13	0.000245
NR2F1	path:03320_1	0.00025
ATF2	path:04110_10	0.000256
SP1	path:04350_5	0.00026
ATF2	path:05142_3	0.00026
STAT1	path:04620_9	0.00026
FOSL1	path:05200_35	0.000264
RELA	path:04650_5	0.000265
STAT3	path:05210_8	0.000265
SMAD2	path:04512_2	0.000267
REL	path:04650_15	0.000267
HNF4A	path:04610_7	0.000271
ATF2	path:05200_25	0.000272
TCF7L2	path:05200_25	0.000276
SP3	path:05200_21	0.000277
SP3	path:05200_18	0.000277
JUN	path:04620_4	0.00028
HNF4A	path:00591_1	0.000287
JUN	path:05200_38	0.000287
NR5A1	path:04060_2	0.000292
TP53	path:04722_15	0.000295
SP1	path:04010_6	0.000298
REL	path:04650_14	0.000298

STAT3	path:05210_5	0.000303
STAT1	path:05200_26	0.000314
STAT1	path:05200_24	0.000314
TP53	path:05200_14	0.000326
RELA	path:04115_2	0.00033
TP63	path:05219_5	0.000334
POU2F1	path:05200_37	0.000343
POU2F1	path:05200_26	0.000343
STAT3	path:04060_1	0.000344
REL	path:04650_13	0.000347
SP3	path:05223_3	0.000351
SP3	path:05200_26	0.000352
TFAP2A	path:05215_9	0.000358
SMAD4	path:05200_30	0.000358
SMAD4	path:05200_26	0.000358
SMAD4	path:05200_24	0.000358
SMAD4	path:04510_13	0.000361
RELA	path:04110_2	0.000367
ZBTB7A	path:05222_1	0.000368
STAT3	path:05200_18	0.000368
STAT3	path:05200_15	0.000368
TP53	path:05200_8	0.000369
TP53	path:05214_2	0.000369
LEF1	path:05200_38	0.000377
ESR1	path:05200_24	0.000378
E2F1	path:04110_4	0.000379
LEF1	path:05200_18	0.000387
LEF1	path:05200_15	0.000387
LEF1	path:04310_5	0.000387
LEF1	path:05200_35	0.000387
LEF1	path:05200_39	0.000387
EGR1	path:04510_12	0.000387
EGR1	path:04510_6	0.000387
EGR1	path:04510_7	0.000387
EGR1	path:05200_30	0.000387
SPI1	path:04620_4	0.000394
GATA4	path:00982_2	0.000395
TP63	path:05214_3	0.000397
TP63	path:05220_4	0.000397
SP3	path:05200_24	0.000398
SP1	path:05014_2	0.000401
SP1	path:05140_4	0.000401
SP1	path:05146_1	0.000401
STAT3	path:05142_3	0.000403

E2F1	path:05200_21	0.000404
STAT1	path:04630_2	0.000411
CEBPB	path:04062_2	0.000413
ELF1	path:04060_1	0.000415
EGR1	path:05200_24	0.000415
FOXA2	path:04610_4	0.000416
FOXA2	path:04610_1	0.000416
FOXA2	path:04610_3	0.000416
FOXA2	path:04610_5	0.000416
FOXA2	path:04610_2	0.000416
SP3	path:05200_2	0.000419
AR	path:05200_37	0.00044
ATF2	path:04350_4	0.000443
SP1	path:00330_4	0.000448
SP1	path:05200_31	0.000448
SP1	path:05200_34	0.000448
NRL	path:04744_2	0.000455
YY1	path:04110_11	0.000455
SP1	path:00980_1	0.000468
SPI1	path:04060_26	0.000468
SMAD4	path:04110_13	0.000472
SP1	path:05215_6	0.000473
E2F1	path:05200_26	0.000474
LEF1	path:05200_25	0.000477
TP53	path:05200_6	0.000487
TP53	path:05214_1	0.000487
YBX1	path:05218_1	0.000487
YBX1	path:05218_2	0.000487
YBX1	path:04520_2	0.000487
EGR1	path:04350_5	0.000503
RELA	path:04110_7	0.000506
TCF7L2	path:05200_37	0.00051
TFAP2A	path:05214_2	0.000513
TFAP2A	path:05215_10	0.000513
SP3	path:04610_6	0.000519
SP1	path:00330_3	0.000536
SMAD3	path:05200_30	0.000539
SMAD3	path:05200_26	0.000539
SMAD3	path:05200_24	0.000539
TXK	path:04650_15	0.000546
TXK	path:05142_3	0.000546
TXK	path:04650_14	0.000546
TXK	path:04350_4	0.000546
TXK	path:04650_13	0.000548

SMAD3	path:04510_14	0.000554
SMAD3	path:04510_1	0.000554
SMAD3	path:04510_6	0.000554
SMAD3	path:04510_5	0.000554
SMAD3	path:04510_7	0.000554
SMAD3	path:04110_13	0.000554
RELA	path:04110_1	0.000556
YY1	path:05200_15	0.000557
YY1	path:05200_39	0.000557
SP3	path:04144_2	0.000562
SP3	path:04115_2	0.000569
JUN	path:04650_5	0.00058
STAT1	path:05210_8	0.000581
KLF5	path:05210_8	0.000591
KLF5	path:05210_5	0.000591
STAT1	path:05210_5	0.000599
STAT1	path:05200_38	0.000599
STAT1	path:05200_18	0.000608
STAT1	path:05200_15	0.000608
STAT1	path:05200_35	0.000608
STAT1	path:05200_39	0.000608
SP1	path:05140_6	0.000608
YBX1	path:04510_19	0.000611
YBX1	path:04520_5	0.000616
YBX1	path:05200_8	0.000616
YBX1	path:04520_3	0.000616
YBX1	path:04520_7	0.000616
YBX1	path:04520_6	0.000616
JUN	path:04210_9	0.000629
GATA4	path:00590_2	0.000641
GATA4	path:00590_1	0.000641
SP1	path:00330_5	0.000643
VDR	path:05200_16	0.000645
POU2F1	path:04110_13	0.000647
TXK	path:04650_5	0.000651
PAX6	path:05100_7	0.000652
SP3	path:04060_20	0.000657
KLF4	path:05200_21	0.000676
TFAP2A	path:05214_1	0.000681
ZBTB7A	path:05218_3	0.000683
TFAP2A	path:05215_8	0.000684
YBX1	path:05218_4	0.000697
ELF1	path:04664_6	0.000698
ZBTB7A	path:05218_5	0.000702

POU2F1	path:05222_5	0.000715
YY1	path:05200_25	0.00073
VDR	path:05200_10	0.000744
JUN	path:04115_2	0.000753
YY1	path:04115_2	0.000773
NR4A1	path:00140_1	0.000776
XBP1	path:04514_19	0.000779
XBP1	path:04612_3	0.000779
STAT1	path:05200_25	0.000791
FOS	path:04670_2	0.000797
YY1	path:04110_2	0.000819
SP1	path:04510_3	0.000821
TP53	path:05200_17	0.000849
RBPJ	path:05140_5	0.000876
RBPJ	path:05215_2	0.000876
STAT5B	path:04630_1	0.000879
STAT5B	path:05200_38	0.000879
STAT5B	path:05200_35	0.000879
STAT5B	path:05200_39	0.000879
LEF1	path:05200_37	0.000889
CEBPB	path:05200_20	0.000891
ZBTB7A	path:04110_4	0.000895
SP3	path:05200_33	0.000897
STAT5A	path:04630_1	0.000897
CREB1	path:05142_3	0.0009
ATF2	path:04650_5	0.00091
ZBTB7A	path:05200_21	0.000913
STAT5B	path:05200_25	0.000954
EGR1	path:05020_2	0.000955
SMAD4	path:05200_18	0.000956
SMAD4	path:05200_15	0.000956
SMAD4	path:05218_4	0.000956
CEBPB	path:04062_1	0.00096
ATF2	path:04650_3	0.000962
RBPJ	path:04210_3	0.000973
ELF1	path:04664_1	0.000987
RBPJ	path:05120_4	0.000987
RBPJ	path:05160_12	0.000987
SMAD4	path:04110_10	0.000989
SP3	path:05212_9	0.000994
VDR	path:05200_19	0.001009
STAT5B	path:05219_3	0.001012
TXK	path:04350_5	0.001014
TXK	path:04350_1	0.001014

POU2F1	path:05200_38	0.001025
POU2F1	path:00590_2	0.001025
POU2F1	path:05200_18	0.001025
POU2F1	path:00590_1	0.001025
POU2F1	path:05200_15	0.001025
POU2F1	path:05222_1	0.001025
POU2F1	path:05200_35	0.001025
POU2F1	path:05200_39	0.001025
STAT6	path:04630_2	0.001034
TFAP2A	path:05219_2	0.001046
ZBTB7A	path:05200_24	0.001054
RBPJ	path:05120_2	0.001071
RBPJ	path:04623_2	0.001071
RBPJ	path:04623_4	0.001071
RBPJ	path:04660_1	0.001071
RBPJ	path:04210_6	0.001071
RBPJ	path:05145_4	0.001071
RBPJ	path:04620_12	0.001071
RBPJ	path:05120_1	0.001071
RBPJ	path:05120_7	0.001071
RBPJ	path:05160_2	0.001071
RBPJ	path:05222_8	0.001071
RBPJ	path:04210_5	0.001071
RBPJ	path:04622_2	0.001071
RBPJ	path:05120_3	0.001071
RBPJ	path:04622_4	0.001071
RBPJ	path:04920_7	0.001071
RBPJ	path:05160_5	0.001071
RBPJ	path:05160_6	0.001071
GATA6	path:00591_1	0.001082
RBPJ	path:04622_5	0.001085
RBPJ	path:04920_6	0.001085
RBPJ	path:05220_6	0.001085
NFIC	path:05200_15	0.001098
NFIC	path:05200_39	0.001098
NFIC	path:05200_25	0.001098
HIF1A	path:05200_39	0.001098
HIF1A	path:05200_25	0.001098
YBX1	path:04510_12	0.0011
E2F1	path:04110_6	0.001101
SP1	path:04520_2	0.001102
SP1	path:05200_8	0.001102
CEBPA	path:04060_37	0.001105
ELF1	path:04664_2	0.001117

RBPJ	path:05200_33	0.001132
RBPJ	path:05222_7	0.001132
MYC	path:05200_15	0.001149
MYC	path:05219_3	0.001149
TP53	path:04510_13	0.00115
TP53	path:04210_8	0.00115
SPI1	path:04810_16	0.00116
JUN	path:04940_2	0.00116
JUN	path:05320_2	0.00116
JUN	path:05330_2	0.00116
JUN	path:05332_1	0.00116
IRF1	path:04060_1	0.001161
RBPJ	path:04062_11	0.001179
RBPJ	path:04621_3	0.001179
TP53	path:04510_6	0.001188
SMAD4	path:04115_2	0.00119
REL	path:04620_3	0.001207
TP53	path:04510_12	0.001207
MYC	path:05200_2	0.001209
MYC	path:04110_5	0.001209
MYC	path:05212_9	0.001209
MYC	path:05216_3	0.001209
SMAD3	path:05200_18	0.001224
SMAD3	path:05200_15	0.001224
RBPJ	path:04662_1	0.001227
RBPJ	path:05131_5	0.001227
SP1	path:04540_7	0.001233
SP1	path:04060_10	0.001233
SP1	path:05200_41	0.001233
SP1	path:05220_5	0.001233
TCF7L2	path:05222_5	0.001235
SP1	path:04010_7	0.001244
ZBTB7A	path:04350_1	0.001265
KLF4	path:04110_13	0.001265
POU2F1	path:04110_5	0.001276
REL	path:04620_1	0.001276
POU2F1	path:05200_25	0.001289
REL	path:04612_5	0.001297
HNF1A	path:04610_6	0.001299
HNF1A	path:04950_3	0.001299
GATA2	path:04060_32	0.0013
YBX1	path:04510_4	0.001311
SP3	path:05200_14	0.001311
STAT5B	path:05200_37	0.001348

STAT5B	path:05219_5	0.001348
RBPJ	path:04210_7	0.001352
RBPJ	path:04722_16	0.001352
RBPJ	path:05142_12	0.001352
YY1	path:05218_3	0.001358
YY1	path:05200_37	0.001358
SMAD3	path:04110_10	0.001365
MYC	path:04110_2	0.001367
POU2F1	path:05200_33	0.001379
RELA	path:04670_4	0.001386
HNF4A	path:00120_1	0.00139
ESR1	path:05200_38	0.001399
MYC	path:05219_5	0.001406
JUN	path:05142_11	0.001428
YY1	path:05218_5	0.001438
SP3	path:05414_2	0.001452
TFAP2A	path:04510_6	0.001477
TFAP2A	path:04510_5	0.001477
TFAP2A	path:04510_7	0.001477
RBPJ	path:04620_8	0.001478
RBPJ	path:04722_10	0.001478
RBPJ	path:05142_6	0.001478
CREB1	path:04620_9	0.00149
CREB1	path:04620_6	0.00149
CREB1	path:04620_8	0.00149
CREB1	path:04620_5	0.00149
ATF2	path:05200_26	0.001508
ESR1	path:05200_15	0.001539
ESR1	path:05200_35	0.001539
ESR1	path:04610_6	0.001539
KLF4	path:05200_14	0.001553
KLF4	path:05200_38	0.001553
KLF4	path:05200_9	0.001553
KLF4	path:05200_13	0.001553
KLF4	path:05219_3	0.001553
KLF4	path:05200_15	0.001553
KLF4	path:05200_35	0.001553
KLF4	path:05200_39	0.001553
MYC	path:04110_7	0.001558
E2F1	path:05200_13	0.001569
JUN	path:04512_3	0.00158
HNF4A	path:04950_2	0.0016
ATF2	path:04612_5	0.0016
FOXA3	path:00591_1	0.001605

FOXA3	path:00982_2	0.001605
MYC	path:04110_1	0.001623
TP53	path:04510_4	0.001663
REL	path:04620_4	0.001668
SMAD3	path:04512_2	0.001671
SP3	path:05218_3	0.001678
FOS	path:04610_6	0.001679
LEF1	path:05222_5	0.001682
STAT5B	path:05214_3	0.001684
STAT5B	path:05220_4	0.001684
KLF4	path:05200_2	0.001709
JUN	path:04512_1	0.00171
YY1	path:04650_3	0.001711
SP1	path:05212_8	0.001715
RELA	path:04670_9	0.001739
ETV4	path:05200_38	0.001742
ETV4	path:05200_35	0.001742
ETV4	path:05200_39	0.001742
EGR1	path:05200_35	0.001751
EGR1	path:05218_4	0.001751
TFAP2C	path:05219_2	0.001767
STAT1	path:05200_37	0.001776
KLF4	path:05200_25	0.001781
RBPJ	path:05142_5	0.001792
TFAP2A	path:04080_5	0.0018
MYC	path:05214_3	0.001802
MYC	path:05220_4	0.001802
E2F1	path:05200_18	0.001813
ETV4	path:05200_25	0.001814
SP3	path:04060_28	0.001816
SP3	path:05144_2	0.001816
RELA	path:04650_15	0.001816
RELA	path:05142_3	0.001816
RELA	path:04670_8	0.001832
IRF5	path:04060_23	0.001849
TP53	path:04210_9	0.001852
TP53	path:04650_3	0.001852
TP53	path:05215_4	0.001852
JUN	path:04310_7	0.001854
KLF4	path:04115_2	0.001857
KLF4	path:05219_5	0.001888
THRB	path:04080_2	0.001909
TP63	path:05218_3	0.001914
TP63	path:05218_5	0.001914

**STAT1**	**path:04620_6**	**0.001938**
**STAT1**	**path:04620_8**	**0.001938**
**SP3**	**path:05200_9**	**0.001963**
**TP73**	**path:04722_15**	**0.001969**
**FOXA3**	**path:00140_1**	**0.00199**
**RELA**	**path:04110_11**	**0.002006**
**RELA**	**path:05200_13**	**0.002006**
**KLF5**	**path:05200_21**	**0.002013**
**NFIC**	**path:05200_37**	**0.002016**
**STAT1**	**path:04620_5**	**0.002032**
**TP63**	**path:05215_4**	**0.00204**
**SP3**	**path:04610_7**	**0.002065**
**SP3**	**path:05220_8**	**0.002065**
**NFIC**	**path:05218_3**	**0.002077**
**MYC**	**path:05213_1**	**0.002078**
**RELA**	**path:04670_6**	**0.002097**
**SMAD2**	**path:04510_13**	**0.002105**
**E2F1**	**path:05014_2**	**0.00212**
**RBPJ**	**path:04620_2**	**0.002128**
**TP63**	**path:05200_1**	**0.002151**
**TP63**	**path:05200_21**	**0.002151**
**POU2F1**	**path:00980_1**	**0.002152**
**POU2F1**	**path:04110_1**	**0.002152**
**FOXA3**	**path:00830_1**	**0.002153**
**FOXA3**	**path:00980_1**	**0.002153**
**SPI1**	**path:04145_1**	**0.002156**
**SPI1**	**path:05150_4**	**0.002156**
**FOS**	**path:04115_2**	**0.00217**
**SPI1**	**path:05200_34**	**0.002179**
**TP53**	**path:05200_1**	**0.002197**
**TP53**	**path:05215_7**	**0.002197**
**NFIC**	**path:00591_1**	**0.002219**
**NFIC**	**path:04080_5**	**0.002219**
**NFIC**	**path:05200_21**	**0.002219**
**NFIC**	**path:05218_1**	**0.002219**
**NFIC**	**path:05218_2**	**0.002219**
**NFIC**	**path:05200_26**	**0.002219**
**YY1**	**path:05200_21**	**0.00222**
**NFIC**	**path:05200_24**	**0.002228**
**SP1**	**path:00590_2**	**0.002236**
**E2F1**	**path:05200_2**	**0.002241**
**EGR1**	**path:05200_25**	**0.002252**
**VDR**	**path:05200_7**	**0.002254**
**CREM**	**path:05200_38**	**0.002267**

CREM	path:05200_35	0.002267
CREM	path:05200_39	0.002267
CREM	path:05200_25	0.002267
TCF7L2	path:05222_1	0.002291
SP1	path:04060_19	0.002309
SP1	path:04510_10	0.002309
SP1	path:04722_15	0.002309
SP1	path:05020_2	0.002309
SP1	path:05200_42	0.002309
SP1	path:05142_12	0.002321
RBPJ	path:04722_9	0.002348
EGR1	path:04512_2	0.002352
MYC	path:05210_8	0.002364
STAT3	path:05221_1	0.002368
STAT3	path:05200_16	0.002368
STAT3	path:05200_30	0.002368
HNF4A	path:04610_6	0.002385
HNF4A	path:00140_1	0.002385
HNF4A	path:00590_2	0.002385
HNF4A	path:00010_1	0.002385
HNF4A	path:04950_3	0.002385
HNF4A	path:00590_1	0.002385
AR	path:05200_12	0.002388
AR	path:04110_13	0.002388
AR	path:05215_9	0.002424
RBPJ	path:05200_20	0.002436
GATA3	path:04612_4	0.002447
STAT3	path:05200_26	0.002456
ELF1	path:04650_10	0.002457
ELF1	path:04650_9	0.002457
ELF1	path:04650_2	0.002457
ELF1	path:04650_8	0.002457
RELA	path:04650_14	0.002465
RELA	path:05200_15	0.002465
HNF1A	path:04610_4	0.002466
HNF1A	path:04610_3	0.002466
SP1	path:04510_19	0.002496
SP1	path:05200_29	0.002496
EGR1	path:04510_5	0.002517
RBPJ	path:04920_1	0.002524
AR	path:05200_14	0.00253
AR	path:05200_38	0.00253
AR	path:05219_3	0.00253
AR	path:05200_9	0.00253

AR	path:05200_13	0.00253
YY1	path:05200_26	0.002558
JUN	path:04670_2	0.002581
ELF1	path:04650_4	0.002588
ESR1	path:00830_1	0.0026
KLF4	path:05214_3	0.002607
KLF4	path:05220_4	0.002607
STAT1	path:05200_21	0.002612
MYC	path:05210_5	0.002658
KLF4	path:05200_37	0.002689
IRF5	path:04650_5	0.002693
IRF5	path:04650_3	0.002693
IRF5	path:04620_7	0.002693
IRF5	path:04620_2	0.002693
IRF5	path:05160_4	0.002693
GATA6	path:00590_2	0.002699
GATA6	path:00590_1	0.002699
SP3	path:04610_2	0.002702
JUN	path:05120_7	0.002705
TP63	path:05200_20	0.002718
SMAD4	path:04012_7	0.002721
AR	path:05200_15	0.002722
AR	path:05216_3	0.002722
AR	path:05200_39	0.002722
AR	path:04110_9	0.002722
AR	path:05200_2	0.002722
AR	path:04110_5	0.002722
AR	path:05219_5	0.002722
POU2F1	path:00591_1	0.002768
POU2F1	path:04664_1	0.002768
TP53	path:04912_7	0.002771
GLI3	path:05217_1	0.002794
GLI3	path:05217_2	0.002794
GLI3	path:05200_27	0.002794
GLI3	path:05200_28	0.002794
NR5A2	path:03320_1	0.002861
ZBTB7A	path:04110_5	0.00295
IRF2	path:04620_3	0.002952
IRF2	path:04620_1	0.002952
RELA	path:04110_10	0.002957
TCF7L2	path:05215_5	0.002985
RELA	path:04110_9	0.002985
RELA	path:05200_2	0.002985
JUN	path:04610_6	0.003004

JUN	path:05120_3	0.003004
SMAD4	path:05210_8	0.003025
TP73	path:05200_33	0.003027
SP3	path:04110_4	0.003036
ATF2	path:05020_4	0.003047
ATF2	path:05219_3	0.003047
NFYA	path:04110_4	0.003087
TP63	path:05215_6	0.003093
TP63	path:05200_3	0.003093
TP63	path:05215_9	0.003093
RELA	path:04110_5	0.003103
SMAD3	path:04012_7	0.003126
LEF1	path:05222_1	0.003143
TFAP2A	path:05215_7	0.003177
NR1I2	path:00982_2	0.00321
SP1	path:04630_1	0.003223
SP1	path:04620_6	0.003223
SP1	path:04620_8	0.003223
SP1	path:05220_8	0.003223
PAX6	path:04670_4	0.003244
RELA	path:04650_13	0.003264
IRF1	path:04620_13	0.003327
JUNB	path:04350_2	0.003328
JUNB	path:04350_3	0.003328
SMAD4	path:05210_5	0.003331
PAX6	path:04670_2	0.003345
PAX6	path:04670_9	0.003345
ESR1	path:00980_1	0.003353
TP63	path:05200_14	0.003368
KLF4	path:05210_8	0.003371
TP53	path:05223_2	0.003372
MYC	path:05223_3	0.00338
SP3	path:05218_1	0.003395
TP63	path:05215_10	0.003441
TP63	path:04110_11	0.003441
TP63	path:05200_9	0.003441
TP63	path:05200_13	0.003441
FOS	path:04620_9	0.003457
FOS	path:04610_7	0.003457
FOS	path:04620_6	0.003457
FOS	path:04620_8	0.003457
SMAD3	path:05210_8	0.003524
FLI1	path:04512_3	0.003526
CREM	path:05200_37	0.003557

FOS	path:04620_5	0.003567
BCL6	path:04210_3	0.00357
NR1I2	path:00830_1	0.003589
NR1I2	path:00980_1	0.003589
TP53	path:05200_20	0.003604
SP1	path:04060_20	0.003616
KLF4	path:05145_11	0.003616
KLF4	path:05210_5	0.003616
POU2F1	path:05200_21	0.003618
TFAP2A	path:05200_16	0.003624
TFAP2A	path:05223_1	0.003624
SP1	path:04210_6	0.003664
SP1	path:00350_2	0.003664
SP1	path:04620_5	0.003664
SP1	path:04650_3	0.003664
SP1	path:05215_4	0.003664
RBPJ	path:05200_14	0.003673
SP3	path:05200_16	0.003683
SP3	path:05218_2	0.003683
SP3	path:00120_2	0.003683
TP63	path:05200_19	0.003688
TP63	path:05200_15	0.003688
TP63	path:05200_39	0.003688
MYC	path:05221_2	0.003701
TFAP2A	path:04520_2	0.003715
TFAP2A	path:05200_26	0.003715
TFAP2A	path:04912_7	0.003715
ATF2	path:04010_11	0.00372
ATF2	path:05212_9	0.00372
SP1	path:05200_17	0.003728
TFAP2A	path:04010_7	0.003765
TFAP2A	path:05212_3	0.003765
AR	path:05214_3	0.003775
AR	path:05220_4	0.003775
CEBPB	path:05200_35	0.003841
CEBPB	path:05200_39	0.003841
YBX1	path:05213_2	0.003844
YBX1	path:05100_10	0.003844
YBX1	path:05100_11	0.003844
YBX1	path:05211_4	0.003844
BCL6	path:04210_6	0.003892
SMAD3	path:05210_5	0.003931
RBPJ	path:05215_10	0.003933
TP63	path:05200_2	0.003951

SRF	path:05416_1	0.003973
GLI3	path:04340_1	0.003996
RBPJ	path:04620_4	0.004027
ATF2	path:04110_11	0.004031
ZBTB7B	path:04512_2	0.004036
ZBTB7B	path:04512_3	0.004036
ZBTB7B	path:04512_1	0.004036
ZBTB7B	path:04510_14	0.004036
ZBTB7B	path:04510_1	0.004036
ZBTB7B	path:04510_13	0.004036
ZBTB7B	path:04510_6	0.004036
ZBTB7B	path:04510_5	0.004036
ZBTB7B	path:04510_7	0.004036
ESR1	path:04610_7	0.004068
TP63	path:05200_25	0.004079
AR	path:04110_1	0.004098
GATA1	path:04060_32	0.004098
ZBTB7A	path:04110_1	0.004114
LEF1	path:05215_5	0.004122
AR	path:05215_1	0.00415
EGR1	path:05200_37	0.004181
EGR1	path:04512_3	0.004181
EGR1	path:05200_31	0.004181
RORA	path:05200_36	0.004284
ATF2	path:05219_5	0.004286
ATF2	path:00140_1	0.004286
ATF2	path:05200_18	0.004286
CEBPB	path:05200_33	0.004299
EGR1	path:04512_1	0.004316
E2F1	path:05222_5	0.004322
IRF1	path:04620_10	0.004324
SP1	path:04060_32	0.004327
SP1	path:04940_2	0.004327
SP1	path:05320_2	0.004327
SP1	path:05330_2	0.004327
SP1	path:05332_1	0.004327
TFAP2A	path:05212_8	0.004356
SP1	path:05020_1	0.004376
SP1	path:05221_2	0.004376
ATF2	path:05200_15	0.004376
IRF1	path:04620_6	0.004384
IRF1	path:04620_8	0.004384
YBX1	path:05211_5	0.004386
YBX1	path:04510_13	0.004386

**YBX1**	**path:04510_6**	**0.004386**
**YBX1**	**path:04510_5**	**0.004386**
**YBX1**	**path:04510_7**	**0.004386**
**FOXO3**	**path:04920_2**	**0.004407**
**TFAP2C**	**path:04912_7**	**0.004411**
**IRF1**	**path:04620_5**	**0.004456**
**ELF1**	**path:04060_34**	**0.004481**
**TP63**	**path:04110_2**	**0.004483**
**STAT3**	**path:04630_1**	**0.004494**
**TP53**	**path:04510_19**	**0.0045**
**SP3**	**path:00120_1**	**0.004574**
**EGR1**	**path:05215_7**	**0.004587**
**KLF5**	**path:05200_35**	**0.004593**
**KLF5**	**path:05200_25**	**0.004593**
**POU2F1**	**path:05200_24**	**0.004597**
**AR**	**path:05200_7**	**0.004618**
**SRF**	**path:05020_3**	**0.004628**
**SRF**	**path:05020_4**	**0.004628**
**RELA**	**path:04612_5**	**0.004631**
**NFIC**	**path:05219_3**	**0.004636**
**STAT6**	**path:00590_2**	**0.004645**
**STAT6**	**path:00590_1**	**0.004645**
**ESR1**	**path:04610_2**	**0.004673**
**SP1**	**path:04520_5**	**0.004701**
**SP1**	**path:04110_4**	**0.004701**
**SP1**	**path:05200_1**	**0.004701**
**TP53**	**path:04360_3**	**0.004707**
**TP53**	**path:05160_10**	**0.004707**
**CEBPB**	**path:05200_25**	**0.004738**
**SPI1**	**path:04620_3**	**0.004758**
**SP1**	**path:04060_46**	**0.004878**
**SP1**	**path:05100_6**	**0.004878**
**SP1**	**path:05145_6**	**0.004878**
**SP1**	**path:05212_10**	**0.004878**
**NFIC**	**path:04510_6**	**0.004886**
**E2F1**	**path:04510_10**	**0.004902**
**JUN**	**path:04310_5**	**0.004909**
**STAT1**	**path:04110_13**	**0.004937**
**STAT1**	**path:04620_1**	**0.004937**
**STAT1**	**path:05219_3**	**0.004937**
**STAT1**	**path:04630_1**	**0.004937**
**YBX1**	**path:05211_1**	**0.004962**
**YBX1**	**path:05212_4**	**0.004962**
**CEBPB**	**path:04010_18**	**0.005023**

TP63	path:04110_7	0.00504
RBPJ	path:05200_35	0.005103
RBPJ	path:05200_39	0.005103
SP1	path:04210_5	0.005211
ATF2	path:04110_9	0.005236
POU2F1	path:05200_20	0.005253
TFAP2A	path:04510_19	0.005264
TFAP2A	path:05200_29	0.005264
SP3	path:04110_2	0.005272
STAT1	path:04670_4	0.005275
STAT1	path:04145_4	0.005275
STAT1	path:05145_8	0.005275
STAT1	path:05216_3	0.005275
STAT1	path:04670_2	0.005275
EGR1	path:05200_16	0.005288
ATF2	path:04110_5	0.005296
ATF2	path:05214_3	0.005296
ATF2	path:05220_4	0.005296
FOXA2	path:00980_1	0.005302
ESR1	path:05200_21	0.005318
TP53	path:05200_3	0.00533
POU2F1	path:00232_1	0.005358
POU2F1	path:04060_4	0.005358
POU2F1	path:05219_3	0.005358
POU2F1	path:04350_1	0.005358
STAT1	path:04670_9	0.005426
NFATC1	path:04060_19	0.005447
NFATC1	path:04060_20	0.005447
ESR1	path:05200_30	0.005467
SP3	path:05200_20	0.00551
RBPJ	path:05200_2	0.005577
POU2F1	path:00982_2	0.005606
RELA	path:04650_3	0.005642
SP1	path:04010_8	0.005644
SP1	path:05214_2	0.005644
SPI1	path:04620_1	0.005646
NFIC	path:05200_13	0.005688
NFIC	path:05200_6	0.005688
NFIC	path:00140_1	0.005688
NFIC	path:05200_18	0.005688
TP63	path:05200_37	0.005735
TP63	path:05200_7	0.005735
STAT1	path:04620_4	0.005738
STAT1	path:04670_8	0.005738

**HNF4A**	**path:00561_2**	**0.005773**
**STAT1**	**path:05219_5**	**0.005807**
**PAX6**	**path:04512_2**	**0.005809**
**CEBPA**	**path:04610_5**	**0.005819**
**TP53**	**path:05014_2**	**0.005838**
**RBPJ**	**path:05200_25**	**0.005865**
**STAT5B**	**path:05200_33**	**0.005876**
**CREM**	**path:05210_8**	**0.005885**
**CREM**	**path:05210_5**	**0.005885**
**SP3**	**path:04810_16**	**0.005886**
**SMAD4**	**path:05211_5**	**0.005916**
**TP53**	**path:04012_5**	**0.005921**
**NFATC2**	**path:04060_19**	**0.005962**
**FOS**	**path:05200_24**	**0.005988**
**KLF5**	**path:05200_37**	**0.005998**
**EGR1**	**path:05212_3**	**0.006036**
**SP1**	**path:05223_1**	**0.006038**
**ETS1**	**path:04610_4**	**0.006052**
**ETS1**	**path:04620_9**	**0.006052**
**ETS1**	**path:04610_1**	**0.006052**
**ETS1**	**path:04610_3**	**0.006052**
**ETS1**	**path:04620_6**	**0.006052**
**ETS1**	**path:04620_5**	**0.006052**
**ETS1**	**path:05215_4**	**0.006052**
**ETS1**	**path:04610_2**	**0.006052**
**ETS1**	**path:04612_4**	**0.006052**
**ETS1**	**path:05212_6**	**0.006052**
**STAT1**	**path:04110_11**	**0.006064**
**ETS1**	**path:05212_1**	**0.006081**
**CEBPB**	**path:04610_4**	**0.006129**
**YY1**	**path:05212_9**	**0.006167**
**ATF2**	**path:04110_2**	**0.006193**
**TP53**	**path:04012_1**	**0.006227**
**STAT1**	**path:04670_6**	**0.006232**
**NFATC2**	**path:04060_20**	**0.006256**
**PAX6**	**path:04510_12**	**0.006286**
**SP1**	**path:04510_17**	**0.006301**
**ELK1**	**path:05200_26**	**0.006362**
**ELK1**	**path:04010_11**	**0.006362**
**ELK1**	**path:05020_3**	**0.006362**
**ELK1**	**path:05020_4**	**0.006362**
**ELK1**	**path:05110_3**	**0.006362**
**NFIC**	**path:04660_9**	**0.006384**
**NFIC**	**path:04662_9**	**0.006384**

NFIC	path:05200_2	0.006384
NR1I3	path:00982_2	0.006406
ELK1	path:05200_18	0.006535
NFIC	path:05214_3	0.006542
NFIC	path:05220_4	0.006542
NFIC	path:04115_2	0.006542
NFIC	path:04512_2	0.006542
RELA	path:04060_35	0.006581
SPI1	path:04650_1	0.006607
POU2F1	path:05216_3	0.006638
NFATC2	path:04060_46	0.006669
ATF2	path:05142_11	0.006684
ATF2	path:05210_8	0.006684
ATF2	path:05222_5	0.006684
ETS1	path:05212_2	0.006742
IRF1	path:04620_2	0.006742
NR2F2	path:00140_1	0.00675
NR2F2	path:03320_1	0.00675
MYC	path:04510_13	0.006799
NR5A1	path:04350_6	0.006802
ETS1	path:05212_3	0.006813
IRF1	path:05160_4	0.006813
TP53	path:05160_9	0.006828
TP53	path:05213_3	0.006828
EGR1	path:05212_8	0.006832
IRF1	path:04060_35	0.006848
IRF3	path:04620_6	0.006879
IRF3	path:04620_8	0.006879
IRF3	path:04620_5	0.006879
IRF7	path:04620_6	0.006879
IRF7	path:04620_8	0.006879
IRF7	path:04620_5	0.006879
SMAD4	path:04350_4	0.006895
ATF2	path:04110_7	0.006925
CREB1	path:04514_19	0.006954
CREB1	path:04612_3	0.006954
IRF2	path:05145_8	0.00698
ATF2	path:04010_10	0.007029
ATF2	path:04110_1	0.007029
ATF2	path:05210_5	0.007029
ELF1	path:00920_1	0.007049
EGR1	path:04144_2	0.007083
SMAD4	path:04510_6	0.007089
SMAD4	path:04510_5	0.007089

**SMAD4**	**path:04510_7**	**0.007089**
**YBX1**	**path:04910_8**	**0.007091**
**SP3**	**path:04110_7**	**0.007122**
**JUN**	**path:04512_2**	**0.007183**
**JUN**	**path:04612_5**	**0.007183**
**JUN**	**path:05200_41**	**0.007183**
**TP53**	**path:04010_11**	**0.007192**
**SMAD2**	**path:05200_21**	**0.007234**
**SMAD2**	**path:05200_30**	**0.007234**
**SMAD2**	**path:05200_26**	**0.007234**
**SMAD2**	**path:05200_24**	**0.007234**
**RELA**	**path:05200_21**	**0.007297**
**ESR1**	**path:05212_8**	**0.007324**
**EGR1**	**path:04350_1**	**0.007341**
**TFAP2A**	**path:04012_5**	**0.007365**
**YY1**	**path:05219_5**	**0.007374**
**YY1**	**path:05200_38**	**0.007374**
**HNF1A**	**path:00982_2**	**0.007452**
**YY1**	**path:05200_13**	**0.007519**
**ETS1**	**path:05212_8**	**0.007522**
**YBX1**	**path:05212_6**	**0.007526**
**YBX1**	**path:05215_7**	**0.007526**
**BCL6**	**path:05220_8**	**0.007528**
**BCL6**	**path:05200_21**	**0.007528**
**TP53**	**path:05200_38**	**0.007528**
**TCF7L2**	**path:05200_24**	**0.007564**
**FOSL1**	**path:05200_38**	**0.007569**
**FOSL1**	**path:05200_39**	**0.007569**
**FOSL1**	**path:05200_25**	**0.007569**
**EGR1**	**path:05200_29**	**0.007606**
**BCL6**	**path:04110_13**	**0.007614**
**BCL6**	**path:04630_1**	**0.007614**
**BCL6**	**path:04110_11**	**0.007614**
**BCL6**	**path:05200_13**	**0.007614**
**BCL6**	**path:05200_15**	**0.007614**
**BCL6**	**path:05200_39**	**0.007614**
**BCL6**	**path:04110_10**	**0.007614**
**BCL6**	**path:04110_9**	**0.007614**
**BCL6**	**path:05200_2**	**0.007614**
**BCL6**	**path:04110_5**	**0.007614**
**BCL6**	**path:05200_25**	**0.007614**
**MYC**	**path:05218_3**	**0.00762**
**BCL6**	**path:04115_2**	**0.007624**
**ETS1**	**path:04620_3**	**0.007626**

MYOD1	path:05200_24	0.007641
MYOD1	path:05200_38	0.007641
MYOD1	path:05200_18	0.007641
MYOD1	path:05200_15	0.007641
MYOD1	path:05200_39	0.007641
MYOD1	path:05200_25	0.007641
BCL6	path:04110_2	0.007648
SP1	path:04210_4	0.007677
TFAP2A	path:04520_5	0.007687
YBX1	path:05212_1	0.007688
YBX1	path:05200_16	0.007688
YBX1	path:05223_1	0.007688
STAT5B	path:05218_3	0.007738
NFATC1	path:04060_34	0.007771
STAT5B	path:05218_5	0.007781
STAT1	path:05214_3	0.007806
STAT1	path:05220_4	0.007806
SP1	path:00330_1	0.007811
SP1	path:05212_3	0.007811
YBX1	path:05212_2	0.007857
YBX1	path:05200_24	0.007857
YBX1	path:05212_3	0.007857
YBX1	path:04910_13	0.007857
YBX1	path:05223_2	0.007857
AR	path:05221_2	0.007863
SP3	path:00600_1	0.007906
STAT1	path:04110_10	0.007932
KLF4	path:05200_33	0.00799
E2F1	path:04210_5	0.007997
MYC	path:05218_5	0.008
JUND	path:05200_24	0.00807
YBX1	path:05212_8	0.008078
STAT1	path:04110_9	0.008119
SMAD4	path:04350_3	0.008193
CEBPB	path:04210_9	0.008204
NFIC	path:04660_10	0.008238
NFIC	path:04662_5	0.008238
POU2F1	path:05219_5	0.008262
CREB1	path:04620_4	0.008263
GLI1	path:04510_10	0.008264
ESR1	path:05219_3	0.008286
ESR1	path:00982_2	0.008286
YBX1	path:04144_2	0.008299
NFIC	path:04010_22	0.008376

SP1	path:05214_1	0.008395
BCL6	path:04110_7	0.0084
BCL6	path:04110_1	0.008428
TFAP2A	path:04020_1	0.008474
STAT1	path:05213_1	0.008595
YY1	path:05200_18	0.008601
FOS	path:05219_3	0.00861
SMAD3	path:04350_4	0.008655
YBX1	path:04910_2	0.008741
YBX1	path:05200_29	0.008741
RELA	path:05219_3	0.00875
SPI1	path:04650_7	0.008788
SPI1	path:05146_1	0.008788
ATF2	path:04010_6	0.008818
ATF2	path:05223_3	0.008818
JUN	path:04610_7	0.008856
NFATC2	path:04060_34	0.00892
BCL6	path:05200_37	0.008936
NFIC	path:04722_15	0.008938
IRF2	path:04060_34	0.008963
SP1	path:05014_1	0.009002
SP1	path:05215_5	0.009002
RBPJ	path:05200_37	0.009007
MYC	path:05200_34	0.009021
ETS2	path:04310_7	0.009024
ETS2	path:05200_9	0.009024
ETS2	path:05200_19	0.009024
ETS2	path:04310_5	0.009024
ETS2	path:05200_2	0.009024
ETS2	path:05200_17	0.009024
SP3	path:05222_1	0.009031
TCF7L2	path:04350_1	0.009037
ETS1	path:04620_1	0.009042
SRF	path:05410_1	0.009074
STAT3	path:04012_4	0.009094
STAT3	path:04060_34	0.009094
EGR1	path:05215_9	0.009118
SP1	path:04660_9	0.009179
SP1	path:04662_9	0.009179
SP1	path:05200_32	0.009179
FOS	path:04310_7	0.00922
YY1	path:05200_35	0.009247
SREBF1	path:04910_1	0.009269
TP53	path:05200_18	0.009346

**STAT3**	**path:05218_4**	**0.009361**
**ELK1**	**path:04010_4**	**0.009417**
**TFAP2A**	**path:04520_3**	**0.009447**
**POU2F1**	**path:04620_4**	**0.009505**
**GLI1**	**path:04510_8**	**0.009525**
**TCF7L2**	**path:04110_13**	**0.009585**
**STAT1**	**path:05145_12**	**0.009623**
**STAT1**	**path:05222_5**	**0.009623**
**YBX1**	**path:05200_10**	**0.009637**
**YBX1**	**path:05215_9**	**0.009637**
**TCF7L2**	**path:04630_1**	**0.009653**
**STAT5B**	**path:05200_21**	**0.009725**
**STAT5B**	**path:05218_1**	**0.009725**
**ATF2**	**path:04650_15**	**0.009755**
**STAT5B**	**path:05218_2**	**0.00976**
**TFAP2A**	**path:04520_7**	**0.009806**
**STAT5B**	**path:05200_26**	**0.009809**
**ETS1**	**path:05215_9**	**0.009853**
**STAT5B**	**path:05200_24**	**0.00987**
**ETS1**	**path:04060_46**	**0.009911**
**NFIC**	**path:05200_7**	**0.00997**

Table S7. The detailed information of the co-motif TFs regulated sub-pathways of TSN with k=4.

TF	Subpathway
HNF4A	path:00010_1
HNF4A	path:00120_1
SP3	path:00120_1
SP3	path:00120_2
ATF2	path:00140_1
FOXA3	path:00140_1
HNF1A	path:00140_1
HNF4A	path:00140_1
NFIC	path:00140_1
NR2F2	path:00140_1
NR4A1	path:00140_1
NR5A1	path:00140_1
NR5A2	path:00140_1
SP1	path:00140_1
SP3	path:00140_1
POU2F1	path:00232_1
SP1	path:00330_1
SP1	path:00330_3
SP1	path:00330_4
SP1	path:00330_5
MITF	path:00350_1
SP1	path:00350_2
NFE2L2	path:00480_1
HNF4A	path:00561_2
GATA4	path:00590_1
GATA6	path:00590_1
HNF4A	path:00590_1
POU2F1	path:00590_1
STAT6	path:00590_1
GATA4	path:00590_2
GATA6	path:00590_2
HNF4A	path:00590_2
POU2F1	path:00590_2
SP1	path:00590_2
STAT6	path:00590_2
FOXA3	path:00591_1
GATA4	path:00591_1
GATA6	path:00591_1
HNF4A	path:00591_1
NFIC	path:00591_1
POU2F1	path:00591_1
SP3	path:00600_1

**ESR1**	**path:00830_1**
**FOXA3**	**path:00830_1**
**HNF4A**	**path:00830_1**
**NR1I2**	**path:00830_1**
**NR1I3**	**path:00830_1**
**POU2F1**	**path:00830_1**
**ELF1**	**path:00920_1**
**ESR1**	**path:00980_1**
**FOXA2**	**path:00980_1**
**FOXA3**	**path:00980_1**
**GATA4**	**path:00980_1**
**HNF1A**	**path:00980_1**
**HNF4A**	**path:00980_1**
**NR1I2**	**path:00980_1**
**NR1I3**	**path:00980_1**
**POU2F1**	**path:00980_1**
**SP1**	**path:00980_1**
**ESR1**	**path:00982_2**
**FOXA3**	**path:00982_2**
**GATA4**	**path:00982_2**
**HNF1A**	**path:00982_2**
**HNF4A**	**path:00982_2**
**NR1I2**	**path:00982_2**
**NR1I3**	**path:00982_2**
**POU2F1**	**path:00982_2**
**HNF4A**	**path:03320_1**
**NR2F1**	**path:03320_1**
**NR2F2**	**path:03320_1**
**NR5A2**	**path:03320_1**
**ATF2**	**path:04010_10**
**ATF2**	**path:04010_11**
**ELK1**	**path:04010_11**
**TP53**	**path:04010_11**
**CEBPB**	**path:04010_18**
**NFIC**	**path:04010_22**
**ELK1**	**path:04010_4**
**ATF2**	**path:04010_6**
**SP1**	**path:04010_6**
**SP1**	**path:04010_7**
**TFAP2A**	**path:04010_7**
**SP1**	**path:04010_8**
**TP53**	**path:04012_1**
**STAT3**	**path:04012_4**
**TFAP2A**	**path:04012_5**

TP53	path:04012_5
SMAD3	path:04012_7
SMAD4	path:04012_7
TFAP2A	path:04020_1
ELF1	path:04060_1
IRF1	path:04060_1
STAT3	path:04060_1
SP1	path:04060_10
NFATC1	path:04060_19
NFATC2	path:04060_19
SP1	path:04060_19
NR5A1	path:04060_2
NFATC1	path:04060_20
NFATC2	path:04060_20
SP1	path:04060_20
SP3	path:04060_20
IRF3	path:04060_23
IRF5	path:04060_23
IRF7	path:04060_23
SPI1	path:04060_26
SP3	path:04060_28
GATA1	path:04060_32
GATA2	path:04060_32
SP1	path:04060_32
ELF1	path:04060_34
IRF2	path:04060_34
NFATC1	path:04060_34
NFATC2	path:04060_34
STAT3	path:04060_34
IRF1	path:04060_35
RELA	path:04060_35
CEBPA	path:04060_37
CEBPB	path:04060_38
NFATC2	path:04060_4
POU2F1	path:04060_4
RELA	path:04060_44
ETS1	path:04060_46
NFATC2	path:04060_46
SP1	path:04060_46
CEBPB	path:04062_1
RELA	path:04062_1
RBPJ	path:04062_11
CEBPB	path:04062_2
RELA	path:04062_2

THRB	path:04080_2
NFIC	path:04080_5
TFAP2A	path:04080_5
AR	path:04110_1
ATF2	path:04110_1
BCL6	path:04110_1
E2F1	path:04110_1
KLF4	path:04110_1
MYC	path:04110_1
NFYA	path:04110_1
POU2F1	path:04110_1
RELA	path:04110_1
SP1	path:04110_1
SP3	path:04110_1
TP53	path:04110_1
TP63	path:04110_1
YY1	path:04110_1
ZBTB7A	path:04110_1
AR	path:04110_10
ATF2	path:04110_10
BCL6	path:04110_10
E2F1	path:04110_10
KLF4	path:04110_10
MYC	path:04110_10
NFYA	path:04110_10
POU2F1	path:04110_10
RELA	path:04110_10
SMAD2	path:04110_10
SMAD3	path:04110_10
SMAD4	path:04110_10
SP1	path:04110_10
SP3	path:04110_10
STAT1	path:04110_10
TP53	path:04110_10
TP63	path:04110_10
YY1	path:04110_10
ZBTB7A	path:04110_10
ATF2	path:04110_11
BCL6	path:04110_11
E2F1	path:04110_11
KLF4	path:04110_11
MYC	path:04110_11
NFYA	path:04110_11
POU2F1	path:04110_11

**RELA**	**path:04110_11**
**SP1**	**path:04110_11**
**SP3**	**path:04110_11**
**STAT1**	**path:04110_11**
**TP53**	**path:04110_11**
**TP63**	**path:04110_11**
**YY1**	**path:04110_11**
**ZBTB7A**	**path:04110_11**
**AR**	**path:04110_13**
**ATF2**	**path:04110_13**
**BCL6**	**path:04110_13**
**E2F1**	**path:04110_13**
**KLF4**	**path:04110_13**
**MYC**	**path:04110_13**
**POU2F1**	**path:04110_13**
**SMAD2**	**path:04110_13**
**SMAD3**	**path:04110_13**
**SMAD4**	**path:04110_13**
**SP1**	**path:04110_13**
**SP3**	**path:04110_13**
**STAT1**	**path:04110_13**
**TCF7L2**	**path:04110_13**
**TP53**	**path:04110_13**
**YY1**	**path:04110_13**
**ZBTB7A**	**path:04110_13**
**ATF2**	**path:04110_2**
**BCL6**	**path:04110_2**
**E2F1**	**path:04110_2**
**MYC**	**path:04110_2**
**NFYA**	**path:04110_2**
**RELA**	**path:04110_2**
**SP1**	**path:04110_2**
**SP3**	**path:04110_2**
**TP53**	**path:04110_2**
**TP63**	**path:04110_2**
**YY1**	**path:04110_2**
**E2F1**	**path:04110_4**
**NFYA**	**path:04110_4**
**SP1**	**path:04110_4**
**SP3**	**path:04110_4**
**ZBTB7A**	**path:04110_4**
**AR**	**path:04110_5**
**ATF2**	**path:04110_5**
**BCL6**	**path:04110_5**

**E2F1**	**path:04110_5**
**KLF4**	**path:04110_5**
**MYC**	**path:04110_5**
**NFYA**	**path:04110_5**
**POU2F1**	**path:04110_5**
**RELA**	**path:04110_5**
**SP1**	**path:04110_5**
**SP3**	**path:04110_5**
**TP53**	**path:04110_5**
**TP63**	**path:04110_5**
**YY1**	**path:04110_5**
**ZBTB7A**	**path:04110_5**
**E2F1**	**path:04110_6**
**NFYA**	**path:04110_6**
**TP53**	**path:04110_6**
**ATF2**	**path:04110_7**
**BCL6**	**path:04110_7**
**E2F1**	**path:04110_7**
**MYC**	**path:04110_7**
**NFYA**	**path:04110_7**
**RELA**	**path:04110_7**
**SP1**	**path:04110_7**
**SP3**	**path:04110_7**
**TP53**	**path:04110_7**
**TP63**	**path:04110_7**
**AR**	**path:04110_9**
**ATF2**	**path:04110_9**
**BCL6**	**path:04110_9**
**E2F1**	**path:04110_9**
**KLF4**	**path:04110_9**
**MYC**	**path:04110_9**
**NFYA**	**path:04110_9**
**POU2F1**	**path:04110_9**
**RELA**	**path:04110_9**
**SP1**	**path:04110_9**
**SP3**	**path:04110_9**
**STAT1**	**path:04110_9**
**TP53**	**path:04110_9**
**TP63**	**path:04110_9**
**YY1**	**path:04110_9**
**ZBTB7A**	**path:04110_9**
**AR**	**path:04115_2**
**BCL6**	**path:04115_2**
**E2F1**	**path:04115_2**

ESR1	path:04115_2
FOS	path:04115_2
JUN	path:04115_2
KLF4	path:04115_2
NFIC	path:04115_2
NFYA	path:04115_2
RELA	path:04115_2
SMAD4	path:04115_2
SP1	path:04115_2
SP3	path:04115_2
TP53	path:04115_2
TP63	path:04115_2
TP73	path:04115_2
YY1	path:04115_2
EGR1	path:04144_2
SP1	path:04144_2
SP3	path:04144_2
TFAP2A	path:04144_2
SPI1	path:04145_1
SPI1	path:04145_4
STAT1	path:04145_4
BCL6	path:04210_3
RBPJ	path:04210_3
SP1	path:04210_4
E2F1	path:04210_5
RBPJ	path:04210_5
SP1	path:04210_5
BCL6	path:04210_6
RBPJ	path:04210_6
SP1	path:04210_6
RBPJ	path:04210_7
TP53	path:04210_8
CEBPB	path:04210_9
JUN	path:04210_9
SP1	path:04210_9
TP53	path:04210_9
ETS2	path:04310_5
JUN	path:04310_5
LEF1	path:04310_5
SP1	path:04310_5
TCF7L2	path:04310_5
ETS2	path:04310_7
FOS	path:04310_7
JUN	path:04310_7

LEF1	path:04310_7
SP1	path:04310_7
TCF7L2	path:04310_7
GLI3	path:04340_1
EGR1	path:04350_1
POU2F1	path:04350_1
SP1	path:04350_1
TCF7L2	path:04350_1
ZBTB7A	path:04350_1
JUNB	path:04350_2
JUNB	path:04350_3
SMAD4	path:04350_3
ATF2	path:04350_4
SMAD3	path:04350_4
SMAD4	path:04350_4
SP1	path:04350_4
EGR1	path:04350_5
SP1	path:04350_5
NR5A1	path:04350_6
TP53	path:04360_3
PAX6	path:04510_1
SMAD2	path:04510_1
SMAD3	path:04510_1
SP1	path:04510_1
SP3	path:04510_1
ZBTB7B	path:04510_1
E2F1	path:04510_10
GLI1	path:04510_10
SP1	path:04510_10
EGR1	path:04510_12
PAX6	path:04510_12
SP1	path:04510_12
SP3	path:04510_12
TFAP2A	path:04510_12
TP53	path:04510_12
EGR1	path:04510_13
MYC	path:04510_13
PAX6	path:04510_13
SMAD2	path:04510_13
SMAD4	path:04510_13
SP1	path:04510_13
SP3	path:04510_13
TFAP2A	path:04510_13
TP53	path:04510_13

ZBTB7B	path:04510_13
PAX6	path:04510_14
SMAD2	path:04510_14
SMAD3	path:04510_14
SP1	path:04510_14
SP3	path:04510_14
ZBTB7B	path:04510_14
PAX6	path:04510_16
SP1	path:04510_16
SP1	path:04510_17
SP1	path:04510_19
TFAP2A	path:04510_19
TP53	path:04510_19
PAX6	path:04510_3
SP1	path:04510_3
PAX6	path:04510_4
SP1	path:04510_4
SP3	path:04510_4
TFAP2A	path:04510_4
TP53	path:04510_4
EGR1	path:04510_5
PAX6	path:04510_5
SMAD2	path:04510_5
SMAD3	path:04510_5
SMAD4	path:04510_5
SP1	path:04510_5
SP3	path:04510_5
TFAP2A	path:04510_5
ZBTB7B	path:04510_5
EGR1	path:04510_6
NFIC	path:04510_6
PAX6	path:04510_6
SMAD2	path:04510_6
SMAD3	path:04510_6
SMAD4	path:04510_6
SP1	path:04510_6
SP3	path:04510_6
TFAP2A	path:04510_6
TP53	path:04510_6
ZBTB7B	path:04510_6
EGR1	path:04510_7
PAX6	path:04510_7
SMAD2	path:04510_7
SMAD3	path:04510_7

**SMAD4**	**path:04510_7**
**SP1**	**path:04510_7**
**SP3**	**path:04510_7**
**TFAP2A**	**path:04510_7**
**ZBTB7B**	**path:04510_7**
**GLI1**	**path:04510_8**
**EGR1**	**path:04512_1**
**FLI1**	**path:04512_1**
**JUN**	**path:04512_1**
**PAX6**	**path:04512_1**
**SMAD2**	**path:04512_1**
**SMAD3**	**path:04512_1**
**SP1**	**path:04512_1**
**SP3**	**path:04512_1**
**ZBTB7B**	**path:04512_1**
**EGR1**	**path:04512_2**
**JUN**	**path:04512_2**
**NFIC**	**path:04512_2**
**PAX6**	**path:04512_2**
**SMAD2**	**path:04512_2**
**SMAD3**	**path:04512_2**
**SP1**	**path:04512_2**
**SP3**	**path:04512_2**
**ZBTB7B**	**path:04512_2**
**EGR1**	**path:04512_3**
**FLI1**	**path:04512_3**
**JUN**	**path:04512_3**
**PAX6**	**path:04512_3**
**SMAD2**	**path:04512_3**
**SMAD3**	**path:04512_3**
**SP1**	**path:04512_3**
**SP3**	**path:04512_3**
**ZBTB7B**	**path:04512_3**
**PAX6**	**path:04514_17**
**CREB1**	**path:04514_19**
**RFX1**	**path:04514_19**
**RFX2**	**path:04514_19**
**RFX3**	**path:04514_19**
**XBP1**	**path:04514_19**
**PAX6**	**path:04514_5**
**SP1**	**path:04520_2**
**TFAP2A**	**path:04520_2**
**TFAP2A**	**path:04520_3**
**SP1**	**path:04520_5**

TFAP2A	path:04520_5
TFAP2A	path:04520_7
SP1	path:04540_7
CEBPA	path:04610_1
ETS1	path:04610_1
FOXA2	path:04610_1
HNF4A	path:04610_1
SP1	path:04610_1
CEBPA	path:04610_2
ESR1	path:04610_2
ETS1	path:04610_2
FOXA2	path:04610_2
HNF1A	path:04610_2
HNF4A	path:04610_2
SP1	path:04610_2
SP3	path:04610_2
CEBPA	path:04610_3
ETS1	path:04610_3
FOXA2	path:04610_3
HNF1A	path:04610_3
HNF4A	path:04610_3
SP1	path:04610_3
CEBPA	path:04610_4
CEBPB	path:04610_4
ETS1	path:04610_4
FOXA2	path:04610_4
HNF1A	path:04610_4
HNF4A	path:04610_4
SP1	path:04610_4
CEBPA	path:04610_5
FOXA2	path:04610_5
HNF4A	path:04610_5
SP1	path:04610_5
ESR1	path:04610_6
FOS	path:04610_6
HNF1A	path:04610_6
HNF4A	path:04610_6
JUN	path:04610_6
SP1	path:04610_6
SP3	path:04610_6
ESR1	path:04610_7
FOS	path:04610_7
HNF4A	path:04610_7
JUN	path:04610_7

**SP1**	**path:04610_7**
**SP3**	**path:04610_7**
**CREB1**	**path:04612_3**
**RFX1**	**path:04612_3**
**RFX2**	**path:04612_3**
**RFX3**	**path:04612_3**
**XBP1**	**path:04612_3**
**ETS1**	**path:04612_4**
**GATA3**	**path:04612_4**
**RUNX3**	**path:04612_4**
**ATF2**	**path:04612_5**
**JUN**	**path:04612_5**
**REL**	**path:04612_5**
**RELA**	**path:04612_5**
**ATF2**	**path:04620_1**
**CEBPB**	**path:04620_1**
**ETS1**	**path:04620_1**
**IRF1**	**path:04620_1**
**IRF2**	**path:04620_1**
**IRF3**	**path:04620_1**
**IRF5**	**path:04620_1**
**IRF7**	**path:04620_1**
**JUN**	**path:04620_1**
**RBPJ**	**path:04620_1**
**REL**	**path:04620_1**
**RELA**	**path:04620_1**
**SPI1**	**path:04620_1**
**STAT1**	**path:04620_1**
**IRF1**	**path:04620_10**
**RBPJ**	**path:04620_12**
**IRF1**	**path:04620_13**
**IRF1**	**path:04620_2**
**IRF3**	**path:04620_2**
**IRF5**	**path:04620_2**
**IRF7**	**path:04620_2**
**RBPJ**	**path:04620_2**
**ATF2**	**path:04620_3**
**CEBPB**	**path:04620_3**
**ETS1**	**path:04620_3**
**IRF1**	**path:04620_3**
**IRF2**	**path:04620_3**
**IRF3**	**path:04620_3**
**IRF5**	**path:04620_3**
**IRF7**	**path:04620_3**

**JUN**	**path:04620_3**
**RBPJ**	**path:04620_3**
**REL**	**path:04620_3**
**RELA**	**path:04620_3**
**SPI1**	**path:04620_3**
**ATF2**	**path:04620_4**
**CEBPB**	**path:04620_4**
**CREB1**	**path:04620_4**
**IRF1**	**path:04620_4**
**IRF2**	**path:04620_4**
**IRF3**	**path:04620_4**
**IRF5**	**path:04620_4**
**IRF7**	**path:04620_4**
**JUN**	**path:04620_4**
**POU2F1**	**path:04620_4**
**RBPJ**	**path:04620_4**
**REL**	**path:04620_4**
**RELA**	**path:04620_4**
**SPI1**	**path:04620_4**
**STAT1**	**path:04620_4**
**ATF2**	**path:04620_5**
**CEBPB**	**path:04620_5**
**CREB1**	**path:04620_5**
**ETS1**	**path:04620_5**
**FOS**	**path:04620_5**
**IRF1**	**path:04620_5**
**IRF3**	**path:04620_5**
**IRF7**	**path:04620_5**
**JUN**	**path:04620_5**
**RBPJ**	**path:04620_5**
**RELA**	**path:04620_5**
**SP1**	**path:04620_5**
**STAT1**	**path:04620_5**
**ATF2**	**path:04620_6**
**CEBPB**	**path:04620_6**
**CREB1**	**path:04620_6**
**ETS1**	**path:04620_6**
**FOS**	**path:04620_6**
**IRF1**	**path:04620_6**
**IRF3**	**path:04620_6**
**IRF7**	**path:04620_6**
**JUN**	**path:04620_6**
**RBPJ**	**path:04620_6**
**RELA**	**path:04620_6**

SP1	path:04620_6
STAT1	path:04620_6
IRF1	path:04620_7
IRF3	path:04620_7
IRF5	path:04620_7
IRF7	path:04620_7
RELA	path:04620_7
ATF2	path:04620_8
CEBPB	path:04620_8
CREB1	path:04620_8
FOS	path:04620_8
IRF1	path:04620_8
IRF3	path:04620_8
IRF7	path:04620_8
JUN	path:04620_8
RBPJ	path:04620_8
RELA	path:04620_8
SP1	path:04620_8
STAT1	path:04620_8
ATF2	path:04620_9
CEBPB	path:04620_9
CREB1	path:04620_9
ETS1	path:04620_9
FOS	path:04620_9
IRF1	path:04620_9
IRF3	path:04620_9
IRF7	path:04620_9
JUN	path:04620_9
RBPJ	path:04620_9
RELA	path:04620_9
SP1	path:04620_9
STAT1	path:04620_9
RBPJ	path:04621_3
RBPJ	path:04622_2
RBPJ	path:04622_4
RBPJ	path:04622_5
RBPJ	path:04623_2
RBPJ	path:04623_4
BCL6	path:04630_1
SP1	path:04630_1
STAT1	path:04630_1
STAT3	path:04630_1
STAT5A	path:04630_1
STAT5B	path:04630_1

TCF7L2	path:04630_1
SP1	path:04630_2
STAT1	path:04630_2
STAT3	path:04630_2
STAT4	path:04630_2
STAT5A	path:04630_2
STAT5B	path:04630_2
STAT6	path:04630_2
SPI1	path:04650_1
ELF1	path:04650_10
REL	path:04650_13
RELA	path:04650_13
REL	path:04650_14
RELA	path:04650_14
ATF2	path:04650_15
REL	path:04650_15
RELA	path:04650_15
ELF1	path:04650_2
ATF2	path:04650_3
IRF3	path:04650_3
IRF5	path:04650_3
IRF7	path:04650_3
JUN	path:04650_3
RELA	path:04650_3
SP1	path:04650_3
TP53	path:04650_3
YY1	path:04650_3
ELF1	path:04650_4
ATF2	path:04650_5
IRF3	path:04650_5
IRF5	path:04650_5
IRF7	path:04650_5
JUN	path:04650_5
REL	path:04650_5
RELA	path:04650_5
YY1	path:04650_5
SPI1	path:04650_7
ELF1	path:04650_8
ELF1	path:04650_9
RBPJ	path:04660_1
NFIC	path:04660_10
TCF7	path:04660_15
NFIC	path:04660_9
SP1	path:04660_9

RBPJ	path:04662_1
NFIC	path:04662_5
NFIC	path:04662_9
SP1	path:04662_9
ELF1	path:04664_1
POU2F1	path:04664_1
ELF1	path:04664_2
ELF1	path:04664_6
FOS	path:04670_2
JUN	path:04670_2
PAX6	path:04670_2
RELA	path:04670_2
SPI1	path:04670_2
STAT1	path:04670_2
PAX6	path:04670_4
RELA	path:04670_4
SPI1	path:04670_4
STAT1	path:04670_4
RELA	path:04670_6
SPI1	path:04670_6
STAT1	path:04670_6
RELA	path:04670_8
SPI1	path:04670_8
STAT1	path:04670_8
PAX6	path:04670_9
RELA	path:04670_9
SPI1	path:04670_9
STAT1	path:04670_9
RBPJ	path:04722_10
NFIC	path:04722_15
SP1	path:04722_15
TP53	path:04722_15
TP73	path:04722_15
RBPJ	path:04722_16
RBPJ	path:04722_9
PAX6	path:04810_16
SP1	path:04810_16
SP3	path:04810_16
SPI1	path:04810_16
SREBF1	path:04910_1
SREBF1	path:04910_4
SP1	path:04912_7
TFAP2A	path:04912_7
TFAP2C	path:04912_7

**TP53**	**path:04912_7**
**LEF1**	**path:04916_3**
**MITF**	**path:04916_3**
**LEF1**	**path:04916_6**
**MITF**	**path:04916_6**
**LEF1**	**path:04916_8**
**MITF**	**path:04916_8**
**RBPJ**	**path:04920_1**
**FOXO3**	**path:04920_2**
**RBPJ**	**path:04920_6**
**RBPJ**	**path:04920_7**
**JUN**	**path:04940_2**
**SP1**	**path:04940_2**
**HNF4A**	**path:04950_2**
**HNF1A**	**path:04950_3**
**HNF4A**	**path:04950_3**
**SP1**	**path:05014_1**
**E2F1**	**path:05014_2**
**SP1**	**path:05014_2**
**TP53**	**path:05014_2**
**SP1**	**path:05020_1**
**EGR1**	**path:05020_2**
**SP1**	**path:05020_2**
**ELK1**	**path:05020_3**
**SRF**	**path:05020_3**
**ATF2**	**path:05020_4**
**ELK1**	**path:05020_4**
**SRF**	**path:05020_4**
**PAX6**	**path:05100_6**
**SP1**	**path:05100_6**
**PAX6**	**path:05100_7**
**ELK1**	**path:05110_3**
**RBPJ**	**path:05120_1**
**RBPJ**	**path:05120_2**
**JUN**	**path:05120_3**
**RBPJ**	**path:05120_3**
**RBPJ**	**path:05120_4**
**JUN**	**path:05120_7**
**RBPJ**	**path:05120_7**
**RBPJ**	**path:05131_5**
**SPI1**	**path:05140_2**
**SPI1**	**path:05140_3**
**SP1**	**path:05140_4**
**RBPJ**	**path:05140_5**

**SP1**	**path:05140_6**
**ATF2**	**path:05142_11**
**JUN**	**path:05142_11**
**NFATC2**	**path:05142_11**
**SP1**	**path:05142_11**
**RBPJ**	**path:05142_12**
**SP1**	**path:05142_12**
**ATF2**	**path:05142_3**
**CREB1**	**path:05142_3**
**RELA**	**path:05142_3**
**SP1**	**path:05142_3**
**STAT3**	**path:05142_3**
**RBPJ**	**path:05142_5**
**RBPJ**	**path:05142_6**
**SP3**	**path:05144_2**
**KLF4**	**path:05145_11**
**STAT1**	**path:05145_12**
**RBPJ**	**path:05145_4**
**SP1**	**path:05145_6**
**IRF2**	**path:05145_8**
**SP1**	**path:05145_8**
**STAT1**	**path:05145_8**
**SP1**	**path:05146_1**
**SPI1**	**path:05146_1**
**SPI1**	**path:05150_4**
**TP53**	**path:05160_10**
**RBPJ**	**path:05160_12**
**RBPJ**	**path:05160_2**
**IRF1**	**path:05160_4**
**IRF3**	**path:05160_4**
**IRF5**	**path:05160_4**
**IRF7**	**path:05160_4**
**RBPJ**	**path:05160_5**
**RBPJ**	**path:05160_6**
**TP53**	**path:05160_9**
**SP1**	**path:05200_1**
**TP53**	**path:05200_1**
**TP63**	**path:05200_1**
**SP1**	**path:05200_10**
**TFAP2A**	**path:05200_10**
**TP53**	**path:05200_10**
**VDR**	**path:05200_10**
**AR**	**path:05200_12**
**AR**	**path:05200_13**

BCL6	path:05200_13
E2F1	path:05200_13
KLF4	path:05200_13
NFIC	path:05200_13
RBPJ	path:05200_13
RELA	path:05200_13
SP1	path:05200_13
SP3	path:05200_13
TP53	path:05200_13
TP63	path:05200_13
YY1	path:05200_13
AR	path:05200_14
KLF4	path:05200_14
RBPJ	path:05200_14
SP1	path:05200_14
SP3	path:05200_14
TP53	path:05200_14
TP63	path:05200_14
AR	path:05200_15
ATF2	path:05200_15
BCL6	path:05200_15
E2F1	path:05200_15
ESR1	path:05200_15
KLF4	path:05200_15
LEF1	path:05200_15
MYC	path:05200_15
MYOD1	path:05200_15
NFIC	path:05200_15
POU2F1	path:05200_15
RBPJ	path:05200_15
RELA	path:05200_15
SMAD3	path:05200_15
SMAD4	path:05200_15
SP1	path:05200_15
SP3	path:05200_15
STAT1	path:05200_15
STAT3	path:05200_15
TCF7L2	path:05200_15
TP53	path:05200_15
TP63	path:05200_15
YY1	path:05200_15
ZBTB7A	path:05200_15
EGR1	path:05200_16
SP1	path:05200_16

SP3	path:05200_16
STAT3	path:05200_16
TFAP2A	path:05200_16
TP53	path:05200_16
VDR	path:05200_16
ETS2	path:05200_17
SP1	path:05200_17
TFAP2A	path:05200_17
TP53	path:05200_17
ATF2	path:05200_18
E2F1	path:05200_18
ELK1	path:05200_18
LEF1	path:05200_18
MYOD1	path:05200_18
NFIC	path:05200_18
POU2F1	path:05200_18
SMAD3	path:05200_18
SMAD4	path:05200_18
SP1	path:05200_18
SP3	path:05200_18
STAT1	path:05200_18
STAT3	path:05200_18
TCF7L2	path:05200_18
TP53	path:05200_18
YY1	path:05200_18
ZBTB7A	path:05200_18
ETS2	path:05200_19
SP1	path:05200_19
TFAP2A	path:05200_19
TP53	path:05200_19
TP63	path:05200_19
VDR	path:05200_19
AR	path:05200_2
BCL6	path:05200_2
E2F1	path:05200_2
ETS2	path:05200_2
KLF4	path:05200_2
MYC	path:05200_2
NFIC	path:05200_2
RBPJ	path:05200_2
RELA	path:05200_2
SP1	path:05200_2
SP3	path:05200_2
TFAP2A	path:05200_2

TP53	path:05200_2
TP63	path:05200_2
CEBPB	path:05200_20
POU2F1	path:05200_20
RBPJ	path:05200_20
SP1	path:05200_20
SP3	path:05200_20
TP53	path:05200_20
TP63	path:05200_20
BCL6	path:05200_21
E2F1	path:05200_21
ESR1	path:05200_21
KLF4	path:05200_21
LEF1	path:05200_21
MYC	path:05200_21
NFIC	path:05200_21
POU2F1	path:05200_21
RELA	path:05200_21
SMAD2	path:05200_21
SP1	path:05200_21
SP3	path:05200_21
STAT1	path:05200_21
STAT3	path:05200_21
STAT5B	path:05200_21
TCF7L2	path:05200_21
TP53	path:05200_21
TP63	path:05200_21
YY1	path:05200_21
ZBTB7A	path:05200_21
EGR1	path:05200_24
ESR1	path:05200_24
FOS	path:05200_24
JUND	path:05200_24
MYC	path:05200_24
MYOD1	path:05200_24
NFIC	path:05200_24
POU2F1	path:05200_24
SMAD2	path:05200_24
SMAD3	path:05200_24
SMAD4	path:05200_24
SP1	path:05200_24
SP3	path:05200_24
STAT1	path:05200_24
STAT3	path:05200_24

**STAT5B**	**path:05200_24**
**TCF7L2**	**path:05200_24**
**TFAP2A**	**path:05200_24**
**TP53**	**path:05200_24**
**ZBTB7A**	**path:05200_24**
**AR**	**path:05200_25**
**ATF2**	**path:05200_25**
**BCL6**	**path:05200_25**
**CEBPB**	**path:05200_25**
**CREM**	**path:05200_25**
**E2F1**	**path:05200_25**
**EGR1**	**path:05200_25**
**ESR1**	**path:05200_25**
**ETV4**	**path:05200_25**
**FOS**	**path:05200_25**
**FOSL1**	**path:05200_25**
**HIF1A**	**path:05200_25**
**JUN**	**path:05200_25**
**JUND**	**path:05200_25**
**KLF4**	**path:05200_25**
**LEF1**	**path:05200_25**
**MYC**	**path:05200_25**
**MYOD1**	**path:05200_25**
**NFIC**	**path:05200_25**
**POU2F1**	**path:05200_25**
**RBPJ**	**path:05200_25**
**RELA**	**path:05200_25**
**SMAD3**	**path:05200_25**
**SMAD4**	**path:05200_25**
**SP1**	**path:05200_25**
**SP3**	**path:05200_25**
**STAT1**	**path:05200_25**
**STAT3**	**path:05200_25**
**STAT5B**	**path:05200_25**
**TCF7L2**	**path:05200_25**
**TFAP2A**	**path:05200_25**
**TP53**	**path:05200_25**
**TP63**	**path:05200_25**
**YY1**	**path:05200_25**
**ZBTB7A**	**path:05200_25**
**ATF2**	**path:05200_26**
**E2F1**	**path:05200_26**
**ELK1**	**path:05200_26**
**LEF1**	**path:05200_26**

NFIC	path:05200_26
POU2F1	path:05200_26
SMAD2	path:05200_26
SMAD3	path:05200_26
SMAD4	path:05200_26
SP1	path:05200_26
SP3	path:05200_26
STAT1	path:05200_26
STAT3	path:05200_26
STAT5B	path:05200_26
TCF7L2	path:05200_26
TFAP2A	path:05200_26
TP53	path:05200_26
YY1	path:05200_26
ZBTB7A	path:05200_26
GLI3	path:05200_27
GLI3	path:05200_28
EGR1	path:05200_29
SP1	path:05200_29
TFAP2A	path:05200_29
TP53	path:05200_3
TP63	path:05200_3
EGR1	path:05200_30
ESR1	path:05200_30
SMAD2	path:05200_30
SMAD3	path:05200_30
SMAD4	path:05200_30
SP1	path:05200_30
STAT3	path:05200_30
TFAP2A	path:05200_30
EGR1	path:05200_31
SP1	path:05200_31
SP1	path:05200_32
CEBPB	path:05200_33
E2F1	path:05200_33
KLF4	path:05200_33
POU2F1	path:05200_33
RBPJ	path:05200_33
SP1	path:05200_33
SP3	path:05200_33
STAT5B	path:05200_33
TP73	path:05200_33
LEF1	path:05200_34
MYC	path:05200_34

SP1	path:05200_34
SPI1	path:05200_34
TCF7L2	path:05200_34
AR	path:05200_35
ATF2	path:05200_35
CEBPB	path:05200_35
CREM	path:05200_35
E2F1	path:05200_35
EGR1	path:05200_35
ESR1	path:05200_35
ETV4	path:05200_35
FOS	path:05200_35
FOSL1	path:05200_35
JUN	path:05200_35
JUND	path:05200_35
KLF4	path:05200_35
LEF1	path:05200_35
MYC	path:05200_35
POU2F1	path:05200_35
RBPJ	path:05200_35
RELA	path:05200_35
SMAD3	path:05200_35
SMAD4	path:05200_35
SP1	path:05200_35
SP3	path:05200_35
STAT1	path:05200_35
STAT3	path:05200_35
STAT5B	path:05200_35
TCF7L2	path:05200_35
TFAP2A	path:05200_35
YY1	path:05200_35
ZBTB7A	path:05200_35
RORA	path:05200_36
AR	path:05200_37
ATF2	path:05200_37
BCL6	path:05200_37
CEBPB	path:05200_37
CREM	path:05200_37
E2F1	path:05200_37
EGR1	path:05200_37
ESR1	path:05200_37
ETV4	path:05200_37
FOS	path:05200_37
HIF1A	path:05200_37

**JUN**	**path:05200_37**
**JUND**	**path:05200_37**
**KLF4**	**path:05200_37**
**LEF1**	**path:05200_37**
**MYC**	**path:05200_37**
**NFIC**	**path:05200_37**
**POU2F1**	**path:05200_37**
**RBPJ**	**path:05200_37**
**RELA**	**path:05200_37**
**SMAD3**	**path:05200_37**
**SMAD4**	**path:05200_37**
**SP1**	**path:05200_37**
**SP3**	**path:05200_37**
**STAT1**	**path:05200_37**
**STAT3**	**path:05200_37**
**STAT5B**	**path:05200_37**
**TCF7L2**	**path:05200_37**
**TFAP2A**	**path:05200_37**
**TP53**	**path:05200_37**
**TP63**	**path:05200_37**
**YY1**	**path:05200_37**
**ZBTB7A**	**path:05200_37**
**AR**	**path:05200_38**
**ATF2**	**path:05200_38**
**CREM**	**path:05200_38**
**E2F1**	**path:05200_38**
**ESR1**	**path:05200_38**
**ETV4**	**path:05200_38**
**FOS**	**path:05200_38**
**FOSL1**	**path:05200_38**
**JUN**	**path:05200_38**
**JUND**	**path:05200_38**
**KLF4**	**path:05200_38**
**LEF1**	**path:05200_38**
**MYC**	**path:05200_38**
**MYOD1**	**path:05200_38**
**POU2F1**	**path:05200_38**
**RELA**	**path:05200_38**
**SMAD3**	**path:05200_38**
**SMAD4**	**path:05200_38**
**SP1**	**path:05200_38**
**SP3**	**path:05200_38**
**STAT1**	**path:05200_38**
**STAT3**	**path:05200_38**

STAT5B	path:05200_38
TCF7L2	path:05200_38
TFAP2A	path:05200_38
TP53	path:05200_38
YY1	path:05200_38
ZBTB7A	path:05200_38
AR	path:05200_39
ATF2	path:05200_39
BCL6	path:05200_39
CEBPB	path:05200_39
CREM	path:05200_39
E2F1	path:05200_39
ESR1	path:05200_39
ETV4	path:05200_39
FOS	path:05200_39
FOSL1	path:05200_39
HIF1A	path:05200_39
JUN	path:05200_39
JUND	path:05200_39
KLF4	path:05200_39
LEF1	path:05200_39
MYC	path:05200_39
MYOD1	path:05200_39
NFIC	path:05200_39
POU2F1	path:05200_39
RBPJ	path:05200_39
RELA	path:05200_39
SMAD3	path:05200_39
SMAD4	path:05200_39
SP1	path:05200_39
SP3	path:05200_39
STAT1	path:05200_39
STAT3	path:05200_39
STAT5B	path:05200_39
TCF7L2	path:05200_39
TFAP2A	path:05200_39
TP53	path:05200_39
TP63	path:05200_39
YY1	path:05200_39
ZBTB7A	path:05200_39
JUN	path:05200_41
SP1	path:05200_41
SP1	path:05200_42
NFIC	path:05200_6

SP1	path:05200_6
TFAP2A	path:05200_6
TP53	path:05200_6
AR	path:05200_7
NFIC	path:05200_7
SP1	path:05200_7
TFAP2A	path:05200_7
TP53	path:05200_7
TP63	path:05200_7
VDR	path:05200_7
SP1	path:05200_8
TFAP2A	path:05200_8
TP53	path:05200_8
AR	path:05200_9
ETS2	path:05200_9
KLF4	path:05200_9
SP1	path:05200_9
SP3	path:05200_9
TFAP2A	path:05200_9
TP53	path:05200_9
TP63	path:05200_9
ATF2	path:05210_5
CREM	path:05210_5
KLF4	path:05210_5
LEF1	path:05210_5
MYC	path:05210_5
SMAD3	path:05210_5
SMAD4	path:05210_5
SP1	path:05210_5
STAT1	path:05210_5
STAT3	path:05210_5
TCF7L2	path:05210_5
ATF2	path:05210_8
CREM	path:05210_8
KLF4	path:05210_8
LEF1	path:05210_8
MYC	path:05210_8
SMAD3	path:05210_8
SMAD4	path:05210_8
SP1	path:05210_8
STAT1	path:05210_8
STAT3	path:05210_8
TCF7L2	path:05210_8
SMAD4	path:05211_5

**ETS1**	**path:05212_1**
**SP1**	**path:05212_10**
**ETS1**	**path:05212_2**
**EGR1**	**path:05212_3**
**ETS1**	**path:05212_3**
**SP1**	**path:05212_3**
**TFAP2A**	**path:05212_3**
**ETS1**	**path:05212_6**
**EGR1**	**path:05212_8**
**ESR1**	**path:05212_8**
**ETS1**	**path:05212_8**
**SP1**	**path:05212_8**
**TFAP2A**	**path:05212_8**
**ATF2**	**path:05212_9**
**E2F1**	**path:05212_9**
**MYC**	**path:05212_9**
**SP1**	**path:05212_9**
**SP3**	**path:05212_9**
**YY1**	**path:05212_9**
**LEF1**	**path:05213_1**
**MYC**	**path:05213_1**
**STAT1**	**path:05213_1**
**TCF7L2**	**path:05213_1**
**TP53**	**path:05213_3**
**SP1**	**path:05214_1**
**TFAP2A**	**path:05214_1**
**TP53**	**path:05214_1**
**SP1**	**path:05214_2**
**TFAP2A**	**path:05214_2**
**TP53**	**path:05214_2**
**AR**	**path:05214_3**
**ATF2**	**path:05214_3**
**E2F1**	**path:05214_3**
**KLF4**	**path:05214_3**
**MYC**	**path:05214_3**
**NFIC**	**path:05214_3**
**SP1**	**path:05214_3**
**SP3**	**path:05214_3**
**STAT1**	**path:05214_3**
**STAT5B**	**path:05214_3**
**TP53**	**path:05214_3**
**TP63**	**path:05214_3**
**YY1**	**path:05214_3**
**ZBTB7A**	**path:05214_3**

AR	path:05215_1
RBPJ	path:05215_10
SP1	path:05215_10
TFAP2A	path:05215_10
TP53	path:05215_10
TP63	path:05215_10
RBPJ	path:05215_2
E2F1	path:05215_3
ETS1	path:05215_4
RBPJ	path:05215_4
SP1	path:05215_4
TP53	path:05215_4
TP63	path:05215_4
LEF1	path:05215_5
SP1	path:05215_5
TCF7L2	path:05215_5
SP1	path:05215_6
TP53	path:05215_6
TP63	path:05215_6
EGR1	path:05215_7
SP1	path:05215_7
TFAP2A	path:05215_7
TP53	path:05215_7
TFAP2A	path:05215_8
AR	path:05215_9
EGR1	path:05215_9
ETS1	path:05215_9
SP1	path:05215_9
TFAP2A	path:05215_9
TP53	path:05215_9
TP63	path:05215_9
AR	path:05216_3
LEF1	path:05216_3
MYC	path:05216_3
POU2F1	path:05216_3
SP1	path:05216_3
STAT1	path:05216_3
TCF7L2	path:05216_3
GLI3	path:05217_1
GLI3	path:05217_2
NFIC	path:05218_1
SP1	path:05218_1
SP3	path:05218_1
STAT5B	path:05218_1

**TP53**	**path:05218_1**
**NFIC**	**path:05218_2**
**SP1**	**path:05218_2**
**SP3**	**path:05218_2**
**STAT5B**	**path:05218_2**
**TP53**	**path:05218_2**
**E2F1**	**path:05218_3**
**MYC**	**path:05218_3**
**NFIC**	**path:05218_3**
**SP1**	**path:05218_3**
**SP3**	**path:05218_3**
**STAT5B**	**path:05218_3**
**TP53**	**path:05218_3**
**TP63**	**path:05218_3**
**YY1**	**path:05218_3**
**ZBTB7A**	**path:05218_3**
**EGR1**	**path:05218_4**
**SMAD4**	**path:05218_4**
**SP1**	**path:05218_4**
**STAT3**	**path:05218_4**
**E2F1**	**path:05218_5**
**MYC**	**path:05218_5**
**SP1**	**path:05218_5**
**SP3**	**path:05218_5**
**STAT5B**	**path:05218_5**
**TP53**	**path:05218_5**
**TP63**	**path:05218_5**
**YY1**	**path:05218_5**
**ZBTB7A**	**path:05218_5**
**TFAP2A**	**path:05219_2**
**TFAP2C**	**path:05219_2**
**AR**	**path:05219_3**
**ATF2**	**path:05219_3**
**E2F1**	**path:05219_3**
**ESR1**	**path:05219_3**
**FOS**	**path:05219_3**
**KLF4**	**path:05219_3**
**MYC**	**path:05219_3**
**NFIC**	**path:05219_3**
**POU2F1**	**path:05219_3**
**RELA**	**path:05219_3**
**SP1**	**path:05219_3**
**SP3**	**path:05219_3**
**STAT1**	**path:05219_3**

**STAT5B**	**path:05219_3**
**TP53**	**path:05219_3**
**TP63**	**path:05219_3**
**YY1**	**path:05219_3**
**ZBTB7A**	**path:05219_3**
**AR**	**path:05219_5**
**ATF2**	**path:05219_5**
**E2F1**	**path:05219_5**
**KLF4**	**path:05219_5**
**MYC**	**path:05219_5**
**POU2F1**	**path:05219_5**
**SP1**	**path:05219_5**
**SP3**	**path:05219_5**
**STAT1**	**path:05219_5**
**STAT5B**	**path:05219_5**
**TP53**	**path:05219_5**
**TP63**	**path:05219_5**
**YY1**	**path:05219_5**
**ZBTB7A**	**path:05219_5**
**AR**	**path:05220_4**
**ATF2**	**path:05220_4**
**E2F1**	**path:05220_4**
**KLF4**	**path:05220_4**
**MYC**	**path:05220_4**
**NFIC**	**path:05220_4**
**SP1**	**path:05220_4**
**SP3**	**path:05220_4**
**STAT1**	**path:05220_4**
**STAT5B**	**path:05220_4**
**TP53**	**path:05220_4**
**TP63**	**path:05220_4**
**YY1**	**path:05220_4**
**ZBTB7A**	**path:05220_4**
**SP1**	**path:05220_5**
**RBPJ**	**path:05220_6**
**BCL6**	**path:05220_8**
**SP1**	**path:05220_8**
**SP3**	**path:05220_8**
**STAT3**	**path:05221_1**
**AR**	**path:05221_2**
**LEF1**	**path:05221_2**
**MYC**	**path:05221_2**
**SP1**	**path:05221_2**
**TCF7L2**	**path:05221_2**

E2F1	path:05222_1
LEF1	path:05222_1
MYC	path:05222_1
POU2F1	path:05222_1
SP1	path:05222_1
SP3	path:05222_1
TCF7L2	path:05222_1
ZBTB7A	path:05222_1
SP1	path:05222_2
ATF2	path:05222_5
E2F1	path:05222_5
LEF1	path:05222_5
MYC	path:05222_5
POU2F1	path:05222_5
SP1	path:05222_5
STAT1	path:05222_5
TCF7L2	path:05222_5
ZBTB7A	path:05222_5
RBPJ	path:05222_7
RBPJ	path:05222_8
SP1	path:05223_1
TFAP2A	path:05223_1
TP53	path:05223_2
ATF2	path:05223_3
E2F1	path:05223_3
MYC	path:05223_3
SP1	path:05223_3
SP3	path:05223_3
E2F1	path:05223_4
JUN	path:05320_2
SP1	path:05320_2
JUN	path:05330_2
SP1	path:05330_2
JUN	path:05332_1
SP1	path:05332_1
SRF	path:05410_1
PAX6	path:05414_2
SP1	path:05414_2
SP3	path:05414_2
SRF	path:05416_1

Table S8. The detailed information of the co-family TFs regulated sub-pathways of TSN with k=4.

Family	TF	Subpathway
Steroid hormone receptors (NR3)	AR	path:05200_35
Steroid hormone receptors (NR3)	AR	path:05200_15
Steroid hormone receptors (NR3)	AR	path:04110_10
Steroid hormone receptors (NR3)	AR	path:05215_9
Steroid hormone receptors (NR3)	AR	path:05216_3
Steroid hormone receptors (NR3)	AR	path:05219_5
Steroid hormone receptors (NR3)	AR	path:05200_7
Steroid hormone receptors (NR3)	AR	path:05200_38
Steroid hormone receptors (NR3)	AR	path:05215_1
Steroid hormone receptors (NR3)	AR	path:05214_3
Steroid hormone receptors (NR3)	AR	path:05200_13
Steroid hormone receptors (NR3)	AR	path:05200_14
Steroid hormone receptors (NR3)	AR	path:05200_12
Steroid hormone receptors (NR3)	AR	path:04110_1
Steroid hormone receptors (NR3)	AR	path:05200_37
Steroid hormone receptors (NR3)	AR	path:05220_4
Steroid hormone receptors (NR3)	AR	path:04110_13
Steroid hormone receptors (NR3)	AR	path:05200_25
Steroid hormone receptors (NR3)	AR	path:05200_9
Steroid hormone receptors (NR3)	AR	path:05200_39
Steroid hormone receptors (NR3)	AR	path:04110_9
Steroid hormone receptors (NR3)	AR	path:05219_3
Steroid hormone receptors (NR3)	AR	path:04110_5
Steroid hormone receptors (NR3)	AR	path:05221_2
Steroid hormone receptors (NR3)	AR	path:05200_2
Steroid hormone receptors (NR3)	AR	path:04115_2
AP 1	ATF2	path:04650_5
AP 1	ATF2	path:04620_6
AP 1	ATF2	path:04620_8
AP 1	ATF2	path:05200_35
AP 1	ATF2	path:04620_9
AP 1	ATF2	path:04110_7
AP 1	ATF2	path:04620_3
AP 1	ATF2	path:04612_5
AP 1	ATF2	path:05200_25
AP 1	ATF2	path:05200_38
AP 1	ATF2	path:05200_15
AP 1	ATF2	path:00140_1
AP 1	ATF2	path:05210_5
AP 1	ATF2	path:05142_3
AP 1	ATF2	path:04110_11
AP 1	ATF2	path:04650_15

**AP 1**	**ATF2**	**path:05200_18**
**AP 1**	**ATF2**	**path:04110_1**
**AP 1**	**ATF2**	**path:05200_37**
**AP 1**	**ATF2**	**path:04110_5**
**AP 1**	**ATF2**	**path:04620_4**
**AP 1**	**ATF2**	**path:04110_9**
**AP 1**	**ATF2**	**path:04110_2**
**AP 1**	**ATF2**	**path:04110_10**
**AP 1**	**ATF2**	**path:05222_5**
**AP 1**	**ATF2**	**path:04010_11**
**AP 1**	**ATF2**	**path:04110_13**
**AP 1**	**ATF2**	**path:05200_39**
**AP 1**	**ATF2**	**path:05020_4**
**AP 1**	**ATF2**	**path:05220_4**
**AP 1**	**ATF2**	**path:05223_3**
**AP 1**	**ATF2**	**path:04010_10**
**AP 1**	**ATF2**	**path:05200_26**
**AP 1**	**ATF2**	**path:05219_5**
**AP 1**	**ATF2**	**path:04620_1**
**AP 1**	**ATF2**	**path:05214_3**
**AP 1**	**ATF2**	**path:04620_5**
**AP 1**	**ATF2**	**path:05210_8**
**AP 1**	**ATF2**	**path:04650_3**
**AP 1**	**ATF2**	**path:05212_9**
**AP 1**	**ATF2**	**path:04350_4**
**AP 1**	**ATF2**	**path:05219_3**
**AP 1**	**ATF2**	**path:04010_6**
**AP 1**	**ATF2**	**path:05142_11**
**C/EBP like factors**	**CEBPA**	**path:04610_1**
**C/EBP like factors**	**CEBPA**	**path:04060_37**
**C/EBP like factors**	**CEBPA**	**path:04610_2**
**C/EBP like factors**	**CEBPA**	**path:04610_3**
**C/EBP like factors**	**CEBPA**	**path:04610_4**
**C/EBP like factors**	**CEBPA**	**path:04610_5**
**C/EBP like factors**	**CEBPB**	**path:04210_9**
**C/EBP like factors**	**CEBPB**	**path:04010_18**
**C/EBP like factors**	**CEBPB**	**path:05200_37**
**C/EBP like factors**	**CEBPB**	**path:04620_1**
**C/EBP like factors**	**CEBPB**	**path:04620_8**
**C/EBP like factors**	**CEBPB**	**path:04620_6**
**C/EBP like factors**	**CEBPB**	**path:04610_4**
**C/EBP like factors**	**CEBPB**	**path:04620_4**
**C/EBP like factors**	**CEBPB**	**path:04060_38**
**C/EBP like factors**	**CEBPB**	**path:04062_2**

**C/EBP like factors**	**CEBPB**	**path:05200_20**
**C/EBP like factors**	**CEBPB**	**path:04620_9**
**C/EBP like factors**	**CEBPB**	**path:05200_39**
**C/EBP like factors**	**CEBPB**	**path:05200_25**
**C/EBP like factors**	**CEBPB**	**path:04062_1**
**C/EBP like factors**	**CEBPB**	**path:04620_5**
**C/EBP like factors**	**CEBPB**	**path:05200_33**
**C/EBP like factors**	**CEBPB**	**path:04620_3**
**C/EBP like factors**	**CEBPB**	**path:05200_35**
**CREB**	**CREB1**	**path:04514_19**
**CREB**	**CREB1**	**path:04620_9**
**CREB**	**CREB1**	**path:04620_6**
**CREB**	**CREB1**	**path:04620_4**
**CREB**	**CREB1**	**path:04620_8**
**CREB**	**CREB1**	**path:05142_3**
**CREB**	**CREB1**	**path:04620_5**
**CREB**	**CREB1**	**path:04612_3**
**CREB**	**CREM**	**path:05210_5**
**CREB**	**CREM**	**path:05200_35**
**CREB**	**CREM**	**path:05200_38**
**CREB**	**CREM**	**path:05200_39**
**CREB**	**CREM**	**path:05200_25**
**CREB**	**CREM**	**path:05200_37**
**CREB**	**CREM**	**path:05210_8**
**Cell cycle controlling factors**	**E2F1**	**path:05220_4**
**Cell cycle controlling factors**	**E2F1**	**path:05218_3**
**Cell cycle controlling factors**	**E2F1**	**path:05200_15**
**Cell cycle controlling factors**	**E2F1**	**path:04110_9**
**Cell cycle controlling factors**	**E2F1**	**path:04110_2**
**Cell cycle controlling factors**	**E2F1**	**path:04110_13**
**Cell cycle controlling factors**	**E2F1**	**path:04110_6**
**Cell cycle controlling factors**	**E2F1**	**path:04110_11**
**Cell cycle controlling factors**	**E2F1**	**path:05200_2**
**Cell cycle controlling factors**	**E2F1**	**path:05200_13**
**Cell cycle controlling factors**	**E2F1**	**path:05214_3**
**Cell cycle controlling factors**	**E2F1**	**path:04110_7**
**Cell cycle controlling factors**	**E2F1**	**path:04110_4**
**Cell cycle controlling factors**	**E2F1**	**path:05200_18**
**Cell cycle controlling factors**	**E2F1**	**path:05223_3**
**Cell cycle controlling factors**	**E2F1**	**path:04110_1**
**Cell cycle controlling factors**	**E2F1**	**path:05219_5**
**Cell cycle controlling factors**	**E2F1**	**path:05223_4**
**Cell cycle controlling factors**	**E2F1**	**path:05200_25**
**Cell cycle controlling factors**	**E2F1**	**path:05200_39**

**Cell cycle controlling factors**	**E2F1**	**path:05200_26**
**Cell cycle controlling factors**	**E2F1**	**path:05200_38**
**Cell cycle controlling factors**	**E2F1**	**path:05219_3**
**Cell cycle controlling factors**	**E2F1**	**path:05212_9**
**Cell cycle controlling factors**	**E2F1**	**path:04210_5**
**Cell cycle controlling factors**	**E2F1**	**path:05222_5**
**Cell cycle controlling factors**	**E2F1**	**path:05222_1**
**Cell cycle controlling factors**	**E2F1**	**path:04110_5**
**Cell cycle controlling factors**	**E2F1**	**path:05200_21**
**Cell cycle controlling factors**	**E2F1**	**path:04115_2**
**Cell cycle controlling factors**	**E2F1**	**path:04510_10**
**Cell cycle controlling factors**	**E2F1**	**path:05200_37**
**Cell cycle controlling factors**	**E2F1**	**path:05014_2**
**Cell cycle controlling factors**	**E2F1**	**path:05200_35**
**Cell cycle controlling factors**	**E2F1**	**path:04110_10**
**Cell cycle controlling factors**	**E2F1**	**path:05218_5**
**Cell cycle controlling factors**	**E2F1**	**path:05200_33**
**Cell cycle controlling factors**	**E2F1**	**path:05215_3**
**Developmental / cell cycle regulators**	**EGR1**	**path:05200_30**
**Developmental / cell cycle regulators**	**EGR1**	**path:04144_2**
**Developmental / cell cycle regulators**	**EGR1**	**path:04510_12**
**Developmental / cell cycle regulators**	**EGR1**	**path:04510_5**
**Developmental / cell cycle regulators**	**EGR1**	**path:05218_4**
**Developmental / cell cycle regulators**	**EGR1**	**path:04510_7**
**Developmental / cell cycle regulators**	**EGR1**	**path:04510_6**
**Developmental / cell cycle regulators**	**EGR1**	**path:05200_16**
**Developmental / cell cycle regulators**	**EGR1**	**path:05200_35**
**Developmental / cell cycle regulators**	**EGR1**	**path:05200_25**
**Developmental / cell cycle regulators**	**EGR1**	**path:05200_24**
**Developmental / cell cycle regulators**	**EGR1**	**path:05200_29**
**Developmental / cell cycle regulators**	**EGR1**	**path:05212_3**
**Developmental / cell cycle regulators**	**EGR1**	**path:05215_9**
**Developmental / cell cycle regulators**	**EGR1**	**path:05020_2**
**Developmental / cell cycle regulators**	**EGR1**	**path:05212_8**
**Developmental / cell cycle regulators**	**EGR1**	**path:04350_1**
**Developmental / cell cycle regulators**	**EGR1**	**path:05215_7**
**Developmental / cell cycle regulators**	**EGR1**	**path:04512_3**
**Developmental / cell cycle regulators**	**EGR1**	**path:04512_1**
**Developmental / cell cycle regulators**	**EGR1**	**path:05200_31**
**Developmental / cell cycle regulators**	**EGR1**	**path:04512_2**
**Developmental / cell cycle regulators**	**EGR1**	**path:04350_5**
**Developmental / cell cycle regulators**	**EGR1**	**path:04510_13**
**Developmental / cell cycle regulators**	**EGR1**	**path:05200_37**
**Ets type**	**ELF1**	**path:04060_34**

**Ets type**	**ELF1**	**path:04650_4**
**Ets type**	**ELF1**	**path:04664_1**
**Ets type**	**ELF1**	**path:04060_1**
**Ets type**	**ELF1**	**path:04650_8**
**Ets type**	**ELF1**	**path:04650_9**
**Ets type**	**ELF1**	**path:04664_6**
**Ets type**	**ELF1**	**path:04650_2**
**Ets type**	**ELF1**	**path:04664_2**
**Ets type**	**ELF1**	**path:00920_1**
**Ets type**	**ELF1**	**path:04650_10**
**Steroid hormone receptors (NR3)**	**ESR1**	**path:04610_2**
**Steroid hormone receptors (NR3)**	**ESR1**	**path:05200_39**
**Steroid hormone receptors (NR3)**	**ESR1**	**path:00982_2**
**Steroid hormone receptors (NR3)**	**ESR1**	**path:05200_25**
**Steroid hormone receptors (NR3)**	**ESR1**	**path:05200_38**
**Steroid hormone receptors (NR3)**	**ESR1**	**path:05212_8**
**Steroid hormone receptors (NR3)**	**ESR1**	**path:05200_37**
**Steroid hormone receptors (NR3)**	**ESR1**	**path:05200_35**
**Steroid hormone receptors (NR3)**	**ESR1**	**path:05200_15**
**Steroid hormone receptors (NR3)**	**ESR1**	**path:05219_3**
**Steroid hormone receptors (NR3)**	**ESR1**	**path:05200_24**
**Steroid hormone receptors (NR3)**	**ESR1**	**path:00830_1**
**Steroid hormone receptors (NR3)**	**ESR1**	**path:04115_2**
**Steroid hormone receptors (NR3)**	**ESR1**	**path:05200_21**
**Steroid hormone receptors (NR3)**	**ESR1**	**path:00980_1**
**Steroid hormone receptors (NR3)**	**ESR1**	**path:05200_30**
**Steroid hormone receptors (NR3)**	**ESR1**	**path:04610_6**
**Steroid hormone receptors (NR3)**	**ESR1**	**path:04610_7**
**Ets type**	**ETV4**	**path:05200_35**
**Ets type**	**ETV4**	**path:05200_25**
**Ets type**	**ETV4**	**path:05200_37**
**Ets type**	**ETV4**	**path:05200_38**
**Ets type**	**ETV4**	**path:05200_39**
**AP 1**	**FOS**	**path:04620_6**
**AP 1**	**FOS**	**path:04620_9**
**AP 1**	**FOS**	**path:04610_6**
**AP 1**	**FOS**	**path:04310_7**
**AP 1**	**FOS**	**path:05200_35**
**AP 1**	**FOS**	**path:04620_5**
**AP 1**	**FOS**	**path:05200_38**
**AP 1**	**FOS**	**path:05200_25**
**AP 1**	**FOS**	**path:04620_8**
**AP 1**	**FOS**	**path:05200_37**
**AP 1**	**FOS**	**path:05200_24**

AP 1	FOS	path:04610_7
AP 1	FOS	path:05219_3
AP 1	FOS	path:05200_39
AP 1	FOS	path:04115_2
AP 1	FOS	path:04670_2
Tissue specific regulators	FOXA2	path:04610_5
Tissue specific regulators	FOXA2	path:04610_1
Tissue specific regulators	FOXA2	path:00980_1
Tissue specific regulators	FOXA2	path:04610_3
Tissue specific regulators	FOXA2	path:04610_2
Tissue specific regulators	FOXA2	path:04610_4
Tissue specific regulators	FOXA3	path:00830_1
Tissue specific regulators	FOXA3	path:00140_1
Tissue specific regulators	FOXA3	path:00980_1
Tissue specific regulators	FOXA3	path:00591_1
Tissue specific regulators	FOXA3	path:00982_2
GATA Factors	GATA1	path:04060_32
GATA Factors	GATA2	path:04060_32
GATA Factors	GATA3	path:04612_4
GATA Factors	GATA4	path:00590_1
GATA Factors	GATA4	path:00982_2
GATA Factors	GATA4	path:00590_2
GATA Factors	GATA4	path:00980_1
GATA Factors	GATA4	path:00591_1
GATA Factors	GATA6	path:00590_1
GATA Factors	GATA6	path:00590_2
GATA Factors	GATA6	path:00591_1
Developmental / cell cycle regulators	GLI1	path:04510_10
Developmental / cell cycle regulators	GLI1	path:04510_8
Developmental / cell cycle regulators	GLI3	path:05217_1
Developmental / cell cycle regulators	GLI3	path:04340_1
Developmental / cell cycle regulators	GLI3	path:05200_27
Developmental / cell cycle regulators	GLI3	path:05200_28
Developmental / cell cycle regulators	GLI3	path:05217_2
Homeo domain only	HNF1A	path:04610_3
Homeo domain only	HNF1A	path:00982_2
Homeo domain only	HNF1A	path:04950_3
Homeo domain only	HNF1A	path:04610_6
Homeo domain only	HNF1A	path:04610_2
Homeo domain only	HNF1A	path:04610_4
Homeo domain only	HNF1A	path:00140_1
Homeo domain only	HNF1A	path:00980_1
Steroid hormone receptors (NR3)	HNF4A	path:00120_1
Steroid hormone receptors (NR3)	HNF4A	path:04950_3

**Steroid hormone receptors (NR3)**	**HNF4A**	**path:00982_2**
**Steroid hormone receptors (NR3)**	**HNF4A**	**path:00561_2**
**Steroid hormone receptors (NR3)**	**HNF4A**	**path:04610_1**
**Steroid hormone receptors (NR3)**	**HNF4A**	**path:00590_1**
**Steroid hormone receptors (NR3)**	**HNF4A**	**path:00010_1**
**Steroid hormone receptors (NR3)**	**HNF4A**	**path:00591_1**
**Steroid hormone receptors (NR3)**	**HNF4A**	**path:04950_2**
**Steroid hormone receptors (NR3)**	**HNF4A**	**path:04610_6**
**Steroid hormone receptors (NR3)**	**HNF4A**	**path:04610_7**
**Steroid hormone receptors (NR3)**	**HNF4A**	**path:04610_2**
**Steroid hormone receptors (NR3)**	**HNF4A**	**path:00590_2**
**Steroid hormone receptors (NR3)**	**HNF4A**	**path:00140_1**
**Steroid hormone receptors (NR3)**	**HNF4A**	**path:04610_5**
**Steroid hormone receptors (NR3)**	**HNF4A**	**path:00980_1**
**Steroid hormone receptors (NR3)**	**HNF4A**	**path:04610_4**
**Steroid hormone receptors (NR3)**	**HNF4A**	**path:04610_3**
**Steroid hormone receptors (NR3)**	**HNF4A**	**path:03320_1**
**Steroid hormone receptors (NR3)**	**HNF4A**	**path:00830_1**
**Interferon regulating factors**	**IRF1**	**path:04620_3**
**Interferon regulating factors**	**IRF1**	**path:04620_4**
**Interferon regulating factors**	**IRF1**	**path:04060_1**
**Interferon regulating factors**	**IRF1**	**path:04620_6**
**Interferon regulating factors**	**IRF1**	**path:04620_2**
**Interferon regulating factors**	**IRF1**	**path:04620_5**
**Interferon regulating factors**	**IRF1**	**path:04620_9**
**Interferon regulating factors**	**IRF1**	**path:04620_7**
**Interferon regulating factors**	**IRF1**	**path:04060_35**
**Interferon regulating factors**	**IRF1**	**path:04620_10**
**Interferon regulating factors**	**IRF1**	**path:04620_8**
**Interferon regulating factors**	**IRF1**	**path:04620_1**
**Interferon regulating factors**	**IRF1**	**path:04620_13**
**Interferon regulating factors**	**IRF1**	**path:05160_4**
**Interferon regulating factors**	**IRF3**	**path:04620_4**
**Interferon regulating factors**	**IRF3**	**path:04650_5**
**Interferon regulating factors**	**IRF3**	**path:04620_6**
**Interferon regulating factors**	**IRF3**	**path:04650_3**
**Interferon regulating factors**	**IRF3**	**path:04060_23**
**Interferon regulating factors**	**IRF3**	**path:04620_9**
**Interferon regulating factors**	**IRF3**	**path:04620_5**
**Interferon regulating factors**	**IRF3**	**path:05160_4**
**Interferon regulating factors**	**IRF3**	**path:04620_1**
**Interferon regulating factors**	**IRF3**	**path:04620_8**
**Interferon regulating factors**	**IRF3**	**path:04620_3**
**Interferon regulating factors**	**IRF3**	**path:04620_7**

Interferon regulating factors	IRF3	path:04620_2
Interferon regulating factors	IRF5	path:04620_4
Interferon regulating factors	IRF5	path:04620_2
Interferon regulating factors	IRF5	path:04650_5
Interferon regulating factors	IRF5	path:05160_4
Interferon regulating factors	IRF5	path:04620_7
Interferon regulating factors	IRF5	path:04650_3
Interferon regulating factors	IRF5	path:04620_1
Interferon regulating factors	IRF5	path:04060_23
Interferon regulating factors	IRF5	path:04620_3
Interferon regulating factors	IRF7	path:04620_8
Interferon regulating factors	IRF7	path:04620_5
Interferon regulating factors	IRF7	path:04620_7
Interferon regulating factors	IRF7	path:05160_4
Interferon regulating factors	IRF7	path:04650_3
Interferon regulating factors	IRF7	path:04620_2
Interferon regulating factors	IRF7	path:04060_23
Interferon regulating factors	IRF7	path:04620_1
Interferon regulating factors	IRF7	path:04620_4
Interferon regulating factors	IRF7	path:04620_6
Interferon regulating factors	IRF7	path:04650_5
Interferon regulating factors	IRF7	path:04620_9
Interferon regulating factors	IRF7	path:04620_3
AP 1	JUN	path:05142_11
AP 1	JUN	path:04620_1
AP 1	JUN	path:05200_38
AP 1	JUN	path:04512_1
AP 1	JUN	path:04620_3
AP 1	JUN	path:04670_2
AP 1	JUN	path:05120_3
AP 1	JUN	path:05200_39
AP 1	JUN	path:04940_2
AP 1	JUN	path:04650_5
AP 1	JUN	path:04310_7
AP 1	JUN	path:04115_2
AP 1	JUN	path:04650_3
AP 1	JUN	path:04610_6
AP 1	JUN	path:04620_9
AP 1	JUN	path:04210_9
AP 1	JUN	path:05200_35
AP 1	JUN	path:04620_5
AP 1	JUN	path:05332_1
AP 1	JUN	path:04620_4
AP 1	JUN	path:05200_37

AP 1	JUN	path:04310_5
AP 1	JUN	path:05120_7
AP 1	JUN	path:04620_8
AP 1	JUN	path:04512_3
AP 1	JUN	path:04610_7
AP 1	JUN	path:05200_41
AP 1	JUN	path:05320_2
AP 1	JUN	path:04612_5
AP 1	JUN	path:04512_2
AP 1	JUN	path:05200_25
AP 1	JUN	path:05330_2
AP 1	JUN	path:04620_6
AP 1	JUNB	path:04350_2
AP 1	JUNB	path:04350_3
AP 1	JUND	path:05200_35
AP 1	JUND	path:05200_38
AP 1	JUND	path:05200_37
AP 1	JUND	path:05200_39
AP 1	JUND	path:05200_25
AP 1	JUND	path:05200_24
Developmental / cell cycle regulators	KLF4	path:04110_11
Developmental / cell cycle regulators	KLF4	path:04110_9
Developmental / cell cycle regulators	KLF4	path:05200_2
Developmental / cell cycle regulators	KLF4	path:05200_25
Developmental / cell cycle regulators	KLF4	path:04110_10
Developmental / cell cycle regulators	KLF4	path:05200_38
Developmental / cell cycle regulators	KLF4	path:05200_14
Developmental / cell cycle regulators	KLF4	path:05210_8
Developmental / cell cycle regulators	KLF4	path:05220_4
Developmental / cell cycle regulators	KLF4	path:05200_37
Developmental / cell cycle regulators	KLF4	path:05210_5
Developmental / cell cycle regulators	KLF4	path:05200_35
Developmental / cell cycle regulators	KLF4	path:05200_33
Developmental / cell cycle regulators	KLF4	path:04110_5
Developmental / cell cycle regulators	KLF4	path:05200_9
Developmental / cell cycle regulators	KLF4	path:05200_21
Developmental / cell cycle regulators	KLF4	path:05200_13
Developmental / cell cycle regulators	KLF4	path:05145_11
Developmental / cell cycle regulators	KLF4	path:04110_1
Developmental / cell cycle regulators	KLF4	path:05200_15
Developmental / cell cycle regulators	KLF4	path:05214_3
Developmental / cell cycle regulators	KLF4	path:04115_2
Developmental / cell cycle regulators	KLF4	path:05219_5
Developmental / cell cycle regulators	KLF4	path:04110_13

Developmental / cell cycle regulators	KLF4	path:05219_3
Developmental / cell cycle regulators	KLF4	path:05200_39
Developmental / cell cycle regulators	KLF5	path:05200_25
Developmental / cell cycle regulators	KLF5	path:05210_5
Developmental / cell cycle regulators	KLF5	path:05200_37
Developmental / cell cycle regulators	KLF5	path:05210_8
Developmental / cell cycle regulators	KLF5	path:05200_35
Developmental / cell cycle regulators	KLF5	path:05200_21
TCF 1	LEF1	path:05200_25
TCF 1	LEF1	path:05200_35
TCF 1	LEF1	path:05200_15
TCF 1	LEF1	path:05200_38
TCF 1	LEF1	path:05222_5
TCF 1	LEF1	path:05200_39
TCF 1	LEF1	path:05216_3
TCF 1	LEF1	path:05200_18
TCF 1	LEF1	path:04310_7
TCF 1	LEF1	path:05200_37
TCF 1	LEF1	path:05210_8
TCF 1	LEF1	path:05215_5
TCF 1	LEF1	path:05200_34
TCF 1	LEF1	path:04310_5
TCF 1	LEF1	path:05210_5
TCF 1	LEF1	path:05222_1
TCF 1	LEF1	path:05200_21
TCF 1	LEF1	path:04916_6
TCF 1	LEF1	path:04916_8
TCF 1	LEF1	path:05200_26
TCF 1	LEF1	path:04916_3
TCF 1	LEF1	path:05221_2
TCF 1	LEF1	path:05213_1
Ubiquitous bHLH ZIP factors	MITF	path:04916_3
Ubiquitous bHLH ZIP factors	MITF	path:04916_6
Ubiquitous bHLH ZIP factors	MITF	path:00350_1
Ubiquitous bHLH ZIP factors	MITF	path:04916_8
Cell cycle controlling factors	MYC	path:04110_2
Cell cycle controlling factors	MYC	path:05214_3
Cell cycle controlling factors	MYC	path:05218_3
Cell cycle controlling factors	MYC	path:04110_1
Cell cycle controlling factors	MYC	path:05222_1
Cell cycle controlling factors	MYC	path:05212_9
Cell cycle controlling factors	MYC	path:05220_4
Cell cycle controlling factors	MYC	path:04110_13
Cell cycle controlling factors	MYC	path:04110_5

**Cell cycle controlling factors**	**MYC**	**path:05200_21**
**Cell cycle controlling factors**	**MYC**	**path:05200_25**
**Cell cycle controlling factors**	**MYC**	**path:04110_9**
**Cell cycle controlling factors**	**MYC**	**path:05223_3**
**Cell cycle controlling factors**	**MYC**	**path:05210_5**
**Cell cycle controlling factors**	**MYC**	**path:05200_2**
**Cell cycle controlling factors**	**MYC**	**path:05219_5**
**Cell cycle controlling factors**	**MYC**	**path:05210_8**
**Cell cycle controlling factors**	**MYC**	**path:04510_13**
**Cell cycle controlling factors**	**MYC**	**path:05219_3**
**Cell cycle controlling factors**	**MYC**	**path:05200_15**
**Cell cycle controlling factors**	**MYC**	**path:05222_5**
**Cell cycle controlling factors**	**MYC**	**path:05200_34**
**Cell cycle controlling factors**	**MYC**	**path:04110_10**
**Cell cycle controlling factors**	**MYC**	**path:05213_1**
**Cell cycle controlling factors**	**MYC**	**path:04110_7**
**Cell cycle controlling factors**	**MYC**	**path:05200_24**
**Cell cycle controlling factors**	**MYC**	**path:05200_38**
**Cell cycle controlling factors**	**MYC**	**path:05218_5**
**Cell cycle controlling factors**	**MYC**	**path:05200_35**
**Cell cycle controlling factors**	**MYC**	**path:05221_2**
**Cell cycle controlling factors**	**MYC**	**path:04110_11**
**Cell cycle controlling factors**	**MYC**	**path:05216_3**
**Cell cycle controlling factors**	**MYC**	**path:05200_39**
**Cell cycle controlling factors**	**MYC**	**path:05200_37**
**NF AT**	**NFATC1**	**path:04060_19**
**NF AT**	**NFATC1**	**path:04060_34**
**NF AT**	**NFATC1**	**path:04060_20**
**AP 1**	**NFE2L2**	**path:00480_1**
**NF 1**	**NFIC**	**path:04080_5**
**NF 1**	**NFIC**	**path:05219_3**
**NF 1**	**NFIC**	**path:05200_7**
**NF 1**	**NFIC**	**path:05200_6**
**NF 1**	**NFIC**	**path:04512_2**
**NF 1**	**NFIC**	**path:04115_2**
**NF 1**	**NFIC**	**path:00591_1**
**NF 1**	**NFIC**	**path:05200_25**
**NF 1**	**NFIC**	**path:04662_9**
**NF 1**	**NFIC**	**path:05200_13**
**NF 1**	**NFIC**	**path:05200_15**
**NF 1**	**NFIC**	**path:05200_18**
**NF 1**	**NFIC**	**path:04662_5**
**NF 1**	**NFIC**	**path:05200_37**
**NF 1**	**NFIC**	**path:05200_24**

**NF 1**	**NFIC**	**path:05220_4**
**NF 1**	**NFIC**	**path:04510_6**
**NF 1**	**NFIC**	**path:05218_2**
**NF 1**	**NFIC**	**path:05200_26**
**NF 1**	**NFIC**	**path:05200_39**
**NF 1**	**NFIC**	**path:00140_1**
**NF 1**	**NFIC**	**path:05218_3**
**NF 1**	**NFIC**	**path:05214_3**
**NF 1**	**NFIC**	**path:04722_15**
**NF 1**	**NFIC**	**path:04010_22**
**NF 1**	**NFIC**	**path:04660_10**
**NF 1**	**NFIC**	**path:05200_2**
**NF 1**	**NFIC**	**path:04660_9**
**NF 1**	**NFIC**	**path:05200_21**
**NF 1**	**NFIC**	**path:05218_1**
**Heteromeric CCAAT factors**	**NFYA**	**path:04115_2**
**Heteromeric CCAAT factors**	**NFYA**	**path:04110_11**
**Heteromeric CCAAT factors**	**NFYA**	**path:04110_10**
**Heteromeric CCAAT factors**	**NFYA**	**path:04110_1**
**Heteromeric CCAAT factors**	**NFYA**	**path:04110_4**
**Heteromeric CCAAT factors**	**NFYA**	**path:04110_9**
**Heteromeric CCAAT factors**	**NFYA**	**path:04110_6**
**Heteromeric CCAAT factors**	**NFYA**	**path:04110_7**
**Heteromeric CCAAT factors**	**NFYA**	**path:04110_5**
**Heteromeric CCAAT factors**	**NFYA**	**path:04110_2**
**Steroid hormone receptors (NR3)**	**NR1I2**	**path:00982_2**
**Steroid hormone receptors (NR3)**	**NR1I2**	**path:00980_1**
**Steroid hormone receptors (NR3)**	**NR1I2**	**path:00830_1**
**Steroid hormone receptors (NR3)**	**NR1I3**	**path:00830_1**
**Steroid hormone receptors (NR3)**	**NR1I3**	**path:00980_1**
**Steroid hormone receptors (NR3)**	**NR1I3**	**path:00982_2**
**Steroid hormone receptors (NR3)**	**NR2F1**	**path:03320_1**
**Steroid hormone receptors (NR3)**	**NR4A1**	**path:00140_1**
**Steroid hormone receptors (NR3)**	**NR5A1**	**path:04350_6**
**Steroid hormone receptors (NR3)**	**NR5A1**	**path:04060_2**
**Steroid hormone receptors (NR3)**	**NR5A1**	**path:00140_1**
**Steroid hormone receptors (NR3)**	**NR5A2**	**path:03320_1**
**Steroid hormone receptors (NR3)**	**NR5A2**	**path:00140_1**
**AP 1**	**NRL**	**path:04744_2**
**Paired plus homeo domain**	**PAX6**	**path:05100_6**
**Paired plus homeo domain**	**PAX6**	**path:04510_5**
**Paired plus homeo domain**	**PAX6**	**path:04510_14**
**Paired plus homeo domain**	**PAX6**	**path:05414_2**
**Paired plus homeo domain**	**PAX6**	**path:04510_13**

Paired plus homeo domain	PAX6	path:04670_2
Paired plus homeo domain	PAX6	path:05100_7
Paired plus homeo domain	PAX6	path:04510_1
Paired plus homeo domain	PAX6	path:04510_7
Paired plus homeo domain	PAX6	path:04510_4
Paired plus homeo domain	PAX6	path:04512_2
Paired plus homeo domain	PAX6	path:04510_3
Paired plus homeo domain	PAX6	path:04514_17
Paired plus homeo domain	PAX6	path:04670_9
Paired plus homeo domain	PAX6	path:04512_1
Paired plus homeo domain	PAX6	path:04510_12
Paired plus homeo domain	PAX6	path:04514_5
Paired plus homeo domain	PAX6	path:04670_4
Paired plus homeo domain	PAX6	path:04510_16
Paired plus homeo domain	PAX6	path:04510_6
Paired plus homeo domain	PAX6	path:04512_3
Paired plus homeo domain	PAX6	path:04810_16
POU domain factors	POU2F1	path:05200_18
POU domain factors	POU2F1	path:05200_38
POU domain factors	POU2F1	path:05200_33
POU domain factors	POU2F1	path:00590_2
POU domain factors	POU2F1	path:05200_24
POU domain factors	POU2F1	path:04110_13
POU domain factors	POU2F1	path:04110_1
POU domain factors	POU2F1	path:04620_4
POU domain factors	POU2F1	path:04664_1
POU domain factors	POU2F1	path:04110_5
POU domain factors	POU2F1	path:05216_3
POU domain factors	POU2F1	path:00591_1
POU domain factors	POU2F1	path:04110_10
POU domain factors	POU2F1	path:05200_26
POU domain factors	POU2F1	path:05200_25
POU domain factors	POU2F1	path:04110_9
POU domain factors	POU2F1	path:05219_5
POU domain factors	POU2F1	path:00590_1
POU domain factors	POU2F1	path:05200_39
POU domain factors	POU2F1	path:00830_1
POU domain factors	POU2F1	path:05219_3
POU domain factors	POU2F1	path:00980_1
POU domain factors	POU2F1	path:04110_11
POU domain factors	POU2F1	path:05222_5
POU domain factors	POU2F1	path:05200_21
POU domain factors	POU2F1	path:05222_1
POU domain factors	POU2F1	path:04350_1

**POU domain factors**	**POU2F1**	**path:04060_4**
**POU domain factors**	**POU2F1**	**path:05200_15**
**POU domain factors**	**POU2F1**	**path:05200_35**
**POU domain factors**	**POU2F1**	**path:05200_20**
**POU domain factors**	**POU2F1**	**path:00982_2**
**POU domain factors**	**POU2F1**	**path:05200_37**
**POU domain factors**	**POU2F1**	**path:00232_1**
**Rel/ankyrin**	**REL**	**path:04650_14**
**Rel/ankyrin**	**REL**	**path:04650_13**
**Rel/ankyrin**	**REL**	**path:04650_15**
**Rel/ankyrin**	**REL**	**path:04620_1**
**Rel/ankyrin**	**REL**	**path:04612_5**
**Rel/ankyrin**	**REL**	**path:04650_5**
**Rel/ankyrin**	**REL**	**path:04620_4**
**Rel/ankyrin**	**REL**	**path:04620_3**
**Rel/ankyrin**	**RELA**	**path:04670_8**
**Rel/ankyrin**	**RELA**	**path:04060_35**
**Rel/ankyrin**	**RELA**	**path:05142_3**
**Rel/ankyrin**	**RELA**	**path:04650_15**
**Rel/ankyrin**	**RELA**	**path:04110_5**
**Rel/ankyrin**	**RELA**	**path:05219_3**
**Rel/ankyrin**	**RELA**	**path:04110_1**
**Rel/ankyrin**	**RELA**	**path:04060_44**
**Rel/ankyrin**	**RELA**	**path:04062_1**
**Rel/ankyrin**	**RELA**	**path:05200_35**
**Rel/ankyrin**	**RELA**	**path:05200_25**
**Rel/ankyrin**	**RELA**	**path:05200_15**
**Rel/ankyrin**	**RELA**	**path:04670_2**
**Rel/ankyrin**	**RELA**	**path:05200_39**
**Rel/ankyrin**	**RELA**	**path:05200_38**
**Rel/ankyrin**	**RELA**	**path:04612_5**
**Rel/ankyrin**	**RELA**	**path:04670_9**
**Rel/ankyrin**	**RELA**	**path:05200_37**
**Rel/ankyrin**	**RELA**	**path:04620_4**
**Rel/ankyrin**	**RELA**	**path:04110_9**
**Rel/ankyrin**	**RELA**	**path:04620_9**
**Rel/ankyrin**	**RELA**	**path:04110_10**
**Rel/ankyrin**	**RELA**	**path:04062_2**
**Rel/ankyrin**	**RELA**	**path:04110_7**
**Rel/ankyrin**	**RELA**	**path:04670_6**
**Rel/ankyrin**	**RELA**	**path:05200_2**
**Rel/ankyrin**	**RELA**	**path:04650_5**
**Rel/ankyrin**	**RELA**	**path:04110_11**
**Rel/ankyrin**	**RELA**	**path:04620_7**

Rel/ankyrin	RELA	path:04110_2
Rel/ankyrin	RELA	path:04620_5
Rel/ankyrin	RELA	path:05200_21
Rel/ankyrin	RELA	path:05200_13
Rel/ankyrin	RELA	path:04650_13
Rel/ankyrin	RELA	path:04620_8
Rel/ankyrin	RELA	path:04620_6
Rel/ankyrin	RELA	path:04650_14
Rel/ankyrin	RELA	path:04115_2
Rel/ankyrin	RELA	path:04620_1
Rel/ankyrin	RELA	path:04650_3
Rel/ankyrin	RELA	path:04620_3
Rel/ankyrin	RELA	path:04670_4
Regulators with a DNA recognition wing	RFX1	path:04514_19
Regulators with a DNA recognition wing	RFX1	path:04612_3
Regulators with a DNA recognition wing	RFX2	path:04514_19
Regulators with a DNA recognition wing	RFX2	path:04612_3
Regulators with a DNA recognition wing	RFX3	path:04514_19
Regulators with a DNA recognition wing	RFX3	path:04612_3
SMAD	SMAD2	path:04510_14
SMAD	SMAD2	path:04510_1
SMAD	SMAD2	path:04510_5
SMAD	SMAD2	path:04110_10
SMAD	SMAD2	path:05200_26
SMAD	SMAD2	path:04512_3
SMAD	SMAD2	path:04110_13
SMAD	SMAD2	path:04512_2
SMAD	SMAD2	path:04510_6
SMAD	SMAD2	path:05200_24
SMAD	SMAD2	path:04512_1
SMAD	SMAD2	path:05200_21
SMAD	SMAD2	path:04510_7
SMAD	SMAD2	path:04510_13
SMAD	SMAD2	path:05200_30
SMAD	SMAD3	path:04510_1
SMAD	SMAD3	path:04510_7
SMAD	SMAD3	path:04510_6
SMAD	SMAD3	path:05200_15
SMAD	SMAD3	path:05200_18
SMAD	SMAD3	path:04512_1
SMAD	SMAD3	path:05210_8
SMAD	SMAD3	path:04510_14
SMAD	SMAD3	path:05200_25
SMAD	SMAD3	path:04510_5

SMAD	SMAD3	path:04110_10
SMAD	SMAD3	path:04512_2
SMAD	SMAD3	path:05200_37
SMAD	SMAD3	path:05210_5
SMAD	SMAD3	path:04012_7
SMAD	SMAD3	path:04110_13
SMAD	SMAD3	path:05200_35
SMAD	SMAD3	path:05200_39
SMAD	SMAD3	path:05200_30
SMAD	SMAD3	path:04350_4
SMAD	SMAD3	path:04512_3
SMAD	SMAD3	path:05200_26
SMAD	SMAD3	path:05200_38
SMAD	SMAD3	path:05200_24
SMAD	SMAD4	path:04110_10
SMAD	SMAD4	path:04110_13
SMAD	SMAD4	path:05210_5
SMAD	SMAD4	path:05200_37
SMAD	SMAD4	path:05200_25
SMAD	SMAD4	path:04012_7
SMAD	SMAD4	path:04510_7
SMAD	SMAD4	path:05210_8
SMAD	SMAD4	path:04510_13
SMAD	SMAD4	path:05200_30
SMAD	SMAD4	path:05200_24
SMAD	SMAD4	path:05200_39
SMAD	SMAD4	path:05200_38
SMAD	SMAD4	path:04350_3
SMAD	SMAD4	path:04510_5
SMAD	SMAD4	path:05200_26
SMAD	SMAD4	path:05200_18
SMAD	SMAD4	path:05211_5
SMAD	SMAD4	path:05200_15
SMAD	SMAD4	path:04510_6
SMAD	SMAD4	path:04350_4
SMAD	SMAD4	path:05200_35
SMAD	SMAD4	path:05218_4
SMAD	SMAD4	path:04115_2
Ubiquitous factors	SP1	path:05212_9
Ubiquitous factors	SP1	path:04610_6
Ubiquitous factors	SP1	path:05200_30
Ubiquitous factors	SP1	path:04610_4
Ubiquitous factors	SP1	path:04540_7
Ubiquitous factors	SP1	path:05218_4

**Ubiquitous factors**	**SP1**	**path:05200_6**
**Ubiquitous factors**	**SP1**	**path:05200_33**
**Ubiquitous factors**	**SP1**	**path:04310_7**
**Ubiquitous factors**	**SP1**	**path:04512_1**
**Ubiquitous factors**	**SP1**	**path:04350_1**
**Ubiquitous factors**	**SP1**	**path:04210_9**
**Ubiquitous factors**	**SP1**	**path:00140_1**
**Ubiquitous factors**	**SP1**	**path:05219_5**
**Ubiquitous factors**	**SP1**	**path:05200_20**
**Ubiquitous factors**	**SP1**	**path:05145_8**
**Ubiquitous factors**	**SP1**	**path:05200_21**
**Ubiquitous factors**	**SP1**	**path:05200_8**
**Ubiquitous factors**	**SP1**	**path:05223_1**
**Ubiquitous factors**	**SP1**	**path:04510_5**
**Ubiquitous factors**	**SP1**	**path:05216_3**
**Ubiquitous factors**	**SP1**	**path:04620_9**
**Ubiquitous factors**	**SP1**	**path:05215_7**
**Ubiquitous factors**	**SP1**	**path:05200_34**
**Ubiquitous factors**	**SP1**	**path:04810_16**
**Ubiquitous factors**	**SP1**	**path:04110_10**
**Ubiquitous factors**	**SP1**	**path:04110_11**
**Ubiquitous factors**	**SP1**	**path:04620_6**
**Ubiquitous factors**	**SP1**	**path:04010_6**
**Ubiquitous factors**	**SP1**	**path:05200_29**
**Ubiquitous factors**	**SP1**	**path:05020_1**
**Ubiquitous factors**	**SP1**	**path:05222_2**
**Ubiquitous factors**	**SP1**	**path:04520_2**
**Ubiquitous factors**	**SP1**	**path:05200_26**
**Ubiquitous factors**	**SP1**	**path:05200_37**
**Ubiquitous factors**	**SP1**	**path:04610_2**
**Ubiquitous factors**	**SP1**	**path:04110_5**
**Ubiquitous factors**	**SP1**	**path:05215_10**
**Ubiquitous factors**	**SP1**	**path:05014_2**
**Ubiquitous factors**	**SP1**	**path:00330_5**
**Ubiquitous factors**	**SP1**	**path:05140_4**
**Ubiquitous factors**	**SP1**	**path:05332_1**
**Ubiquitous factors**	**SP1**	**path:05220_4**
**Ubiquitous factors**	**SP1**	**path:04210_5**
**Ubiquitous factors**	**SP1**	**path:04620_8**
**Ubiquitous factors**	**SP1**	**path:04110_1**
**Ubiquitous factors**	**SP1**	**path:05200_31**
**Ubiquitous factors**	**SP1**	**path:05215_4**
**Ubiquitous factors**	**SP1**	**path:05200_10**
**Ubiquitous factors**	**SP1**	**path:04610_5**

**Ubiquitous factors**	**SP1**	**path:05200_16**
**Ubiquitous factors**	**SP1**	**path:04510_17**
**Ubiquitous factors**	**SP1**	**path:04110_2**
**Ubiquitous factors**	**SP1**	**path:05215_5**
**Ubiquitous factors**	**SP1**	**path:05414_2**
**Ubiquitous factors**	**SP1**	**path:04630_1**
**Ubiquitous factors**	**SP1**	**path:05200_7**
**Ubiquitous factors**	**SP1**	**path:04610_1**
**Ubiquitous factors**	**SP1**	**path:05214_1**
**Ubiquitous factors**	**SP1**	**path:04520_5**
**Ubiquitous factors**	**SP1**	**path:04110_4**
**Ubiquitous factors**	**SP1**	**path:05220_5**
**Ubiquitous factors**	**SP1**	**path:05142_11**
**Ubiquitous factors**	**SP1**	**path:04510_3**
**Ubiquitous factors**	**SP1**	**path:00330_3**
**Ubiquitous factors**	**SP1**	**path:05320_2**
**Ubiquitous factors**	**SP1**	**path:05020_2**
**Ubiquitous factors**	**SP1**	**path:05330_2**
**Ubiquitous factors**	**SP1**	**path:05100_6**
**Ubiquitous factors**	**SP1**	**path:05145_6**
**Ubiquitous factors**	**SP1**	**path:05212_10**
**Ubiquitous factors**	**SP1**	**path:05221_2**
**Ubiquitous factors**	**SP1**	**path:04060_10**
**Ubiquitous factors**	**SP1**	**path:05214_2**
**Ubiquitous factors**	**SP1**	**path:04610_3**
**Ubiquitous factors**	**SP1**	**path:05200_24**
**Ubiquitous factors**	**SP1**	**path:04060_19**
**Ubiquitous factors**	**SP1**	**path:05200_14**
**Ubiquitous factors**	**SP1**	**path:05200_9**
**Ubiquitous factors**	**SP1**	**path:05210_8**
**Ubiquitous factors**	**SP1**	**path:04912_7**
**Ubiquitous factors**	**SP1**	**path:05222_5**
**Ubiquitous factors**	**SP1**	**path:04210_6**
**Ubiquitous factors**	**SP1**	**path:04660_9**
**Ubiquitous factors**	**SP1**	**path:04620_5**
**Ubiquitous factors**	**SP1**	**path:00330_4**
**Ubiquitous factors**	**SP1**	**path:05220_8**
**Ubiquitous factors**	**SP1**	**path:05200_41**
**Ubiquitous factors**	**SP1**	**path:04010_8**
**Ubiquitous factors**	**SP1**	**path:04350_5**
**Ubiquitous factors**	**SP1**	**path:05142_3**
**Ubiquitous factors**	**SP1**	**path:04110_9**
**Ubiquitous factors**	**SP1**	**path:05218_1**
**Ubiquitous factors**	**SP1**	**path:05215_6**

**Ubiquitous factors**	**SP1**	**path:04510_12**
**Ubiquitous factors**	**SP1**	**path:04510_19**
**Ubiquitous factors**	**SP1**	**path:05200_42**
**Ubiquitous factors**	**SP1**	**path:04610_7**
**Ubiquitous factors**	**SP1**	**path:05200_32**
**Ubiquitous factors**	**SP1**	**path:05200_17**
**Ubiquitous factors**	**SP1**	**path:04650_3**
**Ubiquitous factors**	**SP1**	**path:00330_1**
**Ubiquitous factors**	**SP1**	**path:05200_1**
**Ubiquitous factors**	**SP1**	**path:00980_1**
**Ubiquitous factors**	**SP1**	**path:04350_4**
**Ubiquitous factors**	**SP1**	**path:04060_32**
**Ubiquitous factors**	**SP1**	**path:05212_8**
**Ubiquitous factors**	**SP1**	**path:04060_46**
**Ubiquitous factors**	**SP1**	**path:05200_19**
**Ubiquitous factors**	**SP1**	**path:04110_13**
**Ubiquitous factors**	**SP1**	**path:05214_3**
**Ubiquitous factors**	**SP1**	**path:04510_6**
**Ubiquitous factors**	**SP1**	**path:04510_4**
**Ubiquitous factors**	**SP1**	**path:04510_16**
**Ubiquitous factors**	**SP1**	**path:04510_1**
**Ubiquitous factors**	**SP1**	**path:05200_25**
**Ubiquitous factors**	**SP1**	**path:05222_1**
**Ubiquitous factors**	**SP1**	**path:05215_9**
**Ubiquitous factors**	**SP1**	**path:04115_2**
**Ubiquitous factors**	**SP1**	**path:05200_39**
**Ubiquitous factors**	**SP1**	**path:05014_1**
**Ubiquitous factors**	**SP1**	**path:04512_3**
**Ubiquitous factors**	**SP1**	**path:05219_3**
**Ubiquitous factors**	**SP1**	**path:04512_2**
**Ubiquitous factors**	**SP1**	**path:00350_2**
**Ubiquitous factors**	**SP1**	**path:05200_2**
**Ubiquitous factors**	**SP1**	**path:04510_14**
**Ubiquitous factors**	**SP1**	**path:05218_2**
**Ubiquitous factors**	**SP1**	**path:05212_3**
**Ubiquitous factors**	**SP1**	**path:05200_13**
**Ubiquitous factors**	**SP1**	**path:04722_15**
**Ubiquitous factors**	**SP1**	**path:04210_4**
**Ubiquitous factors**	**SP1**	**path:05223_3**
**Ubiquitous factors**	**SP1**	**path:05218_3**
**Ubiquitous factors**	**SP1**	**path:05146_1**
**Ubiquitous factors**	**SP1**	**path:05200_35**
**Ubiquitous factors**	**SP1**	**path:04010_7**
**Ubiquitous factors**	**SP1**	**path:04662_9**

**Ubiquitous factors**	**SP1**	**path:04630_2**
**Ubiquitous factors**	**SP1**	**path:05140_6**
**Ubiquitous factors**	**SP1**	**path:05200_38**
**Ubiquitous factors**	**SP1**	**path:04144_2**
**Ubiquitous factors**	**SP1**	**path:04940_2**
**Ubiquitous factors**	**SP1**	**path:04510_13**
**Ubiquitous factors**	**SP1**	**path:05210_5**
**Ubiquitous factors**	**SP1**	**path:04110_7**
**Ubiquitous factors**	**SP1**	**path:05142_12**
**Ubiquitous factors**	**SP1**	**path:05200_18**
**Ubiquitous factors**	**SP1**	**path:04510_7**
**Ubiquitous factors**	**SP1**	**path:05218_5**
**Ubiquitous factors**	**SP1**	**path:04060_20**
**Ubiquitous factors**	**SP1**	**path:00590_2**
**Ubiquitous factors**	**SP1**	**path:05200_15**
**Ubiquitous factors**	**SP1**	**path:04310_5**
**Ubiquitous factors**	**SP1**	**path:04510_10**
**Ubiquitous factors**	**SP3**	**path:05144_2**
**Ubiquitous factors**	**SP3**	**path:04110_2**
**Ubiquitous factors**	**SP3**	**path:00120_1**
**Ubiquitous factors**	**SP3**	**path:04110_1**
**Ubiquitous factors**	**SP3**	**path:05200_37**
**Ubiquitous factors**	**SP3**	**path:04610_7**
**Ubiquitous factors**	**SP3**	**path:04110_13**
**Ubiquitous factors**	**SP3**	**path:04510_13**
**Ubiquitous factors**	**SP3**	**path:04510_5**
**Ubiquitous factors**	**SP3**	**path:05222_1**
**Ubiquitous factors**	**SP3**	**path:05200_21**
**Ubiquitous factors**	**SP3**	**path:05200_14**
**Ubiquitous factors**	**SP3**	**path:04110_5**
**Ubiquitous factors**	**SP3**	**path:04144_2**
**Ubiquitous factors**	**SP3**	**path:05200_38**
**Ubiquitous factors**	**SP3**	**path:00600_1**
**Ubiquitous factors**	**SP3**	**path:05200_18**
**Ubiquitous factors**	**SP3**	**path:05200_39**
**Ubiquitous factors**	**SP3**	**path:04510_7**
**Ubiquitous factors**	**SP3**	**path:00140_1**
**Ubiquitous factors**	**SP3**	**path:04110_7**
**Ubiquitous factors**	**SP3**	**path:04510_6**
**Ubiquitous factors**	**SP3**	**path:05200_20**
**Ubiquitous factors**	**SP3**	**path:05218_5**
**Ubiquitous factors**	**SP3**	**path:05200_9**
**Ubiquitous factors**	**SP3**	**path:05218_2**
**Ubiquitous factors**	**SP3**	**path:05200_15**

**Ubiquitous factors**	**SP3**	**path:05200_33**
**Ubiquitous factors**	**SP3**	**path:04510_4**
**Ubiquitous factors**	**SP3**	**path:04510_1**
**Ubiquitous factors**	**SP3**	**path:04510_14**
**Ubiquitous factors**	**SP3**	**path:04810_16**
**Ubiquitous factors**	**SP3**	**path:04510_12**
**Ubiquitous factors**	**SP3**	**path:05200_24**
**Ubiquitous factors**	**SP3**	**path:05220_8**
**Ubiquitous factors**	**SP3**	**path:04060_28**
**Ubiquitous factors**	**SP3**	**path:04110_4**
**Ubiquitous factors**	**SP3**	**path:04060_20**
**Ubiquitous factors**	**SP3**	**path:05218_1**
**Ubiquitous factors**	**SP3**	**path:05220_4**
**Ubiquitous factors**	**SP3**	**path:05200_16**
**Ubiquitous factors**	**SP3**	**path:05219_3**
**Ubiquitous factors**	**SP3**	**path:05200_35**
**Ubiquitous factors**	**SP3**	**path:00120_2**
**Ubiquitous factors**	**SP3**	**path:05223_3**
**Ubiquitous factors**	**SP3**	**path:04512_1**
**Ubiquitous factors**	**SP3**	**path:04512_2**
**Ubiquitous factors**	**SP3**	**path:05200_2**
**Ubiquitous factors**	**SP3**	**path:04610_2**
**Ubiquitous factors**	**SP3**	**path:04110_10**
**Ubiquitous factors**	**SP3**	**path:04110_11**
**Ubiquitous factors**	**SP3**	**path:04115_2**
**Ubiquitous factors**	**SP3**	**path:04512_3**
**Ubiquitous factors**	**SP3**	**path:05219_5**
**Ubiquitous factors**	**SP3**	**path:05212_9**
**Ubiquitous factors**	**SP3**	**path:05414_2**
**Ubiquitous factors**	**SP3**	**path:05218_3**
**Ubiquitous factors**	**SP3**	**path:05214_3**
**Ubiquitous factors**	**SP3**	**path:05200_13**
**Ubiquitous factors**	**SP3**	**path:04610_6**
**Ubiquitous factors**	**SP3**	**path:05200_25**
**Ubiquitous factors**	**SP3**	**path:04110_9**
**Ubiquitous factors**	**SP3**	**path:05200_26**
**Ets type**	**SPI1**	**path:04670_6**
**Ets type**	**SPI1**	**path:05140_2**
**Ets type**	**SPI1**	**path:04060_26**
**Ets type**	**SPI1**	**path:05150_4**
**Ets type**	**SPI1**	**path:04670_4**
**Ets type**	**SPI1**	**path:04670_2**
**Ets type**	**SPI1**	**path:04620_4**
**Ets type**	**SPI1**	**path:04620_1**

Ets type	SPI1	path:04145_1
Ets type	SPI1	path:05140_3
Ets type	SPI1	path:04620_3
Ets type	SPI1	path:05200_34
Ets type	SPI1	path:04670_9
Ets type	SPI1	path:04145_4
Ets type	SPI1	path:04670_8
Ets type	SPI1	path:04650_1
Ets type	SPI1	path:04650_7
Ets type	SPI1	path:05146_1
Ets type	SPI1	path:04810_16
Ubiquitous bHLH ZIP factors	SREBF1	path:04910_1
Ubiquitous bHLH ZIP factors	SREBF1	path:04910_4
Responders to external signals	SRF	path:05020_3
Responders to external signals	SRF	path:05020_4
Responders to external signals	SRF	path:05416_1
Responders to external signals	SRF	path:05410_1
STAT	STAT1	path:04145_4
STAT	STAT1	path:04670_2
STAT	STAT1	path:04620_4
STAT	STAT1	path:04670_9
STAT	STAT1	path:05213_1
STAT	STAT1	path:05219_5
STAT	STAT1	path:04110_10
STAT	STAT1	path:05200_18
STAT	STAT1	path:04620_6
STAT	STAT1	path:04620_9
STAT	STAT1	path:05145_8
STAT	STAT1	path:04670_4
STAT	STAT1	path:04670_6
STAT	STAT1	path:05210_5
STAT	STAT1	path:05200_39
STAT	STAT1	path:05219_3
STAT	STAT1	path:05200_26
STAT	STAT1	path:04620_5
STAT	STAT1	path:05200_35
STAT	STAT1	path:05216_3
STAT	STAT1	path:04620_1
STAT	STAT1	path:05210_8
STAT	STAT1	path:04110_9
STAT	STAT1	path:05220_4
STAT	STAT1	path:05214_3
STAT	STAT1	path:05200_38
STAT	STAT1	path:05200_25

**STAT**	**STAT1**	**path:04630_1**
**STAT**	**STAT1**	**path:04110_11**
**STAT**	**STAT1**	**path:05200_15**
**STAT**	**STAT1**	**path:04110_13**
**STAT**	**STAT1**	**path:04670_8**
**STAT**	**STAT1**	**path:05145_12**
**STAT**	**STAT1**	**path:04630_2**
**STAT**	**STAT1**	**path:04620_8**
**STAT**	**STAT1**	**path:05222_5**
**STAT**	**STAT1**	**path:05200_24**
**STAT**	**STAT1**	**path:05200_21**
**STAT**	**STAT1**	**path:05200_37**
**STAT**	**STAT3**	**path:04630_2**
**STAT**	**STAT3**	**path:05210_5**
**STAT**	**STAT3**	**path:05200_39**
**STAT**	**STAT3**	**path:05200_35**
**STAT**	**STAT3**	**path:05200_38**
**STAT**	**STAT3**	**path:05200_21**
**STAT**	**STAT3**	**path:04012_4**
**STAT**	**STAT3**	**path:05200_30**
**STAT**	**STAT3**	**path:05142_3**
**STAT**	**STAT3**	**path:05210_8**
**STAT**	**STAT3**	**path:05200_15**
**STAT**	**STAT3**	**path:05200_26**
**STAT**	**STAT3**	**path:04060_1**
**STAT**	**STAT3**	**path:05200_25**
**STAT**	**STAT3**	**path:05200_18**
**STAT**	**STAT3**	**path:04630_1**
**STAT**	**STAT3**	**path:05200_24**
**STAT**	**STAT3**	**path:04060_34**
**STAT**	**STAT3**	**path:05221_1**
**STAT**	**STAT3**	**path:05200_37**
**STAT**	**STAT3**	**path:05200_16**
**STAT**	**STAT3**	**path:05218_4**
**STAT**	**STAT4**	**path:04630_2**
**STAT**	**STAT5A**	**path:04630_2**
**STAT**	**STAT5A**	**path:04630_1**
**STAT**	**STAT5B**	**path:05200_25**
**STAT**	**STAT5B**	**path:05200_38**
**STAT**	**STAT5B**	**path:05200_26**
**STAT**	**STAT5B**	**path:05200_35**
**STAT**	**STAT5B**	**path:05218_3**
**STAT**	**STAT5B**	**path:05219_3**
**STAT**	**STAT5B**	**path:05219_5**

**STAT**	**STAT5B**	**path:05218_1**
**STAT**	**STAT5B**	**path:05200_24**
**STAT**	**STAT5B**	**path:04630_2**
**STAT**	**STAT5B**	**path:05200_39**
**STAT**	**STAT5B**	**path:05214_3**
**STAT**	**STAT5B**	**path:04630_1**
**STAT**	**STAT5B**	**path:05200_33**
**STAT**	**STAT5B**	**path:05220_4**
**STAT**	**STAT5B**	**path:05200_37**
**STAT**	**STAT5B**	**path:05200_21**
**STAT**	**STAT5B**	**path:05218_5**
**STAT**	**STAT5B**	**path:05218_2**
**STAT**	**STAT6**	**path:00590_1**
**STAT**	**STAT6**	**path:00590_2**
**STAT**	**STAT6**	**path:04630_2**
**TCF 1**	**TCF7**	**path:04660_15**
**AP 2**	**TFAP2A**	**path:05215_7**
**AP 2**	**TFAP2A**	**path:05200_37**
**AP 2**	**TFAP2A**	**path:05200_8**
**AP 2**	**TFAP2A**	**path:05215_9**
**AP 2**	**TFAP2A**	**path:04520_5**
**AP 2**	**TFAP2A**	**path:04520_7**
**AP 2**	**TFAP2A**	**path:04012_5**
**AP 2**	**TFAP2A**	**path:05200_9**
**AP 2**	**TFAP2A**	**path:04520_3**
**AP 2**	**TFAP2A**	**path:05214_2**
**AP 2**	**TFAP2A**	**path:05215_10**
**AP 2**	**TFAP2A**	**path:05219_2**
**AP 2**	**TFAP2A**	**path:05212_8**
**AP 2**	**TFAP2A**	**path:04510_6**
**AP 2**	**TFAP2A**	**path:05200_35**
**AP 2**	**TFAP2A**	**path:05200_7**
**AP 2**	**TFAP2A**	**path:04080_5**
**AP 2**	**TFAP2A**	**path:05215_8**
**AP 2**	**TFAP2A**	**path:05200_19**
**AP 2**	**TFAP2A**	**path:05200_39**
**AP 2**	**TFAP2A**	**path:05214_1**
**AP 2**	**TFAP2A**	**path:05200_17**
**AP 2**	**TFAP2A**	**path:04510_19**
**AP 2**	**TFAP2A**	**path:05212_3**
**AP 2**	**TFAP2A**	**path:05200_16**
**AP 2**	**TFAP2A**	**path:04144_2**
**AP 2**	**TFAP2A**	**path:04020_1**
**AP 2**	**TFAP2A**	**path:04510_4**

**AP 2**	**TFAP2A**	**path:04912_7**
**AP 2**	**TFAP2A**	**path:05223_1**
**AP 2**	**TFAP2A**	**path:04510_5**
**AP 2**	**TFAP2A**	**path:04010_7**
**AP 2**	**TFAP2A**	**path:05200_25**
**AP 2**	**TFAP2A**	**path:05200_6**
**AP 2**	**TFAP2A**	**path:05200_24**
**AP 2**	**TFAP2A**	**path:04510_7**
**AP 2**	**TFAP2A**	**path:04520_2**
**AP 2**	**TFAP2A**	**path:04510_13**
**AP 2**	**TFAP2A**	**path:05200_29**
**AP 2**	**TFAP2A**	**path:05200_38**
**AP 2**	**TFAP2A**	**path:05200_10**
**AP 2**	**TFAP2A**	**path:04510_12**
**AP 2**	**TFAP2A**	**path:05200_30**
**AP 2**	**TFAP2A**	**path:05200_26**
**AP 2**	**TFAP2A**	**path:05200_2**
**AP 2**	**TFAP2C**	**path:05219_2**
**AP 2**	**TFAP2C**	**path:04912_7**
**Steroid hormone receptors (NR3)**	**THRB**	**path:04080_2**
**p53 like**	**TP53**	**path:05219_3**
**p53 like**	**TP53**	**path:05220_4**
**p53 like**	**TP53**	**path:05200_26**
**p53 like**	**TP53**	**path:05215_10**
**p53 like**	**TP53**	**path:05200_2**
**p53 like**	**TP53**	**path:04110_7**
**p53 like**	**TP53**	**path:05200_8**
**p53 like**	**TP53**	**path:04110_1**
**p53 like**	**TP53**	**path:05200_25**
**p53 like**	**TP53**	**path:05200_1**
**p53 like**	**TP53**	**path:05200_14**
**p53 like**	**TP53**	**path:05200_17**
**p53 like**	**TP53**	**path:04912_7**
**p53 like**	**TP53**	**path:05218_3**
**p53 like**	**TP53**	**path:05218_2**
**p53 like**	**TP53**	**path:05200_38**
**p53 like**	**TP53**	**path:05215_4**
**p53 like**	**TP53**	**path:04115_2**
**p53 like**	**TP53**	**path:05215_6**
**p53 like**	**TP53**	**path:05223_2**
**p53 like**	**TP53**	**path:05214_3**
**p53 like**	**TP53**	**path:05200_37**
**p53 like**	**TP53**	**path:05215_7**
**p53 like**	**TP53**	**path:05200_10**

**p53 like**	**TP53**	**path:05200_19**
**p53 like**	**TP53**	**path:05160_10**
**p53 like**	**TP53**	**path:05014_2**
**p53 like**	**TP53**	**path:05200_13**
**p53 like**	**TP53**	**path:04360_3**
**p53 like**	**TP53**	**path:04210_9**
**p53 like**	**TP53**	**path:05214_2**
**p53 like**	**TP53**	**path:05200_39**
**p53 like**	**TP53**	**path:04110_11**
**p53 like**	**TP53**	**path:05160_9**
**p53 like**	**TP53**	**path:05200_7**
**p53 like**	**TP53**	**path:04110_5**
**p53 like**	**TP53**	**path:05200_3**
**p53 like**	**TP53**	**path:04210_8**
**p53 like**	**TP53**	**path:04510_12**
**p53 like**	**TP53**	**path:05200_24**
**p53 like**	**TP53**	**path:04110_9**
**p53 like**	**TP53**	**path:05200_16**
**p53 like**	**TP53**	**path:04110_6**
**p53 like**	**TP53**	**path:04110_13**
**p53 like**	**TP53**	**path:05200_21**
**p53 like**	**TP53**	**path:04722_15**
**p53 like**	**TP53**	**path:04650_3**
**p53 like**	**TP53**	**path:05200_20**
**p53 like**	**TP53**	**path:04510_13**
**p53 like**	**TP53**	**path:05200_15**
**p53 like**	**TP53**	**path:05200_9**
**p53 like**	**TP53**	**path:05219_5**
**p53 like**	**TP53**	**path:04510_6**
**p53 like**	**TP53**	**path:04012_1**
**p53 like**	**TP53**	**path:05218_5**
**p53 like**	**TP53**	**path:04510_19**
**p53 like**	**TP53**	**path:05213_3**
**p53 like**	**TP53**	**path:04510_4**
**p53 like**	**TP53**	**path:05200_6**
**p53 like**	**TP53**	**path:04110_10**
**p53 like**	**TP53**	**path:04010_11**
**p53 like**	**TP53**	**path:05200_18**
**p53 like**	**TP53**	**path:04110_2**
**p53 like**	**TP53**	**path:04012_5**
**p53 like**	**TP53**	**path:05218_1**
**p53 like**	**TP53**	**path:05215_9**
**p53 like**	**TP53**	**path:05214_1**
**p53 like**	**TP63**	**path:05220_4**

**p53 like**	**TP63**	**path:05200_14**
**p53 like**	**TP63**	**path:05200_7**
**p53 like**	**TP63**	**path:05200_37**
**p53 like**	**TP63**	**path:04110_7**
**p53 like**	**TP63**	**path:05200_20**
**p53 like**	**TP63**	**path:05200_15**
**p53 like**	**TP63**	**path:05200_39**
**p53 like**	**TP63**	**path:04110_1**
**p53 like**	**TP63**	**path:05218_3**
**p53 like**	**TP63**	**path:05200_9**
**p53 like**	**TP63**	**path:05214_3**
**p53 like**	**TP63**	**path:05219_3**
**p53 like**	**TP63**	**path:05200_13**
**p53 like**	**TP63**	**path:05215_4**
**p53 like**	**TP63**	**path:05215_10**
**p53 like**	**TP63**	**path:05215_9**
**p53 like**	**TP63**	**path:04115_2**
**p53 like**	**TP63**	**path:05218_5**
**p53 like**	**TP63**	**path:04110_9**
**p53 like**	**TP63**	**path:05200_19**
**p53 like**	**TP63**	**path:04110_5**
**p53 like**	**TP63**	**path:05200_1**
**p53 like**	**TP63**	**path:05200_21**
**p53 like**	**TP63**	**path:05200_3**
**p53 like**	**TP63**	**path:05219_5**
**p53 like**	**TP63**	**path:05200_2**
**p53 like**	**TP63**	**path:04110_11**
**p53 like**	**TP63**	**path:04110_2**
**p53 like**	**TP63**	**path:04110_10**
**p53 like**	**TP63**	**path:05200_25**
**p53 like**	**TP63**	**path:05215_6**
**p53 like**	**TP73**	**path:04722_15**
**p53 like**	**TP73**	**path:05200_33**
**p53 like**	**TP73**	**path:04115_2**
**Steroid hormone receptors (NR3)**	**VDR**	**path:05200_10**
**Steroid hormone receptors (NR3)**	**VDR**	**path:05200_16**
**Steroid hormone receptors (NR3)**	**VDR**	**path:05200_7**
**Steroid hormone receptors (NR3)**	**VDR**	**path:05200_19**
**AP 1**	**XBP1**	**path:04514_19**
**AP 1**	**XBP1**	**path:04612_3**
**csd**	**YBX1**	**path:04910_13**
**csd**	**YBX1**	**path:05212_4**
**csd**	**YBX1**	**path:04510_12**
**csd**	**YBX1**	**path:04910_8**

csd	YBX1	path:05212_1
csd	YBX1	path:05223_2
csd	YBX1	path:05211_1
csd	YBX1	path:04510_19
csd	YBX1	path:05215_7
csd	YBX1	path:04510_6
csd	YBX1	path:04520_3
csd	YBX1	path:05212_6
csd	YBX1	path:05212_2
csd	YBX1	path:04510_7
csd	YBX1	path:04520_6
csd	YBX1	path:05200_24
csd	YBX1	path:04144_2
csd	YBX1	path:05200_10
csd	YBX1	path:04520_7
csd	YBX1	path:04510_4
csd	YBX1	path:05211_5
csd	YBX1	path:05218_4
csd	YBX1	path:04510_5
csd	YBX1	path:05212_3
csd	YBX1	path:05211_4
csd	YBX1	path:05215_9
csd	YBX1	path:04520_5
csd	YBX1	path:05100_10
csd	YBX1	path:05200_8
csd	YBX1	path:04510_13
csd	YBX1	path:05218_1
csd	YBX1	path:05200_16
csd	YBX1	path:05212_8
csd	YBX1	path:05223_1
csd	YBX1	path:05218_2
csd	YBX1	path:05100_11
csd	YBX1	path:05213_2
csd	YBX1	path:05200_29
csd	YBX1	path:04910_2
csd	YBX1	path:04520_2
Ubiquitous factors	YY1	path:04110_2
Ubiquitous factors	YY1	path:04110_13
Ubiquitous factors	YY1	path:04110_11
Ubiquitous factors	YY1	path:04115_2
Ubiquitous factors	YY1	path:04110_10
Ubiquitous factors	YY1	path:04650_3
Ubiquitous factors	YY1	path:05200_39
Ubiquitous factors	YY1	path:05200_35

**Ubiquitous factors**	**YY1**	**path:05218_3**
**Ubiquitous factors**	**YY1**	**path:05200_37**
**Ubiquitous factors**	**YY1**	**path:05218_5**
**Ubiquitous factors**	**YY1**	**path:05200_18**
**Ubiquitous factors**	**YY1**	**path:05220_4**
**Ubiquitous factors**	**YY1**	**path:05200_13**
**Ubiquitous factors**	**YY1**	**path:05219_5**
**Ubiquitous factors**	**YY1**	**path:05214_3**
**Ubiquitous factors**	**YY1**	**path:04110_5**
**Ubiquitous factors**	**YY1**	**path:05212_9**
**Ubiquitous factors**	**YY1**	**path:04110_9**
**Ubiquitous factors**	**YY1**	**path:05219_3**
**Ubiquitous factors**	**YY1**	**path:04650_5**
**Ubiquitous factors**	**YY1**	**path:05200_26**
**Ubiquitous factors**	**YY1**	**path:05200_21**
**Ubiquitous factors**	**YY1**	**path:05200_25**
**Ubiquitous factors**	**YY1**	**path:05200_38**
**Ubiquitous factors**	**YY1**	**path:04110_1**
**Ubiquitous factors**	**YY1**	**path:05200_15**

Table S9. The detailed information of the co-tissue TFs regulated sub-pathways of TSN with k=4.

Tissues	TF	Subpathway
adipocytes	CEBPA	path:04060_37
adipocytes	CEBPA	path:04610_1
adipocytes	CEBPA	path:04610_4
adipocytes	CEBPA	path:04610_2
adipocytes	CEBPA	path:04610_5
adipocytes	CEBPA	path:04610_3
adrenal	SREBF1	path:04910_1
adrenal	SREBF1	path:04910_4
B cell	BCL6	path:04110_13
B cell	BCL6	path:04110_7
B cell	BCL6	path:04110_11
B cell	BCL6	path:04110_5
B cell	BCL6	path:04110_1
B cell	BCL6	path:04110_9
B cell	BCL6	path:04110_10
B cell	BCL6	path:04110_2
B cell	BCL6	path:04115_2
B cell	BCL6	path:04210_3
B cell	BCL6	path:04210_6
B cell	BCL6	path:04630_1
B cell	BCL6	path:05200_13
B cell	BCL6	path:05200_25
B cell	BCL6	path:05200_2
B cell	BCL6	path:05200_39
B cell	BCL6	path:05200_15
B cell	BCL6	path:05200_37
B cell	BCL6	path:05200_21
B cell	BCL6	path:05220_8
B cell	SPI1	path:04060_26
B cell	SPI1	path:04145_4
B cell	SPI1	path:04145_1
B cell	SPI1	path:04620_3
B cell	SPI1	path:04620_4
B cell	SPI1	path:04620_1
B cell	SPI1	path:04650_7
B cell	SPI1	path:04650_1
B cell	SPI1	path:04670_6
B cell	SPI1	path:04670_2
B cell	SPI1	path:04670_4
B cell	SPI1	path:04670_8
B cell	SPI1	path:04670_9

B cell	SPI1	path:04810_16
B cell	SPI1	path:05140_3
B cell	SPI1	path:05140_2
B cell	SPI1	path:05146_1
B cell	SPI1	path:05150_4
B cell	SPI1	path:05200_34
BL	ELK1	path:04010_11
BL	ELK1	path:04010_4
BL	ELK1	path:05020_4
BL	ELK1	path:05020_3
BL	ELK1	path:05110_3
BL	ELK1	path:05200_18
BL	ELK1	path:05200_26
BL	FLI1	path:04512_3
BL	FLI1	path:04512_1
BL	TFAP2A	path:04010_7
BL	TFAP2A	path:04012_5
BL	TFAP2A	path:04020_1
BL	TFAP2A	path:04080_5
BL	TFAP2A	path:04144_2
BL	TFAP2A	path:04510_5
BL	TFAP2A	path:04510_4
BL	TFAP2A	path:04510_19
BL	TFAP2A	path:04510_13
BL	TFAP2A	path:04510_12
BL	TFAP2A	path:04510_7
BL	TFAP2A	path:04510_6
BL	TFAP2A	path:04520_2
BL	TFAP2A	path:04520_7
BL	TFAP2A	path:04520_5
BL	TFAP2A	path:04520_3
BL	TFAP2A	path:04912_7
BL	TFAP2A	path:05200_24
BL	TFAP2A	path:05200_19
BL	TFAP2A	path:05200_6
BL	TFAP2A	path:05200_7
BL	TFAP2A	path:05200_38
BL	TFAP2A	path:05200_35
BL	TFAP2A	path:05200_26
BL	TFAP2A	path:05200_39
BL	TFAP2A	path:05200_29
BL	TFAP2A	path:05200_25
BL	TFAP2A	path:05200_8
BL	TFAP2A	path:05200_37

**BL**	**TFAP2A**	**path:05200_17**
**BL**	**TFAP2A**	**path:05200_2**
**BL**	**TFAP2A**	**path:05200_30**
**BL**	**TFAP2A**	**path:05200_9**
**BL**	**TFAP2A**	**path:05200_10**
**BL**	**TFAP2A**	**path:05200_16**
**BL**	**TFAP2A**	**path:05212_3**
**BL**	**TFAP2A**	**path:05212_8**
**BL**	**TFAP2A**	**path:05214_1**
**BL**	**TFAP2A**	**path:05214_2**
**BL**	**TFAP2A**	**path:05215_8**
**BL**	**TFAP2A**	**path:05215_7**
**BL**	**TFAP2A**	**path:05215_9**
**BL**	**TFAP2A**	**path:05215_10**
**BL**	**TFAP2A**	**path:05219_2**
**BL**	**TFAP2A**	**path:05223_1**
**BL**	**TP53**	**path:04010_11**
**BL**	**TP53**	**path:04012_5**
**BL**	**TP53**	**path:04012_1**
**BL**	**TP53**	**path:04110_10**
**BL**	**TP53**	**path:04110_13**
**BL**	**TP53**	**path:04110_5**
**BL**	**TP53**	**path:04110_11**
**BL**	**TP53**	**path:04110_2**
**BL**	**TP53**	**path:04110_6**
**BL**	**TP53**	**path:04110_7**
**BL**	**TP53**	**path:04110_1**
**BL**	**TP53**	**path:04110_9**
**BL**	**TP53**	**path:04115_2**
**BL**	**TP53**	**path:04210_9**
**BL**	**TP53**	**path:04210_8**
**BL**	**TP53**	**path:04360_3**
**BL**	**TP53**	**path:04510_19**
**BL**	**TP53**	**path:04510_13**
**BL**	**TP53**	**path:04510_12**
**BL**	**TP53**	**path:04510_4**
**BL**	**TP53**	**path:04510_6**
**BL**	**TP53**	**path:04650_3**
**BL**	**TP53**	**path:04722_15**
**BL**	**TP53**	**path:04912_7**
**BL**	**TP53**	**path:05014_2**
**BL**	**TP53**	**path:05160_10**
**BL**	**TP53**	**path:05160_9**
**BL**	**TP53**	**path:05200_7**

BL	TP53	path:05200_15
BL	TP53	path:05200_1
BL	TP53	path:05200_2
BL	TP53	path:05200_18
BL	TP53	path:05200_24
BL	TP53	path:05200_38
BL	TP53	path:05200_19
BL	TP53	path:05200_37
BL	TP53	path:05200_14
BL	TP53	path:05200_26
BL	TP53	path:05200_25
BL	TP53	path:05200_6
BL	TP53	path:05200_10
BL	TP53	path:05200_39
BL	TP53	path:05200_16
BL	TP53	path:05200_17
BL	TP53	path:05200_8
BL	TP53	path:05200_9
BL	TP53	path:05200_20
BL	TP53	path:05200_13
BL	TP53	path:05200_21
BL	TP53	path:05200_3
BL	TP53	path:05213_3
BL	TP53	path:05214_1
BL	TP53	path:05214_2
BL	TP53	path:05214_3
BL	TP53	path:05215_6
BL	TP53	path:05215_4
BL	TP53	path:05215_7
BL	TP53	path:05215_9
BL	TP53	path:05215_10
BL	TP53	path:05218_2
BL	TP53	path:05218_3
BL	TP53	path:05218_1
BL	TP53	path:05218_5
BL	TP53	path:05219_3
BL	TP53	path:05219_5
BL	TP53	path:05220_4
BL	TP53	path:05223_2
bone marrow	AKNA	path:04060_18
bone marrow	AKNA	path:04514_8
bone marrow	AKNA	path:04672_2
bone marrow	AKNA	path:05144_4
bone marrow	AKNA	path:05145_14

bone marrow	AKNA	path:05310_1
bone marrow	AKNA	path:05320_1
bone marrow	AKNA	path:05322_3
bone marrow	AKNA	path:05330_1
bone marrow	AKNA	path:05416_3
bone marrow	FLI1	path:04512_1
bone marrow	FLI1	path:04512_3
bone marrow	IRF5	path:04060_23
bone marrow	IRF5	path:04620_1
bone marrow	IRF5	path:04620_3
bone marrow	IRF5	path:04620_7
bone marrow	IRF5	path:04620_2
bone marrow	IRF5	path:04620_4
bone marrow	IRF5	path:04650_5
bone marrow	IRF5	path:04650_3
bone marrow	IRF5	path:05160_4
bone marrow	SPI1	path:04060_26
bone marrow	SPI1	path:04145_4
bone marrow	SPI1	path:04145_1
bone marrow	SPI1	path:04620_3
bone marrow	SPI1	path:04620_4
bone marrow	SPI1	path:04620_1
bone marrow	SPI1	path:04650_7
bone marrow	SPI1	path:04650_1
bone marrow	SPI1	path:04670_9
bone marrow	SPI1	path:04670_6
bone marrow	SPI1	path:04670_8
bone marrow	SPI1	path:04670_4
bone marrow	SPI1	path:04670_2
bone marrow	SPI1	path:04810_16
bone marrow	SPI1	path:05140_3
bone marrow	SPI1	path:05140_2
bone marrow	SPI1	path:05146_1
bone marrow	SPI1	path:05150_4
bone marrow	SPI1	path:05200_34
bone marrow	TP53	path:04010_11
bone marrow	TP53	path:04012_1
bone marrow	TP53	path:04012_5
bone marrow	TP53	path:04110_11
bone marrow	TP53	path:04110_2
bone marrow	TP53	path:04110_10
bone marrow	TP53	path:04110_1
bone marrow	TP53	path:04110_9
bone marrow	TP53	path:04110_7

bone marrow	TP53	path:04110_5
bone marrow	TP53	path:04110_13
bone marrow	TP53	path:04110_6
bone marrow	TP53	path:04115_2
bone marrow	TP53	path:04210_9
bone marrow	TP53	path:04210_8
bone marrow	TP53	path:04360_3
bone marrow	TP53	path:04510_6
bone marrow	TP53	path:04510_19
bone marrow	TP53	path:04510_13
bone marrow	TP53	path:04510_12
bone marrow	TP53	path:04510_4
bone marrow	TP53	path:04650_3
bone marrow	TP53	path:04722_15
bone marrow	TP53	path:04912_7
bone marrow	TP53	path:05014_2
bone marrow	TP53	path:05160_10
bone marrow	TP53	path:05160_9
bone marrow	TP53	path:05200_7
bone marrow	TP53	path:05200_6
bone marrow	TP53	path:05200_24
bone marrow	TP53	path:05200_10
bone marrow	TP53	path:05200_2
bone marrow	TP53	path:05200_19
bone marrow	TP53	path:05200_1
bone marrow	TP53	path:05200_17
bone marrow	TP53	path:05200_37
bone marrow	TP53	path:05200_14
bone marrow	TP53	path:05200_25
bone marrow	TP53	path:05200_38
bone marrow	TP53	path:05200_15
bone marrow	TP53	path:05200_26
bone marrow	TP53	path:05200_13
bone marrow	TP53	path:05200_16
bone marrow	TP53	path:05200_8
bone marrow	TP53	path:05200_18
bone marrow	TP53	path:05200_20
bone marrow	TP53	path:05200_9
bone marrow	TP53	path:05200_21
bone marrow	TP53	path:05200_3
bone marrow	TP53	path:05200_39
bone marrow	TP53	path:05213_3
bone marrow	TP53	path:05214_1
bone marrow	TP53	path:05214_2

bone marrow	TP53	path:05214_3
bone marrow	TP53	path:05215_6
bone marrow	TP53	path:05215_4
bone marrow	TP53	path:05215_9
bone marrow	TP53	path:05215_7
bone marrow	TP53	path:05215_10
bone marrow	TP53	path:05218_3
bone marrow	TP53	path:05218_1
bone marrow	TP53	path:05218_2
bone marrow	TP53	path:05218_5
bone marrow	TP53	path:05219_3
bone marrow	TP53	path:05219_5
bone marrow	TP53	path:05220_4
bone marrow	TP53	path:05223_2
brain	CREB1	path:04514_19
brain	CREB1	path:04612_3
brain	CREB1	path:04620_6
brain	CREB1	path:04620_9
brain	CREB1	path:04620_8
brain	CREB1	path:04620_4
brain	CREB1	path:04620_5
brain	CREB1	path:05142_3
brain	FOXO3	path:04920_2
brain	NR4A1	path:00140_1
brain	SREBF1	path:04910_4
brain	SREBF1	path:04910_1
brain	TP53	path:04010_11
brain	TP53	path:04012_1
brain	TP53	path:04012_5
brain	TP53	path:04110_10
brain	TP53	path:04110_11
brain	TP53	path:04110_2
brain	TP53	path:04110_7
brain	TP53	path:04110_9
brain	TP53	path:04110_13
brain	TP53	path:04110_5
brain	TP53	path:04110_1
brain	TP53	path:04110_6
brain	TP53	path:04115_2
brain	TP53	path:04210_9
brain	TP53	path:04210_8
brain	TP53	path:04360_3
brain	TP53	path:04510_6
brain	TP53	path:04510_19

**brain**	**TP53**	**path:04510_4**
**brain**	**TP53**	**path:04510_13**
**brain**	**TP53**	**path:04510_12**
**brain**	**TP53**	**path:04650_3**
**brain**	**TP53**	**path:04722_15**
**brain**	**TP53**	**path:04912_7**
**brain**	**TP53**	**path:05014_2**
**brain**	**TP53**	**path:05160_10**
**brain**	**TP53**	**path:05160_9**
**brain**	**TP53**	**path:05200_37**
**brain**	**TP53**	**path:05200_6**
**brain**	**TP53**	**path:05200_26**
**brain**	**TP53**	**path:05200_10**
**brain**	**TP53**	**path:05200_2**
**brain**	**TP53**	**path:05200_9**
**brain**	**TP53**	**path:05200_13**
**brain**	**TP53**	**path:05200_24**
**brain**	**TP53**	**path:05200_17**
**brain**	**TP53**	**path:05200_39**
**brain**	**TP53**	**path:05200_7**
**brain**	**TP53**	**path:05200_25**
**brain**	**TP53**	**path:05200_3**
**brain**	**TP53**	**path:05200_38**
**brain**	**TP53**	**path:05200_20**
**brain**	**TP53**	**path:05200_16**
**brain**	**TP53**	**path:05200_15**
**brain**	**TP53**	**path:05200_14**
**brain**	**TP53**	**path:05200_8**
**brain**	**TP53**	**path:05200_1**
**brain**	**TP53**	**path:05200_18**
**brain**	**TP53**	**path:05200_19**
**brain**	**TP53**	**path:05200_21**
**brain**	**TP53**	**path:05213_3**
**brain**	**TP53**	**path:05214_1**
**brain**	**TP53**	**path:05214_2**
**brain**	**TP53**	**path:05214_3**
**brain**	**TP53**	**path:05215_6**
**brain**	**TP53**	**path:05215_4**
**brain**	**TP53**	**path:05215_7**
**brain**	**TP53**	**path:05215_9**
**brain**	**TP53**	**path:05215_10**
**brain**	**TP53**	**path:05218_2**
**brain**	**TP53**	**path:05218_3**
**brain**	**TP53**	**path:05218_1**

brain	TP53	path:05218_5
brain	TP53	path:05219_5
brain	TP53	path:05219_3
brain	TP53	path:05220_4
brain	TP53	path:05223_2
breast	TCF7	path:04660_15
breast	TP63	path:04110_5
breast	TP63	path:04110_10
breast	TP63	path:04110_11
breast	TP63	path:04110_1
breast	TP63	path:04110_2
breast	TP63	path:04110_7
breast	TP63	path:04110_9
breast	TP63	path:04115_2
breast	TP63	path:05200_15
breast	TP63	path:05200_21
breast	TP63	path:05200_7
breast	TP63	path:05200_20
breast	TP63	path:05200_2
breast	TP63	path:05200_39
breast	TP63	path:05200_37
breast	TP63	path:05200_13
breast	TP63	path:05200_9
breast	TP63	path:05200_1
breast	TP63	path:05200_25
breast	TP63	path:05200_14
breast	TP63	path:05200_3
breast	TP63	path:05200_19
breast	TP63	path:05214_3
breast	TP63	path:05215_4
breast	TP63	path:05215_9
breast	TP63	path:05215_10
breast	TP63	path:05215_6
breast	TP63	path:05218_3
breast	TP63	path:05218_5
breast	TP63	path:05219_3
breast	TP63	path:05219_5
breast	TP63	path:05220_4
breast cancer	ETS2	path:04310_5
breast cancer	ETS2	path:04310_7
breast cancer	ETS2	path:05200_19
breast cancer	ETS2	path:05200_17
breast cancer	ETS2	path:05200_9
breast cancer	ETS2	path:05200_2

**breast cancer**	**ETV4**	**path:05200_39**
**breast cancer**	**ETV4**	**path:05200_25**
**breast cancer**	**ETV4**	**path:05200_38**
**breast cancer**	**ETV4**	**path:05200_37**
**breast cancer**	**ETV4**	**path:05200_35**
**breast cancer**	**HNF1A**	**path:00140_1**
**breast cancer**	**HNF1A**	**path:00980_1**
**breast cancer**	**HNF1A**	**path:00982_2**
**breast cancer**	**HNF1A**	**path:04610_2**
**breast cancer**	**HNF1A**	**path:04610_4**
**breast cancer**	**HNF1A**	**path:04610_6**
**breast cancer**	**HNF1A**	**path:04610_3**
**breast cancer**	**HNF1A**	**path:04950_3**
**Caco**	**GATA4**	**path:00590_2**
**Caco**	**GATA4**	**path:00590_1**
**Caco**	**GATA4**	**path:00591_1**
**Caco**	**GATA4**	**path:00980_1**
**Caco**	**GATA4**	**path:00982_2**
**Caco**	**GATA6**	**path:00590_1**
**Caco**	**GATA6**	**path:00590_2**
**Caco**	**GATA6**	**path:00591_1**
**Caco**	**HNF1A**	**path:00140_1**
**Caco**	**HNF1A**	**path:00980_1**
**Caco**	**HNF1A**	**path:00982_2**
**Caco**	**HNF1A**	**path:04610_2**
**Caco**	**HNF1A**	**path:04610_4**
**Caco**	**HNF1A**	**path:04610_3**
**Caco**	**HNF1A**	**path:04610_6**
**Caco**	**HNF1A**	**path:04950_3**
**colon**	**FOXO3**	**path:04920_2**
**colon**	**HNF4A**	**path:00010_1**
**colon**	**HNF4A**	**path:00120_1**
**colon**	**HNF4A**	**path:00140_1**
**colon**	**HNF4A**	**path:00561_2**
**colon**	**HNF4A**	**path:00590_2**
**colon**	**HNF4A**	**path:00590_1**
**colon**	**HNF4A**	**path:00591_1**
**colon**	**HNF4A**	**path:00830_1**
**colon**	**HNF4A**	**path:00980_1**
**colon**	**HNF4A**	**path:00982_2**
**colon**	**HNF4A**	**path:03320_1**
**colon**	**HNF4A**	**path:04610_6**
**colon**	**HNF4A**	**path:04610_7**
**colon**	**HNF4A**	**path:04610_2**

colon	HNF4A	path:04610_1
colon	HNF4A	path:04610_5
colon	HNF4A	path:04610_4
colon	HNF4A	path:04610_3
colon	HNF4A	path:04950_2
colon	HNF4A	path:04950_3
colon	TP53	path:04010_11
colon	TP53	path:04012_1
colon	TP53	path:04012_5
colon	TP53	path:04110_2
colon	TP53	path:04110_9
colon	TP53	path:04110_11
colon	TP53	path:04110_13
colon	TP53	path:04110_1
colon	TP53	path:04110_7
colon	TP53	path:04110_5
colon	TP53	path:04110_10
colon	TP53	path:04110_6
colon	TP53	path:04115_2
colon	TP53	path:04210_9
colon	TP53	path:04210_8
colon	TP53	path:04360_3
colon	TP53	path:04510_19
colon	TP53	path:04510_6
colon	TP53	path:04510_4
colon	TP53	path:04510_13
colon	TP53	path:04510_12
colon	TP53	path:04650_3
colon	TP53	path:04722_15
colon	TP53	path:04912_7
colon	TP53	path:05014_2
colon	TP53	path:05160_9
colon	TP53	path:05160_10
colon	TP53	path:05200_6
colon	TP53	path:05200_7
colon	TP53	path:05200_10
colon	TP53	path:05200_15
colon	TP53	path:05200_24
colon	TP53	path:05200_25
colon	TP53	path:05200_38
colon	TP53	path:05200_1
colon	TP53	path:05200_2
colon	TP53	path:05200_9
colon	TP53	path:05200_14

colon	TP53	path:05200_16
colon	TP53	path:05200_13
colon	TP53	path:05200_20
colon	TP53	path:05200_26
colon	TP53	path:05200_3
colon	TP53	path:05200_17
colon	TP53	path:05200_37
colon	TP53	path:05200_19
colon	TP53	path:05200_8
colon	TP53	path:05200_39
colon	TP53	path:05200_18
colon	TP53	path:05200_21
colon	TP53	path:05213_3
colon	TP53	path:05214_2
colon	TP53	path:05214_1
colon	TP53	path:05214_3
colon	TP53	path:05215_6
colon	TP53	path:05215_7
colon	TP53	path:05215_4
colon	TP53	path:05215_9
colon	TP53	path:05215_10
colon	TP53	path:05218_2
colon	TP53	path:05218_1
colon	TP53	path:05218_3
colon	TP53	path:05218_5
colon	TP53	path:05219_3
colon	TP53	path:05219_5
colon	TP53	path:05220_4
colon	TP53	path:05223_2
erythroid	GATA1	path:04060_32
erythroid	POU2F1	path:00232_1
erythroid	POU2F1	path:00590_2
erythroid	POU2F1	path:00590_1
erythroid	POU2F1	path:00591_1
erythroid	POU2F1	path:00830_1
erythroid	POU2F1	path:00980_1
erythroid	POU2F1	path:00982_2
erythroid	POU2F1	path:04060_4
erythroid	POU2F1	path:04110_1
erythroid	POU2F1	path:04110_13
erythroid	POU2F1	path:04110_5
erythroid	POU2F1	path:04110_11
erythroid	POU2F1	path:04110_9
erythroid	POU2F1	path:04110_10

erythroid	POU2F1	path:04350_1
erythroid	POU2F1	path:04620_4
erythroid	POU2F1	path:04664_1
erythroid	POU2F1	path:05200_38
erythroid	POU2F1	path:05200_35
erythroid	POU2F1	path:05200_25
erythroid	POU2F1	path:05200_37
erythroid	POU2F1	path:05200_39
erythroid	POU2F1	path:05200_26
erythroid	POU2F1	path:05200_24
erythroid	POU2F1	path:05200_20
erythroid	POU2F1	path:05200_18
erythroid	POU2F1	path:05200_21
erythroid	POU2F1	path:05200_33
erythroid	POU2F1	path:05200_15
erythroid	POU2F1	path:05216_3
erythroid	POU2F1	path:05219_5
erythroid	POU2F1	path:05219_3
erythroid	POU2F1	path:05222_1
erythroid	POU2F1	path:05222_5
heart	CREB1	path:04514_19
heart	CREB1	path:04612_3
heart	CREB1	path:04620_8
heart	CREB1	path:04620_5
heart	CREB1	path:04620_9
heart	CREB1	path:04620_4
heart	CREB1	path:04620_6
heart	CREB1	path:05142_3
heart	FLI1	path:04512_1
heart	FLI1	path:04512_3
heart	FOXO3	path:04920_2
heart	NR1I3	path:00830_1
heart	NR1I3	path:00980_1
heart	NR1I3	path:00982_2
heart	SREBF1	path:04910_4
heart	SREBF1	path:04910_1
heart	TP63	path:04110_11
heart	TP63	path:04110_10
heart	TP63	path:04110_5
heart	TP63	path:04110_7
heart	TP63	path:04110_1
heart	TP63	path:04110_9
heart	TP63	path:04110_2
heart	TP63	path:04115_2

heart	TP63	path:05200_21
heart	TP63	path:05200_7
heart	TP63	path:05200_9
heart	TP63	path:05200_3
heart	TP63	path:05200_14
heart	TP63	path:05200_39
heart	TP63	path:05200_15
heart	TP63	path:05200_25
heart	TP63	path:05200_20
heart	TP63	path:05200_13
heart	TP63	path:05200_2
heart	TP63	path:05200_1
heart	TP63	path:05200_19
heart	TP63	path:05200_37
heart	TP63	path:05214_3
heart	TP63	path:05215_4
heart	TP63	path:05215_6
heart	TP63	path:05215_9
heart	TP63	path:05215_10
heart	TP63	path:05218_3
heart	TP63	path:05218_5
heart	TP63	path:05219_3
heart	TP63	path:05219_5
heart	TP63	path:05220_4
HeLa	ELK1	path:04010_11
HeLa	ELK1	path:04010_4
HeLa	ELK1	path:05020_3
HeLa	ELK1	path:05020_4
HeLa	ELK1	path:05110_3
HeLa	ELK1	path:05200_18
HeLa	ELK1	path:05200_26
HeLa	NFIC	path:00140_1
HeLa	NFIC	path:00591_1
HeLa	NFIC	path:04010_22
HeLa	NFIC	path:04080_5
HeLa	NFIC	path:04115_2
HeLa	NFIC	path:04510_6
HeLa	NFIC	path:04512_2
HeLa	NFIC	path:04660_10
HeLa	NFIC	path:04660_9
HeLa	NFIC	path:04662_5
HeLa	NFIC	path:04662_9
HeLa	NFIC	path:04722_15
HeLa	NFIC	path:05200_18

HeLa	NFIC	path:05200_2
HeLa	NFIC	path:05200_7
HeLa	NFIC	path:05200_13
HeLa	NFIC	path:05200_6
HeLa	NFIC	path:05200_26
HeLa	NFIC	path:05200_25
HeLa	NFIC	path:05200_37
HeLa	NFIC	path:05200_39
HeLa	NFIC	path:05200_24
HeLa	NFIC	path:05200_15
HeLa	NFIC	path:05200_21
HeLa	NFIC	path:05214_3
HeLa	NFIC	path:05218_1
HeLa	NFIC	path:05218_2
HeLa	NFIC	path:05218_3
HeLa	NFIC	path:05219_3
HeLa	NFIC	path:05220_4
HeLa	NFYA	path:04110_11
HeLa	NFYA	path:04110_6
HeLa	NFYA	path:04110_10
HeLa	NFYA	path:04110_5
HeLa	NFYA	path:04110_9
HeLa	NFYA	path:04110_7
HeLa	NFYA	path:04110_2
HeLa	NFYA	path:04110_1
HeLa	NFYA	path:04110_4
HeLa	NFYA	path:04115_2
HeLa	NR2F1	path:03320_1
HeLa	REL	path:04612_5
HeLa	REL	path:04620_1
HeLa	REL	path:04620_4
HeLa	REL	path:04620_3
HeLa	REL	path:04650_14
HeLa	REL	path:04650_15
HeLa	REL	path:04650_13
HeLa	REL	path:04650_5
HeLa	SP1	path:00140_1
HeLa	SP1	path:00330_1
HeLa	SP1	path:00330_4
HeLa	SP1	path:00330_5
HeLa	SP1	path:00330_3
HeLa	SP1	path:00350_2
HeLa	SP1	path:00590_2
HeLa	SP1	path:00980_1

HeLa	SP1	path:04010_7
HeLa	SP1	path:04010_6
HeLa	SP1	path:04010_8
HeLa	SP1	path:04060_19
HeLa	SP1	path:04060_32
HeLa	SP1	path:04060_10
HeLa	SP1	path:04060_20
HeLa	SP1	path:04060_46
HeLa	SP1	path:04110_7
HeLa	SP1	path:04110_13
HeLa	SP1	path:04110_11
HeLa	SP1	path:04110_4
HeLa	SP1	path:04110_5
HeLa	SP1	path:04110_10
HeLa	SP1	path:04110_9
HeLa	SP1	path:04110_1
HeLa	SP1	path:04110_2
HeLa	SP1	path:04115_2
HeLa	SP1	path:04144_2
HeLa	SP1	path:04210_5
HeLa	SP1	path:04210_4
HeLa	SP1	path:04210_9
HeLa	SP1	path:04210_6
HeLa	SP1	path:04310_5
HeLa	SP1	path:04310_7
HeLa	SP1	path:04350_5
HeLa	SP1	path:04350_4
HeLa	SP1	path:04350_1
HeLa	SP1	path:04510_4
HeLa	SP1	path:04510_3
HeLa	SP1	path:04510_5
HeLa	SP1	path:04510_10
HeLa	SP1	path:04510_14
HeLa	SP1	path:04510_19
HeLa	SP1	path:04510_16
HeLa	SP1	path:04510_6
HeLa	SP1	path:04510_7
HeLa	SP1	path:04510_1
HeLa	SP1	path:04510_13
HeLa	SP1	path:04510_12
HeLa	SP1	path:04510_17
HeLa	SP1	path:04512_2
HeLa	SP1	path:04512_3
HeLa	SP1	path:04512_1

HeLa	SP1	path:04520_2
HeLa	SP1	path:04520_5
HeLa	SP1	path:04540_7
HeLa	SP1	path:04610_2
HeLa	SP1	path:04610_6
HeLa	SP1	path:04610_1
HeLa	SP1	path:04610_5
HeLa	SP1	path:04610_7
HeLa	SP1	path:04610_4
HeLa	SP1	path:04610_3
HeLa	SP1	path:04620_5
HeLa	SP1	path:04620_6
HeLa	SP1	path:04620_9
HeLa	SP1	path:04620_8
HeLa	SP1	path:04630_2
HeLa	SP1	path:04630_1
HeLa	SP1	path:04650_3
HeLa	SP1	path:04660_9
HeLa	SP1	path:04662_9
HeLa	SP1	path:04722_15
HeLa	SP1	path:04810_16
HeLa	SP1	path:04912_7
HeLa	SP1	path:04940_2
HeLa	SP1	path:05014_1
HeLa	SP1	path:05014_2
HeLa	SP1	path:05020_2
HeLa	SP1	path:05020_1
HeLa	SP1	path:05100_6
HeLa	SP1	path:05140_4
HeLa	SP1	path:05140_6
HeLa	SP1	path:05142_11
HeLa	SP1	path:05142_12
HeLa	SP1	path:05142_3
HeLa	SP1	path:05145_8
HeLa	SP1	path:05145_6
HeLa	SP1	path:05146_1
HeLa	SP1	path:05200_31
HeLa	SP1	path:05200_7
HeLa	SP1	path:05200_2
HeLa	SP1	path:05200_21
HeLa	SP1	path:05200_42
HeLa	SP1	path:05200_19
HeLa	SP1	path:05200_39
HeLa	SP1	path:05200_1

HeLa	SP1	path:05200_38
HeLa	SP1	path:05200_16
HeLa	SP1	path:05200_35
HeLa	SP1	path:05200_8
HeLa	SP1	path:05200_9
HeLa	SP1	path:05200_33
HeLa	SP1	path:05200_17
HeLa	SP1	path:05200_37
HeLa	SP1	path:05200_13
HeLa	SP1	path:05200_6
HeLa	SP1	path:05200_24
HeLa	SP1	path:05200_25
HeLa	SP1	path:05200_18
HeLa	SP1	path:05200_41
HeLa	SP1	path:05200_29
HeLa	SP1	path:05200_34
HeLa	SP1	path:05200_20
HeLa	SP1	path:05200_10
HeLa	SP1	path:05200_30
HeLa	SP1	path:05200_32
HeLa	SP1	path:05200_15
HeLa	SP1	path:05200_14
HeLa	SP1	path:05200_26
HeLa	SP1	path:05210_8
HeLa	SP1	path:05210_5
HeLa	SP1	path:05212_3
HeLa	SP1	path:05212_8
HeLa	SP1	path:05212_10
HeLa	SP1	path:05212_9
HeLa	SP1	path:05214_3
HeLa	SP1	path:05214_1
HeLa	SP1	path:05214_2
HeLa	SP1	path:05215_10
HeLa	SP1	path:05215_4
HeLa	SP1	path:05215_9
HeLa	SP1	path:05215_5
HeLa	SP1	path:05215_7
HeLa	SP1	path:05215_6
HeLa	SP1	path:05216_3
HeLa	SP1	path:05218_1
HeLa	SP1	path:05218_3
HeLa	SP1	path:05218_2
HeLa	SP1	path:05218_4
HeLa	SP1	path:05218_5

**HeLa**	**SP1**	**path:05219_3**
**HeLa**	**SP1**	**path:05219_5**
**HeLa**	**SP1**	**path:05220_8**
**HeLa**	**SP1**	**path:05220_4**
**HeLa**	**SP1**	**path:05220_5**
**HeLa**	**SP1**	**path:05221_2**
**HeLa**	**SP1**	**path:05222_5**
**HeLa**	**SP1**	**path:05222_2**
**HeLa**	**SP1**	**path:05222_1**
**HeLa**	**SP1**	**path:05223_3**
**HeLa**	**SP1**	**path:05223_1**
**HeLa**	**SP1**	**path:05320_2**
**HeLa**	**SP1**	**path:05330_2**
**HeLa**	**SP1**	**path:05332_1**
**HeLa**	**SP1**	**path:05414_2**
**HeLa**	**SRF**	**path:05020_3**
**HeLa**	**SRF**	**path:05020_4**
**HeLa**	**SRF**	**path:05410_1**
**HeLa**	**SRF**	**path:05416_1**
**HeLa**	**TFAP2A**	**path:04010_7**
**HeLa**	**TFAP2A**	**path:04012_5**
**HeLa**	**TFAP2A**	**path:04020_1**
**HeLa**	**TFAP2A**	**path:04080_5**
**HeLa**	**TFAP2A**	**path:04144_2**
**HeLa**	**TFAP2A**	**path:04510_5**
**HeLa**	**TFAP2A**	**path:04510_4**
**HeLa**	**TFAP2A**	**path:04510_19**
**HeLa**	**TFAP2A**	**path:04510_13**
**HeLa**	**TFAP2A**	**path:04510_12**
**HeLa**	**TFAP2A**	**path:04510_7**
**HeLa**	**TFAP2A**	**path:04510_6**
**HeLa**	**TFAP2A**	**path:04520_3**
**HeLa**	**TFAP2A**	**path:04520_5**
**HeLa**	**TFAP2A**	**path:04520_7**
**HeLa**	**TFAP2A**	**path:04520_2**
**HeLa**	**TFAP2A**	**path:04912_7**
**HeLa**	**TFAP2A**	**path:05200_26**
**HeLa**	**TFAP2A**	**path:05200_24**
**HeLa**	**TFAP2A**	**path:05200_29**
**HeLa**	**TFAP2A**	**path:05200_37**
**HeLa**	**TFAP2A**	**path:05200_19**
**HeLa**	**TFAP2A**	**path:05200_6**
**HeLa**	**TFAP2A**	**path:05200_7**
**HeLa**	**TFAP2A**	**path:05200_38**

HeLa	TFAP2A	path:05200_35
HeLa	TFAP2A	path:05200_39
HeLa	TFAP2A	path:05200_25
HeLa	TFAP2A	path:05200_17
HeLa	TFAP2A	path:05200_8
HeLa	TFAP2A	path:05200_9
HeLa	TFAP2A	path:05200_30
HeLa	TFAP2A	path:05200_2
HeLa	TFAP2A	path:05200_10
HeLa	TFAP2A	path:05200_16
HeLa	TFAP2A	path:05212_3
HeLa	TFAP2A	path:05212_8
HeLa	TFAP2A	path:05214_2
HeLa	TFAP2A	path:05214_1
HeLa	TFAP2A	path:05215_9
HeLa	TFAP2A	path:05215_8
HeLa	TFAP2A	path:05215_7
HeLa	TFAP2A	path:05215_10
HeLa	TFAP2A	path:05219_2
HeLa	TFAP2A	path:05223_1
HeLa	VDR	path:05200_19
HeLa	VDR	path:05200_10
HeLa	VDR	path:05200_7
HeLa	VDR	path:05200_16
hematopoietic	FOXA3	path:00140_1
hematopoietic	FOXA3	path:00591_1
hematopoietic	FOXA3	path:00830_1
hematopoietic	FOXA3	path:00980_1
hematopoietic	FOXA3	path:00982_2
hematopoietic	STAT6	path:00590_2
hematopoietic	STAT6	path:00590_1
hematopoietic	STAT6	path:04630_2
Hep3B	EGR1	path:04144_2
Hep3B	EGR1	path:04350_1
Hep3B	EGR1	path:04350_5
Hep3B	EGR1	path:04510_6
Hep3B	EGR1	path:04510_5
Hep3B	EGR1	path:04510_7
Hep3B	EGR1	path:04510_12
Hep3B	EGR1	path:04510_13
Hep3B	EGR1	path:04512_1
Hep3B	EGR1	path:04512_2
Hep3B	EGR1	path:04512_3
Hep3B	EGR1	path:05020_2

**Hep3B**	**EGR1**	**path:05200_24**
**Hep3B**	**EGR1**	**path:05200_37**
**Hep3B**	**EGR1**	**path:05200_35**
**Hep3B**	**EGR1**	**path:05200_29**
**Hep3B**	**EGR1**	**path:05200_16**
**Hep3B**	**EGR1**	**path:05200_31**
**Hep3B**	**EGR1**	**path:05200_30**
**Hep3B**	**EGR1**	**path:05200_25**
**Hep3B**	**EGR1**	**path:05212_3**
**Hep3B**	**EGR1**	**path:05212_8**
**Hep3B**	**EGR1**	**path:05215_7**
**Hep3B**	**EGR1**	**path:05215_9**
**Hep3B**	**EGR1**	**path:05218_4**
**Hep3B**	**GATA2**	**path:04060_32**
**Hep3B**	**HNF4A**	**path:00010_1**
**Hep3B**	**HNF4A**	**path:00120_1**
**Hep3B**	**HNF4A**	**path:00140_1**
**Hep3B**	**HNF4A**	**path:00561_2**
**Hep3B**	**HNF4A**	**path:00590_1**
**Hep3B**	**HNF4A**	**path:00590_2**
**Hep3B**	**HNF4A**	**path:00591_1**
**Hep3B**	**HNF4A**	**path:00830_1**
**Hep3B**	**HNF4A**	**path:00980_1**
**Hep3B**	**HNF4A**	**path:00982_2**
**Hep3B**	**HNF4A**	**path:03320_1**
**Hep3B**	**HNF4A**	**path:04610_7**
**Hep3B**	**HNF4A**	**path:04610_5**
**Hep3B**	**HNF4A**	**path:04610_2**
**Hep3B**	**HNF4A**	**path:04610_1**
**Hep3B**	**HNF4A**	**path:04610_6**
**Hep3B**	**HNF4A**	**path:04610_4**
**Hep3B**	**HNF4A**	**path:04610_3**
**Hep3B**	**HNF4A**	**path:04950_2**
**Hep3B**	**HNF4A**	**path:04950_3**
**hepatic**	**CEBPA**	**path:04060_37**
**hepatic**	**CEBPA**	**path:04610_1**
**hepatic**	**CEBPA**	**path:04610_4**
**hepatic**	**CEBPA**	**path:04610_5**
**hepatic**	**CEBPA**	**path:04610_2**
**hepatic**	**CEBPA**	**path:04610_3**
**HepG2**	**GATA2**	**path:04060_32**
**HepG2**	**GATA3**	**path:04612_4**
**HepG2**	**NFE2L2**	**path:00480_1**
**HEPG2**	**STAT5B**	**path:04630_1**

**HEPG2**	**STAT5B**	**path:04630_2**
**HEPG2**	**STAT5B**	**path:05200_33**
**HEPG2**	**STAT5B**	**path:05200_25**
**HEPG2**	**STAT5B**	**path:05200_26**
**HEPG2**	**STAT5B**	**path:05200_39**
**HEPG2**	**STAT5B**	**path:05200_35**
**HEPG2**	**STAT5B**	**path:05200_24**
**HEPG2**	**STAT5B**	**path:05200_37**
**HEPG2**	**STAT5B**	**path:05200_38**
**HEPG2**	**STAT5B**	**path:05200_21**
**HEPG2**	**STAT5B**	**path:05214_3**
**HEPG2**	**STAT5B**	**path:05218_1**
**HEPG2**	**STAT5B**	**path:05218_2**
**HEPG2**	**STAT5B**	**path:05218_5**
**HEPG2**	**STAT5B**	**path:05218_3**
**HEPG2**	**STAT5B**	**path:05219_5**
**HEPG2**	**STAT5B**	**path:05219_3**
**HEPG2**	**STAT5B**	**path:05220_4**
**HL60**	**KLF4**	**path:04110_1**
**HL60**	**KLF4**	**path:04110_5**
**HL60**	**KLF4**	**path:04110_11**
**HL60**	**KLF4**	**path:04110_10**
**HL60**	**KLF4**	**path:04110_13**
**HL60**	**KLF4**	**path:04110_9**
**HL60**	**KLF4**	**path:04115_2**
**HL60**	**KLF4**	**path:05145_11**
**HL60**	**KLF4**	**path:05200_15**
**HL60**	**KLF4**	**path:05200_25**
**HL60**	**KLF4**	**path:05200_37**
**HL60**	**KLF4**	**path:05200_13**
**HL60**	**KLF4**	**path:05200_33**
**HL60**	**KLF4**	**path:05200_21**
**HL60**	**KLF4**	**path:05200_39**
**HL60**	**KLF4**	**path:05200_9**
**HL60**	**KLF4**	**path:05200_14**
**HL60**	**KLF4**	**path:05200_35**
**HL60**	**KLF4**	**path:05200_38**
**HL60**	**KLF4**	**path:05200_2**
**HL60**	**KLF4**	**path:05210_8**
**HL60**	**KLF4**	**path:05210_5**
**HL60**	**KLF4**	**path:05214_3**
**HL60**	**KLF4**	**path:05219_5**
**HL60**	**KLF4**	**path:05219_3**
**HL60**	**KLF4**	**path:05220_4**

HL60	TP73	path:04115_2
HL60	TP73	path:04722_15
HL60	TP73	path:05200_33
HL60	VDR	path:05200_19
HL60	VDR	path:05200_16
HL60	VDR	path:05200_10
HL60	VDR	path:05200_7
HUVEC	EGR1	path:04144_2
HUVEC	EGR1	path:04350_1
HUVEC	EGR1	path:04350_5
HUVEC	EGR1	path:04510_6
HUVEC	EGR1	path:04510_12
HUVEC	EGR1	path:04510_5
HUVEC	EGR1	path:04510_7
HUVEC	EGR1	path:04510_13
HUVEC	EGR1	path:04512_1
HUVEC	EGR1	path:04512_3
HUVEC	EGR1	path:04512_2
HUVEC	EGR1	path:05020_2
HUVEC	EGR1	path:05200_24
HUVEC	EGR1	path:05200_29
HUVEC	EGR1	path:05200_37
HUVEC	EGR1	path:05200_35
HUVEC	EGR1	path:05200_16
HUVEC	EGR1	path:05200_31
HUVEC	EGR1	path:05200_30
HUVEC	EGR1	path:05200_25
HUVEC	EGR1	path:05212_3
HUVEC	EGR1	path:05212_8
HUVEC	EGR1	path:05215_7
HUVEC	EGR1	path:05215_9
HUVEC	EGR1	path:05218_4
HUVEC	REL	path:04612_5
HUVEC	REL	path:04620_1
HUVEC	REL	path:04620_4
HUVEC	REL	path:04620_3
HUVEC	REL	path:04650_15
HUVEC	REL	path:04650_14
HUVEC	REL	path:04650_13
HUVEC	REL	path:04650_5
HUVEC	RELA	path:04060_44
HUVEC	RELA	path:04060_35
HUVEC	RELA	path:04062_1
HUVEC	RELA	path:04062_2

HUVEC	RELA	path:04110_10
HUVEC	RELA	path:04110_9
HUVEC	RELA	path:04110_1
HUVEC	RELA	path:04110_2
HUVEC	RELA	path:04110_7
HUVEC	RELA	path:04110_5
HUVEC	RELA	path:04110_11
HUVEC	RELA	path:04115_2
HUVEC	RELA	path:04612_5
HUVEC	RELA	path:04620_8
HUVEC	RELA	path:04620_9
HUVEC	RELA	path:04620_5
HUVEC	RELA	path:04620_1
HUVEC	RELA	path:04620_6
HUVEC	RELA	path:04620_4
HUVEC	RELA	path:04620_3
HUVEC	RELA	path:04620_7
HUVEC	RELA	path:04650_13
HUVEC	RELA	path:04650_5
HUVEC	RELA	path:04650_14
HUVEC	RELA	path:04650_15
HUVEC	RELA	path:04650_3
HUVEC	RELA	path:04670_6
HUVEC	RELA	path:04670_9
HUVEC	RELA	path:04670_8
HUVEC	RELA	path:04670_2
HUVEC	RELA	path:04670_4
HUVEC	RELA	path:05142_3
HUVEC	RELA	path:05200_15
HUVEC	RELA	path:05200_35
HUVEC	RELA	path:05200_21
HUVEC	RELA	path:05200_37
HUVEC	RELA	path:05200_13
HUVEC	RELA	path:05200_25
HUVEC	RELA	path:05200_39
HUVEC	RELA	path:05200_38
HUVEC	RELA	path:05200_2
HUVEC	RELA	path:05219_3
HUVEC	SP1	path:00140_1
HUVEC	SP1	path:00330_4
HUVEC	SP1	path:00330_1
HUVEC	SP1	path:00330_3
HUVEC	SP1	path:00330_5
HUVEC	SP1	path:00350_2

**HUVEC**	**SP1**	**path:00590_2**
**HUVEC**	**SP1**	**path:00980_1**
**HUVEC**	**SP1**	**path:04010_6**
**HUVEC**	**SP1**	**path:04010_8**
**HUVEC**	**SP1**	**path:04010_7**
**HUVEC**	**SP1**	**path:04060_19**
**HUVEC**	**SP1**	**path:04060_32**
**HUVEC**	**SP1**	**path:04060_10**
**HUVEC**	**SP1**	**path:04060_20**
**HUVEC**	**SP1**	**path:04060_46**
**HUVEC**	**SP1**	**path:04110_7**
**HUVEC**	**SP1**	**path:04110_9**
**HUVEC**	**SP1**	**path:04110_4**
**HUVEC**	**SP1**	**path:04110_11**
**HUVEC**	**SP1**	**path:04110_10**
**HUVEC**	**SP1**	**path:04110_13**
**HUVEC**	**SP1**	**path:04110_5**
**HUVEC**	**SP1**	**path:04110_2**
**HUVEC**	**SP1**	**path:04110_1**
**HUVEC**	**SP1**	**path:04115_2**
**HUVEC**	**SP1**	**path:04144_2**
**HUVEC**	**SP1**	**path:04210_5**
**HUVEC**	**SP1**	**path:04210_4**
**HUVEC**	**SP1**	**path:04210_9**
**HUVEC**	**SP1**	**path:04210_6**
**HUVEC**	**SP1**	**path:04310_7**
**HUVEC**	**SP1**	**path:04310_5**
**HUVEC**	**SP1**	**path:04350_4**
**HUVEC**	**SP1**	**path:04350_5**
**HUVEC**	**SP1**	**path:04350_1**
**HUVEC**	**SP1**	**path:04510_6**
**HUVEC**	**SP1**	**path:04510_4**
**HUVEC**	**SP1**	**path:04510_7**
**HUVEC**	**SP1**	**path:04510_14**
**HUVEC**	**SP1**	**path:04510_17**
**HUVEC**	**SP1**	**path:04510_5**
**HUVEC**	**SP1**	**path:04510_13**
**HUVEC**	**SP1**	**path:04510_19**
**HUVEC**	**SP1**	**path:04510_10**
**HUVEC**	**SP1**	**path:04510_16**
**HUVEC**	**SP1**	**path:04510_3**
**HUVEC**	**SP1**	**path:04510_1**
**HUVEC**	**SP1**	**path:04510_12**
**HUVEC**	**SP1**	**path:04512_2**

**HUVEC**	**SP1**	**path:04512_1**
**HUVEC**	**SP1**	**path:04512_3**
**HUVEC**	**SP1**	**path:04520_5**
**HUVEC**	**SP1**	**path:04520_2**
**HUVEC**	**SP1**	**path:04540_7**
**HUVEC**	**SP1**	**path:04610_5**
**HUVEC**	**SP1**	**path:04610_7**
**HUVEC**	**SP1**	**path:04610_4**
**HUVEC**	**SP1**	**path:04610_2**
**HUVEC**	**SP1**	**path:04610_1**
**HUVEC**	**SP1**	**path:04610_6**
**HUVEC**	**SP1**	**path:04610_3**
**HUVEC**	**SP1**	**path:04620_8**
**HUVEC**	**SP1**	**path:04620_6**
**HUVEC**	**SP1**	**path:04620_5**
**HUVEC**	**SP1**	**path:04620_9**
**HUVEC**	**SP1**	**path:04630_2**
**HUVEC**	**SP1**	**path:04630_1**
**HUVEC**	**SP1**	**path:04650_3**
**HUVEC**	**SP1**	**path:04660_9**
**HUVEC**	**SP1**	**path:04662_9**
**HUVEC**	**SP1**	**path:04722_15**
**HUVEC**	**SP1**	**path:04810_16**
**HUVEC**	**SP1**	**path:04912_7**
**HUVEC**	**SP1**	**path:04940_2**
**HUVEC**	**SP1**	**path:05014_2**
**HUVEC**	**SP1**	**path:05014_1**
**HUVEC**	**SP1**	**path:05020_1**
**HUVEC**	**SP1**	**path:05020_2**
**HUVEC**	**SP1**	**path:05100_6**
**HUVEC**	**SP1**	**path:05140_4**
**HUVEC**	**SP1**	**path:05140_6**
**HUVEC**	**SP1**	**path:05142_11**
**HUVEC**	**SP1**	**path:05142_3**
**HUVEC**	**SP1**	**path:05142_12**
**HUVEC**	**SP1**	**path:05145_6**
**HUVEC**	**SP1**	**path:05145_8**
**HUVEC**	**SP1**	**path:05146_1**
**HUVEC**	**SP1**	**path:05200_31**
**HUVEC**	**SP1**	**path:05200_2**
**HUVEC**	**SP1**	**path:05200_21**
**HUVEC**	**SP1**	**path:05200_39**
**HUVEC**	**SP1**	**path:05200_7**
**HUVEC**	**SP1**	**path:05200_19**

HUVEC	SP1	path:05200_42
HUVEC	SP1	path:05200_34
HUVEC	SP1	path:05200_38
HUVEC	SP1	path:05200_25
HUVEC	SP1	path:05200_16
HUVEC	SP1	path:05200_41
HUVEC	SP1	path:05200_35
HUVEC	SP1	path:05200_32
HUVEC	SP1	path:05200_8
HUVEC	SP1	path:05200_9
HUVEC	SP1	path:05200_15
HUVEC	SP1	path:05200_33
HUVEC	SP1	path:05200_13
HUVEC	SP1	path:05200_37
HUVEC	SP1	path:05200_6
HUVEC	SP1	path:05200_24
HUVEC	SP1	path:05200_1
HUVEC	SP1	path:05200_30
HUVEC	SP1	path:05200_29
HUVEC	SP1	path:05200_18
HUVEC	SP1	path:05200_10
HUVEC	SP1	path:05200_17
HUVEC	SP1	path:05200_20
HUVEC	SP1	path:05200_14
HUVEC	SP1	path:05200_26
HUVEC	SP1	path:05210_8
HUVEC	SP1	path:05210_5
HUVEC	SP1	path:05212_3
HUVEC	SP1	path:05212_8
HUVEC	SP1	path:05212_10
HUVEC	SP1	path:05212_9
HUVEC	SP1	path:05214_1
HUVEC	SP1	path:05214_3
HUVEC	SP1	path:05214_2
HUVEC	SP1	path:05215_10
HUVEC	SP1	path:05215_9
HUVEC	SP1	path:05215_4
HUVEC	SP1	path:05215_5
HUVEC	SP1	path:05215_7
HUVEC	SP1	path:05215_6
HUVEC	SP1	path:05216_3
HUVEC	SP1	path:05218_4
HUVEC	SP1	path:05218_3
HUVEC	SP1	path:05218_1

**HUVEC**	**SP1**	**path:05218_2**
**HUVEC**	**SP1**	**path:05218_5**
**HUVEC**	**SP1**	**path:05219_5**
**HUVEC**	**SP1**	**path:05219_3**
**HUVEC**	**SP1**	**path:05220_8**
**HUVEC**	**SP1**	**path:05220_4**
**HUVEC**	**SP1**	**path:05220_5**
**HUVEC**	**SP1**	**path:05221_2**
**HUVEC**	**SP1**	**path:05222_5**
**HUVEC**	**SP1**	**path:05222_2**
**HUVEC**	**SP1**	**path:05222_1**
**HUVEC**	**SP1**	**path:05223_1**
**HUVEC**	**SP1**	**path:05223_3**
**HUVEC**	**SP1**	**path:05320_2**
**HUVEC**	**SP1**	**path:05330_2**
**HUVEC**	**SP1**	**path:05332_1**
**HUVEC**	**SP1**	**path:05414_2**
**IARC-EW17**	**ELK1**	**path:04010_4**
**IARC-EW17**	**ELK1**	**path:04010_11**
**IARC-EW17**	**ELK1**	**path:05020_4**
**IARC-EW17**	**ELK1**	**path:05020_3**
**IARC-EW17**	**ELK1**	**path:05110_3**
**IARC-EW17**	**ELK1**	**path:05200_26**
**IARC-EW17**	**ELK1**	**path:05200_18**
**IARC-EW17**	**FLI1**	**path:04512_1**
**IARC-EW17**	**FLI1**	**path:04512_3**
**intestine**	**AKNA**	**path:04060_18**
**intestine**	**AKNA**	**path:04514_8**
**intestine**	**AKNA**	**path:04672_2**
**intestine**	**AKNA**	**path:05144_4**
**intestine**	**AKNA**	**path:05145_14**
**intestine**	**AKNA**	**path:05310_1**
**intestine**	**AKNA**	**path:05320_1**
**intestine**	**AKNA**	**path:05322_3**
**intestine**	**AKNA**	**path:05330_1**
**intestine**	**AKNA**	**path:05416_3**
**intestine**	**HNF1A**	**path:00140_1**
**intestine**	**HNF1A**	**path:00980_1**
**intestine**	**HNF1A**	**path:00982_2**
**intestine**	**HNF1A**	**path:04610_3**
**intestine**	**HNF1A**	**path:04610_2**
**intestine**	**HNF1A**	**path:04610_6**
**intestine**	**HNF1A**	**path:04610_4**
**intestine**	**HNF1A**	**path:04950_3**

intestine	HNF4A	path:00010_1
intestine	HNF4A	path:00120_1
intestine	HNF4A	path:00140_1
intestine	HNF4A	path:00561_2
intestine	HNF4A	path:00590_2
intestine	HNF4A	path:00590_1
intestine	HNF4A	path:00591_1
intestine	HNF4A	path:00830_1
intestine	HNF4A	path:00980_1
intestine	HNF4A	path:00982_2
intestine	HNF4A	path:03320_1
intestine	HNF4A	path:04610_7
intestine	HNF4A	path:04610_1
intestine	HNF4A	path:04610_6
intestine	HNF4A	path:04610_3
intestine	HNF4A	path:04610_5
intestine	HNF4A	path:04610_2
intestine	HNF4A	path:04610_4
intestine	HNF4A	path:04950_3
intestine	HNF4A	path:04950_2
intestine	TP53	path:04010_11
intestine	TP53	path:04012_1
intestine	TP53	path:04012_5
intestine	TP53	path:04110_9
intestine	TP53	path:04110_2
intestine	TP53	path:04110_11
intestine	TP53	path:04110_6
intestine	TP53	path:04110_13
intestine	TP53	path:04110_1
intestine	TP53	path:04110_10
intestine	TP53	path:04110_5
intestine	TP53	path:04110_7
intestine	TP53	path:04115_2
intestine	TP53	path:04210_8
intestine	TP53	path:04210_9
intestine	TP53	path:04360_3
intestine	TP53	path:04510_4
intestine	TP53	path:04510_19
intestine	TP53	path:04510_6
intestine	TP53	path:04510_12
intestine	TP53	path:04510_13
intestine	TP53	path:04650_3
intestine	TP53	path:04722_15
intestine	TP53	path:04912_7

**intestine**	**TP53**	**path:05014_2**
**intestine**	**TP53**	**path:05160_10**
**intestine**	**TP53**	**path:05160_9**
**intestine**	**TP53**	**path:05200_6**
**intestine**	**TP53**	**path:05200_15**
**intestine**	**TP53**	**path:05200_25**
**intestine**	**TP53**	**path:05200_38**
**intestine**	**TP53**	**path:05200_14**
**intestine**	**TP53**	**path:05200_16**
**intestine**	**TP53**	**path:05200_9**
**intestine**	**TP53**	**path:05200_24**
**intestine**	**TP53**	**path:05200_13**
**intestine**	**TP53**	**path:05200_26**
**intestine**	**TP53**	**path:05200_10**
**intestine**	**TP53**	**path:05200_37**
**intestine**	**TP53**	**path:05200_3**
**intestine**	**TP53**	**path:05200_2**
**intestine**	**TP53**	**path:05200_8**
**intestine**	**TP53**	**path:05200_7**
**intestine**	**TP53**	**path:05200_20**
**intestine**	**TP53**	**path:05200_1**
**intestine**	**TP53**	**path:05200_18**
**intestine**	**TP53**	**path:05200_17**
**intestine**	**TP53**	**path:05200_39**
**intestine**	**TP53**	**path:05200_19**
**intestine**	**TP53**	**path:05200_21**
**intestine**	**TP53**	**path:05213_3**
**intestine**	**TP53**	**path:05214_1**
**intestine**	**TP53**	**path:05214_2**
**intestine**	**TP53**	**path:05214_3**
**intestine**	**TP53**	**path:05215_6**
**intestine**	**TP53**	**path:05215_10**
**intestine**	**TP53**	**path:05215_7**
**intestine**	**TP53**	**path:05215_9**
**intestine**	**TP53**	**path:05215_4**
**intestine**	**TP53**	**path:05218_1**
**intestine**	**TP53**	**path:05218_5**
**intestine**	**TP53**	**path:05218_2**
**intestine**	**TP53**	**path:05218_3**
**intestine**	**TP53**	**path:05219_5**
**intestine**	**TP53**	**path:05219_3**
**intestine**	**TP53**	**path:05220_4**
**intestine**	**TP53**	**path:05223_2**
**JEG3**	**GATA2**	**path:04060_32**

**JEG3**	**GATA3**	**path:04612_4**
**Jurkat**	**ELK1**	**path:04010_4**
**Jurkat**	**ELK1**	**path:04010_11**
**Jurkat**	**ELK1**	**path:05020_3**
**Jurkat**	**ELK1**	**path:05020_4**
**Jurkat**	**ELK1**	**path:05110_3**
**Jurkat**	**ELK1**	**path:05200_18**
**Jurkat**	**ELK1**	**path:05200_26**
**Jurkat**	**FLI1**	**path:04512_3**
**Jurkat**	**FLI1**	**path:04512_1**
**Jurkat**	**KLF4**	**path:04110_5**
**Jurkat**	**KLF4**	**path:04110_1**
**Jurkat**	**KLF4**	**path:04110_13**
**Jurkat**	**KLF4**	**path:04110_11**
**Jurkat**	**KLF4**	**path:04110_9**
**Jurkat**	**KLF4**	**path:04110_10**
**Jurkat**	**KLF4**	**path:04115_2**
**Jurkat**	**KLF4**	**path:05145_11**
**Jurkat**	**KLF4**	**path:05200_15**
**Jurkat**	**KLF4**	**path:05200_25**
**Jurkat**	**KLF4**	**path:05200_9**
**Jurkat**	**KLF4**	**path:05200_35**
**Jurkat**	**KLF4**	**path:05200_13**
**Jurkat**	**KLF4**	**path:05200_2**
**Jurkat**	**KLF4**	**path:05200_33**
**Jurkat**	**KLF4**	**path:05200_37**
**Jurkat**	**KLF4**	**path:05200_39**
**Jurkat**	**KLF4**	**path:05200_21**
**Jurkat**	**KLF4**	**path:05200_14**
**Jurkat**	**KLF4**	**path:05200_38**
**Jurkat**	**KLF4**	**path:05210_5**
**Jurkat**	**KLF4**	**path:05210_8**
**Jurkat**	**KLF4**	**path:05214_3**
**Jurkat**	**KLF4**	**path:05219_5**
**Jurkat**	**KLF4**	**path:05219_3**
**Jurkat**	**KLF4**	**path:05220_4**
**Jurkat**	**RELA**	**path:04060_44**
**Jurkat**	**RELA**	**path:04060_35**
**Jurkat**	**RELA**	**path:04062_1**
**Jurkat**	**RELA**	**path:04062_2**
**Jurkat**	**RELA**	**path:04110_2**
**Jurkat**	**RELA**	**path:04110_10**
**Jurkat**	**RELA**	**path:04110_1**
**Jurkat**	**RELA**	**path:04110_9**

**Jurkat**	**RELA**	**path:04110_7**
**Jurkat**	**RELA**	**path:04110_11**
**Jurkat**	**RELA**	**path:04110_5**
**Jurkat**	**RELA**	**path:04115_2**
**Jurkat**	**RELA**	**path:04612_5**
**Jurkat**	**RELA**	**path:04620_5**
**Jurkat**	**RELA**	**path:04620_8**
**Jurkat**	**RELA**	**path:04620_1**
**Jurkat**	**RELA**	**path:04620_7**
**Jurkat**	**RELA**	**path:04620_9**
**Jurkat**	**RELA**	**path:04620_4**
**Jurkat**	**RELA**	**path:04620_3**
**Jurkat**	**RELA**	**path:04620_6**
**Jurkat**	**RELA**	**path:04650_5**
**Jurkat**	**RELA**	**path:04650_13**
**Jurkat**	**RELA**	**path:04650_3**
**Jurkat**	**RELA**	**path:04650_15**
**Jurkat**	**RELA**	**path:04650_14**
**Jurkat**	**RELA**	**path:04670_6**
**Jurkat**	**RELA**	**path:04670_8**
**Jurkat**	**RELA**	**path:04670_4**
**Jurkat**	**RELA**	**path:04670_9**
**Jurkat**	**RELA**	**path:04670_2**
**Jurkat**	**RELA**	**path:05142_3**
**Jurkat**	**RELA**	**path:05200_37**
**Jurkat**	**RELA**	**path:05200_35**
**Jurkat**	**RELA**	**path:05200_39**
**Jurkat**	**RELA**	**path:05200_15**
**Jurkat**	**RELA**	**path:05200_25**
**Jurkat**	**RELA**	**path:05200_13**
**Jurkat**	**RELA**	**path:05200_21**
**Jurkat**	**RELA**	**path:05200_38**
**Jurkat**	**RELA**	**path:05200_2**
**Jurkat**	**RELA**	**path:05219_3**
**K562**	**EGR1**	**path:04144_2**
**K562**	**EGR1**	**path:04350_5**
**K562**	**EGR1**	**path:04350_1**
**K562**	**EGR1**	**path:04510_6**
**K562**	**EGR1**	**path:04510_13**
**K562**	**EGR1**	**path:04510_7**
**K562**	**EGR1**	**path:04510_5**
**K562**	**EGR1**	**path:04510_12**
**K562**	**EGR1**	**path:04512_1**
**K562**	**EGR1**	**path:04512_2**

K562	EGR1	path:04512_3
K562	EGR1	path:05020_2
K562	EGR1	path:05200_24
K562	EGR1	path:05200_29
K562	EGR1	path:05200_37
K562	EGR1	path:05200_35
K562	EGR1	path:05200_16
K562	EGR1	path:05200_31
K562	EGR1	path:05200_30
K562	EGR1	path:05200_25
K562	EGR1	path:05212_3
K562	EGR1	path:05212_8
K562	EGR1	path:05215_7
K562	EGR1	path:05215_9
K562	EGR1	path:05218_4
K562	ELK1	path:04010_11
K562	ELK1	path:04010_4
K562	ELK1	path:05020_3
K562	ELK1	path:05020_4
K562	ELK1	path:05110_3
K562	ELK1	path:05200_18
K562	ELK1	path:05200_26
K562	FOS	path:04115_2
K562	FOS	path:04310_7
K562	FOS	path:04610_6
K562	FOS	path:04610_7
K562	FOS	path:04620_6
K562	FOS	path:04620_5
K562	FOS	path:04620_8
K562	FOS	path:04620_9
K562	FOS	path:04670_2
K562	FOS	path:05200_24
K562	FOS	path:05200_25
K562	FOS	path:05200_38
K562	FOS	path:05200_39
K562	FOS	path:05200_35
K562	FOS	path:05200_37
K562	FOS	path:05219_3
K562	GATA1	path:04060_32
K562	NFE2L2	path:00480_1
K562	RELA	path:04060_44
K562	RELA	path:04060_35
K562	RELA	path:04062_1
K562	RELA	path:04062_2

K562	RELA	path:04110_10
K562	RELA	path:04110_1
K562	RELA	path:04110_2
K562	RELA	path:04110_9
K562	RELA	path:04110_11
K562	RELA	path:04110_5
K562	RELA	path:04110_7
K562	RELA	path:04115_2
K562	RELA	path:04612_5
K562	RELA	path:04620_5
K562	RELA	path:04620_8
K562	RELA	path:04620_9
K562	RELA	path:04620_1
K562	RELA	path:04620_7
K562	RELA	path:04620_3
K562	RELA	path:04620_6
K562	RELA	path:04620_4
K562	RELA	path:04650_3
K562	RELA	path:04650_5
K562	RELA	path:04650_13
K562	RELA	path:04650_14
K562	RELA	path:04650_15
K562	RELA	path:04670_6
K562	RELA	path:04670_2
K562	RELA	path:04670_4
K562	RELA	path:04670_8
K562	RELA	path:04670_9
K562	RELA	path:05142_3
K562	RELA	path:05200_37
K562	RELA	path:05200_35
K562	RELA	path:05200_15
K562	RELA	path:05200_25
K562	RELA	path:05200_39
K562	RELA	path:05200_13
K562	RELA	path:05200_21
K562	RELA	path:05200_38
K562	RELA	path:05200_2
K562	RELA	path:05219_3
Keratinocyte	CEBPA	path:04060_37
Keratinocyte	CEBPA	path:04610_2
Keratinocyte	CEBPA	path:04610_1
Keratinocyte	CEBPA	path:04610_4
Keratinocyte	CEBPA	path:04610_5
Keratinocyte	CEBPA	path:04610_3

**Keratinocyte**	**TFAP2A**	**path:04010_7**
**Keratinocyte**	**TFAP2A**	**path:04012_5**
**Keratinocyte**	**TFAP2A**	**path:04020_1**
**Keratinocyte**	**TFAP2A**	**path:04080_5**
**Keratinocyte**	**TFAP2A**	**path:04144_2**
**Keratinocyte**	**TFAP2A**	**path:04510_5**
**Keratinocyte**	**TFAP2A**	**path:04510_19**
**Keratinocyte**	**TFAP2A**	**path:04510_13**
**Keratinocyte**	**TFAP2A**	**path:04510_4**
**Keratinocyte**	**TFAP2A**	**path:04510_7**
**Keratinocyte**	**TFAP2A**	**path:04510_12**
**Keratinocyte**	**TFAP2A**	**path:04510_6**
**Keratinocyte**	**TFAP2A**	**path:04520_5**
**Keratinocyte**	**TFAP2A**	**path:04520_3**
**Keratinocyte**	**TFAP2A**	**path:04520_2**
**Keratinocyte**	**TFAP2A**	**path:04520_7**
**Keratinocyte**	**TFAP2A**	**path:04912_7**
**Keratinocyte**	**TFAP2A**	**path:05200_24**
**Keratinocyte**	**TFAP2A**	**path:05200_29**
**Keratinocyte**	**TFAP2A**	**path:05200_37**
**Keratinocyte**	**TFAP2A**	**path:05200_6**
**Keratinocyte**	**TFAP2A**	**path:05200_7**
**Keratinocyte**	**TFAP2A**	**path:05200_8**
**Keratinocyte**	**TFAP2A**	**path:05200_16**
**Keratinocyte**	**TFAP2A**	**path:05200_26**
**Keratinocyte**	**TFAP2A**	**path:05200_25**
**Keratinocyte**	**TFAP2A**	**path:05200_17**
**Keratinocyte**	**TFAP2A**	**path:05200_9**
**Keratinocyte**	**TFAP2A**	**path:05200_39**
**Keratinocyte**	**TFAP2A**	**path:05200_35**
**Keratinocyte**	**TFAP2A**	**path:05200_2**
**Keratinocyte**	**TFAP2A**	**path:05200_19**
**Keratinocyte**	**TFAP2A**	**path:05200_10**
**Keratinocyte**	**TFAP2A**	**path:05200_38**
**Keratinocyte**	**TFAP2A**	**path:05200_30**
**Keratinocyte**	**TFAP2A**	**path:05212_3**
**Keratinocyte**	**TFAP2A**	**path:05212_8**
**Keratinocyte**	**TFAP2A**	**path:05214_2**
**Keratinocyte**	**TFAP2A**	**path:05214_1**
**Keratinocyte**	**TFAP2A**	**path:05215_8**
**Keratinocyte**	**TFAP2A**	**path:05215_9**
**Keratinocyte**	**TFAP2A**	**path:05215_7**
**Keratinocyte**	**TFAP2A**	**path:05215_10**
**Keratinocyte**	**TFAP2A**	**path:05219_2**

Keratinocyte	TFAP2A	path:05223_1
Keratinocyte	TP63	path:04110_5
Keratinocyte	TP63	path:04110_10
Keratinocyte	TP63	path:04110_11
Keratinocyte	TP63	path:04110_1
Keratinocyte	TP63	path:04110_9
Keratinocyte	TP63	path:04110_2
Keratinocyte	TP63	path:04110_7
Keratinocyte	TP63	path:04115_2
Keratinocyte	TP63	path:05200_15
Keratinocyte	TP63	path:05200_2
Keratinocyte	TP63	path:05200_7
Keratinocyte	TP63	path:05200_39
Keratinocyte	TP63	path:05200_9
Keratinocyte	TP63	path:05200_37
Keratinocyte	TP63	path:05200_1
Keratinocyte	TP63	path:05200_19
Keratinocyte	TP63	path:05200_14
Keratinocyte	TP63	path:05200_20
Keratinocyte	TP63	path:05200_3
Keratinocyte	TP63	path:05200_13
Keratinocyte	TP63	path:05200_21
Keratinocyte	TP63	path:05200_25
Keratinocyte	TP63	path:05214_3
Keratinocyte	TP63	path:05215_4
Keratinocyte	TP63	path:05215_10
Keratinocyte	TP63	path:05215_9
Keratinocyte	TP63	path:05215_6
Keratinocyte	TP63	path:05218_5
Keratinocyte	TP63	path:05218_3
Keratinocyte	TP63	path:05219_5
Keratinocyte	TP63	path:05219_3
Keratinocyte	TP63	path:05220_4
kidney	CREB1	path:04514_19
kidney	CREB1	path:04612_3
kidney	CREB1	path:04620_6
kidney	CREB1	path:04620_9
kidney	CREB1	path:04620_5
kidney	CREB1	path:04620_8
kidney	CREB1	path:04620_4
kidney	CREB1	path:05142_3
kidney	FOXO3	path:04920_2
kidney	HNF1A	path:00140_1
kidney	HNF1A	path:00980_1

kidney	HNF1A	path:00982_2
kidney	HNF1A	path:04610_2
kidney	HNF1A	path:04610_6
kidney	HNF1A	path:04610_3
kidney	HNF1A	path:04610_4
kidney	HNF1A	path:04950_3
kidney	HNF4A	path:00010_1
kidney	HNF4A	path:00120_1
kidney	HNF4A	path:00140_1
kidney	HNF4A	path:00561_2
kidney	HNF4A	path:00590_2
kidney	HNF4A	path:00590_1
kidney	HNF4A	path:00591_1
kidney	HNF4A	path:00830_1
kidney	HNF4A	path:00980_1
kidney	HNF4A	path:00982_2
kidney	HNF4A	path:03320_1
kidney	HNF4A	path:04610_7
kidney	HNF4A	path:04610_6
kidney	HNF4A	path:04610_1
kidney	HNF4A	path:04610_5
kidney	HNF4A	path:04610_3
kidney	HNF4A	path:04610_2
kidney	HNF4A	path:04610_4
kidney	HNF4A	path:04950_2
kidney	HNF4A	path:04950_3
kidney	NR1I3	path:00830_1
kidney	NR1I3	path:00980_1
kidney	NR1I3	path:00982_2
kidney	SREBF1	path:04910_4
kidney	SREBF1	path:04910_1
leukemia	SPI1	path:04060_26
leukemia	SPI1	path:04145_4
leukemia	SPI1	path:04145_1
leukemia	SPI1	path:04620_3
leukemia	SPI1	path:04620_4
leukemia	SPI1	path:04620_1
leukemia	SPI1	path:04650_7
leukemia	SPI1	path:04650_1
leukemia	SPI1	path:04670_2
leukemia	SPI1	path:04670_9
leukemia	SPI1	path:04670_8
leukemia	SPI1	path:04670_4
leukemia	SPI1	path:04670_6

leukemia	SPI1	path:04810_16
leukemia	SPI1	path:05140_3
leukemia	SPI1	path:05140_2
leukemia	SPI1	path:05146_1
leukemia	SPI1	path:05150_4
leukemia	SPI1	path:05200_34
leukemia	TP73	path:04115_2
leukemia	TP73	path:04722_15
leukemia	TP73	path:05200_33
liver	AKNA	path:04060_18
liver	AKNA	path:04514_8
liver	AKNA	path:04672_2
liver	AKNA	path:05144_4
liver	AKNA	path:05145_14
liver	AKNA	path:05310_1
liver	AKNA	path:05320_1
liver	AKNA	path:05322_3
liver	AKNA	path:05330_1
liver	AKNA	path:05416_3
liver	CREB1	path:04514_19
liver	CREB1	path:04612_3
liver	CREB1	path:04620_9
liver	CREB1	path:04620_8
liver	CREB1	path:04620_4
liver	CREB1	path:04620_6
liver	CREB1	path:04620_5
liver	CREB1	path:05142_3
liver	FOXA2	path:00980_1
liver	FOXA2	path:04610_1
liver	FOXA2	path:04610_3
liver	FOXA2	path:04610_2
liver	FOXA2	path:04610_4
liver	FOXA2	path:04610_5
liver	FOXA3	path:00140_1
liver	FOXA3	path:00591_1
liver	FOXA3	path:00830_1
liver	FOXA3	path:00980_1
liver	FOXA3	path:00982_2
liver	FOXO3	path:04920_2
liver	HNF1A	path:00140_1
liver	HNF1A	path:00980_1
liver	HNF1A	path:00982_2
liver	HNF1A	path:04610_2
liver	HNF1A	path:04610_3

**liver**	**HNF1A**	**path:04610_6**
**liver**	**HNF1A**	**path:04610_4**
**liver**	**HNF1A**	**path:04950_3**
**liver**	**HNF4A**	**path:00010_1**
**liver**	**HNF4A**	**path:00120_1**
**liver**	**HNF4A**	**path:00140_1**
**liver**	**HNF4A**	**path:00561_2**
**liver**	**HNF4A**	**path:00590_1**
**liver**	**HNF4A**	**path:00590_2**
**liver**	**HNF4A**	**path:00591_1**
**liver**	**HNF4A**	**path:00830_1**
**liver**	**HNF4A**	**path:00980_1**
**liver**	**HNF4A**	**path:00982_2**
**liver**	**HNF4A**	**path:03320_1**
**liver**	**HNF4A**	**path:04610_1**
**liver**	**HNF4A**	**path:04610_6**
**liver**	**HNF4A**	**path:04610_7**
**liver**	**HNF4A**	**path:04610_2**
**liver**	**HNF4A**	**path:04610_5**
**liver**	**HNF4A**	**path:04610_3**
**liver**	**HNF4A**	**path:04610_4**
**liver**	**HNF4A**	**path:04950_2**
**liver**	**HNF4A**	**path:04950_3**
**liver**	**NR1I2**	**path:00830_1**
**liver**	**NR1I2**	**path:00980_1**
**liver**	**NR1I2**	**path:00982_2**
**liver**	**NR1I3**	**path:00830_1**
**liver**	**NR1I3**	**path:00980_1**
**liver**	**NR1I3**	**path:00982_2**
**liver**	**NR4A1**	**path:00140_1**
**liver**	**SPI1**	**path:04060_26**
**liver**	**SPI1**	**path:04145_1**
**liver**	**SPI1**	**path:04145_4**
**liver**	**SPI1**	**path:04620_4**
**liver**	**SPI1**	**path:04620_3**
**liver**	**SPI1**	**path:04620_1**
**liver**	**SPI1**	**path:04650_1**
**liver**	**SPI1**	**path:04650_7**
**liver**	**SPI1**	**path:04670_6**
**liver**	**SPI1**	**path:04670_2**
**liver**	**SPI1**	**path:04670_4**
**liver**	**SPI1**	**path:04670_9**
**liver**	**SPI1**	**path:04670_8**
**liver**	**SPI1**	**path:04810_16**

liver	SPI1	path:05140_2
liver	SPI1	path:05140_3
liver	SPI1	path:05146_1
liver	SPI1	path:05150_4
liver	SPI1	path:05200_34
liver	SREBF1	path:04910_4
liver	SREBF1	path:04910_1
LNCaP	NFIC	path:00140_1
LNCaP	NFIC	path:00591_1
LNCaP	NFIC	path:04010_22
LNCaP	NFIC	path:04080_5
LNCaP	NFIC	path:04115_2
LNCaP	NFIC	path:04510_6
LNCaP	NFIC	path:04512_2
LNCaP	NFIC	path:04660_10
LNCaP	NFIC	path:04660_9
LNCaP	NFIC	path:04662_9
LNCaP	NFIC	path:04662_5
LNCaP	NFIC	path:04722_15
LNCaP	NFIC	path:05200_18
LNCaP	NFIC	path:05200_2
LNCaP	NFIC	path:05200_37
LNCaP	NFIC	path:05200_7
LNCaP	NFIC	path:05200_13
LNCaP	NFIC	path:05200_39
LNCaP	NFIC	path:05200_24
LNCaP	NFIC	path:05200_26
LNCaP	NFIC	path:05200_25
LNCaP	NFIC	path:05200_6
LNCaP	NFIC	path:05200_21
LNCaP	NFIC	path:05200_15
LNCaP	NFIC	path:05214_3
LNCaP	NFIC	path:05218_3
LNCaP	NFIC	path:05218_1
LNCaP	NFIC	path:05218_2
LNCaP	NFIC	path:05219_3
LNCaP	NFIC	path:05220_4
lung	ELK1	path:04010_11
lung	ELK1	path:04010_4
lung	ELK1	path:05020_3
lung	ELK1	path:05020_4
lung	ELK1	path:05110_3
lung	ELK1	path:05200_26
lung	ELK1	path:05200_18

**lung**	**FOXO3**	**path:04920_2**
**lung**	**HIF1A**	**path:05200_37**
**lung**	**HIF1A**	**path:05200_39**
**lung**	**HIF1A**	**path:05200_25**
**lung**	**NR1I3**	**path:00830_1**
**lung**	**NR1I3**	**path:00980_1**
**lung**	**NR1I3**	**path:00982_2**
**lung**	**SREBF1**	**path:04910_4**
**lung**	**SREBF1**	**path:04910_1**
**lung**	**TP63**	**path:04110_5**
**lung**	**TP63**	**path:04110_1**
**lung**	**TP63**	**path:04110_11**
**lung**	**TP63**	**path:04110_2**
**lung**	**TP63**	**path:04110_10**
**lung**	**TP63**	**path:04110_9**
**lung**	**TP63**	**path:04110_7**
**lung**	**TP63**	**path:04115_2**
**lung**	**TP63**	**path:05200_2**
**lung**	**TP63**	**path:05200_39**
**lung**	**TP63**	**path:05200_9**
**lung**	**TP63**	**path:05200_37**
**lung**	**TP63**	**path:05200_3**
**lung**	**TP63**	**path:05200_14**
**lung**	**TP63**	**path:05200_15**
**lung**	**TP63**	**path:05200_7**
**lung**	**TP63**	**path:05200_1**
**lung**	**TP63**	**path:05200_25**
**lung**	**TP63**	**path:05200_13**
**lung**	**TP63**	**path:05200_20**
**lung**	**TP63**	**path:05200_19**
**lung**	**TP63**	**path:05200_21**
**lung**	**TP63**	**path:05214_3**
**lung**	**TP63**	**path:05215_4**
**lung**	**TP63**	**path:05215_6**
**lung**	**TP63**	**path:05215_10**
**lung**	**TP63**	**path:05215_9**
**lung**	**TP63**	**path:05218_5**
**lung**	**TP63**	**path:05218_3**
**lung**	**TP63**	**path:05219_5**
**lung**	**TP63**	**path:05219_3**
**lung**	**TP63**	**path:05220_4**
**lymph node**	**AKNA**	**path:04060_18**
**lymph node**	**AKNA**	**path:04514_8**
**lymph node**	**AKNA**	**path:04672_2**

lymph node	AKNA	path:05144_4
lymph node	AKNA	path:05145_14
lymph node	AKNA	path:05310_1
lymph node	AKNA	path:05320_1
lymph node	AKNA	path:05322_3
lymph node	AKNA	path:05330_1
lymph node	AKNA	path:05416_3
lymph node	IRF5	path:04060_23
lymph node	IRF5	path:04620_1
lymph node	IRF5	path:04620_3
lymph node	IRF5	path:04620_7
lymph node	IRF5	path:04620_2
lymph node	IRF5	path:04620_4
lymph node	IRF5	path:04650_5
lymph node	IRF5	path:04650_3
lymph node	IRF5	path:05160_4
lymph node	IRF7	path:04060_23
lymph node	IRF7	path:04620_8
lymph node	IRF7	path:04620_4
lymph node	IRF7	path:04620_7
lymph node	IRF7	path:04620_1
lymph node	IRF7	path:04620_3
lymph node	IRF7	path:04620_5
lymph node	IRF7	path:04620_6
lymph node	IRF7	path:04620_9
lymph node	IRF7	path:04620_2
lymph node	IRF7	path:04650_5
lymph node	IRF7	path:04650_3
lymph node	IRF7	path:05160_4
lymph node	TP73	path:04115_2
lymph node	TP73	path:04722_15
lymph node	TP73	path:05200_33
lymphocytes	AKNA	path:04060_18
lymphocytes	AKNA	path:04514_8
lymphocytes	AKNA	path:04672_2
lymphocytes	AKNA	path:05144_4
lymphocytes	AKNA	path:05145_14
lymphocytes	AKNA	path:05310_1
lymphocytes	AKNA	path:05320_1
lymphocytes	AKNA	path:05322_3
lymphocytes	AKNA	path:05330_1
lymphocytes	AKNA	path:05416_3
lymphocytes	CREB1	path:04514_19
lymphocytes	CREB1	path:04612_3

lymphocytes	CREB1	path:04620_9
lymphocytes	CREB1	path:04620_6
lymphocytes	CREB1	path:04620_8
lymphocytes	CREB1	path:04620_5
lymphocytes	CREB1	path:04620_4
lymphocytes	CREB1	path:05142_3
lymphocytes	FLI1	path:04512_1
lymphocytes	FLI1	path:04512_3
lymphocytes	NFYA	path:04110_9
lymphocytes	NFYA	path:04110_6
lymphocytes	NFYA	path:04110_11
lymphocytes	NFYA	path:04110_10
lymphocytes	NFYA	path:04110_7
lymphocytes	NFYA	path:04110_5
lymphocytes	NFYA	path:04110_2
lymphocytes	NFYA	path:04110_1
lymphocytes	NFYA	path:04110_4
lymphocytes	NFYA	path:04115_2
lymphocytes	TP53	path:04010_11
lymphocytes	TP53	path:04012_1
lymphocytes	TP53	path:04012_5
lymphocytes	TP53	path:04110_9
lymphocytes	TP53	path:04110_13
lymphocytes	TP53	path:04110_7
lymphocytes	TP53	path:04110_2
lymphocytes	TP53	path:04110_11
lymphocytes	TP53	path:04110_6
lymphocytes	TP53	path:04110_1
lymphocytes	TP53	path:04110_10
lymphocytes	TP53	path:04110_5
lymphocytes	TP53	path:04115_2
lymphocytes	TP53	path:04210_8
lymphocytes	TP53	path:04210_9
lymphocytes	TP53	path:04360_3
lymphocytes	TP53	path:04510_19
lymphocytes	TP53	path:04510_6
lymphocytes	TP53	path:04510_12
lymphocytes	TP53	path:04510_4
lymphocytes	TP53	path:04510_13
lymphocytes	TP53	path:04650_3
lymphocytes	TP53	path:04722_15
lymphocytes	TP53	path:04912_7
lymphocytes	TP53	path:05014_2
lymphocytes	TP53	path:05160_9

**lymphocytes**	**TP53**	**path:05160_10**
**lymphocytes**	**TP53**	**path:05200_6**
**lymphocytes**	**TP53**	**path:05200_15**
**lymphocytes**	**TP53**	**path:05200_7**
**lymphocytes**	**TP53**	**path:05200_24**
**lymphocytes**	**TP53**	**path:05200_25**
**lymphocytes**	**TP53**	**path:05200_38**
**lymphocytes**	**TP53**	**path:05200_14**
**lymphocytes**	**TP53**	**path:05200_16**
**lymphocytes**	**TP53**	**path:05200_9**
**lymphocytes**	**TP53**	**path:05200_13**
**lymphocytes**	**TP53**	**path:05200_2**
**lymphocytes**	**TP53**	**path:05200_20**
**lymphocytes**	**TP53**	**path:05200_26**
**lymphocytes**	**TP53**	**path:05200_3**
**lymphocytes**	**TP53**	**path:05200_17**
**lymphocytes**	**TP53**	**path:05200_18**
**lymphocytes**	**TP53**	**path:05200_37**
**lymphocytes**	**TP53**	**path:05200_10**
**lymphocytes**	**TP53**	**path:05200_1**
**lymphocytes**	**TP53**	**path:05200_39**
**lymphocytes**	**TP53**	**path:05200_8**
**lymphocytes**	**TP53**	**path:05200_19**
**lymphocytes**	**TP53**	**path:05200_21**
**lymphocytes**	**TP53**	**path:05213_3**
**lymphocytes**	**TP53**	**path:05214_2**
**lymphocytes**	**TP53**	**path:05214_1**
**lymphocytes**	**TP53**	**path:05214_3**
**lymphocytes**	**TP53**	**path:05215_6**
**lymphocytes**	**TP53**	**path:05215_9**
**lymphocytes**	**TP53**	**path:05215_7**
**lymphocytes**	**TP53**	**path:05215_4**
**lymphocytes**	**TP53**	**path:05215_10**
**lymphocytes**	**TP53**	**path:05218_2**
**lymphocytes**	**TP53**	**path:05218_1**
**lymphocytes**	**TP53**	**path:05218_3**
**lymphocytes**	**TP53**	**path:05218_5**
**lymphocytes**	**TP53**	**path:05219_3**
**lymphocytes**	**TP53**	**path:05219_5**
**lymphocytes**	**TP53**	**path:05220_4**
**lymphocytes**	**TP53**	**path:05223_2**
**macrophages**	**CEBPA**	**path:04060_37**
**macrophages**	**CEBPA**	**path:04610_5**
**macrophages**	**CEBPA**	**path:04610_1**

**macrophages**	**CEBPA**	**path:04610_2**
**macrophages**	**CEBPA**	**path:04610_4**
**macrophages**	**CEBPA**	**path:04610_3**
**macrophages**	**SPI1**	**path:04060_26**
**macrophages**	**SPI1**	**path:04145_1**
**macrophages**	**SPI1**	**path:04145_4**
**macrophages**	**SPI1**	**path:04620_4**
**macrophages**	**SPI1**	**path:04620_3**
**macrophages**	**SPI1**	**path:04620_1**
**macrophages**	**SPI1**	**path:04650_1**
**macrophages**	**SPI1**	**path:04650_7**
**macrophages**	**SPI1**	**path:04670_9**
**macrophages**	**SPI1**	**path:04670_6**
**macrophages**	**SPI1**	**path:04670_8**
**macrophages**	**SPI1**	**path:04670_4**
**macrophages**	**SPI1**	**path:04670_2**
**macrophages**	**SPI1**	**path:04810_16**
**macrophages**	**SPI1**	**path:05140_3**
**macrophages**	**SPI1**	**path:05140_2**
**macrophages**	**SPI1**	**path:05146_1**
**macrophages**	**SPI1**	**path:05150_4**
**macrophages**	**SPI1**	**path:05200_34**
**MCF7**	**E2F1**	**path:04110_13**
**MCF7**	**E2F1**	**path:04110_2**
**MCF7**	**E2F1**	**path:04110_4**
**MCF7**	**E2F1**	**path:04110_10**
**MCF7**	**E2F1**	**path:04110_9**
**MCF7**	**E2F1**	**path:04110_5**
**MCF7**	**E2F1**	**path:04110_1**
**MCF7**	**E2F1**	**path:04110_7**
**MCF7**	**E2F1**	**path:04110_6**
**MCF7**	**E2F1**	**path:04110_11**
**MCF7**	**E2F1**	**path:04115_2**
**MCF7**	**E2F1**	**path:04210_5**
**MCF7**	**E2F1**	**path:04510_10**
**MCF7**	**E2F1**	**path:05014_2**
**MCF7**	**E2F1**	**path:05200_35**
**MCF7**	**E2F1**	**path:05200_25**
**MCF7**	**E2F1**	**path:05200_37**
**MCF7**	**E2F1**	**path:05200_2**
**MCF7**	**E2F1**	**path:05200_26**
**MCF7**	**E2F1**	**path:05200_33**
**MCF7**	**E2F1**	**path:05200_15**
**MCF7**	**E2F1**	**path:05200_38**

MCF7	E2F1	path:05200_13
MCF7	E2F1	path:05200_21
MCF7	E2F1	path:05200_18
MCF7	E2F1	path:05200_39
MCF7	E2F1	path:05212_9
MCF7	E2F1	path:05214_3
MCF7	E2F1	path:05215_3
MCF7	E2F1	path:05218_5
MCF7	E2F1	path:05218_3
MCF7	E2F1	path:05219_3
MCF7	E2F1	path:05219_5
MCF7	E2F1	path:05220_4
MCF7	E2F1	path:05222_1
MCF7	E2F1	path:05222_5
MCF7	E2F1	path:05223_4
MCF7	E2F1	path:05223_3
MCF7	ESR1	path:00830_1
MCF7	ESR1	path:00980_1
MCF7	ESR1	path:00982_2
MCF7	ESR1	path:04115_2
MCF7	ESR1	path:04610_7
MCF7	ESR1	path:04610_2
MCF7	ESR1	path:04610_6
MCF7	ESR1	path:05200_35
MCF7	ESR1	path:05200_37
MCF7	ESR1	path:05200_30
MCF7	ESR1	path:05200_21
MCF7	ESR1	path:05200_38
MCF7	ESR1	path:05200_15
MCF7	ESR1	path:05200_24
MCF7	ESR1	path:05200_25
MCF7	ESR1	path:05200_39
MCF7	ESR1	path:05212_8
MCF7	ESR1	path:05219_3
MCF7	FOS	path:04115_2
MCF7	FOS	path:04310_7
MCF7	FOS	path:04610_6
MCF7	FOS	path:04610_7
MCF7	FOS	path:04620_8
MCF7	FOS	path:04620_9
MCF7	FOS	path:04620_5
MCF7	FOS	path:04620_6
MCF7	FOS	path:04670_2
MCF7	FOS	path:05200_24

**MCF7**	**FOS**	**path:05200_38**
**MCF7**	**FOS**	**path:05200_39**
**MCF7**	**FOS**	**path:05200_25**
**MCF7**	**FOS**	**path:05200_37**
**MCF7**	**FOS**	**path:05200_35**
**MCF7**	**FOS**	**path:05219_3**
**MCF7**	**GATA1**	**path:04060_32**
**MCF7**	**NFIC**	**path:00140_1**
**MCF7**	**NFIC**	**path:00591_1**
**MCF7**	**NFIC**	**path:04010_22**
**MCF7**	**NFIC**	**path:04080_5**
**MCF7**	**NFIC**	**path:04115_2**
**MCF7**	**NFIC**	**path:04510_6**
**MCF7**	**NFIC**	**path:04512_2**
**MCF7**	**NFIC**	**path:04660_10**
**MCF7**	**NFIC**	**path:04660_9**
**MCF7**	**NFIC**	**path:04662_9**
**MCF7**	**NFIC**	**path:04662_5**
**MCF7**	**NFIC**	**path:04722_15**
**MCF7**	**NFIC**	**path:05200_2**
**MCF7**	**NFIC**	**path:05200_7**
**MCF7**	**NFIC**	**path:05200_24**
**MCF7**	**NFIC**	**path:05200_13**
**MCF7**	**NFIC**	**path:05200_25**
**MCF7**	**NFIC**	**path:05200_6**
**MCF7**	**NFIC**	**path:05200_26**
**MCF7**	**NFIC**	**path:05200_37**
**MCF7**	**NFIC**	**path:05200_18**
**MCF7**	**NFIC**	**path:05200_15**
**MCF7**	**NFIC**	**path:05200_21**
**MCF7**	**NFIC**	**path:05200_39**
**MCF7**	**NFIC**	**path:05214_3**
**MCF7**	**NFIC**	**path:05218_1**
**MCF7**	**NFIC**	**path:05218_3**
**MCF7**	**NFIC**	**path:05218_2**
**MCF7**	**NFIC**	**path:05219_3**
**MCF7**	**NFIC**	**path:05220_4**
**MCF7**	**SP1**	**path:00140_1**
**MCF7**	**SP1**	**path:00330_5**
**MCF7**	**SP1**	**path:00330_1**
**MCF7**	**SP1**	**path:00330_4**
**MCF7**	**SP1**	**path:00330_3**
**MCF7**	**SP1**	**path:00350_2**
**MCF7**	**SP1**	**path:00590_2**

MCF7	SP1	path:00980_1
MCF7	SP1	path:04010_7
MCF7	SP1	path:04010_6
MCF7	SP1	path:04010_8
MCF7	SP1	path:04060_10
MCF7	SP1	path:04060_20
MCF7	SP1	path:04060_19
MCF7	SP1	path:04060_32
MCF7	SP1	path:04060_46
MCF7	SP1	path:04110_7
MCF7	SP1	path:04110_13
MCF7	SP1	path:04110_4
MCF7	SP1	path:04110_10
MCF7	SP1	path:04110_11
MCF7	SP1	path:04110_9
MCF7	SP1	path:04110_5
MCF7	SP1	path:04110_2
MCF7	SP1	path:04110_1
MCF7	SP1	path:04115_2
MCF7	SP1	path:04144_2
MCF7	SP1	path:04210_5
MCF7	SP1	path:04210_6
MCF7	SP1	path:04210_9
MCF7	SP1	path:04210_4
MCF7	SP1	path:04310_7
MCF7	SP1	path:04310_5
MCF7	SP1	path:04350_1
MCF7	SP1	path:04350_5
MCF7	SP1	path:04350_4
MCF7	SP1	path:04510_14
MCF7	SP1	path:04510_17
MCF7	SP1	path:04510_10
MCF7	SP1	path:04510_3
MCF7	SP1	path:04510_1
MCF7	SP1	path:04510_6
MCF7	SP1	path:04510_5
MCF7	SP1	path:04510_19
MCF7	SP1	path:04510_13
MCF7	SP1	path:04510_4
MCF7	SP1	path:04510_16
MCF7	SP1	path:04510_7
MCF7	SP1	path:04510_12
MCF7	SP1	path:04512_3
MCF7	SP1	path:04512_1

MCF7	SP1	path:04512_2
MCF7	SP1	path:04520_5
MCF7	SP1	path:04520_2
MCF7	SP1	path:04540_7
MCF7	SP1	path:04610_6
MCF7	SP1	path:04610_7
MCF7	SP1	path:04610_2
MCF7	SP1	path:04610_4
MCF7	SP1	path:04610_5
MCF7	SP1	path:04610_1
MCF7	SP1	path:04610_3
MCF7	SP1	path:04620_9
MCF7	SP1	path:04620_6
MCF7	SP1	path:04620_8
MCF7	SP1	path:04620_5
MCF7	SP1	path:04630_1
MCF7	SP1	path:04630_2
MCF7	SP1	path:04650_3
MCF7	SP1	path:04660_9
MCF7	SP1	path:04662_9
MCF7	SP1	path:04722_15
MCF7	SP1	path:04810_16
MCF7	SP1	path:04912_7
MCF7	SP1	path:04940_2
MCF7	SP1	path:05014_2
MCF7	SP1	path:05014_1
MCF7	SP1	path:05020_1
MCF7	SP1	path:05020_2
MCF7	SP1	path:05100_6
MCF7	SP1	path:05140_6
MCF7	SP1	path:05140_4
MCF7	SP1	path:05142_11
MCF7	SP1	path:05142_3
MCF7	SP1	path:05142_12
MCF7	SP1	path:05145_6
MCF7	SP1	path:05145_8
MCF7	SP1	path:05146_1
MCF7	SP1	path:05200_2
MCF7	SP1	path:05200_30
MCF7	SP1	path:05200_19
MCF7	SP1	path:05200_21
MCF7	SP1	path:05200_10
MCF7	SP1	path:05200_31
MCF7	SP1	path:05200_32

**MCF7**	**SP1**	**path:05200_8**
**MCF7**	**SP1**	**path:05200_39**
**MCF7**	**SP1**	**path:05200_34**
**MCF7**	**SP1**	**path:05200_20**
**MCF7**	**SP1**	**path:05200_18**
**MCF7**	**SP1**	**path:05200_14**
**MCF7**	**SP1**	**path:05200_35**
**MCF7**	**SP1**	**path:05200_6**
**MCF7**	**SP1**	**path:05200_9**
**MCF7**	**SP1**	**path:05200_29**
**MCF7**	**SP1**	**path:05200_37**
**MCF7**	**SP1**	**path:05200_24**
**MCF7**	**SP1**	**path:05200_42**
**MCF7**	**SP1**	**path:05200_1**
**MCF7**	**SP1**	**path:05200_13**
**MCF7**	**SP1**	**path:05200_26**
**MCF7**	**SP1**	**path:05200_38**
**MCF7**	**SP1**	**path:05200_25**
**MCF7**	**SP1**	**path:05200_7**
**MCF7**	**SP1**	**path:05200_16**
**MCF7**	**SP1**	**path:05200_41**
**MCF7**	**SP1**	**path:05200_15**
**MCF7**	**SP1**	**path:05200_17**
**MCF7**	**SP1**	**path:05200_33**
**MCF7**	**SP1**	**path:05210_8**
**MCF7**	**SP1**	**path:05210_5**
**MCF7**	**SP1**	**path:05212_10**
**MCF7**	**SP1**	**path:05212_3**
**MCF7**	**SP1**	**path:05212_9**
**MCF7**	**SP1**	**path:05212_8**
**MCF7**	**SP1**	**path:05214_1**
**MCF7**	**SP1**	**path:05214_3**
**MCF7**	**SP1**	**path:05214_2**
**MCF7**	**SP1**	**path:05215_9**
**MCF7**	**SP1**	**path:05215_5**
**MCF7**	**SP1**	**path:05215_7**
**MCF7**	**SP1**	**path:05215_10**
**MCF7**	**SP1**	**path:05215_6**
**MCF7**	**SP1**	**path:05215_4**
**MCF7**	**SP1**	**path:05216_3**
**MCF7**	**SP1**	**path:05218_3**
**MCF7**	**SP1**	**path:05218_1**
**MCF7**	**SP1**	**path:05218_2**
**MCF7**	**SP1**	**path:05218_5**

**MCF7**	**SP1**	**path:05218_4**
**MCF7**	**SP1**	**path:05219_5**
**MCF7**	**SP1**	**path:05219_3**
**MCF7**	**SP1**	**path:05220_4**
**MCF7**	**SP1**	**path:05220_8**
**MCF7**	**SP1**	**path:05220_5**
**MCF7**	**SP1**	**path:05221_2**
**MCF7**	**SP1**	**path:05222_2**
**MCF7**	**SP1**	**path:05222_5**
**MCF7**	**SP1**	**path:05222_1**
**MCF7**	**SP1**	**path:05223_1**
**MCF7**	**SP1**	**path:05223_3**
**MCF7**	**SP1**	**path:05320_2**
**MCF7**	**SP1**	**path:05330_2**
**MCF7**	**SP1**	**path:05332_1**
**MCF7**	**SP1**	**path:05414_2**
**MCF7**	**STAT3**	**path:04012_4**
**MCF7**	**STAT3**	**path:04060_1**
**MCF7**	**STAT3**	**path:04060_34**
**MCF7**	**STAT3**	**path:04630_1**
**MCF7**	**STAT3**	**path:04630_2**
**MCF7**	**STAT3**	**path:05142_3**
**MCF7**	**STAT3**	**path:05200_39**
**MCF7**	**STAT3**	**path:05200_25**
**MCF7**	**STAT3**	**path:05200_16**
**MCF7**	**STAT3**	**path:05200_21**
**MCF7**	**STAT3**	**path:05200_35**
**MCF7**	**STAT3**	**path:05200_37**
**MCF7**	**STAT3**	**path:05200_15**
**MCF7**	**STAT3**	**path:05200_24**
**MCF7**	**STAT3**	**path:05200_26**
**MCF7**	**STAT3**	**path:05200_30**
**MCF7**	**STAT3**	**path:05200_38**
**MCF7**	**STAT3**	**path:05200_18**
**MCF7**	**STAT3**	**path:05210_8**
**MCF7**	**STAT3**	**path:05210_5**
**MCF7**	**STAT3**	**path:05218_4**
**MCF7**	**STAT3**	**path:05221_1**
**MCF7**	**TFAP2C**	**path:04912_7**
**MCF7**	**TFAP2C**	**path:05219_2**
**MCF7**	**TP53**	**path:04010_11**
**MCF7**	**TP53**	**path:04012_1**
**MCF7**	**TP53**	**path:04012_5**
**MCF7**	**TP53**	**path:04110_10**

**MCF7**	**TP53**	**path:04110_13**
**MCF7**	**TP53**	**path:04110_5**
**MCF7**	**TP53**	**path:04110_2**
**MCF7**	**TP53**	**path:04110_1**
**MCF7**	**TP53**	**path:04110_11**
**MCF7**	**TP53**	**path:04110_7**
**MCF7**	**TP53**	**path:04110_6**
**MCF7**	**TP53**	**path:04110_9**
**MCF7**	**TP53**	**path:04115_2**
**MCF7**	**TP53**	**path:04210_9**
**MCF7**	**TP53**	**path:04210_8**
**MCF7**	**TP53**	**path:04360_3**
**MCF7**	**TP53**	**path:04510_19**
**MCF7**	**TP53**	**path:04510_13**
**MCF7**	**TP53**	**path:04510_12**
**MCF7**	**TP53**	**path:04510_4**
**MCF7**	**TP53**	**path:04510_6**
**MCF7**	**TP53**	**path:04650_3**
**MCF7**	**TP53**	**path:04722_15**
**MCF7**	**TP53**	**path:04912_7**
**MCF7**	**TP53**	**path:05014_2**
**MCF7**	**TP53**	**path:05160_10**
**MCF7**	**TP53**	**path:05160_9**
**MCF7**	**TP53**	**path:05200_7**
**MCF7**	**TP53**	**path:05200_37**
**MCF7**	**TP53**	**path:05200_1**
**MCF7**	**TP53**	**path:05200_2**
**MCF7**	**TP53**	**path:05200_18**
**MCF7**	**TP53**	**path:05200_38**
**MCF7**	**TP53**	**path:05200_6**
**MCF7**	**TP53**	**path:05200_19**
**MCF7**	**TP53**	**path:05200_26**
**MCF7**	**TP53**	**path:05200_25**
**MCF7**	**TP53**	**path:05200_10**
**MCF7**	**TP53**	**path:05200_39**
**MCF7**	**TP53**	**path:05200_15**
**MCF7**	**TP53**	**path:05200_14**
**MCF7**	**TP53**	**path:05200_16**
**MCF7**	**TP53**	**path:05200_17**
**MCF7**	**TP53**	**path:05200_24**
**MCF7**	**TP53**	**path:05200_20**
**MCF7**	**TP53**	**path:05200_9**
**MCF7**	**TP53**	**path:05200_13**
**MCF7**	**TP53**	**path:05200_3**

MCF7	TP53	path:05200_21
MCF7	TP53	path:05200_8
MCF7	TP53	path:05213_3
MCF7	TP53	path:05214_1
MCF7	TP53	path:05214_3
MCF7	TP53	path:05214_2
MCF7	TP53	path:05215_6
MCF7	TP53	path:05215_4
MCF7	TP53	path:05215_7
MCF7	TP53	path:05215_9
MCF7	TP53	path:05215_10
MCF7	TP53	path:05218_2
MCF7	TP53	path:05218_3
MCF7	TP53	path:05218_1
MCF7	TP53	path:05218_5
MCF7	TP53	path:05219_5
MCF7	TP53	path:05219_3
MCF7	TP53	path:05220_4
MCF7	TP53	path:05223_2
MCF7	VDR	path:05200_10
MCF7	VDR	path:05200_19
MCF7	VDR	path:05200_7
MCF7	VDR	path:05200_16
MDA-MB-231	E2F1	path:04110_10
MDA-MB-231	E2F1	path:04110_2
MDA-MB-231	E2F1	path:04110_4
MDA-MB-231	E2F1	path:04110_13
MDA-MB-231	E2F1	path:04110_5
MDA-MB-231	E2F1	path:04110_1
MDA-MB-231	E2F1	path:04110_11
MDA-MB-231	E2F1	path:04110_9
MDA-MB-231	E2F1	path:04110_7
MDA-MB-231	E2F1	path:04110_6
MDA-MB-231	E2F1	path:04115_2
MDA-MB-231	E2F1	path:04210_5
MDA-MB-231	E2F1	path:04510_10
MDA-MB-231	E2F1	path:05014_2
MDA-MB-231	E2F1	path:05200_37
MDA-MB-231	E2F1	path:05200_25
MDA-MB-231	E2F1	path:05200_26
MDA-MB-231	E2F1	path:05200_2
MDA-MB-231	E2F1	path:05200_18
MDA-MB-231	E2F1	path:05200_15
MDA-MB-231	E2F1	path:05200_13

**MDA-MB-231**	**E2F1**	**path:05200_35**
**MDA-MB-231**	**E2F1**	**path:05200_38**
**MDA-MB-231**	**E2F1**	**path:05200_21**
**MDA-MB-231**	**E2F1**	**path:05200_33**
**MDA-MB-231**	**E2F1**	**path:05200_39**
**MDA-MB-231**	**E2F1**	**path:05212_9**
**MDA-MB-231**	**E2F1**	**path:05214_3**
**MDA-MB-231**	**E2F1**	**path:05215_3**
**MDA-MB-231**	**E2F1**	**path:05218_5**
**MDA-MB-231**	**E2F1**	**path:05218_3**
**MDA-MB-231**	**E2F1**	**path:05219_5**
**MDA-MB-231**	**E2F1**	**path:05219_3**
**MDA-MB-231**	**E2F1**	**path:05220_4**
**MDA-MB-231**	**E2F1**	**path:05222_1**
**MDA-MB-231**	**E2F1**	**path:05222_5**
**MDA-MB-231**	**E2F1**	**path:05223_3**
**MDA-MB-231**	**E2F1**	**path:05223_4**
**MDA-MB-231**	**STAT3**	**path:04012_4**
**MDA-MB-231**	**STAT3**	**path:04060_1**
**MDA-MB-231**	**STAT3**	**path:04060_34**
**MDA-MB-231**	**STAT3**	**path:04630_1**
**MDA-MB-231**	**STAT3**	**path:04630_2**
**MDA-MB-231**	**STAT3**	**path:05142_3**
**MDA-MB-231**	**STAT3**	**path:05200_16**
**MDA-MB-231**	**STAT3**	**path:05200_21**
**MDA-MB-231**	**STAT3**	**path:05200_25**
**MDA-MB-231**	**STAT3**	**path:05200_37**
**MDA-MB-231**	**STAT3**	**path:05200_15**
**MDA-MB-231**	**STAT3**	**path:05200_24**
**MDA-MB-231**	**STAT3**	**path:05200_38**
**MDA-MB-231**	**STAT3**	**path:05200_39**
**MDA-MB-231**	**STAT3**	**path:05200_18**
**MDA-MB-231**	**STAT3**	**path:05200_26**
**MDA-MB-231**	**STAT3**	**path:05200_30**
**MDA-MB-231**	**STAT3**	**path:05200_35**
**MDA-MB-231**	**STAT3**	**path:05210_8**
**MDA-MB-231**	**STAT3**	**path:05210_5**
**MDA-MB-231**	**STAT3**	**path:05218_4**
**MDA-MB-231**	**STAT3**	**path:05221_1**
**MDA-MB-346**	**SP1**	**path:00140_1**
**MDA-MB-346**	**SP1**	**path:00330_1**
**MDA-MB-346**	**SP1**	**path:00330_5**
**MDA-MB-346**	**SP1**	**path:00330_4**
**MDA-MB-346**	**SP1**	**path:00330_3**

MDA-MB-346	SP1	path:00350_2
MDA-MB-346	SP1	path:00590_2
MDA-MB-346	SP1	path:00980_1
MDA-MB-346	SP1	path:04010_6
MDA-MB-346	SP1	path:04010_8
MDA-MB-346	SP1	path:04010_7
MDA-MB-346	SP1	path:04060_19
MDA-MB-346	SP1	path:04060_32
MDA-MB-346	SP1	path:04060_10
MDA-MB-346	SP1	path:04060_20
MDA-MB-346	SP1	path:04060_46
MDA-MB-346	SP1	path:04110_7
MDA-MB-346	SP1	path:04110_13
MDA-MB-346	SP1	path:04110_4
MDA-MB-346	SP1	path:04110_5
MDA-MB-346	SP1	path:04110_11
MDA-MB-346	SP1	path:04110_10
MDA-MB-346	SP1	path:04110_2
MDA-MB-346	SP1	path:04110_9
MDA-MB-346	SP1	path:04110_1
MDA-MB-346	SP1	path:04115_2
MDA-MB-346	SP1	path:04144_2
MDA-MB-346	SP1	path:04210_5
MDA-MB-346	SP1	path:04210_4
MDA-MB-346	SP1	path:04210_6
MDA-MB-346	SP1	path:04210_9
MDA-MB-346	SP1	path:04310_5
MDA-MB-346	SP1	path:04310_7
MDA-MB-346	SP1	path:04350_5
MDA-MB-346	SP1	path:04350_1
MDA-MB-346	SP1	path:04350_4
MDA-MB-346	SP1	path:04510_4
MDA-MB-346	SP1	path:04510_6
MDA-MB-346	SP1	path:04510_14
MDA-MB-346	SP1	path:04510_19
MDA-MB-346	SP1	path:04510_3
MDA-MB-346	SP1	path:04510_10
MDA-MB-346	SP1	path:04510_16
MDA-MB-346	SP1	path:04510_5
MDA-MB-346	SP1	path:04510_1
MDA-MB-346	SP1	path:04510_12
MDA-MB-346	SP1	path:04510_7
MDA-MB-346	SP1	path:04510_17
MDA-MB-346	SP1	path:04510_13

MDA-MB-346	SP1	path:04512_3
MDA-MB-346	SP1	path:04512_2
MDA-MB-346	SP1	path:04512_1
MDA-MB-346	SP1	path:04520_2
MDA-MB-346	SP1	path:04520_5
MDA-MB-346	SP1	path:04540_7
MDA-MB-346	SP1	path:04610_5
MDA-MB-346	SP1	path:04610_6
MDA-MB-346	SP1	path:04610_1
MDA-MB-346	SP1	path:04610_7
MDA-MB-346	SP1	path:04610_2
MDA-MB-346	SP1	path:04610_4
MDA-MB-346	SP1	path:04610_3
MDA-MB-346	SP1	path:04620_9
MDA-MB-346	SP1	path:04620_8
MDA-MB-346	SP1	path:04620_6
MDA-MB-346	SP1	path:04620_5
MDA-MB-346	SP1	path:04630_1
MDA-MB-346	SP1	path:04630_2
MDA-MB-346	SP1	path:04650_3
MDA-MB-346	SP1	path:04660_9
MDA-MB-346	SP1	path:04662_9
MDA-MB-346	SP1	path:04722_15
MDA-MB-346	SP1	path:04810_16
MDA-MB-346	SP1	path:04912_7
MDA-MB-346	SP1	path:04940_2
MDA-MB-346	SP1	path:05014_2
MDA-MB-346	SP1	path:05014_1
MDA-MB-346	SP1	path:05020_2
MDA-MB-346	SP1	path:05020_1
MDA-MB-346	SP1	path:05100_6
MDA-MB-346	SP1	path:05140_6
MDA-MB-346	SP1	path:05140_4
MDA-MB-346	SP1	path:05142_11
MDA-MB-346	SP1	path:05142_12
MDA-MB-346	SP1	path:05142_3
MDA-MB-346	SP1	path:05145_8
MDA-MB-346	SP1	path:05145_6
MDA-MB-346	SP1	path:05146_1
MDA-MB-346	SP1	path:05200_7
MDA-MB-346	SP1	path:05200_2
MDA-MB-346	SP1	path:05200_21
MDA-MB-346	SP1	path:05200_10
MDA-MB-346	SP1	path:05200_31

MDA-MB-346	SP1	path:05200_19
MDA-MB-346	SP1	path:05200_39
MDA-MB-346	SP1	path:05200_1
MDA-MB-346	SP1	path:05200_35
MDA-MB-346	SP1	path:05200_6
MDA-MB-346	SP1	path:05200_38
MDA-MB-346	SP1	path:05200_8
MDA-MB-346	SP1	path:05200_9
MDA-MB-346	SP1	path:05200_37
MDA-MB-346	SP1	path:05200_24
MDA-MB-346	SP1	path:05200_32
MDA-MB-346	SP1	path:05200_42
MDA-MB-346	SP1	path:05200_34
MDA-MB-346	SP1	path:05200_20
MDA-MB-346	SP1	path:05200_18
MDA-MB-346	SP1	path:05200_16
MDA-MB-346	SP1	path:05200_25
MDA-MB-346	SP1	path:05200_13
MDA-MB-346	SP1	path:05200_41
MDA-MB-346	SP1	path:05200_29
MDA-MB-346	SP1	path:05200_14
MDA-MB-346	SP1	path:05200_15
MDA-MB-346	SP1	path:05200_26
MDA-MB-346	SP1	path:05200_17
MDA-MB-346	SP1	path:05200_30
MDA-MB-346	SP1	path:05200_33
MDA-MB-346	SP1	path:05210_8
MDA-MB-346	SP1	path:05210_5
MDA-MB-346	SP1	path:05212_3
MDA-MB-346	SP1	path:05212_10
MDA-MB-346	SP1	path:05212_8
MDA-MB-346	SP1	path:05212_9
MDA-MB-346	SP1	path:05214_1
MDA-MB-346	SP1	path:05214_3
MDA-MB-346	SP1	path:05214_2
MDA-MB-346	SP1	path:05215_9
MDA-MB-346	SP1	path:05215_10
MDA-MB-346	SP1	path:05215_5
MDA-MB-346	SP1	path:05215_7
MDA-MB-346	SP1	path:05215_6
MDA-MB-346	SP1	path:05215_4
MDA-MB-346	SP1	path:05216_3
MDA-MB-346	SP1	path:05218_3
MDA-MB-346	SP1	path:05218_1

MDA-MB-346	SP1	path:05218_2
MDA-MB-346	SP1	path:05218_4
MDA-MB-346	SP1	path:05218_5
MDA-MB-346	SP1	path:05219_5
MDA-MB-346	SP1	path:05219_3
MDA-MB-346	SP1	path:05220_4
MDA-MB-346	SP1	path:05220_8
MDA-MB-346	SP1	path:05220_5
MDA-MB-346	SP1	path:05221_2
MDA-MB-346	SP1	path:05222_5
MDA-MB-346	SP1	path:05222_2
MDA-MB-346	SP1	path:05222_1
MDA-MB-346	SP1	path:05223_3
MDA-MB-346	SP1	path:05223_1
MDA-MB-346	SP1	path:05320_2
MDA-MB-346	SP1	path:05330_2
MDA-MB-346	SP1	path:05332_1
MDA-MB-346	SP1	path:05414_2
MDA-MB-361	STAT3	path:04012_4
MDA-MB-361	STAT3	path:04060_1
MDA-MB-361	STAT3	path:04060_34
MDA-MB-361	STAT3	path:04630_1
MDA-MB-361	STAT3	path:04630_2
MDA-MB-361	STAT3	path:05142_3
MDA-MB-361	STAT3	path:05200_39
MDA-MB-361	STAT3	path:05200_35
MDA-MB-361	STAT3	path:05200_21
MDA-MB-361	STAT3	path:05200_30
MDA-MB-361	STAT3	path:05200_25
MDA-MB-361	STAT3	path:05200_15
MDA-MB-361	STAT3	path:05200_16
MDA-MB-361	STAT3	path:05200_26
MDA-MB-361	STAT3	path:05200_37
MDA-MB-361	STAT3	path:05200_24
MDA-MB-361	STAT3	path:05200_18
MDA-MB-361	STAT3	path:05200_38
MDA-MB-361	STAT3	path:05210_8
MDA-MB-361	STAT3	path:05210_5
MDA-MB-361	STAT3	path:05218_4
MDA-MB-361	STAT3	path:05221_1
MDA-MB-435	STAT3	path:04012_4
MDA-MB-435	STAT3	path:04060_34
MDA-MB-435	STAT3	path:04060_1
MDA-MB-435	STAT3	path:04630_1

**MDA-MB-435**	**STAT3**	**path:04630_2**
**MDA-MB-435**	**STAT3**	**path:05142_3**
**MDA-MB-435**	**STAT3**	**path:05200_16**
**MDA-MB-435**	**STAT3**	**path:05200_21**
**MDA-MB-435**	**STAT3**	**path:05200_24**
**MDA-MB-435**	**STAT3**	**path:05200_25**
**MDA-MB-435**	**STAT3**	**path:05200_37**
**MDA-MB-435**	**STAT3**	**path:05200_15**
**MDA-MB-435**	**STAT3**	**path:05200_35**
**MDA-MB-435**	**STAT3**	**path:05200_18**
**MDA-MB-435**	**STAT3**	**path:05200_26**
**MDA-MB-435**	**STAT3**	**path:05200_38**
**MDA-MB-435**	**STAT3**	**path:05200_30**
**MDA-MB-435**	**STAT3**	**path:05200_39**
**MDA-MB-435**	**STAT3**	**path:05210_8**
**MDA-MB-435**	**STAT3**	**path:05210_5**
**MDA-MB-435**	**STAT3**	**path:05218_4**
**MDA-MB-435**	**STAT3**	**path:05221_1**
**MDA-MB-453**	**STAT3**	**path:04012_4**
**MDA-MB-453**	**STAT3**	**path:04060_34**
**MDA-MB-453**	**STAT3**	**path:04060_1**
**MDA-MB-453**	**STAT3**	**path:04630_2**
**MDA-MB-453**	**STAT3**	**path:04630_1**
**MDA-MB-453**	**STAT3**	**path:05142_3**
**MDA-MB-453**	**STAT3**	**path:05200_18**
**MDA-MB-453**	**STAT3**	**path:05200_24**
**MDA-MB-453**	**STAT3**	**path:05200_16**
**MDA-MB-453**	**STAT3**	**path:05200_21**
**MDA-MB-453**	**STAT3**	**path:05200_25**
**MDA-MB-453**	**STAT3**	**path:05200_37**
**MDA-MB-453**	**STAT3**	**path:05200_15**
**MDA-MB-453**	**STAT3**	**path:05200_38**
**MDA-MB-453**	**STAT3**	**path:05200_30**
**MDA-MB-453**	**STAT3**	**path:05200_26**
**MDA-MB-453**	**STAT3**	**path:05200_35**
**MDA-MB-453**	**STAT3**	**path:05200_39**
**MDA-MB-453**	**STAT3**	**path:05210_8**
**MDA-MB-453**	**STAT3**	**path:05210_5**
**MDA-MB-453**	**STAT3**	**path:05218_4**
**MDA-MB-453**	**STAT3**	**path:05221_1**
**MDA-MB-468**	**E2F1**	**path:04110_2**
**MDA-MB-468**	**E2F1**	**path:04110_10**
**MDA-MB-468**	**E2F1**	**path:04110_4**
**MDA-MB-468**	**E2F1**	**path:04110_13**

**MDA-MB-468**	**E2F1**	**path:04110_1**
**MDA-MB-468**	**E2F1**	**path:04110_9**
**MDA-MB-468**	**E2F1**	**path:04110_5**
**MDA-MB-468**	**E2F1**	**path:04110_7**
**MDA-MB-468**	**E2F1**	**path:04110_11**
**MDA-MB-468**	**E2F1**	**path:04110_6**
**MDA-MB-468**	**E2F1**	**path:04115_2**
**MDA-MB-468**	**E2F1**	**path:04210_5**
**MDA-MB-468**	**E2F1**	**path:04510_10**
**MDA-MB-468**	**E2F1**	**path:05014_2**
**MDA-MB-468**	**E2F1**	**path:05200_37**
**MDA-MB-468**	**E2F1**	**path:05200_25**
**MDA-MB-468**	**E2F1**	**path:05200_26**
**MDA-MB-468**	**E2F1**	**path:05200_2**
**MDA-MB-468**	**E2F1**	**path:05200_15**
**MDA-MB-468**	**E2F1**	**path:05200_18**
**MDA-MB-468**	**E2F1**	**path:05200_33**
**MDA-MB-468**	**E2F1**	**path:05200_35**
**MDA-MB-468**	**E2F1**	**path:05200_21**
**MDA-MB-468**	**E2F1**	**path:05200_13**
**MDA-MB-468**	**E2F1**	**path:05200_38**
**MDA-MB-468**	**E2F1**	**path:05200_39**
**MDA-MB-468**	**E2F1**	**path:05212_9**
**MDA-MB-468**	**E2F1**	**path:05214_3**
**MDA-MB-468**	**E2F1**	**path:05215_3**
**MDA-MB-468**	**E2F1**	**path:05218_5**
**MDA-MB-468**	**E2F1**	**path:05218_3**
**MDA-MB-468**	**E2F1**	**path:05219_5**
**MDA-MB-468**	**E2F1**	**path:05219_3**
**MDA-MB-468**	**E2F1**	**path:05220_4**
**MDA-MB-468**	**E2F1**	**path:05222_1**
**MDA-MB-468**	**E2F1**	**path:05222_5**
**MDA-MB-468**	**E2F1**	**path:05223_3**
**MDA-MB-468**	**E2F1**	**path:05223_4**
**MDA-MB-468**	**STAT3**	**path:04012_4**
**MDA-MB-468**	**STAT3**	**path:04060_1**
**MDA-MB-468**	**STAT3**	**path:04060_34**
**MDA-MB-468**	**STAT3**	**path:04630_2**
**MDA-MB-468**	**STAT3**	**path:04630_1**
**MDA-MB-468**	**STAT3**	**path:05142_3**
**MDA-MB-468**	**STAT3**	**path:05200_30**
**MDA-MB-468**	**STAT3**	**path:05200_35**
**MDA-MB-468**	**STAT3**	**path:05200_39**
**MDA-MB-468**	**STAT3**	**path:05200_25**

**MDA-MB-468**	**STAT3**	**path:05200_15**
**MDA-MB-468**	**STAT3**	**path:05200_21**
**MDA-MB-468**	**STAT3**	**path:05200_37**
**MDA-MB-468**	**STAT3**	**path:05200_24**
**MDA-MB-468**	**STAT3**	**path:05200_16**
**MDA-MB-468**	**STAT3**	**path:05200_26**
**MDA-MB-468**	**STAT3**	**path:05200_18**
**MDA-MB-468**	**STAT3**	**path:05200_38**
**MDA-MB-468**	**STAT3**	**path:05210_5**
**MDA-MB-468**	**STAT3**	**path:05210_8**
**MDA-MB-468**	**STAT3**	**path:05218_4**
**MDA-MB-468**	**STAT3**	**path:05221_1**
**melanoma**	**GLI1**	**path:04510_10**
**melanoma**	**GLI1**	**path:04510_8**
**muscle**	**FOXO3**	**path:04920_2**
**muscle**	**NR1I3**	**path:00830_1**
**muscle**	**NR1I3**	**path:00980_1**
**muscle**	**NR1I3**	**path:00982_2**
**muscle**	**NR4A1**	**path:00140_1**
**muscle**	**SREBF1**	**path:04910_1**
**muscle**	**SREBF1**	**path:04910_4**
**myoblasts**	**MYOD1**	**path:05200_39**
**myoblasts**	**MYOD1**	**path:05200_25**
**myoblasts**	**MYOD1**	**path:05200_15**
**myoblasts**	**MYOD1**	**path:05200_24**
**myoblasts**	**MYOD1**	**path:05200_38**
**myoblasts**	**MYOD1**	**path:05200_18**
**NBE**	**E2F1**	**path:04110_2**
**NBE**	**E2F1**	**path:04110_4**
**NBE**	**E2F1**	**path:04110_13**
**NBE**	**E2F1**	**path:04110_10**
**NBE**	**E2F1**	**path:04110_1**
**NBE**	**E2F1**	**path:04110_5**
**NBE**	**E2F1**	**path:04110_7**
**NBE**	**E2F1**	**path:04110_9**
**NBE**	**E2F1**	**path:04110_11**
**NBE**	**E2F1**	**path:04110_6**
**NBE**	**E2F1**	**path:04115_2**
**NBE**	**E2F1**	**path:04210_5**
**NBE**	**E2F1**	**path:04510_10**
**NBE**	**E2F1**	**path:05014_2**
**NBE**	**E2F1**	**path:05200_25**
**NBE**	**E2F1**	**path:05200_37**
**NBE**	**E2F1**	**path:05200_26**

NBE	E2F1	path:05200_39
NBE	E2F1	path:05200_15
NBE	E2F1	path:05200_2
NBE	E2F1	path:05200_33
NBE	E2F1	path:05200_35
NBE	E2F1	path:05200_38
NBE	E2F1	path:05200_13
NBE	E2F1	path:05200_18
NBE	E2F1	path:05200_21
NBE	E2F1	path:05212_9
NBE	E2F1	path:05214_3
NBE	E2F1	path:05215_3
NBE	E2F1	path:05218_5
NBE	E2F1	path:05218_3
NBE	E2F1	path:05219_3
NBE	E2F1	path:05219_5
NBE	E2F1	path:05220_4
NBE	E2F1	path:05222_1
NBE	E2F1	path:05222_5
NBE	E2F1	path:05223_4
NBE	E2F1	path:05223_3
neuroblastoma	GATA3	path:04612_4
neuroblastoma	HIF1A	path:05200_25
neuroblastoma	HIF1A	path:05200_39
neuroblastoma	HIF1A	path:05200_37
neuroblastoma	TP73	path:04115_2
neuroblastoma	TP73	path:04722_15
neuroblastoma	TP73	path:05200_33
nucleus	TCF7	path:04660_15
ovary	AKNA	path:04060_18
ovary	AKNA	path:04514_8
ovary	AKNA	path:04672_2
ovary	AKNA	path:05144_4
ovary	AKNA	path:05145_14
ovary	AKNA	path:05310_1
ovary	AKNA	path:05320_1
ovary	AKNA	path:05322_3
ovary	AKNA	path:05330_1
ovary	AKNA	path:05416_3
ovary	FLI1	path:04512_3
ovary	FLI1	path:04512_1
ovary	FOXO3	path:04920_2
pancreas	ELF1	path:00920_1
pancreas	ELF1	path:04060_34

pancreas	ELF1	path:04060_1
pancreas	ELF1	path:04650_2
pancreas	ELF1	path:04650_8
pancreas	ELF1	path:04650_10
pancreas	ELF1	path:04650_9
pancreas	ELF1	path:04650_4
pancreas	ELF1	path:04664_1
pancreas	ELF1	path:04664_6
pancreas	ELF1	path:04664_2
pancreas	FOXA3	path:00140_1
pancreas	FOXA3	path:00591_1
pancreas	FOXA3	path:00830_1
pancreas	FOXA3	path:00980_1
pancreas	FOXA3	path:00982_2
pancreas	FOXO3	path:04920_2
pancreas	HNF4A	path:00010_1
pancreas	HNF4A	path:00120_1
pancreas	HNF4A	path:00140_1
pancreas	HNF4A	path:00561_2
pancreas	HNF4A	path:00590_1
pancreas	HNF4A	path:00590_2
pancreas	HNF4A	path:00591_1
pancreas	HNF4A	path:00830_1
pancreas	HNF4A	path:00980_1
pancreas	HNF4A	path:00982_2
pancreas	HNF4A	path:03320_1
pancreas	HNF4A	path:04610_2
pancreas	HNF4A	path:04610_6
pancreas	HNF4A	path:04610_5
pancreas	HNF4A	path:04610_1
pancreas	HNF4A	path:04610_3
pancreas	HNF4A	path:04610_7
pancreas	HNF4A	path:04610_4
pancreas	HNF4A	path:04950_2
pancreas	HNF4A	path:04950_3
peripheral blood	AKNA	path:04060_18
peripheral blood	AKNA	path:04514_8
peripheral blood	AKNA	path:04672_2
peripheral blood	AKNA	path:05144_4
peripheral blood	AKNA	path:05145_14
peripheral blood	AKNA	path:05310_1
peripheral blood	AKNA	path:05320_1
peripheral blood	AKNA	path:05322_3
peripheral blood	AKNA	path:05330_1

**peripheral blood**	**AKNA**	**path:05416_3**
**peripheral blood**	**IRF5**	**path:04060_23**
**peripheral blood**	**IRF5**	**path:04620_1**
**peripheral blood**	**IRF5**	**path:04620_3**
**peripheral blood**	**IRF5**	**path:04620_2**
**peripheral blood**	**IRF5**	**path:04620_4**
**peripheral blood**	**IRF5**	**path:04620_7**
**peripheral blood**	**IRF5**	**path:04650_5**
**peripheral blood**	**IRF5**	**path:04650_3**
**peripheral blood**	**IRF5**	**path:05160_4**
**peripheral blood**	**IRF7**	**path:04060_23**
**peripheral blood**	**IRF7**	**path:04620_4**
**peripheral blood**	**IRF7**	**path:04620_8**
**peripheral blood**	**IRF7**	**path:04620_7**
**peripheral blood**	**IRF7**	**path:04620_6**
**peripheral blood**	**IRF7**	**path:04620_2**
**peripheral blood**	**IRF7**	**path:04620_9**
**peripheral blood**	**IRF7**	**path:04620_1**
**peripheral blood**	**IRF7**	**path:04620_3**
**peripheral blood**	**IRF7**	**path:04620_5**
**peripheral blood**	**IRF7**	**path:04650_5**
**peripheral blood**	**IRF7**	**path:04650_3**
**peripheral blood**	**IRF7**	**path:05160_4**
**pituitary**	**NR5A1**	**path:00140_1**
**pituitary**	**NR5A1**	**path:04060_2**
**pituitary**	**NR5A1**	**path:04350_6**
**placenta**	**FOXO3**	**path:04920_2**
**placenta**	**HIF1A**	**path:05200_25**
**placenta**	**HIF1A**	**path:05200_39**
**placenta**	**HIF1A**	**path:05200_37**
**placenta**	**KLF5**	**path:05200_35**
**placenta**	**KLF5**	**path:05200_25**
**placenta**	**KLF5**	**path:05200_37**
**placenta**	**KLF5**	**path:05200_21**
**placenta**	**KLF5**	**path:05210_5**
**placenta**	**KLF5**	**path:05210_8**
**placenta**	**SREBF1**	**path:04910_4**
**placenta**	**SREBF1**	**path:04910_1**
**placenta**	**TP63**	**path:04110_10**
**placenta**	**TP63**	**path:04110_11**
**placenta**	**TP63**	**path:04110_5**
**placenta**	**TP63**	**path:04110_1**
**placenta**	**TP63**	**path:04110_7**
**placenta**	**TP63**	**path:04110_2**

**placenta**	**TP63**	**path:04110_9**
**placenta**	**TP63**	**path:04115_2**
**placenta**	**TP63**	**path:05200_15**
**placenta**	**TP63**	**path:05200_21**
**placenta**	**TP63**	**path:05200_7**
**placenta**	**TP63**	**path:05200_2**
**placenta**	**TP63**	**path:05200_39**
**placenta**	**TP63**	**path:05200_25**
**placenta**	**TP63**	**path:05200_9**
**placenta**	**TP63**	**path:05200_13**
**placenta**	**TP63**	**path:05200_14**
**placenta**	**TP63**	**path:05200_3**
**placenta**	**TP63**	**path:05200_1**
**placenta**	**TP63**	**path:05200_19**
**placenta**	**TP63**	**path:05200_20**
**placenta**	**TP63**	**path:05200_37**
**placenta**	**TP63**	**path:05214_3**
**placenta**	**TP63**	**path:05215_4**
**placenta**	**TP63**	**path:05215_10**
**placenta**	**TP63**	**path:05215_9**
**placenta**	**TP63**	**path:05215_6**
**placenta**	**TP63**	**path:05218_3**
**placenta**	**TP63**	**path:05218_5**
**placenta**	**TP63**	**path:05219_5**
**placenta**	**TP63**	**path:05219_3**
**placenta**	**TP63**	**path:05220_4**
**PMA**	**EGR1**	**path:04144_2**
**PMA**	**EGR1**	**path:04350_5**
**PMA**	**EGR1**	**path:04350_1**
**PMA**	**EGR1**	**path:04510_7**
**PMA**	**EGR1**	**path:04510_13**
**PMA**	**EGR1**	**path:04510_12**
**PMA**	**EGR1**	**path:04510_5**
**PMA**	**EGR1**	**path:04510_6**
**PMA**	**EGR1**	**path:04512_3**
**PMA**	**EGR1**	**path:04512_2**
**PMA**	**EGR1**	**path:04512_1**
**PMA**	**EGR1**	**path:05020_2**
**PMA**	**EGR1**	**path:05200_16**
**PMA**	**EGR1**	**path:05200_24**
**PMA**	**EGR1**	**path:05200_35**
**PMA**	**EGR1**	**path:05200_29**
**PMA**	**EGR1**	**path:05200_31**
**PMA**	**EGR1**	**path:05200_30**

**PMA**	**EGR1**	**path:05200_25**
**PMA**	**EGR1**	**path:05200_37**
**PMA**	**EGR1**	**path:05212_3**
**PMA**	**EGR1**	**path:05212_8**
**PMA**	**EGR1**	**path:05215_7**
**PMA**	**EGR1**	**path:05215_9**
**PMA**	**EGR1**	**path:05218_4**
**PMA**	**FOS**	**path:04115_2**
**PMA**	**FOS**	**path:04310_7**
**PMA**	**FOS**	**path:04610_6**
**PMA**	**FOS**	**path:04610_7**
**PMA**	**FOS**	**path:04620_6**
**PMA**	**FOS**	**path:04620_9**
**PMA**	**FOS**	**path:04620_8**
**PMA**	**FOS**	**path:04620_5**
**PMA**	**FOS**	**path:04670_2**
**PMA**	**FOS**	**path:05200_24**
**PMA**	**FOS**	**path:05200_38**
**PMA**	**FOS**	**path:05200_39**
**PMA**	**FOS**	**path:05200_37**
**PMA**	**FOS**	**path:05200_25**
**PMA**	**FOS**	**path:05200_35**
**PMA**	**FOS**	**path:05219_3**
**PMA**	**RELA**	**path:04060_44**
**PMA**	**RELA**	**path:04060_35**
**PMA**	**RELA**	**path:04062_1**
**PMA**	**RELA**	**path:04062_2**
**PMA**	**RELA**	**path:04110_10**
**PMA**	**RELA**	**path:04110_2**
**PMA**	**RELA**	**path:04110_1**
**PMA**	**RELA**	**path:04110_9**
**PMA**	**RELA**	**path:04110_7**
**PMA**	**RELA**	**path:04110_11**
**PMA**	**RELA**	**path:04110_5**
**PMA**	**RELA**	**path:04115_2**
**PMA**	**RELA**	**path:04612_5**
**PMA**	**RELA**	**path:04620_5**
**PMA**	**RELA**	**path:04620_8**
**PMA**	**RELA**	**path:04620_1**
**PMA**	**RELA**	**path:04620_9**
**PMA**	**RELA**	**path:04620_4**
**PMA**	**RELA**	**path:04620_3**
**PMA**	**RELA**	**path:04620_7**
**PMA**	**RELA**	**path:04620_6**

PMA	RELA	path:04650_13
PMA	RELA	path:04650_3
PMA	RELA	path:04650_5
PMA	RELA	path:04650_14
PMA	RELA	path:04650_15
PMA	RELA	path:04670_8
PMA	RELA	path:04670_6
PMA	RELA	path:04670_4
PMA	RELA	path:04670_9
PMA	RELA	path:04670_2
PMA	RELA	path:05142_3
PMA	RELA	path:05200_35
PMA	RELA	path:05200_39
PMA	RELA	path:05200_37
PMA	RELA	path:05200_15
PMA	RELA	path:05200_25
PMA	RELA	path:05200_13
PMA	RELA	path:05200_2
PMA	RELA	path:05200_21
PMA	RELA	path:05200_38
PMA	RELA	path:05219_3
prostate	HNF1A	path:00140_1
prostate	HNF1A	path:00980_1
prostate	HNF1A	path:00982_2
prostate	HNF1A	path:04610_2
prostate	HNF1A	path:04610_3
prostate	HNF1A	path:04610_4
prostate	HNF1A	path:04610_6
prostate	HNF1A	path:04950_3
prostate	TP63	path:04110_10
prostate	TP63	path:04110_11
prostate	TP63	path:04110_5
prostate	TP63	path:04110_2
prostate	TP63	path:04110_1
prostate	TP63	path:04110_7
prostate	TP63	path:04110_9
prostate	TP63	path:04115_2
prostate	TP63	path:05200_15
prostate	TP63	path:05200_2
prostate	TP63	path:05200_21
prostate	TP63	path:05200_39
prostate	TP63	path:05200_7
prostate	TP63	path:05200_14
prostate	TP63	path:05200_9

prostate	TP63	path:05200_37
prostate	TP63	path:05200_20
prostate	TP63	path:05200_3
prostate	TP63	path:05200_1
prostate	TP63	path:05200_13
prostate	TP63	path:05200_19
prostate	TP63	path:05200_25
prostate	TP63	path:05214_3
prostate	TP63	path:05215_4
prostate	TP63	path:05215_10
prostate	TP63	path:05215_9
prostate	TP63	path:05215_6
prostate	TP63	path:05218_3
prostate	TP63	path:05218_5
prostate	TP63	path:05219_5
prostate	TP63	path:05219_3
prostate	TP63	path:05220_4
retina	NRL	path:04744_2
salivary gland	TP63	path:04110_11
salivary gland	TP63	path:04110_10
salivary gland	TP63	path:04110_7
salivary gland	TP63	path:04110_5
salivary gland	TP63	path:04110_2
salivary gland	TP63	path:04110_1
salivary gland	TP63	path:04110_9
salivary gland	TP63	path:04115_2
salivary gland	TP63	path:05200_21
salivary gland	TP63	path:05200_15
salivary gland	TP63	path:05200_9
salivary gland	TP63	path:05200_39
salivary gland	TP63	path:05200_7
salivary gland	TP63	path:05200_25
salivary gland	TP63	path:05200_2
salivary gland	TP63	path:05200_14
salivary gland	TP63	path:05200_3
salivary gland	TP63	path:05200_13
salivary gland	TP63	path:05200_1
salivary gland	TP63	path:05200_20
salivary gland	TP63	path:05200_19
salivary gland	TP63	path:05200_37
salivary gland	TP63	path:05214_3
salivary gland	TP63	path:05215_4
salivary gland	TP63	path:05215_6
salivary gland	TP63	path:05215_10

**salivary gland**	**TP63**	**path:05215_9**
**salivary gland**	**TP63**	**path:05218_5**
**salivary gland**	**TP63**	**path:05218_3**
**salivary gland**	**TP63**	**path:05219_5**
**salivary gland**	**TP63**	**path:05219_3**
**salivary gland**	**TP63**	**path:05220_4**
**Saos**	**ELK1**	**path:04010_4**
**Saos**	**ELK1**	**path:04010_11**
**Saos**	**ELK1**	**path:05020_3**
**Saos**	**ELK1**	**path:05020_4**
**Saos**	**ELK1**	**path:05110_3**
**Saos**	**ELK1**	**path:05200_18**
**Saos**	**ELK1**	**path:05200_26**
**Saos**	**MYOD1**	**path:05200_25**
**Saos**	**MYOD1**	**path:05200_18**
**Saos**	**MYOD1**	**path:05200_15**
**Saos**	**MYOD1**	**path:05200_39**
**Saos**	**MYOD1**	**path:05200_24**
**Saos**	**MYOD1**	**path:05200_38**
**skeletal**	**BCL6**	**path:04110_5**
**skeletal**	**BCL6**	**path:04110_11**
**skeletal**	**BCL6**	**path:04110_13**
**skeletal**	**BCL6**	**path:04110_7**
**skeletal**	**BCL6**	**path:04110_2**
**skeletal**	**BCL6**	**path:04110_10**
**skeletal**	**BCL6**	**path:04110_1**
**skeletal**	**BCL6**	**path:04110_9**
**skeletal**	**BCL6**	**path:04115_2**
**skeletal**	**BCL6**	**path:04210_3**
**skeletal**	**BCL6**	**path:04210_6**
**skeletal**	**BCL6**	**path:04630_1**
**skeletal**	**BCL6**	**path:05200_13**
**skeletal**	**BCL6**	**path:05200_25**
**skeletal**	**BCL6**	**path:05200_39**
**skeletal**	**BCL6**	**path:05200_37**
**skeletal**	**BCL6**	**path:05200_2**
**skeletal**	**BCL6**	**path:05200_15**
**skeletal**	**BCL6**	**path:05200_21**
**skeletal**	**BCL6**	**path:05220_8**
**skeletal**	**FOXO3**	**path:04920_2**
**skeletal**	**TP63**	**path:04110_10**
**skeletal**	**TP63**	**path:04110_5**
**skeletal**	**TP63**	**path:04110_11**
**skeletal**	**TP63**	**path:04110_1**

skeletal	TP63	path:04110_7
skeletal	TP63	path:04110_9
skeletal	TP63	path:04110_2
skeletal	TP63	path:04115_2
skeletal	TP63	path:05200_15
skeletal	TP63	path:05200_21
skeletal	TP63	path:05200_7
skeletal	TP63	path:05200_14
skeletal	TP63	path:05200_20
skeletal	TP63	path:05200_13
skeletal	TP63	path:05200_39
skeletal	TP63	path:05200_37
skeletal	TP63	path:05200_9
skeletal	TP63	path:05200_1
skeletal	TP63	path:05200_2
skeletal	TP63	path:05200_3
skeletal	TP63	path:05200_25
skeletal	TP63	path:05200_19
skeletal	TP63	path:05214_3
skeletal	TP63	path:05215_4
skeletal	TP63	path:05215_10
skeletal	TP63	path:05215_9
skeletal	TP63	path:05215_6
skeletal	TP63	path:05218_3
skeletal	TP63	path:05218_5
skeletal	TP63	path:05219_5
skeletal	TP63	path:05219_3
skeletal	TP63	path:05220_4
small intestine	HNF4A	path:00010_1
small intestine	HNF4A	path:00120_1
small intestine	HNF4A	path:00140_1
small intestine	HNF4A	path:00561_2
small intestine	HNF4A	path:00590_1
small intestine	HNF4A	path:00590_2
small intestine	HNF4A	path:00591_1
small intestine	HNF4A	path:00830_1
small intestine	HNF4A	path:00980_1
small intestine	HNF4A	path:00982_2
small intestine	HNF4A	path:03320_1
small intestine	HNF4A	path:04610_7
small intestine	HNF4A	path:04610_6
small intestine	HNF4A	path:04610_2
small intestine	HNF4A	path:04610_1
small intestine	HNF4A	path:04610_3

**small intestine**	**HNF4A**	**path:04610_5**
**small intestine**	**HNF4A**	**path:04610_4**
**small intestine**	**HNF4A**	**path:04950_3**
**small intestine**	**HNF4A**	**path:04950_2**
**small intestine**	**NR1I2**	**path:00830_1**
**small intestine**	**NR1I2**	**path:00980_1**
**small intestine**	**NR1I2**	**path:00982_2**
**smooth**	**KLF5**	**path:05200_35**
**smooth**	**KLF5**	**path:05200_25**
**smooth**	**KLF5**	**path:05200_21**
**smooth**	**KLF5**	**path:05200_37**
**smooth**	**KLF5**	**path:05210_5**
**smooth**	**KLF5**	**path:05210_8**
**spleen**	**AKNA**	**path:04060_18**
**spleen**	**AKNA**	**path:04514_8**
**spleen**	**AKNA**	**path:04672_2**
**spleen**	**AKNA**	**path:05144_4**
**spleen**	**AKNA**	**path:05145_14**
**spleen**	**AKNA**	**path:05310_1**
**spleen**	**AKNA**	**path:05320_1**
**spleen**	**AKNA**	**path:05322_3**
**spleen**	**AKNA**	**path:05330_1**
**spleen**	**AKNA**	**path:05416_3**
**spleen**	**CREB1**	**path:04514_19**
**spleen**	**CREB1**	**path:04612_3**
**spleen**	**CREB1**	**path:04620_9**
**spleen**	**CREB1**	**path:04620_6**
**spleen**	**CREB1**	**path:04620_8**
**spleen**	**CREB1**	**path:04620_4**
**spleen**	**CREB1**	**path:04620_5**
**spleen**	**CREB1**	**path:05142_3**
**spleen**	**ELF1**	**path:00920_1**
**spleen**	**ELF1**	**path:04060_34**
**spleen**	**ELF1**	**path:04060_1**
**spleen**	**ELF1**	**path:04650_10**
**spleen**	**ELF1**	**path:04650_9**
**spleen**	**ELF1**	**path:04650_2**
**spleen**	**ELF1**	**path:04650_8**
**spleen**	**ELF1**	**path:04650_4**
**spleen**	**ELF1**	**path:04664_1**
**spleen**	**ELF1**	**path:04664_2**
**spleen**	**ELF1**	**path:04664_6**
**spleen**	**FLI1**	**path:04512_1**
**spleen**	**FLI1**	**path:04512_3**

**spleen**	**HNF1A**	**path:00140_1**
**spleen**	**HNF1A**	**path:00980_1**
**spleen**	**HNF1A**	**path:00982_2**
**spleen**	**HNF1A**	**path:04610_2**
**spleen**	**HNF1A**	**path:04610_6**
**spleen**	**HNF1A**	**path:04610_3**
**spleen**	**HNF1A**	**path:04610_4**
**spleen**	**HNF1A**	**path:04950_3**
**spleen**	**IRF5**	**path:04060_23**
**spleen**	**IRF5**	**path:04620_1**
**spleen**	**IRF5**	**path:04620_3**
**spleen**	**IRF5**	**path:04620_2**
**spleen**	**IRF5**	**path:04620_7**
**spleen**	**IRF5**	**path:04620_4**
**spleen**	**IRF5**	**path:04650_5**
**spleen**	**IRF5**	**path:04650_3**
**spleen**	**IRF5**	**path:05160_4**
**spleen**	**IRF7**	**path:04060_23**
**spleen**	**IRF7**	**path:04620_8**
**spleen**	**IRF7**	**path:04620_7**
**spleen**	**IRF7**	**path:04620_6**
**spleen**	**IRF7**	**path:04620_4**
**spleen**	**IRF7**	**path:04620_2**
**spleen**	**IRF7**	**path:04620_1**
**spleen**	**IRF7**	**path:04620_3**
**spleen**	**IRF7**	**path:04620_9**
**spleen**	**IRF7**	**path:04620_5**
**spleen**	**IRF7**	**path:04650_5**
**spleen**	**IRF7**	**path:04650_3**
**spleen**	**IRF7**	**path:05160_4**
**spleen**	**SPI1**	**path:04060_26**
**spleen**	**SPI1**	**path:04145_1**
**spleen**	**SPI1**	**path:04145_4**
**spleen**	**SPI1**	**path:04620_4**
**spleen**	**SPI1**	**path:04620_3**
**spleen**	**SPI1**	**path:04620_1**
**spleen**	**SPI1**	**path:04650_7**
**spleen**	**SPI1**	**path:04650_1**
**spleen**	**SPI1**	**path:04670_9**
**spleen**	**SPI1**	**path:04670_6**
**spleen**	**SPI1**	**path:04670_4**
**spleen**	**SPI1**	**path:04670_2**
**spleen**	**SPI1**	**path:04670_8**
**spleen**	**SPI1**	**path:04810_16**

**spleen**	**SPI1**	**path:05140_3**
**spleen**	**SPI1**	**path:05140_2**
**spleen**	**SPI1**	**path:05146_1**
**spleen**	**SPI1**	**path:05150_4**
**spleen**	**SPI1**	**path:05200_34**
**stomach**	**HNF1A**	**path:00140_1**
**stomach**	**HNF1A**	**path:00980_1**
**stomach**	**HNF1A**	**path:00982_2**
**stomach**	**HNF1A**	**path:04610_6**
**stomach**	**HNF1A**	**path:04610_2**
**stomach**	**HNF1A**	**path:04610_3**
**stomach**	**HNF1A**	**path:04610_4**
**stomach**	**HNF1A**	**path:04950_3**
**T cells**	**AKNA**	**path:04060_18**
**T cells**	**AKNA**	**path:04514_8**
**T cells**	**AKNA**	**path:04672_2**
**T cells**	**AKNA**	**path:05144_4**
**T cells**	**AKNA**	**path:05145_14**
**T cells**	**AKNA**	**path:05310_1**
**T cells**	**AKNA**	**path:05320_1**
**T cells**	**AKNA**	**path:05322_3**
**T cells**	**AKNA**	**path:05330_1**
**T cells**	**AKNA**	**path:05416_3**
**T cells**	**CREM**	**path:05200_37**
**T cells**	**CREM**	**path:05200_35**
**T cells**	**CREM**	**path:05200_25**
**T cells**	**CREM**	**path:05200_38**
**T cells**	**CREM**	**path:05200_39**
**T cells**	**CREM**	**path:05210_8**
**T cells**	**CREM**	**path:05210_5**
**T cells**	**ETS2**	**path:04310_5**
**T cells**	**ETS2**	**path:04310_7**
**T cells**	**ETS2**	**path:05200_19**
**T cells**	**ETS2**	**path:05200_9**
**T cells**	**ETS2**	**path:05200_17**
**T cells**	**ETS2**	**path:05200_2**
**T cells**	**FOXO3**	**path:04920_2**
**T cells**	**GATA3**	**path:04612_4**
**T cells**	**TCF7**	**path:04660_15**
**T47D**	**ESR1**	**path:00830_1**
**T47D**	**ESR1**	**path:00980_1**
**T47D**	**ESR1**	**path:00982_2**
**T47D**	**ESR1**	**path:04115_2**
**T47D**	**ESR1**	**path:04610_7**

T47D	ESR1	path:04610_2
T47D	ESR1	path:04610_6
T47D	ESR1	path:05200_37
T47D	ESR1	path:05200_30
T47D	ESR1	path:05200_25
T47D	ESR1	path:05200_21
T47D	ESR1	path:05200_38
T47D	ESR1	path:05200_15
T47D	ESR1	path:05200_35
T47D	ESR1	path:05200_24
T47D	ESR1	path:05200_39
T47D	ESR1	path:05212_8
T47D	ESR1	path:05219_3
T47D	NFIC	path:00140_1
T47D	NFIC	path:00591_1
T47D	NFIC	path:04010_22
T47D	NFIC	path:04080_5
T47D	NFIC	path:04115_2
T47D	NFIC	path:04510_6
T47D	NFIC	path:04512_2
T47D	NFIC	path:04660_10
T47D	NFIC	path:04660_9
T47D	NFIC	path:04662_9
T47D	NFIC	path:04662_5
T47D	NFIC	path:04722_15
T47D	NFIC	path:05200_7
T47D	NFIC	path:05200_2
T47D	NFIC	path:05200_13
T47D	NFIC	path:05200_24
T47D	NFIC	path:05200_6
T47D	NFIC	path:05200_25
T47D	NFIC	path:05200_26
T47D	NFIC	path:05200_18
T47D	NFIC	path:05200_39
T47D	NFIC	path:05200_37
T47D	NFIC	path:05200_15
T47D	NFIC	path:05200_21
T47D	NFIC	path:05214_3
T47D	NFIC	path:05218_1
T47D	NFIC	path:05218_3
T47D	NFIC	path:05218_2
T47D	NFIC	path:05219_3
T47D	NFIC	path:05220_4
T47D	SP1	path:00140_1

**T47D**	**SP1**	**path:00330_3**
**T47D**	**SP1**	**path:00330_4**
**T47D**	**SP1**	**path:00330_1**
**T47D**	**SP1**	**path:00330_5**
**T47D**	**SP1**	**path:00350_2**
**T47D**	**SP1**	**path:00590_2**
**T47D**	**SP1**	**path:00980_1**
**T47D**	**SP1**	**path:04010_6**
**T47D**	**SP1**	**path:04010_8**
**T47D**	**SP1**	**path:04010_7**
**T47D**	**SP1**	**path:04060_19**
**T47D**	**SP1**	**path:04060_32**
**T47D**	**SP1**	**path:04060_20**
**T47D**	**SP1**	**path:04060_10**
**T47D**	**SP1**	**path:04060_46**
**T47D**	**SP1**	**path:04110_7**
**T47D**	**SP1**	**path:04110_9**
**T47D**	**SP1**	**path:04110_13**
**T47D**	**SP1**	**path:04110_4**
**T47D**	**SP1**	**path:04110_10**
**T47D**	**SP1**	**path:04110_11**
**T47D**	**SP1**	**path:04110_5**
**T47D**	**SP1**	**path:04110_2**
**T47D**	**SP1**	**path:04110_1**
**T47D**	**SP1**	**path:04115_2**
**T47D**	**SP1**	**path:04144_2**
**T47D**	**SP1**	**path:04210_5**
**T47D**	**SP1**	**path:04210_4**
**T47D**	**SP1**	**path:04210_9**
**T47D**	**SP1**	**path:04210_6**
**T47D**	**SP1**	**path:04310_7**
**T47D**	**SP1**	**path:04310_5**
**T47D**	**SP1**	**path:04350_5**
**T47D**	**SP1**	**path:04350_4**
**T47D**	**SP1**	**path:04350_1**
**T47D**	**SP1**	**path:04510_4**
**T47D**	**SP1**	**path:04510_14**
**T47D**	**SP1**	**path:04510_12**
**T47D**	**SP1**	**path:04510_6**
**T47D**	**SP1**	**path:04510_13**
**T47D**	**SP1**	**path:04510_7**
**T47D**	**SP1**	**path:04510_19**
**T47D**	**SP1**	**path:04510_5**
**T47D**	**SP1**	**path:04510_3**

T47D	SP1	path:04510_17
T47D	SP1	path:04510_10
T47D	SP1	path:04510_16
T47D	SP1	path:04510_1
T47D	SP1	path:04512_2
T47D	SP1	path:04512_3
T47D	SP1	path:04512_1
T47D	SP1	path:04520_2
T47D	SP1	path:04520_5
T47D	SP1	path:04540_7
T47D	SP1	path:04610_1
T47D	SP1	path:04610_7
T47D	SP1	path:04610_2
T47D	SP1	path:04610_5
T47D	SP1	path:04610_4
T47D	SP1	path:04610_6
T47D	SP1	path:04610_3
T47D	SP1	path:04620_9
T47D	SP1	path:04620_8
T47D	SP1	path:04620_6
T47D	SP1	path:04620_5
T47D	SP1	path:04630_2
T47D	SP1	path:04630_1
T47D	SP1	path:04650_3
T47D	SP1	path:04660_9
T47D	SP1	path:04662_9
T47D	SP1	path:04722_15
T47D	SP1	path:04810_16
T47D	SP1	path:04912_7
T47D	SP1	path:04940_2
T47D	SP1	path:05014_2
T47D	SP1	path:05014_1
T47D	SP1	path:05020_2
T47D	SP1	path:05020_1
T47D	SP1	path:05100_6
T47D	SP1	path:05140_4
T47D	SP1	path:05140_6
T47D	SP1	path:05142_11
T47D	SP1	path:05142_12
T47D	SP1	path:05142_3
T47D	SP1	path:05145_8
T47D	SP1	path:05145_6
T47D	SP1	path:05146_1
T47D	SP1	path:05200_31

T47D	SP1	path:05200_7
T47D	SP1	path:05200_39
T47D	SP1	path:05200_20
T47D	SP1	path:05200_37
T47D	SP1	path:05200_6
T47D	SP1	path:05200_19
T47D	SP1	path:05200_35
T47D	SP1	path:05200_42
T47D	SP1	path:05200_8
T47D	SP1	path:05200_1
T47D	SP1	path:05200_38
T47D	SP1	path:05200_25
T47D	SP1	path:05200_16
T47D	SP1	path:05200_21
T47D	SP1	path:05200_24
T47D	SP1	path:05200_9
T47D	SP1	path:05200_33
T47D	SP1	path:05200_13
T47D	SP1	path:05200_30
T47D	SP1	path:05200_2
T47D	SP1	path:05200_18
T47D	SP1	path:05200_29
T47D	SP1	path:05200_34
T47D	SP1	path:05200_15
T47D	SP1	path:05200_41
T47D	SP1	path:05200_10
T47D	SP1	path:05200_32
T47D	SP1	path:05200_14
T47D	SP1	path:05200_26
T47D	SP1	path:05200_17
T47D	SP1	path:05210_5
T47D	SP1	path:05210_8
T47D	SP1	path:05212_3
T47D	SP1	path:05212_9
T47D	SP1	path:05212_8
T47D	SP1	path:05212_10
T47D	SP1	path:05214_2
T47D	SP1	path:05214_1
T47D	SP1	path:05214_3
T47D	SP1	path:05215_4
T47D	SP1	path:05215_10
T47D	SP1	path:05215_9
T47D	SP1	path:05215_6
T47D	SP1	path:05215_7

T47D	SP1	path:05215_5
T47D	SP1	path:05216_3
T47D	SP1	path:05218_4
T47D	SP1	path:05218_1
T47D	SP1	path:05218_2
T47D	SP1	path:05218_3
T47D	SP1	path:05218_5
T47D	SP1	path:05219_3
T47D	SP1	path:05219_5
T47D	SP1	path:05220_5
T47D	SP1	path:05220_4
T47D	SP1	path:05220_8
T47D	SP1	path:05221_2
T47D	SP1	path:05222_5
T47D	SP1	path:05222_2
T47D	SP1	path:05222_1
T47D	SP1	path:05223_3
T47D	SP1	path:05223_1
T47D	SP1	path:05320_2
T47D	SP1	path:05330_2
T47D	SP1	path:05332_1
T47D	SP1	path:05414_2
T98	HIF1A	path:05200_37
T98	HIF1A	path:05200_39
T98	HIF1A	path:05200_25
T98	NFIC	path:00140_1
T98	NFIC	path:00591_1
T98	NFIC	path:04010_22
T98	NFIC	path:04080_5
T98	NFIC	path:04115_2
T98	NFIC	path:04510_6
T98	NFIC	path:04512_2
T98	NFIC	path:04660_10
T98	NFIC	path:04660_9
T98	NFIC	path:04662_5
T98	NFIC	path:04662_9
T98	NFIC	path:04722_15
T98	NFIC	path:05200_24
T98	NFIC	path:05200_7
T98	NFIC	path:05200_21
T98	NFIC	path:05200_25
T98	NFIC	path:05200_26
T98	NFIC	path:05200_13
T98	NFIC	path:05200_2

T98	NFIC	path:05200_6
T98	NFIC	path:05200_18
T98	NFIC	path:05200_39
T98	NFIC	path:05200_37
T98	NFIC	path:05200_15
T98	NFIC	path:05214_3
T98	NFIC	path:05218_3
T98	NFIC	path:05218_2
T98	NFIC	path:05218_1
T98	NFIC	path:05219_3
T98	NFIC	path:05220_4
T-cell	EGR1	path:04144_2
T-cell	EGR1	path:04350_5
T-cell	EGR1	path:04350_1
T-cell	EGR1	path:04510_6
T-cell	EGR1	path:04510_7
T-cell	EGR1	path:04510_5
T-cell	EGR1	path:04510_12
T-cell	EGR1	path:04510_13
T-cell	EGR1	path:04512_2
T-cell	EGR1	path:04512_1
T-cell	EGR1	path:04512_3
T-cell	EGR1	path:05020_2
T-cell	EGR1	path:05200_24
T-cell	EGR1	path:05200_29
T-cell	EGR1	path:05200_37
T-cell	EGR1	path:05200_35
T-cell	EGR1	path:05200_16
T-cell	EGR1	path:05200_31
T-cell	EGR1	path:05200_30
T-cell	EGR1	path:05200_25
T-cell	EGR1	path:05212_3
T-cell	EGR1	path:05212_8
T-cell	EGR1	path:05215_7
T-cell	EGR1	path:05215_9
T-cell	EGR1	path:05218_4
T-cell	ETS1	path:04060_46
T-cell	ETS1	path:04610_4
T-cell	ETS1	path:04610_1
T-cell	ETS1	path:04610_3
T-cell	ETS1	path:04610_2
T-cell	ETS1	path:04612_4
T-cell	ETS1	path:04620_9
T-cell	ETS1	path:04620_5

**T-cell**	**ETS1**	**path:04620_6**
**T-cell**	**ETS1**	**path:04620_1**
**T-cell**	**ETS1**	**path:04620_3**
**T-cell**	**ETS1**	**path:05212_3**
**T-cell**	**ETS1**	**path:05212_1**
**T-cell**	**ETS1**	**path:05212_2**
**T-cell**	**ETS1**	**path:05212_8**
**T-cell**	**ETS1**	**path:05212_6**
**T-cell**	**ETS1**	**path:05215_4**
**T-cell**	**ETS1**	**path:05215_9**
**T-cell**	**LEF1**	**path:04310_5**
**T-cell**	**LEF1**	**path:04310_7**
**T-cell**	**LEF1**	**path:04916_6**
**T-cell**	**LEF1**	**path:04916_3**
**T-cell**	**LEF1**	**path:04916_8**
**T-cell**	**LEF1**	**path:05200_18**
**T-cell**	**LEF1**	**path:05200_34**
**T-cell**	**LEF1**	**path:05200_35**
**T-cell**	**LEF1**	**path:05200_25**
**T-cell**	**LEF1**	**path:05200_21**
**T-cell**	**LEF1**	**path:05200_26**
**T-cell**	**LEF1**	**path:05200_15**
**T-cell**	**LEF1**	**path:05200_37**
**T-cell**	**LEF1**	**path:05200_39**
**T-cell**	**LEF1**	**path:05200_38**
**T-cell**	**LEF1**	**path:05210_5**
**T-cell**	**LEF1**	**path:05210_8**
**T-cell**	**LEF1**	**path:05213_1**
**T-cell**	**LEF1**	**path:05215_5**
**T-cell**	**LEF1**	**path:05216_3**
**T-cell**	**LEF1**	**path:05221_2**
**T-cell**	**LEF1**	**path:05222_5**
**T-cell**	**LEF1**	**path:05222_1**
**T-cell**	**NFATC1**	**path:04060_20**
**T-cell**	**NFATC1**	**path:04060_34**
**T-cell**	**NFATC1**	**path:04060_19**
**T-cell**	**SPI1**	**path:04060_26**
**T-cell**	**SPI1**	**path:04145_1**
**T-cell**	**SPI1**	**path:04145_4**
**T-cell**	**SPI1**	**path:04620_3**
**T-cell**	**SPI1**	**path:04620_4**
**T-cell**	**SPI1**	**path:04620_1**
**T-cell**	**SPI1**	**path:04650_1**
**T-cell**	**SPI1**	**path:04650_7**

T-cell	SPI1	path:04670_8
T-cell	SPI1	path:04670_4
T-cell	SPI1	path:04670_6
T-cell	SPI1	path:04670_9
T-cell	SPI1	path:04670_2
T-cell	SPI1	path:04810_16
T-cell	SPI1	path:05140_2
T-cell	SPI1	path:05140_3
T-cell	SPI1	path:05146_1
T-cell	SPI1	path:05150_4
T-cell	SPI1	path:05200_34
testis	AKNA	path:04060_18
testis	AKNA	path:04514_8
testis	AKNA	path:04672_2
testis	AKNA	path:05144_4
testis	AKNA	path:05145_14
testis	AKNA	path:05310_1
testis	AKNA	path:05320_1
testis	AKNA	path:05322_3
testis	AKNA	path:05330_1
testis	AKNA	path:05416_3
testis	CREB1	path:04514_19
testis	CREB1	path:04612_3
testis	CREB1	path:04620_9
testis	CREB1	path:04620_5
testis	CREB1	path:04620_6
testis	CREB1	path:04620_8
testis	CREB1	path:04620_4
testis	CREB1	path:05142_3
testis	ELK1	path:04010_11
testis	ELK1	path:04010_4
testis	ELK1	path:05020_3
testis	ELK1	path:05020_4
testis	ELK1	path:05110_3
testis	ELK1	path:05200_26
testis	ELK1	path:05200_18
testis	HNF4A	path:00010_1
testis	HNF4A	path:00120_1
testis	HNF4A	path:00140_1
testis	HNF4A	path:00561_2
testis	HNF4A	path:00590_1
testis	HNF4A	path:00590_2
testis	HNF4A	path:00591_1
testis	HNF4A	path:00830_1

**testis**	**HNF4A**	**path:00980_1**
**testis**	**HNF4A**	**path:00982_2**
**testis**	**HNF4A**	**path:03320_1**
**testis**	**HNF4A**	**path:04610_7**
**testis**	**HNF4A**	**path:04610_6**
**testis**	**HNF4A**	**path:04610_1**
**testis**	**HNF4A**	**path:04610_2**
**testis**	**HNF4A**	**path:04610_3**
**testis**	**HNF4A**	**path:04610_5**
**testis**	**HNF4A**	**path:04610_4**
**testis**	**HNF4A**	**path:04950_2**
**testis**	**HNF4A**	**path:04950_3**
**testis**	**KLF5**	**path:05200_35**
**testis**	**KLF5**	**path:05200_25**
**testis**	**KLF5**	**path:05200_37**
**testis**	**KLF5**	**path:05200_21**
**testis**	**KLF5**	**path:05210_5**
**testis**	**KLF5**	**path:05210_8**
**testis**	**SPI1**	**path:04060_26**
**testis**	**SPI1**	**path:04145_4**
**testis**	**SPI1**	**path:04145_1**
**testis**	**SPI1**	**path:04620_3**
**testis**	**SPI1**	**path:04620_4**
**testis**	**SPI1**	**path:04620_1**
**testis**	**SPI1**	**path:04650_7**
**testis**	**SPI1**	**path:04650_1**
**testis**	**SPI1**	**path:04670_4**
**testis**	**SPI1**	**path:04670_9**
**testis**	**SPI1**	**path:04670_8**
**testis**	**SPI1**	**path:04670_6**
**testis**	**SPI1**	**path:04670_2**
**testis**	**SPI1**	**path:04810_16**
**testis**	**SPI1**	**path:05140_2**
**testis**	**SPI1**	**path:05140_3**
**testis**	**SPI1**	**path:05146_1**
**testis**	**SPI1**	**path:05150_4**
**testis**	**SPI1**	**path:05200_34**
**testis**	**TP53**	**path:04010_11**
**testis**	**TP53**	**path:04012_1**
**testis**	**TP53**	**path:04012_5**
**testis**	**TP53**	**path:04110_2**
**testis**	**TP53**	**path:04110_13**
**testis**	**TP53**	**path:04110_7**
**testis**	**TP53**	**path:04110_9**

testis	TP53	path:04110_11
testis	TP53	path:04110_6
testis	TP53	path:04110_1
testis	TP53	path:04110_5
testis	TP53	path:04110_10
testis	TP53	path:04115_2
testis	TP53	path:04210_9
testis	TP53	path:04210_8
testis	TP53	path:04360_3
testis	TP53	path:04510_6
testis	TP53	path:04510_19
testis	TP53	path:04510_4
testis	TP53	path:04510_13
testis	TP53	path:04510_12
testis	TP53	path:04650_3
testis	TP53	path:04722_15
testis	TP53	path:04912_7
testis	TP53	path:05014_2
testis	TP53	path:05160_10
testis	TP53	path:05160_9
testis	TP53	path:05200_37
testis	TP53	path:05200_6
testis	TP53	path:05200_10
testis	TP53	path:05200_15
testis	TP53	path:05200_13
testis	TP53	path:05200_24
testis	TP53	path:05200_3
testis	TP53	path:05200_2
testis	TP53	path:05200_9
testis	TP53	path:05200_7
testis	TP53	path:05200_20
testis	TP53	path:05200_14
testis	TP53	path:05200_25
testis	TP53	path:05200_26
testis	TP53	path:05200_38
testis	TP53	path:05200_16
testis	TP53	path:05200_8
testis	TP53	path:05200_1
testis	TP53	path:05200_17
testis	TP53	path:05200_18
testis	TP53	path:05200_19
testis	TP53	path:05200_21
testis	TP53	path:05200_39
testis	TP53	path:05213_3

testis	TP53	path:05214_1
testis	TP53	path:05214_2
testis	TP53	path:05214_3
testis	TP53	path:05215_6
testis	TP53	path:05215_4
testis	TP53	path:05215_7
testis	TP53	path:05215_9
testis	TP53	path:05215_10
testis	TP53	path:05218_1
testis	TP53	path:05218_3
testis	TP53	path:05218_2
testis	TP53	path:05218_5
testis	TP53	path:05219_5
testis	TP53	path:05219_3
testis	TP53	path:05220_4
testis	TP53	path:05223_2
thymus	AKNA	path:04060_18
thymus	AKNA	path:04514_8
thymus	AKNA	path:04672_2
thymus	AKNA	path:05144_4
thymus	AKNA	path:05145_14
thymus	AKNA	path:05310_1
thymus	AKNA	path:05320_1
thymus	AKNA	path:05322_3
thymus	AKNA	path:05330_1
thymus	AKNA	path:05416_3
thymus	CREB1	path:04514_19
thymus	CREB1	path:04612_3
thymus	CREB1	path:04620_9
thymus	CREB1	path:04620_6
thymus	CREB1	path:04620_5
thymus	CREB1	path:04620_8
thymus	CREB1	path:04620_4
thymus	CREB1	path:05142_3
thymus	ELF1	path:00920_1
thymus	ELF1	path:04060_34
thymus	ELF1	path:04060_1
thymus	ELF1	path:04650_8
thymus	ELF1	path:04650_2
thymus	ELF1	path:04650_4
thymus	ELF1	path:04650_10
thymus	ELF1	path:04650_9
thymus	ELF1	path:04664_1
thymus	ELF1	path:04664_6

thymus	ELF1	path:04664_2
thymus	FLI1	path:04512_1
thymus	FLI1	path:04512_3
thymus	IRF5	path:04060_23
thymus	IRF5	path:04620_1
thymus	IRF5	path:04620_3
thymus	IRF5	path:04620_4
thymus	IRF5	path:04620_2
thymus	IRF5	path:04620_7
thymus	IRF5	path:04650_5
thymus	IRF5	path:04650_3
thymus	IRF5	path:05160_4
thymus	IRF7	path:04060_23
thymus	IRF7	path:04620_6
thymus	IRF7	path:04620_4
thymus	IRF7	path:04620_7
thymus	IRF7	path:04620_8
thymus	IRF7	path:04620_1
thymus	IRF7	path:04620_9
thymus	IRF7	path:04620_3
thymus	IRF7	path:04620_5
thymus	IRF7	path:04620_2
thymus	IRF7	path:04650_5
thymus	IRF7	path:04650_3
thymus	IRF7	path:05160_4
thymus	LEF1	path:04310_5
thymus	LEF1	path:04310_7
thymus	LEF1	path:04916_6
thymus	LEF1	path:04916_8
thymus	LEF1	path:04916_3
thymus	LEF1	path:05200_21
thymus	LEF1	path:05200_35
thymus	LEF1	path:05200_34
thymus	LEF1	path:05200_37
thymus	LEF1	path:05200_25
thymus	LEF1	path:05200_39
thymus	LEF1	path:05200_38
thymus	LEF1	path:05200_15
thymus	LEF1	path:05200_26
thymus	LEF1	path:05200_18
thymus	LEF1	path:05210_5
thymus	LEF1	path:05210_8
thymus	LEF1	path:05213_1
thymus	LEF1	path:05215_5

**thymus**	**LEF1**	**path:05216_3**
**thymus**	**LEF1**	**path:05221_2**
**thymus**	**LEF1**	**path:05222_5**
**thymus**	**LEF1**	**path:05222_1**
**thymus**	**SPI1**	**path:04060_26**
**thymus**	**SPI1**	**path:04145_4**
**thymus**	**SPI1**	**path:04145_1**
**thymus**	**SPI1**	**path:04620_3**
**thymus**	**SPI1**	**path:04620_4**
**thymus**	**SPI1**	**path:04620_1**
**thymus**	**SPI1**	**path:04650_7**
**thymus**	**SPI1**	**path:04650_1**
**thymus**	**SPI1**	**path:04670_4**
**thymus**	**SPI1**	**path:04670_8**
**thymus**	**SPI1**	**path:04670_9**
**thymus**	**SPI1**	**path:04670_6**
**thymus**	**SPI1**	**path:04670_2**
**thymus**	**SPI1**	**path:04810_16**
**thymus**	**SPI1**	**path:05140_2**
**thymus**	**SPI1**	**path:05140_3**
**thymus**	**SPI1**	**path:05146_1**
**thymus**	**SPI1**	**path:05150_4**
**thymus**	**SPI1**	**path:05200_34**
**thymus**	**TP63**	**path:04110_11**
**thymus**	**TP63**	**path:04110_7**
**thymus**	**TP63**	**path:04110_5**
**thymus**	**TP63**	**path:04110_10**
**thymus**	**TP63**	**path:04110_2**
**thymus**	**TP63**	**path:04110_1**
**thymus**	**TP63**	**path:04110_9**
**thymus**	**TP63**	**path:04115_2**
**thymus**	**TP63**	**path:05200_19**
**thymus**	**TP63**	**path:05200_25**
**thymus**	**TP63**	**path:05200_39**
**thymus**	**TP63**	**path:05200_9**
**thymus**	**TP63**	**path:05200_21**
**thymus**	**TP63**	**path:05200_7**
**thymus**	**TP63**	**path:05200_14**
**thymus**	**TP63**	**path:05200_15**
**thymus**	**TP63**	**path:05200_2**
**thymus**	**TP63**	**path:05200_13**
**thymus**	**TP63**	**path:05200_3**
**thymus**	**TP63**	**path:05200_20**
**thymus**	**TP63**	**path:05200_37**

thymus	TP63	path:05200_1
thymus	TP63	path:05214_3
thymus	TP63	path:05215_4
thymus	TP63	path:05215_6
thymus	TP63	path:05215_9
thymus	TP63	path:05215_10
thymus	TP63	path:05218_5
thymus	TP63	path:05218_3
thymus	TP63	path:05219_3
thymus	TP63	path:05219_5
thymus	TP63	path:05220_4
thyroid	HNF1A	path:00140_1
thyroid	HNF1A	path:00980_1
thyroid	HNF1A	path:00982_2
thyroid	HNF1A	path:04610_2
thyroid	HNF1A	path:04610_6
thyroid	HNF1A	path:04610_3
thyroid	HNF1A	path:04610_4
thyroid	HNF1A	path:04950_3
thyroid	NR4A1	path:00140_1
trachea	TP63	path:04110_11
trachea	TP63	path:04110_5
trachea	TP63	path:04110_7
trachea	TP63	path:04110_1
trachea	TP63	path:04110_10
trachea	TP63	path:04110_9
trachea	TP63	path:04110_2
trachea	TP63	path:04115_2
trachea	TP63	path:05200_39
trachea	TP63	path:05200_19
trachea	TP63	path:05200_25
trachea	TP63	path:05200_9
trachea	TP63	path:05200_7
trachea	TP63	path:05200_15
trachea	TP63	path:05200_14
trachea	TP63	path:05200_21
trachea	TP63	path:05200_13
trachea	TP63	path:05200_1
trachea	TP63	path:05200_20
trachea	TP63	path:05200_3
trachea	TP63	path:05200_2
trachea	TP63	path:05200_37
trachea	TP63	path:05214_3
trachea	TP63	path:05215_4

trachea	TP63	path:05215_6
trachea	TP63	path:05215_10
trachea	TP63	path:05215_9
trachea	TP63	path:05218_3
trachea	TP63	path:05218_5
trachea	TP63	path:05219_3
trachea	TP63	path:05219_5
trachea	TP63	path:05220_4
trophoblast	GATA2	path:04060_32
trophoblast	GATA3	path:04612_4
U251	HIF1A	path:05200_37
U251	HIF1A	path:05200_39
U251	HIF1A	path:05200_25
U251	NFIC	path:00140_1
U251	NFIC	path:00591_1
U251	NFIC	path:04010_22
U251	NFIC	path:04080_5
U251	NFIC	path:04115_2
U251	NFIC	path:04510_6
U251	NFIC	path:04512_2
U251	NFIC	path:04660_10
U251	NFIC	path:04660_9
U251	NFIC	path:04662_9
U251	NFIC	path:04662_5
U251	NFIC	path:04722_15
U251	NFIC	path:05200_7
U251	NFIC	path:05200_2
U251	NFIC	path:05200_24
U251	NFIC	path:05200_13
U251	NFIC	path:05200_25
U251	NFIC	path:05200_6
U251	NFIC	path:05200_26
U251	NFIC	path:05200_18
U251	NFIC	path:05200_37
U251	NFIC	path:05200_15
U251	NFIC	path:05200_21
U251	NFIC	path:05200_39
U251	NFIC	path:05214_3
U251	NFIC	path:05218_1
U251	NFIC	path:05218_2
U251	NFIC	path:05218_3
U251	NFIC	path:05219_3
U251	NFIC	path:05220_4
UT-7	GATA1	path:04060_32

uterus	TP63	path:04110_5
uterus	TP63	path:04110_9
uterus	TP63	path:04110_1
uterus	TP63	path:04110_2
uterus	TP63	path:04110_11
uterus	TP63	path:04110_10
uterus	TP63	path:04110_7
uterus	TP63	path:04115_2
uterus	TP63	path:05200_2
uterus	TP63	path:05200_39
uterus	TP63	path:05200_9
uterus	TP63	path:05200_37
uterus	TP63	path:05200_7
uterus	TP63	path:05200_19
uterus	TP63	path:05200_1
uterus	TP63	path:05200_15
uterus	TP63	path:05200_13
uterus	TP63	path:05200_3
uterus	TP63	path:05200_14
uterus	TP63	path:05200_20
uterus	TP63	path:05200_25
uterus	TP63	path:05200_21
uterus	TP63	path:05214_3
uterus	TP63	path:05215_4
uterus	TP63	path:05215_6
uterus	TP63	path:05215_10
uterus	TP63	path:05215_9
uterus	TP63	path:05218_3
uterus	TP63	path:05218_5
uterus	TP63	path:05219_5
uterus	TP63	path:05219_3
uterus	TP63	path:05220_4

Table S10. Real vs. random Jaccard coefficient of the TSN with k=4 at the every pathway level

	Subpathway	Pathway	Pathway Class
**common-motif**	**0.0705 vs. 0.0628**	**0.1067 vs 0.0860**	**0.3864 vs 0.3706**
	**(*p* = 0.0020)**	**(*p* < 1.0e-6))**	**(*p* = 0.0075)**
**common-family**	**0.1406 vs 0.0099**	**0.1727 vs 0.0893**	**0.3745 vs 0.2981**
	**(*p* < 1.0e-6)),**	**(*p* < 1.0e-6))**	**(*p* < 1.0e-6))**
**common-tissue**	**0.0486 vs 0.0088**	**0.0790 vs 0.0769**	**0.4086 vs 0.2956**
	**(*p* < 1.0e-6))**	**(*p* < 1.0e-6))**	**(*p* < 1.0e-6))**
